# Comparative Genomic Analysis of *Rhodococcus equi*: An Insight into Genomic Diversity and Genome Evolution

**DOI:** 10.1155/2019/8987436

**Published:** 2019-12-18

**Authors:** Jianchao Ying, Jun Ye, Teng Xu, Qian Wang, Qiyu Bao, Aifang Li

**Affiliations:** ^1^Central Laboratory, Institute of Emergency Medicine, The First Affiliated Hospital of Wenzhou Medical University, Wenzhou, China; ^2^Key Laboratory of Laboratory Medicine, Ministry of Education, School of Laboratory Medicine and Life Sciences, Wenzhou Medical University, Wenzhou, China; ^3^Department of Clinical Laboratory, The Second Affiliated Hospital of Guizhou Medical University, Kaili, China; ^4^Institute of Translational Medicine, Baotou Central Clinical Hospital of Inner Mogolia Medical University, Baotou, China; ^5^Department of Clinical Laboratory, Wenzhou People's Hospital, The Third Clinical Institute Affiliated to Wenzhou Medical University, Wenzhou, China; ^6^The Fifth Affiliated Hospital of Wenzhou Medical University, Lishui, China

## Abstract

*Rhodococcus equi*, a member of the *Rhodococcus* genus, is a gram-positive pathogenic bacterium. *Rhodococcus* possesses an open pan-genome that constitutes the basis of its high genomic diversity and allows for adaptation to specific niche conditions and the changing host environments. Our analysis further showed that the core genome of *R. equi* contributes to the pathogenicity and niche adaptation of *R. equi*. Comparative genomic analysis revealed that the genomes of *R. equi* shared identical collinearity relationship, and heterogeneity was mainly acquired by means of genomic islands and prophages. Moreover, genomic islands in *R. equi* were always involved in virulence, resistance, or niche adaptation and possibly working with prophages to cause the majority of genome expansion. These findings provide an insight into the genomic diversity, evolution, and structural variation of *R. equi* and a valuable resource for functional genomic studies.

## 1. Introduction

*Rhodococcus* is a genus of aerobic, nonsporulating, and nonmotile actinomycetes that is closely related to *Nocardia*, *Mycobacterium*, and *Corynebacterium* [1, 2]. It comprises more than 50 species that are widely distributed in a broad range of environments, including soil, water, and eukaryotic cells. Most species are benign and assumed to be important for industries [3, 4], while a few species are pathogenic, including the plant pathogen *Rhodococcus fascians* [5] and the animal pathogen *Rhodococcus equi* [6]. *R. equi* is commonly found in dry and dusty soil and is a multihost pathogen that causes purulent infections in various animal species [7]. It causes chronic pyogranulomatous adenitis in animals (horses, goats, pigs, and cattle) [6]. In addition, *R. equi* can infect immunocompromised humans such as HIV-AIDS patients or transplant recipients, resulting in severe opportunistic infections [7]. The pathogenic *R. equi* harbors a virulence plasmid, which confers the ability to survive and replicate within host macrophages. Furthermore, the virulence-associated proteins (named VapA/B/N), which are major plasmid-encoded surface antigens located within the virulence plasmid, have been shown to be essential for virulence [8–11].

Previous studies [7, 12–14] have focused on *R. equi*'s genomic constitution, annotation, and classification of respective isolates, as well as reconstructed its phylogenetic relationship with other different strains or species. Previous studies have also provided information about the determinants related to virulence and niche adaptation in *R. equi* [7] and performed pan-genome analysis of *R. equi* and discussed the importance of core genome in physiology, virulence, and niche adaptation [14]. *R. equi* genome lacks a substantial signature of host adaptation as previously reported [12]. Therefore, apart from the importance of the virulence plasmid, the knowledge of the role of chromosome genome in virulence, resistance, and niche adaptation (including host and environmental derivations) is insufficient. A clinical strain designated *R. equi* WY was isolated from the sputum of a patient with pulmonary infection in Lishui of China. This work determined the complete genomic sequence of *R. equi* strain WY and analyzed the genetic basis of adaptation of *R. equi* to various niches. We also conducted an evolutionary and comparative analysis among the *Rhodococcus* genus and *R. equi* to gain insight into the genomic diversity and phylogenetic relationship.

## 2. Results and Discussion

### 2.1. General Genomic Information and Diversity of *Rhodococcus* Genus

For this work, 94 *Rhodococcus* whole-genome sequences covering 22 explicit species were selected (Supplementary Tables [Supplementary-material supplementary-material-1] and [Supplementary-material supplementary-material-1]). Among these, three species had the largest number of sequenced strains: *R. fascians* [15], *R. erythropolis* [16], and *R. equi* [9], while 11 species only had one sequenced strain. The *Rhodococcus* spp. were isolated from different sources, such as soil and seawater with *R. erythropolis*, plant with *R. fascians*, and animal host (e.g., equine, swine, and human) and soil with *R. equi*. The complete genome sequence of *R. equi* WY was determined in this study and was also included in Supplementary [Supplementary-material supplementary-material-1]. *R. equi* WY has a circular 5.13 Mb chromosome with an average GC content of 68.76%, as well as a circular 82 kb plasmid. The genome size of *R. equi* WY is larger than the previously sequenced 103S (5.04 Mb) and DSM 20295 (4.97 Mb) but smaller than the other six genomes (5.17-5.26 Mb). We identified 4781 putative open reading frames (ORFs), and about half of them (2344/4781) are forward strand genes (Supplementary [Supplementary-material supplementary-material-1]). Nearly three-quarters of them are predicted to encode proteins with known functions.

The genome sizes of the 22 *Rhodococcus* spp. varied from species to species (3.89-12.41 Mb). *R. wratislaviensis* had the highest average genome size (9.77 Mb, 9.16-10.38 Mb); this is 5.8 Mb larger than that of *R. corynebacterioides* (3.89 Mb), which has the smallest genome (Supplementary [Supplementary-material supplementary-material-1]). Moreover, the GC content of the 94 genomes ranged from 61.67% to 70.67%. This enormous genome diversity suggests flexibility in the *Rhodococcus* genome and may have enabled them to adapt to a broad spectrum of environments (e.g., soil, plants, water, and animals).

**Figure 1:**
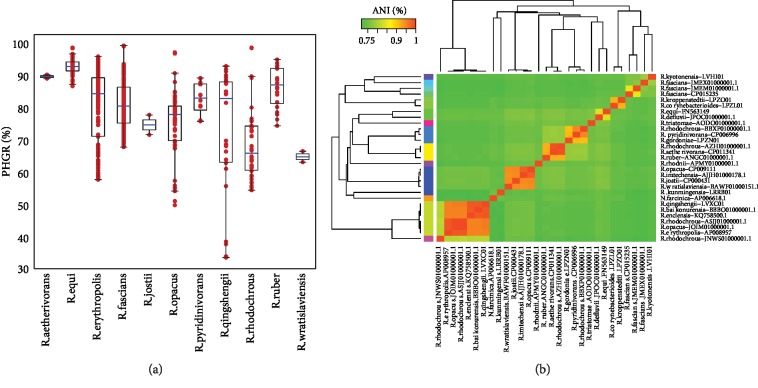
The genomic conservation of *Rhodococcus* species. (a) PHGR of *Rhodococcus* species. It was determined by summation of all homologous genes found in any pair of genomes divided by the total gene number of each genome and was shown as boxplots. Boxplots indicate 0.25 and 0.75 quantiles, and the blue lines represent the median values of PHGR of each species. (b) Clustering analysis of 28 representative *Rhodococcus* genomes and a closely related genome (*Nocardia farcinica*) based on the ANI matrix.

To evaluate conservation of different species within genus *Rhodococcus* (including no less than two genomes), the pairwise homologous gene rate (PHGR) was determined in each species by summation of all homologous genes found in any pair of genomes divided by the total gene number of each genome (Figure 1(a)). Our analysis showed that the average PHGR of each species varied from 65.16% to 93.01% across 11 *Rhodococcus* species. *R. equi* had the highest average PHGR (93.01%) and showed a high degree of conservation among the 11 species, while *R. wratislaviensis* had the lowest genome conservation (65.16%) (Figure 1(a)). Furthermore, the PHGR values of *R. qingshengii* ranged from 33.97% to 93.16%, and the values spanned greater than 50%, suggesting the presence of broad HGT events or extensive mutations during speciation which caused less genomic conservations among *R. qingshengii*. The average nucleotide identity (ANI) [16] was also calculated to infer the similarity between any pair of genomes. ANI values ranged from 71.702% to 99.997%, indicating high genomic diversity among *Rhodococcus* genomes (Supplementary Fig. [Supplementary-material supplementary-material-1]). Clustering analysis was performed using a 94% ANI value as a threshold for determining species-level groups. Most species had one cluster, with the exception of *R. rhodochrous*, *R. fascians*, and *R. opacus* with 4, 3, and 2 clusters, respectively. A total of 28 representative sequences of each species in each cluster (species-level groups) were obtained (Figure 1(b)). This finding indicates that unlike other species in genus *Rhodococcus*, *R. rhodochrous*, *R. fascians*, and *R. opacus* have relatively larger intraspecies genome divergence.

### 2.2. Pan-Genome Analysis of *Rhodococcus* and *R. equi*

Pan-genome analyses have shed light on the dynamics and evolution of bacterial genomes [17]. The pan/core genome curve of *Rhodococcus* based on 22 *Rhodococcus* species was created. The number of pan-genome genes has not yet reached saturation, even though there are more than 22 species. According to the Heaps' law model [18], the pan-genome is considered open [19], which is typical of species colonizing multiple environments and having multiple ways of exchanging genetic material [20], such as *Rhodococcus* species adapting to a wide variety of niches and having large genome diversity. In contrast to the pan-genome (40,911 pan genes) of the 22 *Rhodococcus* species, the number of the core genome converged to a relatively constant number of 796 (Supplementary Fig. [Supplementary-material supplementary-material-1]).

**Figure 2:**
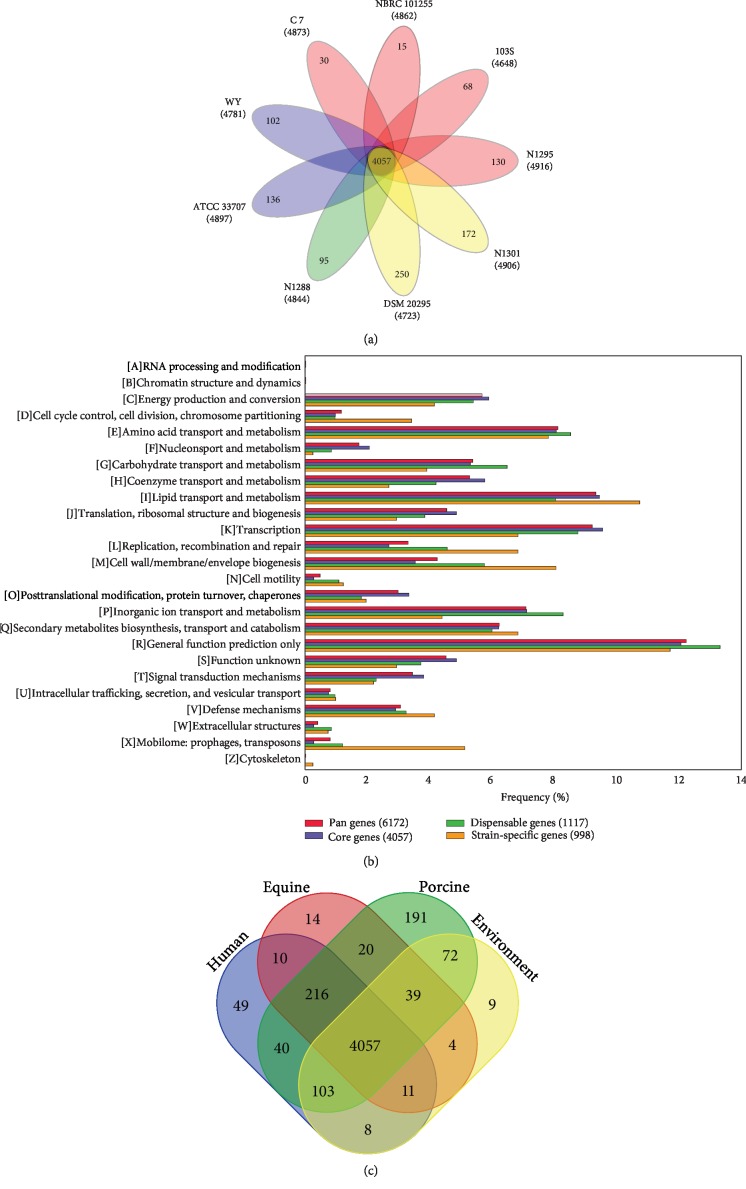
Pan-genome analysis of *R. equi*. (a) The core gene number (in the center) and strain-specific gene number (in the petals) in nine *Rhodococcus* strains as illustrated by a flower plot. (b) The COG function classification comparison of pan, core, dispensable, and strain-specific genes in *R. equi*. (c) Venn diagram of the four core genomes from four different sources (human, equine, porcine, and environment). The numbers represent the respective number of orthologous clusters. The petals in (a) are color-coded by source, and the colors are correlated with (c).

*R. equi* also displayed an open pan-genome and a relatively constant core genome (Supplementary Fig. [Supplementary-material supplementary-material-1]). Therefore, the analysis of core genes in *R. equi* will be relatively reliable. Similar situations also occurred in *R. erythropolis* and *R. fascians* (pan/core genome curves were shown in Supplementary Fig. [Supplementary-material supplementary-material-1]). Further analysis identified 4057 core genes in *R. equi*, accounting for 65.7% (4057/6172) of pan-genome. This ratio was higher than *R. erythropolis* (3853/17184; 22.4%), *R. fascians* (3192/12646; 25.2%), and other species of *Rhodococcus*. *R. equi* also contains 2115 accessory genes, including 1117 dispensable genes (genes present in two or more genomes [20]) and 998 strain-specific genes (strain-unique gene specific to single genome [20]). Among these, two strains of *R. equi* isolated from the environment have the most strain-specific genes, while only 15 strain-specific genes exist in NBRC 101255 isolated from equine, indicating that there are large differences in the genomes isolated from different sources (Figure 2(a)).

We also compared the COG function classification to further understand the functional differences of pan genes, core genes, dispensable genes, and strain-specific genes of *R. equi* (Figure 2(b)). Our analysis showed that the transport and metabolism system accounts for a high proportion of the pan-genome, which enables the efficient transport of substrates and products. There were also a higher proportion of genes related to transcription, translation, ribosomal structure, and biosynthesis and metabolism, which are essential for cell growth and/or the rapid and efficient response to nutrient environment sources. These abilities confer a survival advantage to changing environments. The majority of the core genes (49.86%) are related to transport- and metabolism-related functions and are higher in proportion than dispensable genes and strain-specific genes (47.78%, 40.83%). Compared with pan and core genes, dispensable and strain-specific genes contain a higher proportion of genes related to the cell envelope (5.76%, 8.05%), cell motility (1.08%, 1.22%), and mobilome (1.20%, 5.12%). The bacterial cell envelope is a complex multilayered structure that serves to protect these organisms from unpredictable and often hostile environment [21]. Motility confers bacteria an ability to choose favorable environment containing positive stimuli, light, gravity, or chemicals and avoid unfavorable conditions of habitat [22]. These functions are associated with host and environment interactions, suggesting that some accessory genes may also be involved in niche adaptation, and were probably gained through mobile genetic elements.

We identified a total of 376 virulence-related genes in the *R. equi* pan-genome, and 295 of them were significantly enriched in core genes (*P* < 0.05, Supplementary [Supplementary-material supplementary-material-1]). Less than 22% are in dispensable and strain-specific genes. Meanwhile, we also reconstructed the *R. equi* metabolic pathway using the pan-genome and found a significant enrichment of core genes not only in fundamental metabolic pathways (e.g., nitrogen metabolism, glyoxylate and dicarboxylate metabolism) but also in the two-component system (TCS), bacterial secretion system, and protein export (all FDR < 0.05, Supplementary [Supplementary-material supplementary-material-1]), suggesting the important role of TCS and protein transport in the pathogenicity of *R. equi*. TCSs, the predominant signal transduction pathways in bacteria, are essential for bacterial survival, growth, and development by enabling them to adapt to the environment [23]. Previous studies [15, 24] have revealed that TCSs are also involved in the virulence and antibiotic resistance of opportunistic bacterial pathogens. TCSs typically consist of a membrane-bound histidine kinase (HK), which senses a specific environmental stimulus, and a corresponding response regulator (RR), which mediates the cellular response, mostly through the regulation of differential expression of target genes [25]. Moreover, bacteria have also evolved a more intricate phosphorelay system in which an additional histidine phosphotransfer protein (PP) accepts the phosphoryl group from the first response regulator domain and transfers it to the second response regulator domain [26]. Further analysis identified 229 TCS proteins in the pan-genome and found that TCS genes were significantly enriched in core genes (*P* value < 0.05, Supplementary [Supplementary-material supplementary-material-1]). Interestingly, a similar situation was also observed in resistance genes. This finding supports the notion that the core genome may contribute to the niche adaptation and pathogenicity of *R. equi*.

The core genes and accessory genes of the four core genomes from four different sources (human, equine, porcine, and environment) were also identified to determine differences among different sources (Figure 2(c)). At least two genomes were obtained from each source except porcine. Among these, the core genome from the porcine source showed the most strain-specific genes (191). We compared the COG function classification of core genes according to each source and found no significant difference (Supplementary Fig. [Supplementary-material supplementary-material-1]). A previous study [12] has revealed a lack of a substantial signature of host adaptation in *R. equi*. However, many metabolic pathways were found in the core genes of those obtained from animals (216 animal-unique genes), such as transport metabolism function (e.g., energy production and conversion, amino acid transport and metabolism, and lipid transport and metabolism), cell wall/membrane/envelope biogenesis, cell motility, and signal transduction mechanisms (Supplementary [Supplementary-material supplementary-material-1]). With further analysis, we found 10 virulence-related genes (e.g., pilus assembly related the secretory protein kinase, fibronectin-binding protein A, and lipopolysaccharide biosynthesis protein) that are related to the animal pathogenicity of *R. equi* (Supplementary [Supplementary-material supplementary-material-1]). Moreover, 18 resistance genes (e.g., transpeptidase, major facilitator superfamily, chloramphenicol efflux pump, and universal stress protein), 5 TCS proteins (including 1 classic HK, 1 unclassified RR, and 3 HisKa-PP), and several transport proteins seem to be animal-unique. The presence of a large number of potential virulence, resistance, and TCS genes possibly confers the ability of *R. equi* to adapt to the animal niche. We also found the existence of the TCS protein (HisKa-PP), the potential resistance gene (belonging to the dioxygenase superfamily), and the heat shock protein in the human-unique gene [27], which may contribute to specific human host adaptation (Supplementary [Supplementary-material supplementary-material-1]).

### 2.3. Mobile Genetic Elements in *Rhodococcus* Genome

Mobile genetic elements (MGEs) are the major contributors to genome diversity and are responsible for the widespread horizontal gene transfers (HGT) and genome rearrangements [28]. Phages and transposons are predominant members of the MGE population, which play a central role in mobilizing and reorganizing genes within a given genome (intracellular mobility) or between bacterial cells (intercellular mobility) [28, 29]. Therefore, a comprehensive survey of the genomic MGEs (including transposase and phage-derived genes) of *Rhodococcus* spp. was conducted (Supplementary Fig. [Supplementary-material supplementary-material-1]). We found that transposase and phage were commonly presented in *Rhodococcus* genus, which indicated that the genomes of *Rhodococcus* spp. could be affected by HGT. Interestingly, all strains of *Rhodococcus* showed a high ratio of phage-derived genes (2.76%-7.50%), especially *R. equi* which has the highest rate at 6.48%-7.50% (except *R. equi* 103S) (Supplementary Fig. [Supplementary-material supplementary-material-1]). In contrast, there is a great difference among the ratios of transposase (0%-1.55%). In some strains (e.g., *R. ruber*, *R. opacus*, and *R. pyridinivorans*), the proportion of transposase was greater than 1%. But for *R. equi*, the overall proportion was lower, and only three strains (3/9) contained the transposase gene, implying that HGT occurred in the genome of *R. equi* mainly by means of phage.

### 2.4. Stable Core Genes and Phylogenetic Analysis of *Rhodococcus* and *R. equi*

**Figure 3:**
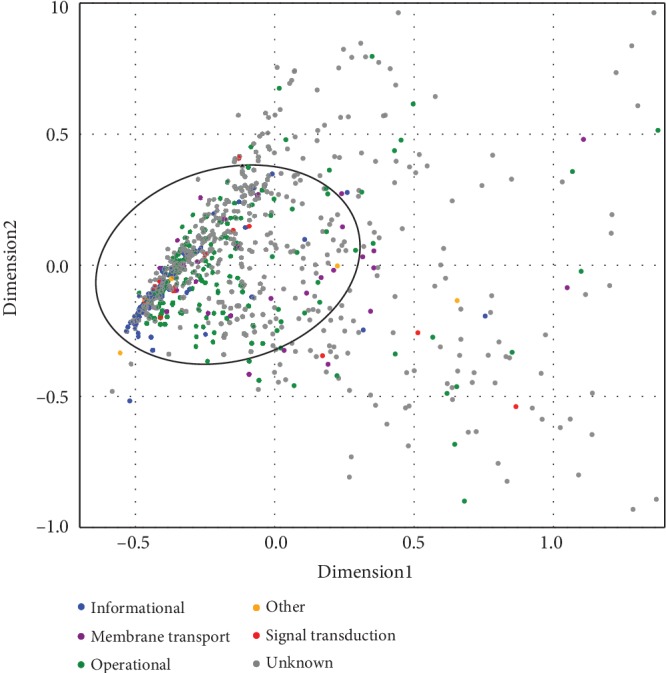
Plot of the two first axes of the PCoA made from 796 ML trees. The other 33 genes are excluded due to the limit of axis demarcation. The ellipse depicts 562 orthologs in the densest region (stable core region) of the cloud that share a common phylogenetic signal, whereas trees present in the marginal area (the shell) are much more likely to be perturbed by horizontal transfers. The genes are color-coded based on its association to biological pathways.

To extrapolate evolutionary trajectories of the *Rhodococcus* core genome, we constructed the phylogenetic trees based on amino acid sequences of 796 homologous protein families using the maximum likelihood (ML) method. We calculated tree distances among all possible pairs of the orthologous sets. The pairwise distances were then used to conduct a principal coordinate analysis (PCoA) (Figure 3). The results showed a set of 562 genes designated stable core that share similar evolutionary histories (coevolving and rarely transferred) as opposed to the other 234 (no more than 1/3) that exhibit divergent phylogenies (independently evolving and frequently transferred). This indicates that a majority of the core genome of *Rhodococcus* is relatively stable. In addition, we found that, although many genes are unknown, there are informational genes (transcription, translation, and related) and operational genes (metabolism and cellular processes) gathered in stable core region, especially genes involved in ribosome and biosynthesis of amino acids (FDR < 0.05; [Supplementary-material supplementary-material-1]). These findings are in line with a previous study, which revealed a highly conserved core set of ribosomal proteins in Cyanobacteria [30]. This suggests that transcription- and translation-related genes are so highly conserved, possibly resisting HGT. It is worth noting that some stable core genes were associated with membrane transport and signal transduction (TCS), suggesting that the stable core may also be involved in niche adaptation of *Rhodococcus*.

**Figure 4:**
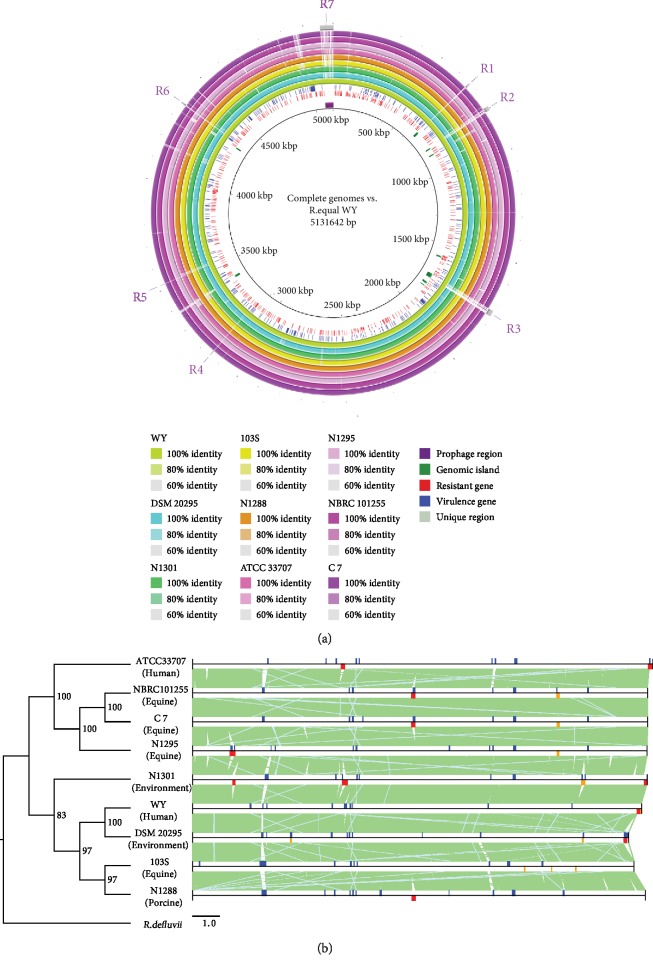
Comparative genomic analysis of nine *R. equi* strains. (a) The nucleotide sequence of *R. equi* WY was used as a reference (backbone) and compared with those of eight query genomes. Counting from the outside toward the center: gray color (slot 1) indicated unique regions in *R. equi* WY, which were labeled R1-R7. Slots 2-10 showed that the corresponding query regions had higher sequence identity (>60%) with the reference sequence. Empty regions on the query slots indicated parts without similar hits between the reference sequence and the query sequences. Labels for slots 2-14 are shown in the legend. (b) Phylogenetic analysis (left) and comparison of genome structure (right) of nine *R. equi* strains. Phylogenetic reconstruction was based on concatenated SNPs of nine *R. equi* genomes rooted with *R. defluvii*. The identical sequence regions are connected by light green bars, while nonidentical regions are left empty. The different functional elements are labeled in different colors, with genomic islands in blue, complete prophage regions in red, and incomplete prophage regions in orange.

We constructed the phylogenetic tree using the species-level groups determined by ANI analyses, based on the concatenated stable core proteins rather than 16s rDNA [31] or universal proteins [12, 14] which have been used in some previous phylogenetic analyses (Supplementary Fig. [Supplementary-material supplementary-material-1]). The phylogenetic relationship showed that most *Rhodococcus* species were grouped within five deep branches, while *R. kunmingensis* clustered more closely to the most recent common ancestor. *R. equi* clustered together with *R. defluvii* and *R. triatomae*, which indicated that *R. equi* might have a closer phylogenetic relationship with *R. defluvii* and *R. triatomae* than with other species as previously reported [13, 14] (Supplementary Fig. [Supplementary-material supplementary-material-1]). Consistent with the clustering result based on ANI aforementioned, the genomes from the same species were not fully clustered together, such as *R. rhodochrous* and *R. opacus* which were located in two separate clusters. We also constructed a phylogenetic tree based on SNP concatenated sequences of nine strains of *R. equi*, which was rooted by *R. defluvii* (Figure 4(b)). The results showed that some *R. equi* strains derived from the same source were not clustered together but mixed with strains isolated from different sources. These findings are consistent with a recent study [14] and demonstrated that there was no correlation in evolution between *R. equi* genome and its niche adaptation.

### 2.5. Comparative Genomic Analyses of *R. equi*

The most effective approach for identifying gene gains and losses is to conduct a direct molecular genetic analysis of DNA sequences [32]. Thus, to further characterize the differences among several *R. equi* genomes, we conducted a comparative genomic analysis of *R. equi* WY with other *R. equi* genomes (Figure 4). Comparative analysis showed that the nine *R. equi* genomes shared a highly consistent collinearity relationship and lacked significant chromosomal rearrangement. Furthermore, most of the genes in *R. equi* WY (85.37%) were shared with the other eight genomes. Compared to the other eight genomes, only seven regions (size larger than 2 kb) of *R. equi* WY were uniquely present in the strain WY, including a complete prophage region (Figure 4(a)). In addition, *R. equi* WY possessed several nonalignment regions, which mainly belonged to genomic islands and prophage regions related to HGT. This suggests that the diversity of *R. equi* genome probably resulted from HGT (Figure 4(b)).

### 2.6. Genomic Islands in *R. equi* WY

Genomic islands (GIs) are clusters of genes of probable horizontal origin in bacterial and archaeal genomes [33]. They play a significant role in the genome evolution of such microbes, encoding genes involved in adaptations of medical or environmental interest [33, 34]. Many virulence factors and/or antimicrobial resistance genes are shared and acquired via GIs [35]. Nine GIs were identified in *R. equi* WY which ranged from 4.3 kb to 29.5 kb, including three unique GIs in *R. equi* WY. We considered the unique GIs, and the GIs encoding genes involved in virulence, resistance, or niche adaptation were worth further analysis. In order to facilitate comparison of the structure of GIs, we only included complete genomes in our analysis.

**Figure 5:**
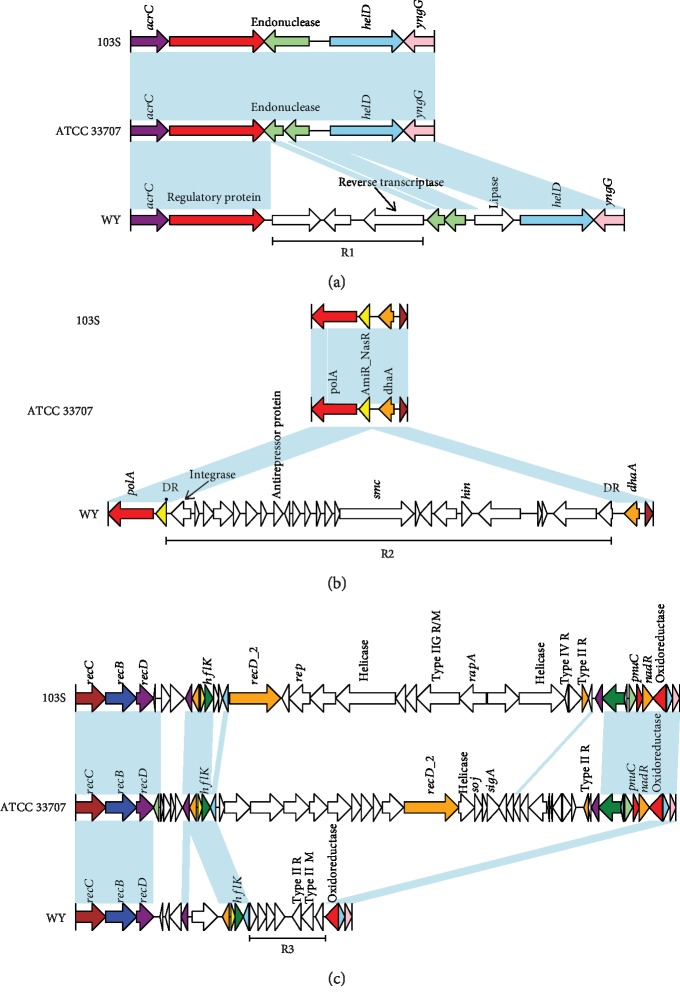
Comparison of the structure of three unique regions (R1-R3) of *R. equi* WY with the corresponding region of two other *R. equi* complete genomes. R1-R3 correspond to R1-R3 in Figure 4(a). The identical sequence regions (>60% identity) are connected by light blue bars. Homologous genes are marked with the same color, whereas nonhomologous genes are left blank.

Although most of the genes in GIs were of unknown function, several potential MGEs were found through comparative analysis. A reverse transcriptase was identified (Figure 5(a), R1), which is used to generate complementary DNA (cDNA) from an RNA template. This 4.6 kb fragment may be derived from retroviruses. A 26 kb unique GI (Figure 5(b), R2) including putative prophage phiRv2 integrase was located between AmiR_NasR (a RR of TCS) and *dhaA* (haloalkane dehalogenase). Moreover, genes encoding antirepressor protein, chromosome partition protein, and DNA-invertase *hin* were found downstream of the integrase gene. In addition, there were two identical 26 bp direct repeats (attL/attR) in both ends of R2 (Figure 5(b), R2). This finding suggests that R2 originated from phage. Interestingly, we also observed a potential GI that may be associated with the acquisition of a restriction modification (RM) system, which acts as an important immune system for bacteria that prevents the uptake of exogenous DNA [36] (Figure 5(c), R3). A gene cluster encoding type II RM system was present in *R. equi* WY, as well as different types of RM located in *R. equi* 103S and ATCC 33707 (type IIG R/M, type IV R, and type II R in 103S; type II R in ATCC 33707). HGT involved in the RM systems has been widely observed, which could be mediated by MGE, such as phage [37] and insertion sequence [38]. However, there seem to be no phages or MGEs involved in these acquired genes, consistent with a previous finding in *Arthrospira platensis* [39]. We cannot exclude the possibility that the genomic architecture in *R. equi* may have undergone various HGT events after the acquisition of RM systems.

**Figure 6:**
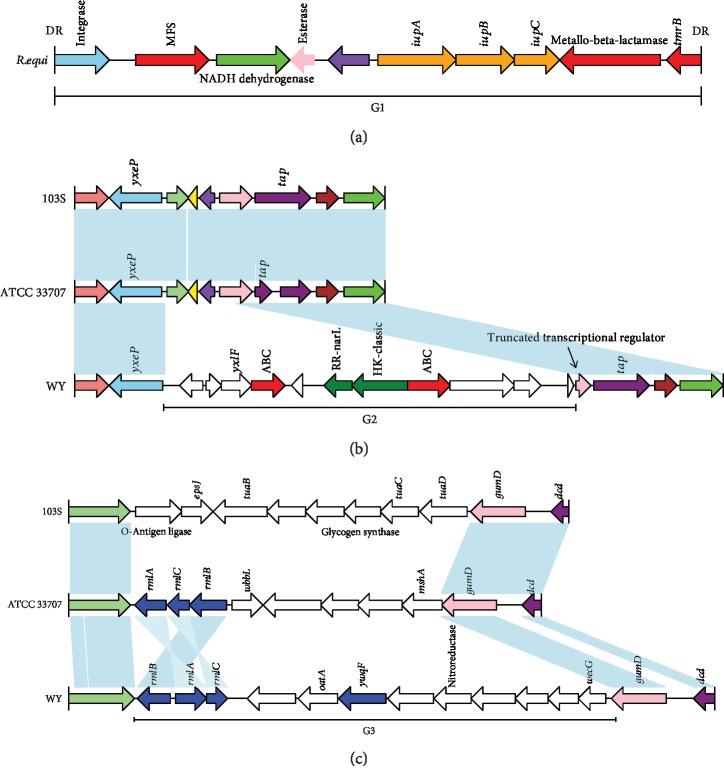
Comparison of the structure of the genomic islands (G1-G3) of *R. equi* WY with the corresponding region of two other *R. equi* complete genomes. The identical sequence regions (>60% identity) are connected by light blue bars. Homologous genes are marked with the same color, whereas nonhomologous genes are left blank. The different functional elements are labeled in different colors, with resistance genes in red, virulence genes in blue, and TCS genes in green.

GIs were also identified harboring genes encoding virulence or resistance genes. Nine genomes of *R. equi* share a 12.2 kb GI region containing a putative integrase followed by a major facilitator superfamily efflux pump (MFS), a metallo-beta-lactamase and a tunicamycin resistance protein (*tmrB*) (Figure 6(a), G1). It also contains an *iupABC* operon consisting of *iupA*, *iupB*, and *iupC*. The first gene *iupA* of the *iupABC* operon encodes an ABC transport system highly similar to siderophore uptake systems and confers *R. equi* the ability to use heme and hemoglobin as a source of iron [40]. Moreover, two 7 bp direct repeats (DRs) were detected in both upstream of integrase and downstream of *tmrB*, which suggests that the resistance and transport genes may be acquired by HGT under the help of MGE. Some GIs were only shared by partial strains in *R. equi*. For instance, a GI composed of a pair of TCS proteins and ABC transport systems was only detected in *R. equi* WY, C 7, NBRC 101255, N1295, and DSM 20295, but not in the other four strains (Figure 6(b), G2; comparison of incomplete genomes was not shown). The ABC transport gene downstream of TCS proteins shows 46.71% similarity with that of the daunorubicin resistance ABC transporter, which may be a potential drug resistance factor. Although no potential MGEs were found in the flanking region, a truncated gene encoding a transcriptional regulator existed at the edge of G2, with an intervening sequence identical to that of 103S and ATCC 33707 (a total of 777 bp in length). This suggests that the G2 region may have been acquired by HGT, resulting in the break of transcriptional regulator gene in *R. equi* WY. A similar event was also observed in G3, where a 13 kb GI shared by *R. equi* WY, DSM 20295, and N1288 was detected (Figure 6(c), G3; comparison of incomplete genomes was not shown). It harbored three potential virulence genes (*rmlA*, *rmlB*, and *rmlC*) involved in thymidine diphosphate-L-rhamnose biosynthesis and required for the assembly of surface glycoconjugates in a growing list of bacterial pathogens [41]. The putative UDP-glucose dehydrogenase encoded by *ywqF* also existed in G3. A previous study has demonstrated that UDP-glucose dehydrogenase can catalyze the conversion of UDP-glucose to UDP-glucuronic acid and is required for the virulence of *Xanthomonas campestris* [42].

### 2.7. Comparative Analysis of Prophages in *R. equi*

Phages and prophages allow host bacteria to acquire antibiotic resistance and virulence, to adapt to new environmental niche, or to become pathogenic [43]. The former analysis has revealed that the *R. equi* contains a higher proportion of phage-derived genes. Therefore, it is necessary to analyze the structure and function of prophage in the genome of *R. equi*. A total of 11 complete prophages and 10 incomplete phage regions were detected in the genomes of nine *R. equi* strains (Figure 4(b)). Since a number of *R. equi* phages have been reported, we also attempted to determine the type of *R. equi* prophages. However, no known type of prophage was identified, indicating the high diversity of *R. equi* phages.

Unexpectedly, antibiotic resistance and virulence genes were not found in the complete prophage region, except for a putative acetyltransferase-coding gene in the prophage region of *R. equi* N1301 (Supplementary [Supplementary-material supplementary-material-1]), which can transfer an acetyl group to a substrate and confer antibiotic resistance by catalyzing the acetylation of amino groups in aminoglycoside antibiotics [44]. Likewise, the potential virulence factors, thioredoxin and thioredoxin reductase, were found in the incomplete phage region of *R. equi* NBRC 101255 (Supplementary [Supplementary-material supplementary-material-1]), which can protect bacteria from oxidative damage and promote intracellular replication and virulence in *Salmonella enterica* serovar Typhimurium [45].

**Figure 7:**
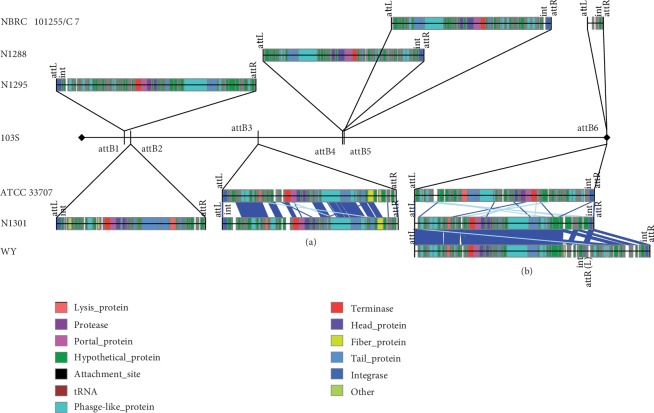
Comparative analysis of prophages in *R. equi* genomes. The spots for recombination were identified using *R. equi* 103S genome as the reference sequence. Regions of the phage genomes which shared high identity (>80%) and the same *attB* are connected by blue bars. All of the prophages displayed similar structures, and the integrase is present in the extremity of prophage and adjacent to the *attL*/*attR*. The different functional elements in phages are also shown (see figure legend). (a) Proteins of two phages, including lysis protein, fiber protein, and tail protein, shared high identities. (b) Sequence of the prophage of WY showed a high identity with that of N1301 but was different from that of ATCC 33707.

In order to identify the insertion sites of prophages in *R. equi*, a comparative analysis was performed using *R. equi* 103S genome as the reference sequence, because it has suffered little from phages (Figure 7). We detected nine kinds of complete prophage with attachment sites (*attP*, *attL*/*attR*); C 7 and NBRC 101255 shared the same complete prophage, while the insertion site of complete prophage in DSM 20295 cannot be identified. Through the analysis of the insertion sites, six different prophage insertion sites (*attB*) were found in *R. equi* 103S. Among these, two insertion sites (*attB*3, *attB*6) can be considered preferred spots of recombination, by two prophages (42.9 kb and 43.4 kb) from ATCC 33707 and N1301 inserted at *attB*3 (Figure 7(a)) and by three prophages from ATCC 33707, N1301, and WY inserted at *attB*6 (Figure 7(b)). The prophage of *R. equi* WY was 5 kb larger than the other two prophages but showed a high similarity with that of N1301. Interestingly, another integrase gene (*int*) was found and a direct repeat (*attR*/*attL*) downstream of *int* was also identified, which was identical to that of both ends *attL*/*attR* and *attB*6. This suggests that this prophage of *R. equi* WY was a mosaic phage formed by two lysogenic phages, and the recombination event may have occurred before or after the integration to *R. equi* WY. In addition, an integrated short fragment of *R. equi* C 7, NBRC 101255, and N1295 using *attB*6 as insertion site was also detected. This fragment encoded 7 CDSs including an integrase and a nucleoid-associated protein, globally surrounded by two 23 bp *attP* (*attL*/*attR*) (Figure 7(b)). In summary, the genomes of *R. equi* suffered from wide phage invasion in the evolutionary process, which accounted for the majority of genome expansion. They also appeared to be two preferred spots for recombination observed in this analysis.

In conclusion, our analysis revealed that virulence factors, resistance genes, and TCS genes were significantly enriched in the core genome of *R. equi* and suggested that the core genome contributes to the pathogenicity and niche adaptation of *R. equi*. Additionally, comparative genomic analysis demonstrated that the genomes of *R. equi* shared identical collinearity relationship and lacked significant chromosomal rearrangement, and the genes located in nonalignment regions were mainly acquired in the form of GIs and prophages. This study utilizes a direct comparative method to analyze *Rhodococcus* and *R. equi* and will facilitate in better understanding the genomic diversity, evolution, and structural variation of *R. equi*, as well as offer a valuable resource for functional genomic studies.

## 3. Methods

In this work, only explicit species from *Rhodococcus* were selected for analysis. All sequences were obtained from NCBI database (http://www.ncbi.nlm.nih.gov) (Supplementary [Supplementary-material supplementary-material-1]) in July 2016. The detailed genomic annotation of *R. equi* WY was shown in Supplementary [Supplementary-material supplementary-material-1]. *R. equi* WY was isolated from the laboratory of the central hospital of Lishui, Lishui, China in 2014. The genome sequence of WY was generated using SMRTbell Template Prep Kit (Pacific Biosciences, Menlo Park, CA), according to the PacBio standard protocol. A 20 kb library was constructed and sequenced on a PacBio RS II instrument with three SMRT cells. For draft assembly, a 300 bp Illumina PE library and a 3 kb Mate-pair library were also constructed, and 101 bp paired-end reads were generated using an Illumina HiSeq 2000 Sequencing System. Canu v1.5 [46] was used to process reads produced from the PacBio sequencing, which included correcting errors, trimming for quality, and then assembling the processed reads using an error rate of 0.025. This resulted in 25,912 reads with a mean length of 5,385 bp, an N50 read length of 6,325 bp, and a final assembly into a single contig (complete genome sequence). Adaptor trimming and quality filtering of short Illumina reads were performed using NGS QC Toolkit v2.3.3 [47]. And the clean reads were mapped onto the draft assembly to correct bases and fix misassemblies by Bwa v0.7.12 [48] and Pilon v1.16 [49], respectively. The complete nucleotide sequence of *R. equi* WY has been submitted to a public database and will be released soon.

Potential open reading frames (ORFs) were predicted and annotated using prodigal v2.6.2 [27] and Prokka v1.11 [50], respectively. ANI was calculated using OrthoANI v1.20 [51], and the cluster analysis was conducted with a threshold of 94% ANI value. Orthologous groups of genes from *Rhodococcus* were identified using InParanoid v4.1 [52] and QuickParanoid (http://pl.postech.ac.kr/QuickParanoid/). Pan-genome analysis of *Rhodococcus* and *R. equi* based on orthologous groups were performed using PanGP v1.0.1 [17]. Functional pathway annotation of genes was performed based on KEGG database by KAAS (http://www.genome.jp/tools/kaas). Hypergeometric test was used to assess whether the KEGG pathways or biological functions (e.g., virulence, resistance, and TCS genes) were overrepresented in specific gene list (e.g., core genes and stable core genes). Prokaryotic transposases and antibiotic resistance determinants were predicted by using HMMER v3.1b2 (http://www.hmmer.org/) searching against TnpPred (https://www.mobilomics.cl/tnppred/tnppred.php) [29] and Resfams HMM database v1.2 [53] with an *e*-value threshold of 1*e*-10, respectively. BLASTp (NCBI blast 2.2.31+, *e*-value < 1*e*-10) was used to identify phage-derived proteins, virulence factors, TCS proteins, and transport proteins against the public protein database (ACLAME v0.4 [28], VFDB 2016 [54]/MvirDB (http://mvirdb.llnl.gov/) [55], P2CS (http://www.p2cs.org/) [56], and TransportDB 2.0 [57]). The prophage regions in genomes were predicted using PHASTER (http://phaster.ca/) [43], and the genomic islands were identified by IslandViewer 3 (http://www.pathogenomics.sfu.ca/islandviewer).

Protein sequences were aligned with MAFFT v7.266 [58], followed by selecting the most reliable positions in the alignments using trimAl v1.4.rev15 [59] with “gappyout” mode. Maximum likelihood (ML) trees for each orthologous groups were constructed with RAxML v8.2.4 [60] using the JTT model of substitution and the gamma-based method for correcting the rate heterogeneity among sites. Trees were compared with Treedist program in PHYLIP v3.696 using the branch score distance of Kuhner and Felsenstein to generate an *n* × *n* distance matrix (*n* is the number of trees) [61]. Branch score distance is a distance measure that considers both topology and branch length and satisfies the requirements of a distance metric [62]. Subsequently, principal coordinate analysis (PCoA) was performed with the multidimensional scaling procedure with R script (Supplementary materials). By plotting the objects (the trees) along the most significant two first dimensions of PCoA, the major trends and groupings in the data can be visualized graphically [30]. Similarly, the ML tree of *Rhodococcus* species was build based on the concatenated stable core proteins, and *N. farcinica* was used as outgroup for rooting the tree. Whole-genome sequences including nine *R. equi* and one *R. defluvii* were aligned using mugsy v1r2.3 [63] with “fullsearch” mode, followed by SNP calling using in-house Python script (Supplementary materials). SNP concatenation tree rooted with *R. defluvii* was reconstructed by RAxML v8.2.4 [60]. The draft genomes of *R. equi* excluding plasmid fragments were reordered according to the reference genome (*R. equi* 103S [34]) by Mauve (version snapshot 2015-02-13 build 0) [64]. Comparisons of the nucleotide sequences were made using BLASTn (NCBI blast 2.2.31+, *e*-value < 1*e*-10). BRIG v0.95 [65] was used to show sequence alignments that were subsequently employed in comparative analysis.

## Data Availability

The genomic sequence of *R. equi* strain WY is available in NCBI database, and the accession numbers of the chromosome and plasmid are CP041647 and CP041646. The scripts mentioned in Methods are attached in the supplementary material.

## Supplementary Materials

Supplementary Materials Supplementary Figures: Fig. S1: cluster analysis of each Rhodococcus genome based on the average nucleotide identity. The values were calculated for 8742 pairwise comparisons of the 94 Rhodococcus genomes, compiled into a distance matrix, and used to perform cluster analysis. Fig. S2: accumulation curves for the pan/core genome of Rhodococcus (A) and R. equi (B). The unfilled circles denote the number of orthologous clusters for the different strain combinations. The connected median values represent the relationship between the species number and the orthologous cluster number. Blue curves denote the pan-genome, and red curves denote the core genome. Fig. S3: accumulation curves for the pan/core genome of R. erythropolis (A) and R. fascians (B). The unfilled circles denote the number of orthologous clusters for the different strain combinations. The connected median values represent the relationship between the species number and the orthologous cluster number. Blue curves denote the pan-genome, and red curves denote the core genome. Fig. S4: comparison of COG function classification of core genes according to each source of R. equi. Fig. S5: the ratio of mobile genetic elements (MGE) including transposase (A) and phage-derived genes (B) in Rhodococcus species. The colored dots denote the corresponding ordinate values. Boxplots indicate 0.25 and 0.75 quantiles, and the colored (red/blue) lines represent the median values of each species. Fig. S6: phylogenetic reconstruction of 28 representative genomes of Rhodococcus genus based on concatenated stable core genes rooted with N. farcinica. The asterisk denotes the bootstrap value < 100. The labels in the phylogenetic tree are listed in Supplementary Table S2. Supplementary Tables: Table S1: genomic information of the Rhodococcus species selected in this study. Table S2: the information of the explicit Rhodococcus species selected in this study. Table S3: genomic annotation information of R. equi WY. Table S4: enrichment analysis of virulence, resistance, and TCS genes in pan/core genome of R. equi. Table S5: pathway enrichment analysis of the virulent genes. Table S6: the COG function classification and functional annotation of the animal-unique genes. Table S7: functional annotation of the human-unique genes. Table S8: pathway enrichment analysis of the stable core genes. Table S9: prediction of prophages and functional annotation in R. equi N1301 and NBRC 101255. In-house scripts: PCoA_analysis.R—principal coordinate analysis (PCoA) was performed by using “cmdscale” function in “stats” R-package. Call_SNPs_from_mugsy.py—SNP calling procedure from sequence alignment by mugsy.
