# Supplementary material for: Comparative Genomic Analysis of Rhodococcus equi: An Insight into Genomic Diversity and Genome Evolution
Source: Int J Genomics. 2019 Dec 18;2019:8987436. doi: 10.1155/2019/8987436 (PMC6948317; doi:10.1155/2019/8987436)
Supplement: Supplementary Materials — Supplementary Figures: Fig. S1: cluster analysis of each Rhodococcus genome based on the average nucleotide identity. The values were calculated for 8742 pairwise comparisons of the 94 Rhodococcus genomes, compiled into a distance matrix, and used to perform cluster analysis. Fig. S2: accumulation curves for the pan/core genome of Rhodococcus (A) and R. equi (B). The unfilled circles denote the number of orthologous clusters for the different strain combinations. The connected median values represent the relationship between the species number and the orthologous cluster number. Blue curves denote the pan-genome, and red curves denote the core genome. Fig. S3: accumulation curves for the pan/core genome of R. erythropolis (A) and R. fascians (B). The unfilled circles denote the number of orthologous clusters for the different strain combinations. The connected median values represent the relationship between the species number and the orthologous cluster number. Blue curves denote the pan-genome, and red curves denote the core genome. Fig. S4: comparison of COG function classification of core genes according to each source of R. equi. Fig. S5: the ratio of mobile genetic elements (MGE) including transposase (A) and phage-derived genes (B) in Rhodococcus species. The colored dots denote the corresponding ordinate values. Boxplots indicate 0.25 and 0.75 quantiles, and the colored (red/blue) lines represent the median values of each species. Fig. S6: phylogenetic reconstruction of 28 representative genomes of Rhodococcus genus based on concatenated stable core genes rooted with N. farcinica. The asterisk denotes the bootstrap value < 100. The labels in the phylogenetic tree are listed in Supplementary Table S2. Supplementary Tables: Table S1: genomic information of the Rhodococcus species selected in this study. Table S2: the information of the explicit Rhodococcus species selected in this study. Table S3: genomic annotation information of R. equi WY. Table S4: enrichm [file 8987436.f1.pdf]

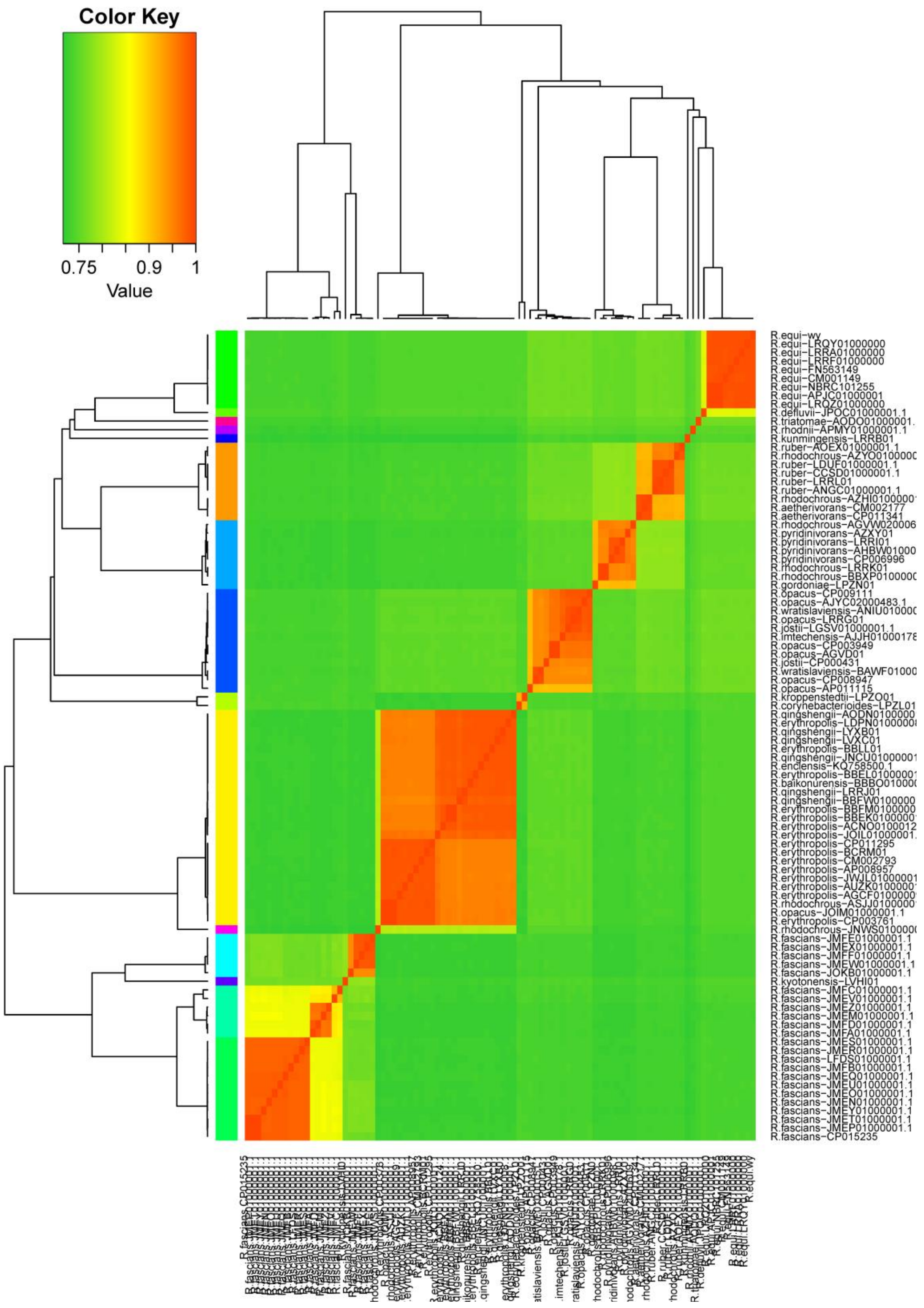

Fig. S1. Cluster analysis of each *Rhodococcus* genome based on the average nucleotide identity. The values were calculated for 8742 pairwise comparisons of the 94 *Rhodococcus* genomes, compiled into a distance matrix, and used to perform cluster analysis.

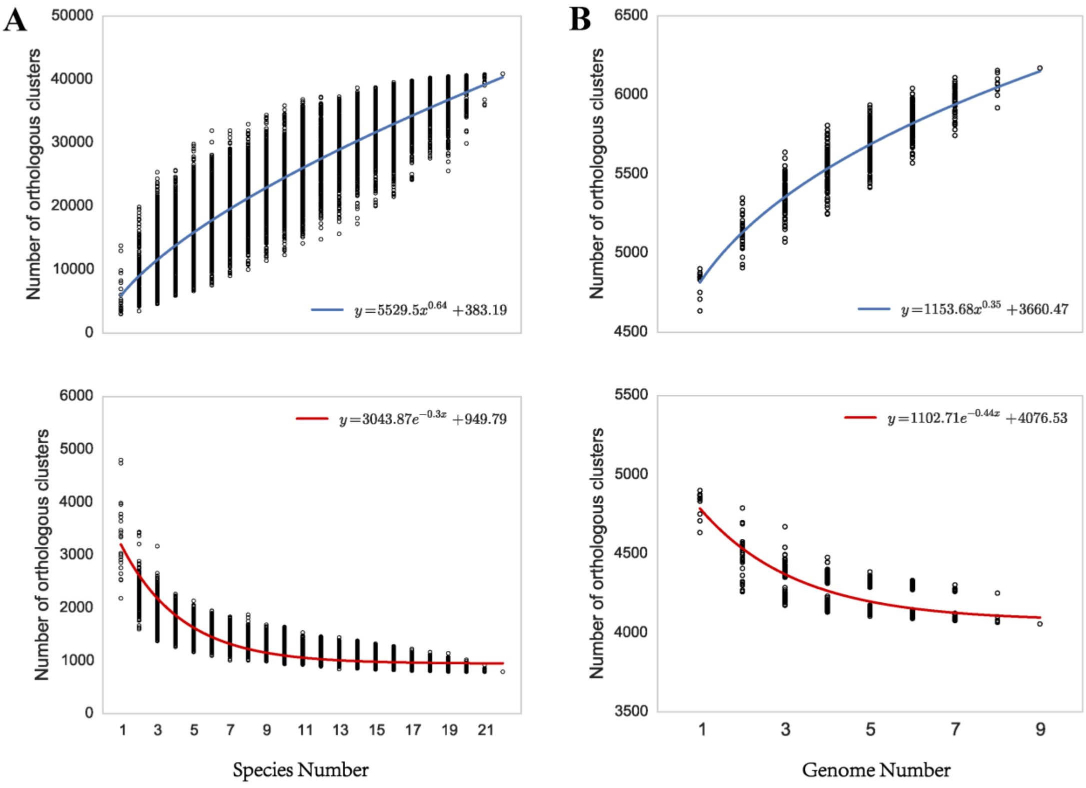

Fig. S2. Accumulation curves for the pan/core-genome of *Rhodococcus* (A) and *R. equi* (B). The unfilled circles denote the number of orthologous clusters for the different strain combinations. The connected median values represent the relationship between the species number and orthologous cluster number. Blue curves denote the pan-genome and red curves denote the core-genome.

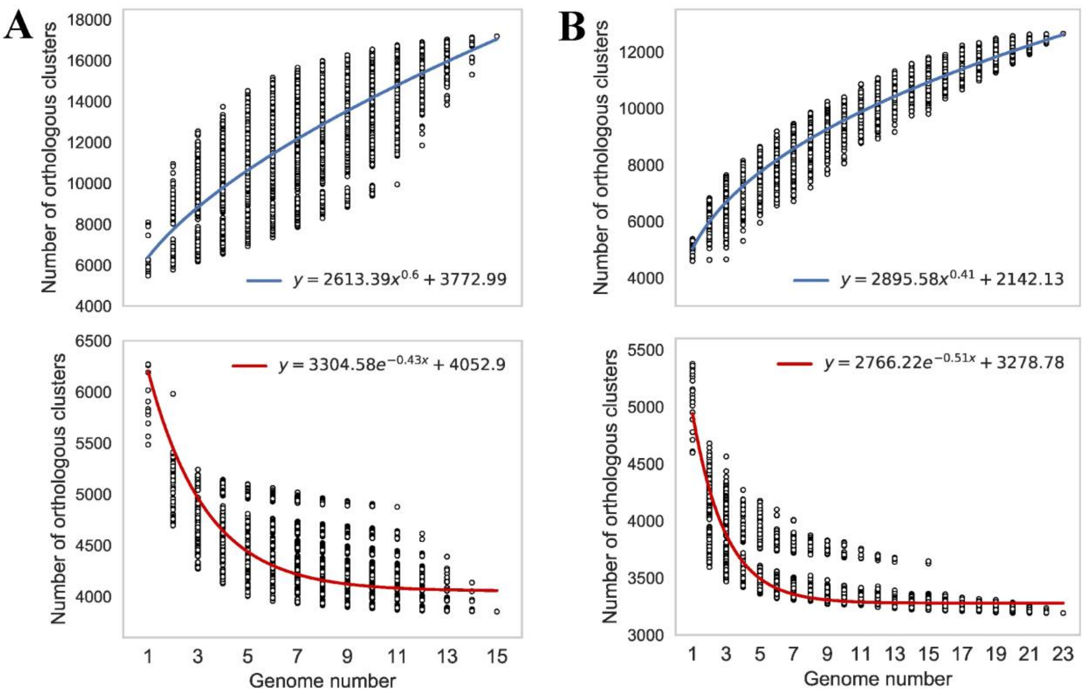

Fig. S3. Accumulation curves for the pan/core-genome of *R. erythropolis* (A) and *R. fascians* (B). The unfilled circles denote the number of orthologous clusters for the different strain combinations. The connected median values represent the relationship between the species number and orthologous cluster number. Blue curves denote the pan-genome and red curves denote the core-genome.

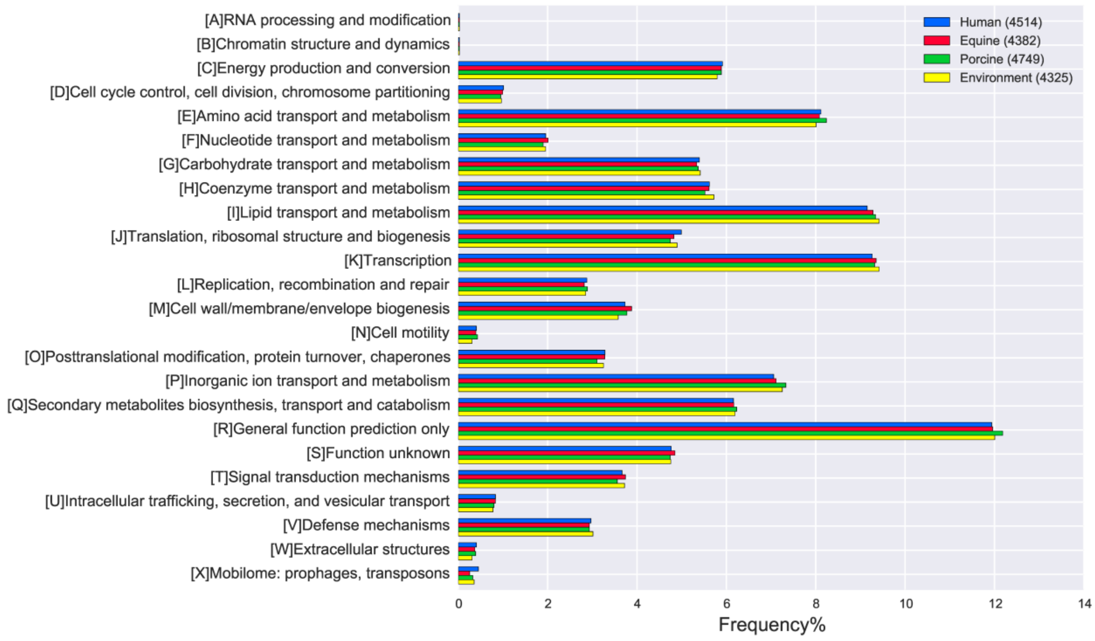

Fig. S4. Comparison of COG function classification of core genes according to each source of *R. equi*.

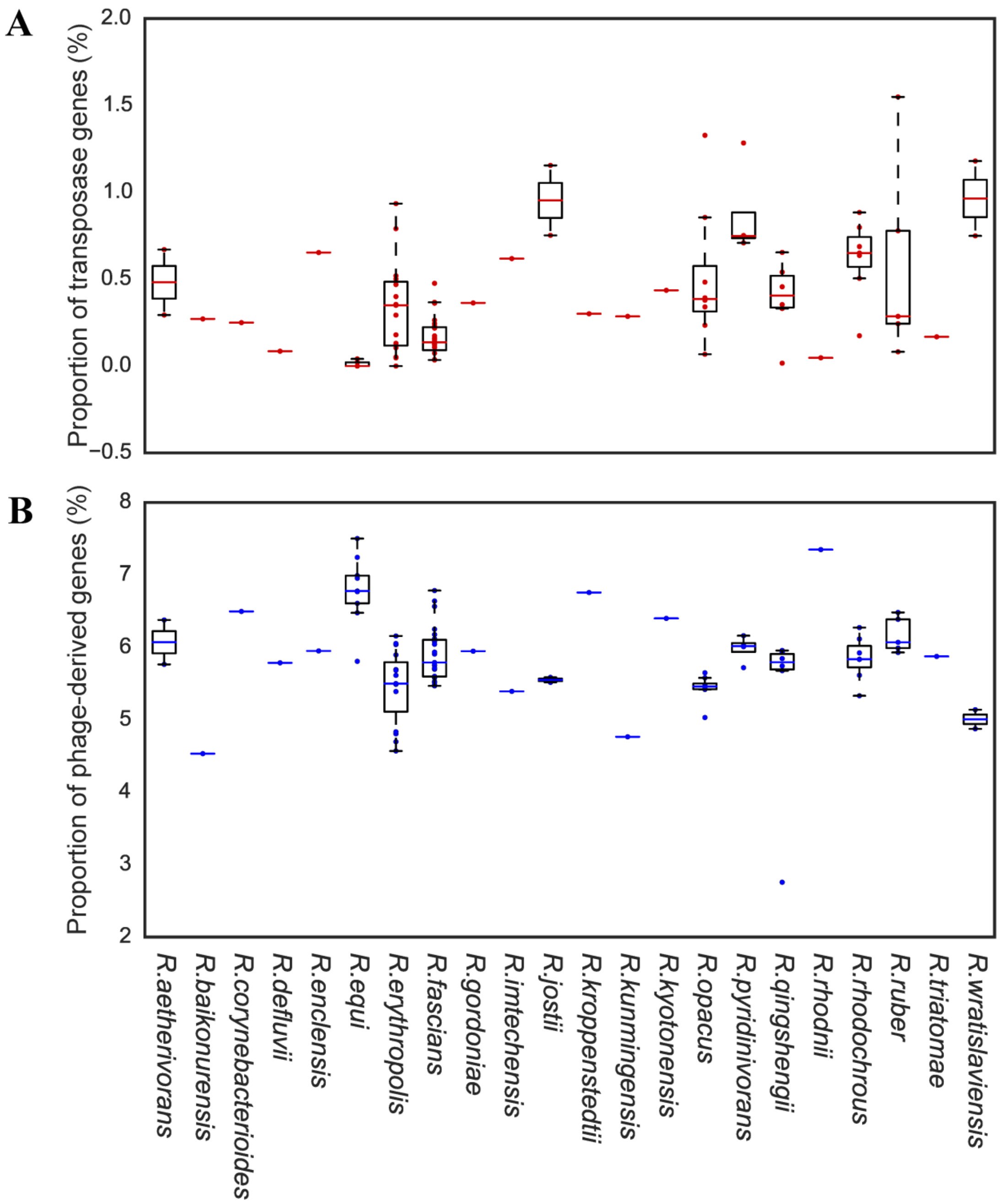

Fig. S5. The ratio of mobile genetic elements (MGE) including transposase (A) and phage-derived genes (B) in *Rhodococcus* species. The colored dots denote the corresponding ordinate values. Boxplots indicate 0.25 and 0.75 quantiles and the colored (red/blue) lines represent the median values of each species.

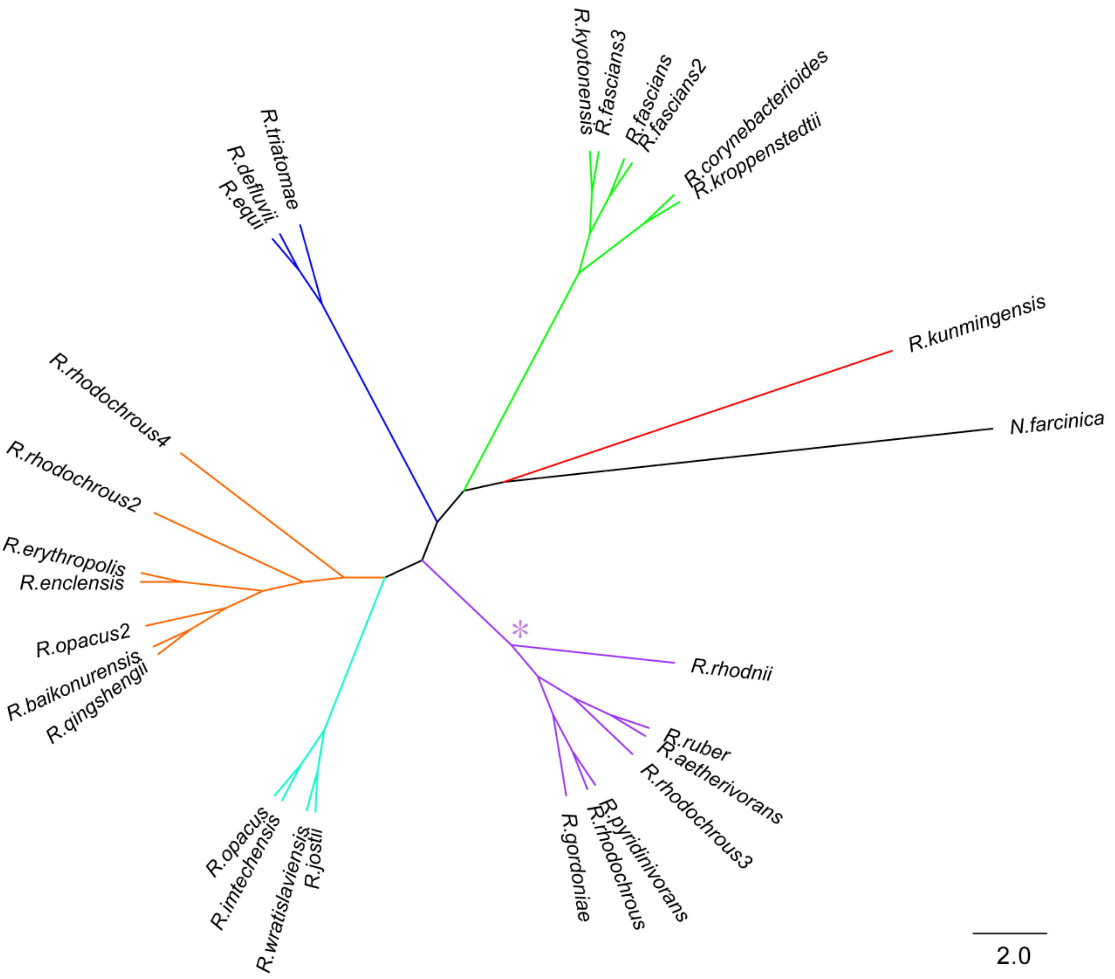

Fig. S6. Phylogenetic reconstruction of 28 representative genomes of *Rhodococcus* genus based on concatenated stable core genes rooted with *N. farcinica*. The asterisk denotes the bootstrap value < 100. The labels in the phylogenetic tree are listed in Supplementary Table S2.

Supplementary Table S1. Genomic information of the *Rhodococcus* species selected in this study

Species	Genome Size(Mb)	Gene Number	GC content	Core genes	Pan genes
R.aetherivorans(n=2)	5.96(5.92-5.99)	5481(5445-5517)	70.56%(70.51%-70.62%)	4924	6029
R.baikonurensis(n=1)	6.59	8845	62.36%	8845	8845
R.corynebacterioides(n=1)	3.89	3601	70.26%	3601	3601
R.defluvii(n=1)	5.13	4682	68.73%	4682	4682
R.enclensis(n=1)	7.36	7039	62.30%	7039	7039
R.equi(n=9)	5.15(4.97-5.26)	4828(4648-4916	68.78%(68.76%-68.82	4057	6172

		)	%)		
R.erythropolis(n=15)	6.66(6.28-7.11)	6807(5786-8555)	62.37%(62.19%-62.47%)	3853	17184
R.fascians(n=23)	5.62(5.09-6.1)	5319(4790-5736)	64.53%(64.15%-64.83%)	3192	12646
R.gordoniae(n=1)	4.81	4404	67.96%	4404	4404
R.imtechensis(n=1)	7.34	6952	67.35%	6952	6952
R.jostii(n=2)	7.99(7.8-8.18)	7484(7177-7792)	67.41%(67.3%-67.52%)	5558	9290
R.kroppenstedtii(n=1)	3.9	3654	70.14%	3654	3654
R.kunmingensis(n=1)	5.6	5245	66.20%	5245	5245
R.kyotonensis(n=1)	5.47	5045	65.19%	5045	5045
R.opacus(n=8)	8.05(6.36-8.87)	7418(5885-8278)	66.75%(62.43%-67.89%)	3530	14048
R.pyridinivorans(n=4)	5.36(5.17-5.81)	4962(4791-5296)	67.88%(67.7%-67.98%)	3694	6891
R.qingshengii(n=6)	6.84(6.4-7.13)	7372(6025-11170)	62.38%(62.01%-62.53%)	3481	16061
R.rhodnii(n=1)	4.38	4205	69.73	4205	4205
R.rhodochrous(n=7)	6.07(5.16-6.77)	5633(4759-6339)	66.82%(61.67%-70.23%)	2661	11615
R.ruber(n=5)	5.67(5.27-6.11)	5248(4873-5649)	70.35%(69.72%-70.67%)	4093	7350
R.triatomae(n=1)	5.82	5343	69.02%	5343	5343
R.wratislaviensis(n=2)	9.77(9.16-10.38)	9228(8982-9474)	66.92%(66.78%-67.06%)	5953	12331

Supplementary Table S2. The information of the explicit *Rhodococcus* species selected in this study.										
**Organism/Name**	**BioProject Accession**	**BioProject ID**	**BioSample Accession**	**Assembly Accession**	**WGS**	**Chromosomes/RefSeq**	**Chromosomes/INSDC**	**Reference defined by NCBI**	**Representative defined in cluster analysis**	**Label in phylogenetic analysis**
Rhodococcus aetherivorans	PRJNA281168	281168	SAMN03486836	GCA_000982715.1	-	NZ_CP011341.1	CP011341.1	REFR	REP	*R. aetherivorans*
Rhodococcus aetherivorans	PRJNA213668	213668	SAMN02470652	GCA_000470885.1	AVAE01	NZ_CM002177.1	CM002177.1	-		
Rhodococcus baikonurensis JCM 18801	PRJDB796	252060	SAMD00016817	GCA_001311605.1	BBBO01	-	-	-	REP	*R. baikonurensis*
Rhodococcus corynebacterioides	PRJNA306614	306614	SAMN04357305	GCA_001646675.1	LPZL01	-	-	-	REP	*R. corynebacterioides*
Rhodococcus defluvii	PRJNA255084	255084	SAMN02910065	GCA_000738775.1	JPOC01	-	-	REFR	REP	*R. defluvii*
Rhodococcus enclensis	PRJNA302094	302094	SAMN04263893	GCA_001456965.1	LNQH01	-	-	-	REP	*R. enclensis*
Rhodococcus equi N1288	PRJNA306614	306614	SAMN04357306	GCA_001646885.1	LRQY01	-	-	-		
Rhodococcus equi N1301	PRJNA306614	306614	SAMN04357308	GCA_001646925.1	LRRA01	-	-	-		
Rhodococcus equi N1295	PRJNA306614	306614	SAMN04357307	GCA_001646905.1	LRQZ01	-	-	-		
Rhodococcus equi DSM 20295	PRJNA306614	306614	SAMN04357304	GCA_001646645.1	LRRF0	-	-	-		

					1					
Rhodococcus equi 103S	PRJEA41335	41335	SAMEA2272695	GCA_000196695.1	-	NC_014659.1	FN563149.1	REFR	REP	*R. equi*
Rhodococcus equi ATCC 33707	PRJNA31525	31525	SAMN00001487	GCA_000164155.2	ADNW02	NZ_CM001149.1	CM001149.1	-		
Rhodococcus equi C 7	PRJNA190104	190104	SAMN02471952	GCA_000473915.1	APJC01	-	-	-		
Rhodococcus equi NBRC 101255	PRJDB292	172260	SAMD00046489	GCA_001552575.1	BCRL01	-	-	-		
Rhodococcus equi WY										
Rhodococcus erythropolis	PRJNA280916	280916	SAMN03480662	GCA_000975175.1	-	NZ_CP011295.1	CP011295.1	-		
Rhodococcus erythropolis	PRJNA284797	284797	SAMN03733238	GCA_001020225.1	LDPN01	-	-	-		
Rhodococcus erythropolis	PRJNA238534	238534	SAMN02645506	GCA_000719985.1	JOIL01	-	-	-		
Rhodococcus erythropolis	PRJDB3086	259846	SAMD00019512	GCA_000747745.1	BBLL01	-	-	-		
Rhodococcus erythropolis	PRJNA268657	268657	SAMN03222825	GCA_000830355.1	JWJL01	-	-	-		
Rhodococcus erythropolis CCM2595	PRJNA81583	81583	SAMN02603403	GCA_000454045.1	-	NC_022115.1	CP003761.1	-		
Rhodococcus erythropolis DN1	PRJNA214035	214035	SAMN02470607	GCA_000454425.1	AUZK01	-	-	-		

Rhodococcus erythropolis JCM 9803	PRJDB891	243769	SAMD00016839	GCA_001312725.1	BBEK01	-	-	-		
Rhodococcus erythropolis JCM 9804	PRJDB926	243788	SAMD00016840	GCA_001313245.1	BBFM01	-	-	-		
Rhodococcus erythropolis JCM 9805	PRJDB892	243770	SAMD00016841	GCA_001312745.1	BBEL01	-	-	-		
Rhodococcus erythropolis NBRC 15567	PRJDB293	172261	SAMD00046519	GCA_001552595.1	BCRM01	-	-	-		
Rhodococcus erythropolis PR4	PRJDA20395	20395	-	GCA_000010105.1	-	NC_012490.1	AP008957.1	REFR	REP	*R. erythropolis*
Rhodococcus erythropolis R138	PRJNA188397	188397	SAMN02639992	GCA_000696675.2	-	NZ_CP007255.1	CP007255.1	-		
Rhodococcus erythropolis SK121	PRJNA34067	34067	SAMN00002168	GCA_000174835.1	ACNO01	-	-	-		
Rhodococcus erythropolis XP	PRJNA72225	72225	SAMN02470952	GCA_000225665.2	AGCF01	-	-	-		
Rhodococcus fascians	PRJNA286803	286803	SAMN03770810	GCA_001037935.1	LFDS01	-	-	-		
Rhodococcus fascians	PRJNA252927	252927	SAMN02864791	GCA_000759005.1	JOKB01	-	-	-		
Rhodococcus fascians 02-815	PRJNA233522	233522	SAMN02569985	GCA_000760835.1	JMFF01	-	-	-		
Rhodococcus fascians 02-816c	PRJNA233522	233522	SAMN02569986	GCA_0007608	JMFE	-	-	-		

				55.1	01					
Rhodococcus fascians 04-516	PRJNA233522	233522	SAMN02569987	GCA_000760685.1	JMFD01	-	-	-		
Rhodococcus fascians 05-339-1	PRJNA233522	233522	SAMN02569988	GCA_000760895.1	JMFC01	-	-	-		
Rhodococcus fascians 05-561-1	PRJNA233522	233522	SAMN02569989	GCA_000760875.1	JMFB01	-	-	-		
Rhodococcus fascians A21d2	PRJNA233522	233522	SAMN02569990	GCA_000760905.1	JMFA01	-	-	-		
Rhodococcus fascians A25f	PRJNA233522	233522	SAMN02569991	GCA_000760935.1	JMEZ01	-	-	-		
Rhodococcus fascians A3b	PRJNA233522	233522	SAMN02569992	GCA_000760675.1	JMEY01	-	-	-		
Rhodococcus fascians A44A	PRJNA233522	233522	SAMN02569993	GCA_000760735.1	JMEX01	-	-	REFR	REP	*R. fascians* 3
Rhodococcus fascians A73a	PRJNA233522	233522	SAMN02569994	GCA_000760755.1	JMEW01	-	-	-		
Rhodococcus fascians A76	PRJNA233522	233522	SAMN02569995	GCA_000760955.1	JMEV01	-	-	-		
Rhodococcus fascians A78	PRJNA233522	233522	SAMN02569996	GCA_000760775.1	JMEU01	-	-	-		
Rhodococcus fascians	PRJNA313	313428	SAMN045	GCA_001	-	NZ_CP0152	CP015235.1	REFR	REP	*R. fascians*

D188-1	428		21220	6203 05.1		35.1				
Rhodococcus fascians D188-2	PRJN A233 522	233 522	SAM N025 69997	GCA _000 7609 75.1	J M E T0 1	-	-	-		
Rhodococcus fascians GIC26	PRJN A233 522	233 522	SAM N025 69998	GCA _000 7607 95.1	J M ES 01	-	-	-		
Rhodococcus fascians GIC36	PRJN A233 522	233 522	SAM N025 69999	GCA _000 7608 15.1	J M E R0 1	-	-	-		
Rhodococcus fascians LMG 3602	PRJN A233 522	233 522	SAM N025 70000	GCA _000 7609 95.1	J M E Q 01	-	-	-		
Rhodococcus fascians LMG 3605	PRJN A233 522	233 522	SAM N025 70001	GCA _000 7610 15.1	J M EP 01	-	-	-		
Rhodococcus fascians LMG 3616	PRJN A233 522	233 522	SAM N025 70002	GCA _000 7610 35.1	J M E O 01	-	-	-		
Rhodococcus fascians LMG 3625	PRJN A233 522	233 522	SAM N025 70004	GCA _000 7610 75.1	J M E M 01	-	-	-	REP	*R. fascians* 2
Rhodococcus fascians NBRC 12155 = LMG 3623	PRJN A233 522	233 522	SAM N025 70003	GCA _000 7610 55.1	J M E N 01	-	-	-		
Rhodococcus gordoniae	PRJN A306 614	306 614	SAM N043 57310	GCA _001 6466 55.1	LP Z N 01	-	-	-	REP	*R. gordonia e*
Rhodococcus imtechensis RKJ300 = JCM 13270	PRJN A112 531	112 531	SAM N024 70011	GCA _000 2608 15.1	AJ JH 01	-	-	-	REP	*R. imtechen sis*
Rhodococcus jostii	PRJN A289 318	289 318	SAM N038 52607	GCA _001 2928 45.1	L G S V 01	-	-	-		

Rhodococcus jostii RHA1	PRJNA13693	13693	SAMN02604146	GCA_000014565.1	-	NC_008268.1	CP000431.1	REFR	REP	*R. jostii*
Rhodococcus kroppenstedtii	PRJNA306614	306614	SAMN04357311	GCA_001646725.1	LPZO01	-	-	-	REP	*R. kroppenstedtii*
Rhodococcus kunmingensis	PRJNA306614	306614	SAMN04357316	GCA_001646865.1	LRRB01	-	-	-	REP	*R. kunmingensis*
Rhodococcus kyotonensis	PRJNA314554	314554	SAMN04545449	GCA_001645385.1	LVHI01	-	-	-	REP	*R. kyotonensis*
Rhodococcus opacus	PRJNA246296	246296	SAMN02769606	GCA_000736435.1	-	-	CP008947.1	-		
Rhodococcus opacus	PRJNA238534	238534	SAMN02645507	GCA_000719995.1	JOIM01	-	-	-	REP	*R. opacus*2
Rhodococcus opacus	PRJNA306614	306614	SAMN04357312	GCA_001646735.1	LRRG01	-	-	-		
Rhodococcus opacus B4	PRJDA34839	34839	-	GCA_000010805.1	-	NC_012522.1	AP011115.1	-		
Rhodococcus opacus M213	PRJNA158507	158507	SAMN02469789	GCA_000264745.2	AJYC02	-	-	-		
Rhodococcus opacus PD630	PRJNA178618	178618	SAMN03081444	GCA_000599545.1	-	NZ_CP003949.1	CP003949.1	-		
Rhodococcus opacus PD630	PRJNA30413	30413	SAMN02596738	GCA_000234335.1	AGVD01	-	-	-		
Rhodococcus opacus strain 1CP							CP009111.1		REP	*R. opacus*
Rhodococcus pyridinivorans	PRJNA306614	306614	SAMN04357314	GCA_0016468	LRRI	-	-	-		

				05.1	01					
Rhodococcus pyridinivorans AK37	PRJNA76789	76789	SAMN02469321	GCA_000236965.2	AHBW01	-	-	-		
Rhodococcus pyridinivorans KG-16	PRJNA232743	232743	SAMN02486559	GCA_001465325.1	AZXY01	-	-	-		
Rhodococcus pyridinivorans SB3094	PRJNA231235	231235	SAMN02641478	GCA_000511305.1	-	NC_023150.1	CP006996.1	REFR	REP	*R. pyridinivorans*
Rhodococcus qingshengii	PRJNA316739	316739	SAMN04589880	GCA_001623435.1	LVXC01	-	-	-	REP	*R. qingshengii*
Rhodococcus qingshengii	PRJNA323519	323519	SAMN05180531	GCA_001662505.1	LYXB01	-	-	-		
Rhodococcus qingshengii	PRJNA306614	306614	SAMN04357315	GCA_001646745.1	LRRJ01	-	-	-		
Rhodococcus qingshengii	PRJNA246036	246036	SAMN02742160	GCA_000698455.1	JNCU01	-	-	-		
Rhodococcus qingshengii BKS 20-40	PRJNA185383	185383	SAMN02469985	GCA_000341815.1	AODN01	-	-	-		
Rhodococcus qingshengii JCM 15477	PRJDB938	243792	SAMD00016939	GCA_001313445.1	BBFW01	-	-	-		
Rhodococcus rhodnii LMG 5362	PRJNA183987	183987	SAMN02471027	GCA_000389715.1	APMY01	-	-	REFR	REP	*R. rhodnii*
Rhodococcus rhodochrous	PRJNA238534	238534	SAMN02645197	GCA_000716895.1	JNWS01	-	-	-	REP	*R. rhodochrous*4

Rhodococcus rhodochrous	PRJNA306614	306614	SAMN04357317	GCA_001646825.1	LRRK01	-	-	-		
Rhodococcus rhodochrous ATCC 17895	PRJNA201088	201088	SAMN02469312	GCA_000469645.1	ASJJ01	-	-	-	REP	*R. rhodochrous*2
Rhodococcus rhodochrous ATCC 21198	PRJNA229768	229768	SAMN02597428	GCA_000517665.1	AZHI01	-	-	-	REP	*R. rhodochrous*3
Rhodococcus rhodochrous BKS6-46	PRJNA76791	76791	SAMN02470012	GCA_000239135.3	AGVW02	-	-	-		
Rhodococcus rhodochrous KG-21	PRJNA232791	232791	SAMN02527332	GCA_001278665.1	AZYO01	-	-	-		
Rhodococcus rhodochrous NBRC 16069	PRJDB303	172271	SAMD00034188	GCA_001047055.1	BBXP01	-	-	-	REP	*R. rhodochrous*
Rhodococcus ruber	PRJNA285434	285434	SAMN03753826	GCA_001051275.1	LDUF01	-	-	-		
Rhodococcus ruber	PRJEB6917	268807	SAMEA2708319	GCA_000824945.1	CCSD01	-	-	-		
Rhodococcus ruber	PRJNA306614	306614	SAMN04357318	GCA_001646835.1	LRRL01	-	-	-		
Rhodococcus ruber BKS 20-38	PRJNA185259	185259	SAMN02470007	GCA_000341965.1	AOEX01	-	-	-		
Rhodococcus ruber Chol-4	PRJNA176883	176883	SAMN01804522	GCA_000347955.2	ANGC02	-	-	-	REP	*R. ruber*

Rhodococcus triatomae BKS 15-14	PRJNA185384	185384	SAMN02469993	GCA_000341795.1	AODO01	-	-	REFR	REP	*R. triatomae*
Rhodococcus wratislaviensis IFP 2016	PRJNA167644	167644	SAMN00998637	GCA_000325625.1	ANIU01	-	-	-		
Rhodococcus wratislaviensis NBRC 100605	PRJDB307	172275	SAMD00041784	GCA_000583735.1	BAWF01	-	-	-	REP	*R. wratislaviensis*

**Supplementary Table S3. Genomic annotation information of *R. equi* WY**						
Locus tag	Gene	Start	End	Strand	Length	Protein
wy_00001	-	333	473	+	141	hypothetical protein
wy_00002	*corA_1*	882	1934	+	1053	Magnesium transport protein CorA
wy_00003	*arpA_1*	2140	2796	+	657	A-factor receptor protein
wy_00008	-	3512	4270	-	759	IclR helix-turn-helix domain protein
wy_00009	*kstR_1*	4507	5109	+	603	HTH-type transcriptional repressor KstR
wy_00010	*hsaA_1*	5288	6496	+	1209	Flavin-dependent monooxygenase, oxygenase subunit HsaA
wy_00011	-	6530	8152	+	1623	3-oxo-5-alpha-steroid 4-dehydrogenase
wy_00012	*fadR_1*	8221	8730	+	510	Fatty acid metabolism regulator protein
wy_00013	*fabG_1*	8753	9529	-	777	3-oxoacyl-[acyl-carrier-protein] reductase FabG
wy_00014	*rluA_1*	9641	10534	-	894	Ribosomal large subunit pseudouridine synthase A
wy_00015	*fbpC_1*	11039	12028	+	990	Diacylglycerol acyltransferase/mycolyltransferase Ag85C precursor
wy_00016	*acr1_1*	12084	12965	+	882	Fatty acyl-CoA reductase
wy_00017	*catE*	12971	13510	+	540	Catechol-2,3-dioxygenase
wy_00018	-	13512	14948	-	1437	putative multidrug-efflux transporter/MT1670
wy_00019	*clpC_1*	15202	17634	+	2433	Negative regulator of genetic competence ClpC/MecB

wy_0002 0	*kstR2_1*	17699	18280	-	582	HTH-type transcriptional repressor KstR2
wy_0002 1	*fabG_2*	18466	19221	+	756	3-oxoacyl-[acyl-carrier-protein] reductase FabG
wy_0002 2	-	19323	19595	+	273	hypothetical protein
wy_0002 3	*kshA_1*	19685	20845	+	1161	3-ketosteroid-9-alpha-monooxygenase oxygenase subunit
wy_0002 4	*kdgR_1*	20921	21691	-	771	Pectin degradation repressor protein KdgR
wy_0002 5	*yedK_1*	21862	22587	-	726	Putative SOS response-associated peptidase YedK
wy_0002 6	-	22809	23426	+	618	hypothetical protein
wy_0002 7	*bmr3_1*	23621	25066	+	1446	Multidrug resistance protein 3
wy_0002 8	*gltA_1*	25236	26357	-	1122	Citrate synthase
wy_0002 9	*prpB_1*	26354	27292	-	939	Methylisocitrate lyase
wy_0003 0	*prpD*	27294	28796	-	1503	2-methylcitrate dehydratase
wy_0003 1	-	28967	30385	+	1419	Helix-turn-helix domain protein
wy_0003 2	-	30487	31152	+	666	hypothetical protein
wy_0003 3	*efpA_1*	31273	32724	+	1452	putative MFS-type transporter EfpA
wy_0003 4	*garR_1*	32753	33628	+	876	2-hydroxy-3-oxopropionate reductase
wy_0003 5	*metE_1*	33672	36044	-	2373	5-methyltetrahydropteroyltriglutamate--homocysteine methyltransferase
wy_0003 6	*gsiB_1*	36436	38049	-	1614	Glutathione-binding protein GsiB precursor
wy_0003 7	-	38046	39218	-	1173	Putative aminoglycoside phosphotransferase
wy_0003 8	-	39248	40483	-	1236	Steroid C26-monooxygenase
wy_0003 9	-	40648	41286	+	639	putative DNA-binding transcriptional regulator
wy_0004 0	*nox*	41314	41997	-	684	NADH dehydrogenase
wy_0004 1	-	42119	42604	-	486	hypothetical protein
wy_0004 2	*aseR*	42608	42961	-	354	HTH-type transcriptional repressor AseR
wy_0004 3	*aroG_1*	43254	44330	+	1077	Phospho-2-dehydro-3-deoxyheptonate aldolase, Phe-sensitive
wy_0004 4	-	44450	44875	+	426	Glyoxalase-like domain protein
wy_0004	*nlhH_1*	44880	45986	-	1107	Carboxylesterase NlhH

5						
wy_00046	-	46188	46799	+	612	transcriptional regulator BetI
wy_00047	*rcp1_1*	46938	48149	+	1212	Response regulator rcp1
wy_00048	*cph1*	48146	49768	+	1623	Phytochrome-like protein cph1
wy_00049	*rcp1_2*	49765	50208	+	444	Response regulator rcp1
wy_00050	-	50242	50670	-	429	hypothetical protein
wy_00051	-	50842	52521	+	1680	cardiolipin synthase 2
wy_00052	*sigF_1*	52528	53316	-	789	RNA polymerase sigma factor SigF
wy_00053	*rsbW_1*	53320	53694	-	375	Anti-sigma-F factor RsbW
wy_00054	*rsfB*	54257	54622	-	366	Anti-sigma-F factor antagonist RsfB
wy_00055	-	54660	54809	-	150	hypothetical protein
wy_00056	-	54997	55455	+	459	hypothetical protein
wy_00057	*whiB7_1*	55544	55867	+	324	putative transcriptional regulator WhiB7
wy_00058	*virS_1*	55827	56843	-	1017	HTH-type transcriptional regulator VirS
wy_00059	*dapC*	56840	58024	-	1185	putative N-succinyldiaminopimelate aminotransferase DapC
wy_00060	-	58153	58755	+	603	hypothetical protein
wy_00061	*sigJ*	58682	59653	-	972	ECF RNA polymerase sigma factor SigJ
wy_00062	-	60019	60834	+	816	hypothetical protein
wy_00063	*crtQ_1*	60831	62099	+	1269	4,4'-diaponeurosporenoate glycosyltransferase
wy_00064	*carA2_1*	62096	63715	+	1620	zeta-carotene-forming phytoene desaturase
wy_00065	*crtB_1*	63708	64694	+	987	All-trans-phytoene synthase
wy_00066	*idi*	64731	65324	+	594	Isopentenyl-diphosphate Delta-isomerase
wy_00067	-	65486	66742	+	1257	mce related protein
wy_00068	*otsA_1*	66743	68284	-	1542	Trehalose-phosphate synthase
wy_00069	-	68708	68956	+	249	hypothetical protein
wy_00070	-	69015	69683	-	669	hypothetical protein
wy_0007	-	69696	71354	-	1659	Type III restriction enzyme, res subunit

1						
wy_00072	-	71486	71638	-	153	hypothetical protein
wy_00073	-	71640	71852	-	213	hypothetical protein
wy_00074	-	71864	71989	-	126	hypothetical protein
wy_00075	-	72092	74392	-	2301	hypothetical protein
wy_00076	-	74522	74641	+	120	hypothetical protein
wy_00077	*rpfA*	74711	75343	-	633	Resuscitation-promoting factor RpfA precursor
wy_00078	*yccF*	75851	76255	-	405	Inner membrane protein YccF
wy_00079	-	76267	76506	-	240	hypothetical protein
wy_00080	-	76658	77251	+	594	hypothetical protein
wy_00081	-	77366	77965	-	600	hypothetical protein
wy_00082	-	78053	79231	-	1179	hypothetical protein
wy_00083	*sipW_1*	79228	79821	-	594	Signal peptidase I W
wy_00084	-	79829	80425	-	597	hypothetical protein
wy_00085	*sipW_2*	80439	81077	-	639	Signal peptidase I W
wy_00086	-	81079	81588	-	510	hypothetical protein
wy_00087	-	81629	82150	-	522	hypothetical protein
wy_00088	*xsc*	82467	84122	-	1656	Sulfoacetaldehyde acetyltransferase
wy_00089	*kynB_1*	84119	84895	-	777	Kynurenine formamidase
wy_00090	*metA*	84999	85913	-	915	Homoserine O-succinyltransferase
wy_00091	*cspD*	86149	86562	+	414	Cold shock-like protein CspD
wy_00092	-	86639	87136	-	498	hypothetical protein
wy_00093	-	87164	88909	-	1746	Major Facilitator Superfamily protein
wy_00094	-	88997	89806	-	810	Glutamine cyclotransferase
wy_00095	-	89816	90715	+	900	hypothetical protein
wy_00096	-	90712	93465	+	2754	hypothetical protein
wy_0009	-	93462	94019	+	558	phosphatidylglycerophosphatase B

7						
wy_00098	-	94066	94284	-	219	hypothetical protein
wy_00099	*pbuG*	94396	95886	+	1491	Guanine/hypoxanthine permease PbuG
wy_00100	-	95883	96353	+	471	DNA-binding transcriptional repressor MarR
wy_00101	-	96448	97665	+	1218	enterobactin exporter EntS
wy_00102	-	97617	98168	-	552	hypothetical protein
wy_00104	*rlmB*	98879	99685	+	807	23S rRNA (guanosine-2'-O-)-methyltransferase RlmB
wy_00105	-	99682	99954	+	273	hypothetical protein
wy_00106	-	99961	100731	-	771	hypothetical protein
wy_00107	-	100737	101606	-	870	hypothetical protein
wy_00108	*serC*	101912	103030	-	1119	Phosphoserine aminotransferase
wy_00109	*citA*	103087	104214	-	1128	Citrate synthase 1
wy_00110	*pdxH_1*	104442	105092	+	651	Pyridoxine/pyridoxamine 5'-phosphate oxidase
wy_00111	*entS_1*	105089	106369	+	1281	Enterobactin exporter EntS
wy_00112	*kdgK*	106362	107282	+	921	2-dehydro-3-deoxygluconokinase
wy_00113	*gltA2*	107340	108638	+	1299	Citrate synthase 1
wy_00114	*fkbP*	108676	109401	+	726	FK506-binding protein
wy_00115	*fbpA_1*	109562	110647	-	1086	Diacylglycerol acyltransferase/mycolyltransferase Ag85A precursor
wy_00116	-	110800	113190	-	2391	hypothetical protein
wy_00117	*prrB_1*	113180	114484	-	1305	Sensor-type histidine kinase PrrB
wy_00118	*prrA_1*	114481	115215	-	735	Transcriptional regulatory protein PrrA
wy_00119	*badR*	115221	115682	-	462	Transcriptional activatory protein BadR
wy_00120	-	115753	116598	-	846	Ureidoglycolate lyase
wy_00121	*csiR*	116722	117384	+	663	HTH-type transcriptional repressor CsiR
wy_00122	*phrA*	117608	118939	+	1332	Deoxyribodipyrimidine photo-lyase
wy_00123	-	119034	119390	+	357	hypothetical protein

wy_00124	-	119557	119853	+	297	hypothetical protein
wy_00125	*stcD_1*	119950	121149	+	1200	putative N-methylproline demethylase
wy_00126	-	121162	122001	-	840	hypothetical protein
wy_00127	*acsA_1*	122238	124151	-	1914	Acetyl-coenzyme A synthetase
wy_00128	-	124318	125079	-	762	DNA alkylation repair enzyme
wy_00129	-	125076	125945	-	870	ANTAR domain protein
wy_00130	*cnrH*	125985	126545	-	561	RNA polymerase sigma factor CnrH
wy_00131	-	126884	127243	+	360	hypothetical protein
wy_00132	-	127268	127426	+	159	hypothetical protein
wy_00133	-	127453	127950	+	498	hypothetical protein
wy_00134	*yfmJ*	128021	129025	-	1005	Putative NADP-dependent oxidoreductase YfmJ
wy_00135	-	129054	129440	-	387	hypothetical protein
wy_00136	*comR_1*	129519	130130	+	612	HTH-type transcriptional repressor ComR
wy_00137	*accD*	130162	131667	-	1506	Acetyl-coenzyme A carboxylase carboxyl transferase subunit beta
wy_00138	-	131743	132945	-	1203	ABC-2 family transporter protein
wy_00139	*ybhF_1*	132942	133841	-	900	putative ABC transporter ATP-binding protein YbhF
wy_00140	-	133838	134389	-	552	hypothetical protein
wy_00141	-	134382	134681	-	300	helix-turn-helix protein
wy_00142	-	134810	135316	+	507	hypothetical protein
wy_00143	-	135434	136012	+	579	hypothetical protein
wy_00144	*efeN_1*	136005	137198	+	1194	putative deferrochelatase/peroxidase EfeN precursor
wy_00145	*yjjK_1*	137267	138928	-	1662	putative ABC transporter ATP-binding protein YjjK
wy_00146	-	139020	139634	+	615	Bacterial regulatory proteins, tetR family
wy_00147	-	139631	140647	+	1017	Phthiodiolone/phenolphthiodiolone dimycocerosates ketoreductase
wy_00148	*echA8_1*	140644	141456	+	813	putative enoyl-CoA hydratase echA8
wy_00149	*acr1_2*	141447	142376	+	930	Fatty acyl-CoA reductase

wy_00150	*acr1_3*	142429	143226	-	798	Fatty acyl-CoA reductase
wy_00151	*acr1_4*	143294	144091	-	798	Fatty acyl-CoA reductase
wy_00152	*ksdD_1*	144335	146041	+	1707	3-oxosteroid 1-dehydrogenase
wy_00153	*bchM_1*	146093	146698	-	606	Magnesium-protoporphyrin O-methyltransferase
wy_00154	-	146836	147672	+	837	putative oxidoreductase/MSMEI_2347
wy_00155	-	147783	148391	+	609	Transcriptional regulator PadR-like family protein
wy_00156	*emrB_1*	148388	149887	+	1500	Multidrug export protein EmrB
wy_00157	-	149964	150986	-	1023	AFG1-like ATPase
wy_00158	-	151027	151620	+	594	transcriptional regulator BetI
wy_00159	-	151710	153011	-	1302	HNH endonuclease
wy_00160	*ribH2*	153130	153567	+	438	6,7-dimethyl-8-ribityllumazine synthase 2
wy_00161	-	153693	154172	-	480	hypothetical protein
wy_00162	-	154304	155620	+	1317	hypothetical protein
wy_00163	*czcO_1*	155627	156697	-	1071	putative oxidoreductase CzcO
wy_00164	*echA6*	156866	157597	+	732	putative enoyl-CoA hydratase echA6
wy_00165	-	157614	158711	+	1098	metal-dependent hydrolase
wy_00166	*bglB*	158721	161099	-	2379	Thermostable beta-glucosidase B
wy_00167	*ctpF_1*	161179	163644	+	2466	putative cation-transporting ATPase F
wy_00168	*arpA_2*	163752	164402	+	651	A-factor receptor protein
wy_00169	-	164471	165157	+	687	hypothetical protein
wy_00170	-	165198	165389	+	192	Antitoxin/MT0933
wy_00171	*nhaK_1*	165467	167077	+	1611	Sodium, potassium, lithium and rubidium/H(+) antiporter
wy_00172	-	167107	168300	+	1194	putative inactive lipase/MT1628
wy_00173	*yehU*	168297	169505	+	1209	Sensor histidine kinase YehU
wy_00174	*lytR_1*	169564	170361	+	798	Sensory transduction protein LytR
wy_00175	-	170358	170732	+	375	hypothetical protein

wy_00176	*actP_1*	170743	172497	+	1755	Cation/acetate symporter ActP
wy_00177	*kshA_2*	172606	172968	+	363	3-ketosteroid-9-alpha-monooxygenase oxygenase subunit
wy_00178	*dan_1*	172998	174734	-	1737	D-aminoacylase
wy_00179	*yjcH*	175016	175378	+	363	Inner membrane protein YjcH
wy_00180	*actP_2*	175375	177018	+	1644	Cation/acetate symporter ActP
wy_00181	-	177109	177540	+	432	Toxin/MT0934
wy_00182	-	177610	179247	+	1638	hypothetical protein
wy_00183	*scoB_1*	179290	179937	-	648	Succinyl-CoA:3-ketoacid coenzyme A transferase subunit B
wy_00184	*scoA_1*	179934	180698	-	765	putative succinyl-CoA:3-ketoacid coenzyme A transferase subunit A
wy_00185	*yjjL*	180739	182109	-	1371	L-galactonate transporter
wy_00186	-	182192	182575	-	384	hypothetical protein
wy_00187	*virS_2*	182753	183781	-	1029	HTH-type transcriptional regulator VirS
wy_00188	*qorA_1*	184073	185050	+	978	Quinone oxidoreductase 1
wy_00189	*tauD_1*	185082	185981	+	900	Alpha-ketoglutarate-dependent taurine dioxygenase
wy_00190	*kce*	186025	187080	+	1056	3-keto-5-aminohexanoate cleavage enzyme
wy_00191	-	187183	187776	+	594	Alpha/beta hydrolase family protein
wy_00192	*lcfB_1*	187812	189347	+	1536	Long-chain-fatty-acid--CoA ligase
wy_00193	-	189464	190930	+	1467	Cellulase (glycosyl hydrolase family 5)
wy_00194	*kstR2_2*	190974	191597	-	624	HTH-type transcriptional repressor KstR2
wy_00195	*acdA_1*	191721	192866	+	1146	Acyl-CoA dehydrogenase
wy_00196	*glnH_1*	192897	193799	-	903	ABC transporter glutamine-binding protein GlnH precursor
wy_00197	-	193879	196119	+	2241	Putative DNA ligase-like protein/MT0965
wy_00198	*hspR_1*	196137	196487	-	351	Putative heat shock protein HspR
wy_00199	-	196758	197144	+	387	hypothetical protein
wy_00200	-	197152	197529	-	378	hypothetical protein
wy_0020	-	197757	197915	+	159	hypothetical protein

1						
wy_00202	-	198006	198383	+	378	hypothetical protein
wy_00204	-	199537	199782	+	246	hypothetical protein
wy_00205	*mepM_1*	199797	200630	+	834	Murein DD-endopeptidase MepM
wy_00206	-	200713	200961	-	249	hypothetical protein
wy_00207	*lin*	201097	201897	-	801	Linocin-M18
wy_00208	*yfeX*	201894	202916	-	1023	putative deferrochelatase/peroxidase YfeX
wy_00209	*hmp_1*	203007	204224	-	1218	Flavohemoprotein
wy_00210	*nsrR*	204256	204702	-	447	HTH-type transcriptional repressor NsrR
wy_00211	-	204955	205881	+	927	Universal stress protein/MT2061
wy_00212	-	205945	206973	+	1029	Phthiodiolone/phenolphthiodiolone dimycocerosates ketoreductase
wy_00213	-	207114	208010	+	897	Universal stress protein/MT2085
wy_00214	-	208082	209650	-	1569	Fusaric acid resistance protein family protein
wy_00215	-	209942	211144	+	1203	hypothetical protein
wy_00216	*spoVD*	211141	214014	+	2874	Stage V sporulation protein D
wy_00217	*mreB*	214015	215004	-	990	Rod shape-determining protein MreB
wy_00218	*htpG*	215170	217122	-	1953	Chaperone protein HtpG
wy_00219	-	217282	219606	+	2325	Putative HTH-type transcriptional regulator/MT0914
wy_00220	-	219612	220250	-	639	Stress responsive A/B Barrel Domain protein
wy_00221	-	220243	220599	-	357	hypothetical protein
wy_00222	*devR_1*	220751	221383	+	633	Transcriptional regulatory protein DevR (DosR)
wy_00223	*devS*	221452	223137	-	1686	Redox sensor histidine kinase response regulator DevS
wy_00224	-	223331	224047	+	717	hypothetical protein
wy_00225	-	224106	224720	+	615	Bacterial regulatory proteins, tetR family
wy_00226	-	224721	225047	-	327	hypothetical protein
wy_00227	*folA*	225156	225638	-	483	Dihydrofolate reductase
wy_0022	*thyA*	225638	226459	-	822	Thymidylate synthase

8						
wy_00229	-	226520	227260	+	741	precorrin 6A synthase
wy_00230	*fpg2*	227337	228203	+	867	putative formamidopyrimidine-DNA glycosylase-like protein
wy_00231	-	228186	228626	-	441	GtrA-like protein
wy_00232	*pnbA_1*	228685	230160	-	1476	Para-nitrobenzyl esterase
wy_00233	*pnbA_2*	230157	231680	-	1524	Para-nitrobenzyl esterase
wy_00234	-	231755	232360	-	606	hypothetical protein
wy_00235	-	232471	232875	+	405	hypothetical protein
wy_00236	*lcfB_2*	232989	234647	-	1659	Long-chain-fatty-acid--CoA ligase
wy_00237	-	234839	235474	-	636	RES domain protein
wy_00238	-	235480	236076	-	597	hypothetical protein
wy_00239	-	236191	236940	+	750	biotin biosynthesis protein BioC
wy_00240	-	237024	237170	-	147	hypothetical protein
wy_00241	*merA*	237323	238774	-	1452	Mercuric reductase
wy_00242	*drrB_1*	238825	239616	-	792	Daunorubicin/doxorubicin resistance ABC transporter permease protein DrrB
wy_00243	*drrA_1*	239613	240575	-	963	Daunorubicin/doxorubicin resistance ATP-binding protein DrrA
wy_00244	-	240608	241453	-	846	hypothetical protein
wy_00245	-	241495	242010	-	516	hypothetical protein
wy_00246	-	242097	243353	-	1257	hypothetical protein
wy_00247	*caiD_1*	243386	244153	-	768	Carnitinyl-CoA dehydratase
wy_00248	-	244323	245123	+	801	ApbE family protein
wy_00249	*pgi*	245126	246775	-	1650	Glucose-6-phosphate isomerase
wy_00250	*gabD_1*	246853	248316	+	1464	Succinate-semialdehyde dehydrogenase [NADP(+)] GabD
wy_00251	-	248407	248700	-	294	Intracellular chorismate mutase
wy_00252	*uvrD1*	249034	251508	+	2475	ATP-dependent DNA helicase UvrD1
wy_00253	*mepM_2*	251594	252595	-	1002	Murein DD-endopeptidase MepM

wy_00254	*sucC*	253142	254311	+	1170	Succinyl-CoA ligase [ADP-forming] subunit beta
wy_00255	*sucD*	254327	255229	+	903	Succinyl-CoA ligase [ADP-forming] subunit alpha
wy_00256	*yigZ*	255301	255930	-	630	IMPACT family member YigZ
wy_00257	-	256008	257405	+	1398	hypothetical protein
wy_00258	-	257498	258388	+	891	hypothetical protein
wy_00259	-	258483	260099	+	1617	hypothetical protein
wy_00260	*purN*	260187	260795	+	609	Phosphoribosylglycinamide formyltransferase
wy_00261	*purH*	260788	262374	+	1587	Bifunctional purine biosynthesis protein PurH
wy_00262	-	262418	262690	-	273	hypothetical protein
wy_00263	-	262770	262919	-	150	hypothetical protein
wy_00264	-	263049	264014	-	966	Alpha/beta hydrolase family protein
wy_00265	*bchI_1*	264191	265495	+	1305	Magnesium-chelatase 38 kDa subunit
wy_00266	-	265509	267458	+	1950	hypothetical protein
wy_00267	-	267465	267851	-	387	Glyoxalase/Bleomycin resistance protein/Dioxygenase superfamily protein
wy_00268	*sdpR_1*	267848	268165	-	318	Transcriptional repressor SdpR
wy_00269	*aam_1*	268321	269739	+	1419	Acylamidase
wy_00270	*liaR_1*	269761	270456	+	696	Transcriptional regulatory protein LiaR
wy_00271	-	270467	271393	-	927	putative transporter YfdV
wy_00272	*hspR_2*	271539	271859	+	321	Putative heat shock protein HspR
wy_00273	-	271908	272303	+	396	hypothetical protein
wy_00274	*yusV_1*	272321	273097	-	777	putative siderophore transport system ATP-binding protein YusV
wy_00275	*hmuU_1*	273094	274131	-	1038	Hemin transport system permease protein HmuU
wy_00276	-	274128	275315	-	1188	Periplasmic binding protein
wy_00277	*rpsR1*	275417	275671	-	255	30S ribosomal protein S18 1
wy_00278	*rpsN*	275682	275987	-	306	30S ribosomal protein S14
wy_00279	*rpmG2*	275992	276156	-	165	50S ribosomal protein L33 2

wy_00280	*rpmB_1*	276162	276398	-	237	50S ribosomal protein L28
wy_00281	*yciC_1*	276512	277741	+	1230	Putative metal chaperone YciC
wy_00282	*rpmE2*	277738	278007	+	270	50S ribosomal protein L31 type B
wy_00283	-	278011	278184	+	174	50S ribosomal protein L32
wy_00284	-	278218	278829	+	612	hypothetical protein
wy_00285	*mprA*	278892	279578	+	687	Response regulator MprA
wy_00286	*mprB*	279575	281044	+	1470	Signal transduction histidine-protein kinase/phosphatase MprB
wy_00287	*degP1*	281224	282609	+	1386	putative periplasmic serine endoprotease DegP-like precursor
wy_00288	*moaB*	282708	283184	+	477	Molybdenum cofactor biosynthesis protein B
wy_00289	-	283181	283348	+	168	hypothetical protein
wy_00290	*mscL*	283448	283885	-	438	Large-conductance mechanosensitive channel
wy_00291	-	283964	284668	-	705	SAF domain protein
wy_00292	-	284777	285061	-	285	Zinc ribbon domain protein
wy_00293	-	285175	285765	-	591	5-formyltetrahydrofolate cyclo-ligase family protein
wy_00294	*gtaB*	285944	286750	+	807	UTP--glucose-1-phosphate uridylyltransferase
wy_00295	*moeA_1*	286804	288063	+	1260	Molybdopterin molybdenumtransferase
wy_00296	*ydaF*	288023	288706	+	684	Putative ribosomal N-acetyltransferase YdaF
wy_00297	*mspA*	288761	289510	-	750	Porin MspA precursor
wy_00298	-	290292	290825	+	534	hypothetical protein
wy_00300	*espR*	291145	291561	-	417	Nucleoid-associated protein EspR
wy_00301	-	291784	291939	+	156	hypothetical protein
wy_00302	*sadH_1*	292070	292909	+	840	Putative oxidoreductase SadH
wy_00303	*ytrE*	292893	293585	-	693	ABC transporter ATP-binding protein YtrE
wy_00304	-	293612	294679	-	1068	FtsX-like permease family protein
wy_00305	*liaS_1*	294760	296016	+	1257	Sensor histidine kinase LiaS
wy_00306	*liaR_2*	296013	296639	+	627	Transcriptional regulatory protein LiaR

wy_00307	-	296662	297171	+	510	hypothetical protein
wy_00308	*soxR*	297183	297638	-	456	Redox-sensitive transcriptional activator SoxR
wy_00309	*arcA*	297736	298962	+	1227	Arginine deiminase
wy_00310	*arsC*	298974	299339	-	366	Arsenate reductase
wy_00311	*yhfK_1*	299412	300098	+	687	putative sugar epimerase YhfK
wy_00312	*ytrA_1*	300139	300498	+	360	HTH-type transcriptional repressor YtrA
wy_00313	*catD_1*	300552	300980	+	429	Putative oxidoreductase CatD
wy_00314	*betP*	301065	302621	+	1557	Glycine betaine transporter BetP
wy_00315	-	302634	303716	-	1083	hypothetical protein
wy_00316	*pmt*	303754	305313	-	1560	putative dolichyl-phosphate-mannose--protein mannosyltransferase
wy_00317	*rsmI*	305337	306167	+	831	Ribosomal RNA small subunit methyltransferase I
wy_00318	-	306217	306390	-	174	hypothetical protein
wy_00319	-	306682	310749	+	4068	translocation protein TolB
wy_00320	*pabB*	310670	311965	-	1296	Aminodeoxychorismate synthase component 1
wy_00321	-	312057	313319	-	1263	hypothetical protein
wy_00322	*metG*	313365	314951	-	1587	Methionine--tRNA ligase
wy_00323	*ycfH*	314972	315811	+	840	putative deoxyribonuclease YcfH
wy_00324	*yhhW_1*	315840	316832	-	993	Quercetin 2,3-dioxygenase
wy_00325	*rpfB_1*	317033	318160	+	1128	Resuscitation-promoting factor RpfB precursor
wy_00326	*rpfB_2*	318170	319297	+	1128	Resuscitation-promoting factor RpfB precursor
wy_00327	*rsmA_1*	319327	320217	+	891	Ribosomal RNA small subunit methyltransferase A
wy_00328	-	320334	320951	+	618	hypothetical protein
wy_00329	-	321099	321899	+	801	hypothetical protein
wy_00330	*mppA*	322024	323631	+	1608	Periplasmic murein peptide-binding protein precursor
wy_00331	*copA_1*	323704	325962	-	2259	Copper-exporting P-type ATPase A
wy_00332	-	325962	326894	-	933	hypothetical protein

wy_00333	*copZ_1*	326903	327112	-	210	Copper chaperone CopZ
wy_00334	*csp_1*	327451	327654	+	204	Cold shock protein
wy_00335	-	327807	328286	+	480	Putative lumazine-binding protein
wy_00336	-	328271	329239	-	969	HTH domain protein
wy_00337	*drrA_2*	329340	330308	+	969	Daunorubicin/doxorubicin resistance ATP-binding protein DrrA
wy_00338	*ybhR_1*	330358	331101	+	744	Inner membrane transport permease YbhR
wy_00339	*ispE*	331164	332099	+	936	4-diphosphocytidyl-2-C-methyl-D-erythritol kinase
wy_00340	-	332099	333889	+	1791	putative ABC transporter ATP-binding protein
wy_00341	*baiE_1*	334001	334495	+	495	Bile acid 7-alpha dehydratase
wy_00342	-	334492	334902	+	411	Glyoxalase-like domain protein
wy_00343	*pdxH_2*	334912	335616	+	705	Pyridoxine/pyridoxamine 5'-phosphate oxidase
wy_00344	*caiD_2*	335642	336676	+	1035	Carnitinyl-CoA dehydratase
wy_00345	*prrB_2*	336683	337975	-	1293	Sensor-type histidine kinase PrrB
wy_00346	*prrA_2*	337972	338664	-	693	Transcriptional regulatory protein PrrA
wy_00347	-	338695	339147	-	453	hypothetical protein
wy_00348	-	339268	340095	-	828	putative oxidoreductase/MSMEI_2347
wy_00349	*ydhP_1*	340139	341335	-	1197	Inner membrane transport protein YdhP
wy_00350	-	341481	341930	+	450	transcriptional regulator SlyA
wy_00351	-	341942	342811	-	870	Luciferase-like monooxygenase
wy_00352	-	342840	343508	-	669	Nicotinamide mononucleotide transporter
wy_00353	*prfC*	343505	345181	-	1677	Peptide chain release factor 3
wy_00354	-	345340	346968	+	1629	Putative ligase/MSMEI_5285
wy_00355	*selD*	346988	347992	-	1005	Selenide, water dikinase
wy_00357	*selA*	348198	349451	+	1254	L-seryl-tRNA(Sec) selenium transferase
wy_00358	*selB*	349451	351202	+	1752	Selenocysteine-specific elongation factor
wy_00359	-	351187	352122	-	936	Polysulfide reductase, NrfD

wy_0036 0	*fdnH*	352176	353123	-	948	Formate dehydrogenase, nitrate-inducible, iron-sulfur subunit
wy_0036 1	*fdoG*	353126	355690	-	2565	Formate dehydrogenase-O major subunit precursor
wy_0036 2	*fdhA_1*	355799	356368	-	570	Formate dehydrogenase subunit alpha precursor
wy_0036 3	*pth*	356459	357037	-	579	Peptidyl-tRNA hydrolase
wy_0036 4	*rplY*	357150	357743	-	594	50S ribosomal protein L25
wy_0036 5	*ifcA_1*	357872	359476	-	1605	Fumarate reductase flavoprotein subunit precursor
wy_0036 6	-	359658	360152	-	495	hypothetical protein
wy_0036 7	*csd*	360251	361429	-	1179	putative cysteine desulfurase
wy_0036 8	*gshA*	361517	362755	+	1239	Glutamate--cysteine ligase GshA
wy_0036 9	*egtB*	362752	364044	+	1293	Iron(II)-dependent oxidoreductase EgtB
wy_0037 0	*egtC*	364044	364772	+	729	Amidohydrolase EgtC
wy_0037 1	*egtD*	364830	365801	+	972	Histidine-specific methyltransferase EgtD
wy_0037 2	*yncA*	365815	366303	-	489	N-acyltransferase YncA
wy_0037 3	-	366317	367075	-	759	hypothetical protein
wy_0037 4	-	367150	367785	-	636	hypothetical protein
wy_0037 5	*prs*	367856	368836	-	981	Ribose-phosphate pyrophosphokinase
wy_0037 6	*glmU*	368833	370302	-	1470	Bifunctional protein GlmU
wy_0037 8	*icaR*	370860	371450	+	591	Biofilm operon icaADBC HTH-type negative transcriptional regulator IcaR
wy_0037 9	*mfd*	371547	375233	+	3687	Transcription-repair-coupling factor
wy_0038 0	*mazG*	375239	375925	+	687	Nucleoside triphosphate pyrophosphohydrolase
wy_0038 1	*yjjK_2*	375933	377540	-	1608	putative ABC transporter ATP-binding protein YjjK
wy_0038 2	*efeN_2*	377855	379171	-	1317	putative deferrochelatase/peroxidase EfeN precursor
wy_0038 3	*efeO*	379152	380312	-	1161	Iron uptake system component EfeO precursor
wy_0038 4	*efeU*	380309	381217	-	909	Ferrous iron permease EfeU
wy_0038 5	-	381411	382271	+	861	hypothetical protein
wy_0038 6	*eno*	382453	383739	+	1287	Enolase

wy_00387	*ftsL*	383748	384473	+	726	Cell division protein FtsL
wy_00388	-	384470	385006	+	537	hypothetical protein
wy_00389	*gppA_1*	385003	385959	+	957	Guanosine-5'-triphosphate,3'-diphosphate pyrophosphatase
wy_00391	-	386391	386864	+	474	hypothetical protein
wy_00392	-	387908	388348	+	441	hypothetical protein
wy_00393	*cspA_1*	388509	388712	+	204	putative cold shock protein A
wy_00394	-	388799	389137	-	339	hypothetical protein
wy_00395	*aidB*	389193	390830	-	1638	Putative acyl-CoA dehydrogenase AidB
wy_00396	*luxA_1*	390975	392063	+	1089	Alkanal monooxygenase alpha chain
wy_00397	*ssuE*	392060	392689	+	630	FMN reductase (NADPH)
wy_00398	*phbC*	392738	393793	+	1056	Poly-beta-hydroxybutyrate polymerase
wy_00399	*rlmG*	394019	395152	+	1134	Ribosomal RNA large subunit methyltransferase G
wy_00400	-	395156	395500	-	345	hypothetical protein
wy_00401	-	395736	396590	+	855	Bax inhibitor 1 like protein
wy_00402	*fadA_1*	396752	397969	-	1218	3-ketoacyl-CoA thiolase
wy_00403	-	398008	398754	-	747	GDSL-like Lipase/Acylhydrolase
wy_00404	*cbs*	399128	400510	+	1383	Putative cystathionine beta-synthase
wy_00405	*metB*	400605	401759	+	1155	Cystathionine gamma-synthase
wy_00406	*ripA_1*	401922	403280	+	1359	Peptidoglycan endopeptidase RipA precursor
wy_00407	-	403290	405161	+	1872	SEC-C motif protein
wy_00408	*greA*	405234	405728	-	495	Transcription elongation factor GreA
wy_00409	-	405975	406400	-	426	hypothetical protein
wy_00410	*mca_1*	406625	407485	+	861	Mycothiol S-conjugate amidase
wy_00411	-	407482	407820	+	339	hypothetical protein
wy_00412	-	407829	409805	+	1977	hypothetical protein
wy_00413	-	409903	410049	-	147	hypothetical protein

wy_00414	-	410180	410287	-	108	hypothetical protein
wy_00415	-	410661	410777	-	117	hypothetical protein
wy_00416	-	410894	411007	-	114	hypothetical protein
wy_00417	*fbpC_2*	411257	412288	-	1032	Diacylglycerol acyltransferase/mycolyltransferase Ag85C precursor
wy_00418	-	412459	412575	-	117	hypothetical protein
wy_00419	-	412687	412881	-	195	hypothetical protein
wy_00420	-	413006	414523	-	1518	Putative diacylglycerol O-acyltransferase/MT1468
wy_00421	-	414601	415269	-	669	hemolysin-III related
wy_00422	*uppS*	415447	416214	+	768	(2Z,6E)-farnesyl diphosphate synthase
wy_00423	*ttgW_1*	416248	416877	-	630	putative HTH-type transcriptional regulator TtgW
wy_00424	*pamO_1*	417024	418358	+	1335	Phenylacetone monooxygenase
wy_00425	*estB_1*	418355	419494	+	1140	Esterase EstB
wy_00426	*coaA*	419543	420475	-	933	Pantothenate kinase
wy_00427	-	420659	421903	+	1245	hypothetical protein
wy_00428	*glyA1*	421946	423256	+	1311	Serine hydroxymethyltransferase 1
wy_00429	-	423308	423922	-	615	uridine kinase
wy_00430	*ybeZ_1*	424220	425527	+	1308	PhoH-like protein
wy_00431	*kstD_1*	425905	427608	+	1704	3-oxosteroid 1-dehydrogenase
wy_00432	-	427595	428005	-	411	Carboxymuconolactone decarboxylase family protein
wy_00433	*ephG*	428070	428492	-	423	Epoxide hydrolase EphG
wy_00434	*fumC*	428521	429927	-	1407	Fumarate hydratase class II
wy_00435	*glpX*	430021	431061	-	1041	Fructose-1,6-bisphosphatase class 2
wy_00436	-	431190	431807	+	618	hypothetical protein
wy_00437	-	431827	432057	-	231	exodeoxyribonuclease VII small subunit
wy_00438	*xseA*	432054	433310	-	1257	Exodeoxyribonuclease 7 large subunit
wy_0043	-	433307	433909	-	603	hypothetical protein

9						
wy_00440	*ispH*	434029	435033	+	1005	4-hydroxy-3-methylbut-2-enyl diphosphate reductase
wy_00441	-	435046	435939	-	894	hypothetical protein
wy_00442	*sbcD*	436086	437279	+	1194	Nuclease SbcCD subunit D
wy_00443	*sbcC*	437276	440257	+	2982	Nuclease SbcCD subunit C
wy_00444	*tqsA_1*	440254	441495	-	1242	AI-2 transport protein TqsA
wy_00445	-	441638	442135	+	498	hypothetical protein
wy_00446	*gltA_2*	442163	443740	-	1578	Glutamate synthase [NADPH] large chain
wy_00447	*marR*	443839	444270	+	432	Multiple antibiotic resistance protein MarR
wy_00448	-	444348	444803	+	456	MarR family protein
wy_00449	*bmr3_2*	444874	446550	+	1677	Multidrug resistance protein 3
wy_00450	*bchM_2*	446577	447221	+	645	Magnesium-protoporphyrin O-methyltransferase
wy_00451	-	447288	447692	+	405	Interferon-induced transmembrane protein
wy_00452	*ychF*	447764	448843	+	1080	Ribosome-binding ATPase YchF
wy_00453	-	449257	450243	+	987	hypothetical protein
wy_00454	-	450484	451371	+	888	P-aminobenzoate N-oxygenase AurF
wy_00455	*fprA_1*	451384	452796	+	1413	NADPH-ferredoxin reductase FprA
wy_00456	*rluA_2*	452732	453727	-	996	Ribosomal large subunit pseudouridine synthase A
wy_00457	-	453820	454887	-	1068	Excalibur calcium-binding domain protein
wy_00458	-	455028	455297	-	270	hypothetical protein
wy_00459	*ssuA_1*	455662	456672	+	1011	Putative aliphatic sulfonates-binding protein precursor
wy_00460	*ssuC_1*	456669	457511	+	843	Putative aliphatic sulfonates transport permease protein SsuC
wy_00461	*ssuB_1*	457484	458218	+	735	Aliphatic sulfonates import ATP-binding protein SsuB
wy_00462	*msuD_1*	458215	459312	+	1098	Methanesulfonate monooxygenase
wy_00463	-	459397	459885	+	489	Fibronectin-binding protein (FBP)
wy_00464	-	459896	460786	-	891	hypothetical protein
wy_0046	*rtcB_1*	460853	461566	+	714	RNA-splicing ligase RtcB

5						
wy_00466	*rtcB_2*	461576	462151	+	576	RNA-splicing ligase RtcB
wy_00467	-	462218	462307	-	90	hypothetical protein
wy_00468	-	462439	462918	-	480	hypothetical protein
wy_00469	*acr1_5*	462940	463860	-	921	Fatty acyl-CoA reductase
wy_00470	-	464051	464527	+	477	3-demethylubiquinone-9 3-methyltransferase
wy_00471	*mug*	464568	465137	+	570	G/U mismatch-specific DNA glycosylase
wy_00472	-	465211	465813	+	603	Isoprenylcysteine carboxyl methyltransferase (ICMT) family protein
wy_00473	*resA_1*	465868	466467	+	600	Thiol-disulfide oxidoreductase ResA
wy_00474	*adhT_1*	466483	467385	-	903	Alcohol dehydrogenase
wy_00475	*fumB*	467656	469353	-	1698	Fumarate hydratase class I, anaerobic
wy_00476	-	469588	470349	+	762	CAAX amino terminal protease self- immunity
wy_00477	*rutR_1*	470480	471055	+	576	HTH-type transcriptional regulator RutR
wy_00478	*hapE_1*	471666	472580	+	915	4-hydroxyacetophenone monooxygenase
wy_00479	*choD_1*	472577	474316	+	1740	Cholesterol oxidase
wy_00480	-	474304	474957	+	654	Acetoacetate decarboxylase (ADC)
wy_00481	-	474967	476112	-	1146	hypothetical protein
wy_00482	*kstR2_3*	476145	476741	+	597	HTH-type transcriptional repressor KstR2
wy_00483	*nlhH_2*	476745	477671	-	927	Carboxylesterase NlhH
wy_00484	-	477699	478634	-	936	Putative short-chain type dehydrogenase/reductase
wy_00485	*hapE_2*	478870	480732	+	1863	4-hydroxyacetophenone monooxygenase
wy_00486	*nlhH_3*	480947	482014	+	1068	Carboxylesterase NlhH
wy_00487	-	481937	482845	-	909	hypothetical protein
wy_00488	-	482880	483071	+	192	hypothetical protein
wy_00489	*pspB_1*	483163	483939	-	777	Putative phosphoserine phosphatase 2
wy_00490	-	484200	484781	-	582	hypothetical protein
wy_0049	*bgaB*	484865	485998	+	1134	Beta-galactosidase bgaB

1						
wy_00492	-	486004	486273	+	270	hypothetical protein
wy_00493	*frc_1*	486277	487461	-	1185	Formyl-coenzyme A transferase
wy_00494	*oxc*	487458	489140	-	1683	putative oxalyl-CoA decarboxylase
wy_00495	*ifcA_2*	489281	490762	-	1482	Fumarate reductase flavoprotein subunit precursor
wy_00496	-	490849	491265	-	417	Carboxymuconolactone decarboxylase family protein
wy_00497	*garR_2*	491262	492158	-	897	2-hydroxy-3-oxopropionate reductase
wy_00498	*hdhA_1*	492155	492904	-	750	7-alpha-hydroxysteroid dehydrogenase
wy_00499	-	492932	494404	-	1473	Putative aldehyde dehydrogenase
wy_00500	-	494524	495381	+	858	Putative short-chain type dehydrogenase/reductase/MSMEI_5872
wy_00501	-	495411	495992	+	582	Bacterial regulatory proteins, tetR family
wy_00502	-	495997	497202	+	1206	Cytochrome P450 107B1
wy_00503	*fabG1_1*	497202	498002	+	801	3-oxoacyl-[acyl-carrier-protein] reductase FabG1
wy_00504	-	497999	499420	+	1422	Lanosterol 14-alpha demethylase
wy_00505	*suaB*	499423	499617	+	195	Ferredoxin-1
wy_00506	-	499667	500218	+	552	hypothetical protein
wy_00507	-	500215	501474	+	1260	hypothetical protein
wy_00508	-	501504	503129	+	1626	Long-chain-fatty-acid--CoA ligase FadD19
wy_00511	-	506629	507285	+	657	Sortase family protein
wy_00512	-	507286	507507	-	222	hypothetical protein
wy_00513	*nfdA_1*	507693	509309	+	1617	N-substituted formamide deformylase precursor
wy_00514	-	509378	510478	-	1101	hypothetical protein
wy_00515	-	510623	511207	+	585	transcriptional regulator BetI
wy_00516	*ifcA_3*	511329	512765	+	1437	Fumarate reductase flavoprotein subunit precursor
wy_00517	*mrr*	512887	513855	+	969	Mrr restriction system protein
wy_00518	-	513983	514717	+	735	hypothetical protein
wy_0051	-	514809	515171	+	363	hypothetical protein

9						
wy_00520	-	515243	515743	-	501	SnoaL-like domain protein
wy_00521	-	515854	516096	-	243	hypothetical protein
wy_00522	-	516264	516353	+	90	hypothetical protein
wy_00523	*bmr3_3*	516425	517864	-	1440	Multidrug resistance protein 3
wy_00524	-	518028	518447	+	420	MarR family protein
wy_00525	-	518444	518782	-	339	hypothetical protein
wy_00526	-	518889	519698	-	810	hypothetical protein
wy_00527	*pimE*	520030	521298	+	1269	Polyprenol-phosphate-mannose-dependent alpha-(1-2)-phosphatidylinositol pentamannoside mannosyltransferase
wy_00528	-	521240	521530	-	291	Putative pterin-4-alpha-carbinolamine dehydratase
wy_00529	*nudG*	521564	521956	+	393	CTP pyrophosphohydrolase
wy_00530	-	521966	522148	-	183	hypothetical protein
wy_00531	*typA*	522547	524442	+	1896	GTP-binding protein TypA/BipA
wy_00532	-	524535	526343	+	1809	putative monoacyl phosphatidylinositol tetramannoside-binding protein LpqW precursor
wy_00533	*mshB_1*	526349	527224	+	876	1D-myo-inositol 2-acetamido-2-deoxy-alpha-D-glucopyranoside deacetylase
wy_00534	-	527311	527469	+	159	hypothetical protein
wy_00535	-	527519	527929	+	411	hypothetical protein
wy_00536	-	527920	528858	-	939	Bacterial membrane flanked domain protein
wy_00537	-	529453	529977	-	525	Bacterial membrane flanked domain protein
wy_00538	-	530004	530273	+	270	hypothetical protein
wy_00539	*mqnE*	530314	532920	+	2607	Aminodeoxyfutalosine synthase
wy_00540	-	532984	533790	+	807	metal-dependent hydrolase
wy_00541	*ydaP*	533835	534890	-	1056	Putative thiamine pyrophosphate-containing protein YdaP
wy_00542	*fdxA_1*	535214	535537	+	324	Ferredoxin
wy_00543	*alaC*	535568	536653	+	1086	Glutamate-pyruvate aminotransferase AlaC

wy_00544	*rocA1*	536856	538505	+	1650	1-pyrroline-5-carboxylate dehydrogenase 1
wy_00545	*fadM*	538502	539470	+	969	Proline dehydrogenase 1
wy_00546	*lcfB_3*	539496	540899	+	1404	Long-chain-fatty-acid--CoA ligase
wy_00547	*mmgB*	541099	541956	-	858	putative 3-hydroxybutyryl-CoA dehydrogenase
wy_00548	*curA*	542140	543081	+	942	NADPH-dependent curcumin reductase
wy_00549	*tauD_2*	543085	544047	-	963	Alpha-ketoglutarate-dependent taurine dioxygenase
wy_00550	-	544044	544676	-	633	hypothetical protein
wy_00551	-	544755	545411	+	657	hypothetical protein
wy_00552	*echA8_2*	545408	546217	-	810	putative enoyl-CoA hydratase echA8
wy_00553	*gabP*	546424	547758	-	1335	GABA permease
wy_00554	*dapD*	547829	548743	-	915	2,3,4,5-tetrahydropyridine-2,6-dicarboxylate N-succinyltransferase
wy_00555	*dapE_1*	548899	549996	+	1098	Succinyl-diaminopimelate desuccinylase
wy_00556	*yvdD_1*	550084	550821	+	738	LOG family protein YvdD
wy_00557	*fas6*	550815	551375	+	561	LOG family protein ORF6 in fasciation locus
wy_00558	-	551465	553240	+	1776	Long-chain-fatty-acid--CoA ligase FadD17
wy_00559	*folP2*	553270	554196	+	927	Inactive dihydropteroate synthase 2
wy_00560	*gpgS*	554193	555116	+	924	Glucosyl-3-phosphoglycerate synthase
wy_00561	-	555166	555495	+	330	hypothetical protein
wy_00562	*tag*	555492	556064	+	573	DNA-3-methyladenine glycosylase 1
wy_00563	-	556215	556382	+	168	hypothetical protein
wy_00564	*rlmAII*	556518	557372	+	855	23S rRNA (guanine(748)-N(1))-methyltransferase
wy_00565	*glgA*	557345	558505	-	1161	Capsular glucan synthase
wy_00566	*glgC*	558636	559832	+	1197	Glucose-1-phosphate adenylyltransferase
wy_00567	-	559836	560468	-	633	Putative O-methyltransferase/MSMEI_4947
wy_00568	*sigE*	560739	561413	+	675	ECF RNA polymerase sigma factor SigE
wy_00569	*rseA*	561432	561794	+	363	Anti-sigma-E factor RseA

wy_00570	*hhoB*	561873	563369	+	1497	Putative serine protease HhoB precursor
wy_00571	*tatB*	563442	563846	+	405	Sec-independent protein translocase protein TatB
wy_00572	*ylxH*	563921	565057	-	1137	Flagellum site-determining protein YlxH
wy_00573	-	565081	566193	-	1113	hypothetical protein
wy_00574	-	566368	566907	-	540	hypothetical protein
wy_00575	-	566900	568174	-	1275	Magnesium transporter MgtE
wy_00576	*mcl1*	568230	569189	+	960	Malyl-CoA lyase
wy_00577	-	569288	569776	+	489	hypothetical protein
wy_00578	*bmr3_4*	569707	571218	-	1512	Multidrug resistance protein 3
wy_00579	-	571403	571966	+	564	Suppressor of fused protein (SUFU)
wy_00580	-	572088	572984	+	897	hypothetical protein
wy_00581	-	572981	573301	+	321	hypothetical protein
wy_00582	-	573647	574837	+	1191	NAD-dependent malic enzyme
wy_00583	*bpoA2*	574951	575826	+	876	Non-heme bromoperoxidase BPO-A2
wy_00584	*opuCB_1*	575789	576496	-	708	Glycine betaine/carnitine/choline transport system permease protein OpuCB
wy_00585	*opuBB_1*	576496	577185	-	690	Choline transport system permease protein OpuBB
wy_00586	*opuCA_1*	577190	578311	-	1122	Carnitine transport ATP-binding protein OpuCA
wy_00587	*osmF_1*	578308	579255	-	948	Putative osmoprotectant uptake system substrate-binding protein OsmF precursor
wy_00588	-	579445	580371	+	927	Substrate binding domain of ABC-type glycine betaine transport system
wy_00589	*lipR_1*	580330	581265	-	936	Putative acetyl-hydrolase LipR precursor
wy_00590	*sadH_2*	581280	582110	-	831	Putative oxidoreductase SadH
wy_00591	*hapE_3*	582107	583693	-	1587	4-hydroxyacetophenone monooxygenase
wy_00592	*kstR2_4*	583765	584376	+	612	HTH-type transcriptional repressor KstR2
wy_00593	*kgd*	584545	588315	-	3771	Multifunctional 2-oxoglutarate metabolism enzyme
wy_00594	-	588428	592159	-	3732	Putative multidrug export ATP-binding/permease protein
wy_0059	-	592326	593090	-	765	ABC-2 family transporter protein

5						
wy_00596	*drrA_3*	593087	594130	-	1044	Daunorubicin/doxorubicin resistance ATP-binding protein DrrA
wy_00597	-	594266	594967	-	702	hypothetical protein
wy_00598	-	595051	595449	-	399	hypothetical protein
wy_00599	*mhpC_1*	595480	596298	-	819	2-hydroxy-6-oxononadienedioate/2-hydroxy-6-oxononatrienedioate hydrolase
wy_00600	-	596332	597072	-	741	hypothetical protein
wy_00601	-	597232	597618	+	387	Glyoxalase-like domain protein
wy_00602	*yqjG*	597626	598696	-	1071	Glutathionyl-hydroquinone reductase YqjG
wy_00603	-	598726	599430	-	705	hypothetical protein
wy_00604	-	599525	601888	+	2364	Putative HTH-type transcriptional regulator/MT0914
wy_00605	*yvaM*	602006	602887	+	882	AB hydrolase superfamily protein YvaM
wy_00606	*fadB_1*	602910	603728	+	819	putative enoyl-CoA hydratase
wy_00607	*lcfB_4*	603814	605328	+	1515	Long-chain-fatty-acid--CoA ligase
wy_00608	*caiD_3*	605318	606079	+	762	Carnitinyl-CoA dehydratase
wy_00609	-	606115	607053	+	939	Adenine deaminase
wy_00610	-	607143	607403	-	261	hypothetical protein
wy_00611	-	607488	607727	-	240	CsbD-like protein
wy_00612	*dps*	607786	608325	-	540	DNA protection during starvation protein
wy_00613	-	608536	609408	+	873	hypothetical protein
wy_00614	-	609433	610689	+	1257	Major Facilitator Superfamily protein
wy_00615	*fadA_2*	610751	611923	-	1173	3-ketoacyl-CoA thiolase
wy_00616	*gatA_1*	612011	613369	-	1359	Glutamyl-tRNA(Gln) amidotransferase subunit A
wy_00617	-	613404	614714	-	1311	L-gulono-1,4-lactone dehydrogenase
wy_00618	*ttgR_1*	614792	615463	+	672	HTH-type transcriptional regulator TtgR
wy_00619	-	615523	616701	+	1179	D-threonine aldolase
wy_00620	*recD_1*	616671	620201	-	3531	RecBCD enzyme subunit RecD

wy_00621	-	620302	620937	+	636	Alpha/beta hydrolase family protein
wy_00623	-	621771	623558	-	1788	Long-chain-fatty-acid--CoA ligase FadD15
wy_00624	-	623724	624458	-	735	hypothetical protein
wy_00625	-	624557	625195	-	639	Putative phosphoribosyl transferase/MT0597
wy_00626	-	625328	626491	+	1164	Lactate 2-monooxygenase
wy_00627	*trpB_1*	626559	627842	-	1284	Tryptophan synthase beta chain
wy_00628	*deaD_1*	627987	629819	+	1833	ATP-dependent RNA helicase DeaD
wy_00629	-	629994	630596	+	603	putative endopeptidase precursor
wy_00630	-	630622	632511	-	1890	putative ABC transporter ATP-binding protein
wy_00631	-	632614	633243	-	630	hypothetical protein
wy_00632	*end*	633329	634105	-	777	putative endonuclease 4
wy_00633	*butA_1*	634221	634982	+	762	Diacetyl reductase [(S)-acetoin forming]
wy_00634	-	634979	635560	+	582	hypothetical protein
wy_00635	*atsA_1*	635632	638031	+	2400	Arylsulfatase
wy_00636	*bceA_1*	638157	638915	+	759	Bacitracin export ATP-binding protein BceA
wy_00637	*macB_1*	638920	640683	+	1764	Macrolide export ATP-binding/permease protein MacB
wy_00638	*ytrF*	640680	641447	+	768	ABC transporter permease YtrF precursor
wy_00639	-	641517	641960	+	444	hypothetical protein
wy_00640	-	641902	642426	-	525	Hydrogenase maturation protease
wy_00641	*hoxH*	642423	643742	-	1320	NAD-reducing hydrogenase HoxS subunit beta
wy_00642	*hoxY*	643732	644523	-	792	NAD-reducing hydrogenase HoxS subunit delta
wy_00643	*asrB*	644523	645356	-	834	Anaerobic sulfite reductase subunit B
wy_00644	-	645353	645799	-	447	Cyclic nucleotide-binding domain protein
wy_00645	*asrA*	645796	646929	-	1134	Anaerobic sulfite reductase subunit A
wy_00646	*pfkB*	647070	648116	+	1047	Putative phosphofructokinase PfkB
wy_00647	*ctpF_2*	648094	650712	+	2619	putative cation-transporting ATPase F

wy_00648	*devR_2*	650720	651355	-	636	Transcriptional regulatory protein DevR (DosR)
wy_00649	*ppsA_1*	651598	653916	+	2319	Phosphoenolpyruvate synthase
wy_00650	*hspX*	653961	654380	-	420	Alpha-crystallin
wy_00651	*acdA_2*	654603	655778	+	1176	Acyl-CoA dehydrogenase
wy_00652	*acrC*	655788	656921	+	1134	Acryloyl-CoA reductase (NADH)
wy_00653	-	656975	659845	+	2871	regulatory protein
wy_00654	-	660087	661547	+	1461	hypothetical protein
wy_00655	-	661653	662462	-	810	hypothetical protein
wy_00656	-	662866	664665	-	1800	Reverse transcriptase
wy_00657	-	664785	665315	-	531	endonuclease
wy_00658	-	665303	665938	-	636	hypothetical protein
wy_00659	-	666237	667403	+	1167	lipase
wy_00660	*helD*	667619	669838	+	2220	Helicase IV
wy_00661	*yngG*	669843	670775	-	933	Hydroxymethylglutaryl-CoA lyase YngG
wy_00662	*fldA_1*	670775	672076	-	1302	E-cinnamoyl-CoA:R-phenyllactate CoA transferase
wy_00663	*fabL*	672157	673020	+	864	Enoyl-[acyl-carrier-protein] reductase [NADPH] FabL
wy_00664	-	673231	673431	+	201	hypothetical protein
wy_00665	-	673490	674662	+	1173	hypothetical protein
wy_00666	-	674807	675307	+	501	hypothetical protein
wy_00667	*pdxS_1*	675393	676325	-	933	Pyridoxal biosynthesis lyase PdxS
wy_00668	*kstR2_5*	676369	676959	-	591	HTH-type transcriptional repressor KstR2
wy_00669	*epsN*	677195	678511	+	1317	Putative pyridoxal phosphate-dependent aminotransferase EpsN
wy_00670	-	678707	679942	+	1236	Amidohydrolase
wy_00671	*ped*	679939	680799	+	861	(S)-1-Phenylethanol dehydrogenase
wy_00672	-	680796	681659	+	864	Amidohydrolase
wy_00673	-	681666	683867	+	2202	Acyl-CoA dehydrogenase fadE12

wy_00674	*fabG_3*	683864	684739	+	876	3-oxoacyl-[acyl-carrier-protein] reductase FabG
wy_00675	*kstR_2*	684756	685370	+	615	HTH-type transcriptional repressor KstR
wy_00676	*kshA_3*	685463	686581	-	1119	3-ketosteroid-9-alpha-monooxygenase oxygenase subunit
wy_00677	*dppA_1*	687074	688690	+	1617	Periplasmic dipeptide transport protein precursor
wy_00678	*rhmR*	688744	689631	+	888	putative HTH-type transcriptional regulator RhmR
wy_00679	-	689693	690646	+	954	hypothetical protein
wy_00680	*xecD*	690679	691365	-	687	2-(R)-hydroxypropyl-CoM dehydrogenase
wy_00681	-	691440	691772	-	333	hypothetical protein
wy_00682	*paaF_1*	692001	693257	-	1257	2,3-dehydroadipyl-CoA hydratase
wy_00683	*bbsF_1*	693325	694584	+	1260	Succinyl-CoA:(R)-benzylsuccinate CoA-transferase subunit BbsF
wy_00684	*nfdA_2*	694581	695990	+	1410	N-substituted formamide deformylase precursor
wy_00685	-	696166	696462	+	297	hypothetical protein
wy_00686	*lcfB_5*	696471	697973	+	1503	Long-chain-fatty-acid--CoA ligase
wy_00687	*bbsF_2*	697995	700394	+	2400	Succinyl-CoA:(R)-benzylsuccinate CoA-transferase subunit BbsF
wy_00688	-	700416	701582	+	1167	thiolase
wy_00689	-	701579	702031	+	453	hypothetical protein
wy_00690	*fabG_4*	702028	702921	+	894	3-oxoacyl-[acyl-carrier-protein] reductase FabG
wy_00691	-	703009	704172	+	1164	Amidohydrolase
wy_00692	-	704169	704432	+	264	hypothetical protein
wy_00693	-	704435	705568	+	1134	D-arabitol-phosphate dehydrogenase
wy_00694	-	705658	706173	-	516	Excalibur calcium-binding domain protein
wy_00695	-	706640	706861	+	222	hypothetical protein
wy_00696	-	706875	707843	-	969	hypothetical protein
wy_00697	*fbpA_2*	708158	709315	+	1158	Diacylglycerol acyltransferase/mycolyltransferase Ag85A precursor
wy_00698	-	709326	709916	-	591	hypothetical protein

wy_00699	-	710035	710196	-	162	hypothetical protein
wy_00700	*atzF*	710578	711861	+	1284	Allophanate hydrolase
wy_00701	*ssuA_2*	712030	713019	+	990	Putative aliphatic sulfonates-binding protein precursor
wy_00702	*cmpB_1*	713012	713830	+	819	Bicarbonate transport system permease protein CmpB
wy_00703	*cmpD_1*	713868	714683	+	816	Bicarbonate transport ATP-binding protein CmpD
wy_00704	-	714707	715411	+	705	transcriptional regulator NanR
wy_00705	-	715503	715775	-	273	hypothetical protein
wy_00706	-	715878	716387	+	510	MarR family protein
wy_00707	-	716380	718173	+	1794	Long-chain-fatty-acid--CoA ligase FadD15
wy_00708	*cobB_1*	718184	718936	-	753	NAD-dependent protein deacylase
wy_00709	-	719039	719911	+	873	Uracil DNA glycosylase superfamily protein
wy_00710	-	720037	720555	+	519	hypothetical protein
wy_00711	*kstD_2*	720651	722189	+	1539	3-oxosteroid 1-dehydrogenase
wy_00712	-	722290	722997	-	708	hypothetical protein
wy_00713	*cmpC*	723219	723980	-	762	Bicarbonate transport ATP-binding protein CmpC
wy_00714	*ssuC_2*	723973	724788	-	816	Putative aliphatic sulfonates transport permease protein SsuC
wy_00715	-	724766	725785	-	1020	Putative thiamine biosynthesis protein
wy_00716	*tenA*	725791	726519	-	729	Thiaminase-2
wy_00717	*pnbA_3*	726700	728268	+	1569	Para-nitrobenzyl esterase
wy_00718	-	728261	728896	-	636	5,6-dimethylbenzimidazole synthase
wy_00719	-	728965	729261	-	297	hypothetical protein
wy_00720	-	729258	730523	-	1266	putative multidrug-efflux transporter/MT1297
wy_00721	-	730625	733132	-	2508	outer membrane-specific lipoprotein transporter subunit LolE
wy_00722	-	733135	733905	-	771	putative ABC transporter ATP-binding protein/MT1014
wy_00723	*birA_1*	733991	734980	-	990	Bifunctional ligase/repressor BirA
wy_00724	-	735084	735560	+	477	hypothetical protein

wy_00725	*degS_1*	735605	736855	+	1251	Signal transduction histidine-protein kinase/phosphatase DegS
wy_00726	*nreC_1*	736843	737487	+	645	Oxygen regulatory protein NreC
wy_00727	-	737495	737839	-	345	hypothetical protein
wy_00728	-	737836	742101	-	4266	Calcium-transporting ATPase
wy_00729	-	742102	742554	-	453	hypothetical protein
wy_00730	*ssuA_3*	742841	743875	+	1035	Putative aliphatic sulfonates-binding protein precursor
wy_00731	*ssuC_3*	743878	744735	+	858	Putative aliphatic sulfonates transport permease protein SsuC
wy_00732	*ssuB_2*	744732	745544	+	813	Aliphatic sulfonates import ATP-binding protein SsuB
wy_00733	*soxB*	745541	746578	+	1038	2'-hydroxybiphenyl-2-sulfinate desulfinase
wy_00734	*msuD_2*	746575	747621	+	1047	Methanesulfonate monooxygenase
wy_00735	*msuD_3*	747618	748775	+	1158	Methanesulfonate monooxygenase
wy_00736	-	748786	749493	+	708	hypothetical protein
wy_00737	*ssuA_4*	749518	750462	+	945	Putative aliphatic sulfonates-binding protein precursor
wy_00738	-	750565	751452	+	888	spermidine synthase
wy_00739	-	751814	753118	-	1305	HNH endonuclease
wy_00740	-	753150	753545	-	396	hypothetical protein
wy_00741	-	753608	754015	-	408	OsmC-like protein
wy_00742	*ntaA_1*	754012	755376	-	1365	Nitrilotriacetate monooxygenase component A
wy_00743	*ssuA_5*	755385	756464	-	1080	Putative aliphatic sulfonates-binding protein precursor
wy_00744	*ssuB_3*	756502	757236	-	735	Aliphatic sulfonates import ATP-binding protein SsuB
wy_00745	*ssuC_4*	757233	758081	-	849	Putative aliphatic sulfonates transport permease protein SsuC
wy_00746	*lutR_1*	758154	758897	+	744	HTH-type transcriptional regulator LutR
wy_00747	*echA8_3*	758898	759683	-	786	putative enoyl-CoA hydratase echA8
wy_00748	-	759826	760752	+	927	SPFH domain / Band 7 family protein
wy_00749	-	760764	760967	+	204	hypothetical protein
wy_0075	*gloB_1*	761012	762010	-	999	Hydroxyacylglutathione hydrolase

0						
wy_0075 1	-	762117	762395	-	279	hypothetical protein
wy_0075 2	-	762586	763050	+	465	hypothetical protein
wy_0075 3	-	763067	763438	+	372	hypothetical protein
wy_0075 4	-	763460	763837	+	378	hypothetical protein
wy_0075 5	-	763912	764190	+	279	hypothetical protein
wy_0075 6	-	764203	765606	-	1404	hypothetical protein
wy_0075 7	-	765603	766052	-	450	hypothetical protein
wy_0075 8	-	766184	767203	-	1020	hypothetical protein
wy_0075 9	-	767330	767986	-	657	hypothetical protein
wy_0076 0	*draG*	768051	769019	+	969	ADP-ribosyl-[dinitrogen reductase] glycohydrolase
wy_0076 1	*ymdB*	769016	769525	+	510	O-acetyl-ADP-ribose deacetylase
wy_0076 2	*lutR_2*	769527	770258	-	732	HTH-type transcriptional regulator LutR
wy_0076 3	*lutP*	770294	771961	-	1668	L-lactate permease
wy_0076 4	*lutA*	772176	772931	+	756	Lactate utilization protein A
wy_0076 5	*lutB*	772928	774442	+	1515	Lactate utilization protein B
wy_0076 6	*lutC*	774439	775062	+	624	Lactate utilization protein C
wy_0076 7	-	775141	775434	+	294	hypothetical protein
wy_0076 8	-	775431	776432	+	1002	hypothetical protein
wy_0076 9	-	776423	777358	+	936	hypothetical protein
wy_0077 0	-	777355	777903	+	549	putative lipoprotein LpqN
wy_0077 1	-	778087	778473	-	387	Glyoxalase-like domain protein
wy_0077 2	-	778485	779282	-	798	Helix-turn-helix domain protein
wy_0077 3	-	779343	779930	-	588	hypothetical protein
wy_0077 4	*recC*	780071	783328	+	3258	RecBCD enzyme subunit RecC
wy_0077 5	*recB*	783325	786645	+	3321	RecBCD enzyme subunit RecB
wy_0077	*recD_2*	786642	788486	+	1845	RecBCD enzyme subunit RecD

6						
wy_00777	-	789177	789467	-	291	hypothetical protein
wy_00778	-	789759	790154	-	396	hypothetical protein
wy_00779	-	790204	791442	-	1239	hypothetical protein
wy_00780	-	791439	792128	-	690	hypothetical protein
wy_00781	-	792654	795353	+	2700	hypothetical protein
wy_00782	-	795894	796616	-	723	hypothetical protein
wy_00783	-	796799	797266	+	468	hypothetical protein
wy_00784	*hflK*	797260	798132	+	873	Modulator of FtsH protease HflK
wy_00785	-	798139	798723	-	585	hypothetical protein
wy_00786	-	798837	799547	+	711	hypothetical protein
wy_00787	-	799733	800623	+	891	hypothetical protein
wy_00788	-	800631	801563	+	933	hypothetical protein
wy_00789	-	801550	802593	+	1044	hypothetical
wy_00790	*Type II R*	803407	804336	-	930	Type-2 restriction enzyme NaeI
wy_00791	*Type II M*	804385	805653	-	1269	Modification methylase AplI
wy_00792	-	805922	806698	-	777	hypothetical protein
wy_00793	-	806986	808365	-	1380	oxidoreductase
wy_00794	-	808375	809082	+	708	hypothetical protein
wy_00795	-	809102	809842	+	741	hypothetical protein
wy_00796	-	809949	810473	+	525	hypothetical protein
wy_00797	-	810493	810855	+	363	hypothetical protein
wy_00798	-	810949	812442	+	1494	PPE family protein
wy_00799	-	812465	813055	-	591	hypothetical protein
wy_00800	*plsC_1*	813069	814508	-	1440	1-acyl-sn-glycerol-3-phosphate acyltransferase
wy_00801	*plsB*	814505	816805	-	2301	Glycerol-3-phosphate acyltransferase
wy_0080	-	816802	818289	-	1488	Putative diacylglycerol O-

2						acyltransferase/MT2557
wy_00803	-	818541	818771	+	231	hypothetical protein
wy_00804	*amdA*	818802	820055	+	1254	Acetamidase
wy_00805	-	820409	820780	+	372	hypothetical protein
wy_00806	-	820738	821397	-	660	putative peptidase
wy_00807	-	821401	822222	-	822	hypothetical protein
wy_00808	-	822219	822950	-	732	DSBA-like thioredoxin domain protein
wy_00809	*lcfB_6*	823119	824657	+	1539	Long-chain-fatty-acid--CoA ligase
wy_00810	-	824672	825034	-	363	hypothetical protein
wy_00811	*pntAA*	825304	826419	+	1116	NAD(P) transhydrogenase subunit alpha part 1
wy_00812	*pntA*	826420	826746	+	327	NAD(P) transhydrogenase subunit alpha
wy_00813	*pntB*	826746	828176	+	1431	NAD(P) transhydrogenase subunit beta
wy_00814	*ybjJ_1*	828312	829499	-	1188	Inner membrane protein YbjJ
wy_00815	*bkdC*	829619	830731	-	1113	Dihydrolipoyllysine-residue acyltransferase component of branched-chain alpha-ketoacid dehydrogenase complex
wy_00816	*bkdB*	830728	831729	-	1002	3-methyl-2-oxobutanoate dehydrogenase subunit beta
wy_00817	*bkdA*	831726	832349	-	624	3-methyl-2-oxobutanoate dehydrogenase subunit alpha
wy_00818	*lrp_1*	832951	833502	+	552	Leucine-responsive regulatory protein
wy_00819	-	833621	834649	+	1029	Phosphotransferase enzyme family protein
wy_00820	-	834657	835115	-	459	hypothetical protein
wy_00821	-	835144	835626	-	483	hypothetical protein
wy_00822	-	835636	836430	-	795	Shikimate 5-dehydrogenase-like protein
wy_00823	-	836522	837226	-	705	hypothetical protein
wy_00824	-	837305	837622	-	318	hypothetical protein
wy_00825	-	837711	840518	+	2808	ATP-dependent helicase HepA
wy_00826	-	840515	841318	+	804	hypothetical protein
wy_00827	*yxeP*	841336	842607	-	1272	putative hydrolase YxeP

wy_00828	-	843016	843567	-	552	hypothetical protein
wy_00829	-	843649	844014	+	366	hypothetical protein
wy_00830	*yxlF*	844011	844730	+	720	putative ABC transporter ATP-binding protein YxlF
wy_00831	-	844727	845524	+	798	ABC
wy_00832	-	845684	845947	-	264	hypothetical protein
wy_00833	*RR-NarL*	846459	847130	-	672	Oxygen regulatory protein NreC
wy_00834	*HK-Classic*	847115	848449	-	1335	Oxygen sensor histidine kinase NreB
wy_00835	*ABC*	848453	849469	+	1017	putative ABC transporter ATP-binding protein YxlF
wy_00836	-	849466	851004	+	1539	hypothetical protein
wy_00837	-	850991	851632	+	642	hypothetical protein
wy_00838	-	852278	852412	+	135	hypothetical protein
wy_00839	-	852470	852823	+	354	transcriptional regulator
wy_00840	*tap*	852896	854212	+	1317	Tripeptidyl aminopeptidase precursor
wy_00841	-	854354	854878	+	525	hypothetical protein
wy_00842	-	854952	855980	+	1029	hypothetical protein
wy_00843	-	856060	856758	+	699	Methyltransferase
wy_00844	*betC*	856772	858448	-	1677	Choline-sulfatase
wy_00845	*mmsA*	858595	860169	+	1575	Methylmalonate-semialdehyde dehydrogenase [acylating]
wy_00846	*mmgC_1*	860174	861319	+	1146	Acyl-CoA dehydrogenase
wy_00847	*acdA_3*	861372	862520	+	1149	Acyl-CoA dehydrogenase
wy_00848	*echA8_4*	862517	863584	+	1068	putative enoyl-CoA hydratase echA8
wy_00849	*mmsB_1*	863581	864492	+	912	3-hydroxyisobutyrate dehydrogenase
wy_00850	*echA8_5*	864489	865265	+	777	putative enoyl-CoA hydratase echA8
wy_00851	*hsaD_1*	865328	866224	-	897	4,5:9,10-diseco-3-hydroxy-5,9, 17-trioxoandrosta-1(10),2-diene-4-oate hydrolase
wy_00852	*slyA*	866419	866871	+	453	Transcriptional regulator SlyA
wy_0085	*yusO_1*	866920	867456	-	537	putative HTH-type transcriptional regulator

3						YusO
wy_00854	*pbuE*	867567	868793	+	1227	Purine efflux pump PbuE
wy_00855	*bcr_1*	868797	869945	-	1149	Bicyclomycin resistance protein
wy_00856	-	870390	871793	-	1404	hypothetical protein
wy_00857	-	871911	873059	-	1149	Sulfotransferase domain protein
wy_00858	*hdhA_2*	873083	873865	-	783	7-alpha-hydroxysteroid dehydrogenase
wy_00859	-	873869	875035	-	1167	hypothetical protein
wy_00860	-	875129	876064	+	936	IclR helix-turn-helix domain protein
wy_00861	*cysD_1*	876185	877111	+	927	Sulfate adenylyltransferase subunit 2
wy_00862	*cysNC_1*	877111	878964	+	1854	Bifunctional enzyme CysN/CysC
wy_00863	*cysQ_1*	878961	880076	+	1116	3'-phosphoadenosine 5'-phosphate phosphatase
wy_00864	-	880082	880555	-	474	hypothetical protein
wy_00865	-	880552	880905	-	354	HTH-type transcriptional regulator
wy_00866	*embC_1*	880966	884256	-	3291	putative arabinosyltransferase C
wy_00867	*fgd1_1*	884360	885385	+	1026	F420-dependent glucose-6-phosphate dehydrogenase
wy_00868	-	885411	886673	+	1263	hypothetical protein
wy_00870	-	887001	887726	-	726	hypothetical protein
wy_00871	-	887735	888646	-	912	hypothetical protein
wy_00872	*argS*	888815	890467	+	1653	Arginine--tRNA ligase
wy_00873	*lysA_1*	890464	891885	+	1422	Diaminopimelate decarboxylase
wy_00874	*hom*	891882	893195	+	1314	Homoserine dehydrogenase
wy_00875	*thrC*	893192	894295	+	1104	Threonine synthase
wy_00876	*thrB*	894295	895263	+	969	Homoserine kinase
wy_00877	-	895592	897622	+	2031	hypothetical protein
wy_00878	*rpmE*	897831	898064	+	234	50S ribosomal protein L31
wy_00879	*prfA*	898211	899290	+	1080	Peptide chain release factor 1
wy_0088	-	899303	899707	-	405	hypothetical protein

0						
wy_00881	*prmC_1*	899850	900743	+	894	Release factor glutamine methyltransferase
wy_00882	*ywlC*	900777	901436	+	660	Threonylcarbamoyl-AMP synthase
wy_00883	*wecA*	901521	902681	+	1161	Decaprenyl-phosphate N-acetylglucosaminephosphotransferase
wy_00884	-	902732	903187	+	456	hypothetical protein
wy_00885	*atpB*	903576	904355	+	780	ATP synthase subunit a
wy_00886	*atpE*	904426	904677	+	252	ATP synthase subunit c
wy_00887	*atpF*	904687	905247	+	561	ATP synthase subunit b
wy_00888	*atpH*	905253	906077	+	825	ATP synthase subunit delta
wy_00889	*atpA*	906139	907782	+	1644	ATP synthase subunit alpha
wy_00890	*atpG*	907831	908811	+	981	ATP synthase gamma chain
wy_00891	*atpD*	908815	910263	+	1449	ATP synthase subunit beta
wy_00892	*atpC*	910270	910638	+	369	ATP synthase epsilon chain
wy_00893	-	910675	911109	+	435	hypothetical protein
wy_00894	-	911174	911749	-	576	Cob(I)yrinic acid a,c-diamide adenosyltransferase
wy_00895	*murA*	911859	913076	+	1218	UDP-N-acetylglucosamine 1-carboxyvinyltransferase
wy_00899	*cyaB_1*	918944	920542	-	1599	Adenylate cyclase 2
wy_00900	-	920650	921276	+	627	hypothetical protein
wy_00901	-	921701	922639	-	939	hypothetical protein
wy_00902	-	922739	923218	-	480	Glyoxalase/Bleomycin resistance protein/Dioxygenase superfamily protein
wy_00903	-	923309	924502	+	1194	putative acetyl-CoA acetyltransferase
wy_00904	-	924586	924957	+	372	hypothetical protein
wy_00905	*trxA_1*	925065	925973	+	909	Thioredoxin
wy_00906	-	925996	927441	-	1446	hypothetical protein
wy_00907	*glgB*	927491	929686	-	2196	1,4-alpha-glucan branching enzyme GlgB
wy_00908	*glgE*	929683	931692	-	2010	Alpha-1,4-glucan:maltose-1-phosphate maltosyltransferase

wy_00909	*malP*	932130	934748	+	2619	Maltodextrin phosphorylase
wy_00910	*davT*	934781	936133	-	1353	5-aminovalerate aminotransferase DavT
wy_00911	-	936149	937798	-	1650	ABC-2 family transporter protein
wy_00912	-	937795	938121	-	327	hypothetical protein
wy_00913	*ybhF_2*	938193	938717	-	525	putative ABC transporter ATP-binding protein YbhF
wy_00914	-	938797	940854	-	2058	hypothetical protein
wy_00915	-	940906	942315	-	1410	Putative cytochrome P450 120
wy_00916	-	942383	943003	+	621	transcriptional regulator BetI
wy_00917	*nicF*	943017	943604	-	588	Maleamate amidohydrolase
wy_00918	*pncB1*	943601	944968	-	1368	Nicotinate phosphoribosyltransferase pncB1
wy_00919	-	945057	945395	+	339	ATP-dependent Clp protease adaptor protein ClpS
wy_00920	-	945498	946025	+	528	hypothetical protein
wy_00921	-	946022	947110	+	1089	Peptidase family S58
wy_00922	-	947145	947825	+	681	Rhomboid family protein
wy_00923	*rbn*	947949	948707	+	759	Ribonuclease BN
wy_00924	*rph*	948728	949510	+	783	Ribonuclease PH
wy_00925	-	949507	950118	+	612	Non-canonical purine NTP pyrophosphatase
wy_00926	-	950198	950578	-	381	hypothetical protein
wy_00927	-	950575	950943	-	369	hypothetical protein
wy_00928	*rpsP*	951208	951699	+	492	30S ribosomal protein S16
wy_00929	-	951699	951941	+	243	hypothetical protein
wy_00930	*rimM*	951948	952514	+	567	Ribosome maturation factor RimM
wy_00931	*trmD*	952525	953223	+	699	tRNA (guanine-N(1)-)-methyltransferase
wy_00932	*puo_1*	953302	954735	+	1434	Putrescine oxidase
wy_00933	-	954689	955627	-	939	hypothetical protein
wy_00934	-	955704	958028	+	2325	hypothetical protein

wy_00935	-	958073	958744	-	672	hypothetical protein
wy_00936	*pkn1*	958855	959829	+	975	Serine/threonine-protein kinase pkn1
wy_00937	-	959829	960713	+	885	hypothetical protein
wy_00938	*rplS*	960787	961128	+	342	50S ribosomal protein L19
wy_00939	*lepB*	961226	961999	+	774	Signal peptidase I
wy_00940	*rnhB*	962050	962688	+	639	Ribonuclease HII
wy_00941	-	962737	963054	+	318	hypothetical protein
wy_00942	-	963268	963624	+	357	hypothetical protein
wy_00943	*comM*	963624	965132	+	1509	Competence protein ComM
wy_00944	-	965129	966250	+	1122	hypothetical protein
wy_00945	-	966262	966795	-	534	hypothetical protein
wy_00946	-	966947	968080	+	1134	hypothetical protein
wy_00947	*viuB_1*	968156	968935	+	780	Vibriobactin utilization protein ViuB
wy_00948	*bcr_2*	968955	970106	-	1152	Bicyclomycin resistance protein
wy_00949	*xerC*	970192	971133	+	942	Tyrosine recombinase XerC
wy_00950	-	971118	971711	-	594	Peptidase family M23
wy_00951	*rpsB*	972042	972872	+	831	30S ribosomal protein S2
wy_00952	*tsf*	972987	973811	+	825	Elongation factor Ts
wy_00953	*pyrH*	974082	974810	+	729	Uridylate kinase
wy_00954	*frr*	974878	975435	+	558	Ribosome-recycling factor
wy_00955	*cdsA*	975438	976298	+	861	Phosphatidate cytidylyltransferase
wy_00956	-	976295	977029	+	735	putative S-adenosylmethionine-dependent methyltransferase/MSMEI_2290
wy_00957	-	977090	978379	+	1290	putative inactive lipase/MT1628
wy_00958	*citE_1*	978383	979225	-	843	Citrate lyase subunit beta-like protein
wy_00959	-	979297	979695	-	399	hypothetical protein
wy_00960	*rlmN*	979775	980884	+	1110	Dual-specificity RNA methyltransferase RlmN

wy_00961	-	980953	981222	-	270	hypothetical protein
wy_00962	*dxr*	981384	982541	+	1158	1-deoxy-D-xylulose 5-phosphate reductoisomerase
wy_00963	*rip1*	982548	983780	+	1233	Zinc metalloprotease Rip1
wy_00964	*ispG*	983842	985005	+	1164	4-hydroxy-3-methylbut-2-en-1-yl diphosphate synthase
wy_00965	*mshD_1*	985120	985956	+	837	Mycothiol acetyltransferase
wy_00966	*comC*	985963	986751	-	789	Type 4 prepilin-like proteins leader peptide-processing enzyme
wy_00967	-	987078	987284	+	207	Flp/Fap pilin component
wy_00968	-	987366	987767	+	402	TadE-like protein
wy_00969	-	987776	988720	+	945	hypothetical protein
wy_00970	-	988730	989467	+	738	hypothetical protein
wy_00971	*minD*	989464	990663	+	1200	Septum site-determining protein MinD
wy_00972	-	990660	992057	+	1398	Putative conjugal transfer protein/MT3759
wy_00973	-	992054	993925	+	1872	Bacterial type II secretion system protein F domain protein
wy_00974	-	993948	994838	+	891	Bacterial type II secretion system protein F domain protein
wy_00975	-	994851	995651	-	801	hypothetical protein
wy_00976	-	995731	996153	+	423	Interferon-induced transmembrane protein
wy_00977	*vjbR*	996173	997093	-	921	HTH-type quorum sensing-dependent transcriptional regulator VjbR
wy_00978	*acsA_2*	997146	998765	+	1620	Acetyl-coenzyme A synthetase
wy_00979	*phnV*	998828	999616	-	789	Putative 2-aminoethylphosphonate transport system permease protein PhnV
wy_00980	*phnU*	999591	1000496	-	906	Putative 2-aminoethylphosphonate transport system permease protein PhnU
wy_00981	*phnT*	1000493	1001698	-	1206	Putative 2-aminoethylphosphonate import ATP-binding protein PhnT
wy_00982	*phnS*	1001700	1002761	-	1062	Putative 2-aminoethylphosphonate-binding periplasmic protein precursor
wy_00983	*phnX*	1002820	1003497	-	678	Phosphonoacetaldehyde hydrolase
wy_00984	-	1003522	1004667	-	1146	D-amino acid dehydrogenase small subunit
wy_00985	-	1004749	1005279	+	531	hypothetical protein

wy_00986	*phnF*	1005276	1005977	+	702	HTH-type transcriptional repressor PhnF
wy_00987	-	1005978	1006235	-	258	hypothetical protein
wy_00988	-	1006409	1008106	+	1698	putative sulfate transporter/MT1781
wy_00989	*dagK_1*	1008116	1009432	+	1317	Diacylglycerol kinase
wy_00990	-	1009439	1009756	-	318	hypothetical protein
wy_00991	*map_1*	1009910	1010767	+	858	Methionine aminopeptidase 2
wy_00992	*cobQ*	1010764	1012296	+	1533	Cobyric acid synthase
wy_00993	-	1012427	1013638	+	1212	D-alanyl-D-alanine carboxypeptidase precursor
wy_00994	-	1013680	1014246	+	567	hypothetical protein
wy_00995	*dauA*	1014290	1015774	+	1485	C4-dicarboxylic acid transporter DauA
wy_00996	*cefF*	1016126	1017124	+	999	Deacetoxycephalosporin C hydroxylase
wy_00997	*metQ*	1017156	1017989	+	834	Methionine-binding lipoprotein MetQ precursor
wy_00998	*metN*	1017989	1019008	+	1020	Methionine import ATP-binding protein MetN
wy_00999	*metP*	1019005	1019673	+	669	Methionine import system permease protein MetP
wy_01000	-	1019776	1020204	+	429	hypothetical protein
wy_01001	*mtr*	1020184	1021560	-	1377	Mycothione reductase
wy_01002	-	1021557	1022579	-	1023	Alpha/beta hydrolase family protein
wy_01003	*mqo*	1022775	1024292	+	1518	putative malate:quinone oxidoreductase
wy_01004	*bchI_2*	1024534	1026435	+	1902	Magnesium-chelatase 38 kDa subunit
wy_01005	*btuR*	1026479	1027093	+	615	Cob(I)yrinic acid a,c-diamide adenosyltransferase
wy_01006	*cobB_2*	1027087	1028469	+	1383	Cobyrinic acid A,C-diamide synthase
wy_01007	-	1028462	1028824	+	363	cobalamin biosynthesis protein CbiG
wy_01008	*cysG*	1028871	1030097	+	1227	Siroheme synthase
wy_01009	*efpA_2*	1030109	1031590	+	1482	putative MFS-type transporter EfpA
wy_01010	-	1031665	1032531	+	867	hypothetical protein
wy_01011	-	1032524	1033267	-	744	hypothetical protein

wy_01012	-	1033358	1034698	-	1341	hypothetical protein
wy_01013	-	1034883	1036958	-	2076	IPT/TIG domain protein
wy_01014	*proS_1*	1037189	1038922	+	1734	Proline--tRNA ligase
wy_01015	*sadH_3*	1038973	1039818	-	846	Putative oxidoreductase SadH
wy_01016	*lcfB_7*	1039815	1041437	-	1623	Long-chain-fatty-acid--CoA ligase
wy_01017	*gabD2_1*	1041449	1043026	-	1578	Putative succinate-semialdehyde dehydrogenase [NADP(+)] 2
wy_01018	*rutR_2*	1043070	1043606	-	537	HTH-type transcriptional regulator RutR
wy_01019	-	1043688	1045043	-	1356	Putative cytochrome P450 120
wy_01020	-	1045071	1046003	-	933	hypothetical protein
wy_01021	-	1046187	1047920	+	1734	Acyl-CoA dehydrogenase, short-chain specific
wy_01022	-	1048036	1048656	+	621	hypothetical protein
wy_01023	-	1048672	1049217	-	546	hypothetical protein
wy_01024	-	1049317	1049730	+	414	putative response regulatory protein/MT3230
wy_01025	*ndhC*	1049924	1050292	+	369	NAD(P)H-quinone oxidoreductase subunit 3
wy_01026	*nqo6*	1050332	1050904	+	573	NADH-quinone oxidoreductase subunit 6
wy_01027	*nuoC1*	1050904	1051656	+	753	NADH-quinone oxidoreductase subunit C 1
wy_01028	*nqo4*	1051653	1052987	+	1335	NADH-quinone oxidoreductase subunit 4
wy_01029	*nqo1*	1052984	1055050	+	2067	NADH-quinone oxidoreductase subunit 1
wy_01030	*nqo3*	1055047	1057500	+	2454	NADH-quinone oxidoreductase subunit 3
wy_01031	*nqo8*	1057497	1058801	+	1305	NADH-quinone oxidoreductase subunit 8
wy_01032	*nqo9*	1058794	1059339	+	546	NADH-quinone oxidoreductase subunit 9
wy_01033	-	1059339	1060169	+	831	NADH-quinone oxidoreductase subunit 10
wy_01034	-	1060166	1060465	+	300	NADH-quinone oxidoreductase subunit 11
wy_01035	-	1060467	1062398	+	1932	NADH-quinone oxidoreductase subunit 12
wy_01036	*nuoM*	1062395	1063975	+	1581	NADH-quinone oxidoreductase subunit M
wy_01037	*nuoN*	1063972	1065558	+	1587	NADH-quinone oxidoreductase subunit N

wy_01038	-	1065630	1065785	-	156	hypothetical protein
wy_01039	-	1065990	1066601	-	612	DNA-binding transcriptional repressor AcrR
wy_01040	*qacA_1*	1066734	1068269	+	1536	Antiseptic resistance protein
wy_01041	-	1068203	1068691	-	489	hypothetical protein
wy_01042	-	1068794	1069240	-	447	hypothetical protein
wy_01043	-	1069237	1069731	-	495	hypothetical protein
wy_01044	*rimP*	1069958	1070527	+	570	Ribosome maturation factor RimP
wy_01045	-	1070524	1071555	+	1032	hypothetical protein
wy_01046	*infB*	1072143	1075001	+	2859	Translation initiation factor IF-2
wy_01047	-	1075101	1075391	+	291	hypothetical protein
wy_01048	*rbfA*	1075445	1075909	+	465	Ribosome-binding factor A
wy_01049	*nrnA*	1075922	1076923	+	1002	Bifunctional oligoribonuclease and PAP phosphatase NrnA
wy_01050	*dinF*	1076925	1078295	+	1371	DNA-damage-inducible protein F
wy_01051	-	1078315	1079223	+	909	Calcineurin-like phosphoesterase
wy_01052	*npt*	1079237	1079908	+	672	4'-phosphopantetheinyl transferase Npt
wy_01053	*truB*	1079934	1080860	+	927	tRNA pseudouridine synthase B
wy_01054	-	1080868	1081278	-	411	hypothetical protein
wy_01055	*ideR_1*	1081335	1081985	-	651	Iron-dependent repressor IdeR
wy_01056	*ribF*	1082221	1083165	+	945	Riboflavin biosynthesis protein RibF
wy_01057	*rpsO*	1083298	1083567	+	270	30S ribosomal protein S15
wy_01058	*pnp*	1083846	1086104	+	2259	Polyribonucleotide nucleotidyltransferase
wy_01059	*ptrA*	1086281	1087621	+	1341	Protease 3 precursor
wy_01060	*atsA_2*	1087631	1090015	-	2385	Arylsulfatase
wy_01061	-	1090263	1091135	+	873	Putative S-adenosyl-L-methionine-dependent methyltransferase
wy_01062	*dapB*	1091292	1091987	+	696	4-hydroxy-tetrahydrodipicolinate reductase
wy_01063	-	1091984	1092451	+	468	hypothetical protein

wy_01064	*fldA_2*	1092455	1092931	+	477	Flavodoxin
wy_01065	-	1092934	1093590	-	657	hypothetical protein
wy_01066	-	1093602	1094036	-	435	Polyketide cyclase / dehydrase and lipid transport
wy_01067	-	1094141	1094641	-	501	hypothetical protein
wy_01068	*bdhA_1*	1094710	1095504	-	795	D-beta-hydroxybutyrate dehydrogenase
wy_01069	*thyX*	1095684	1096436	+	753	Thymidylate synthase ThyX
wy_01070	*dapA_1*	1096503	1097411	+	909	4-hydroxy-tetrahydrodipicolinate synthase
wy_01071	-	1097408	1099327	+	1920	Putative ribonuclease J
wy_01072	-	1099375	1099962	+	588	hypothetical protein
wy_01073	-	1099967	1100398	-	432	Pyridoxamine 5'-phosphate oxidase
wy_01074	-	1100506	1101129	+	624	hypothetical protein
wy_01075	*spoIIIE*	1101508	1103943	+	2436	DNA translocase SpoIIIE
wy_01076	*alx*	1104155	1105153	+	999	Inner membrane protein alx
wy_01077	-	1105203	1105487	-	285	YciI-like protein
wy_01078	*argA*	1105517	1106005	-	489	Amino-acid acetyltransferase
wy_01079	*pgsA2*	1106174	1106809	+	636	Putative CDP-diacylglycerol--glycerol-3-phosphate 3-phosphatidyl-transferase 2
wy_01080	*cinA_1*	1106806	1107294	+	489	Putative competence-damage inducible protein
wy_01081	*clgR*	1107368	1107718	+	351	Transcriptional regulator ClgR
wy_01082	-	1107890	1108714	+	825	PspA/IM30 family protein
wy_01083	-	1108865	1109617	+	753	hypothetical protein
wy_01084	*bioN*	1109630	1110235	-	606	Energy-coupling factor transporter transmembrane protein BioN
wy_01085	*bioM*	1110232	1110912	-	681	Biotin transport ATP-binding protein BioM
wy_01086	*bioY*	1110928	1111539	-	612	Biotin transporter BioY
wy_01087	-	1111766	1111942	+	177	hypothetical protein
wy_01088	*gltC_1*	1111939	1112823	-	885	HTH-type transcriptional regulator GltC
wy_01089	*ynfM_1*	1112972	1114129	+	1158	Inner membrane transport protein YnfM

wy_01090	*mca_2*	1114134	1114847	+	714	Mycothiol S-conjugate amidase
wy_01091	*dagK_2*	1114851	1115741	-	891	Diacylglycerol kinase
wy_01092	-	1115738	1117366	-	1629	putative FAD-linked oxidoreductase
wy_01093	-	1117387	1117974	+	588	Bacterial regulatory proteins, tetR family
wy_01094	*glpD_1*	1118012	1119523	+	1512	Aerobic glycerol-3-phosphate dehydrogenase
wy_01095	*rutA_1*	1119739	1120722	+	984	Pyrimidine monooxygenase RutA
wy_01096	*rebG*	1120758	1121906	+	1149	4'-demethylrebeccamycin synthase
wy_01097	-	1121947	1122141	+	195	hypothetical protein
wy_01098	-	1122336	1123373	+	1038	DNA recombination protein RecA
wy_01099	*recX*	1123433	1124122	+	690	Regulatory protein RecX
wy_01100	-	1124140	1124412	-	273	hypothetical protein
wy_01101	*artQ_1*	1124802	1125725	-	924	Arginine transport system permease protein ArtQ
wy_01102	*glnM*	1125722	1126405	-	684	putative glutamine ABC transporter permease protein GlnM
wy_01103	*glnH_2*	1126421	1127266	-	846	ABC transporter glutamine-binding protein GlnH precursor
wy_01104	*glnQ_1*	1127284	1128057	-	774	Glutamine transport ATP-binding protein GlnQ
wy_01105	*miaB*	1128224	1129741	+	1518	(Dimethylallyl)adenosine tRNA methylthiotransferase MiaB
wy_01106	-	1129738	1130340	+	603	hypothetical protein
wy_01107	-	1130415	1131773	-	1359	hypothetical protein
wy_01108	-	1131939	1132679	+	741	hypothetical protein
wy_01109	*miaA*	1132676	1133602	+	927	tRNA dimethylallyltransferase
wy_01110	*dapF*	1133624	1134493	+	870	Diaminopimelate epimerase
wy_01111	*hflX*	1134574	1136052	+	1479	GTPase HflX
wy_01112	-	1136156	1137184	+	1029	hypothetical protein
wy_01113	-	1137366	1138982	+	1617	hypothetical protein
wy_01114	*rutG_1*	1139194	1140450	+	1257	Putative pyrimidine permease RutG
wy_0111	*mmgC_2*	114061	114176	+	1146	Acyl-CoA dehydrogenase

5		5	0			
wy_01116	*lexA_1*	1141824	1142552	-	729	LexA repressor
wy_01117	-	1142811	1143395	+	585	hypothetical protein
wy_01118	*nrdR*	1143637	1144125	+	489	Transcriptional repressor NrdR
wy_01119	*hrpB*	1144167	1148108	-	3942	ATP-dependent RNA helicase HrpB
wy_01120	-	1148253	1149479	+	1227	Omega-amino acid--pyruvate aminotransferase
wy_01121	*rbsR*	1149496	1150494	-	999	Ribose operon repressor
wy_01122	*rbsK_1*	1150679	1151653	+	975	Ribokinase
wy_01123	*mglA*	1151650	1153143	+	1494	Galactose/methyl galactoside import ATP-binding protein MglA
wy_01124	*rbsB*	1153149	1155134	+	1986	D-ribose-binding periplasmic protein precursor
wy_01125	-	1155138	1155389	-	252	hypothetical protein
wy_01126	-	1155407	1156111	+	705	hypothetical protein
wy_01127	*rpfE_1*	1156458	1157045	+	588	Resuscitation-promoting factor RpfE precursor
wy_01128	-	1157194	1157496	+	303	hypothetical protein
wy_01129	*oxyR_1*	1157569	1158498	+	930	putative hydrogen peroxide-inducible genes activator
wy_01130	*hcaR_1*	1158514	1159194	-	681	Hca operon transcriptional activator
wy_01131	-	1159358	1159789	+	432	hypothetical protein
wy_01132	-	1159829	1160128	-	300	hypothetical protein
wy_01133	-	1160216	1160866	-	651	hypothetical protein
wy_01134	-	1160940	1163459	-	2520	ski2-like helicase
wy_01135	-	1163532	1164569	-	1038	Soluble epoxide hydrolase
wy_01136	-	1164751	1165704	-	954	PAC2 family protein
wy_01137	-	1165917	1166612	+	696	hypothetical protein
wy_01138	-	1166827	1167876	+	1050	hypothetical protein
wy_01139	*galE_1*	1167873	1168868	-	996	UDP-glucose 4-epimerase
wy_01140	*ideR_2*	1168914	1169600	-	687	Iron-dependent repressor IdeR
wy_0114	*acuC*	116983	117105	+	1218	Acetoin utilization protein AcuC

1		4	1			
wy_0114 2	-	117104 4	117374 0	+	2697	Acetyltransferase Pat
wy_0114 3	*sigB*	117384 0	117480 8	-	969	RNA polymerase sigma factor SigB
wy_0114 4	*rsmC*	117496 0	117643 8	-	1479	Ribosomal RNA small subunit methyltransferase C
wy_0114 5	-	117648 6	117684 2	-	357	hypothetical protein
wy_0114 6	-	117691 1	117713 8	+	228	hypothetical protein
wy_0114 7	-	117711 1	117822 0	-	1110	hypothetical protein
wy_0114 8	-	117848 3	118021 9	+	1737	Type III restriction enzyme, res subunit
wy_0114 9	-	118030 8	118051 1	-	204	hypothetical protein
wy_0115 0	-	118075 8	118112 6	+	369	hypothetical protein
wy_0115 1	*khtT*	118119 3	118167 5	+	483	K(+)/H(+) antiporter subunit KhtT
wy_0115 2	*yhaU*	118169 7	118290 2	+	1206	K(+)/H(+) antiporter YhaU
wy_0115 3	*sigA*	118291 8	118429 7	-	1380	RNA polymerase sigma factor SigA
wy_0115 4	*ppgK*	118442 1	118519 7	-	777	Polyphosphate glucokinase
wy_0115 5	*suhB*	118532 4	118626 5	+	942	Inositol-1-monophosphatase SuhB
wy_0115 6	-	118627 5	118695 5	-	681	hypothetical protein
wy_0115 7	-	118743 7	118773 9	+	303	hypothetical protein
wy_0115 8	-	118777 5	118821 2	-	438	hypothetical protein
wy_0115 9	*dut*	118830 3	118884 5	+	543	Deoxyuridine 5'-triphosphate nucleotidohydrolase
wy_0116 0	-	118884 8	118966 3	+	816	hypothetical protein
wy_0116 1	-	118967 1	119039 3	-	723	Alpha/beta hydrolase family protein
wy_0116 2	-	119059 5	119097 2	+	378	hypothetical protein
wy_0116 3	-	119102 7	119175 2	+	726	hypothetical protein
wy_0116 4	*trkA_1*	119172 9	119238 5	-	657	Trk system potassium uptake protein TrkA
wy_0116 5	*trkA_2*	119243 2	119309 7	-	666	Trk system potassium uptake protein TrkA
wy_0116 6	-	119331 0	119530 4	+	1995	hypothetical protein
wy_0116	-	119525	119658	+	1326	putative RNA methyltransferase/cg2084

7		8	3			
wy_01168	-	1196742	1197224	+	483	transcriptional regulator SlyA
wy_01169	*bmr3_5*	1197221	1198819	+	1599	Multidrug resistance protein 3
wy_01170	*drrB_2*	1198925	1199755	-	831	Daunorubicin/doxorubicin resistance ABC transporter permease protein DrrB
wy_01171	*drrA_4*	1199752	1200741	-	990	Daunorubicin/doxorubicin resistance ATP-binding protein DrrA
wy_01172	*glsA1*	1200910	1202733	-	1824	Glutaminase 1
wy_01173	*dxs*	1202873	1204843	+	1971	1-deoxy-D-xylulose-5-phosphate synthase
wy_01174	*ohyA*	1204850	1206445	-	1596	Oleate hydratase
wy_01175	*spkB*	1206671	1207486	+	816	Serine/threonine-protein kinase B
wy_01176	*xylE*	1207489	1208925	-	1437	D-xylose-proton symporter
wy_01177	*yfiY_1*	1209020	1210036	-	1017	putative siderophore-binding lipoprotein YfiY precursor
wy_01178	*fadJ_1*	1210138	1212243	-	2106	Fatty acid oxidation complex subunit alpha
wy_01179	*paaJ_1*	1212244	1213470	-	1227	3-oxoadipyl-CoA/3-oxo-5,6-dehydrosuberyl-CoA thiolase
wy_01180	*rnd*	1213592	1214833	-	1242	Ribonuclease D
wy_01181	-	1215065	1215916	+	852	short chain dehydrogenase
wy_01182	-	1215861	1217270	-	1410	Putative diacylglycerol O-acyltransferase/MT1809
wy_01183	*nlhH_4*	1217350	1218618	+	1269	Carboxylesterase NlhH
wy_01184	-	1218615	1219214	-	600	hypothetical protein
wy_01185	*hemE*	1219303	1220400	+	1098	Uroporphyrinogen decarboxylase
wy_01186	*hemY*	1220397	1221761	+	1365	Protoporphyrinogen oxidase
wy_01187	*cld*	1221766	1222461	+	696	Chlorite dismutase precursor
wy_01188	*dorA*	1222614	1224884	+	2271	Dimethyl sulfoxide/trimethylamine N-oxide reductase precursor
wy_01189	*ytrA_2*	1224921	1225271	+	351	HTH-type transcriptional repressor YtrA
wy_01190	-	1225268	1226272	+	1005	hypothetical protein
wy_01191	*msrB*	1226344	1226784	-	441	Peptide methionine sulfoxide reductase MsrB
wy_01192	-	1226816	1228006	-	1191	Alpha-(1->3)-arabinofuranosyltransferase

wy_01193	*caeA_1*	1228122	1229663	-	1542	Carboxylesterase A precursor
wy_01194	*ribD2*	1229693	1230481	-	789	2,5-diamino-6-ribosylamino-4(3H)-pyrimidinone 5'-phosphate reductase
wy_01195	-	1230564	1231517	+	954	AFG1-like ATPase
wy_01196	*paiA*	1231546	1232076	-	531	Protease synthase and sporulation negative regulatory protein PAI 1
wy_01197	*ybjJ_2*	1232140	1233342	-	1203	Inner membrane protein YbjJ
wy_01198	*bglA_1*	1233350	1234786	+	1437	Beta-glucosidase
wy_01205	-	1236103	1236870	-	768	26 kDa periplasmic immunogenic protein precursor
wy_01206	-	1236974	1237453	+	480	Putative transmembrane protein (PGPGW)
wy_01207	*thrS*	1237564	1239615	+	2052	Threonine--tRNA ligase
wy_01208	-	1239612	1240172	+	561	AP-4-A phosphorylase
wy_01209	*pgsA1*	1240267	1240953	+	687	CDP-diacylglycerol--inositol 3-phosphatidyltransferase
wy_01210	-	1240950	1241855	+	906	Phosphatidylinositol mannoside acyltransferase
wy_01211	*pimA*	1241852	1242979	+	1128	GDP-mannose-dependent alpha-(1-2)-phosphatidylinositol mannosyltransferase
wy_01212	-	1242976	1243992	+	1017	dihydroneopterin triphosphate pyrophosphatase
wy_01213	*pdxS_2*	1244183	1245085	+	903	Pyridoxal biosynthesis lyase PdxS
wy_01214	*tesB_1*	1245196	1246023	+	828	Acyl-CoA thioesterase 2
wy_01215	*pdxT*	1246115	1246720	+	606	Glutamine amidotransferase subunit PdxT
wy_01216	*fbpC_3*	1246751	1247785	-	1035	Diacylglycerol acyltransferase/mycolyltransferase Ag85C precursor
wy_01217	-	1248081	1248833	+	753	putative transcriptional regulatory protein/MSMEI_2866
wy_01218	*ruvC*	1248931	1249506	+	576	Crossover junction endodeoxyribonuclease RuvC
wy_01219	*ruvA*	1249503	1250108	+	606	Holliday junction ATP-dependent DNA helicase RuvA
wy_01220	*ruvB*	1250105	1251238	+	1134	Holliday junction ATP-dependent DNA helicase RuvB
wy_01221	-	1251417	1251758	+	342	preprotein translocase subunit YajC
wy_01222	-	1251970	1253583	+	1614	preprotein translocase subunit SecD
wy_01223	-	1253586	1254761	+	1176	preprotein translocase subunit SecF

wy_01224	-	1254763	1256502	+	1740	putative monoacyl phosphatidylinositol tetramannoside-binding protein LpqW precursor
wy_01225	*apt*	1256469	1257077	+	609	Adenine phosphoribosyltransferase
wy_01226	*relA*	1257159	1259495	+	2337	Bifunctional (p)ppGpp synthase/hydrolase relA
wy_01227	*cypB_1*	1259573	1260361	-	789	Peptidyl-prolyl cis-trans isomerase B
wy_01228	*cypB_2*	1260361	1261221	-	861	Peptidyl-prolyl cis-trans isomerase B
wy_01229	-	1261418	1262098	+	681	putative metallo-hydrolase
wy_01230	*hisS*	1262114	1263394	+	1281	Histidine--tRNA ligase
wy_01231	-	1263441	1264022	+	582	hypothetical protein
wy_01232	-	1264128	1266731	+	2604	transcriptional regulator MalT
wy_01233	*mdh*	1266838	1268010	+	1173	Malate dehydrogenase
wy_01234	-	1267875	1269221	-	1347	hypothetical protein
wy_01235	-	1269218	1269547	-	330	hypothetical protein
wy_01236	*kstR2_6*	1269611	1270318	-	708	HTH-type transcriptional repressor KstR2
wy_01237	*yhdG_1*	1270562	1272097	+	1536	putative amino acid permease YhdG
wy_01238	-	1272099	1272977	+	879	hypothetical protein
wy_01239	-	1272974	1273639	+	666	hypothetical protein
wy_01240	*kipA_1*	1273636	1275621	+	1986	KipI antagonist
wy_01241	*stp_1*	1275770	1277254	+	1485	Multidrug resistance protein stp
wy_01242	*ttgR_2*	1277251	1277826	+	576	HTH-type transcriptional regulator TtgR
wy_01243	-	1277887	1279128	-	1242	hypothetical protein
wy_01244	-	1279609	1279926	-	318	SMP-30/Gluconolaconase/LRE-like region
wy_01245	-	1280326	1281168	-	843	hypothetical protein
wy_01246	*copA_2*	1281216	1282757	-	1542	Copper resistance protein A precursor
wy_01247	*htpX_1*	1282888	1283835	-	948	Protease HtpX
wy_01248	*blaI_1*	1283832	1284209	-	378	Transcriptional regulator BlaI
wy_0124	*nlpD*	128461	128525	+	645	Murein hydrolase activator NlpD precursor

9		0	4			
wy_01250	*yabJ_1*	1285340	1285801	-	462	Enamine/imine deaminase
wy_01251	-	1285820	1286845	+	1026	Radical SAM superfamily protein
wy_01252	*pcaF_1*	1286867	1288018	-	1152	Beta-ketoadipyl-CoA thiolase
wy_01253	-	1288178	1289341	+	1164	pheromone autoinducer 2 transporter
wy_01254	*lcfB_8*	1289345	1290868	-	1524	Long-chain-fatty-acid--CoA ligase
wy_01255	-	1290927	1291856	-	930	Putative neutral zinc metallopeptidase
wy_01256	*aspS*	1292024	1293805	+	1782	Aspartate--tRNA ligase
wy_01257	-	1293830	1294795	+	966	hypothetical protein
wy_01258	-	1294844	1296022	+	1179	hypothetical protein
wy_01259	-	1296034	1296789	-	756	Bacterial regulatory proteins, tetR family
wy_01260	*mca_3*	1296944	1297624	+	681	Mycothiol S-conjugate amidase
wy_01261	-	1297723	1297881	+	159	hypothetical protein
wy_01262	*gsiA_1*	1297937	1299571	-	1635	Glutathione import ATP-binding protein GsiA
wy_01263	*gsiD_1*	1299593	1300459	-	867	Glutathione transport system permease protein GsiD
wy_01264	*gsiC_1*	1300456	1301415	-	960	Glutathione transport system permease protein GsiC
wy_01265	*hbpA*	1301422	1302996	-	1575	Heme-binding protein A precursor
wy_01266	*atsA_3*	1303185	1305515	-	2331	Arylsulfatase
wy_01267	-	1305737	1306702	-	966	putative dioxygenase/MT3514
wy_01268	*tauD_3*	1306746	1307552	-	807	Alpha-ketoglutarate-dependent taurine dioxygenase
wy_01269	-	1307747	1308400	+	654	putative DNA-binding transcriptional regulator
wy_01270	*rarA*	1308446	1309852	+	1407	Replication-associated recombination protein A
wy_01271	-	1309786	1309923	-	138	hypothetical protein
wy_01272	*alaS_1*	1310004	1310213	+	210	Alanine--tRNA ligase
wy_01273	*alaS_2*	1310468	1312678	+	2211	Alanine--tRNA ligase
wy_01274	*yrrK*	1312681	1313241	+	561	Putative Holliday junction resolvase
wy_0127	-	131323	131468	+	1452	putative aminodeoxychorismate lyase

5		8	9			
wy_01276	*aroE_1*	1314695	1315519	+	825	Shikimate dehydrogenase
wy_01277	*aroC*	1315622	1316818	+	1197	Chorismate synthase
wy_01278	*aroK*	1316823	1317377	+	555	Shikimate kinase
wy_01279	*aroB*	1317382	1318491	+	1110	3-dehydroquinate synthase
wy_01280	*aroQ*	1318488	1318916	+	429	3-dehydroquinate dehydratase
wy_01281	-	1319017	1319655	-	639	hypothetical protein
wy_01282	-	1319758	1320876	+	1119	putative peptidase
wy_01283	*efp*	1320893	1321456	+	564	Elongation factor P
wy_01284	-	1321457	1321960	+	504	hypothetical protein
wy_01285	*pyrR*	1322156	1322788	+	633	Bifunctional protein PyrR
wy_01286	*pyrB*	1322785	1323726	+	942	Aspartate carbamoyltransferase
wy_01287	*pyrC*	1323783	1325102	+	1320	Dihydroorotase
wy_01288	-	1325105	1325647	+	543	hypothetical protein
wy_01289	*carA*	1325644	1326804	+	1161	Carbamoyl-phosphate synthase small chain
wy_01290	*carB*	1326804	1330151	+	3348	Carbamoyl-phosphate synthase large chain
wy_01291	-	1330148	1330984	+	837	orotidine 5'-phosphate decarboxylase
wy_01292	-	1331461	1331781	+	321	hypothetical protein
wy_01293	*gmk*	1331791	1332426	+	636	Guanylate kinase
wy_01294	*rpoZ*	1332494	1332799	+	306	DNA-directed RNA polymerase subunit omega
wy_01295	*coaBC*	1332814	1334100	+	1287	Coenzyme A biosynthesis bifunctional protein CoaBC
wy_01296	*metK*	1334272	1335483	+	1212	S-adenosylmethionine synthase
wy_01297	*priA_1*	1335545	1337527	+	1983	Primosomal protein N'
wy_01298	-	1337524	1337889	-	366	EthD protein
wy_01299	*corA_2*	1337939	1338955	-	1017	Magnesium transport protein CorA
wy_01300	*estB_2*	1339136	1340131	+	996	Extracellular esterase EstB precursor
wy_0130	-	134015	134035	-	207	hypothetical protein

1		2	8			
wy_01302	-	1340581	1342230	+	1650	hypothetical protein
wy_01303	*fmt*	1342338	1343261	+	924	Methionyl-tRNA formyltransferase
wy_01304	*rsmB*	1343258	1344745	+	1488	Ribosomal RNA small subunit methyltransferase B
wy_01305	-	1344768	1345028	-	261	hypothetical protein
wy_01306	-	1345102	1345713	-	612	Nodulation protein S (NodS)
wy_01307	*mshB_2*	1345710	1346489	-	780	1D-myo-inositol 2-acetamido-2-deoxy-alpha-D-glucopyranoside deacetylase
wy_01308	-	1346486	1347433	-	948	Acyl-CoA dehydrogenase, middle domain
wy_01309	-	1347430	1348215	-	786	Glycosyl transferase family 2
wy_01310	*xpkA*	1348312	1350702	-	2391	Xylulose-5-phosphate phosphoketolase
wy_01311	*adh_1*	1350933	1351985	+	1053	Alcohol dehydrogenase
wy_01312	*treP*	1352059	1354434	+	2376	Alpha,alpha-trehalose phosphorylase
wy_01313	*rpe*	1354457	1355128	+	672	Ribulose-phosphate 3-epimerase
wy_01314	*ribD*	1355139	1356149	+	1011	Riboflavin biosynthesis protein RibD
wy_01315	*ribE*	1356177	1356788	+	612	Riboflavin synthase
wy_01316	*ribBA*	1356896	1358143	+	1248	Riboflavin biosynthesis protein RibBA
wy_01317	*ribH*	1358140	1358637	+	498	6,7-dimethyl-8-ribityllumazine synthase
wy_01318	-	1358634	1359092	+	459	hypothetical protein
wy_01319	*uvrC*	1359170	1361260	+	2091	UvrABC system protein C
wy_01320	-	1361257	1362156	+	900	glmZ(sRNA)-inactivating NTPase
wy_01321	-	1362153	1363214	+	1062	Putative gluconeogenesis factor
wy_01322	*whiA*	1363205	1364188	+	984	Putative sporulation transcription regulator WhiA
wy_01323	*gapA*	1364330	1365349	+	1020	Glyceraldehyde-3-phosphate dehydrogenase
wy_01324	*pgk*	1365370	1366596	+	1227	Phosphoglycerate kinase
wy_01325	*tpiA*	1366600	1367385	+	786	Triosephosphate isomerase
wy_01326	*iphP_1*	1367476	1368390	-	915	Tyrosine-protein phosphatase precursor
wy_0132	*iphP_2*	136853	136944	-	915	Tyrosine-protein phosphatase precursor

7		4	8			
wy_01328	-	1369680	1369913	+	234	preprotein translocase subunit SecG
wy_01329	-	1369916	1370326	+	411	Putative pyridoxine/pyridoxamine 5'-phosphate oxidase
wy_01330	*ppc*	1370380	1373172	+	2793	Phosphoenolpyruvate carboxylase
wy_01331	-	1373203	1373679	-	477	MarR family protein
wy_01332	*ybfK*	1373860	1374483	+	624	Carboxylesterase YbfK
wy_01333	-	1374500	1374955	-	456	hypothetical protein
wy_01334	*pgl*	1375256	1376005	-	750	6-phosphogluconolactonase
wy_01335	-	1375998	1376909	-	912	Glucose-6-phosphate dehydrogenase subunit
wy_01336	*zwf*	1376906	1378447	-	1542	Glucose-6-phosphate 1-dehydrogenase
wy_01337	*tal*	1378451	1379584	-	1134	Transaldolase
wy_01338	*tkt*	1379581	1381689	-	2109	Transketolase
wy_01339	*ctaB*	1381894	1382883	+	990	Protoheme IX farnesyltransferase
wy_01340	*qorA_2*	1382943	1383908	-	966	Quinone oxidoreductase 1
wy_01341	-	1383991	1384344	+	354	hypothetical protein
wy_01342	*ctaA*	1384411	1385385	-	975	Heme A synthase
wy_01343	-	1385460	1387043	-	1584	hypothetical protein
wy_01344	-	1387112	1387909	-	798	ABC-2 family transporter protein
wy_01345	*drrA_5*	1387906	1388862	-	957	Daunorubicin/doxorubicin resistance ATP-binding protein DrrA
wy_01346	-	1388890	1390560	-	1671	hypothetical protein
wy_01347	-	1390756	1391454	+	699	Helix-turn-helix domain protein
wy_01348	*sufB*	1391514	1392959	+	1446	FeS cluster assembly protein SufB
wy_01349	*sufD*	1392959	1394143	+	1185	FeS cluster assembly protein SufD
wy_01350	*yurY*	1394198	1394971	+	774	Vegetative protein 296
wy_01351	*sufS_1*	1394978	1396240	+	1263	Cysteine desulfurase
wy_01352	*nifU*	1396243	1396731	+	489	NifU-like protein
wy_0135	*paaD*	139672	139713	+	405	Putative 1,2-phenylacetyl-CoA epoxidase,

3		8	2			subunit D
wy_01354	-	1397148	1397645	+	498	hypothetical protein
wy_01355	*ykoV*	1397653	1398573	-	921	putative DNA repair protein YkoV
wy_01356	-	1398676	1399683	+	1008	phosphoglyceromutase
wy_01357	*crtL*	1399685	1400716	-	1032	Lycopene beta cyclase
wy_01358	*fabG_5*	1400800	1401663	-	864	3-oxoacyl-[acyl-carrier-protein] reductase FabG
wy_01359	-	1401725	1403356	+	1632	putative ABC transporter ATP-binding protein
wy_01360	-	1403498	1403722	+	225	hypothetical protein
wy_01361	-	1403719	1404138	+	420	hypothetical protein
wy_01362	-	1404271	1404642	+	372	hypothetical protein
wy_01363	*acnR*	1404735	1405304	-	570	HTH-type transcriptional repressor AcnR
wy_01364	*acnA*	1405415	1408216	-	2802	Aconitate hydratase
wy_01365	-	1408545	1409102	+	558	hypothetical protein
wy_01366	*ripA_2*	1409366	1410838	+	1473	Peptidoglycan endopeptidase RipA precursor
wy_01367	-	1410941	1412068	+	1128	ATPase family associated with various cellular activities (AAA)
wy_01368	-	1412065	1413021	+	957	hypothetical protein
wy_01369	-	1413018	1413998	+	981	von Willebrand factor type A domain protein
wy_01370	*fabG1_2*	1414077	1414799	+	723	3-oxoacyl-[acyl-carrier-protein] reductase FabG1
wy_01371	*inhA*	1414864	1415661	+	798	Enoyl-[acyl-carrier-protein] reductase [NADH]
wy_01372	*hemH*	1415705	1416775	+	1071	Ferrochelatase
wy_01373	-	1416823	1417131	+	309	hypothetical protein
wy_01374	-	1417135	1417683	+	549	hypothetical protein
wy_01375	-	1417703	1418485	-	783	hypothetical protein
wy_01376	-	1418595	1419404	+	810	hypothetical protein
wy_01377	-	1419489	1419920	+	432	hypothetical protein
wy_01378	*hflK_2*	1419905	1421095	+	1191	Modulator of FtsH protease HflK
wy_0137	-	142109	142148	+	384	hypothetical protein

9		9	2			
wy_01380	-	1421501	1422010	-	510	hypothetical protein
wy_01381	-	1422051	1422920	-	870	hypothetical protein
wy_01382	-	1422925	1423455	-	531	hypothetical protein
wy_01383	-	1423452	1424033	-	582	hypothetical protein
wy_01384	*ydjZ*	1424086	1424739	-	654	TVP38/TMEM64 family inner membrane protein YdjZ
wy_01385	-	1424805	1425626	-	822	hypothetical protein
wy_01386	*mutA*	1425797	1427710	+	1914	Methylmalonyl-CoA mutase small subunit
wy_01387	*mutB*	1427725	1429986	+	2262	Methylmalonyl-CoA mutase large subunit
wy_01388	-	1429973	1430974	+	1002	putative GTPase/MT1543
wy_01389	-	1431045	1431482	-	438	Glyoxalase-like domain protein
wy_01390	*arpA_3*	1431582	1432253	-	672	A-factor receptor protein
wy_01391	*rihA*	1432351	1433409	-	1059	Pyrimidine-specific ribonucleoside hydrolase RihA
wy_01392	-	1433406	1433885	-	480	OsmC-like protein
wy_01393	-	1433950	1434660	+	711	YhhN-like protein
wy_01394	*frc_2*	1434612	1435874	-	1263	Formyl-coenzyme A transferase
wy_01395	-	1436024	1436551	+	528	DinB superfamily protein
wy_01396	-	1436633	1437508	+	876	glucosamine--fructose-6-phosphate aminotransferase
wy_01397	-	1437512	1438762	-	1251	Putative trans-acting enoyl reductase
wy_01398	-	1438804	1439181	-	378	hypothetical protein
wy_01399	-	1439239	1439634	-	396	Universal stress protein
wy_01400	-	1439726	1441180	-	1455	3-ketoacyl-(acyl-carrier-protein) reductase
wy_01401	*isp*	1441393	1442607	+	1215	Major intracellular serine protease precursor
wy_01403	*dapE_2*	1442997	1444352	+	1356	Succinyl-diaminopimelate desuccinylase
wy_01404	-	1444373	1444903	+	531	putative kinase inhibitor
wy_01405	*pyrD*	1444965	1446035	-	1071	Dihydroorotate dehydrogenase (quinone)
wy_0140	-	144608	144707	-	996	hypothetical protein

6		2	7			
wy_01407	*hmuV*	1447202	1448068	-	867	Hemin import ATP-binding protein HmuV
wy_01408	*hmuU_2*	1448068	1449126	-	1059	Hemin transport system permease protein HmuU
wy_01409	*hmuT*	1449123	1450142	-	1020	Hemin-binding periplasmic protein HmuT precursor
wy_01410	*hmuO*	1450139	1450822	-	684	Heme oxygenase
wy_01411	*yhdN*	1450939	1451895	-	957	General stress protein 69
wy_01412	*uppP*	1452072	1452929	+	858	Undecaprenyl-diphosphatase
wy_01413	*pspA_1*	1452966	1453655	+	690	Phosphoserine phosphatase 1
wy_01414	-	1453695	1454288	+	594	hypothetical protein
wy_01415	-	1454294	1455115	+	822	Phosphatidylinositol 3- and 4-kinase
wy_01416	-	1455112	1455570	-	459	transcriptional regulator SlyA
wy_01417	*ydgH*	1455659	1457806	+	2148	Putative membrane protein YdgH
wy_01418	*mshC*	1458431	1459060	+	630	L-cysteine:1D-myo-inositol 2-amino-2-deoxy-alpha-D-glucopyranoside ligase
wy_01419	*ftsH_1*	1459062	1460243	-	1182	ATP-dependent zinc metalloprotease FtsH
wy_01420	*guaD_1*	1460557	1461123	+	567	Guanine deaminase
wy_01421	-	1461125	1462087	+	963	Antibiotic biosynthesis monooxygenase
wy_01422	*mntH*	1462102	1463358	-	1257	Divalent metal cation transporter MntH
wy_01423	-	1463529	1464365	-	837	Chitinase class I
wy_01424	*smc_1*	1464847	1469019	-	4173	Chromosome partition protein Smc
wy_01425	-	1469041	1470453	-	1413	hypothetical protein
wy_01426	-	1470450	1471964	-	1515	hypothetical protein
wy_01427	-	1472014	1473093	-	1080	hypothetical protein
wy_01428	*hsrA_1*	1473260	1474411	-	1152	putative transport protein HsrA
wy_01429	-	1474408	1475316	-	909	PAC2 family protein
wy_01430	*metH*	1475368	1478937	+	3570	Methionine synthase
wy_01431	-	1478930	1479625	+	696	Phosphorylated carbohydrates phosphatase
wy_0143	-	147962	148047	+	852	putative rhodanese-related sulfurtransferase

2		7	8			
wy_0143 3	*rluA_3*	148048 8	148137 5	+	888	Ribosomal large subunit pseudouridine synthase A
wy_0143 4	*hisE*	148137 6	148167 8	+	303	Phosphoribosyl-ATP pyrophosphatase
wy_0143 5	*hisG*	148174 3	148259 4	+	852	ATP phosphoribosyltransferase
wy_0143 6	-	148268 4	148349 3	-	810	hypothetical protein
wy_0143 7	-	148349 0	148437 7	-	888	PD-(D/E)XK nuclease superfamily protein
wy_0143 8	*trmI*	148446 3	148531 4	+	852	tRNA (adenine(58)-N(1))-methyltransferase TrmI
wy_0143 9	-	148538 3	148586 8	-	486	hypothetical protein
wy_0144 0	*arc*	148606 5	148782 8	+	1764	Proteasome-associated ATPase
wy_0144 1	-	148782 5	148834 3	-	519	Nudix hydrolase
wy_0144 2	*hmrR*	148837 7	148940 2	-	1026	HTH-type transcriptional regulator HmrR
wy_0144 3	-	148942 6	149008 2	-	657	hypothetical protein
wy_0144 4	-	149020 2	149058 8	+	387	hypothetical protein
wy_0144 5	*dop*	149064 4	149214 3	+	1500	Pup deamidase/depupylase
wy_0144 6	*pup*	149227 5	149246 9	+	195	Prokaryotic ubiquitin-like protein Pup
wy_0144 7	*prcB1*	149246 6	149334 1	+	876	Proteasome subunit beta 1 precursor
wy_0144 8	*prcA1*	149333 8	149413 2	+	795	Proteasome subunit alpha 1
wy_0144 9	*dagK_3*	149412 9	149554 7	-	1419	Diacylglycerol kinase
wy_0145 0	*pafA*	149568 0	149702 3	+	1344	Pup--protein ligase
wy_0145 1	-	149713 9	149812 5	+	987	hypothetical protein
wy_0145 2	-	149812 2	149910 5	+	984	hypothetical protein
wy_0145 3	*tatA*	149933 5	149960 7	+	273	Sec-independent protein translocase protein TatA
wy_0145 4	*tatC*	149973 5	150063 1	+	897	Sec-independent protein translocase protein TatC
wy_0145 5	-	150062 8	150332 4	+	2697	ski2-like helicase
wy_0145 6	-	150342 9	150369 2	+	264	hypothetical protein
wy_0145 7	-	150388 8	150460 4	+	717	hypothetical protein
wy_0145	*polA_1*	150462	150556	-	945	DNA polymerase I

8		0	4			
wy_01459	*pepQ*	1505601	1506755	+	1155	Xaa-Pro dipeptidase
wy_01460	-	1506768	1507109	-	342	hypothetical protein
wy_01461	-	1507341	1507754	-	414	Pyridoxamine 5'-phosphate oxidase
wy_01462	*actIII*	1507799	1508554	+	756	Putative ketoacyl reductase
wy_01463	*cobL*	1508551	1509795	+	1245	Precorrin-6Y C(5,15)-methyltransferase [decarboxylating]
wy_01464	*cobM*	1509792	1510541	+	750	Precorrin-4 C(11)-methyltransferase
wy_01465	*cobK*	1510550	1511302	+	753	Precorrin-6A reductase
wy_01466	-	1511464	1512330	+	867	putative oxidoreductase
wy_01467	-	1512388	1513053	+	666	hypothetical protein
wy_01468	-	1513064	1513276	+	213	hypothetical protein
wy_01469	-	1513293	1513976	-	684	Fatty acid hydroxylase superfamily protein
wy_01470	*mce2R*	1513973	1514707	-	735	HTH-type transcriptional regulator Mce2R
wy_01471	-	1514740	1515171	-	432	hypothetical protein
wy_01472	-	1515207	1515923	-	717	hypothetical protein
wy_01473	-	1516052	1516804	+	753	hypothetical protein
wy_01474	*cobI*	1516890	1518380	-	1491	Precorrin-2 C(20)-methyltransferase
wy_01475	*cobH*	1518377	1519003	-	627	Precorrin-8X methylmutase
wy_01476	-	1519023	1520162	-	1140	ferredoxin-nitrite reductase
wy_01477	*cobN*	1520372	1523971	+	3600	Aerobic cobaltochelatase subunit CobN
wy_01478	-	1523981	1524622	-	642	hypothetical protein
wy_01479	-	1524801	1527110	+	2310	ABC transporter ATP-binding/permease protein
wy_01480	-	1527115	1528182	-	1068	Calcineurin-like phosphoesterase
wy_01481	-	1528324	1528710	+	387	Pyridoxamine 5'-phosphate oxidase
wy_01482	-	1528782	1529300	+	519	hypothetical protein
wy_01483	-	1529392	1530594	-	1203	site-specific tyrosine recombinase XerD
wy_0148	-	153082	153180	-	981	hypothetical protein

4		1	1			
wy_0148 5	*fgd1_2*	153230 1	153328 7	-	987	F420-dependent glucose-6-phosphate dehydrogenase
wy_0148 6	-	153331 2	153370 4	-	393	hypothetical protein
wy_0148 7	-	153371 3	153508 0	-	1368	Putative modulator of DNA gyrase
wy_0148 8	-	153510 6	153665 6	-	1551	protease TldD
wy_0148 9	-	153672 4	153824 4	-	1521	hypothetical protein
wy_0149 0	-	153836 1	153899 6	-	636	Bacterial regulatory proteins, tetR family
wy_0149 1	-	153916 7	153976 9	+	603	hypothetical protein
wy_0149 2	-	153984 0	154049 0	-	651	hypothetical protein
wy_0149 3	-	154070 4	154119 5	+	492	phage T7 F exclusion suppressor FxsA
wy_0149 4	-	154120 8	154280 9	+	1602	imidazolonepropionase
wy_0149 5	*lnt*	154279 9	154443 0	+	1632	Apolipoprotein N-acyltransferase
wy_0149 6	-	154458 9	154481 3	+	225	MbtH-like protein
wy_0149 7	*lgrB_1*	154501 6	156988 8	+	24873	Linear gramicidin synthase subunit B
wy_0149 8	*dhbF_1*	156988 5	157182 2	+	1938	Dimodular nonribosomal peptide synthase
wy_0149 9	-	157194 8	157233 4	+	387	hypothetical protein
wy_0150 0	-	157236 3	157336 1	-	999	P-aminobenzoate N-oxygenase AurF
wy_0150 1	*tyrA*	157335 8	157428 4	-	927	T-protein
wy_0150 2	*trpE_1*	157428 1	157641 6	-	2136	Anthranilate synthase component 1
wy_0150 3	-	157642 4	157679 5	-	372	bifunctional chorismate mutase/prephenate dehydrogenase
wy_0150 4	-	157722 2	157846 9	+	1248	putative inactive lipase/MT1628
wy_0150 5	*rbpA*	157853 6	157887 4	-	339	RNA polymerase-binding protein RbpA
wy_0150 6	-	157904 6	157937 8	+	333	hypothetical protein
wy_0150 7	-	157947 9	157964 9	+	171	hypothetical protein
wy_0150 8	*cmk_1*	157960 3	158114 7	+	1545	Cytidylate kinase
wy_0150 9	*acg*	158114 4	158211 2	-	969	Putative NAD(P)H nitroreductase acg
wy_0151	-	158232	158317	+	852	Universal stress protein/MT2061

0		5	6			
wy_01511	-	1583168	1584124	-	957	Universal stress protein/MT2061
wy_01512	-	1584134	1586662	-	2529	putative glycosyl hydrolase/MT2062
wy_01513	*virF*	1586750	1587190	+	441	Virulence regulon transcriptional activator VirF
wy_01514	-	1587201	1587611	+	411	Glyoxalase-like domain protein
wy_01515	-	1587613	1588224	+	612	Glyoxalase-like domain protein
wy_01516	*uvrA_1*	1588274	1590664	+	2391	UvrABC system protein A
wy_01517	-	1591120	1591800	-	681	hypothetical protein
wy_01518	-	1591825	1592058	-	234	hypothetical protein
wy_01519	*integrase*	1592489	1593514	+	1026	Tyrosine recombinase XerD
wy_01520	*MFS*	1594018	1595391	+	1374	Multidrug resistance protein 3
wy_01521	-	1595543	1596916	+	1374	NADH dehydrogenase
wy_01522	-	1596928	1597389	-	462	esterase
wy_01523	-	1597647	1598411	-	765	hypothetical protein
wy_01524	*iupA*	1598583	1600061	+	1479	putative siderophore transport system permease protein YfiZ precursor
wy_01525	*iupB*	1600058	1601161	+	1104	putative siderophore transport system permease protein YfhA
wy_01526	*iupC*	1601158	1602006	+	849	putative siderophore transport system ATP-binding protein YusV
wy_01527	-	1602013	1603908	-	1896	Metallo-beta-lactamase
wy_01528	*tmrB*	1604027	1604677	-	651	Tunicamycin resistance protein
wy_01529	-	1604723	1605466	-	744	Alpha/beta hydrolase family protein
wy_01530	-	1605539	1606234	+	696	HTH domain protein
wy_01531	-	1606277	1606897	-	621	RES domain protein
wy_01532	-	1606894	1607427	-	534	hypothetical protein
wy_01533	*mprF*	1607512	1610055	-	2544	Phosphatidylglycerol lysyltransferase
wy_01534	-	1610052	1611359	-	1308	Putative esterase
wy_01535	*blaI_2*	1611493	1611945	+	453	Transcriptional regulator BlaI

wy_01536	*htpX_2*	1611973	1612932	+	960	Protease HtpX
wy_01537	*deaD_2*	1613174	1615360	+	2187	ATP-dependent RNA helicase DeaD
wy_01538	*gndA*	1615512	1616972	+	1461	6-phosphogluconate dehydrogenase, NADP(+)-dependent, decarboxylating
wy_01539	-	1617172	1617540	-	369	hypothetical protein
wy_01540	*rhlE_1*	1617625	1618971	+	1347	ATP-dependent RNA helicase RhlE
wy_01541	-	1618975	1619850	+	876	hypothetical protein
wy_01542	*yhhW_2*	1619929	1620954	+	1026	Quercetin 2,3-dioxygenase
wy_01543	*guaB_1*	1621586	1622431	+	846	Inosine-5'-monophosphate dehydrogenase
wy_01544	-	1622568	1622723	+	156	hypothetical protein
wy_01545	*corC_1*	1622720	1624090	+	1371	Magnesium and cobalt efflux protein CorC
wy_01546	*tlyC_1*	1624083	1625171	+	1089	Hemolysin C
wy_01547	-	1625177	1626055	+	879	hypothetical protein
wy_01548	-	1626195	1628537	+	2343	Putative HTH-type transcriptional regulator/MT0914
wy_01549	*lplA*	1628546	1629595	-	1050	putative lipoate-protein ligase A
wy_01550	-	1629707	1630063	-	357	Glyoxalase-like domain protein
wy_01551	*glcB*	1630647	1632830	+	2184	Malate synthase G
wy_01552	-	1632978	1634723	+	1746	von Willebrand factor type A domain protein
wy_01553	-	1634726	1635874	+	1149	putative metallophosphoesterase
wy_01554	*bvgA*	1636042	1638516	+	2475	Virulence factors putative positive transcription regulator BvgA
wy_01555	-	1638658	1639098	-	441	hypothetical protein
wy_01556	*ubiE_1*	1639135	1639974	-	840	Demethylmenaquinone methyltransferase
wy_01557	*gcvP*	1640161	1643001	-	2841	Glycine dehydrogenase (decarboxylating)
wy_01558	-	1643357	1643983	-	627	hypothetical protein
wy_01559	-	1644211	1644684	-	474	hypothetical protein
wy_01560	-	1644784	1645521	-	738	zinc-responsive transcriptional regulator
wy_01561	*garA_1*	1645521	1646015	-	495	Glycogen accumulation regulator GarA

wy_01562	*gcvH*	1646231	1646635	-	405	Glycine cleavage system H protein
wy_01563	-	1646680	1647450	-	771	hypothetical protein
wy_01564	-	1647456	1647812	-	357	hypothetical protein
wy_01565	-	1647809	1648675	-	867	hypothetical protein
wy_01566	*pgsA*	1648672	1649283	-	612	CDP-diacylglycerol--glycerol-3-phosphate 3-phosphatidyltransferase
wy_01567	-	1649377	1650420	-	1044	Strictosidine synthase
wy_01568	-	1650462	1651079	-	618	putative NUDIX hydrolase
wy_01569	-	1651307	1651777	+	471	Bacterial transcription activator, effector binding domain
wy_01570	-	1651841	1652338	+	498	hypothetical protein
wy_01571	-	1652346	1652570	+	225	hypothetical protein
wy_01573	*der*	1652878	1654323	-	1446	GTPase Der
wy_01574	*cmk_2*	1654320	1655012	-	693	Cytidylate kinase
wy_01575	*rluB*	1655009	1655974	-	966	Ribosomal large subunit pseudouridine synthase B
wy_01576	*scpB*	1655987	1656700	-	714	Segregation and condensation protein B
wy_01577	*scpA*	1656697	1657524	-	828	Segregation and condensation protein A
wy_01578	*soj_1*	1657524	1658453	-	930	Sporulation initiation inhibitor protein Soj
wy_01579	*xerD_2*	1658554	1659270	-	717	Tyrosine recombinase XerD
wy_01580	*nudF*	1659344	1660132	-	789	ADP-ribose pyrophosphatase
wy_01581	*pyrG*	1660139	1661887	-	1749	CTP synthase
wy_01582	*mctB*	1662046	1662981	-	936	Copper transporter MctB precursor
wy_01583	-	1662987	1664183	-	1197	hypothetical protein
wy_01584	*recN*	1664271	1666046	-	1776	DNA repair protein RecN
wy_01585	*ppnK*	1666049	1666990	-	942	Inorganic polyphosphate/ATP-NAD kinase
wy_01586	*tlyA*	1666987	1667766	-	780	16S/23S rRNA (cytidine-2'-O)-methyltransferase TlyA
wy_01587	*yutF*	1668117	1669151	-	1035	putative hydrolase YutF
wy_01588	-	1669148	1669795	-	648	hypothetical protein

wy_01589	-	1669750	1670241	-	492	hypothetical protein
wy_01593	*tyrS*	1676159	1677436	-	1278	Tyrosine--tRNA ligase
wy_01594	-	1677455	1678078	-	624	3-methyladenine DNA glycosylase
wy_01595	*ybhF_3*	1678188	1678949	+	762	putative ABC transporter ATP-binding protein YbhF
wy_01596	*ybhR_2*	1678946	1679707	+	762	Inner membrane transport permease YbhR
wy_01597	*kstR2_7*	1679704	1680375	+	672	HTH-type transcriptional repressor KstR2
wy_01598	-	1680320	1680535	-	216	hypothetical protein
wy_01599	*lcfB_9*	1680600	1683599	-	3000	Long-chain-fatty-acid--CoA ligase
wy_01600	*argH*	1683711	1685135	-	1425	Argininosuccinate lyase
wy_01601	*argG*	1685139	1686338	-	1200	Argininosuccinate synthase
wy_01602	*argR*	1686388	1686906	-	519	Arginine repressor
wy_01603	*argF*	1686903	1687841	-	939	Ornithine carbamoyltransferase
wy_01604	*argD*	1687845	1689065	-	1221	Acetylornithine aminotransferase
wy_01605	*argB*	1689062	1689985	-	924	Acetylglutamate kinase
wy_01606	*argJ*	1689982	1691220	-	1239	Arginine biosynthesis bifunctional protein ArgJ
wy_01607	*argC*	1691217	1692299	-	1083	N-acetyl-gamma-glutamyl-phosphate reductase
wy_01608	-	1692426	1693205	-	780	GDSL-like Lipase/Acylhydrolase
wy_01609	-	1693262	1693699	-	438	hypothetical protein
wy_01610	*pheT*	1693767	1696256	-	2490	Phenylalanine--tRNA ligase beta subunit
wy_01611	*pheS*	1696287	1697357	-	1071	Phenylalanine--tRNA ligase alpha subunit
wy_01612	*aviRb*	1697463	1698263	-	801	23S rRNA (uridine(2479)-2'-O)-methyltransferase
wy_01613	*rplT*	1698287	1698676	-	390	50S ribosomal protein L20
wy_01614	*rpmI*	1698740	1698934	-	195	50S ribosomal protein L35
wy_01615	*infC*	1698994	1699515	-	522	Translation initiation factor IF-3
wy_01616	-	1699989	1700366	+	378	hypothetical protein
wy_01617	-	1700986	1701108	+	123	hypothetical protein

wy_01618	*uvrA_2*	1701191	1704163	-	2973	UvrABC system protein A
wy_01619	*baeB_1*	1704317	1705009	+	693	putative polyketide biosynthesis zinc-dependent hydrolase BaeB
wy_01620	-	1705178	1708834	+	3657	putative glycosyl hydrolase/MT2062
wy_01621	-	1709044	1709958	+	915	DoxX
wy_01622	-	1710052	1710711	-	660	hypothetical protein
wy_01623	*gabD1*	1710726	1712189	-	1464	Succinate-semialdehyde dehydrogenase [NADP(+)] 1
wy_01624	*helD_2*	1712370	1714649	+	2280	Helicase IV
wy_01625	-	1714731	1715147	+	417	hypothetical protein
wy_01626	-	1715152	1715604	-	453	Universal stress protein/MT1672
wy_01627	-	1715804	1716676	-	873	hypothetical protein
wy_01628	*uvrB*	1716698	1718854	-	2157	UvrABC system protein B
wy_01629	-	1718958	1719542	+	585	hypothetical protein
wy_01630	*coaE*	1719549	1720808	-	1260	Dephospho-CoA kinase
wy_01631	*rpsA*	1721390	1722862	-	1473	30S ribosomal protein S1
wy_01632	-	1723163	1724011	+	849	Phthiotriol/phenolphthiotriol dimycocerosates methyltransferase
wy_01633	*glnQ_2*	1724015	1724779	-	765	Glutamine transport ATP-binding protein GlnQ
wy_01634	*yecS*	1724776	1725720	-	945	Inner membrane amino-acid ABC transporter permease protein YecS
wy_01635	*glnH_3*	1725741	1726637	-	897	Glutamine-binding periplasmic protein precursor
wy_01636	*hicd*	1726723	1727820	-	1098	Homoisocitrate dehydrogenase
wy_01637	-	1727840	1728367	+	528	hypothetical protein
wy_01638	*trxC*	1728505	1728870	+	366	Putative thioredoxin-2
wy_01639	*polA*	1728941	1731604	-	2664	DNA polymerase I
wy_01640	*AmiR_NasR*	1731761	1732381	-	621	putative transcriptional regulatory protein pdtaR
wy_01642	-	1732664	1733821	-	1158	integrase
wy_01643	-	1734067	1734312	+	246	hypothetical
wy_0164	-	173458	173517	+	597	hypothetical protein

4		1	7			
wy_01645	-	1735174	1736319	+	1146	hypothetical protein
wy_01646	-	1736341	1736727	+	387	hypothetical protein
wy_01647	-	1737095	1737757	+	663	hypothetical protein
wy_01648	-	1737973	1738302	+	330	hypothetical protein
wy_01649	-	1738735	1739259	+	525	antirepressor protein
wy_01650	-	1739374	1739658	-	285	hypothetical protein
wy_01651	-	1739856	1740281	+	426	hypothetical protein
wy_01652	-	1740559	1740888	+	330	hypothetical protein
wy_01653	-	1741264	1741482	+	219	hypothetical protein
wy_01655	-	1741757	1742176	+	420	hypothetical protein
wy_01656	-	1742335	1742634	+	300	hypothetical protein
wy_01657	*smc*	1742635	1746993	+	4359	Chromosome partition protein Smc
wy_01658	-	1747105	1747347	+	243	hypothetical protein
wy_01659	-	1747378	1748034	-	657	hypothetical protein
wy_01660	-	1748196	1749515	-	1320	hypothetical protein
wy_01661	*hin*	1749840	1750424	+	585	DNA-invertase hin
wy_01662	-	1750824	1753277	-	2454	hypothetical protein
wy_01663	-	1754329	1754553	+	225	hypothetical protein
wy_01664	-	1754581	1754865	+	285	hypothetical protein
wy_01665	-	1755245	1757761	-	2517	hypothetical protein
wy_01666	-	1757922	1758716	-	795	hypothetical protein
wy_01667	*dhaA*	1759425	1760300	-	876	Haloalkane dehalogenase
wy_01668	-	1760673	1761125	+	453	hypothetical protein
wy_01669	-	1761122	1762315	+	1194	lipid-transfer protein
wy_01670	-	1762409	1763068	+	660	N-carbamoylsarcosine amidase
wy_0167	-	176309	176333	+	234	hypothetical protein

1		8	1			
wy_01672	*rpfE_2*	1763504	1763830	+	327	Resuscitation-promoting factor RpfE precursor
wy_01673	*bacA*	1763900	1765753	-	1854	Vitamin B12 transport ATP-binding protein BacA
wy_01674	*tesB_2*	1765846	1766742	-	897	Acyl-CoA thioesterase 2
wy_01675	*pyk*	1766758	1768092	-	1335	Pyruvate kinase
wy_01676	*gltB_1*	1768248	1769753	-	1506	Glutamate synthase [NADPH] small chain
wy_01677	*gltB_2*	1769746	1774329	-	4584	Glutamate synthase [NADPH] large chain
wy_01678	-	1774618	1775364	-	747	hypothetical protein
wy_01679	*lgt*	1775361	1776296	-	936	Prolipoprotein diacylglyceryl transferase
wy_01680	*trpA*	1776302	1777105	-	804	Tryptophan synthase alpha chain
wy_01681	*trpB_2*	1777102	1778385	-	1284	Tryptophan synthase beta chain
wy_01682	*trpC*	1778471	1779280	-	810	Indole-3-glycerol phosphate synthase
wy_01683	-	1779358	1780095	-	738	Tryptophan-associated transmembrane protein (Trp_oprn_chp)
wy_01684	*trpE_2*	1780088	1781689	-	1602	Anthranilate synthase component 1
wy_01685	*bcp_1*	1781748	1782206	+	459	Putative peroxiredoxin bcp
wy_01686	-	1782214	1782990	-	777	LigB family dioxygenase
wy_01687	-	1783135	1783629	+	495	MarR family protein
wy_01688	-	1783634	1784440	+	807	DNA-binding transcriptional regulator AraC
wy_01689	-	1784496	1785245	+	750	Transglutaminase-like superfamily protein
wy_01690	*dmpI*	1785193	1785372	-	180	2-hydroxymuconate tautomerase
wy_01691	-	1785676	1786479	+	804	NADP-dependent 7-alpha-hydroxysteroid dehydrogenase
wy_01692	-	1786427	1787245	-	819	hypothetical protein
wy_01693	-	1787279	1787605	-	327	hypothetical protein
wy_01694	-	1787647	1788870	-	1224	EAL domain protein
wy_01695	-	1789028	1789531	+	504	hypothetical protein
wy_01696	-	1789566	1790000	-	435	hypothetical protein

wy_01697	-	1790024	1790503	-	480	hypothetical protein
wy_01698	-	1790694	1791167	+	474	Polyketide cyclase / dehydrase and lipid transport
wy_01699	*crtI_1*	1791172	1792776	+	1605	Phytoene desaturase (lycopene-forming)
wy_01700	-	1792779	1793846	+	1068	short chain dehydrogenase
wy_01701	-	1793850	1794326	-	477	hypothetical protein
wy_01702	-	1794499	1794924	+	426	hypothetical protein
wy_01703	-	1794978	1795148	+	171	hypothetical protein
wy_01704	*tuaD_1*	1795162	1795338	-	177	UDP-glucose 6-dehydrogenase TuaD
wy_01705	*tuaD_2*	1795335	1796468	-	1134	UDP-glucose 6-dehydrogenase TuaD
wy_01706	*rfaQ*	1796477	1797403	-	927	Lipopolysaccharide core heptosyltransferase RfaQ
wy_01707	-	1797394	1798083	-	690	putative oxidoreductase
wy_01708	-	1798533	1799192	+	660	hypothetical protein
wy_01709	*ydhP_2*	1799331	1800524	-	1194	Inner membrane transport protein YdhP
wy_01710	*comR_2*	1800610	1801212	-	603	HTH-type transcriptional repressor ComR
wy_01711	*ydbC_1*	1801280	1802152	-	873	Putative oxidoreductase YdbC
wy_01712	*ytcD*	1802247	1802729	+	483	putative HTH-type transcriptional regulator YtcD
wy_01713	-	1802817	1803320	-	504	CGNR zinc finger
wy_01714	*dhaA_2*	1803398	1804276	+	879	Haloalkane dehalogenase
wy_01715	*yhfK_2*	1804273	1804959	+	687	putative sugar epimerase YhfK
wy_01716	-	1805221	1805577	+	357	hypothetical protein
wy_01717	-	1805910	1806578	-	669	hypothetical protein
wy_01718	-	1806925	1808748	+	1824	hypothetical protein
wy_01719	-	1809613	1810068	-	456	hypothetical protein
wy_01720	-	1810206	1810502	+	297	hypothetical protein
wy_01721	*yflN_1*	1810708	1811412	+	705	putative metallo-hydrolase YflN
wy_01722	-	1811440	1812015	+	576	hypothetical protein

wy_01723	-	1812084	1813241	-	1158	haloalkane dehalogenase
wy_01724	-	1813320	1813574	-	255	hypothetical protein
wy_01725	-	1813763	1814506	+	744	hypothetical protein
wy_01726	-	1814512	1815528	+	1017	Polyketide cyclase / dehydrase and lipid transport
wy_01727	*gvpA_1*	1815532	1815930	+	399	Gas vesicle structural protein
wy_01728	-	1815927	1816709	+	783	Gas vesicle synthesis protein GvpL/GvpF
wy_01729	-	1816711	1816962	+	252	Gas vesicle protein G
wy_01730	-	1816969	1817268	+	300	Gas vesicle synthesis protein GvpO
wy_01731	*gvpA_2*	1817265	1817678	+	414	Gas vesicle structural protein
wy_01732	-	1817668	1818447	+	780	Gas vesicle synthesis protein GvpL/GvpF
wy_01733	-	1818444	1818641	+	198	Gas vesicle protein
wy_01734	-	1818638	1818973	+	336	Gas vesicle protein K
wy_01735	*mshA_1*	1819136	1820353	+	1218	D-inositol 3-phosphate glycosyltransferase
wy_01736	*rffG*	1820350	1821180	+	831	dTDP-glucose 4,6-dehydratase 2
wy_01737	*novN*	1821177	1821449	+	273	Decarbamoylnovobiocin carbamoyltransferase
wy_01738	*rsbW_2*	1821798	1822238	+	441	Anti-sigma-F factor RsbW
wy_01739	*sigF_2*	1822235	1823014	+	780	RNA polymerase sigma factor SigF
wy_01740	-	1823240	1823674	+	435	STAS domain protein
wy_01741	-	1823702	1824430	+	729	ANTAR domain protein
wy_01742	-	1824688	1824945	-	258	ANTAR domain protein
wy_01743	*cinA_2*	1825236	1825715	+	480	Putative competence-damage inducible protein
wy_01744	*fdm*	1825749	1826927	-	1179	Formaldehyde dismutase
wy_01745	-	1827160	1827444	+	285	hypothetical protein
wy_01746	-	1827515	1827760	+	246	CsbD-like protein
wy_01747	*prmC_2*	1827764	1828492	+	729	Release factor glutamine methyltransferase
wy_01748	*nepI*	1828692	1829867	-	1176	Purine ribonucleoside efflux pump NepI

wy_01749	*sdpR_2*	1829971	1830282	-	312	Transcriptional repressor SdpR
wy_01750	-	1830673	1831452	-	780	Methyltransferase domain protein
wy_01751	-	1831608	1832345	+	738	hypothetical protein
wy_01752	-	1832448	1832771	+	324	hypothetical protein
wy_01753	-	1832722	1833123	+	402	hypothetical protein
wy_01754	-	1833714	1834478	+	765	hypothetical protein
wy_01755	-	1835572	1835661	+	90	hypothetical protein
wy_01756	-	1835979	1836428	+	450	MarR family protein
wy_01757	-	1836483	1836905	-	423	ChaB
wy_01758	*fgd1_3*	1836943	1837893	-	951	F420-dependent glucose-6-phosphate dehydrogenase
wy_01759	*ydbD*	1838103	1838993	+	891	putative manganese catalase
wy_01760	-	1839075	1839650	-	576	hypothetical protein
wy_01761	*yghA_1*	1839790	1840488	+	699	putative oxidoreductase YghA
wy_01762	*ydaD*	1840455	1840688	+	234	General stress protein 39
wy_01763	-	1840715	1841134	-	420	hypothetical protein
wy_01764	-	1841131	1842558	-	1428	Bifunctional P-450/NADPH-P450 reductase
wy_01765	-	1842683	1842859	+	177	hypothetical protein
wy_01766	-	1842957	1843214	+	258	hypothetical protein
wy_01767	*petC1*	1843219	1844739	+	1521	Cytochrome b6-f complex iron-sulfur subunit 1
wy_01768	*speA*	1845023	1845265	+	243	Arginine decarboxylase
wy_01769	-	1845342	1845494	+	153	hypothetical protein
wy_01770	-	1845511	1846029	+	519	hypothetical protein
wy_01771	*fdhA_2*	1846051	1847217	+	1167	Glutathione-independent formaldehyde dehydrogenase
wy_01772	*ybdK_1*	1847235	1848338	+	1104	Carboxylate-amine ligase YbdK
wy_01773	-	1848442	1848861	+	420	hypothetical protein
wy_01774	-	1848858	1849118	+	261	hypothetical protein

wy_01775	*slmA*	1849106	1849717	+	612	Nucleoid occlusion factor SlmA
wy_01776	*murE_1*	1849714	1851219	-	1506	UDP-N-acetylmuramoyl-L-alanyl-D-glutamate--2, 6-diaminopimelate ligase
wy_01777	-	1851529	1852413	+	885	hypothetical protein
wy_01778	*fabG_6*	1852492	1852710	+	219	3-oxoacyl-[acyl-carrier-protein] reductase FabG
wy_01779	*adaA*	1852721	1854178	-	1458	Bifunctional transcriptional activator/DNA repair enzyme AdaA
wy_01780	-	1854661	1855068	-	408	MarR family protein
wy_01781	*amiC_1*	1855209	1856282	+	1074	Aliphatic amidase expression-regulating protein
wy_01782	*ureI*	1856364	1856999	+	636	Acid-activated urea channel
wy_01783	*amiE*	1857036	1858073	+	1038	Aliphatic amidase
wy_01784	*clpB_1*	1858088	1859122	+	1035	Chaperone protein ClpB
wy_01785	-	1859399	1859857	-	459	18 kDa heat shock protein
wy_01786	*ssuD_1*	1860114	1860986	-	873	Alkanesulfonate monooxygenase
wy_01787	-	1860983	1861546	-	564	Putative lumazine-binding protein
wy_01788	-	1861623	1861910	-	288	hypothetical protein
wy_01789	-	1862073	1862633	-	561	transcriptional regulator BetI
wy_01790	-	1862633	1863154	-	522	hypothetical protein
wy_01791	-	1863275	1864471	-	1197	D-alanyl-D-alanine carboxypeptidase precursor
wy_01792	-	1864845	1865786	+	942	Putative DNA ligase-like protein/MT0965
wy_01793	-	1865836	1866183	+	348	hypothetical protein
wy_01794	-	1866434	1867174	-	741	ANTAR domain protein
wy_01795	-	1867319	1868275	-	957	hypothetical protein
wy_01796	-	1868407	1868967	-	561	putative transcriptional regulator
wy_01797	-	1869088	1870296	+	1209	Major Facilitator Superfamily protein
wy_01798	-	1870323	1870670	-	348	phosphoribosyl-AMP cyclohydrolase
wy_01799	*hisF*	1870667	1871440	-	774	Imidazole glycerol phosphate synthase subunit HisF
wy_01800	*impA*	1871437	1872270	-	834	Inositol-1-monophosphatase ImpA

wy_01801	*priA_2*	1872285	1873019	-	735	Phosphoribosyl isomerase A
wy_01802	-	1873038	1873322	-	285	hypothetical protein
wy_01803	*hisH*	1873319	1873954	-	636	Imidazole glycerol phosphate synthase subunit HisH
wy_01804	-	1873964	1875115	-	1152	Major Facilitator Superfamily protein
wy_01805	-	1875115	1875267	-	153	hypothetical protein
wy_01806	*hisB*	1875272	1875886	-	615	Imidazoleglycerol-phosphate dehydratase
wy_01807	*hisC*	1875883	1877031	-	1149	Histidinol-phosphate aminotransferase
wy_01808	*hisD*	1877028	1878350	-	1323	Histidinol dehydrogenase
wy_01809	*choA*	1878586	1880193	+	1608	Cholesterol oxidase precursor
wy_01810	*bglA_2*	1880204	1881490	+	1287	Beta-glucosidase A
wy_01811	*rhtB*	1881539	1882156	+	618	Homoserine/homoserine lactone efflux protein
wy_01812	-	1882159	1882557	-	399	Low affinity iron permease
wy_01813	-	1882635	1883702	-	1068	hypothetical protein
wy_01814	-	1883702	1883947	-	246	Iron-binding zinc finger CDGSH type
wy_01815	-	1883944	1884654	-	711	hypothetical protein
wy_01816	*katE*	1884718	1886841	-	2124	Catalase HPII
wy_01817	-	1886880	1887755	-	876	hypothetical protein
wy_01818	-	1887752	1888750	-	999	hypothetical protein
wy_01819	-	1888747	1889862	-	1116	Glycogen synthase
wy_01820	-	1889859	1890926	-	1068	hypothetical protein
wy_01821	-	1891014	1891163	-	150	hypothetical protein
wy_01822	*sigF_3*	1891666	1892475	+	810	RNA polymerase sigma factor SigF
wy_01823	*pknK_1*	1892518	1895109	-	2592	Serine/threonine-protein kinase PknK
wy_01824	-	1895249	1895851	-	603	hypothetical protein
wy_01825	*nadC*	1895870	1896730	-	861	Nicotinate-nucleotide pyrophosphorylase [carboxylating]
wy_01826	*nadB*	1896727	1898304	-	1578	L-aspartate oxidase

wy_01827	*nadA*	1898304	1899335	-	1032	Quinolinate synthase A
wy_01828	-	1899439	1900173	+	735	bifunctional nicotinamide mononucleotide adenylyltransferase/ADP-ribose pyrophosphatase
wy_01829	-	1900217	1901833	+	1617	hypothetical protein
wy_01830	-	1901986	1902210	-	225	hypothetical protein
wy_01831	-	1902595	1903476	+	882	hypothetical protein
wy_01832	-	1903484	1903996	-	513	hypothetical protein
wy_01833	-	1904059	1904304	-	246	hypothetical protein
wy_01834	*bioB*	1904291	1905310	-	1020	Biotin synthase
wy_01835	*tetR_1*	1905498	1906130	+	633	Tetracycline repressor protein class E
wy_01836	-	1906162	1906485	-	324	hypothetical protein
wy_01837	-	1906631	1906966	-	336	hypothetical protein
wy_01838	-	1907111	1908250	+	1140	Secretory lipase
wy_01839	-	1908267	1909013	-	747	hypothetical protein
wy_01840	*fosA*	1909075	1909458	-	384	Glutathione transferase FosA
wy_01841	*lgrB_2*	1909564	1926006	-	16443	Linear gramicidin synthase subunit B
wy_01842	-	1926199	1926519	-	321	hypothetical protein
wy_01843	*frdB*	1926767	1927522	-	756	Fumarate reductase iron-sulfur subunit
wy_01844	*sdhA_1*	1927524	1929455	-	1932	Succinate dehydrogenase flavoprotein subunit
wy_01845	-	1929523	1930326	-	804	hypothetical protein
wy_01846	-	1930338	1930622	-	285	hypothetical protein
wy_01847	*rutA_2*	1930880	1931755	+	876	Pyrimidine monooxygenase RutA
wy_01848	*ahpC*	1931959	1932522	+	564	Alkyl hydroperoxide reductase subunit C
wy_01849	*ahpF*	1932700	1934289	+	1590	Alkyl hydroperoxide reductase subunit F
wy_01850	-	1934463	1934978	+	516	hypothetical protein
wy_01851	*bioD*	1934968	1935663	-	696	ATP-dependent dethiobiotin synthetase BioD
wy_0185	*bioF1*	193566	193682	-	1167	8-amino-7-oxononanoate synthase 1

2		0	6			
wy_01853	*bioA*	1936980	1938284	-	1305	Adenosylmethionine-8-amino-7-oxononanoate aminotransferase
wy_01854	*oatA_1*	1938400	1940490	+	2091	O-acetyltransferase OatA
wy_01855	-	1940500	1941747	-	1248	Steroid C26-monooxygenase
wy_01856	-	1941966	1942748	+	783	putative acetyltransferase
wy_01857	-	1942788	1943372	+	585	hypothetical protein
wy_01858	-	1943403	1944017	+	615	hypothetical protein
wy_01859	-	1944230	1945528	+	1299	HNH endonuclease
wy_01860	-	1945727	1946932	-	1206	hypothetical protein
wy_01861	-	1947038	1947400	-	363	hypothetical protein
wy_01862	*adh_2*	1947530	1948570	+	1041	Alcohol dehydrogenase
wy_01863	-	1948567	1948962	+	396	hypothetical protein
wy_01864	-	1949009	1950274	+	1266	putative ion channel protein
wy_01865	*amt_1*	1950267	1951943	-	1677	Ammonia channel precursor
wy_01866	-	1952117	1952425	-	309	hypothetical protein
wy_01867	*glgX*	1952569	1954686	+	2118	Glycogen debranching enzyme
wy_01868	*treY*	1954683	1957061	+	2379	Maltooligosyl trehalose synthase
wy_01869	-	1957174	1957986	+	813	Putative short-chain type dehydrogenase/reductase/MSMEI_5872
wy_01870	*treZ*	1958052	1959812	+	1761	Malto-oligosyltrehalose trehalohydrolase
wy_01871	-	1959793	1960530	-	738	hypothetical protein
wy_01872	-	1960545	1960697	-	153	hypothetical protein
wy_01873	-	1960702	1961505	-	804	Sortase family protein
wy_01874	-	1961539	1961679	-	141	hypothetical protein
wy_01875	-	1961829	1966559	-	4731	hypothetical protein
wy_01876	-	1967081	1971841	-	4761	Translocon-associated protein beta (TRAPB)
wy_01877	*qacA_2*	1972292	1973806	-	1515	Antiseptic resistance protein

wy_01878	*ttgW_2*	1973927	1974493	+	567	putative HTH-type transcriptional regulator TtgW
wy_01879	*ilvA*	1974486	1975838	-	1353	L-threonine dehydratase biosynthetic IlvA
wy_01880	*poxB*	1975912	1977687	-	1776	Pyruvate dehydrogenase [ubiquinone]
wy_01881	-	1977783	1978220	-	438	Putative nitroreductase/MT1609
wy_01882	-	1978384	1978629	+	246	hypothetical protein
wy_01883	-	1978750	1979637	+	888	hypothetical protein
wy_01884	*desA3_1*	1979684	1980079	-	396	Stearoyl-CoA 9-desaturase
wy_01885	*desA3_2*	1980031	1980879	-	849	Stearoyl-CoA 9-desaturase
wy_01886	-	1980939	1981493	-	555	hypothetical protein
wy_01887	*dnaE*	1981653	1985189	-	3537	DNA polymerase III subunit alpha
wy_01888	-	1985234	1986166	-	933	EamA-like transporter family protein
wy_01889	*nlhH_5*	1986240	1987265	+	1026	Carboxylesterase NlhH
wy_01890	-	1987273	1987863	-	591	Secreted chorismate mutase precursor
wy_01891	-	1987956	1988630	-	675	hypothetical protein
wy_01892	*rluD*	1988632	1989540	-	909	Ribosomal large subunit pseudouridine synthase D
wy_01893	*lspA*	1989555	1990187	-	633	Lipoprotein signal peptidase
wy_01894	-	1990250	1991200	+	951	hypothetical protein
wy_01895	-	1991188	1991946	+	759	hypothetical protein
wy_01896	-	1992030	1992551	-	522	hypothetical protein
wy_01897	*dinB_1*	1992659	1994002	-	1344	DNA polymerase IV
wy_01898	*ileS*	1994066	1997233	-	3168	Isoleucine--tRNA ligase
wy_01899	-	1997482	1997736	-	255	hypothetical protein
wy_01900	-	1997780	1998637	-	858	Cell wall synthesis protein Wag31
wy_01901	-	1998752	1999054	-	303	YGGT family protein
wy_01902	*sepF*	1999172	1999759	-	588	Cell division protein SepF
wy_01903	-	1999857	2000585	-	729	hypothetical protein

wy_01904	*yfiH*	2000608	2001333	-	726	Laccase domain protein YfiH
wy_01905	*ftsZ*	2001345	2002574	-	1230	Cell division protein FtsZ
wy_01906	*ftsQ*	2002802	2003575	-	774	Cell division protein FtsQ
wy_01907	*murC*	2003635	2005131	-	1497	UDP-N-acetylmuramate--L-alanine ligase
wy_01908	*murG*	2005128	2006282	-	1155	UDP-N-acetylglucosamine--N-acetylmuramyl-(pentapeptide) pyrophosphoryl-undecaprenol N-acetylglucosamine transferase
wy_01909	*ftsW_1*	2006279	2007775	-	1497	Lipid II flippase FtsW
wy_01910	*murD*	2007772	2009283	-	1512	UDP-N-acetylmuramoylalanine--D-glutamate ligase
wy_01911	*mraY*	2009287	2010381	-	1095	Phospho-N-acetylmuramoyl-pentapeptide-transferase
wy_01912	*murF*	2010378	2011898	-	1521	UDP-N-acetylmuramoyl-tripeptide--D-alanyl-D- alanine ligase
wy_01913	*murE_2*	2011895	2013538	-	1644	UDP-N-acetylmuramoyl-L-alanyl-D-glutamate--2, 6-diaminopimelate ligase
wy_01914	*pbpB*	2013716	2015476	-	1761	Penicillin-binding protein PbpB
wy_01915	-	2015590	2016282	-	693	hypothetical protein
wy_01916	*rsmH*	2016286	2017290	-	1005	Ribosomal RNA small subunit methyltransferase H
wy_01917	-	2017479	2017910	-	432	cell division protein MraZ
wy_01918	-	2018374	2018814	-	441	hypothetical protein
wy_01919	-	2019063	2019578	-	516	hypothetical protein
wy_01920	-	2019751	2020371	+	621	Acetyltransferase (GNAT) family protein
wy_01921	*crtI_2*	2020368	2022005	+	1638	Phytoene desaturase (lycopene-forming)
wy_01922	-	2022002	2022802	+	801	hypothetical protein
wy_01923	-	2022826	2023569	+	744	hypothetical protein
wy_01924	*crtQ_2*	2023583	2024752	+	1170	4,4'-diaponeurosporenoate glycosyltransferase
wy_01925	*metF*	2024756	2025736	-	981	5,10-methylenetetetrahydrofolate reductase
wy_01926	-	2025963	2026997	+	1035	(2E,6E)-farnesyl diphosphate synthase
wy_01927	*carA2_2*	2027012	2028589	+	1578	zeta-carotene-forming phytoene desaturase
wy_01928	-	2028675	2030300	+	1626	hypothetical protein

wy_01929	*crtB_2*	2030297	2031259	+	963	All-trans-phytoene synthase
wy_01930	-	2031290	2031631	-	342	DNA-binding protein
wy_01931	*pknL*	2031802	2033796	+	1995	Serine/threonine-protein kinase PknL
wy_01932	*aroG_2*	2033870	2035258	-	1389	Phospho-2-dehydro-3-deoxyheptonate aldolase AroG
wy_01933	-	2035346	2035837	-	492	3'-5' exoribonuclease/MT2234.1
wy_01934	-	2035934	2037241	+	1308	Polyprenol-phosphate-mannose-dependent alpha-(1-2)-phosphatidylinositol mannoside mannosyltransferase
wy_01935	-	2037333	2037524	+	192	hypothetical protein
wy_01936	-	2038138	2038794	-	657	vancomycin high temperature exclusion protein
wy_01937	*plsC_2*	2038802	2039530	-	729	1-acyl-sn-glycerol-3-phosphate acyltransferase
wy_01938	*glkA*	2039645	2040631	-	987	Glucokinase
wy_01939	-	2040666	2041052	-	387	hypothetical protein
wy_01940	*arsA_1*	2041040	2042116	-	1077	Arsenical pump-driving ATPase
wy_01941	-	2042275	2042712	-	438	Polyketide cyclase / dehydrase and lipid transport
wy_01942	-	2042798	2043190	-	393	hypothetical protein
wy_01943	-	2043355	2045166	+	1812	Long-chain-fatty-acid--CoA ligase FadD15
wy_01944	*pimB_1*	2045248	2046375	-	1128	GDP-mannose-dependent alpha-(1-6)-phosphatidylinositol monomannoside mannosyltransferase
wy_01945	-	2046419	2047705	-	1287	hypothetical protein
wy_01946	-	2047886	2048992	-	1107	putative endopeptidase precursor
wy_01947	-	2049179	2049787	-	609	putative endopeptidase precursor
wy_01948	*polC*	2050449	2052182	+	1734	DNA polymerase III PolC-type
wy_01949	-	2052192	2052467	+	276	AsnC family protein
wy_01950	*trpD*	2052482	2053573	-	1092	Anthranilate phosphoribosyltransferase
wy_01951	-	2053653	2054000	-	348	hypothetical protein
wy_01952	*ctaE*	2054292	2054834	+	543	Cytochrome c oxidase subunit 3
wy_01953	*qcrC*	2054934	2055761	+	828	Menaquinol-cytochrome c reductase cytochrome c subunit

wy_01954	*aioB*	2055758	2056894	+	1137	Arsenite oxidase subunit AioB precursor
wy_01955	*petB*	2056891	2058543	+	1653	Cytochrome b6
wy_01956	-	2058683	2059087	+	405	Carotenoid cleavage oxygenase
wy_01957	-	2059098	2060135	+	1038	Carotenoid cleavage oxygenase
wy_01958	*ctaF*	2060209	2060625	-	417	Cytochrome c oxidase polypeptide 4
wy_01959	*ctaC*	2060661	2061578	-	918	Cytochrome c oxidase subunit 2 precursor
wy_01960	*asnB_1*	2062027	2063952	+	1926	Putative asparagine synthetase [glutamine-hydrolyzing]
wy_01961	*adoK*	2064022	2064996	-	975	Adenosine kinase
wy_01962	*erpA*	2065124	2065474	-	351	Iron-sulfur cluster insertion protein ErpA
wy_01963	*garK*	2065534	2066622	-	1089	Glycerate 2-kinase
wy_01964	-	2066731	2067411	+	681	hypothetical protein
wy_01965	*cobU*	2067482	2068072	+	591	Bifunctional adenosylcobalamin biosynthesis protein CobU
wy_01966	*cobT*	2068074	2069183	+	1110	Nicotinate-nucleotide--dimethylbenzimidazole phosphoribosyltransferase
wy_01967	*cobS*	2069186	2069947	+	762	Cobalamin synthase
wy_01968	*ilvE_1*	2070012	2071115	-	1104	Branched-chain-amino-acid aminotransferase
wy_01969	*gcvT_1*	2071169	2072284	-	1116	Aminomethyltransferase
wy_01970	*pepA*	2072392	2073912	+	1521	Cytosol aminopeptidase
wy_01971	-	2073981	2074322	-	342	hypothetical protein
wy_01972	*dlaT*	2074628	2076388	+	1761	Dihydrolipoyllysine-residue acetyltransferase component of pyruvate dehydrogenase complex
wy_01973	-	2076497	2077390	+	894	Epimerase family protein
wy_01974	*lipB*	2077402	2078175	+	774	Octanoyltransferase
wy_01975	*lipA*	2078228	2079244	+	1017	Lipoyl synthase
wy_01976	-	2079305	2080060	+	756	hypothetical protein
wy_01977	-	2080156	2080677	-	522	RDD family protein
wy_01978	*glnA_1*	2080881	2082317	+	1437	Glutamine synthetase 1

wy_01979	*fabH_1*	2082498	2083535	+	1038	3-oxoacyl-[acyl-carrier-protein] synthase 3
wy_01980	-	2083612	2084112	-	501	putative endopeptidase precursor
wy_01981	-	2084501	2084803	-	303	hypothetical protein
wy_01982	*msuD_4*	2084919	2086028	-	1110	Methanesulfonate monooxygenase
wy_01983	*thiM*	2086025	2086873	-	849	Hydroxyethylthiazole kinase
wy_01984	*sseA_1*	2086933	2087835	-	903	3-mercaptopyruvate sulfurtransferase
wy_01985	*pdhR_1*	2087955	2088641	+	687	Pyruvate dehydrogenase complex repressor
wy_01986	*isdE*	2088903	2090003	+	1101	High-affinity heme uptake system protein IsdE precursor
wy_01987	-	2090044	2091045	+	1002	putative ABC transporter permease protein
wy_01988	-	2091038	2091823	+	786	putative ABC transporter ATP-binding protein
wy_01989	-	2091820	2092455	+	636	hypothetical protein
wy_01990	-	2092452	2093132	+	681	hypothetical protein
wy_01991	-	2093213	2093725	-	513	hypothetical protein
wy_01992	*glnE*	2093728	2096742	-	3015	Glutamate-ammonia-ligase adenylyltransferase
wy_01993	*glnA_2*	2096840	2098180	-	1341	putative glutamine synthetase 2
wy_01994	*caeA_2*	2098248	2099978	-	1731	Carboxylesterase A precursor
wy_01995	*panB*	2100009	2100848	+	840	3-methyl-2-oxobutanoate hydroxymethyltransferase
wy_01996	-	2100973	2101425	+	453	hypothetical protein
wy_01997	*pip_1*	2101429	2102385	+	957	Proline iminopeptidase
wy_01998	-	2102464	2103327	+	864	CHAD domain protein
wy_01999	-	2103403	2103630	-	228	hypothetical protein
wy_02000	-	2103795	2103902	+	108	hypothetical protein
wy_02001	-	2104466	2104993	+	528	hypothetical protein
wy_02002	*arsB*	2105017	2106252	+	1236	Arsenical pump membrane protein
wy_02003	*dctA_1*	2106259	2107644	-	1386	C4-dicarboxylate transport protein
wy_02004	-	2107959	2108414	+	456	hypothetical protein

wy_02005	*pspB_2*	2108392	2109525	-	1134	Putative phosphoserine phosphatase 2
wy_02006	-	2109522	2110259	-	738	Putative zinc ribbon domain protein
wy_02007	-	2110383	2111531	-	1149	Putative GTP cyclohydrolase 1 type 2
wy_02008	*cobD*	2111528	2112574	-	1047	Threonine-phosphate decarboxylase
wy_02009	-	2112638	2113315	+	678	5'-nucleotidase
wy_02010	*ptpA_1*	2113323	2113778	+	456	putative low molecular weight protein-tyrosine-phosphatase
wy_02011	-	2113799	2114773	+	975	SURF1 family protein
wy_02012	-	2114789	2115064	-	276	Antibiotic biosynthesis monooxygenase
wy_02013	-	2115061	2115939	-	879	cobalamin biosynthesis protein
wy_02014	*speE*	2116094	2117659	-	1566	Spermidine synthase
wy_02015	-	2117656	2118126	-	471	hypothetical protein
wy_02016	-	2118159	2118551	-	393	hypothetical protein
wy_02017	-	2118560	2119063	-	504	hypothetical protein
wy_02018	-	2119060	2119656	-	597	hypothetical protein
wy_02019	-	2119791	2120537	+	747	VIT family protein
wy_02020	-	2120514	2121059	-	546	Dual specificity phosphatase, catalytic domain
wy_02022	-	2121275	2121739	-	465	Putative peroxiredoxin/MT2298
wy_02023	-	2121825	2122247	-	423	hypothetical protein
wy_02024	*aceE*	2122587	2125493	+	2907	Pyruvate dehydrogenase E1 component
wy_02025	-	2125592	2126941	+	1350	carbohydrate diacid transcriptional activator CdaR
wy_02026	*fabD*	2127114	2127986	+	873	Malonyl CoA-acyl carrier protein transacylase
wy_02027	*acpM*	2128077	2128388	+	312	Meromycolate extension acyl carrier protein
wy_02028	*kasA*	2128396	2129643	+	1248	3-oxoacyl-[acyl-carrier-protein] synthase 1
wy_02029	*accD6*	2129740	2131173	+	1434	putative propionyl-CoA carboxylase beta chain 6
wy_02030	-	2131399	2131908	-	510	hypothetical protein
wy_02031	-	2132048	2132869	-	822	putative periplasmic esterase

wy_02032	*adhD_1*	2132904	2134031	-	1128	Putative alcohol dehydrogenase D
wy_02033	-	2134227	2135495	-	1269	Putative diacylglycerol O-acyltransferase/MT1468
wy_02034	*yflN_2*	2135637	2136362	-	726	putative metallo-hydrolase YflN
wy_02036	-	2137184	2137870	+	687	DNA polymerase III subunit epsilon
wy_02038	-	2138148	2138396	+	249	hypothetical protein
wy_02039	*dnaG*	2138416	2140347	-	1932	DNA primase
wy_02040	*dgt*	2140426	2141715	-	1290	Deoxyguanosinetriphosphate triphosphohydrolase
wy_02041	-	2141712	2142431	-	720	hypothetical protein
wy_02042	-	2142475	2144526	+	2052	hypothetical protein
wy_02043	*glyQS*	2144604	2145995	-	1392	Glycine--tRNA ligase
wy_02044	*smtB*	2146107	2146559	+	453	HTH-type transcriptional repressor SmtB
wy_02045	*zur*	2146556	2147002	+	447	Zinc uptake regulation protein
wy_02046	-	2147227	2147790	+	564	hypothetical protein
wy_02047	-	2147821	2148381	+	561	hypothetical protein
wy_02048	*sipW_3*	2148378	2148926	+	549	Signal peptidase I W
wy_02049	-	2148930	2149541	+	612	Camelysin metallo-endopeptidase
wy_02050	-	2149538	2151466	+	1929	hypothetical protein
wy_02051	-	2151590	2151994	-	405	hypothetical protein
wy_02052	-	2151991	2152803	-	813	Trans,polycis-polyprenyl diphosphate synthase ((2Z,6E)-farnesyl diphosphate specific)
wy_02053	*recO*	2152760	2153575	-	816	DNA repair protein RecO
wy_02054	*aam_2*	2153698	2155125	+	1428	Acylamidase
wy_02055	-	2155129	2155698	+	570	putative DNA-binding transcriptional regulator
wy_02056	-	2155733	2156263	-	531	hypothetical protein
wy_02057	*era*	2156419	2157336	-	918	GTPase Era
wy_02058	*corC_2*	2157338	2158660	-	1323	Magnesium and cobalt efflux protein CorC
wy_02059	*ybeY*	2158685	2159227	-	543	Endoribonuclease YbeY

wy_02060	*ybeZ_2*	2159286	2160287	-	1002	PhoH-like protein
wy_02061	*ttgW_3*	2160475	2161131	-	657	putative HTH-type transcriptional regulator TtgW
wy_02062	*qacA_3*	2161267	2162877	+	1611	Antiseptic resistance protein
wy_02063	-	2162868	2163623	-	756	Alpha/beta hydrolase family protein
wy_02064	*rsmE*	2163716	2164477	-	762	Ribosomal RNA small subunit methyltransferase E
wy_02065	*dnaJ_1*	2164493	2165644	-	1152	Chaperone protein DnaJ
wy_02066	*hrcA*	2165744	2166787	-	1044	Heat-inducible transcription repressor HrcA
wy_02067	-	2167015	2167323	+	309	hypothetical protein
wy_02068	*hemN*	2167336	2168487	-	1152	Oxygen-independent coproporphyrinogen-III oxidase 1
wy_02069	*sir*	2168917	2170626	+	1710	Sulfite reductase [ferredoxin]
wy_02070	*cysH*	2170623	2171366	+	744	Phosphoadenosine phosphosulfate reductase
wy_02071	*cysD_2*	2171363	2172304	+	942	Sulfate adenylyltransferase subunit 2
wy_02072	*cysNC_2*	2172307	2173602	+	1296	Bifunctional enzyme CysN/CysC
wy_02073	*sirB*	2173676	2174356	+	681	Sirohydrochlorin ferrochelatase
wy_02074	-	2174373	2175158	+	786	Pentapeptide repeats (8 copies)
wy_02075	-	2175155	2175382	-	228	hypothetical protein
wy_02076	*ntaB*	2175488	2176003	+	516	FMN reductase (NADH) NtaB
wy_02077	*ampH*	2176091	2177368	+	1278	D-alanyl-D-alanine-carboxypeptidase/endopeptidase AmpH precursor
wy_02078	*cysA*	2177437	2178384	-	948	Sulfate/thiosulfate import ATP-binding protein CysA
wy_02079	*cysW*	2178381	2179187	-	807	Sulfate transport system permease protein CysW
wy_02080	*cysT*	2179184	2180065	-	882	Sulfate transport system permease protein CysT
wy_02081	*sbpA_1*	2180065	2181096	-	1032	Sulfate-binding protein precursor
wy_02082	-	2181541	2181765	-	225	hypothetical protein
wy_02083	*sbpA_2*	2181860	2182888	-	1029	Sulfate-binding protein precursor
wy_02084	-	2183314	2185281	+	1968	Trehalase
wy_0208	-	218532	218570	+	378	Glyoxalase-like domain protein

5		8	5			
wy_02086	-	2185716	2186648	-	933	Nitronate monooxygenase
wy_02087	*lepA*	2186676	2188538	-	1863	Elongation factor 4
wy_02088	-	2189096	2189647	+	552	PemK-like protein
wy_02089	*aqpZ*	2189709	2190479	-	771	Aquaporin Z
wy_02090	*rpsT*	2190807	2191067	+	261	30S ribosomal protein S20
wy_02091	-	2191225	2192202	-	978	hypothetical protein
wy_02092	-	2192199	2194577	-	2379	ComEC family competence protein
wy_02093	*comEA*	2194574	2195422	-	849	ComE operon protein 1
wy_02094	-	2195518	2196396	-	879	DegV domain-containing protein/cg2579
wy_02095	-	2196401	2197156	-	756	hypothetical protein
wy_02096	*gpgP_1*	2197197	2197928	-	732	Glucosyl-3-phosphoglycerate phosphatase
wy_02097	*rsfS*	2197925	2198341	-	417	Ribosomal silencing factor RsfS
wy_02098	*nadD*	2198342	2199082	-	741	putative nicotinate-nucleotide adenylyltransferase
wy_02099	-	2199119	2200567	-	1449	VWA domain containing CoxE-like protein
wy_02100	-	2200581	2201462	-	882	ATPase family associated with various cellular activities (AAA)
wy_02101	*proA*	2201482	2202765	-	1284	Gamma-glutamyl phosphate reductase
wy_02102	*cycA*	2202837	2204222	+	1386	D-serine/D-alanine/glycine transporter
wy_02103	*rbsK_2*	2204278	2205135	+	858	Ribokinase
wy_02104	*yjaB*	2205137	2205640	+	504	putative N-acetyltransferase YjaB
wy_02105	*recQ_1*	2205693	2207801	+	2109	ATP-dependent DNA helicase RecQ
wy_02106	*ectA*	2207934	2208461	+	528	L-2,4-diaminobutyric acid acetyltransferase
wy_02107	*ectB*	2208475	2209734	+	1260	Diaminobutyrate--2-oxoglutarate transaminase
wy_02108	*ectC*	2209778	2210194	+	417	L-ectoine synthase
wy_02109	-	2210265	2212172	-	1908	ABC-2 family transporter protein
wy_02110	-	2212313	2212786	-	474	Glyoxalase-like domain protein
wy_0211	*cobB_3*	221286	221367	+	807	NAD-dependent protein deacetylase

1		7	3			
wy_02112	*proB*	2213682	2214782	-	1101	Glutamate 5-kinase
wy_02113	*obg*	2214779	2216239	-	1461	GTPase Obg
wy_02114	*rpmA*	2216432	2216695	-	264	50S ribosomal protein L27
wy_02115	*rplU*	2216747	2217058	-	312	50S ribosomal protein L21
wy_02116	*rng*	2217321	2220599	-	3279	Ribonuclease G
wy_02117	*ndkA*	2221056	2221475	-	420	Nucleoside diphosphate kinase
wy_02118	-	2221494	2221895	-	402	hypothetical protein
wy_02119	*folC*	2221892	2223322	-	1431	Bifunctional protein FolC
wy_02120	*valS*	2223319	2225883	-	2565	Valine--tRNA ligase
wy_02121	-	2226134	2226745	-	612	hypothetical protein
wy_02122	*ptpA_2*	2226882	2228921	-	2040	Prolyl tripeptidyl peptidase precursor
wy_02123	-	2228958	2229560	-	603	Acetyltransferase (GNAT) family protein
wy_02124	*yfiY_2*	2229583	2230596	-	1014	putative siderophore-binding lipoprotein YfiY precursor
wy_02125	-	2230610	2230993	+	384	hypothetical protein
wy_02126	-	2230994	2231818	+	825	formate dehydrogenase accessory protein
wy_02127	-	2231900	2232364	+	465	hypothetical protein
wy_02128	-	2232361	2233566	-	1206	putative inactive lipase/MT1628
wy_02129	-	2233634	2234269	+	636	Bacterial regulatory proteins, tetR family
wy_02130	*clpX*	2234330	2235607	-	1278	ATP-dependent Clp protease ATP-binding subunit ClpX
wy_02131	*clpP2_1*	2235896	2236567	-	672	ATP-dependent Clp protease proteolytic subunit 2
wy_02132	*clpP2_2*	2236617	2237225	-	609	ATP-dependent Clp protease proteolytic subunit 2
wy_02133	*tig*	2237356	2238726	-	1371	Trigger factor
wy_02136	-	2239295	2240428	+	1134	Nitronate monooxygenase
wy_02137	*dinB_2*	2240446	2241702	-	1257	DNA polymerase IV
wy_02138	-	2241807	2242601	+	795	hypothetical protein
wy_0213	*nei1_1*	224259	224339	-	798	Endonuclease 8 1

9		8	5			
wy_02140	*rpiB*	2243404	2243886	-	483	Ribose-5-phosphate isomerase B
wy_02141	-	2243965	2244603	-	639	DSBA-like thioredoxin domain protein
wy_02142	*thcD_1*	2244768	2246009	+	1242	Rhodocoxin reductase
wy_02143	*pepN_1*	2246143	2248704	+	2562	Aminopeptidase N
wy_02144	-	2248800	2249276	-	477	hypothetical protein
wy_02145	-	2249263	2249511	-	249	hypothetical protein
wy_02146	-	2249573	2250238	-	666	HNH endonuclease
wy_02147	*glbO*	2250393	2250821	+	429	Group 2 truncated hemoglobin GlbO
wy_02148	*malL*	2250920	2252524	+	1605	Oligo-1,6-glucosidase 1
wy_02149	*kstD_3*	2252656	2254347	+	1692	3-oxosteroid 1-dehydrogenase
wy_02150	-	2254374	2255147	+	774	3-alpha-(or 20-beta)-hydroxysteroid dehydrogenase
wy_02151	-	2255154	2256290	+	1137	hypothetical protein
wy_02152	*kipR_1*	2256383	2257096	+	714	HTH-type transcriptional regulator KipR
wy_02153	*mhpA_1*	2257303	2259027	+	1725	3-(3-hydroxy-phenyl)propionate/3-hydroxycinnamic acid hydroxylase
wy_02154	*xylF_1*	2259039	2259902	+	864	2-hydroxymuconate semialdehyde hydrolase
wy_02155	*mhpB_1*	2259919	2260860	+	942	2,3-dihydroxyphenylpropionate/2, 3-dihydroxicinnamic acid 1,2-dioxygenase
wy_02156	-	2260931	2262151	+	1221	Cytochrome P450 130
wy_02157	-	2262154	2262735	+	582	DNA-binding transcriptional repressor AcrR
wy_02158	-	2262814	2263341	+	528	hypothetical protein
wy_02159	-	2263349	2264029	-	681	hypothetical protein
wy_02160	-	2264022	2264489	-	468	hypothetical protein
wy_02161	*gdh_1*	2264486	2269351	-	4866	NAD-specific glutamate dehydrogenase
wy_02162	-	2269473	2269895	-	423	acyl-CoA thioesterase YbgC
wy_02163	-	2269900	2271573	-	1674	putative ABC transporter ATP-binding protein/MT2552
wy_02164	-	2272027	2273169	-	1143	Secretory lipase

wy_02165	*ssb_1*	2273269	2273742	-	474	Single-stranded DNA-binding protein
wy_02166	*yebZ*	2273940	2275970	-	2031	Inner membrane protein YebZ
wy_02167	-	2275951	2277252	-	1302	VWA domain containing CoxE-like protein
wy_02168	-	2277249	2277914	-	666	Carbon monoxide dehydrogenase subunit G (CoxG)
wy_02169	-	2277925	2278824	-	900	AAA domain (dynein-related subfamily)
wy_02170	*nboR_1*	2278821	2279411	-	591	Nicotine blue oxidoreductase
wy_02171	-	2279402	2280139	-	738	hypothetical protein
wy_02172	*cutL*	2280302	2282698	-	2397	Carbon monoxide dehydrogenase large chain
wy_02173	*cutS_1*	2282695	2283192	-	498	Carbon monoxide dehydrogenase small chain
wy_02174	*cutM*	2283194	2284078	-	885	Carbon monoxide dehydrogenase medium chain
wy_02175	*pucA_1*	2284185	2285309	-	1125	putative xanthine dehydrogenase subunit A
wy_02176	*cysL*	2285476	2286354	+	879	HTH-type transcriptional regulator CysL
wy_02178	-	2286510	2287076	-	567	Lipase (class 2)
wy_02179	-	2287101	2287472	-	372	hypothetical protein
wy_02180	-	2287608	2289020	-	1413	hypothetical protein
wy_02181	*gdhA*	2289375	2290721	+	1347	NAD(P)-specific glutamate dehydrogenase
wy_02182	-	2290790	2291002	-	213	hypothetical protein
wy_02183	*desA3_3*	2291017	2292201	-	1185	Stearoyl-CoA 9-desaturase
wy_02184	-	2292235	2293320	-	1086	Stearoyl-CoA 9-desaturase electron transfer partner
wy_02185	*fabR*	2293454	2294101	+	648	HTH-type transcriptional repressor FabR
wy_02186	*dhaA_3*	2294123	2294968	+	846	Haloalkane dehalogenase
wy_02187	*yxaF*	2294976	2295545	-	570	putative HTH-type transcriptional regulator YxaF
wy_02188	-	2295635	2296462	+	828	hypothetical protein
wy_02189	-	2296523	2297869	-	1347	Vacuole effluxer Atg22 like protein
wy_02190	*panF*	2298447	2300135	+	1689	Sodium/pantothenate symporter
wy_02191	-	2300183	2300314	+	132	hypothetical protein

wy_02192	-	2300415	2300969	+	555	hypothetical protein
wy_02193	*fadH_1*	2300989	2303019	+	2031	2,4-dienoyl-CoA reductase [NADPH]
wy_02194	*isfD*	2303065	2303868	+	804	Sulfoacetaldehyde reductase
wy_02195	*lysA_2*	2304009	2305352	+	1344	Diaminopimelate decarboxylase
wy_02196	-	2305450	2306568	+	1119	NDMA-dependent alcohol dehydrogenase
wy_02197	-	2306588	2308195	-	1608	AAA-like domain protein
wy_02198	*orn*	2308312	2308878	+	567	Oligoribonuclease
wy_02200	-	2309438	2309554	+	117	hypothetical protein
wy_02201	-	2309687	2309914	-	228	hypothetical protein
wy_02202	*lppS_1*	2309967	2311145	-	1179	Putative L,D-transpeptidase LppS precursor
wy_02205	-	2311810	2312031	-	222	hypothetical protein
wy_02206	*bcp_2*	2312176	2312646	+	471	Putative peroxiredoxin/MT2597
wy_02207	-	2312658	2314028	-	1371	N-formyl-4-amino-5-aminomethyl-2-methylpyrimidine deformylase
wy_02208	-	2314106	2315812	-	1707	hypothetical protein
wy_02209	*tetR_2*	2315814	2316575	-	762	Tetracycline repressor protein class E
wy_02210	*drrA_6*	2316686	2317639	+	954	Daunorubicin/doxorubicin resistance ATP-binding protein DrrA
wy_02211	*drrB_3*	2317636	2318424	+	789	Daunorubicin/doxorubicin resistance ABC transporter permease protein DrrB
wy_02212	-	2318532	2319185	+	654	Bacterial regulatory proteins, tetR family
wy_02213	*acpS*	2319296	2319709	-	414	Holo-[acyl-carrier-protein] synthase
wy_02214	*fabF*	2319709	2329008	-	9300	3-oxoacyl-[acyl-carrier-protein] synthase 2
wy_02215	-	2329453	2330118	+	666	hypothetical protein
wy_02217	-	2330644	2331351	+	708	hypothetical protein
wy_02218	*yidC*	2331373	2332254	-	882	Membrane protein insertase YidC
wy_02219	-	2332378	2332698	-	321	hypothetical protein
wy_02220	-	2332851	2333837	+	987	SnoaL-like polyketide cyclase
wy_0222	-	233400	233510	-	1095	Amidohydrolase family protein

1		9	3			
wy_02222	*ffh*	2335110	2336690	-	1581	Signal recognition particle protein
wy_02223	*glnD*	2336815	2339352	-	2538	Bifunctional uridylyltransferase/uridylyl-removing enzyme
wy_02224	*glnB*	2339342	2339680	-	339	Nitrogen regulatory protein P-II
wy_02225	*amt_2*	2339684	2341057	-	1374	Ammonia channel precursor
wy_02226	*ftsY*	2341294	2342403	-	1110	Signal recognition particle receptor FtsY
wy_02227	*gpx1*	2342906	2343439	-	534	Hydroperoxy fatty acid reductase gpx1
wy_02228	*smc_3*	2343507	2347109	-	3603	Chromosome partition protein Smc
wy_02229	*acyP*	2347132	2347422	-	291	Acylphosphatase
wy_02230	-	2347422	2348519	-	1098	hypothetical protein
wy_02231	-	2349270	2350571	+	1302	hypothetical protein
wy_02232	-	2350625	2351059	-	435	OsmC-like protein
wy_02233	*alsT*	2351052	2352482	-	1431	Amino-acid carrier protein AlsT
wy_02234	*kdpE*	2352553	2353233	-	681	KDP operon transcriptional regulatory protein KdpE
wy_02235	*kdpD*	2353292	2355805	-	2514	Sensor protein KdpD
wy_02236	*kdpC*	2355783	2356355	-	573	Potassium-transporting ATPase C chain
wy_02237	*kdpB*	2356431	2358569	-	2139	Potassium-transporting ATPase B chain
wy_02238	*kdpA*	2358566	2360266	-	1701	Potassium-transporting ATPase A chain
wy_02239	-	2360287	2360385	-	99	F subunit of K+-transporting ATPase (Potass_KdpF)
wy_02240	-	2360382	2360474	-	93	hypothetical protein
wy_02241	-	2360612	2361271	-	660	Pyridoxamine 5'-phosphate oxidase
wy_02242	*gabR*	2361350	2362822	+	1473	HTH-type transcriptional regulatory protein GabR
wy_02243	*fpg1*	2362758	2363624	-	867	Formamidopyrimidine-DNA glycosylase 1
wy_02244	*rnc*	2363649	2364344	-	696	Ribonuclease 3
wy_02245	*rpmF*	2364436	2364618	-	183	50S ribosomal protein L32
wy_02246	-	2364621	2365097	-	477	hypothetical protein
wy_0224	-	236534	236609	-	756	Cell wall synthesis protein Wag31

7		4	9			
wy_02248	*coaD*	2366216	2366710	-	495	Phosphopantetheine adenylyltransferase
wy_02249	*rsmD*	2366707	2367234	-	528	Ribosomal RNA small subunit methyltransferase D
wy_02250	*cfiB*	2367301	2370708	-	3408	2-oxoglutarate carboxylase small subunit
wy_02251	*recG*	2370918	2373173	-	2256	ATP-dependent DNA helicase RecG
wy_02252	-	2373214	2374863	-	1650	DAK2 domain protein
wy_02253	*rpmB_2*	2375069	2375368	+	300	50S ribosomal protein L28
wy_02254	-	2375524	2376327	+	804	4-chlorobenzoyl coenzyme A dehalogenase
wy_02255	-	2376355	2376564	-	210	hypothetical protein
wy_02256	*ung*	2376590	2377267	-	678	Uracil-DNA glycosylase
wy_02257	*dapH*	2377267	2377788	-	522	2,3,4,5-tetrahydropyridine-2,6-dicarboxylate N-acetyltransferase
wy_02258	*thiL*	2377798	2378763	-	966	Thiamine-monophosphate kinase
wy_02259	-	2378941	2379246	+	306	hypothetical protein
wy_02260	-	2379248	2379835	+	588	hypothetical protein
wy_02261	*ddl*	2379884	2380990	-	1107	D-alanine--D-alanine ligase
wy_02262	*gpsA_1*	2381072	2382070	-	999	Glycerol-3-phosphate dehydrogenase [NAD(P)+]
wy_02263	*cofC*	2382224	2382940	+	717	2-phospho-L-lactate guanylyltransferase
wy_02264	*ppk*	2382998	2385217	+	2220	Polyphosphate kinase
wy_02265	*mutT4_1*	2385282	2386214	+	933	Putative mutator protein MutT4
wy_02266	-	2386302	2387030	-	729	hypothetical protein
wy_02267	*leuD*	2387187	2387798	-	612	3-isopropylmalate dehydratase small subunit
wy_02268	*leuC*	2387825	2389249	-	1425	3-isopropylmalate dehydratase large subunit
wy_02269	*kipR_2*	2389379	2390080	+	702	HTH-type transcriptional regulator KipR
wy_02270	-	2390172	2390669	+	498	Pyridoxamine 5'-phosphate oxidase
wy_02271	-	2390766	2390933	+	168	hypothetical protein
wy_02275	*gltX_1*	2391609	2393081	-	1473	Glutamate--tRNA ligase
wy_0227	-	239318	239391	-	732	Ureidoglycolate lyase

6		2	3			
wy_02277	*exuT_1*	2394083	2395285	+	1203	Hexuronate transporter
wy_02278	*leuB*	2395360	2396373	-	1014	3-isopropylmalate dehydrogenase
wy_02279	*serA_1*	2396484	2398076	-	1593	D-3-phosphoglycerate dehydrogenase
wy_02280	*ilvC*	2398340	2399341	-	1002	Ketol-acid reductoisomerase
wy_02281	*ilvH*	2399418	2399921	-	504	Putative acetolactate synthase small subunit
wy_02282	*ilvB1*	2399918	2401867	-	1950	Acetolactate synthase large subunit IlvB1
wy_02283	*cfp6*	2402153	2402656	+	504	Low molecular weight protein antigen 6
wy_02284	*ilvD_1*	2402846	2404687	+	1842	Dihydroxy-acid dehydratase
wy_02285	*mhqP*	2404763	2405662	-	900	Putative oxidoreductase MhqP
wy_02286	*yliI_1*	2405780	2406922	+	1143	Soluble aldose sugar dehydrogenase YliI precursor
wy_02287	*kefC_1*	2406926	2408134	-	1209	Glutathione-regulated potassium-efflux system protein KefC
wy_02288	*gatB*	2408146	2409660	-	1515	Aspartyl/glutamyl-tRNA(Asn/Gln) amidotransferase subunit B
wy_02289	*pfkA*	2409677	2410708	-	1032	6-phosphofructokinase
wy_02290	*vraR*	2410790	2411452	-	663	Response regulator protein VraR
wy_02291	*liaS_2*	2411462	2412607	-	1146	Sensor histidine kinase LiaS
wy_02292	-	2412814	2413422	+	609	hypothetical protein
wy_02293	-	2413419	2414768	+	1350	hypothetical protein
wy_02294	-	2414814	2415338	+	525	DinB superfamily protein
wy_02295	*gatA_2*	2415423	2416907	-	1485	Glutamyl-tRNA(Gln) amidotransferase subunit A
wy_02296	*gatC*	2416904	2417203	-	300	Aspartyl/glutamyl-tRNA(Asn/Gln) amidotransferase subunit C
wy_02297	-	2417392	2418042	+	651	hypothetical protein
wy_02298	-	2418048	2418536	-	489	hypothetical protein
wy_02299	*ligA*	2418881	2421019	-	2139	DNA ligase
wy_02300	-	2421071	2422102	-	1032	Cobalamin-independent synthase, Catalytic domain
wy_02301	*mnmA*	2422099	2423217	-	1119	tRNA-specific 2-thiouridylase MnmA

wy_02302	*iscS*	2423218	2424435	-	1218	Cysteine desulfurase
wy_02303	-	2424490	2425332	-	843	2-acyl-glycerophospho-ethanolamine acyltransferase
wy_02304	-	2425329	2426144	-	816	hypothetical protein
wy_02305	*etfA*	2426354	2427310	-	957	Electron transfer flavoprotein subunit alpha
wy_02306	*etfB*	2427356	2428156	-	801	Electron transfer flavoprotein subunit beta
wy_02307	-	2428496	2429212	+	717	putative S-adenosylmethionine-dependent methyltransferase/MSMEI_2290
wy_02308	-	2429209	2430792	+	1584	1,4-alpha-glucan branching enzyme
wy_02309	-	2430792	2432036	+	1245	Glycogen synthase
wy_02310	-	2432060	2432851	-	792	Putative acetyltransferase
wy_02311	-	2432922	2433386	-	465	hypothetical protein
wy_02312	*ald*	2433500	2434624	-	1125	Alanine dehydrogenase
wy_02313	-	2434752	2436059	+	1308	outer membrane biogenesis protein BamB
wy_02314	-	2436063	2437220	-	1158	hypothetical protein
wy_02315	*rebM*	2437238	2438212	-	975	Demethylrebeccamycin-D-glucose O-methyltransferase
wy_02316	-	2438349	2439128	-	780	putative enoyl-CoA hydratase echA17
wy_02317	-	2439149	2439958	-	810	NUDIX domain protein
wy_02318	*ylmA_1*	2440105	2440935	-	831	putative ABC transporter ATP-binding protein YlmA
wy_02319	-	2440995	2441744	-	750	peptidyl-tRNA hydrolase
wy_02320	*serB_1*	2441741	2442967	-	1227	Phosphoserine phosphatase
wy_02321	*ctaD_1*	2443112	2444851	-	1740	Cytochrome c oxidase subunit 1
wy_02322	*yfiY_3*	2445217	2446218	+	1002	putative siderophore-binding lipoprotein YfiY precursor
wy_02323	*yfiY_4*	2446265	2447269	+	1005	putative siderophore-binding lipoprotein YfiY precursor
wy_02324	*nudB*	2447279	2447755	+	477	Dihydroneopterin triphosphate pyrophosphatase
wy_02325	-	2447768	2448154	-	387	Glyoxalase-like domain protein
wy_02326	-	2448289	2448783	+	495	hypothetical protein
wy_02327	*nrdF2*	2448861	2449868	-	1008	Ribonucleoside-diphosphate reductase subunit beta nrdF2

wy_02328	*nrdE*	2449915	2452101	-	2187	Ribonucleoside-diphosphate reductase subunit alpha
wy_02329	-	2452059	2452517	-	459	Putative NrdI-like protein
wy_02330	*nrdH*	2452575	2452808	-	234	Glutaredoxin-like protein NrdH
wy_02331	*viuB_2*	2453364	2454257	+	894	Vibriobactin utilization protein ViuB
wy_02332	*azr_1*	2454336	2454899	-	564	FMN-dependent NADPH-azoreductase
wy_02333	*bm3R1_1*	2454995	2455618	+	624	HTH-type transcriptional repressor Bm3R1
wy_02334	-	2455628	2456437	+	810	heat-inducible protein
wy_02335	-	2456448	2457281	-	834	NmrA-like family protein
wy_02336	*apc4*	2457286	2459241	-	1956	Acetophenone carboxylase delta subunit
wy_02337	*apc3*	2459238	2461340	-	2103	Acetophenone carboxylase gamma subunit
wy_02338	-	2461529	2462209	-	681	Carbon monoxide dehydrogenase subunit G (CoxG)
wy_02339	*cdhA*	2462206	2464710	-	2505	Caffeine dehydrogenase subunit alpha
wy_02340	*cdhC*	2464707	2465351	-	645	Caffeine dehydrogenase subunit gamma
wy_02341	*kdhA*	2465354	2466265	-	912	6-hydroxypseudooxynicotine dehydrogenase complex subunit alpha
wy_02342	*rutG_2*	2466353	2467783	-	1431	Putative pyrimidine permease RutG
wy_02343	*pucR*	2467962	2469599	-	1638	Purine catabolism regulatory protein
wy_02344	*pucA_2*	2469699	2470811	-	1113	putative xanthine dehydrogenase subunit A
wy_02345	*gatA_3*	2470796	2472223	-	1428	Glutamyl-tRNA(Gln) amidotransferase subunit A
wy_02346	-	2472367	2473569	+	1203	Major Facilitator Superfamily protein
wy_02347	*nboR_2*	2473566	2474189	+	624	Nicotine blue oxidoreductase
wy_02348	*mtnN*	2474186	2474749	-	564	Aminodeoxyfutalosine nucleosidase
wy_02349	*nadE*	2474854	2475699	+	846	NH(3)-dependent NAD(+) synthetase
wy_02350	-	2475736	2476506	+	771	hypothetical protein
wy_02351	-	2476514	2476795	-	282	hypothetical protein
wy_02352	*moaA*	2476788	2477867	-	1080	Cyclic pyranopterin monophosphate synthase
wy_02353	*moeA_2*	2477931	2479127	+	1197	Molybdopterin molybdenumtransferase

wy_02354	*moaC2*	2479142	2480137	+	996	Cyclic pyranopterin monophosphate synthase accessory protein 2
wy_02355	*moaE1*	2480130	2480543	+	414	Molybdopterin synthase catalytic subunit 1
wy_02356	*rhlE_2*	2480778	2482280	+	1503	ATP-dependent RNA helicase RhlE
wy_02357	*bluB*	2482297	2482905	-	609	5,6-dimethylbenzimidazole synthase
wy_02358	*yciC_2*	2482972	2484045	-	1074	Putative metal chaperone YciC
wy_02359	-	2484203	2484661	+	459	molybdopterin biosynthesis protein MoeB
wy_02360	-	2484740	2485480	+	741	hypothetical protein
wy_02361	-	2485666	2485962	+	297	Antibiotic biosynthesis monooxygenase
wy_02362	-	2486139	2486267	+	129	hypothetical protein
wy_02364	*pknE*	2486630	2487379	-	750	Serine/threonine-protein kinase PknE
wy_02365	-	2487467	2487940	-	474	Methylamine utilization protein MauE
wy_02366	*mdtH*	2488103	2489437	-	1335	Multidrug resistance protein MdtH
wy_02367	*pgm*	2489473	2491110	-	1638	Phosphoglucomutase
wy_02368	*crcB*	2491180	2491551	-	372	Putative fluoride ion transporter CrcB
wy_02369	-	2491521	2491883	-	363	camphor resistance protein CrcB
wy_02370	-	2491910	2492662	-	753	hypothetical protein
wy_02371	-	2492702	2493331	-	630	Alpha/beta hydrolase family protein
wy_02372	*pip_2*	2493376	2494374	-	999	Proline iminopeptidase
wy_02374	-	2495058	2496263	+	1206	putative inactive lipase/MT1628
wy_02375	-	2496397	2498463	+	2067	Membrane dipeptidase (Peptidase family M19)
wy_02376	*smpB*	2498541	2499014	-	474	SsrA-binding protein
wy_02377	*ftsX*	2499116	2500021	-	906	Cell division protein FtsX
wy_02378	*ftsE*	2500082	2500771	-	690	Cell division ATP-binding protein FtsE
wy_02379	-	2500840	2501328	-	489	hypothetical protein
wy_02380	*ykuT_1*	2501325	2502230	-	906	putative MscS family protein YkuT
wy_02381	*prfB*	2502236	2503339	-	1104	Peptide chain release factor 2

wy_02382	*hisN*	2503458	2504228	+	771	Histidinol-phosphatase
wy_02383	-	2504337	2504663	+	327	hypothetical protein
wy_02384	-	2504635	2506137	-	1503	hypothetical protein
wy_02385	*baeS*	2506227	2507216	-	990	Signal transduction histidine-protein kinase BaeS
wy_02386	*cseB*	2507213	2507902	-	690	Transcriptional regulatory protein CseB
wy_02387	*acdA_4*	2508154	2509557	+	1404	Acyl-CoA dehydrogenase
wy_02388	*acrC_2*	2509591	2510799	+	1209	Acryloyl-CoA reductase (NADH)
wy_02389	-	2511181	2511657	-	477	hypothetical protein
wy_02390	-	2511654	2513138	-	1485	hypothetical protein
wy_02391	-	2513292	2513828	-	537	hypothetical protein
wy_02392	*lcfB_10*	2513956	2515599	+	1644	Long-chain-fatty-acid--CoA ligase
wy_02394	*rpfE_3*	2516296	2516847	+	552	Resuscitation-promoting factor RpfE precursor
wy_02395	-	2516932	2519910	-	2979	hypothetical protein
wy_02396	-	2520024	2520665	+	642	hypothetical protein
wy_02397	*lon*	2520662	2521708	-	1047	Lon protease
wy_02398	-	2521832	2523274	+	1443	hypothetical protein
wy_02399	-	2523271	2523831	-	561	SprT-like family protein
wy_02400	-	2524031	2524924	+	894	hypothetical protein
wy_02401	*ubiB_1*	2524932	2526275	+	1344	putative protein kinase UbiB
wy_02402	-	2526478	2527119	+	642	hypothetical protein
wy_02403	*whiB7_2*	2527199	2527531	-	333	putative transcriptional regulator WhiB7
wy_02404	*uvrD2*	2528024	2530186	-	2163	ATP-dependent DNA helicase UvrD2
wy_02405	-	2530321	2530545	+	225	Putative glutaredoxin.1/MT3292
wy_02406	*nudC*	2530574	2531476	-	903	NADH pyrophosphatase
wy_02407	*kefC_2*	2531519	2532586	-	1068	Glutathione-regulated potassium-efflux system protein KefC
wy_02408	-	2532688	2533125	+	438	hypothetical protein

wy_02409	*pcrA_1*	2533135	2536503	-	3369	ATP-dependent DNA helicase PcrA
wy_02410	*pcrA_2*	2536509	2539826	-	3318	ATP-dependent DNA helicase PcrA
wy_02411	*ybfF*	2539883	2540659	+	777	Esterase YbfF
wy_02412	*ogt_1*	2540668	2540964	+	297	Methylated-DNA--protein-cysteine methyltransferase
wy_02413	*moeA_3*	2540988	2542775	+	1788	Molybdopterin molybdenumtransferase
wy_02414	-	2542783	2543133	-	351	hypothetical protein
wy_02415	*fbpA_3*	2543271	2544335	-	1065	Diacylglycerol acyltransferase/mycolyltransferase Ag85A precursor
wy_02416	*rsgA*	2545052	2545831	+	780	Putative ribosome biogenesis GTPase RsgA
wy_02417	-	2545863	2546372	+	510	Acetyltransferase (GNAT) family protein
wy_02418	-	2546345	2547385	-	1041	hypothetical protein
wy_02419	*nrdB*	2547441	2548412	-	972	R2-like ligand binding oxidase
wy_02420	-	2548646	2549455	+	810	hypothetical protein
wy_02421	*cbiX_1*	2549409	2550140	-	732	Sirohydrochlorin cobaltochelatase
wy_02422	-	2550137	2551306	-	1170	bifunctional uroporphyrinogen-III synthetase/response regulator domain protein
wy_02423	*nirD*	2551303	2551725	-	423	Nitrite reductase (NADH) small subunit
wy_02424	*nasD_1*	2551789	2554314	-	2526	Nitrite reductase [NAD(P)H]
wy_02425	*nasD_2*	2554311	2555798	-	1488	Nitrite reductase [NAD(P)H]
wy_02426	*nasC*	2555795	2557915	-	2121	Assimilatory nitrate reductase catalytic subunit
wy_02427	*narK*	2557912	2559360	-	1449	Nitrate/nitrite transporter NarK
wy_02428	*yycB*	2559493	2560767	-	1275	putative transporter YycB
wy_02429	*pdhR_2*	2560915	2561580	+	666	Pyruvate dehydrogenase complex repressor
wy_02430	-	2561608	2562462	-	855	hypothetical protein
wy_02431	*moeZ*	2562542	2563720	-	1179	putative adenylyltransferase/sulfurtransferase MoeZ
wy_02432	-	2563763	2564518	-	756	putative glycosyl hydrolase/MT2062
wy_02433	-	2564528	2565616	-	1089	hypothetical protein
wy_0243	*rutR_3*	256597	256664	+	672	HTH-type transcriptional regulator RutR

4		7	8			
wy_02435	-	2566716	2566946	-	231	hypothetical protein
wy_02436	-	2567053	2567733	-	681	hypothetical protein
wy_02437	*cshA*	2568104	2569741	+	1638	DEAD-box ATP-dependent RNA helicase CshA
wy_02438	-	2569762	2570976	+	1215	hypothetical protein
wy_02439	*soj_2*	2570992	2571795	-	804	Sporulation initiation inhibitor protein Soj
wy_02440	*pspA_2*	2571851	2572465	+	615	Phosphoserine phosphatase 1
wy_02441	-	2572462	2572977	+	516	putative acetyltransferase
wy_02442	-	2572991	2573359	-	369	hypothetical protein
wy_02443	*dagK_4*	2573459	2574415	+	957	Diacylglycerol kinase
wy_02444	*whiB1*	2574754	2575008	+	255	Transcriptional regulator WhiB1
wy_02445	*pdtaS*	2575098	2576609	-	1512	putative sensor histidine kinase pdtaS
wy_02446	-	2576638	2576853	-	216	Biotinylated protein TB7.3
wy_02447	*rshA*	2577109	2577408	-	300	Anti-sigma factor RshA
wy_02448	*sigH_1*	2577405	2578220	-	816	ECF RNA polymerase sigma factor SigH
wy_02449	*ybaK*	2578311	2578805	+	495	Cys-tRNA(Pro)/Cys-tRNA(Cys) deacylase YbaK
wy_02450	*luxA_2*	2578825	2579904	+	1080	Alkanal monooxygenase alpha chain
wy_02451	*yedK_2*	2579891	2580646	-	756	Putative SOS response-associated peptidase YedK
wy_02452	*aroA*	2580759	2582033	+	1275	3-phosphoshikimate 1-carboxyvinyltransferase
wy_02453	-	2581862	2582644	-	783	hypothetical protein
wy_02454	-	2582853	2584397	-	1545	NADH oxidase
wy_02455	-	2584511	2584894	-	384	hypothetical protein
wy_02456	*desA3_4*	2585048	2586286	-	1239	Stearoyl-CoA 9-desaturase
wy_02457	*desA3_5*	2586357	2587616	-	1260	Stearoyl-CoA 9-desaturase
wy_02458	-	2587738	2588853	-	1116	Stearoyl-CoA 9-desaturase electron transfer partner
wy_02459	-	2588972	2589487	-	516	hypothetical protein
wy_0246	*tgs3*	258950	259094	-	1437	putative diacylglycerol O-acyltransferase tgs3

0		9	5			
wy_02461	-	2590945	2591385	-	441	Phosphatase/MT3486
wy_02462	-	2591476	2592105	+	630	hypothetical protein
wy_02463	-	2592116	2594905	-	2790	preprotein translocase subunit SecA
wy_02464	*lrtA*	2595109	2595786	-	678	Light-repressed protein A
wy_02465	-	2596027	2596716	-	690	orotate phosphoribosyltransferase
wy_02466	*lpqB*	2596744	2598516	-	1773	Lipoprotein LpqB precursor
wy_02467	*mtrB_1*	2598513	2600360	-	1848	Sensor histidine kinase MtrB
wy_02468	*mtrA*	2600357	2601034	-	678	DNA-binding response regulator MtrA
wy_02469	*tmk*	2601101	2601763	-	663	Thymidylate kinase
wy_02470	-	2601829	2603106	-	1278	hypothetical protein
wy_02471	*ahcY*	2603226	2604704	-	1479	Adenosylhomocysteinase
wy_02472	-	2604780	2605388	-	609	Bacterial regulatory proteins, tetR family
wy_02473	*rubA*	2605422	2605604	-	183	Rubredoxin
wy_02474	*alkF*	2605625	2605786	-	162	Rubredoxin-1
wy_02475	*alkB_1*	2605783	2607000	-	1218	Alkane 1-monooxygenase
wy_02476	*yhdG_2*	2607109	2608635	-	1527	putative amino acid permease YhdG
wy_02477	*manA*	2608794	2610032	-	1239	Mannose-6-phosphate isomerase
wy_02478	-	2610032	2611147	-	1116	hypothetical protein
wy_02479	*algC*	2611232	2612614	-	1383	Phosphomannomutase/phosphoglucomutase
wy_02480	-	2612804	2613136	-	333	hypothetical protein
wy_02481	-	2613476	2613838	+	363	Possibl zinc metallo-peptidase
wy_02482	*whmD*	2613846	2614250	-	405	Transcriptional regulator WhiB2
wy_02483	*cofD*	2614560	2615555	+	996	2-phospho-L-lactate transferase
wy_02484	*fbiB*	2615552	2616919	+	1368	Coenzyme F420:L-glutamate ligase
wy_02485	-	2616920	2617483	+	564	NUDIX domain protein

wy_02486	*scoB_2*	2617522	2618169	-	648	Succinyl-CoA:3-ketoacid coenzyme A transferase subunit B
wy_02487	*scoA_2*	2618166	2618927	-	762	putative succinyl-CoA:3-ketoacid coenzyme A transferase subunit A
wy_02488	*pcaH*	2619094	2619816	+	723	Protocatechuate 3,4-dioxygenase beta chain
wy_02489	*pcaG*	2619809	2620453	+	645	Protocatechuate 3,4-dioxygenase alpha chain
wy_02490	*pcaB*	2620467	2621798	+	1332	3-carboxy-cis,cis-muconate cycloisomerase
wy_02491	*catD_2*	2621795	2622982	+	1188	3-oxoadipate enol-lactonase 2
wy_02492	*pcaR*	2622985	2623815	+	831	Pca regulon regulatory protein
wy_02493	*bktB*	2623808	2625019	+	1212	Beta-ketothiolase BktB
wy_02494	-	2625103	2625924	-	822	Xylose isomerase-like TIM barrel
wy_02495	*ksdD_2*	2625921	2627645	-	1725	3-oxosteroid 1-dehydrogenase
wy_02496	*afr*	2627642	2628736	-	1095	1,5-anhydro-D-fructose reductase
wy_02497	-	2628736	2629539	-	804	putative oxidoreductase/MSMEI_2347
wy_02498	-	2629536	2631512	-	1977	NADH oxidase
wy_02499	*aroE_2*	2631690	2632580	-	891	Quinate/shikimate dehydrogenase
wy_02500	*proP_1*	2632577	2633953	-	1377	Proline/betaine transporter
wy_02501	*gltC_2*	2634123	2635025	-	903	HTH-type transcriptional regulator GltC
wy_02502	*fabG_7*	2635154	2635849	+	696	3-oxoacyl-[acyl-carrier-protein] reductase FabG
wy_02503	-	2635891	2636808	-	918	NAD dependent epimerase/dehydratase family protein
wy_02504	*pobA*	2636962	2638149	-	1188	p-hydroxybenzoate hydroxylase
wy_02505	*kipR_3*	2638267	2639037	-	771	HTH-type transcriptional regulator KipR
wy_02506	*pcaK*	2639162	2640445	+	1284	4-hydroxybenzoate transporter PcaK
wy_02507	-	2640511	2641287	-	777	GDSL-like Lipase/Acylhydrolase
wy_02508	*hddC*	2641337	2642416	-	1080	D-glycero-alpha-D-manno-heptose 1-phosphate guanylyltransferase
wy_02509	*wbbL*	2642413	2643315	-	903	N-acetylglucosaminyl-diphospho-decaprenol L-rhamnosyltransferase
wy_02510	*rmlD*	2643350	2644195	-	846	dTDP-4-dehydrorhamnose reductase
wy_0251	*msrR_1*	264431	264581	+	1506	Regulatory protein MsrR

1		4	9			
wy_02512	-	2645859	2646578	+	720	hypothetical protein
wy_02513	*macB_2*	2646584	2649025	-	2442	Macrolide export ATP-binding/permease protein MacB
wy_02514	*bceA_2*	2649025	2649780	-	756	Bacitracin export ATP-binding protein BceA
wy_02515	*liaR_3*	2650271	2650939	-	669	Transcriptional regulatory protein LiaR
wy_02516	*desK_1*	2650950	2652134	-	1185	Sensor histidine kinase DesK
wy_02517	*purE*	2652167	2652676	-	510	N5-carboxyaminoimidazole ribonucleotide mutase
wy_02518	*purK*	2652725	2653930	-	1206	N5-carboxyaminoimidazole ribonucleotide synthase
wy_02519	-	2654122	2654670	+	549	GtrA-like protein
wy_02520	*tcrY_1*	2654667	2655992	-	1326	putative sensor histidine kinase TcrY
wy_02521	*arlR*	2656005	2656685	-	681	Response regulator ArlR
wy_02522	-	2656725	2657309	-	585	Bacterial membrane flanked domain protein
wy_02523	-	2657352	2657927	-	576	acid-resistance membrane protein
wy_02524	-	2658141	2658563	+	423	hypothetical protein
wy_02525	*birA_2*	2658567	2659400	-	834	Bifunctional ligase/repressor BirA
wy_02526	*accD5_1*	2659517	2661157	+	1641	putative propionyl-CoA carboxylase beta chain 5
wy_02527	-	2661154	2661492	+	339	hypothetical protein
wy_02528	*maf*	2661510	2662154	+	645	Septum formation protein Maf
wy_02529	-	2662164	2662646	-	483	hypothetical protein
wy_02530	*sseA_2*	2662668	2663579	+	912	Putative thiosulfate sulfurtransferase SseA
wy_02531	*csdE*	2663576	2663989	+	414	Sulfur acceptor protein CsdE
wy_02532	*papA3*	2664101	2665528	+	1428	Acyltransferase papA3
wy_02533	*accA1_1*	2665686	2667476	+	1791	Acetyl-/propionyl-coenzyme A carboxylase alpha chain
wy_02534	-	2667569	2667970	+	402	hypothetical protein
wy_02535	-	2667970	2668311	+	342	hypothetical protein
wy_02536	-	2668308	2668670	+	363	VanZ like family protein
wy_0253	*iphP_3*	266867	266949	-	816	Tyrosine-protein phosphatase precursor

7		8	3			
wy_02538	-	2669574	2671124	-	1551	Putative Rieske 2Fe-2S iron-sulfur protein/MSMEI_6242
wy_02539	*lat*	2671234	2672574	-	1341	putative L-lysine-epsilon aminotransferase
wy_02540	-	2672640	2673905	+	1266	hypothetical protein
wy_02541	*gabD_2*	2673902	2675431	+	1530	Succinate-semialdehyde dehydrogenase [NADP(+)]
wy_02542	*sadH_4*	2675428	2676270	-	843	Putative oxidoreductase SadH
wy_02543	*hapE_4*	2676267	2677733	-	1467	4-hydroxyacetophenone monooxygenase
wy_02544	-	2677730	2678677	-	948	Patatin-like phospholipase
wy_02545	*ttgR_3*	2678739	2679398	+	660	HTH-type transcriptional regulator TtgR
wy_02546	-	2679379	2679978	-	600	hypothetical protein
wy_02547	-	2680318	2684886	+	4569	putative ATP-dependent helicase Lhr
wy_02548	*nei1_2*	2684891	2685688	+	798	Endonuclease 8 1
wy_02549	-	2685661	2686278	-	618	DNA-binding transcriptional repressor FabR
wy_02550	-	2686434	2686973	+	540	hypothetical protein
wy_02551	*glpD_2*	2686951	2688705	-	1755	Aerobic glycerol-3-phosphate dehydrogenase
wy_02552	*glpK*	2688781	2690295	+	1515	Glycerol kinase
wy_02553	*relB*	2690259	2690837	-	579	Antitoxin RelB
wy_02554	*lysN*	2690961	2692184	-	1224	2-aminoadipate transaminase
wy_02555	*lpdA*	2692380	2693786	-	1407	NAD(P)H dehydrogenase (quinone)
wy_02556	-	2693965	2694435	+	471	hypothetical protein
wy_02557	*yxeP_2*	2694443	2695594	-	1152	putative hydrolase YxeP
wy_02558	*abgB*	2695591	2696727	-	1137	p-aminobenzoyl-glutamate hydrolase subunit B
wy_02559	*caiD_4*	2696798	2697523	-	726	Carnitinyl-CoA dehydratase
wy_02560	*punA*	2697551	2698336	+	786	Purine nucleoside phosphorylase
wy_02561	-	2698333	2699829	+	1497	putative phosphomannomutase
wy_02562	-	2699877	2700494	+	618	Peptidase propeptide and YPEB domain protein
wy_0256	-	270058	270126	+	687	hypothetical protein

3		1	7			
wy_02564	*upp*	2701268	2701840	-	573	Uracil phosphoribosyltransferase
wy_02565	-	2702002	2702337	+	336	hypothetical protein
wy_02566	*pgdS*	2702334	2703488	+	1155	Gamma-DL-glutamyl hydrolase precursor
wy_02567	-	2703515	2703868	+	354	hypothetical protein
wy_02568	-	2703865	2704170	+	306	hypothetical protein
wy_02569	-	2704218	2705459	+	1242	hypothetical protein
wy_02570	-	2705474	2706562	-	1089	Aminodeoxyfutalosine deaminase
wy_02571	-	2707152	2707556	+	405	Succinate dehydrogenase/Fumarate reductase transmembrane subunit
wy_02572	-	2707558	2708001	+	444	Succinate dehydrogenase/Fumarate reductase transmembrane subunit
wy_02573	*sdhA_2*	2708017	2709768	+	1752	Succinate dehydrogenase flavoprotein subunit
wy_02574	*sdhB*	2709768	2710544	+	777	Succinate dehydrogenase iron-sulfur subunit
wy_02575	*dacB*	2710658	2711995	+	1338	D-alanyl-D-alanine carboxypeptidase DacB precursor
wy_02576	*yhjD*	2712002	2713027	-	1026	Inner membrane protein YhjD
wy_02577	*trpS*	2713035	2714078	-	1044	Tryptophan--tRNA ligase
wy_02578	-	2714093	2714536	-	444	Barstar (barnase inhibitor)
wy_02579	-	2714536	2715024	-	489	Ribonuclease
wy_02580	*exoA*	2715024	2715851	-	828	Exodeoxyribonuclease
wy_02581	*ydhP_3*	2715848	2717047	-	1200	Inner membrane transport protein YdhP
wy_02582	*icd*	2717155	2718372	-	1218	Isocitrate dehydrogenase [NADP]
wy_02583	*mdeA*	2718931	2720244	+	1314	Methionine gamma-lyase
wy_02584	*metX_1*	2720241	2721386	+	1146	Homoserine O-acetyltransferase
wy_02585	-	2721417	2721692	-	276	hypothetical protein
wy_02586	*folD*	2721702	2722571	-	870	Bifunctional protein FolD protein
wy_02587	*aacA4*	2722594	2723100	+	507	Aminoglycoside N(6')-acetyltransferase type 1
wy_02588	*pipB2_1*	2723113	2723727	-	615	Secreted effector protein pipB2

wy_02589	*trmL*	2723819	2724271	+	453	tRNA (cytidine(34)-2'-O)-methyltransferase
wy_02590	*cmpR_1*	2724268	2725170	-	903	HTH-type transcriptional activator CmpR
wy_02591	-	2725273	2726310	+	1038	hypothetical protein
wy_02592	*fbpA_4*	2726482	2727507	+	1026	Diacylglycerol acyltransferase/mycolyltransferase Ag85A precursor
wy_02593	*cmpR_2*	2727529	2728422	-	894	HTH-type transcriptional activator CmpR
wy_02594	-	2728514	2729578	+	1065	C4-dicarboxylate transporter/malic acid transport protein
wy_02595	-	2729592	2729978	-	387	hypothetical protein
wy_02596	*csoR_1*	2730016	2730282	-	267	Copper-sensing transcriptional repressor CsoR
wy_02597	*ydfJ_1*	2730375	2732540	-	2166	Membrane protein YdfJ
wy_02598	*pspE*	2732731	2733039	-	309	Thiosulfate sulfurtransferase PspE precursor
wy_02599	*cdr*	2733067	2734722	-	1656	Coenzyme A disulfide reductase
wy_02600	*dnaE2*	2734790	2738137	-	3348	Error-prone DNA polymerase
wy_02601	*estB_3*	2738212	2739393	-	1182	Esterase EstB
wy_02602	-	2739509	2740501	-	993	transcriptional regulator EutR
wy_02603	-	2740539	2741147	-	609	hypothetical protein
wy_02604	*ydfJ_2*	2741241	2743361	+	2121	Membrane protein YdfJ
wy_02605	*acr1_6*	2743412	2745418	+	2007	Fatty acyl-CoA reductase
wy_02606	-	2745642	2746997	+	1356	Flavin-binding monooxygenase-like protein
wy_02607	-	2747005	2747943	-	939	Sucrase/ferredoxin-like protein
wy_02608	-	2747976	2748551	-	576	hypothetical protein
wy_02609	-	2748553	2749074	-	522	hypothetical protein
wy_02610	-	2749113	2749883	-	771	Arylesterase
wy_02611	*copB*	2749880	2751841	-	1962	Copper-exporting P-type ATPase B
wy_02612	-	2752021	2753577	-	1557	DNA polymerase IV
wy_02613	-	2753574	2754356	-	783	hypothetical protein
wy_0261	*prkC*	275459	275575	+	1164	Serine/threonine-protein kinase PrkC

4		2	5			
wy_02615	*liaR_4*	2755768	2756442	-	675	Transcriptional regulatory protein LiaR
wy_02616	*comP*	2756452	2757783	-	1332	Sensor histidine kinase ComP
wy_02617	-	2757924	2759222	+	1299	DNA-binding transcriptional activator PspC
wy_02618	-	2759215	2759493	+	279	hypothetical protein
wy_02619	*guaA*	2759562	2761040	-	1479	GMP synthase [glutamine-hydrolyzing]
wy_02620	*choD_2*	2761175	2762926	-	1752	Cholesterol oxidase
wy_02621	-	2763127	2764266	-	1140	putative oxidoreductase/MSMEI_1564
wy_02622	*guaB_2*	2764297	2765820	-	1524	Inosine-5'-monophosphate dehydrogenase
wy_02623	-	2766014	2766418	+	405	hypothetical protein
wy_02624	*rsdA*	2766519	2767499	-	981	Anti-sigma-D factor RsdA
wy_02625	*sigD_1*	2767492	2768070	-	579	ECF RNA polymerase sigma factor SigD
wy_02626	-	2768200	2769003	-	804	hypothetical protein
wy_02627	*whiB3_1*	2769501	2769791	+	291	putative transcriptional regulator WhiB3
wy_02628	-	2769953	2770612	+	660	Cell surface lipoprotein MPB83 precursor
wy_02629	*sigK_1*	2770663	2771328	+	666	ECF RNA polymerase sigma factor SigK
wy_02630	*rskA*	2771330	2772034	+	705	Anti-sigma-K factor RskA
wy_02631	*groL2*	2772111	2773724	-	1614	60 kDa chaperonin 2
wy_02632	*groS*	2773841	2774143	-	303	10 kDa chaperonin
wy_02633	-	2774340	2774774	-	435	Putative esterase
wy_02634	*ccrA2*	2774771	2775742	-	972	Crotonyl-CoA reductase
wy_02635	*bphA*	2775981	2777318	+	1338	Biphenyl dioxygenase subunit alpha
wy_02636	*hcaC*	2777315	2777662	+	348	3-phenylpropionate/cinnamic acid dioxygenase ferredoxin subunit
wy_02637	*camA*	2777671	2778897	+	1227	Putidaredoxin reductase
wy_02638	*bphE*	2778905	2779474	+	570	Biphenyl dioxygenase subunit beta
wy_02639	*hsaD_2*	2779541	2780410	+	870	4,5:9,10-diseco-3-hydroxy-5,9, 17-trioxoandrosta-1(10),2-diene-4-oate hydrolase

wy_02640	*bphB*	2780407	2781243	+	837	Cis-2,3-dihydrobiphenyl-2,3-diol dehydrogenase
wy_02641	-	2781240	2782841	+	1602	Long-chain-fatty-acid--CoA ligase FadD13
wy_02642	-	2782838	2783236	+	399	hypothetical protein
wy_02643	-	2783391	2784221	+	831	Leucine carboxyl methyltransferase
wy_02644	-	2784258	2785259	+	1002	Endonuclease/Exonuclease/phosphatase family protein
wy_02645	-	2785229	2786941	-	1713	hypothetical protein
wy_02646	-	2787167	2787403	+	237	hypothetical protein
wy_02647	*acoC*	2787465	2788283	-	819	Dihydrolipoyllysine-residue acetyltransferase component of acetoin cleaving system
wy_02648	*mhpB_2*	2788391	2789323	-	933	2,3-dihydroxyphenylpropionate/2, 3-dihydroxicinnamic acid 1,2-dioxygenase
wy_02649	*kdgR_2*	2789390	2790280	-	891	Transcriptional regulator KdgR
wy_02650	-	2790404	2791000	+	597	anaerobic benzoate catabolism transcriptional regulator
wy_02651	-	2791093	2792820	+	1728	hypothetical protein
wy_02652	-	2792817	2793410	-	594	Ion channel
wy_02653	-	2793658	2795079	+	1422	Hsp70 protein
wy_02654	*tsaD*	2795024	2796064	-	1041	tRNA N6-adenosine threonylcarbamoyltransferase
wy_02655	-	2796070	2796567	-	498	ribosomal-protein-alanine N-acetyltransferase
wy_02656	*tsaB*	2796564	2797238	-	675	tRNA threonylcarbamoyladenosine biosynthesis protein TsaB
wy_02657	-	2797380	2798258	+	879	hypothetical protein
wy_02658	*tsaE*	2798263	2798808	-	546	tRNA threonylcarbamoyladenosine biosynthesis protein TsaE
wy_02659	*catD_3*	2798765	2799859	-	1095	3-oxoadipate enol-lactonase 2
wy_02660	*alr*	2799856	2801082	-	1227	Alanine racemase
wy_02661	*nnr*	2801141	2802604	-	1464	Bifunctional NAD(P)H-hydrate repair enzyme Nnr
wy_02662	-	2802610	2803386	-	777	27 kDa antigen Cfp30B
wy_02663	*mauC_1*	2803461	2803832	+	372	Amicyanin precursor
wy_02664	*glmS*	2803857	2805719	-	1863	Glutamine--fructose-6-phosphate aminotransferase [isomerizing]
wy_0266	-	280574	280667	+	927	Alpha/beta hydrolase family protein

5		5	1			
wy_02666	-	2806505	2807227	-	723	hypothetical protein
wy_02667	-	2807312	2808325	-	1014	hypothetical protein
wy_02668	-	2808325	2808627	-	303	hypothetical protein
wy_02669	*glmM*	2808701	2810038	-	1338	Phosphoglucosamine mutase
wy_02670	*rpsI*	2810181	2810687	-	507	30S ribosomal protein S9
wy_02671	*rplM*	2810684	2811127	-	444	50S ribosomal protein L13
wy_02672	*rocD*	2811314	2812555	-	1242	Ornithine aminotransferase
wy_02673	*esxA*	2812721	2813002	-	282	6 kDa early secretory antigenic target
wy_02674	-	2813043	2813357	-	315	Proteins of 100 residues with WXG
wy_02675	-	2813540	2814946	-	1407	hypothetical protein
wy_02676	*eccCa1*	2814928	2818923	-	3996	ESX-1 secretion system protein EccCa1
wy_02677	*eccD1*	2819152	2820627	+	1476	ESX-1 secretion system protein eccD1
wy_02678	-	2820627	2822015	+	1389	Thermophilic serine proteinase precursor
wy_02679	*eccB1*	2822012	2823484	-	1473	ESX-1 secretion system protein eccB1
wy_02680	*eccE1*	2823597	2825132	+	1536	ESX-1 secretion system protein EccE1
wy_02681	*azr_2*	2825144	2825806	-	663	FMN-dependent NADPH-azoreductase
wy_02682	-	2825846	2826415	-	570	Bacterial regulatory proteins, tetR family
wy_02683	*acdA_5*	2826488	2827630	+	1143	Acyl-CoA dehydrogenase
wy_02684	*truA*	2827637	2828527	-	891	tRNA pseudouridine synthase A
wy_02685	*rplQ*	2828533	2829075	-	543	50S ribosomal protein L17
wy_02686	*rpoA*	2829131	2830195	-	1065	DNA-directed RNA polymerase subunit alpha
wy_02687	*rpsD*	2830296	2830901	-	606	30S ribosomal protein S4
wy_02688	*rpsK*	2830928	2831341	-	414	30S ribosomal protein S11
wy_02689	*rpsM*	2831341	2831709	-	369	30S ribosomal protein S13
wy_02690	*rpmJ*	2831920	2832033	-	114	50S ribosomal protein L36
wy_0269	*infA*	283211	283236	-	258	Translation initiation factor IF-1

1		1	8			
wy_02692	*dtd*	2832563	2833000	+	438	D-tyrosyl-tRNA(Tyr) deacylase
wy_02693	*map-1_1*	2833085	2833267	-	183	Methionine aminopeptidase 1
wy_02694	*map-1_2*	2833264	2833680	-	417	Methionine aminopeptidase 1
wy_02695	*adk*	2833680	2834225	-	546	Adenylate kinase
wy_02696	-	2834238	2835746	-	1509	preprotein translocase subunit SecY
wy_02697	*rplO*	2835969	2836409	-	441	50S ribosomal protein L15
wy_02698	*rpmD*	2836412	2836591	-	180	50S ribosomal protein L30
wy_02699	*rpsE*	2836591	2837232	-	642	30S ribosomal protein S5
wy_02700	*rplR*	2837264	2837671	-	408	50S ribosomal protein L18
wy_02701	*rplF*	2837673	2838212	-	540	50S ribosomal protein L6
wy_02702	*rpsH*	2838227	2838625	-	399	30S ribosomal protein S8
wy_02703	*rpsZ*	2838796	2838981	-	186	30S ribosomal protein S14 type Z
wy_02704	*rplE*	2838986	2839552	-	567	50S ribosomal protein L5
wy_02705	*rplX*	2839554	2839871	-	318	50S ribosomal protein L24
wy_02706	*rplN*	2839871	2840239	-	369	50S ribosomal protein L14
wy_02707	*rpsQ*	2840410	2840700	-	291	30S ribosomal protein S17
wy_02708	*rpmC*	2840697	2840933	-	237	50S ribosomal protein L29
wy_02709	*rplP*	2840933	2841349	-	417	50S ribosomal protein L16
wy_02710	*rpsC*	2841353	2842150	-	798	30S ribosomal protein S3
wy_02711	*rplV*	2842150	2842554	-	405	50S ribosomal protein L22
wy_02712	*rpsS*	2842551	2842832	-	282	30S ribosomal protein S19
wy_02713	*rplB*	2842851	2843684	-	834	50S ribosomal protein L2
wy_02714	*rplW*	2843725	2844030	-	306	50S ribosomal protein L23
wy_02715	*rplD*	2844027	2844692	-	666	50S ribosomal protein L4
wy_02716	*rplC*	2844689	2845342	-	654	50S ribosomal protein L3
wy_0271	*rpsJ*	284537	284567	-	306	30S ribosomal protein S10

7		1	6			
wy_02718	-	2846162	2846641	+	480	hypothetical protein
wy_02719	*mcbR*	2846790	2847476	+	687	HTH-type transcriptional regulator McbR
wy_02720	*drrA_7*	2847674	2848645	+	972	Doxorubicin resistance ATP-binding protein DrrA
wy_02721	*drrB_4*	2848642	2849505	+	864	Doxorubicin resistance ABC transporter permease protein DrrB
wy_02722	*drrB_5*	2849502	2850374	+	873	Daunorubicin/doxorubicin resistance ABC transporter permease protein DrrB
wy_02723	*estB_4*	2850545	2851477	+	933	Extracellular esterase EstB precursor
wy_02724	*tycC_1*	2851557	2863739	-	12183	Tyrocidine synthase 3
wy_02725	*grsB*	2863842	2874545	-	10704	Gramicidin S synthase 2
wy_02726	*tycC_2*	2874633	2895389	-	20757	Tyrocidine synthase 3
wy_02727	-	2895746	2896183	-	438	Pyridoxamine 5'-phosphate oxidase
wy_02728	*pdhR_3*	2896228	2896983	-	756	Pyruvate dehydrogenase complex repressor
wy_02729	*acsA_3*	2897145	2899106	-	1962	Acetyl-coenzyme A synthetase
wy_02730	*citE_2*	2899110	2899967	-	858	Citrate lyase subunit beta-like protein
wy_02731	*paaZ*	2899964	2900437	-	474	Bifunctional protein PaaZ
wy_02732	*acdA_6*	2900441	2901604	-	1164	Acyl-CoA dehydrogenase
wy_02733	*accA1_2*	2901601	2903625	-	2025	Acetyl-/propionyl-coenzyme A carboxylase alpha chain
wy_02734	-	2903634	2905193	-	1560	Methylmalonyl-CoA carboxyltransferase 12S subunit
wy_02735	*ethR_1*	2905311	2905940	+	630	HTH-type transcriptional regulator EthR
wy_02736	-	2905946	2906209	-	264	hypothetical protein
wy_02737	*adhD_2*	2906329	2907441	-	1113	Putative alcohol dehydrogenase D
wy_02738	*feaB*	2907481	2908974	-	1494	Phenylacetaldehyde dehydrogenase
wy_02739	*acoR*	2909081	2910724	-	1644	Acetoin dehydrogenase operon transcriptional activator AcoR
wy_02740	*lcfB_11*	2910737	2912389	-	1653	Long-chain-fatty-acid--CoA ligase
wy_02741	*yhjE_1*	2912561	2913877	-	1317	Inner membrane metabolite transport protein YhjE
wy_02742	-	2913993	2914649	-	657	hypothetical protein

wy_02743	-	2914711	2915271	-	561	hypothetical protein
wy_02744	-	2915336	2915866	-	531	hypothetical protein
wy_02745	*adh_3*	2915863	2916888	-	1026	Alcohol dehydrogenase
wy_02746	*thcA*	2916916	2918439	-	1524	EPTC-inducible aldehyde dehydrogenase
wy_02747	-	2918697	2920319	+	1623	Long-chain-fatty-acid--CoA ligase FadD19
wy_02748	*betA*	2920321	2921802	-	1482	Oxygen-dependent choline dehydrogenase
wy_02749	*pgaC*	2921805	2923106	-	1302	Poly-beta-1,6-N-acetyl-D-glucosamine synthase
wy_02750	*crnA*	2923276	2923989	-	714	Creatinine amidohydrolase
wy_02751	-	2923986	2924837	-	852	hypothetical protein
wy_02752	-	2924908	2926209	+	1302	HNH endonuclease
wy_02753	*stcD_2*	2926237	2928195	-	1959	putative N-methylproline demethylase
wy_02754	-	2928208	2929020	-	813	putative NAD-dependent oxidoreductase
wy_02755	*stcD_3*	2929030	2930961	-	1932	putative N-methylproline demethylase
wy_02756	*lldD*	2930974	2932152	-	1179	L-lactate dehydrogenase [cytochrome]
wy_02757	*albA*	2932186	2933436	-	1251	Antilisterial bacteriocin subtilosin biosynthesis protein AlbA
wy_02758	-	2933498	2933800	-	303	hypothetical protein
wy_02759	-	2933803	2933904	-	102	hypothetical protein
wy_02760	*ethR_2*	2934029	2934661	+	633	HTH-type transcriptional regulator EthR
wy_02761	-	2934658	2935461	+	804	Methyltransferase domain protein
wy_02762	*hmp_2*	2935621	2936676	+	1056	3-ketosteroid-9-alpha-hydroxylase reductase subunit
wy_02763	-	2936869	2937231	+	363	SPW repeat protein
wy_02764	*acrR_1*	2937257	2937874	+	618	HTH-type transcriptional regulator AcrR
wy_02765	*murI*	2937898	2938665	-	768	Glutamate racemase
wy_02766	*thcD_2*	2938684	2939925	-	1242	Rhodocoxin reductase
wy_02767	-	2940068	2940547	+	480	Alkaline shock protein 23
wy_02768	-	2940544	2940864	+	321	hypothetical protein

wy_02769	-	2940861	2941040	+	180	hypothetical protein
wy_02770	-	2940988	2941986	+	999	hypothetical protein
wy_02771	-	2941983	2942600	+	618	hypothetical protein
wy_02772	*pipB2_2*	2942605	2943234	+	630	Secreted effector protein pipB2
wy_02773	-	2943228	2943599	-	372	SnoaL-like domain protein
wy_02774	*gltC_3*	2943702	2944574	+	873	HTH-type transcriptional regulator GltC
wy_02775	*tuf*	2944678	2945868	-	1191	Elongation factor Tu
wy_02776	*fusA*	2945958	2948060	-	2103	Elongation factor G
wy_02777	*rpsG*	2948212	2948682	-	471	30S ribosomal protein S7
wy_02778	*rpsL*	2948682	2949056	-	375	30S ribosomal protein S12
wy_02779	-	2949387	2949974	-	588	hypothetical protein
wy_02780	-	2949976	2950479	-	504	hypothetical protein
wy_02781	*betI*	2950566	2951180	-	615	HTH-type transcriptional regulator BetI
wy_02782	-	2951390	2951524	+	135	hypothetical protein
wy_02783	*fadB_2*	2951579	2952367	-	789	putative enoyl-CoA hydratase
wy_02784	-	2952379	2953542	-	1164	Acyl-CoA dehydrogenase fadE12
wy_02785	*accA1_3*	2953539	2955548	-	2010	Acetyl-/propionyl-coenzyme A carboxylase alpha chain
wy_02786	*accD5_2*	2955557	2957155	-	1599	putative propionyl-CoA carboxylase beta chain 5
wy_02787	*mmgC_3*	2957152	2958300	-	1149	Acyl-CoA dehydrogenase
wy_02788	-	2958297	2960048	-	1752	hypothetical protein
wy_02789	-	2960045	2960845	-	801	mycothiol-dependent maleylpyruvate isomerase
wy_02790	-	2960955	2961308	-	354	hypothetical protein
wy_02791	*hsrA_2*	2961454	2962965	-	1512	putative transport protein HsrA
wy_02792	-	2962962	2963423	-	462	MarR family protein
wy_02793	*adhT_2*	2963584	2964549	-	966	Alcohol dehydrogenase
wy_02794	*fprA_2*	2964621	2966315	-	1695	NADPH-ferredoxin reductase FprA

wy_02795	-	2966524	2967333	+	810	Acyl-ACP thioesterase
wy_02796	-	2967335	2967535	-	201	hypothetical protein
wy_02797	*rpoC*	2967651	2971610	-	3960	DNA-directed RNA polymerase subunit beta'
wy_02798	*rpoB*	2971744	2975232	-	3489	DNA-directed RNA polymerase subunit beta
wy_02799	-	2975639	2976361	-	723	hypothetical protein
wy_02800	-	2976431	2977057	-	627	hypothetical protein
wy_02801	-	2977098	2978348	-	1251	mce related protein
wy_02802	-	2978348	2979595	-	1248	mce related protein
wy_02803	-	2979592	2980839	-	1248	mce related protein
wy_02804	-	2980841	2981836	-	996	mce related protein
wy_02805	-	2981833	2982912	-	1080	mce related protein
wy_02806	-	2982909	2984240	-	1332	mce related protein
wy_02807	*mlaE_1*	2984237	2985097	-	861	putative phospholipid ABC transporter permease protein MlaE
wy_02808	*mlaE_2*	2985102	2985896	-	795	putative phospholipid ABC transporter permease protein MlaE
wy_02809	-	2985908	2986954	-	1047	putative ABC transporter ATP-binding protein
wy_02810	*rplL*	2987189	2987575	-	387	50S ribosomal protein L7/L12
wy_02811	*rplJ*	2987660	2988217	-	558	50S ribosomal protein L10
wy_02812	*rplA*	2988667	2989380	-	714	50S ribosomal protein L1
wy_02813	*rplK*	2989503	2989937	-	435	50S ribosomal protein L11
wy_02814	-	2990015	2990812	-	798	hypothetical protein
wy_02815	-	2990872	2991360	-	489	preprotein translocase subunit SecE
wy_02817	-	2991627	2992052	-	426	bifunctional enoyl-CoA hydratase/phosphate acetyltransferase
wy_02818	-	2992039	2992587	-	549	hypothetical protein
wy_02819	*rpmG*	2992674	2992841	-	168	50S ribosomal protein L33
wy_02822	*geoB_1*	2993236	2994732	-	1497	Geranial dehydrogenase
wy_0282	-	299518	299567	+	492	putative nucleotide-binding protein

4		7	8			
wy_02825	-	2995669	2996661	-	993	hypothetical protein
wy_02826	*hepT*	2996767	2997795	-	1029	Heptaprenyl diphosphate synthase component 2
wy_02827	-	2997930	2999213	+	1284	Putative oxidoreductase/MT0587
wy_02828	*ubiE_2*	2999241	2999975	-	735	Ubiquinone/menaquinone biosynthesis C-methyltransferase UbiE
wy_02829	-	2999962	3000606	-	645	undecaprenyl pyrophosphate phosphatase
wy_02830	-	3000670	3001092	-	423	hypothetical protein
wy_02831	*menD*	3001089	3002816	-	1728	2-succinyl-5-enolpyruvyl-6-hydroxy-3-cyclohexene-1-carboxylate synthase
wy_02832	*flr*	3002844	3003443	+	600	Flavoredoxin
wy_02833	*viuB_3*	3003510	3004334	+	825	Vibriobactin utilization protein ViuB
wy_02834	-	3004403	3004849	-	447	hypothetical protein
wy_02835	*ykfB*	3004895	3005893	-	999	L-Ala-D/L-Glu epimerase
wy_02836	-	3005890	3006555	-	666	hypothetical protein
wy_02837	*menB_1*	3006641	3007534	+	894	1,4-Dihydroxy-2-naphthoyl-CoA synthase
wy_02838	-	3008285	3008917	+	633	Putative phosphoribosyl transferase/MT0597
wy_02839	*recQ_2*	3008914	3010533	+	1620	ATP-dependent DNA helicase RecQ
wy_02840	-	3010568	3011017	+	450	hypothetical protein
wy_02841	-	3011043	3011423	+	381	Glyoxalase-like domain protein
wy_02842	-	3011485	3011838	-	354	hypothetical protein
wy_02843	*livF_1*	3011841	3012593	-	753	High-affinity branched-chain amino acid transport ATP-binding protein LivF
wy_02844	*lptB_1*	3012593	3013336	-	744	Lipopolysaccharide export system ATP-binding protein LptB
wy_02845	*livH_1*	3013329	3015629	-	2301	High-affinity branched-chain amino acid transport system permease protein LivH
wy_02846	-	3015756	3017171	-	1416	hypothetical protein
wy_02847	*pitA*	3017543	3018835	+	1293	Low-affinity inorganic phosphate transporter 1
wy_02848	-	3018849	3019121	+	273	hypothetical protein
wy_02849	-	3019156	3019515	+	360	hypothetical protein

wy_02850	-	3019538	3019840	+	303	hypothetical protein
wy_02851	*menE*	3019882	3021048	+	1167	2-succinylbenzoate--CoA ligase
wy_02852	-	3021427	3021987	+	561	hypothetical protein
wy_02853	-	3022216	3022749	+	534	hypothetical protein
wy_02854	*drrA_8*	3022882	3023856	+	975	Daunorubicin/doxorubicin resistance ATP-binding protein DrrA
wy_02855	*drrB_6*	3023853	3024683	+	831	Daunorubicin/doxorubicin resistance ABC transporter permease protein DrrB
wy_02856	-	3024688	3027945	-	3258	Putative HTH-type transcriptional regulator/MT0914
wy_02857	*menA*	3028066	3028935	+	870	1,4-dihydroxy-2-naphthoate octaprenyltransferase
wy_02858	-	3028960	3029340	-	381	Membrane protein of unknown function
wy_02859	-	3029393	3029734	-	342	hypothetical protein
wy_02860	-	3029887	3030216	+	330	hypothetical protein
wy_02861	-	3030195	3030947	-	753	Acyltransferase
wy_02862	-	3031034	3031951	-	918	Phthiodiolone/phenolphthiodiolone dimycocerosates ketoreductase
wy_02863	*fabG_8*	3031995	3032846	-	852	3-oxoacyl-[acyl-carrier-protein] reductase FabG
wy_02864	-	3033033	3033332	+	300	hypothetical protein
wy_02865	-	3033335	3033877	+	543	putative acetyltransferase
wy_02866	*sbnA*	3033874	3034968	-	1095	putative siderophore biosynthesis protein SbnA
wy_02867	-	3034965	3035252	-	288	hypothetical protein
wy_02868	-	3035352	3035549	-	198	hypothetical protein
wy_02869	-	3035581	3035787	-	207	hypothetical protein
wy_02870	-	3036192	3036404	+	213	Helix-turn-helix domain protein
wy_02871	-	3036486	3036707	+	222	hypothetical protein
wy_02872	*ccsA*	3036817	3037791	-	975	Cytochrome c biogenesis protein CcsA
wy_02873	*ccsB*	3037794	3039485	-	1692	Cytochrome c biogenesis protein CcsB
wy_02874	-	3039500	3040327	-	828	Cytochrome C biogenesis protein transmembrane region
wy_0287	*resA_2*	304032	304093	-	615	Thiol-disulfide oxidoreductase ResA

5		4	8			
wy_02876	*gpgP_2*	3040935	3041612	-	678	Glucosyl-3-phosphoglycerate phosphatase
wy_02877	*hemL2*	3041669	3043003	-	1335	Glutamate-1-semialdehyde 2,1-aminomutase 2
wy_02878	*mmgC_4*	3043152	3045074	+	1923	Acyl-CoA dehydrogenase
wy_02879	*prmB*	3045068	3045874	-	807	50S ribosomal protein L3 glutamine methyltransferase
wy_02880	*kynB_2*	3046095	3047006	-	912	Kynurenine formamidase
wy_02881	*yliI_2*	3047019	3048188	-	1170	Soluble aldose sugar dehydrogenase YliI precursor
wy_02882	-	3048300	3048770	-	471	hypothetical protein
wy_02883	-	3048879	3049112	-	234	hypothetical protein
wy_02884	*copA_3*	3049238	3051406	-	2169	Copper-exporting P-type ATPase A
wy_02885	*copZ_2*	3051406	3051609	-	204	Copper chaperone CopZ
wy_02886	*csoR_2*	3051644	3051934	-	291	Copper-sensing transcriptional repressor CsoR
wy_02887	-	3051927	3052190	-	264	hypothetical protein
wy_02888	-	3052187	3052774	-	588	hypothetical protein
wy_02889	-	3052749	3053159	-	411	hypothetical protein
wy_02890	-	3053224	3053340	-	117	hypothetical protein
wy_02891	-	3053433	3054080	-	648	hypothetical protein
wy_02892	*hemB*	3054077	3054955	-	879	Delta-aminolevulinic acid dehydratase
wy_02893	*nasF*	3055072	3056637	-	1566	Uroporphyrinogen-III C-methyltransferase
wy_02894	*hemC*	3056710	3057714	-	1005	Porphobilinogen deaminase
wy_02895	*hemA*	3057711	3059084	-	1374	Glutamyl-tRNA reductase
wy_02896	*rex*	3059081	3059758	-	678	Redox-sensing transcriptional repressor Rex
wy_02897	-	3060088	3060351	-	264	hypothetical protein
wy_02898	*serB_2*	3060543	3061688	+	1146	Phosphoserine phosphatase
wy_02899	-	3061646	3062677	-	1032	2-acyl-glycerophospho-ethanolamine acyltransferase
wy_02900	*galE_2*	3062757	3063827	-	1071	UDP-glucose 4-epimerase
wy_0290	-	306400	306410	-	102	hypothetical protein

1		4	5			
wy_02902	-	3064316	3064558	-	243	Helix-turn-helix domain protein
wy_02903	-	3064735	3065448	-	714	CAAX amino terminal protease self- immunity
wy_02904	*proC*	3065540	3066355	-	816	Pyrroline-5-carboxylate reductase
wy_02905	-	3066437	3067294	-	858	hypothetical protein
wy_02906	-	3067291	3067995	-	705	hypothetical protein
wy_02907	-	3068062	3068481	+	420	hypothetical protein
wy_02908	-	3068515	3069357	-	843	fructoselysine 3-epimerase
wy_02909	-	3069396	3070511	-	1116	hypothetical protein
wy_02910	*gppA_2*	3070508	3071377	-	870	Guanosine-5'-triphosphate,3'-diphosphate pyrophosphatase
wy_02911	-	3071399	3072316	+	918	hypothetical protein
wy_02912	*regX3*	3072387	3073076	-	690	Sensory transduction protein regX3
wy_02913	*senX3*	3073073	3074290	-	1218	Signal-transduction histidine kinase senX3
wy_02914	*gpmA*	3074571	3075317	-	747	2,3-bisphosphoglycerate-dependent phosphoglycerate mutase
wy_02915	-	3075385	3075669	-	285	Antibiotic biosynthesis monooxygenase
wy_02916	*rpfE_4*	3075899	3076228	-	330	Resuscitation-promoting factor RpfE precursor
wy_02917	-	3076647	3077201	-	555	hypothetical protein
wy_02918	*mshA_2*	3077198	3078532	-	1335	D-inositol 3-phosphate glycosyltransferase
wy_02919	*cpo_1*	3078558	3079439	-	882	Non-heme chloroperoxidase
wy_02920	-	3079463	3080650	-	1188	Major Facilitator Superfamily protein
wy_02921	*mshB_3*	3080770	3081543	+	774	1D-myo-inositol 2-acetamido-2-deoxy-alpha-D-glucopyranoside deacetylase
wy_02922	-	3081593	3082612	+	1020	hypothetical protein
wy_02923	-	3082632	3083945	+	1314	putative inactive lipase/MT1628
wy_02924	-	3083942	3085195	+	1254	hypothetical protein
wy_02925	*nagC*	3085215	3086516	-	1302	N-acetylglucosamine repressor
wy_02926	*sdh*	3086725	3087591	+	867	Serine 3-dehydrogenase

wy_02927	-	3087601	3088653	+	1053	hypothetical protein
wy_02928	-	3088664	3088828	-	165	hypothetical protein
wy_02929	-	3088842	3089738	-	897	hypothetical protein
wy_02930	-	3089738	3090052	-	315	hypothetical protein
wy_02931	-	3090045	3090572	-	528	hypothetical protein
wy_02932	-	3090671	3091447	-	777	hypothetical protein
wy_02933	-	3091484	3091984	-	501	hypothetical protein
wy_02934	-	3091995	3092807	-	813	Alpha/beta hydrolase family protein
wy_02935	*lppS_2*	3092910	3094055	-	1146	Putative L,D-transpeptidase LppS precursor
wy_02936	*murB*	3094182	3095285	-	1104	UDP-N-acetylenolpyruvoylglucosamine reductase
wy_02937	-	3095309	3095812	+	504	hypothetical protein
wy_02938	-	3095858	3096358	+	501	hypothetical protein
wy_02939	-	3096364	3097143	+	780	hypothetical protein
wy_02940	-	3097154	3098188	-	1035	hypothetical protein
wy_02941	-	3098273	3098884	-	612	hypothetical protein
wy_02942	-	3099010	3099774	+	765	hypothetical protein
wy_02943	*deoC*	3099782	3100462	-	681	Deoxyribose-phosphate aldolase
wy_02944	*ubiE_3*	3100500	3101342	+	843	Demethylmenaquinone methyltransferase
wy_02945	*purU*	3101361	3102248	+	888	Formyltetrahydrofolate deformylase
wy_02946	*cpo_2*	3102255	3103283	-	1029	Non-heme chloroperoxidase
wy_02947	-	3103301	3104062	-	762	zinc-responsive transcriptional regulator
wy_02948	-	3104152	3105084	-	933	P-aminobenzoate N-oxygenase AurF
wy_02949	-	3105239	3105532	-	294	hypothetical protein
wy_02950	*hbhA*	3105631	3106488	-	858	Heparin-binding hemagglutinin
wy_02951	*sinR_1*	3106485	3106952	-	468	HTH-type transcriptional regulator SinR
wy_02952	-	3107055	3108266	-	1212	hypothetical protein

wy_02953	-	3108495	3109256	+	762	transcriptional regulator BetI
wy_02954	-	3109297	3109647	+	351	hypothetical protein
wy_02955	*ubiB_2*	3109722	3111095	+	1374	putative protein kinase UbiB
wy_02956	-	3111136	3111996	+	861	Polyphosphate kinase 2 (PPK2)
wy_02957	-	3112010	3112231	-	222	hypothetical protein
wy_02958	*adhC*	3112388	3113428	-	1041	NADP-dependent alcohol dehydrogenase C
wy_02959	-	3113607	3115085	+	1479	Cellulase (glycosyl hydrolase family 5)
wy_02960	*metE_2*	3115161	3117449	-	2289	5-methyltetrahydropteroyltriglutamate--homocysteine methyltransferase
wy_02961	*fadB2_1*	3117541	3118437	-	897	3-hydroxybutyryl-CoA dehydrogenase
wy_02962	*aceA*	3118529	3119818	-	1290	Isocitrate lyase
wy_02963	-	3120267	3121130	-	864	hypothetical protein
wy_02964	-	3121507	3122916	+	1410	anaerobic benzoate catabolism transcriptional regulator
wy_02965	*amiC_2*	3123125	3124393	+	1269	Aliphatic amidase expression-regulating protein
wy_02966	*livH_2*	3124427	3125311	+	885	High-affinity branched-chain amino acid transport system permease protein LivH
wy_02967	-	3125308	3126399	+	1092	leucine/isoleucine/valine transporter permease subunit
wy_02968	*occP*	3126396	3127193	+	798	Octopine permease ATP-binding protein P
wy_02969	*livF_2*	3127196	3127888	+	693	High-affinity branched-chain amino acid transport ATP-binding protein LivF
wy_02970	*accA1_4*	3127941	3131528	+	3588	Acetyl-/propionyl-coenzyme A carboxylase alpha chain
wy_02971	*fadK_1*	3131518	3133149	-	1632	Short-chain-fatty-acid--CoA ligase
wy_02972	*livF_3*	3133152	3133865	-	714	High-affinity branched-chain amino acid transport ATP-binding protein LivF
wy_02973	*lptB_2*	3133862	3134632	-	771	Lipopolysaccharide export system ATP-binding protein LptB
wy_02974	-	3134629	3135669	-	1041	leucine/isoleucine/valine transporter permease subunit
wy_02975	*rbsC*	3135669	3136544	-	876	Ribose transport system permease protein RbsC
wy_02976	*braC_1*	3136560	3137753	-	1194	Leucine-, isoleucine-, valine-, threonine-, and alanine-binding protein precursor
wy_02977	*kstR2_8*	3137888	3138643	-	756	HTH-type transcriptional repressor KstR2
wy_0297	-	313880	313969	+	885	putative NAD-dependent oxidoreductase

8		9	3			
wy_02979	-	3139690	3140259	+	570	Carboxymuconolactone decarboxylase family protein
wy_02980	*ywaC*	3140325	3140987	+	663	GTP pyrophosphokinase YwaC
wy_02981	*efpA_3*	3141156	3142745	+	1590	putative MFS-type transporter EfpA
wy_02982	*qorB*	3142838	3143695	-	858	Quinone oxidoreductase 2
wy_02983	*hxlR*	3143739	3144203	+	465	HTH-type transcriptional activator HxlR
wy_02984	*lipR_2*	3144227	3145111	+	885	Putative acetyl-hydrolase LipR precursor
wy_02985	*lpdC*	3145131	3146534	-	1404	Dihydrolipoyl dehydrogenase
wy_02986	-	3146677	3146829	-	153	hypothetical protein
wy_02987	-	3148282	3148383	-	102	hypothetical protein
wy_02988	-	3148397	3148513	-	117	hypothetical protein
wy_02989	-	3148531	3148728	-	198	hypothetical protein
wy_02990	-	3148756	3148869	-	114	hypothetical protein
wy_02991	-	3148906	3149037	-	132	hypothetical protein
wy_02992	-	3149730	3150452	-	723	hypothetical protein
wy_02993	*lrpC_1*	3150958	3151917	+	960	HTH-type transcriptional regulator LrpC
wy_02994	-	3151880	3153925	+	2046	Prolyl endopeptidase
wy_02995	*fbpA_5*	3153922	3154923	+	1002	Diacylglycerol acyltransferase/mycolyltransferase Ag85A precursor
wy_02996	-	3154991	3155131	-	141	hypothetical protein
wy_02997	*rutD*	3155257	3156093	-	837	Putative aminoacrylate hydrolase RutD
wy_02998	*pspC*	3156354	3156554	+	201	Phage shock protein C
wy_02999	*groL1*	3156657	3158282	-	1626	60 kDa chaperonin 1
wy_03000	-	3158526	3159050	-	525	hypothetical protein
wy_03001	*nagD_1*	3159161	3160003	-	843	Ribonucleotide monophosphatase NagD
wy_03002	-	3160073	3160540	+	468	Polyketide cyclase / dehydrase and lipid transport
wy_03003	-	3160635	3161348	+	714	phosphatidylserine decarboxylase

wy_03004	-	3161396	3162184	+	789	CDP-alcohol phosphatidyltransferase
wy_03005	-	3162344	3163036	-	693	hypothetical protein
wy_03006	*ftsH_2*	3163721	3165649	+	1929	ATP-dependent zinc metalloprotease FtsH
wy_03007	*prpB_2*	3165662	3166414	-	753	Methylisocitrate lyase
wy_03008	*bm3R1_2*	3166515	3167177	-	663	HTH-type transcriptional repressor Bm3R1
wy_03009	*efpA_4*	3167346	3168848	+	1503	putative MFS-type transporter EfpA
wy_03010	-	3168934	3170283	+	1350	hypothetical protein
wy_03011	-	3170309	3170710	-	402	nucleoside triphosphate pyrophosphohydrolase
wy_03012	*mrpA*	3170929	3173763	+	2835	Na(+)/H(+) antiporter subunit A
wy_03013	*mrpC*	3173760	3174317	+	558	Na(+)/H(+) antiporter subunit C
wy_03014	*mrpD*	3174314	3175915	+	1602	Na(+)/H(+) antiporter subunit D
wy_03015	*mnhE1*	3175912	3176484	+	573	Na(+)/H(+) antiporter subunit E1
wy_03016	-	3176481	3176741	+	261	putative monovalent cation/H+ antiporter subunit F
wy_03017	*mrpG*	3176738	3177076	+	339	Na(+)/H(+) antiporter subunit G
wy_03018	-	3176958	3178469	-	1512	PAP2 superfamily protein
wy_03019	*ybdK_2*	3178494	3179612	-	1119	Carboxylate-amine ligase YbdK
wy_03020	*sodC*	3179727	3180437	-	711	Superoxide dismutase [Cu-Zn] precursor
wy_03021	-	3180500	3181072	-	573	hypothetical protein
wy_03022	-	3181154	3181480	-	327	hypothetical protein
wy_03023	*def*	3181717	3182334	+	618	Peptide deformylase
wy_03024	-	3182346	3182585	+	240	hypothetical protein
wy_03025	*xthA*	3182616	3183416	+	801	Exodeoxyribonuclease III
wy_03026	-	3183386	3183766	-	381	hypothetical protein
wy_03027	-	3183858	3184217	-	360	hypothetical protein
wy_03028	-	3184596	3185204	-	609	hypothetical protein
wy_03029	-	3185289	3186062	-	774	hypothetical protein

wy_03030	-	3186912	3187817	+	906	hypothetical protein
wy_03031	*thiD*	3187946	3188797	+	852	Hydroxymethylpyrimidine/phosphomethylpyrimidine kinase
wy_03032	-	3188798	3188983	-	186	hypothetical protein
wy_03033	-	3189058	3189924	-	867	pH-sensitive adenylate cyclase
wy_03034	*lieA*	3189989	3191446	-	1458	Leupeptin-inactivating enzyme 1 precursor
wy_03035	-	3191480	3192298	-	819	Dienelactone hydrolase family protein
wy_03036	*cdhR_1*	3192389	3193381	-	993	HTH-type transcriptional regulator CdhR
wy_03037	-	3193465	3194217	-	753	ABC-2 family transporter protein
wy_03038	*skfE*	3194214	3195179	-	966	SkfA peptide export ATP-binding protein SkfE
wy_03039	*pipB2_3*	3195290	3195829	-	540	Secreted effector protein pipB2
wy_03040	*thiG*	3195940	3196689	-	750	Thiazole synthase
wy_03041	-	3196691	3196900	-	210	sulfur carrier protein ThiS
wy_03042	*hcnC*	3196912	3197946	-	1035	Hydrogen cyanide synthase subunit HcnC precursor
wy_03043	*thiE*	3198116	3198808	+	693	Thiamine-phosphate synthase
wy_03044	-	3198815	3199249	-	435	hypothetical protein
wy_03045	-	3199266	3200528	-	1263	Polyprenol-phosphate-mannose-dependent alpha-(1-2)-phosphatidylinositol mannoside mannosyltransferase
wy_03046	-	3200811	3201212	+	402	hypothetical protein
wy_03047	*stp_2*	3201884	3203263	+	1380	Multidrug resistance protein stp
wy_03048	-	3203486	3203896	+	411	Pyridoxamine 5'-phosphate oxidase
wy_03049	-	3203906	3204745	-	840	hypothetical protein
wy_03050	-	3204905	3206269	+	1365	hypothetical protein
wy_03051	*glnH_4*	3206266	3207249	+	984	ABC transporter glutamine-binding protein GlnH precursor
wy_03052	*pknG*	3207269	3209521	+	2253	Serine/threonine-protein kinase PknG
wy_03053	-	3209454	3209747	-	294	hypothetical protein
wy_03054	*ctaD_2*	3209750	3211510	-	1761	Cytochrome c oxidase subunit 1

wy_03055	*ackA*	3211676	3212866	-	1191	Acetate kinase
wy_03056	*pta*	3212863	3214500	-	1638	Phosphate acetyltransferase
wy_03057	*ddn_1*	3214986	3215507	+	522	Deazaflavin-dependent nitroreductase
wy_03058	*yqcF*	3215535	3216137	-	603	Antitoxin YqcF
wy_03059	*fgd_1*	3216176	3217189	-	1014	F420-dependent glucose-6-phosphate dehydrogenase
wy_03060	-	3217267	3217740	-	474	RDD family protein
wy_03061	-	3217737	3218483	-	747	hypothetical protein
wy_03062	*fbpB_1*	3218635	3219639	+	1005	Diacylglycerol acyltransferase/mycolyltransferase Ag85B precursor
wy_03063	*nagD_2*	3219636	3220451	+	816	Ribonucleotide monophosphatase NagD
wy_03064	*estB_5*	3220462	3221601	-	1140	Esterase EstB
wy_03066	-	3224274	3224720	-	447	hypothetical protein
wy_03067	-	3224799	3225221	-	423	hypothetical protein
wy_03068	*fprA_3*	3225532	3226896	+	1365	NADPH-ferredoxin reductase FprA
wy_03069	*ligI*	3226983	3227726	+	744	2-pyrone-4,6-dicarbaxylate hydrolase
wy_03070	-	3227728	3228210	-	483	hypothetical protein
wy_03071	*purT*	3228221	3229435	-	1215	Phosphoribosylglycinamide formyltransferase 2
wy_03072	-	3229477	3229902	+	426	hypothetical protein
wy_03073	*xylF_2*	3229907	3230623	-	717	2-hydroxymuconate semialdehyde hydrolase
wy_03074	-	3230628	3230948	-	321	Glyoxalase-like domain protein
wy_03075	*bbsF_3*	3231139	3232239	+	1101	Succinyl-CoA:(R)-benzylsuccinate CoA-transferase subunit BbsF
wy_03076	-	3232605	3234014	+	1410	hypothetical protein
wy_03077	-	3234063	3235232	+	1170	hypothetical protein
wy_03078	*norG*	3235291	3236772	-	1482	HTH-type transcriptional regulator NorG
wy_03079	-	3236839	3237132	+	294	hypothetical protein
wy_03080	*degU_1*	3237129	3238394	+	1266	Transcriptional regulatory protein DegU
wy_0308	-	323852	323961	+	1089	S-(hydroxymethyl)mycothiol dehydrogenase

1		9	7			
wy_03082	*baeB_2*	3239614	3240246	+	633	putative polyketide biosynthesis zinc-dependent hydrolase BaeB
wy_03083	*glnT_1*	3240835	3242199	+	1365	Glutamine synthetase 3
wy_03084	*nodM*	3242229	3243125	+	897	Glutamine--fructose-6-phosphate aminotransferase [isomerizing]
wy_03085	-	3243113	3243811	+	699	GXGXG motif protein
wy_03086	*gltB_3*	3243836	3245161	+	1326	Glutamate synthase [NADPH] large chain precursor
wy_03087	-	3245226	3245330	-	105	hypothetical protein
wy_03088	-	3245563	3246210	+	648	DNA-binding transcriptional repressor PuuR
wy_03089	*yfiY_5*	3246323	3247354	+	1032	putative siderophore-binding lipoprotein YfiY precursor
wy_03090	-	3247355	3247897	-	543	MarR family protein
wy_03091	*amt_3*	3248034	3249398	-	1365	Ammonia channel
wy_03092	*mlr*	3249592	3250794	+	1203	4-methylaminobutanoate oxidase (formaldehyde-forming)
wy_03093	-	3250808	3251452	-	645	hypothetical protein
wy_03094	*purA*	3251529	3252818	-	1290	Adenylosuccinate synthetase
wy_03095	-	3253104	3253778	+	675	hypothetical protein
wy_03096	-	3253805	3254584	+	780	Peptidase family M50
wy_03097	-	3254647	3254928	-	282	Intracellular chorismate mutase
wy_03098	*ilvE_2*	3255023	3255937	-	915	Branched-chain-amino-acid aminotransferase
wy_03099	-	3256054	3257013	-	960	HTH domain protein
wy_03100	-	3257076	3257540	+	465	hypothetical protein
wy_03101	*fabG_9*	3257537	3258460	+	924	3-oxoacyl-[acyl-carrier-protein] reductase FabG
wy_03102	*czcD*	3258483	3259409	-	927	Cadmium, cobalt and zinc/H(+)-K(+) antiporter
wy_03103	-	3259471	3260616	-	1146	hypothetical protein
wy_03104	-	3260603	3261028	-	426	hypothetical protein
wy_03105	-	3261101	3261628	+	528	hypothetical protein
wy_03106	*fba*	3261718	3262755	-	1038	Fructose-bisphosphate aldolase

wy_03107	*yqjA_1*	3262818	3263675	+	858	Inner membrane protein YqjA
wy_03108	*yqjA_2*	3263678	3264340	+	663	Inner membrane protein YqjA
wy_03109	-	3264348	3265421	-	1074	Glycosyl hydrolase family 76
wy_03110	*trmH*	3265537	3266217	-	681	tRNA (guanosine(18)-2'-O)-methyltransferase
wy_03111	-	3266235	3266843	-	609	hypothetical protein
wy_03112	*budC_1*	3266915	3267706	-	792	Diacetyl reductase [(S)-acetoin forming]
wy_03113	-	3267746	3268900	-	1155	hypothetical protein
wy_03114	-	3268900	3269829	-	930	putative metal-dependent hydrolase
wy_03115	-	3269988	3270617	+	630	Bacterial regulatory proteins, tetR family
wy_03116	-	3270645	3271091	+	447	hypothetical protein
wy_03117	-	3271088	3271825	-	738	YhhN-like protein
wy_03118	-	3272039	3272485	+	447	MarR family protein
wy_03119	*yfmO*	3272515	3273759	+	1245	Multidrug efflux protein YfmO
wy_03120	-	3273877	3274431	-	555	hypothetical protein
wy_03121	-	3274568	3275686	-	1119	hypothetical protein
wy_03122	*galK*	3275873	3277048	-	1176	Galactokinase
wy_03123	*galT*	3277045	3278202	-	1158	Galactose-1-phosphate uridylyltransferase
wy_03124	-	3278316	3278822	+	507	hypothetical protein
wy_03125	*pyrE*	3278904	3279437	-	534	Orotate phosphoribosyltransferase
wy_03126	-	3279522	3280058	-	537	LemA family protein
wy_03127	-	3280138	3280818	-	681	hypothetical protein
wy_03128	-	3280872	3281762	-	891	hypothetical protein
wy_03129	*hslR*	3281801	3282172	-	372	Heat shock protein 15
wy_03130	*pamO_2*	3282483	3284108	+	1626	Phenylacetone monooxygenase
wy_03131	-	3284170	3284568	-	399	hypothetical protein
wy_03132	-	3284663	3284806	-	144	hypothetical protein

wy_03133	*estB_6*	3284955	3285899	+	945	Extracellular esterase EstB precursor
wy_03134	*clpB_2*	3285965	3288517	-	2553	Chaperone protein ClpB
wy_03135	*dmpP*	3288761	3289924	+	1164	Phenol hydroxylase P5 protein
wy_03136	-	3290123	3291343	+	1221	hypothetical protein
wy_03137	-	3291360	3291857	+	498	SnoaL-like domain protein
wy_03138	*azoB*	3291919	3292773	+	855	NAD(P)H azoreductase
wy_03139	-	3293015	3293227	+	213	hypothetical protein
wy_03140	*xylA*	3293271	3294029	+	759	Xylene monooxygenase electron transfer component
wy_03141	*lcfB_12*	3294055	3295632	-	1578	Long-chain-fatty-acid--CoA ligase
wy_03142	*ptlF*	3295782	3296606	-	825	1-deoxy-11-beta-hydroxypentalenate dehydrogenase
wy_03143	-	3296617	3297450	-	834	Putative short-chain type dehydrogenase/reductase/MSMEI_5872
wy_03144	-	3297450	3298259	-	810	Putative short-chain type dehydrogenase/reductase/MSMEI_5872
wy_03145	*kshA_4*	3298517	3299683	+	1167	3-ketosteroid-9-alpha-monooxygenase oxygenase subunit
wy_03146	*hsaA_2*	3299726	3300907	+	1182	Flavin-dependent monooxygenase, oxygenase subunit HsaA
wy_03147	*hsaC_1*	3300952	3301884	+	933	Iron-dependent extradiol dioxygenase
wy_03148	*kdgR_3*	3302082	3302873	+	792	Pectin degradation repressor protein KdgR
wy_03149	-	3302910	3303614	-	705	Transcriptional regulator PadR-like family protein
wy_03150	-	3303765	3304016	+	252	hypothetical protein
wy_03151	*fgd1_4*	3304089	3304961	+	873	F420-dependent glucose-6-phosphate dehydrogenase
wy_03152	*rutA_3*	3304966	3305850	-	885	Pyrimidine monooxygenase RutA
wy_03153	*livF_4*	3305963	3306682	-	720	High-affinity branched-chain amino acid transport ATP-binding protein LivF
wy_03154	*lptB_3*	3306682	3307761	-	1080	Lipopolysaccharide export system ATP-binding protein LptB
wy_03155	-	3308062	3308286	+	225	hypothetical protein
wy_03156	*livH_3*	3308510	3309367	-	858	High-affinity branched-chain amino acid transport system permease protein LivH
wy_03157	*braC_2*	3309414	3310628	-	1215	Leucine-, isoleucine-, valine-, threonine-, and alanine-binding protein precursor

wy_03158	-	3311216	3311917	+	702	hypothetical protein
wy_03159	*hspR_3*	3311990	3312397	-	408	Putative heat shock protein HspR
wy_03160	*dnaJ_2*	3312431	3313603	-	1173	Chaperone protein DnaJ
wy_03161	-	3313638	3314207	-	570	heat shock protein GrpE
wy_03162	*dnaK_1*	3314204	3316045	-	1842	Chaperone protein DnaK
wy_03163	-	3316297	3316869	+	573	hypothetical protein
wy_03164	-	3316866	3318281	+	1416	hypothetical protein
wy_03165	-	3318315	3318464	-	150	hypothetical protein
wy_03166	-	3318497	3318736	-	240	hypothetical protein
wy_03167	*blaB*	3318988	3319881	-	894	Beta-lactamase regulatory protein BlaB
wy_03168	*hcaR_2*	3319878	3320774	-	897	Hca operon transcriptional activator
wy_03169	*pbpA_1*	3320898	3322706	+	1809	Penicillin-binding protein A
wy_03170	*iniC*	3322707	3324254	-	1548	Isoniazid-induced protein IniC
wy_03171	*iniA*	3324251	3326095	-	1845	Isoniazid-induced protein IniA
wy_03172	-	3326242	3328725	-	2484	hypothetical protein
wy_03173	*dnaK_2*	3329028	3330803	+	1776	Chaperone protein DnaK
wy_03174	*devR_3*	3330807	3333308	+	2502	Transcriptional regulatory protein DevR (DosR)
wy_03175	*alpha-LP*	3333391	3334767	+	1377	Alpha-lytic protease precursor
wy_03176	-	3334929	3336245	+	1317	Serine protease 2
wy_03177	-	3336345	3336980	-	636	hypothetical protein
wy_03178	-	3337499	3340729	+	3231	succinate dehydrogenase/fumarate reductase iron-sulfur subunit
wy_03179	-	3341136	3341924	+	789	hypothetical protein
wy_03180	*ptpA_3*	3342026	3343993	-	1968	Prolyl tripeptidyl peptidase precursor
wy_03181	-	3344024	3344446	+	423	Pyridoxamine 5'-phosphate oxidase
wy_03182	-	3344543	3344710	+	168	hypothetical protein
wy_03183	*alaA*	3344800	3346062	+	1263	Glutamate-pyruvate aminotransferase AlaA

wy_03184	-	3346346	3347668	+	1323	YibE/F-like protein
wy_03185	-	3347679	3348857	-	1179	hypothetical protein
wy_03186	*wzb*	3348953	3349468	+	516	Low molecular weight protein-tyrosine-phosphatase wzb
wy_03187	*ywqD*	3349436	3350869	-	1434	Tyrosine-protein kinase YwqD
wy_03188	*tycC_3*	3350932	3369654	-	18723	Tyrocidine synthase 3
wy_03189	-	3369862	3371709	+	1848	hypothetical protein
wy_03190	*rmlB*	3371795	3372721	-	927	dTDP-glucose 4,6-dehydratase
wy_03191	*rmlA*	3372873	3373742	+	870	Glucose-1-phosphate thymidylyltransferase
wy_03192	*rmlC*	3373744	3374331	+	588	dTDP-4-dehydrorhamnose 3-epimerase
wy_03193	-	3374888	3376237	-	1350	hypothetical protein
wy_03194	*oatA*	3376317	3377426	-	1110	O-acetyltransferase OatA
wy_03195	*ywqF*	3377459	3378781	-	1323	UDP-glucose 6-dehydrogenase YwqF
wy_03196	-	3378838	3380121	-	1284	hypothetical protein
wy_03197	-	3380139	3381182	-	1044	Nitroreductase
wy_03198	-	3381179	3382420	-	1242	
wy_03199	-	3382422	3383339	-	918	
wy_03200	-	3383336	3384199	-	864	
wy_03201	*wecG*	3384196	3384963	-	768	UDP-N-acetyl-D-mannosaminuronic acid transferase
wy_03202	*gumD*	3385126	3386664	-	1539	UDP-glucose:undecaprenyl-phosphate glucose-1-phosphate transferase
wy_03203	*dcd*	3387440	3388027	-	588	Deoxycytidine triphosphate deaminase
wy_03205	-	3388368	3389033	-	666	hypothetical protein
wy_03206	-	3389356	3389748	+	393	hypothetical protein
wy_03207	-	3389822	3390619	-	798	hypothetical protein
wy_03208	-	3390769	3391284	-	516	hypothetical protein
wy_03209	*sprE*	3391381	3392631	-	1251	Glutamyl endopeptidase 2
wy_03210	-	3392755	3393312	-	558	regulatory protein

wy_03211	-	3393673	3394212	+	540	hypothetical protein
wy_03212	-	3394221	3395270	+	1050	hypothetical protein
wy_03213	*ssuB_4*	3395246	3395992	+	747	Aliphatic sulfonates import ATP-binding protein SsuB
wy_03214	*nrtA*	3395989	3397161	+	1173	Nitrate transport protein NrtA precursor
wy_03215	*cmpB_2*	3397158	3397973	+	816	Bicarbonate transport system permease protein CmpB
wy_03216	-	3398168	3398470	+	303	hypothetical protein
wy_03217	-	3398528	3398701	+	174	hypothetical protein
wy_03218	-	3398914	3400320	+	1407	Putative diacylglycerol O-acyltransferase/MT0919
wy_03219	-	3400330	3401295	-	966	Excalibur calcium-binding domain protein
wy_03220	*gpr*	3401460	3402404	+	945	L-glyceraldehyde 3-phosphate reductase
wy_03221	*fabH_2*	3402465	3403481	-	1017	3-oxoacyl-[acyl-carrier-protein] synthase 3
wy_03222	-	3403861	3404985	+	1125	hypothetical protein
wy_03223	-	3405067	3405501	+	435	3-phenylpropionate dioxygenase subunit beta
wy_03224	-	3405557	3406222	+	666	tellurite resistance protein TehB
wy_03225	-	3406321	3408156	+	1836	Acyl-CoA dehydrogenase, short-chain specific
wy_03226	*lcp*	3408290	3409498	+	1209	Latex clearing protein precursor
wy_03227	-	3409616	3410320	+	705	hypothetical protein
wy_03228	-	3410323	3412215	+	1893	ABC-2 family transporter protein
wy_03229	*vanW*	3412312	3414312	-	2001	Vancomycin B-type resistance protein VanW
wy_03230	*fadI*	3414599	3415978	-	1380	3-ketoacyl-CoA thiolase
wy_03231	*fabG_10*	3416097	3417443	+	1347	3-oxoacyl-[acyl-carrier-protein] reductase FabG
wy_03232	-	3417443	3418315	+	873	MaoC like domain protein
wy_03233	*hspR_4*	3418384	3418689	-	306	Putative heat shock protein HspR
wy_03234	-	3418896	3419270	-	375	hypothetical protein
wy_03235	-	3419699	3419839	+	141	hypothetical protein
wy_03236	*whiB3_2*	3419889	3420188	+	300	Redox-responsive transcriptional regulator WhiB3

wy_03237	-	3420272	3420427	+	156	hypothetical protein
wy_03238	*fadR_2*	3420495	3421097	-	603	Fatty acid metabolism regulator protein
wy_03239	-	3421182	3422363	-	1182	Beta-hexosaminidase A precursor
wy_03240	*nhaX*	3422494	3422961	+	468	Stress response protein NhaX
wy_03241	*dhbC_1*	3423017	3424156	+	1140	Isochorismate synthase DhbC
wy_03243	*aftD*	3424350	3428741	+	4392	Alpha-(1->3)-arabinofuranosyltransferase
wy_03244	*oatA_3*	3428612	3429811	-	1200	O-acetyltransferase OatA
wy_03245	-	3430018	3431154	+	1137	hypothetical protein
wy_03246	-	3431401	3432609	-	1209	hypothetical protein
wy_03247	-	3432642	3434405	+	1764	hypothetical protein
wy_03248	*pimB_2*	3434322	3435551	-	1230	GDP-mannose-dependent alpha-(1-6)-phosphatidylinositol monomannoside mannosyltransferase
wy_03249	-	3435708	3436463	+	756	putative methyltransferase
wy_03250	*nanK*	3436609	3437496	+	888	N-acetylmannosamine kinase
wy_03251	*geoB_2*	3437583	3439031	+	1449	Geranial dehydrogenase
wy_03252	-	3439134	3440087	+	954	zinc transporter ZitB
wy_03253	-	3440490	3442151	+	1662	hypothetical protein
wy_03254	-	3442214	3442822	-	609	hypothetical protein
wy_03255	*hsdA_1*	3442948	3443802	-	855	3-alpha-hydroxysteroid dehydrogenase/carbonyl reductase
wy_03256	*cysE*	3443959	3444537	-	579	Serine acetyltransferase
wy_03257	*cysK1*	3444543	3445475	-	933	O-acetylserine sulfhydrylase
wy_03258	*pckG*	3445713	3447542	-	1830	Phosphoenolpyruvate carboxykinase [GTP]
wy_03259	-	3447831	3448142	-	312	hypothetical protein
wy_03260	-	3448176	3449609	-	1434	hypothetical protein
wy_03261	-	3449606	3450325	-	720	hypothetical protein
wy_03262	*trmB*	3450534	3451355	+	822	tRNA (guanine-N(7)-)-methyltransferase

wy_03263	-	3451352	3452044	+	693	hypothetical protein
wy_03264	*ydfJ_3*	3452056	3454758	+	2703	Membrane protein YdfJ
wy_03265	-	3454759	3455409	-	651	hypothetical protein
wy_03266	*tqsA_2*	3455446	3456651	-	1206	AI-2 transport protein TqsA
wy_03267	*cyaB_2*	3456819	3458276	+	1458	Adenylate cyclase 2
wy_03268	-	3458278	3459453	+	1176	hypothetical protein
wy_03269	*lldR*	3459463	3460149	-	687	Putative L-lactate dehydrogenase operon regulatory protein
wy_03270	*leuA_1*	3460318	3462018	+	1701	2-isopropylmalate synthase
wy_03271	-	3462077	3462694	-	618	hypothetical protein
wy_03272	-	3462997	3463548	+	552	hypothetical protein
wy_03273	*pcpB*	3463626	3465053	-	1428	Pentachlorophenol 4-monooxygenase
wy_03274	-	3465480	3465911	+	432	hypothetical protein
wy_03275	-	3466001	3466405	+	405	hypothetical protein
wy_03276	*dtpT*	3466453	3467919	+	1467	Di-/tripeptide transporter
wy_03277	*pspB_3*	3468068	3468667	-	600	Putative phosphoserine phosphatase 2
wy_03278	*fabG_11*	3468670	3469407	-	738	3-oxoacyl-[acyl-carrier-protein] reductase FabG
wy_03279	-	3469404	3470432	-	1029	Putative aminoglycoside phosphotransferase
wy_03280	*bbsG_1*	3470429	3471643	-	1215	(R)-benzylsuccinyl-CoA dehydrogenase
wy_03281	-	3471794	3472852	+	1059	hypothetical protein
wy_03282	*kstR2_9*	3472877	3473488	-	612	HTH-type transcriptional repressor KstR2
wy_03283	-	3473532	3475214	-	1683	hypothetical protein
wy_03284	*pbpA_2*	3475347	3477083	-	1737	Penicillin-binding protein A
wy_03285	-	3477198	3477773	+	576	hypothetical protein
wy_03286	*mtcA1*	3477781	3478272	-	492	Beta-carbonic anhydrase 1
wy_03287	*yheS_1*	3478544	3480136	+	1593	putative ABC transporter ATP-binding protein YheS
wy_03288	-	3480361	3480636	+	276	hypothetical protein

wy_03289	*nikMN*	3480730	3481458	+	729	Fused nickel transport protein NikMN
wy_03290	-	3481455	3481805	+	351	cobalt transport protein CbiN
wy_03291	*ecfT*	3481807	3482565	+	759	Energy-coupling factor transporter transmembrane protein EcfT
wy_03292	*cbiO*	3482567	3483319	+	753	Cobalt import ATP-binding protein CbiO
wy_03293	*hspR_5*	3483342	3483746	-	405	Putative heat shock protein HspR
wy_03294	-	3484199	3485020	-	822	hypothetical protein
wy_03295	*hypD*	3485017	3486147	-	1131	Hydrogenase expression/formation protein HypD
wy_03296	*hypC*	3486176	3486448	-	273	Hydrogenase isoenzymes formation protein HypC
wy_03297	*hypE*	3486467	3487564	-	1098	Hydrogenase expression/formation protein HypE
wy_03298	*gmhA1*	3487569	3488270	-	702	Phosphoheptose isomerase 1
wy_03299	-	3488267	3489010	-	744	HupF/HypC family protein
wy_03300	*hypF*	3489007	3491385	-	2379	Carbamoyltransferase HypF
wy_03301	-	3491382	3491648	-	267	hydrogenase 2 accessory protein HypG
wy_03302	*hybD*	3491652	3492149	-	498	Hydrogenase 2 maturation protease
wy_03303	-	3492154	3492273	-	120	hypothetical protein
wy_03304	-	3492270	3493694	-	1425	hypothetical protein
wy_03305	-	3493691	3494332	-	642	hypothetical protein
wy_03306	-	3494329	3495000	-	672	hypothetical protein
wy_03307	*nfuA*	3494997	3495932	-	936	Fe/S biogenesis protein NfuA
wy_03308	-	3495940	3497733	-	1794	Periplasmic [NiFeSe] hydrogenase large subunit
wy_03309	-	3497774	3498859	-	1086	Periplasmic [NiFeSe] hydrogenase small subunit precursor
wy_03310	*hypB*	3498977	3499786	-	810	Hydrogenase isoenzymes nickel incorporation protein HypB
wy_03311	-	3499789	3500118	-	330	hydrogenase nickel incorporation protein
wy_03312	-	3500205	3500558	-	354	hypothetical protein
wy_03313	*dehH1_1*	3500613	3501404	+	792	Haloacetate dehalogenase H-1
wy_0331	*yvoA*	350144	350221	-	771	HTH-type transcriptional repressor YvoA

4		7	7			
wy_03315	*mcl2*	3502299	3503141	-	843	(3S)-malyl-CoA thioesterase
wy_03316	*gdh_2*	3503227	3506646	-	3420	NAD-specific glutamate dehydrogenase
wy_03317	*hutU*	3506913	3508589	+	1677	Urocanate hydratase
wy_03318	*mmgC_5*	3508909	3510072	+	1164	Acyl-CoA dehydrogenase
wy_03319	*bbsF_4*	3510094	3511242	+	1149	Succinyl-CoA:(R)-benzylsuccinate CoA-transferase subunit BbsF
wy_03320	*kdgR_4*	3511293	3511958	+	666	Transcriptional regulator KdgR
wy_03321	*allR_1*	3512085	3512873	+	789	HTH-type transcriptional repressor AllR
wy_03322	*hutH*	3513027	3514586	+	1560	Histidine ammonia-lyase
wy_03323	*pat_1*	3514583	3515659	+	1077	Putative phenylalanine aminotransferase
wy_03324	-	3515692	3517056	+	1365	putative FAD-linked oxidoreductase
wy_03325	-	3517237	3518679	+	1443	allantoin permease
wy_03326	*hutI*	3518737	3519930	+	1194	Imidazolonepropionase
wy_03327	*hutG*	3519952	3520917	+	966	Formimidoylglutamase
wy_03328	-	3521240	3522832	+	1593	Divergent AAA domain protein
wy_03329	-	3522935	3523165	+	231	hypothetical protein
wy_03330	-	3523237	3523953	-	717	mycothiol-dependent maleylpyruvate isomerase
wy_03331	-	3523962	3524783	-	822	Ureidoglycolate lyase
wy_03332	*nagI*	3524788	3525858	-	1071	Gentisate 1,2-dioxygenase
wy_03333	*rutA_4*	3526098	3527231	+	1134	Pyrimidine monooxygenase RutA
wy_03334	-	3527250	3527846	+	597	NAD(P)H-dependent FAD/FMN reductase
wy_03335	*aldA*	3527843	3529303	+	1461	Putative aldehyde dehydrogenase AldA
wy_03336	-	3529359	3530360	-	1002	transcriptional regulator EutR
wy_03337	*allR_2*	3530496	3531329	-	834	HTH-type transcriptional repressor AllR
wy_03338	-	3531419	3532630	+	1212	3-hydroxybenzoate 6-hydroxylase
wy_03339	*fprA_4*	3532591	3533997	-	1407	NADPH-ferredoxin reductase FprA
wy_0334	-	353409	353549	+	1401	putative FAD-linked oxidoreductase

0		9	9			
wy_03341	*mhpE*	3535675	3536706	-	1032	4-hydroxy-2-oxovalerate aldolase
wy_03342	*mhpF*	3536703	3537647	-	945	Acetaldehyde dehydrogenase
wy_03343	*mhpD*	3537644	3538468	-	825	2-keto-4-pentenoate hydratase
wy_03344	*kdgR_5*	3538538	3539299	+	762	Pectin degradation repressor protein KdgR
wy_03345	-	3539300	3540346	-	1047	leucine-responsive transcriptional regulator
wy_03346	*yxeP_3*	3540442	3541647	+	1206	putative hydrolase YxeP
wy_03347	*mmgC_6*	3541694	3542938	-	1245	Acyl-CoA dehydrogenase
wy_03348	*ohrR_1*	3543095	3543580	+	486	Organic hydroperoxide resistance transcriptional regulator
wy_03349	-	3543617	3544009	+	393	hypothetical protein
wy_03350	*gloB_2*	3544017	3545012	-	996	Hydroxyacylglutathione hydrolase
wy_03351	-	3545130	3546296	-	1167	hypothetical protein
wy_03352	*sigK_2*	3546507	3547778	-	1272	ECF RNA polymerase sigma factor SigK
wy_03353	-	3547785	3548129	-	345	YCII-related domain protein
wy_03354	-	3548230	3549150	-	921	Soluble epoxide hydrolase
wy_03355	*ytpA*	3549222	3550058	-	837	Phospholipase YtpA
wy_03356	*garA_2*	3550164	3550784	+	621	Glycogen accumulation regulator GarA
wy_03357	*sigD_2*	3550789	3551406	-	618	ECF RNA polymerase sigma factor SigD
wy_03358	*sigG*	3552056	3552541	+	486	ECF RNA polymerase sigma factor SigG
wy_03359	-	3552563	3552967	-	405	Glyoxalase-like domain protein
wy_03360	-	3553140	3553385	+	246	hypothetical protein
wy_03361	*cpo_3*	3553599	3554483	+	885	Non-heme chloroperoxidase
wy_03362	-	3554438	3554884	-	447	hypothetical protein
wy_03363	*fadK_2*	3554881	3556539	-	1659	Short-chain-fatty-acid--CoA ligase
wy_03364	*pdhR_4*	3556656	3557474	+	819	Pyruvate dehydrogenase complex repressor
wy_03365	-	3557485	3558735	-	1251	Amidohydrolase
wy_0336	*novR_1*	355882	355961	-	795	Decarboxylase NovR

6		1	5			
wy_03367	-	3559805	3560392	-	588	HD domain protein
wy_03368	*kstR2_10*	3560526	3561263	-	738	HTH-type transcriptional repressor KstR2
wy_03369	-	3561260	3561823	-	564	HD domain protein
wy_03370	*kgtP_1*	3562099	3563448	+	1350	Alpha-ketoglutarate permease
wy_03371	*malT*	3563608	3566169	+	2562	HTH-type transcriptional regulator MalT
wy_03372	*yhhW_3*	3566264	3567229	+	966	Quercetin 2,3-dioxygenase
wy_03373	*yhhW_4*	3567435	3568199	+	765	Quercetin 2,3-dioxygenase
wy_03374	-	3568348	3569271	+	924	Thioesterase superfamily protein
wy_03375	-	3569283	3570140	+	858	Dioxygenase
wy_03376	-	3570241	3571263	+	1023	Dioxygenase
wy_03377	*pepO*	3571346	3573304	+	1959	Neutral endopeptidase
wy_03378	-	3573341	3574399	-	1059	hypothetical protein
wy_03379	-	3574496	3574822	-	327	hypothetical protein
wy_03380	*lppS_3*	3574894	3575889	-	996	Putative L,D-transpeptidase LppS precursor
wy_03381	*fprA_5*	3576015	3577700	-	1686	NADPH-ferredoxin reductase FprA
wy_03382	-	3577838	3578044	-	207	hypothetical protein
wy_03383	*pbpX_1*	3578058	3579308	-	1251	Putative penicillin-binding protein PbpX
wy_03384	*ykoU*	3579435	3580481	-	1047	putative ATP-dependent DNA ligase YkoU
wy_03385	-	3580485	3581525	-	1041	Putative DNA ligase-like protein/MT0965
wy_03386	-	3581619	3582143	-	525	OsmC-like protein
wy_03387	*corA_3*	3582340	3583416	-	1077	Magnesium transport protein CorA
wy_03388	-	3583744	3584271	-	528	UDP-N-acetylglucosamine acyltransferase
wy_03389	*lutR_3*	3584315	3585022	-	708	HTH-type transcriptional regulator LutR
wy_03390	*mdtL*	3585095	3586369	+	1275	Multidrug resistance protein MdtL
wy_03391	*liaR_5*	3586359	3587351	-	993	Transcriptional regulatory protein LiaR
wy_0339	-	358736	358896	+	1602	Long-chain-fatty-acid--CoA ligase

2		2	3			
wy_03393	*adh_4*	3589106	3590128	+	1023	NADP-dependent isopropanol dehydrogenase
wy_03394	*mmgC_7*	3590138	3592339	-	2202	Acyl-CoA dehydrogenase
wy_03395	-	3592344	3593213	-	870	Putative short-chain type dehydrogenase/reductase
wy_03396	-	3593262	3594125	-	864	MaoC like domain protein
wy_03397	-	3594130	3594561	-	432	Toxin/MT0934
wy_03398	*pcaF_2*	3594596	3595786	-	1191	Beta-ketoadipyl-CoA thiolase
wy_03399	-	3595888	3596547	+	660	hypothetical protein
wy_03400	*lgrD_1*	3596566	3620988	-	24423	Linear gramicidin synthase subunit D
wy_03401	-	3621121	3621918	+	798	DNA-binding transcriptional repressor AcrR
wy_03402	*acdA_7*	3622078	3623235	+	1158	Acyl-CoA dehydrogenase
wy_03403	-	3623403	3623537	+	135	hypothetical protein
wy_03404	*fadB2_2*	3623715	3624548	+	834	3-hydroxybutyryl-CoA dehydrogenase
wy_03405	-	3624694	3624828	-	135	hypothetical protein
wy_03406	-	3625051	3625725	-	675	hypothetical protein
wy_03407	-	3625815	3626033	-	219	hypothetical protein
wy_03408	*kshA_5*	3626036	3627193	-	1158	3-ketosteroid-9-alpha-monooxygenase oxygenase subunit
wy_03409	-	3627326	3628123	-	798	hypothetical protein
wy_03410	*amt_4*	3628187	3629527	-	1341	Ammonia channel
wy_03411	*kstR2_11*	3629650	3630321	-	672	HTH-type transcriptional repressor KstR2
wy_03412	*glnT_2*	3630447	3631808	+	1362	Glutamine synthetase 3
wy_03413	*metX_2*	3631810	3632838	+	1029	Homoserine O-acetyltransferase
wy_03414	-	3632801	3633310	-	510	hypothetical protein
wy_03415	*stp_3*	3633557	3634960	+	1404	Multidrug resistance protein stp
wy_03416	*nylA_1*	3634968	3636077	-	1110	6-aminohexanoate-cyclic-dimer hydrolase
wy_03417	*oppF_1*	3636490	3637479	-	990	Oligopeptide transport ATP-binding protein OppF
wy_0341	*oppD_1*	363747	363854	-	1068	Oligopeptide transport ATP-binding protein

8		9	6			OppD
wy_03419	*gsiD_2*	3638543	3639649	-	1107	Glutathione transport system permease protein GsiD
wy_03420	*dppB_1*	3639642	3640595	-	954	Dipeptide transport system permease protein DppB
wy_03421	*gsiB_2*	3640660	3642273	-	1614	Glutathione-binding protein GsiB precursor
wy_03422	*nfdA_3*	3642306	3643895	-	1590	N-substituted formamide deformylase precursor
wy_03423	*phnR*	3644109	3644819	-	711	Putative transcriptional regulator of 2-aminoethylphosphonate degradation operons
wy_03424	*ddpA*	3645347	3646951	+	1605	putative D,D-dipeptide-binding periplasmic protein DdpA precursor
wy_03425	*dan_2*	3647031	3648755	-	1725	D-aminoacylase
wy_03426	*nlhH_6*	3648748	3649728	-	981	Carboxylesterase NlhH
wy_03427	*kstR_3*	3649860	3650486	-	627	HTH-type transcriptional repressor KstR
wy_03428	*pamO_3*	3650587	3652200	+	1614	Phenylacetone monooxygenase
wy_03429	*guaD_2*	3652219	3652686	-	468	Guanine deaminase
wy_03430	*cynS*	3652723	3653193	-	471	Cyanate hydratase
wy_03431	*cynT_1*	3653228	3653794	-	567	Carbonic anhydrase 1
wy_03432	*cynR*	3653906	3654853	+	948	HTH-type transcriptional regulator CynR
wy_03433	*ilvD_2*	3654878	3656599	+	1722	Dihydroxy-acid dehydratase
wy_03434	*kgtP_2*	3656619	3657920	-	1302	Alpha-ketoglutarate permease
wy_03435	*fadD*	3657968	3659614	-	1647	Long-chain-fatty-acid--CoA ligase
wy_03436	-	3659867	3660523	-	657	hypothetical protein
wy_03437	-	3660708	3661469	-	762	hypothetical protein
wy_03438	-	3661593	3662630	+	1038	pH-sensitive adenylate cyclase
wy_03439	*kstR_4*	3662556	3663293	-	738	HTH-type transcriptional repressor KstR
wy_03440	*qacA_4*	3663366	3664931	+	1566	Antiseptic resistance protein
wy_03441	*oppF_2*	3664937	3665611	-	675	Oligopeptide transport ATP-binding protein OppF
wy_03442	-	3665601	3665744	-	144	hypothetical protein
wy_03443	*oppD_2*	3665838	3666266	-	429	Oligopeptide transport ATP-binding protein OppD

wy_03444	-	3666641	3666799	-	159	hypothetical protein
wy_03445	*gsiD_3*	3666786	3667679	-	894	Glutathione transport system permease protein GsiD
wy_03446	*dppB_2*	3667676	3668617	-	942	Dipeptide transport system permease protein DppB
wy_03447	*gsiB_3*	3668657	3670234	-	1578	Glutathione-binding protein GsiB precursor
wy_03448	-	3670606	3672246	-	1641	Long-chain-fatty-acid--CoA ligase FadD13
wy_03449	*fabG_12*	3672294	3673118	-	825	3-oxoacyl-[acyl-carrier-protein] reductase FabG
wy_03450	*fadA_3*	3673118	3674266	-	1149	Putative acyltransferase
wy_03451	*mdlB*	3674346	3675566	-	1221	(S)-mandelate dehydrogenase
wy_03452	*ghrA*	3675563	3676465	-	903	Glyoxylate/hydroxypyruvate reductase A
wy_03453	-	3676462	3676770	-	309	hypothetical protein
wy_03454	*novR_2*	3676767	3677522	-	756	Decarboxylase NovR
wy_03455	*fac-dex*	3677571	3678443	-	873	Fluoroacetate dehalogenase
wy_03456	-	3678455	3679660	-	1206	Amidohydrolase
wy_03457	*iclR_1*	3679767	3680501	+	735	Acetate operon repressor
wy_03458	-	3680632	3681549	+	918	P-aminobenzoate N-oxygenase AurF
wy_03459	*fadB_3*	3681826	3682617	+	792	putative enoyl-CoA hydratase
wy_03460	*ssuA_6*	3682652	3683632	+	981	Putative aliphatic sulfonates-binding protein precursor
wy_03461	*bbsF_5*	3683759	3686098	+	2340	Succinyl-CoA:(R)-benzylsuccinate CoA-transferase subunit BbsF
wy_03462	*adh_5*	3686157	3687104	+	948	Alcohol dehydrogenase
wy_03463	*sauT*	3687085	3688644	+	1560	putative sulfoacetate--CoA ligase
wy_03464	*kdgR_6*	3688654	3689544	-	891	Pectin degradation repressor protein KdgR
wy_03465	-	3689683	3690534	-	852	Soluble epoxide hydrolase
wy_03466	*fdxA_2*	3690534	3690854	-	321	Ferredoxin
wy_03467	*yumC*	3690858	3691940	-	1083	Ferredoxin--NADP reductase 2
wy_03468	*novR_3*	3692024	3692806	-	783	Decarboxylase NovR
wy_03469	*ssuC_5*	3692840	3693673	-	834	Putative aliphatic sulfonates transport permease protein SsuC

wy_0347 0	*cmpD_2*	369367 0	369447 9	-	810	Bicarbonate transport ATP-binding protein CmpD
wy_0347 1	*tauA*	369447 6	369545 6	-	981	Taurine-binding periplasmic protein precursor
wy_0347 2	*tkrA*	369564 8	369659 5	-	948	Glyoxylate/hydroxypyruvate reductase B
wy_0347 3	*hsaD_3*	369667 5	369754 7	-	873	4,5:9,10-diseco-3-hydroxy-5,9, 17-trioxoandrosta-1(10),2-diene-4-oate hydrolase
wy_0347 4	*mhpB_3*	369758 3	369853 6	-	954	2,3-dihydroxyphenylpropionate/2, 3-dihydroxicinnamic acid 1,2-dioxygenase
wy_0347 5	*naaA_1*	369853 6	369987 9	-	1344	5-nitroanthranilic acid aminohydrolase
wy_0347 6	-	369987 6	369999 2	-	117	hypothetical protein
wy_0347 7	*carAa_1*	370000 4	370130 8	-	1305	Carbazole 1,9a-dioxygenase, terminal oxygenase component CarAa
wy_0347 8	-	370140 3	370180 4	-	402	hypothetical protein
wy_0347 9	-	370180 1	370228 6	-	486	hypothetical protein
wy_0348 0	*hsaD_4*	370238 9	370324 0	+	852	4,5:9,10-diseco-3-hydroxy-5,9, 17-trioxoandrosta-1(10),2-diene-4-oate hydrolase
wy_0348 1	*bcr_3*	370323 7	370449 3	+	1257	Bicyclomycin resistance protein
wy_0348 2	*kdgR_7*	370445 6	370519 9	-	744	Pectin degradation repressor protein KdgR
wy_0348 3	*serA_2*	370531 6	370628 4	-	969	D-3-phosphoglycerate dehydrogenase
wy_0348 4	*hdhA_3*	370629 0	370705 1	-	762	7-alpha-hydroxysteroid dehydrogenase
wy_0348 5	*fadH_2*	370723 3	370800 9	+	777	putative 2,4-dienoyl-CoA reductase
wy_0348 6	-	370808 1	370889 6	-	816	MaoC like domain protein
wy_0348 7	-	370892 2	371010 0	-	1179	lipid-transfer protein
wy_0348 8	-	371010 0	371054 6	-	447	MaoC like domain protein
wy_0348 9	*acdA_8*	371054 3	371165 5	-	1113	Acyl-CoA dehydrogenase
wy_0349 0	-	371165 5	371265 3	-	999	hypothetical protein
wy_0349 1	-	371265 0	371381 6	-	1167	Acyl-CoA dehydrogenase fadE12
wy_0349 2	*fabG_13*	371399 4	371483 6	-	843	3-oxoacyl-[acyl-carrier-protein] reductase FabG
wy_0349 3	-	371483 8	371650 2	-	1665	Long-chain-fatty-acid--CoA ligase FadD17
wy_0349 5	-	371844 1	371892 3	+	483	hypothetical protein
wy_0349	*rutA_5*	371900	371992	+	915	Pyrimidine monooxygenase RutA

6		9	3			
wy_03497	-	3719955	3720683	+	729	Helix-turn-helix domain protein
wy_03498	-	3720620	3721009	-	390	Glyoxalase-like domain protein
wy_03499	*ykuT_2*	3721064	3721765	-	702	putative MscS family protein YkuT
wy_03500	-	3722148	3722462	+	315	hypothetical protein
wy_03501	*budC_2*	3722470	3723258	-	789	Diacetyl reductase [(S)-acetoin forming]
wy_03502	-	3723348	3723998	-	651	hypothetical protein
wy_03503	*phaJ*	3723995	3724867	-	873	(R)-specific enoyl-CoA hydratase
wy_03504	*paaJ_2*	3724872	3726080	-	1209	3-oxoadipyl-CoA/3-oxo-5,6-dehydrosuberyl-CoA thiolase
wy_03505	*acdA_9*	3726092	3728296	-	2205	Acyl-CoA dehydrogenase
wy_03506	*fadA_4*	3728399	3729562	-	1164	3-ketoacyl-CoA thiolase
wy_03507	*kstR_5*	3729856	3730488	-	633	HTH-type transcriptional repressor KstR
wy_03508	*hsaA_3*	3730710	3731876	-	1167	Flavin-dependent monooxygenase, oxygenase subunit HsaA
wy_03509	*kshA_6*	3732152	3733309	+	1158	3-ketosteroid-9-alpha-monooxygenase oxygenase subunit
wy_03510	-	3733290	3733553	+	264	hypothetical protein
wy_03511	*fabG_14*	3733582	3734373	+	792	3-oxoacyl-[acyl-carrier-protein] reductase FabG
wy_03512	*baiE_2*	3734458	3735351	+	894	Bile acid 7-alpha dehydratase
wy_03513	-	3735495	3736193	+	699	hypothetical protein
wy_03514	-	3736272	3737087	-	816	hypothetical protein
wy_03515	*fbpA_6*	3737201	3738385	+	1185	Diacylglycerol acyltransferase/mycolyltransferase Ag85A precursor
wy_03516	*hsaC_2*	3738480	3739385	-	906	Iron-dependent extradiol dioxygenase
wy_03517	*hsaA_4*	3739426	3740598	-	1173	Flavin-dependent monooxygenase, oxygenase subunit HsaA
wy_03518	*ksdD_3*	3741041	3742729	-	1689	3-oxosteroid 1-dehydrogenase
wy_03519	*hsaD_5*	3742744	3743610	-	867	4,5:9,10-diseco-3-hydroxy-5,9, 17-trioxoandrosta-1(10),2-diene-4-oate hydrolase
wy_03520	*hsaB_1*	3743866	3744390	+	525	Flavin-dependent monooxygenase, reductase subunit HsaB

wy_03521	*kdgR_8*	3744487	3745380	-	894	Transcriptional regulator KdgR
wy_03522	*limB_1*	3745656	3746537	+	882	Limonene 1,2-monooxygenase
wy_03523	-	3746647	3747132	+	486	SnoaL-like domain protein
wy_03524	-	3747199	3748818	-	1620	Long-chain-fatty-acid--CoA ligase FadD19
wy_03525	-	3749104	3750045	+	942	Putative short-chain type dehydrogenase/reductase
wy_03526	*fabG_15*	3750065	3750817	+	753	3-oxoacyl-[acyl-carrier-protein] reductase FabG
wy_03527	*choB*	3750953	3752608	+	1656	Cholesterol oxidase precursor
wy_03528	-	3752742	3753521	+	780	27 kDa antigen Cfp30B
wy_03529	-	3753531	3754448	-	918	Amidohydrolase
wy_03530	-	3754613	3755752	+	1140	NADH oxidase
wy_03531	-	3755866	3756306	+	441	hypothetical protein
wy_03532	-	3756355	3757608	-	1254	Major Facilitator Superfamily protein
wy_03533	*gltR_1*	3757733	3758674	+	942	HTH-type transcriptional regulator GltR
wy_03534	-	3758775	3760073	-	1299	HNH endonuclease
wy_03535	-	3760571	3761002	+	432	hypothetical protein
wy_03536	*rnhA*	3761122	3761676	-	555	Ribonuclease HI
wy_03537	-	3761750	3762682	+	933	hypothetical protein
wy_03538	*ycdF_1*	3762722	3763525	-	804	Glucose 1-dehydrogenase 2
wy_03539	-	3763518	3764561	-	1044	D-arabitol-phosphate dehydrogenase
wy_03540	*limB_2*	3764558	3765724	-	1167	Limonene 1,2-monooxygenase
wy_03541	*devR_4*	3765858	3766628	+	771	Transcriptional regulatory protein DevR (DosR)
wy_03542	*novJ*	3766601	3767362	-	762	Short-chain reductase protein NovJ
wy_03543	-	3767422	3767871	-	450	hypothetical protein
wy_03544	-	3768010	3768510	+	501	hypothetical protein
wy_03545	*calB*	3768681	3770117	+	1437	Coniferyl aldehyde dehydrogenase
wy_03546	*linA*	3770185	3770637	+	453	Gamma-hexachlorocyclohexane dehydrochlorinase

wy_03547	*kstR2_12*	3770780	3771418	+	639	HTH-type transcriptional repressor KstR2
wy_03548	*baiE_3*	3771539	3772054	+	516	Bile acid 7-alpha dehydratase
wy_03549	*baiE_4*	3772126	3772650	+	525	Bile acid 7-alpha dehydratase
wy_03550	-	3772647	3773795	+	1149	NADH oxidase
wy_03551	*adh_6*	3773869	3774906	-	1038	Alcohol dehydrogenase
wy_03552	-	3774884	3775849	-	966	Alpha/beta hydrolase family protein
wy_03553	*ethR_3*	3775846	3776481	-	636	HTH-type transcriptional regulator EthR
wy_03554	*pamO_4*	3776522	3777922	-	1401	Phenylacetone monooxygenase
wy_03555	*caiD_5*	3778028	3778777	-	750	Carnitinyl-CoA dehydratase
wy_03556	-	3779194	3779505	+	312	hypothetical protein
wy_03557	*yvdT*	3779610	3780245	+	636	putative HTH-type transcriptional regulator YvdT
wy_03558	*scoB_3*	3780283	3780933	-	651	putative succinyl-CoA:3-ketoacid coenzyme A transferase subunit B
wy_03559	*scoA_3*	3780930	3781676	-	747	putative succinyl-CoA:3-ketoacid coenzyme A transferase subunit A
wy_03560	-	3781673	3782857	-	1185	Fumarylacetoacetate (FAA) hydrolase family protein
wy_03561	-	3782854	3783795	-	942	hypothetical protein
wy_03562	-	3783762	3784961	-	1200	homogentisate 1,2-dioxygenase
wy_03563	-	3785312	3785887	-	576	transcriptional repressor MprA
wy_03564	-	3786147	3786830	+	684	DNA-binding transcriptional activator MhpR
wy_03565	*thcB*	3787225	3789531	+	2307	Cytochrome P450 116
wy_03566	*yhjE_2*	3789573	3790922	+	1350	Inner membrane metabolite transport protein YhjE
wy_03567	*dehH1_2*	3791052	3791813	+	762	Haloacetate dehalogenase H-1
wy_03568	-	3791903	3792391	-	489	Putative lumazine-binding protein
wy_03569	*proP_2*	3792457	3793812	-	1356	Proline/betaine transporter
wy_03570	*ifcA_4*	3793855	3795315	-	1461	Fumarate reductase flavoprotein subunit precursor
wy_03571	*cpnA_1*	3795312	3796124	-	813	Cyclopentanol dehydrogenase
wy_03572	*ycdF_2*	3796117	3796953	-	837	Glucose 1-dehydrogenase 2

wy_03573	-	3797024	3797473	-	450	Ring hydroxylating beta subunit
wy_03574	*kipR_4*	3797609	3798349	+	741	HTH-type transcriptional regulator KipR
wy_03575	*exuT_2*	3798370	3799587	-	1218	Hexuronate transporter
wy_03576	*mhpA_2*	3799580	3801496	-	1917	3-(3-hydroxy-phenyl)propionate/3-hydroxycinnamic acid hydroxylase
wy_03577	*yhjE_3*	3801501	3802826	-	1326	Inner membrane metabolite transport protein YhjE
wy_03578	*oxyR_2*	3803081	3804019	-	939	Hydrogen peroxide-inducible genes activator
wy_03579	*mmgC_8*	3804137	3806107	+	1971	Acyl-CoA dehydrogenase
wy_03580	-	3806210	3807067	+	858	short chain dehydrogenase
wy_03581	*yedI*	3807075	3807911	-	837	Inner membrane protein YedI
wy_03582	*polA_3*	3808184	3809749	+	1566	DNA polymerase I, thermostable
wy_03583	*tlyC_2*	3809873	3811141	-	1269	Hemolysin C
wy_03584	*polS*	3811279	3812022	+	744	Sorbitol dehydrogenase
wy_03585	*ydfJ_4*	3812143	3814368	+	2226	Membrane protein YdfJ
wy_03586	*katA_1*	3814557	3816071	+	1515	Catalase
wy_03587	-	3816140	3817519	+	1380	hypothetical protein
wy_03588	-	3817516	3818913	+	1398	PQQ enzyme repeat protein
wy_03589	*alkA*	3818928	3819788	-	861	DNA-3-methyladenine glycosylase 2
wy_03590	*alkB_2*	3819785	3820450	-	666	Alpha-ketoglutarate-dependent dioxygenase AlkB
wy_03591	-	3820451	3820714	-	264	hypothetical protein
wy_03592	-	3820722	3821435	-	714	hypothetical protein
wy_03593	*ogt_2*	3821432	3822019	-	588	Methylated-DNA--protein-cysteine methyltransferase
wy_03594	*sigH_2*	3822016	3822522	-	507	ECF RNA polymerase sigma factor SigH
wy_03595	*ogt_3*	3822569	3823072	-	504	Methylated-DNA--protein-cysteine methyltransferase
wy_03596	-	3823130	3823735	+	606	hypothetical protein
wy_03597	-	3823770	3824273	+	504	hypothetical protein
wy_03598	*lrpC_2*	3824270	3824752	-	483	HTH-type transcriptional regulator LrpC

wy_03599	-	3824760	3825611	-	852	Universal stress protein/MT2698
wy_03600	-	3825712	3827172	-	1461	Putative diacylglycerol O-acyltransferase/MT3172
wy_03601	*hpd*	3827473	3828648	+	1176	4-hydroxyphenylpyruvate dioxygenase
wy_03602	-	3828717	3829529	-	813	putative NAD-dependent oxidoreductase
wy_03604	-	3830019	3830492	+	474	hypothetical protein
wy_03605	*lon2*	3830492	3831154	+	663	Lon protease 2
wy_03606	-	3831216	3831662	+	447	Polyketide cyclase / dehydrase and lipid transport
wy_03607	*lipF*	3831664	3832638	-	975	Carboxylesterase LipF
wy_03608	-	3832723	3833874	-	1152	Type I phosphodiesterase / nucleotide pyrophosphatase
wy_03609	-	3834006	3834956	+	951	A-factor biosynthesis hotdog domain protein
wy_03610	*arpA_4*	3834926	3835567	-	642	A-factor receptor protein
wy_03611	-	3835777	3836592	+	816	3-beta hydroxysteroid dehydrogenase/isomerase family protein
wy_03612	*gpsA_2*	3836627	3837634	+	1008	Glycerol-3-phosphate dehydrogenase [NAD(P)+]
wy_03613	*hapE_5*	3837724	3839217	+	1494	4-hydroxyacetophenone monooxygenase
wy_03614	-	3839410	3840171	+	762	Alpha/beta hydrolase family protein
wy_03615	-	3840293	3841555	+	1263	hypothetical protein
wy_03616	*leuA_2*	3841732	3842208	+	477	2-isopropylmalate synthase
wy_03617	*blc*	3842294	3842914	+	621	Outer membrane lipoprotein Blc precursor
wy_03618	-	3843024	3844508	-	1485	3 beta-hydroxysteroid dehydrogenase/Delta 5-->4-isomerase
wy_03619	-	3844530	3845183	+	654	Alpha/beta hydrolase family protein
wy_03620	*limC_1*	3845205	3846038	-	834	(-)-trans-carveol dehydrogenase
wy_03621	-	3846224	3847342	+	1119	hypothetical protein
wy_03622	*clpC1_1*	3847355	3848125	-	771	ATP-dependent Clp protease ATP-binding subunit ClpC1
wy_03623	*fgd_2*	3848221	3849075	+	855	F420-dependent glucose-6-phosphate dehydrogenase
wy_03624	*mauF*	3849122	3849973	-	852	Methylamine utilization protein MauF
wy_0362	-	385001	385033	-	327	hypothetical protein

5		2	8			
wy_0362 6	*mauC_2*	385033 5	385069 1	-	357	Amicyanin precursor
wy_0362 7	*copA_4*	385070 3	385230 1	-	1599	Copper resistance protein A precursor
wy_0362 8	-	385229 8	385362 9	-	1332	hypothetical protein
wy_0362 9	*mauA*	385362 6	385433 3	-	708	Methylamine dehydrogenase light chain precursor
wy_0363 0	*mauD*	385436 1	385497 5	-	615	Methylamine utilization protein MauD
wy_0363 1	*mauE*	385500 5	385558 9	-	585	Methylamine utilization protein MauE
wy_0363 2	-	385581 0	385734 5	+	1536	Amino acid permease
wy_0363 3	*kdgR_9*	385734 5	385810 3	+	759	Pectin degradation repressor protein KdgR
wy_0363 4	*osmF_2*	385810 5	385909 1	-	987	Putative osmoprotectant uptake system substrate-binding protein OsmF precursor
wy_0363 5	*opuBB_ 2*	385909 3	385985 4	-	762	Choline transport system permease protein OpuBB
wy_0363 6	*opuCA_ 2*	385985 1	386104 1	-	1191	Glycine betaine/carnitine/choline transport ATP-binding protein OpuCA
wy_0363 7	*opuCB_ 2*	386103 4	386169 0	-	657	Glycine betaine/carnitine/choline transport system permease protein OpuCB
wy_0363 8	-	386187 3	386214 8	-	276	RNHCP domain protein
wy_0363 9	-	386228 6	386262 1	-	336	RNHCP domain protein
wy_0364 0	*fdhF*	386290 4	386528 2	-	2379	Formate dehydrogenase H
wy_0364 1	-	386537 3	386596 6	-	594	Bacterial regulatory proteins, tetR family
wy_0364 2	*paaG_1*	386605 5	386685 8	+	804	1,2-epoxyphenylacetyl-CoA isomerase
wy_0364 3	*fabG_16*	386689 1	386766 1	+	771	3-oxoacyl-[acyl-carrier-protein] reductase FabG
wy_0364 4	*fabG3_1*	386778 2	386857 3	+	792	3-alpha-(or 20-beta)-hydroxysteroid dehydrogenase
wy_0364 5	*yhjX*	386861 5	386996 1	+	1347	putative MFS-type transporter YhjX
wy_0364 6	*hchA*	387000 4	387069 6	+	693	Molecular chaperone Hsp31 and glyoxalase 3
wy_0364 7	*lytR_2*	387076 1	387204 1	+	1281	Transcriptional regulator LytR
wy_0364 8	*qorA_3*	387205 8	387298 7	-	930	Quinone oxidoreductase 1
wy_0364 9	*kshA_7*	387305 0	387420 4	-	1155	3-ketosteroid-9-alpha-monooxygenase oxygenase subunit
wy_0365 0	-	387447 3	387531 5	+	843	Alpha/beta hydrolase family protein

wy_03651	*dacF*	3875324	3876223	-	900	D-alanyl-D-alanine carboxypeptidase DacF precursor
wy_03652	*afsQ1*	3876335	3877018	+	684	Transcriptional regulatory protein AfsQ1
wy_03653	*mtrB_2*	3877015	3878463	+	1449	Sensor histidine kinase MtrB
wy_03654	*tfdF*	3878747	3879916	+	1170	Maleylacetate reductase
wy_03655	*kstR2_13*	3880016	3880669	-	654	HTH-type transcriptional repressor KstR2
wy_03656	*lcfB_13*	3880656	3882266	-	1611	Long-chain-fatty-acid--CoA ligase
wy_03657	*hapE_6*	3882548	3884491	+	1944	4-hydroxyacetophenone monooxygenase
wy_03658	*fldA_3*	3884488	3885660	+	1173	E-cinnamoyl-CoA:R-phenyllactate CoA transferase
wy_03659	*lcfB_14*	3885657	3887243	+	1587	Long-chain-fatty-acid--CoA ligase
wy_03660	-	3887300	3887611	-	312	hypothetical protein
wy_03661	-	3887744	3888721	-	978	hypothetical protein
wy_03662	*csgD*	3888795	3891509	+	2715	CsgBAC operon transcriptional regulatory protein
wy_03663	*hsrA_3*	3891633	3893171	+	1539	putative transport protein HsrA
wy_03664	*ureH*	3893207	3894124	-	918	Urease accessory protein UreH
wy_03665	*ureG*	3894121	3894735	-	615	Urease accessory protein UreG
wy_03666	*ureF*	3894756	3895445	-	690	Urease accessory protein UreF
wy_03667	*ureC*	3895453	3897624	-	2172	Urease subunit alpha
wy_03668	*cbiX_2*	3897742	3898488	-	747	Sirohydrochlorin cobaltochelatase
wy_03669	*ureA*	3898493	3898798	-	306	Urease subunit gamma
wy_03670	*appA*	3899144	3900748	+	1605	Oligopeptide-binding protein AppA precursor
wy_03671	*gsiC_2*	3900733	3901719	+	987	Glutathione transport system permease protein GsiC
wy_03672	*oppC_1*	3901712	3902545	+	834	Oligopeptide transport system permease protein OppC
wy_03673	*ddpD*	3902542	3903468	+	927	putative D,D-dipeptide transport ATP-binding protein DdpD
wy_03674	*oppF_3*	3903465	3904133	+	669	Oligopeptide transport ATP-binding protein OppF
wy_03675	-	3904108	3904674	-	567	hypothetical protein
wy_03676	-	3904799	3905317	+	519	hypothetical protein

wy_03677	*cat1*	3905343	3906908	-	1566	Succinyl-CoA:coenzyme A transferase
wy_03678	-	3907072	3907398	-	327	Branched-chain amino acid transport protein (AzlD)
wy_03679	*ygaZ*	3907391	3908041	-	651	Inner membrane protein YgaZ
wy_03680	*lrp_2*	3908232	3908642	+	411	Leucine-responsive regulatory protein
wy_03681	*gntK*	3908632	3909162	-	531	Thermoresistant gluconokinase
wy_03682	*mepM_3*	3909708	3910571	+	864	Murein DD-endopeptidase MepM
wy_03683	*lcfB_15*	3910583	3912121	-	1539	Long-chain-fatty-acid--CoA ligase
wy_03684	*limA*	3912209	3912580	-	372	Limonene-1,2-epoxide hydrolase
wy_03685	-	3912577	3913473	-	897	DNA-binding transcriptional activator MhpR
wy_03686	*cypX*	3913561	3914772	+	1212	Pulcherriminic acid synthase
wy_03687	-	3914942	3915535	-	594	Bacterial regulatory proteins, tetR family
wy_03688	-	3915744	3916934	+	1191	Putative aminoglycoside phosphotransferase
wy_03689	-	3916934	3918094	+	1161	hypothetical protein
wy_03690	-	3918342	3918485	-	144	hypothetical protein
wy_03691	-	3918574	3918981	+	408	hypothetical protein
wy_03692	-	3919074	3919808	+	735	Undecaprenyl-phosphate mannosyltransferase
wy_03693	*eis*	3919882	3921087	+	1206	Enhanced intracellular survival protein
wy_03694	-	3921186	3921593	+	408	hypothetical protein
wy_03695	*benM_1*	3921600	3922457	-	858	HTH-type transcriptional regulator BenM
wy_03696	-	3922542	3923540	+	999	Major Facilitator Superfamily protein
wy_03697	-	3923654	3924292	-	639	Bacterial regulatory proteins, tetR family
wy_03698	*yvdD_2*	3924304	3924906	+	603	LOG family protein YvdD
wy_03699	-	3924957	3926579	-	1623	Long-chain-fatty-acid--CoA ligase
wy_03700	-	3926685	3927878	-	1194	Amidohydrolase
wy_03701	*glnA_3*	3927923	3929296	+	1374	Glutamine synthetase
wy_03702	-	3929331	3929888	-	558	hypothetical protein

wy_03703	*yabJ_2*	3929997	3930413	-	417	Enamine/imine deaminase
wy_03704	-	3930622	3932115	+	1494	Purine catabolism regulatory protein-like family protein
wy_03705	-	3932121	3932729	-	609	hypothetical protein
wy_03706	-	3932939	3933052	+	114	hypothetical protein
wy_03707	-	3933209	3934510	+	1302	hypothetical protein
wy_03708	-	3934528	3936516	-	1989	NADH oxidase
wy_03709	*ygcS*	3936858	3938231	+	1374	Inner membrane metabolite transport protein YgcS
wy_03710	*gabD_3*	3938273	3939733	+	1461	Succinate-semialdehyde dehydrogenase [NADP(+)] GabD
wy_03711	*citE_3*	3939730	3940563	+	834	Citrate lyase subunit beta
wy_03712	*pld*	3940568	3942232	-	1665	Phospholipase D precursor
wy_03713	-	3942400	3943167	-	768	hypothetical protein
wy_03714	*cueR*	3943164	3943616	-	453	HTH-type transcriptional regulator CueR
wy_03715	-	3943692	3943877	+	186	hypothetical protein
wy_03716	*yeaN*	3943892	3944911	+	1020	Inner membrane transport protein YeaN
wy_03717	-	3944927	3945313	-	387	hypothetical protein
wy_03718	-	3945473	3945880	+	408	hypothetical protein
wy_03719	-	3945959	3946090	-	132	hypothetical protein
wy_03720	*pepN_2*	3946256	3948754	-	2499	Aminopeptidase N
wy_03721	*echA8_6*	3948882	3949661	+	780	putative enoyl-CoA hydratase echA8
wy_03722	-	3949770	3950084	+	315	hypothetical protein
wy_03723	*eutC*	3950115	3950861	-	747	Ethanolamine ammonia-lyase light chain
wy_03724	*eutB*	3950858	3952270	-	1413	Ethanolamine ammonia-lyase heavy chain
wy_03725	-	3952368	3953033	-	666	hypothetical protein
wy_03726	-	3953048	3953929	-	882	Aminoglycoside/hydroxyurea antibiotic resistance kinase
wy_03727	-	3953953	3954300	-	348	Zn-finger in ubiquitin-hydrolases and other protein
wy_03728	*ethA*	3954452	3955972	+	1521	FAD-containing monooxygenase EthA

wy_03729	*silP*	3956042	3958390	+	2349	Silver exporting P-type ATPase
wy_03730	*sadH_5*	3958961	3959782	+	822	Putative oxidoreductase SadH
wy_03731	-	3959851	3960885	+	1035	hypothetical protein
wy_03732	-	3960919	3962658	-	1740	Cutinase
wy_03733	*sigI_1*	3962786	3964087	-	1302	putative ECF RNA polymerase sigma factor SigI
wy_03734	-	3964092	3964514	-	423	YCII-related domain protein
wy_03735	-	3964686	3965369	-	684	chaperone protein HchA
wy_03736	-	3965491	3966171	+	681	DNA alkylation repair enzyme
wy_03737	*benM_2*	3966173	3967174	-	1002	HTH-type transcriptional regulator BenM
wy_03738	-	3967297	3968397	+	1101	Major Facilitator Superfamily protein
wy_03739	-	3968531	3969010	-	480	hypothetical protein
wy_03740	-	3969116	3969445	-	330	Zn-finger in ubiquitin-hydrolases and other protein
wy_03741	*nhaK_2*	3969511	3971109	+	1599	Sodium, potassium, lithium and rubidium/H(+) antiporter
wy_03742	-	3971012	3972142	-	1131	NYN domain protein
wy_03743	*gap*	3972343	3973806	-	1464	Glyceraldehyde-3-phosphate dehydrogenase
wy_03744	-	3974105	3974647	-	543	hypothetical protein
wy_03745	*dnaB*	3974728	3976245	-	1518	Replicative DNA helicase
wy_03746	*rplI*	3976628	3977083	-	456	50S ribosomal protein L9
wy_03747	*rpsR*	3977101	3977343	-	243	30S ribosomal protein S18
wy_03748	*ssb_2*	3977383	3977916	-	534	Single-stranded DNA-binding protein
wy_03749	*rpsF*	3978009	3978296	-	288	30S ribosomal protein S6
wy_03750	-	3978481	3979845	+	1365	hypothetical protein
wy_03751	-	3979947	3980321	+	375	hypothetical protein
wy_03752	-	3980341	3981897	-	1557	hypothetical protein
wy_03753	*ponA1_1*	3981972	3984506	-	2535	Penicillin-binding protein 1A
wy_03754	-	3984582	3985001	-	420	hypothetical protein

wy_03755	-	3985047	3986033	+	987	hypothetical protein
wy_03756	-	3986036	3986992	+	957	hypothetical protein
wy_03757	-	3986932	3987579	+	648	hypothetical protein
wy_03758	-	3987582	3988388	+	807	hypothetical protein
wy_03759	-	3988495	3989061	+	567	Transcriptional regulator PadR-like family protein
wy_03760	*ino1*	3989054	3990148	+	1095	Inositol-3-phosphate synthase
wy_03761	*yheS_2*	3990429	3992039	+	1611	putative ABC transporter ATP-binding protein YheS
wy_03762	-	3992054	3992812	-	759	putative oxidoreductase
wy_03763	-	3992809	3993888	-	1080	Putative aminoglycoside phosphotransferase
wy_03764	*acrC_3*	3993885	3995117	-	1233	Acryloyl-CoA reductase (NADH)
wy_03765	*luxR*	3995209	3995814	+	606	HTH-type transcriptional regulator LuxR
wy_03766	-	3995811	3997466	-	1656	hypothetical protein
wy_03767	*ponA1_2*	3997536	3999680	-	2145	Penicillin-binding protein 1A
wy_03768	*ytrA_3*	3999878	4000273	+	396	HTH-type transcriptional repressor YtrA
wy_03769	*ytrB*	4000270	4001130	+	861	ABC transporter ATP-binding protein YtrB
wy_03770	-	4001127	4002179	+	1053	ABC-2 family transporter protein
wy_03771	*emrY*	4002548	4004092	+	1545	putative multidrug resistance protein EmrY
wy_03772	*ponA1_3*	4004125	4006338	-	2214	Penicillin-binding protein 1A
wy_03773	-	4006694	4006891	-	198	hypothetical protein
wy_03774	*sugB*	4006893	4007849	-	957	Trehalose transport system permease protein SugB
wy_03775	*sugA*	4007849	4008895	-	1047	Trehalose transport system permease protein SugA
wy_03776	*lpqY*	4008974	4010257	-	1284	Trehalose-binding lipoprotein LpqY precursor
wy_03777	*sugC*	4010404	4011486	-	1083	Trehalose import ATP-binding protein SugC
wy_03778	-	4011588	4012070	-	483	hypothetical protein
wy_03779	-	4012149	4012388	+	240	hypothetical protein
wy_03780	-	4012566	4013360	+	795	Phthiodiolone/phenolphthiodiolone dimycocerosates ketoreductase

wy_03781	*artQ_2*	4013373	4015169	+	1797	Arginine transport system permease protein ArtQ
wy_03782	*artM*	4015173	4015904	+	732	Arginine transport ATP-binding protein ArtM
wy_03783	-	4016045	4017118	+	1074	Sugar-binding cellulase-like protein
wy_03784	-	4017194	4018690	-	1497	Beta-monoglucosyldiacylglycerol synthase
wy_03785	-	4018687	4020255	-	1569	hypothetical protein
wy_03786	-	4020636	4022597	+	1962	hypothetical protein
wy_03787	-	4022594	4024336	+	1743	hypothetical protein
wy_03788	*ycdF_3*	4024340	4025128	-	789	Glucose 1-dehydrogenase 2
wy_03789	*acm*	4025141	4025827	-	687	Lysozyme M1 precursor
wy_03790	-	4025972	4027309	-	1338	alpha-glucosidase
wy_03791	*polX*	4027326	4028351	-	1026	DNA polymerase/3'-5' exonuclease PolX
wy_03792	*ohrB*	4028389	4028814	-	426	Organic hydroperoxide resistance protein OhrB
wy_03793	*ohrR_2*	4028811	4029317	-	507	Organic hydroperoxide resistance transcriptional regulator
wy_03794	-	4029351	4029755	+	405	hypothetical protein
wy_03795	-	4029769	4030122	+	354	hypothetical protein
wy_03796	-	4030144	4030827	+	684	putative oxidoreductase
wy_03797	-	4030837	4031367	-	531	HutD
wy_03798	*ksi_1*	4031491	4031862	-	372	Steroid Delta-isomerase
wy_03799	*yclQ*	4032120	4033112	+	993	putative ABC transporter solute-binding protein YclQ precursor
wy_03800	*feuB*	4033191	4034171	+	981	Iron-uptake system permease protein FeuB
wy_03801	*feuC*	4034164	4035219	+	1056	Iron-uptake system permease protein FeuC
wy_03802	*fhuC_1*	4035216	4035971	+	756	Iron(3+)-hydroxamate import ATP-binding protein FhuC
wy_03803	-	4036060	4037568	+	1509	putative inactive lipase/MT1628
wy_03804	*leuS*	4037638	4040490	-	2853	Leucine--tRNA ligase
wy_03805	-	4040492	4041010	+	519	hypothetical protein
wy_0380	-	404102	404200	+	981	hypothetical protein

6		8	8			
wy_03807	*gltR_2*	4042022	4042864	-	843	HTH-type transcriptional regulator GltR
wy_03808	-	4042949	4043935	+	987	Sodium Bile acid symporter family protein
wy_03809	*gadB*	4044032	4045435	+	1404	Glutamate decarboxylase beta
wy_03810	*nlhH_7*	4045472	4046440	+	969	Carboxylesterase NlhH
wy_03811	-	4046449	4047054	-	606	hypothetical protein
wy_03812	-	4047109	4047369	-	261	hypothetical protein
wy_03813	*ygaP*	4047399	4047749	-	351	Inner membrane protein YgaP
wy_03814	-	4047854	4048546	-	693	putative HTH-type transcriptional regulator/MT1864
wy_03815	-	4048687	4049634	+	948	3 beta-hydroxysteroid dehydrogenase/Delta 5-->4-isomerase
wy_03816	-	4049644	4050945	+	1302	2-acyl-glycerophospho-ethanolamine acyltransferase
wy_03817	-	4050942	4051451	-	510	hypothetical protein
wy_03818	-	4051568	4053076	+	1509	hypothetical protein
wy_03819	*fbpC_4*	4053049	4054143	-	1095	Fe(3+) ions import ATP-binding protein FbpC
wy_03820	*modB*	4054140	4054946	-	807	Molybdenum transport system permease protein ModB
wy_03821	*modA*	4054943	4055719	-	777	Molybdate-binding periplasmic protein precursor
wy_03822	-	4055716	4056123	-	408	TOBE domain protein
wy_03823	*cca*	4056186	4057640	-	1455	CCA-adding enzyme
wy_03824	*mutT4_2*	4057766	4058329	+	564	Putative mutator protein MutT4
wy_03825	-	4058326	4060803	+	2478	hypothetical protein
wy_03826	*mviN*	4061067	4064882	+	3816	putative peptidoglycan biosynthesis protein MviN
wy_03827	*sigM*	4064943	4065524	+	582	ECF RNA polymerase sigma factor SigM
wy_03828	*rsmA_2*	4065589	4066356	+	768	Anti-sigma-M factor RsmA
wy_03829	-	4066363	4066983	-	621	Bacterial regulatory proteins, tetR family
wy_03830	-	4067209	4068336	+	1128	FtsX-like permease family protein
wy_03831	*lolD*	4068333	4069058	+	726	Lipoprotein-releasing system ATP-binding protein LolD
wy_0383	*fadR_3*	406908	406968	+	594	Fatty acid metabolism regulator protein

2		9	2			
wy_03833	*trxB_1*	4069889	4070878	+	990	Thioredoxin reductase
wy_03834	*trxA_2*	4070931	4071254	+	324	Thioredoxin
wy_03835	*lytC*	4071397	4072599	+	1203	N-acetylmuramoyl-L-alanine amidase LytC precursor
wy_03836	-	4072649	4073338	-	690	hypothetical protein
wy_03837	*parB*	4073573	4074724	-	1152	putative chromosome-partitioning protein ParB
wy_03838	*soj_3*	4074726	4075733	-	1008	Sporulation initiation inhibitor protein Soj
wy_03839	*rsmG_1*	4075804	4076475	-	672	Ribosomal RNA small subunit methyltransferase G
wy_03840	*estB_7*	4077733	4078695	+	963	Extracellular esterase EstB precursor
wy_03841	*rsmG_2*	4078751	4079428	-	678	Ribosomal RNA small subunit methyltransferase G
wy_03842	-	4079531	4080082	-	552	R3H domain protein
wy_03843	*misCA*	4080103	4081194	-	1092	Membrane protein insertase MisCA precursor
wy_03844	*yidD*	4081270	4081608	-	339	Putative membrane protein insertion efficiency factor
wy_03845	*rnpA*	4081605	4081982	-	378	Ribonuclease P protein component
wy_03846	*dnaA*	4082801	4084375	+	1575	Chromosomal replication initiator protein DnaA
wy_03847	*dnaN*	4084910	4086079	+	1170	DNA polymerase III subunit beta
wy_03848	*gntZ*	4086154	4087077	+	924	6-phosphogluconate dehydrogenase, NAD(+)-dependent, decarboxylating
wy_03849	*recF*	4087077	4088303	+	1227	DNA replication and repair protein RecF
wy_03850	-	4088300	4088851	+	552	hypothetical protein
wy_03851	-	4088967	4089875	+	909	putative metal-dependent hydrolase
wy_03852	*ophA1*	4089872	4090993	+	1122	Phthalate dioxygenase reductase
wy_03853	*dhaA_4*	4091019	4091939	+	921	Haloalkane dehalogenase 3
wy_03854	*gyrB*	4092170	4094206	+	2037	DNA gyrase subunit B
wy_03855	*gyrA*	4094305	4096824	+	2520	DNA gyrase subunit A
wy_03856	-	4096825	4097889	+	1065	hypothetical protein
wy_03858	-	4098068	4098178	+	111	hypothetical protein
wy_0386	*ksi_2*	409844	409881	-	375	Steroid Delta-isomerase

0		2	6			
wy_0386 1	*kstR2_1 4*	409942 5	410069 0	+	1266	HTH-type transcriptional repressor KstR2
wy_0386 2	-	410072 5	410198 4	+	1260	putative inactive lipase/MT1628
wy_0386 3	-	410198 8	410211 0	-	123	hypothetical protein
wy_0386 4	*ppiA*	410216 6	410274 7	+	582	Peptidyl-prolyl cis-trans isomerase A
wy_0386 5	*gluP*	410275 8	410366 0	+	903	Rhomboid protease GluP
wy_0386 6	-	410367 7	410414 7	-	471	hypothetical protein
wy_0386 7	*crgA*	410425 5	410451 8	-	264	Cell division protein CrgA
wy_0386 8	-	410467 5	410543 9	+	765	hypothetical protein
wy_0386 9	*trpG*	410547 1	410613 6	+	666	Anthranilate synthase component 2
wy_0387 0	*pknB_1*	410616 0	410809 1	-	1932	Serine/threonine-protein kinase PknB
wy_0387 1	*pknA*	410808 8	410945 5	-	1368	Serine/threonine-protein kinase PknA
wy_0387 2	*pbpA_3*	410945 9	411094 6	-	1488	Penicillin-binding protein A
wy_0387 3	*ftsW_2*	411094 3	411238 5	-	1443	Lipid II flippase FtsW
wy_0387 4	*pstP*	411238 5	411381 8	-	1434	PP2C-family Ser/Thr phosphatase
wy_0387 5	*fhaB*	411381 5	411428 2	-	468	FHA domain-containing protein FhaB
wy_0387 6	*fhaA*	411444 6	411580 1	-	1356	FHA domain-containing protein FhaA
wy_0387 8	-	411626 2	411722 1	-	960	Alpha/beta hydrolase family protein
wy_0387 9	*ttgW_4*	411733 9	411792 0	+	582	putative HTH-type transcriptional regulator TtgW
wy_0388 0	-	411800 2	411850 2	-	501	hypothetical protein
wy_0388 1	*yjgH_1*	411852 2	411893 2	-	411	RutC family protein YjgH
wy_0388 2	*yybR*	411901 6	411939 9	+	384	putative HTH-type transcriptional regulator YybR
wy_0388 3	*gylR*	411940 4	411987 4	-	471	Glycerol operon regulatory protein
wy_0388 4	*plaP*	412014 8	412168 0	-	1533	Low-affinity putrescine importer PlaP
wy_0388 5	*rebO*	412187 4	412345 1	+	1578	Flavin-dependent L-tryptophan oxidase RebO precursor
wy_0388 6	-	412348 1	412415 5	+	675	Endoribonuclease L-PSP
wy_0388	*tatAy*	412415	412435	+	201	Sec-independent protein translocase protein

7		5	5			TatAy
wy_03888	-	4124418	4124873	-	456	MarR family protein
wy_03889	*bmr3_7*	4125057	4126496	+	1440	Multidrug resistance protein 3
wy_03890	*hpxO*	4126671	4127690	-	1020	FAD-dependent urate hydroxylase
wy_03891	*vgb*	4127874	4128839	+	966	Virginiamycin B lyase
wy_03892	*gltB_4*	4129226	4133812	+	4587	Glutamate synthase [NADPH] large chain
wy_03893	*gltB_5*	4133805	4135256	+	1452	Glutamate synthase [NADPH] small chain
wy_03894	-	4135515	4137347	+	1833	hypothetical protein
wy_03895	-	4137352	4139115	+	1764	hypothetical protein
wy_03896	-	4139330	4140289	+	960	Cutinase
wy_03897	-	4140296	4140631	-	336	hypothetical protein
wy_03898	-	4141536	4142117	+	582	hypothetical protein
wy_03899	*ydfG*	4142178	4142978	-	801	NADP-dependent 3-hydroxy acid dehydrogenase YdfG
wy_03900	-	4142975	4144636	-	1662	KsdD-like steroid dehydrogenase
wy_03901	*acrR_2*	4144654	4145277	-	624	HTH-type transcriptional regulator AcrR
wy_03902	-	4145357	4145851	+	495	hypothetical protein
wy_03903	-	4145908	4146201	+	294	Putative monooxygenase
wy_03904	*cocE*	4146351	4148372	+	2022	Cocaine esterase
wy_03905	-	4148458	4149150	-	693	hypothetical protein
wy_03906	*lysX*	4149158	4152523	-	3366	Lysylphosphatidylglycerol biosynthesis bifunctional protein LysX
wy_03907	-	4152609	4152839	+	231	ribosome-associated protein
wy_03908	-	4152926	4154446	+	1521	TMAO/DMSO reductase
wy_03909	-	4154463	4155143	-	681	transcriptional regulator NanR
wy_03910	-	4155281	4156654	+	1374	putative FAD-linked oxidoreductase
wy_03911	-	4156686	4156808	+	123	hypothetical protein
wy_03912	*echA8_7*	4156830	4157612	-	783	putative enoyl-CoA hydratase echA8
wy_0391	*lcfB_16*	415760	415904	-	1434	Long-chain-fatty-acid--CoA ligase

3		9	2			
wy_03914	*iclR_2*	4159177	4159914	+	738	Acetate operon repressor
wy_03915	-	4159924	4160571	-	648	hypothetical protein
wy_03916	*carAa_2*	4160858	4162051	+	1194	Carbazole 1,9a-dioxygenase, terminal oxygenase component CarAa
wy_03917	*mmoC*	4162093	4163106	+	1014	Methane monooxygenase component C
wy_03918	*dbfB*	4163159	4164037	+	879	2,2',3-trihydroxybiphenyl dioxygenase
wy_03919	*gatA_4*	4164034	4165365	+	1332	Glutamyl-tRNA(Gln) amidotransferase subunit A
wy_03920	*naaA_2*	4165358	4166692	+	1335	5-nitroanthranilic acid aminohydrolase
wy_03921	-	4166689	4167444	+	756	Asp/Glu/Hydantoin racemase
wy_03922	*xylF_3*	4167453	4168313	+	861	2-hydroxymuconate semialdehyde hydrolase
wy_03923	*hapE_7*	4168310	4169845	+	1536	4-hydroxyacetophenone monooxygenase
wy_03924	-	4170087	4171388	+	1302	Alpha/beta hydrolase family protein
wy_03925	*rraA*	4171451	4171921	-	471	Putative regulator of ribonuclease activity
wy_03926	-	4171984	4172364	-	381	hypothetical protein
wy_03927	*kstR2_15*	4172408	4172992	-	585	HTH-type transcriptional repressor KstR2
wy_03928	-	4173168	4174205	-	1038	Putative aminoglycoside phosphotransferase
wy_03929	-	4174355	4175179	+	825	fructoselysine 3-epimerase
wy_03930	*gctA_1*	4175201	4176133	+	933	Glutaconate CoA-transferase subunit A
wy_03931	*catJ_1*	4176130	4176888	+	759	3-oxoadipate CoA-transferase subunit B
wy_03932	-	4176885	4178012	+	1128	Nitronate monooxygenase
wy_03933	-	4178012	4178887	+	876	Putative short-chain type dehydrogenase/reductase
wy_03934	-	4178925	4179371	+	447	hypothetical protein
wy_03935	-	4179371	4179778	+	408	bifunctional enoyl-CoA hydratase/phosphate acetyltransferase
wy_03936	*qorA_4*	4179775	4180755	+	981	Quinone oxidoreductase 1
wy_03937	*acrC_4*	4180759	4181958	+	1200	Acryloyl-CoA reductase (NADH)
wy_03939	-	4183688	4185535	-	1848	Long-chain-fatty-acid--CoA ligase FadD15

wy_03940	-	4185648	4186640	+	993	hypothetical protein
wy_03941	-	4186643	4188274	-	1632	ABC-2 family transporter protein
wy_03942	*drrA_9*	4188271	4189170	-	900	Daunorubicin/doxorubicin resistance ATP-binding protein DrrA
wy_03943	-	4189167	4189829	-	663	transcriptional regulator BetI
wy_03944	-	4190234	4190758	+	525	hypothetical protein
wy_03945	-	4190800	4191816	+	1017	hypothetical protein
wy_03946	*gabD2_2*	4191938	4193491	+	1554	Putative succinate-semialdehyde dehydrogenase [NADP(+)] 2
wy_03947	-	4193652	4194254	+	603	Response regulator receiver domain protein
wy_03948	-	4194251	4195993	+	1743	hypothetical protein
wy_03949	-	4196165	4196842	+	678	hypothetical protein
wy_03950	*pnbA_4*	4196869	4198425	+	1557	Para-nitrobenzyl esterase
wy_03951	*qacA_5*	4198574	4200229	+	1656	Antiseptic resistance protein
wy_03952	-	4200288	4200485	-	198	hypothetical protein
wy_03953	-	4200485	4200922	-	438	SPW repeat protein
wy_03954	*mmsA_2*	4201077	4202555	-	1479	Methylmalonate-semialdehyde dehydrogenase [acylating]
wy_03955	*proP_3*	4202588	4203958	-	1371	Proline/betaine transporter
wy_03956	*mmsB_2*	4203955	4204869	-	915	3-hydroxyisobutyrate dehydrogenase
wy_03957	*dmlR*	4205010	4205906	+	897	HTH-type transcriptional regulator DmlR
wy_03958	*acr1_7*	4205916	4206776	-	861	Fatty acyl-CoA reductase
wy_03959	-	4207061	4207591	+	531	hypothetical protein
wy_03960	-	4207656	4208651	+	996	hypothetical protein
wy_03961	-	4208654	4209388	+	735	hypothetical protein
wy_03962	-	4209398	4210342	-	945	Copper resistance protein D
wy_03963	*ycnJ*	4210359	4210907	-	549	Copper transport protein YcnJ precursor
wy_03964	-	4210904	4211557	-	654	hypothetical protein
wy_0396	-	421160	421208	-	480	hypothetical protein

5		1	0			
wy_0396 6	-	421214 8	421277 7	-	630	hypothetical protein
wy_0396 7	-	421293 5	421449 4	+	1560	hypothetical protein
wy_0396 8	-	421463 2	421514 1	+	510	Pyridoxamine 5'-phosphate oxidase
wy_0396 9	*fabG_17*	421518 8	421595 5	-	768	3-oxoacyl-[acyl-carrier-protein] reductase FabG
wy_0397 0	*yabJ_3*	421612 6	421649 4	+	369	Enamine/imine deaminase
wy_0397 1	*otemo*	421655 8	421810 2	+	1545	2-oxo-Delta(3)-4,5, 5-trimethylcyclopentenylacetyl-CoA monooxygenase
wy_0397 2	-	421818 7	421877 1	+	585	hypothetical protein
wy_0397 3	-	421877 3	421940 8	+	636	hypothetical protein
wy_0397 4	-	421948 2	422008 4	+	603	hypothetical protein
wy_0397 5	-	422014 8	422095 4	+	807	hypothetical protein
wy_0397 6	-	422097 9	422134 4	-	366	hypothetical protein
wy_0397 7	-	422136 0	422189 9	-	540	hypothetical protein
wy_0397 8	*yqjI*	422207 0	422271 7	+	648	Transcriptional regulator YqjI
wy_0397 9	-	422274 2	422328 7	-	546	hypothetical protein
wy_0398 0	*liaR_6*	422329 1	422391 1	-	621	Transcriptional regulatory protein LiaR
wy_0398 1	*degS_2*	422390 8	422506 5	-	1158	Signal transduction histidine-protein kinase/phosphatase DegS
wy_0398 2	-	422520 7	422579 7	+	591	hypothetical protein
wy_0398 3	-	422579 4	422643 5	+	642	Ankyrin repeats (3 copies)
wy_0398 4	-	422644 3	422726 4	-	822	hypothetical protein
wy_0398 5	*nylA_2*	422729 9	422873 8	-	1440	6-aminohexanoate-cyclic-dimer hydrolase
wy_0398 6	*kstR2_1 6*	422877 3	422954 3	-	771	HTH-type transcriptional repressor KstR2
wy_0398 7	*hsaC_3*	422964 8	423052 9	-	882	Iron-dependent extradiol dioxygenase
wy_0398 8	*hsaA_5*	423054 5	423170 8	-	1164	Flavin-dependent monooxygenase, oxygenase subunit HsaA
wy_0398 9	*hsaD_6*	423174 4	423258 0	-	837	4,5:9,10-diseco-3-hydroxy-5,9, 17-trioxoandrosta-1(10),2-diene-4-oate hydrolase
wy_0399	*sodB*	423283	423347	+	639	Superoxide dismutase [Fe]

0		4	2			
wy_03991	*nrdF1*	4233465	4234433	+	969	Ribonucleoside-diphosphate reductase subunit beta nrdF1
wy_03992	-	4234438	4234923	-	486	hypothetical protein
wy_03993	-	4234933	4235640	-	708	hypothetical protein
wy_03994	-	4235911	4236438	-	528	hypothetical protein
wy_03995	*araB*	4236601	4237461	-	861	L-arabinolactonase
wy_03996	-	4237587	4238447	+	861	VTC domain protein
wy_03997	-	4238447	4239124	+	678	hypothetical protein
wy_03998	-	4239124	4240782	+	1659	CotH protein
wy_03999	*sodA_1*	4240972	4241601	-	630	Superoxide dismutase [Mn]
wy_04000	-	4241838	4243028	+	1191	Acyltransferase family protein
wy_04001	*msrA*	4243038	4243706	+	669	Peptide methionine sulfoxide reductase MsrA
wy_04002	*stp_4*	4243773	4245278	-	1506	Multidrug resistance protein stp
wy_04003	-	4245524	4246453	-	930	Phthiodiolone/phenolphthiodiolone dimycocerosates ketoreductase
wy_04004	*glpE*	4246973	4247296	+	324	Thiosulfate sulfurtransferase GlpE
wy_04005	-	4247297	4248226	+	930	hypothetical protein
wy_04006	*glpQ1_1*	4248223	4248987	+	765	putative glycerophosphoryl diester phosphodiesterase 1
wy_04007	-	4249569	4249937	+	369	hypothetical protein
wy_04008	*bfrB_1*	4250012	4250560	-	549	Ferritin BfrB
wy_04009	*bfrB_2*	4250719	4251264	-	546	Ferritin BfrB
wy_04010	*yvhJ*	4251328	4252242	-	915	Putative transcriptional regulator YvhJ
wy_04011	-	4252719	4253537	-	819	CAAX amino terminal protease self- immunity
wy_04012	*pbpX_2*	4253574	4254809	-	1236	Putative penicillin-binding protein PbpX
wy_04013	-	4254806	4256191	-	1386	Putative diacylglycerol O-acyltransferase/MT3848
wy_04014	-	4256284	4257045	-	762	hypothetical protein
wy_04015	-	4257202	4257891	+	690	hypothetical protein

wy_04016	*pheA*	4257915	4258862	+	948	Prephenate dehydratase
wy_04017	*gpgP_3*	4258859	4259548	+	690	Glucosyl-3-phosphoglycerate phosphatase
wy_04018	*espG1*	4259553	4260347	-	795	ESX-1 secretion-associated protein EspG1
wy_04019	-	4260344	4261576	-	1233	putative PPE family protein PPE47/PPE48
wy_04020	-	4261620	4261928	-	309	PE family protein
wy_04021	-	4262059	4262409	-	351	Possibl zinc metallo-peptidase
wy_04022	-	4262411	4263484	-	1074	putative membrane protein/MT3943
wy_04023	-	4263545	4263967	+	423	Ankyrin repeats (3 copies)
wy_04024	*serS*	4263992	4265257	+	1266	Serine--tRNA ligase
wy_04025	-	4265337	4265825	-	489	hypothetical protein
wy_04026	-	4266339	4269566	+	3228	hypothetical protein
wy_04027	-	4270016	4273162	+	3147	hypothetical protein
wy_04028	-	4273443	4274690	+	1248	hypothetical protein
wy_04029	-	4274848	4276935	+	2088	hypothetical protein
wy_04030	-	4277209	4278744	+	1536	hypothetical protein
wy_04031	*plsC_3*	4278798	4279622	+	825	1-acyl-sn-glycerol-3-phosphate acyltransferase
wy_04032	*ywpJ*	4279704	4280528	+	825	Putative phosphatase YwpJ
wy_04033	-	4280605	4282656	-	2052	N-acetylmuramoyl-L-alanine amidase
wy_04034	*lytB*	4283030	4284748	+	1719	Amidase enhancer precursor
wy_04035	*tipA*	4284892	4285650	+	759	HTH-type transcriptional activator TipA
wy_04036	*glf*	4285664	4286950	+	1287	UDP-galactopyranose mutase
wy_04037	*glfT2_1*	4286947	4288797	+	1851	Galactofuranosyl transferase GlfT2
wy_04038	-	4288781	4289296	+	516	PAP2 superfamily protein
wy_04039	-	4289293	4290198	+	906	Decaprenyl-phosphate phosphoribosyltransferase
wy_04040	-	4290176	4292092	+	1917	hypothetical protein
wy_04041	*fbpC_5*	4292384	4293403	+	1020	Diacylglycerol acyltransferase/mycolyltransferase Ag85C

						precursor
wy_04042	*fbpB_2*	4293600	4295489	+	1890	Diacylglycerol acyltransferase/mycolyltransferase Ag85B precursor
wy_04043	-	4295605	4296162	+	558	hypothetical protein
wy_04044	-	4296241	4297155	+	915	Cutinase
wy_04045	*fgd_3*	4297318	4298319	+	1002	F420-dependent glucose-6-phosphate dehydrogenase
wy_04046	-	4298557	4300452	+	1896	Long-chain-fatty-acid--AMP ligase FadD32
wy_04047	*ppsA_2*	4300484	4305409	+	4926	Phthiocerol/phenolphthiocerol synthesis polyketide synthase type I PpsA
wy_04048	*accD5_3*	4305410	4306954	+	1545	putative propionyl-CoA carboxylase beta chain 5
wy_04049	-	4307022	4308113	-	1092	hypothetical protein
wy_04050	*embB*	4308145	4311465	-	3321	putative arabinosyltransferase B
wy_04051	*embC_2*	4311539	4314862	-	3324	putative arabinosyltransferase C
wy_04052	-	4314891	4316696	-	1806	hypothetical protein
wy_04053	-	4316951	4317400	+	450	hypothetical protein
wy_04054	*cmtR*	4317387	4317722	+	336	HTH-type transcriptional regulator CmtR
wy_04055	-	4317688	4317882	-	195	helix-turn-helix protein
wy_04056	*map_2*	4318017	4318787	+	771	Methionine aminopeptidase 1
wy_04057	-	4318797	4321604	+	2808	putative FAD-linked oxidoreductase
wy_04058	-	4321619	4321801	+	183	hypothetical protein
wy_04059	-	4321807	4322295	+	489	hypothetical protein
wy_04063	-	4328226	4329158	+	933	hypothetical protein
wy_04064	-	4329299	4330102	+	804	hypothetical protein
wy_04068	-	4336433	4336591	-	159	hypothetical protein
wy_04069	-	4336588	4336953	-	366	MarR family protein
wy_04070	-	4337030	4338448	+	1419	hypothetical protein
wy_04071	*aftA*	4338450	4340297	-	1848	Arabinofuranosyltransferase AftA

wy_04072	-	4340430	4341308	+	879	Tropinesterase
wy_04073	*kstR_6*	4341378	4342037	-	660	HTH-type transcriptional repressor KstR
wy_04074	*cysQ_2*	4342071	4342892	+	822	3'-phosphoadenosine 5'-phosphate phosphatase
wy_04075	*cysD_3*	4342889	4343794	+	906	Sulfate adenylyltransferase subunit 2
wy_04076	*cysNC_3*	4343794	4345695	+	1902	Bifunctional enzyme CysN/CysC
wy_04077	*ksi_3*	4345737	4346096	+	360	Steroid Delta-isomerase
wy_04078	*hdhA_4*	4346093	4346905	+	813	7-alpha-hydroxysteroid dehydrogenase
wy_04079	-	4346929	4348071	+	1143	hypothetical protein
wy_04080	-	4348097	4349158	+	1062	hypothetical protein
wy_04081	*dppA_2*	4349248	4350858	+	1611	Periplasmic dipeptide transport protein precursor
wy_04082	-	4351040	4351324	+	285	hypothetical protein
wy_04083	*phbB*	4351389	4352150	-	762	Acetoacetyl-CoA reductase
wy_04084	*dprE1_1*	4352147	4353631	-	1485	putative decaprenylphosphoryl-beta-D-ribose oxidase
wy_04085	*sodA_2*	4353722	4354351	-	630	Superoxide dismutase [Mn]
wy_04086	-	4354448	4354915	+	468	GtrA-like protein
wy_04087	*ssuD_2*	4355004	4355855	+	852	Alkanesulfonate monooxygenase
wy_04088	*htrA*	4355856	4356974	-	1119	Putative serine protease HtrA
wy_04089	*rmlC*	4356979	4357575	-	597	dTDP-4-dehydrorhamnose 3,5-epimerase
wy_04090	*rmlA_2*	4357578	4358453	-	876	Glucose-1-phosphate thymidylyltransferase
wy_04091	*proP_4*	4358665	4360047	+	1383	Proline/betaine transporter
wy_04092	*rmlB_2*	4360079	4361074	+	996	dTDP-glucose 4,6-dehydratase
wy_04093	-	4361190	4361816	+	627	putative PPE family protein PPE42
wy_04094	-	4361832	4362839	-	1008	hypothetical protein
wy_04095	*lpxA*	4362892	4363617	-	726	Acyl-[acyl-carrier-protein]--UDP-N-acetylglucosamine O-acyltransferase
wy_04096	*fdtB*	4363728	4364828	+	1101	dTDP-3-amino-3,6-dideoxy-alpha-D-galactopyranose transaminase

wy_04097	*ligC*	4364831	4365790	+	960	4-carboxy-2-hydroxymuconate-6-semialdehyde dehydrogenase
wy_04098	-	4365783	4368005	-	2223	hypothetical protein
wy_04099	-	4368114	4368887	+	774	N-glycosyltransferase
wy_04100	*glfT2_2*	4368953	4370803	+	1851	Galactofuranosyl transferase GlfT2
wy_04101	-	4370816	4371676	-	861	Macrocin-O-methyltransferase (TylF)
wy_04102	*glfT1*	4371783	4372694	-	912	Galactofuranosyl transferase GlfT1
wy_04103	*tagH*	4372691	4373509	-	819	Teichoic acids export ATP-binding protein TagH
wy_04104	*tagG*	4373530	4374411	-	882	Teichoic acid translocation permease protein TagG
wy_04105	-	4374466	4375014	-	549	hypothetical protein
wy_04106	*csdA*	4375085	4376377	+	1293	Cysteine desulfurase CsdA
wy_04107	*qorA_5*	4376387	4377454	-	1068	Quinone oxidoreductase 1
wy_04108	-	4377748	4378206	+	459	transcriptional repressor MprA
wy_04110	*yocK*	4378393	4378734	-	342	General stress protein 16O
wy_04111	*fbpA_7*	4379030	4380031	+	1002	Diacylglycerol acyltransferase/mycolyltransferase Ag85A precursor
wy_04112	*embR*	4380038	4381996	-	1959	Transcriptional regulatory protein EmbR
wy_04113	*dehH1_3*	4382090	4382983	+	894	Haloacetate dehalogenase H-1
wy_04114	-	4383312	4383401	-	90	hypothetical protein
wy_04115	*yhcR*	4383528	4385231	-	1704	Endonuclease YhcR precursor
wy_04116	*pat_2*	4385311	4385778	-	468	Putative phenylalanine aminotransferase
wy_04117	*pat_3*	4385856	4386398	-	543	Putative phenylalanine aminotransferase
wy_04118	-	4386460	4387188	+	729	Dienelactone hydrolase family protein
wy_04121	-	4387578	4387781	-	204	hypothetical protein
wy_04122	-	4387789	4388241	-	453	hypothetical protein
wy_04123	-	4388539	4388991	+	453	hypothetical protein
wy_04124	-	4389057	4389392	-	336	hypothetical protein
wy_0412	-	438968	439056	+	882	Phosphatidylinositol mannoside acyltransferase

5		2	3			
wy_04126	-	4390604	4390777	-	174	hypothetical protein
wy_04127	-	4391013	4392482	-	1470	hypothetical protein
wy_04128	-	4392720	4392845	-	126	hypothetical protein
wy_04129	-	4392974	4393195	-	222	hypothetical protein
wy_04130	*xdhA*	4393269	4395371	-	2103	Xanthine dehydrogenase molybdenum-binding subunit
wy_04131	*hcrB*	4395368	4396360	-	993	4-hydroxybenzoyl-CoA reductase subunit beta
wy_04132	*cutS_2*	4396357	4396863	-	507	Carbon monoxide dehydrogenase small chain
wy_04133	*tlyC_3*	4396961	4397977	-	1017	Hemolysin C
wy_04134	*tlyC_4*	4397970	4399340	-	1371	Hemolysin C
wy_04135	*hprA*	4399567	4400499	+	933	Glycerate dehydrogenase
wy_04136	*rbtD*	4400590	4401126	+	537	Ribitol 2-dehydrogenase
wy_04137	*butA_2*	4401176	4401466	+	291	Diacetyl reductase [(S)-acetoin forming]
wy_04138	*paaI*	4401482	4401907	-	426	Acyl-coenzyme A thioesterase PaaI
wy_04139	-	4401904	4402800	-	897	hypothetical protein
wy_04140	-	4402797	4403558	-	762	Mg-protoporphyrin IX methyl transferase
wy_04141	*glpQ1_2*	4403759	4404859	-	1101	putative glycerophosphoryl diester phosphodiesterase 1
wy_04142	-	4405096	4405917	+	822	CAAX amino terminal protease self- immunity
wy_04143	*liaS_3*	4405914	4407119	+	1206	Sensor histidine kinase LiaS
wy_04144	*degU_2*	4407116	4407781	+	666	Transcriptional regulatory protein DegU
wy_04145	-	4407808	4408638	-	831	UDP-glucose 4-epimerase
wy_04146	*vsr*	4408703	4409107	-	405	Very short patch repair protein
wy_04147	*ydiP*	4409130	4410326	-	1197	putative BsuMI modification methylase subunit YdiP
wy_04148	-	4410572	4411162	+	591	hypothetical protein
wy_04149	-	4411459	4412139	-	681	hypothetical protein
wy_04150	*clpC_2*	4412698	4415178	-	2481	ATP-dependent Clp protease ATP-binding subunit ClpC
wy_0415	-	441542	441639	+	975	hypothetical protein

1		3	7			
wy_04152	-	4416550	4417050	+	501	hypothetical protein
wy_04153	*atzE*	4417184	4418518	-	1335	Biuret hydrolase
wy_04154	-	4418527	4419492	-	966	prephenate dehydrogenase
wy_04155	-	4419542	4420081	+	540	hypothetical protein
wy_04156	*tadA*	4420098	4420556	+	459	tRNA-specific adenosine deaminase
wy_04157	-	4420702	4420899	+	198	CsbD-like protein
wy_04159	-	4421104	4421220	-	117	hypothetical protein
wy_04160	-	4421531	4422373	+	843	Putative short-chain type dehydrogenase/reductase/MSMEI_5872
wy_04161	*czcO_2*	4422390	4424174	+	1785	putative oxidoreductase CzcO
wy_04162	-	4424212	4424628	+	417	hypothetical protein
wy_04163	-	4424649	4425146	-	498	DinB superfamily protein
wy_04164	*puuR_1*	4425243	4425863	+	621	HTH-type transcriptional regulator PuuR
wy_04165	-	4425881	4427284	+	1404	hypothetical protein
wy_04166	-	4427299	4427970	+	672	Phosphorylated carbohydrates phosphatase
wy_04167	*epsJ*	4428036	4428911	+	876	putative glycosyltransferase EpsJ
wy_04168	-	4428956	4430044	+	1089	hypothetical protein
wy_04169	-	4430037	4430453	+	417	hypothetical protein
wy_04170	-	4430450	4430704	+	255	hypothetical protein
wy_04171	-	4430774	4430875	-	102	hypothetical protein
wy_04172	-	4431087	4431941	+	855	Nuclease-related domain protein
wy_04173	*lsr2_1*	4432017	4432352	+	336	Nucleoid-associated protein Lsr2
wy_04174	-	4432356	4433177	+	822	hypothetical protein
wy_04175	-	4433324	4433782	+	459	DNA-binding transcriptional repressor MarR
wy_04176	*bacE*	4433779	4435110	+	1332	Putative bacilysin exporter BacE
wy_04177	*bmrR*	4435144	4436205	+	1062	Multidrug-efflux transporter 1 regulator
wy_0417	-	443622	443671	+	492	hypothetical protein

8		4	5			
wy_04179	-	4436754	4437437	+	684	hypothetical protein
wy_04180	-	4437369	4438331	-	963	Fatty acid hydroxylase superfamily protein
wy_04181	*cymR*	4438451	4438909	+	459	HTH-type transcriptional regulator CymR
wy_04182	*tam*	4438906	4439670	-	765	Trans-aconitate 2-methyltransferase
wy_04183	-	4439833	4440432	+	600	Bacterial regulatory proteins, tetR family
wy_04184	*ydfJ_5*	4440461	4442794	+	2334	Membrane protein YdfJ
wy_04185	-	4442895	4444628	+	1734	putative ABC transporter ATP-binding protein
wy_04186	-	4444625	4446553	+	1929	putative ABC transporter ATP-binding protein
wy_04187	*mmgC_9*	4446555	4447718	+	1164	Acyl-CoA dehydrogenase
wy_04188	*norB*	4447943	4450219	+	2277	Nitric oxide reductase subunit B
wy_04189	-	4450216	4450878	+	663	hypothetical protein
wy_04190	-	4450875	4451687	+	813	FtsH protease regulator HflK
wy_04191	-	4452127	4452588	+	462	Putative pyridoxine/pyridoxamine 5'-phosphate oxidase
wy_04192	-	4452601	4452942	-	342	hypothetical protein
wy_04193	-	4453002	4453439	+	438	hypothetical protein
wy_04194	-	4453482	4454282	-	801	hypothetical protein
wy_04195	*ubiE_4*	4454470	4455309	+	840	Ubiquinone/menaquinone biosynthesis C-methyltransferase UbiE
wy_04196	*tgt*	4455366	4456619	+	1254	Queuine tRNA-ribosyltransferase
wy_04197	-	4456586	4457338	-	753	hypothetical protein
wy_04198	-	4457463	4458458	-	996	hypothetical protein
wy_04199	*gltX_2*	4458566	4459480	-	915	Glutamate--tRNA ligase
wy_04200	*ctaD_3*	4459685	4461484	+	1800	Cytochrome c oxidase subunit 1
wy_04201	-	4461508	4462065	-	558	hypothetical protein
wy_04202	-	4462062	4462877	-	816	transcriptional activator FtrA
wy_04203	-	4462956	4463450	+	495	Secreted repeat of unknown function
wy_0420	*yghA_2*	446348	446440	-	921	putative oxidoreductase YghA

4		7	7			
wy_04206	-	4464947	4466239	+	1293	Putative aminotransferase/MSMEI_6121
wy_04207	*fepB*	4466313	4467344	-	1032	Ferrienterobactin-binding periplasmic protein precursor
wy_04208	-	4468335	4468577	+	243	enterobactin/ferric enterobactin esterase
wy_04209	-	4468857	4469993	+	1137	hypothetical protein
wy_04210	-	4470040	4470624	+	585	HhH-GPD superfamily base excision DNA repair protein
wy_04211	-	4470759	4471598	+	840	Methyltransferase domain protein
wy_04212	-	4471628	4472068	+	441	Glyoxalase-like domain protein
wy_04213	*ftsW_3*	4472037	4473212	-	1176	Lipid II flippase FtsW
wy_04214	-	4473587	4473985	-	399	fosfomycin resistance protein FosB
wy_04215	*yfiY_6*	4474119	4475108	+	990	putative siderophore-binding lipoprotein YfiY precursor
wy_04216	-	4475118	4476065	+	948	Pyridoxamine 5'-phosphate oxidase
wy_04217	-	4476116	4476727	-	612	transcriptional regulator BetI
wy_04218	*puo_2*	4476880	4478265	+	1386	Putrescine oxidase
wy_04219	-	4478268	4478768	+	501	Polyketide cyclase / dehydrase and lipid transport
wy_04220	*ydjH*	4478801	4479727	+	927	putative sugar kinase YdjH
wy_04221	*fucA*	4479724	4480383	+	660	L-fuculose phosphate aldolase
wy_04222	*csp_2*	4480943	4481146	-	204	Cold shock protein
wy_04223	*desA3_6*	4481712	4483082	-	1371	Stearoyl-CoA 9-desaturase
wy_04224	*nrgA*	4483628	4484938	-	1311	Ammonium transporter NrgA
wy_04225	-	4485080	4486831	-	1752	hypothetical protein
wy_04226	-	4487193	4487297	+	105	hypothetical protein
wy_04227	*kdgR_10*	4487436	4488323	-	888	Transcriptional regulator KdgR
wy_04228	-	4488541	4488990	-	450	putative acetyltransferase
wy_04229	*clcA*	4489055	4489588	-	534	Chlorocatechol 1,2-dioxygenase
wy_04230	*cdhR_2*	4489612	4490553	-	942	HTH-type transcriptional regulator CdhR
wy_0423	-	449060	449109	+	498	hypothetical protein

1		0	7			
wy_0423 2	-	449123 8	449193 3	+	696	hypothetical protein
wy_0423 3	*nreB_2*	449199 2	449395 0	+	1959	Oxygen sensor histidine kinase NreB
wy_0423 4	*degU_3*	449394 7	449458 5	+	639	Transcriptional regulatory protein DegU
wy_0423 5	*gatA_5*	449459 6	449631 1	-	1716	Glutamyl-tRNA(Gln) amidotransferase subunit A
wy_0423 6	*fadA_5*	449654 8	449776 2	+	1215	Putative acyltransferase
wy_0423 7	*fadJ_2*	449779 4	449993 5	+	2142	Fatty acid oxidation complex subunit alpha
wy_0423 8	-	450011 8	450110 1	+	984	Soluble epoxide hydrolase
wy_0423 9	-	450113 3	450256 3	-	1431	hypothetical protein
wy_0424 0	-	450282 9	450334 7	+	519	Bacterial regulatory proteins, tetR family
wy_0424 1	*fabG_18*	450334 9	450348 3	+	135	3-oxoacyl-[acyl-carrier-protein] reductase FabG
wy_0424 2	*kipR_5*	450351 3	450428 0	+	768	HTH-type transcriptional regulator KipR
wy_0424 3	*cfa*	450433 0	450562 8	-	1299	Cyclopropane-fatty-acyl-phospholipid synthase
wy_0424 4	*dprE1_2*	450562 5	450710 3	-	1479	putative decaprenylphosphoryl-beta-D-ribose oxidase
wy_0424 5	*dppA_3*	450722 3	450882 4	-	1602	Periplasmic dipeptide transport protein precursor
wy_0424 6	-	450899 5	450942 9	+	435	Polyketide cyclase / dehydrase and lipid transport
wy_0424 7	-	450950 2	450980 1	+	300	Nucleoid-associated protein/MT3819
wy_0424 8	*recR*	450981 1	451041 9	+	609	Recombination protein RecR
wy_0424 9	*catM*	451042 0	451135 5	-	936	HTH-type transcriptional regulator CatM
wy_0425 0	*ndh*	451149 6	451283 6	+	1341	NADH dehydrogenase
wy_0425 1	*baiB*	451290 4	451444 8	-	1545	Bile acid-coenzyme A ligase
wy_0425 2	-	451456 2	451547 6	-	915	EamA-like transporter family protein
wy_0425 3	*gltC_4*	451555 1	451645 3	+	903	HTH-type transcriptional regulator GltC
wy_0425 4	-	451646 0	451739 5	-	936	hypothetical protein
wy_0425 5	*gabD_4*	451740 1	451893 6	-	1536	Succinate-semialdehyde dehydrogenase [NADP(+)]
wy_0425 6	*mmgC_1 0*	451911 2	452025 4	+	1143	Acyl-CoA dehydrogenase
wy_0425	*ydfH_1*	452026	452094	+	681	putative HTH-type transcriptional regulator

7		3	3			YdfH
wy_04258	-	4520981	4521823	+	843	hypothetical protein
wy_04259	-	4521854	4522330	-	477	hypothetical protein
wy_04260	*xylB*	4522327	4523457	-	1131	Aryl-alcohol dehydrogenase
wy_04261	*mtrR*	4523454	4524716	-	1263	HTH-type transcriptional regulator MtrR
wy_04262	-	4524999	4525982	+	984	Nitronate monooxygenase
wy_04263	*paaG_2*	4525979	4526761	+	783	1,2-epoxyphenylacetyl-CoA isomerase
wy_04264	*lcfB_17*	4527028	4528626	+	1599	Long-chain-fatty-acid--CoA ligase
wy_04265	*mlaE_3*	4528703	4529512	+	810	putative phospholipid ABC transporter permease protein MlaE
wy_04266	*mlaE_4*	4529512	4530369	+	858	putative phospholipid ABC transporter permease protein MlaE
wy_04267	-	4530371	4531552	+	1182	mce related protein
wy_04268	-	4531549	4532595	+	1047	mce related protein
wy_04269	-	4532592	4533662	+	1071	mce related protein
wy_04270	-	4533659	4534774	+	1116	mce related protein
wy_04271	-	4534774	4535814	+	1041	mce related protein
wy_04272	-	4535811	4537022	+	1212	mce related protein
wy_04273	-	4537019	4537609	+	591	hypothetical protein
wy_04274	-	4537632	4538534	+	903	hypothetical protein
wy_04275	-	4538788	4539501	-	714	cobyric acid synthase
wy_04276	-	4539494	4540708	-	1215	putative bifunctional UDP-N-acetylmuramoylalanyl-D-glutamate--2, 6-diaminopimelate ligase/UDP-N-acetylmuramoyl-tripeptide:D-alanyl-D-alanine ligase
wy_04277	-	4540874	4541254	-	381	cardiolipin synthetase
wy_04278	*narX_1*	4541457	4542224	-	768	Nitrate reductase-like protein NarX
wy_04279	*narX_2*	4542221	4542826	-	606	Nitrate reductase-like protein NarX
wy_04280	*narY*	4542823	4544505	-	1683	Respiratory nitrate reductase 2 beta chain
wy_04281	*narG*	4544505	4548221	-	3717	Nitrate reductase alpha subunit

wy_04282	-	4548410	4549309	+	900	C4-dicarboxylate transporter/malic acid transport protein
wy_04283	*linC_1*	4549414	4550172	+	759	2,5-dichloro-2,5-cyclohexadiene-1,4-diol dehydrogenase
wy_04284	-	4550238	4552367	-	2130	NADH oxidase
wy_04285	-	4552494	4553024	-	531	TspO/MBR family protein
wy_04286	-	4553053	4553415	-	363	hypothetical protein
wy_04287	*leuA_3*	4553718	4555526	-	1809	2-isopropylmalate synthase
wy_04288	-	4555705	4556112	+	408	hypothetical protein
wy_04289	*pknK_2*	4556465	4559086	-	2622	Serine/threonine-protein kinase PknK
wy_04290	*atsA_4*	4559194	4561539	-	2346	Arylsulfatase
wy_04291	-	4561685	4563346	+	1662	putative sulfate transporter/MT1781
wy_04292	-	4563343	4563528	+	186	putative sulfate transporter/MT1781
wy_04293	-	4563574	4564590	+	1017	putative sulfate transporter/MT1781
wy_04294	-	4564594	4565790	-	1197	Bacterial low temperature requirement A protein (LtrA)
wy_04295	*ask*	4565926	4567191	+	1266	Aspartokinase
wy_04296	*asd*	4567197	4568228	+	1032	Aspartate-semialdehyde dehydrogenase
wy_04297	*opuD*	4568476	4570317	+	1842	Glycine betaine transporter OpuD
wy_04298	-	4570489	4572009	+	1521	Methylmalonyl-CoA carboxyltransferase 12S subunit
wy_04299	*yjgH_2*	4572051	4572416	+	366	RutC family protein YjgH
wy_04300	-	4572470	4572937	-	468	hypothetical protein
wy_04301	-	4573246	4573914	+	669	Cna protein B-type domain protein
wy_04302	-	4573996	4574583	-	588	hypothetical protein
wy_04303	*sigD_3*	4574862	4575437	-	576	ECF RNA polymerase sigma factor SigD
wy_04304	-	4575474	4576370	-	897	hypothetical protein
wy_04305	-	4576543	4577817	+	1275	putative ion channel protein
wy_04306	*hsaD_7*	4577838	4578704	-	867	4,5:9,10-diseco-3-hydroxy-5,9, 17-trioxoandrosta-1(10),2-diene-4-oate hydrolase
wy_04307	*cotA*	4578944	4580788	+	1845	Spore coat protein A

wy_04308	*sdpR_3*	4580846	4581232	+	387	Transcriptional repressor SdpR
wy_04309	-	4581229	4582320	+	1092	Sodium Bile acid symporter family protein
wy_04310	*arsC1*	4582325	4582960	+	636	Arsenate-mycothiol transferase ArsC1
wy_04311	-	4582969	4583097	-	129	hypothetical protein
wy_04312	*cadI*	4583270	4583734	-	465	Cadmium-induced protein CadI
wy_04313	-	4583831	4584199	+	369	hypothetical protein
wy_04314	*ybdK_3*	4584408	4585619	+	1212	Carboxylate-amine ligase YbdK
wy_04315	*asnB_2*	4585600	4587384	+	1785	Asparagine synthetase [glutamine-hydrolyzing] 1
wy_04316	-	4587381	4589264	+	1884	Prolyl endopeptidase precursor
wy_04317	*yxeP_4*	4589276	4590427	+	1152	putative hydrolase YxeP
wy_04318	*ubiE_5*	4590444	4591073	+	630	Ubiquinone/menaquinone biosynthesis C-methyltransferase UbiE
wy_04319	-	4591070	4591525	+	456	hypothetical protein
wy_04320	-	4591564	4592676	-	1113	Alpha/beta hydrolase family protein
wy_04321	*kdgR_11*	4592756	4593562	-	807	Transcriptional regulator KdgR
wy_04322	*hsdA_2*	4593603	4594379	-	777	3-alpha-hydroxysteroid dehydrogenase/carbonyl reductase
wy_04323	*kstR2_17*	4594509	4595138	+	630	HTH-type transcriptional repressor KstR2
wy_04324	-	4595185	4595610	+	426	Polyketide cyclase / dehydrase and lipid transport
wy_04325	*furA*	4595795	4596349	+	555	Transcriptional regulator FurA
wy_04326	*katA_2*	4596346	4597812	+	1467	Catalase
wy_04327	-	4597952	4598149	+	198	hypothetical protein
wy_04328	-	4598206	4598409	+	204	hypothetical protein
wy_04329	*gadA*	4598586	4599848	-	1263	Glutamate decarboxylase alpha
wy_04330	-	4599848	4600069	-	222	hypothetical protein
wy_04331	*yjmB*	4600069	4601433	-	1365	putative symporter YjmB
wy_04332	-	4601590	4602402	+	813	inner membrane protein
wy_04333	-	4602443	4603552	-	1110	hypothetical protein

wy_04334	-	4603792	4605594	+	1803	Cholesterol oxidase, substrate-binding
wy_04335	-	4605672	4606100	+	429	hypothetical protein
wy_04336	*yjcA*	4606324	4606788	+	465	Sporulation protein YjcA
wy_04337	*carC*	4606822	4607694	+	873	2-hydroxy-6-oxo-6-(2'-aminophenyl)hexa-2, 4-dienoic acid hydrolase
wy_04338	*yfiY_7*	4607768	4608808	-	1041	putative siderophore-binding lipoprotein YfiY precursor
wy_04339	-	4608853	4609329	-	477	Endoribonuclease L-PSP
wy_04340	-	4609361	4610737	-	1377	putative FAD-linked oxidoreductase
wy_04341	-	4610749	4612326	-	1578	N-acyl-D-glutamate deacylase
wy_04342	-	4612339	4613691	-	1353	Na+/H+ antiporter family protein
wy_04343	*hcaR_3*	4613943	4614803	+	861	Hca operon transcriptional activator
wy_04345	-	4614987	4615967	-	981	putative metallophosphoesterase
wy_04346	-	4616032	4616508	+	477	Yqey-like protein
wy_04347	-	4616618	4617409	+	792	hypothetical protein
wy_04348	*yraA*	4617413	4617976	-	564	Putative cysteine protease YraA
wy_04349	-	4617973	4618614	-	642	hypothetical protein
wy_04350	-	4618698	4618853	-	156	hypothetical protein
wy_04351	*pbpG*	4618947	4621196	-	2250	Penicillin-binding protein 2D
wy_04352	*whiB4*	4621577	4621900	+	324	Transcriptional regulator WhiB4
wy_04353	*arsA_2*	4622014	4623153	-	1140	Arsenical pump-driving ATPase
wy_04354	-	4623150	4624190	-	1041	Anion-transporting ATPase
wy_04355	-	4624246	4624410	+	165	hypothetical protein
wy_04356	*tdcF*	4624416	4624886	+	471	Putative reactive intermediate deaminase TdcF
wy_04357	-	4624934	4625584	-	651	Bacterial regulatory proteins, tetR family
wy_04358	*gdh_3*	4625714	4626478	+	765	Glucose 1-dehydrogenase
wy_04359	-	4626605	4627390	+	786	putative metallo-hydrolase
wy_04360	-	4627425	4627994	-	570	hypothetical protein

wy_04361	*rapA*	4627991	4629748	-	1758	RNA polymerase-associated protein RapA
wy_04362	-	4629800	4630621	+	822	hypothetical protein
wy_04363	*amnC*	4630727	4632202	+	1476	2-aminomuconic 6-semialdehyde dehydrogenase
wy_04364	*amnE*	4632222	4633001	+	780	4-oxalocrotonate decarboxylase
wy_04365	*amnD*	4632998	4633456	+	459	2-aminomuconate deaminase
wy_04366	*nbaC*	4633453	4633974	+	522	3-hydroxyanthranilate 3,4-dioxygenase
wy_04367	-	4633976	4634971	+	996	Amidohydrolase
wy_04368	*yhjE_4*	4635142	4636485	+	1344	Inner membrane metabolite transport protein YhjE
wy_04369	*gbpR*	4636548	4637480	-	933	HTH-type transcriptional regulator GbpR
wy_04370	*nlhH_8*	4637581	4638435	-	855	Carboxylesterase NlhH
wy_04371	*crp*	4638595	4639269	-	675	cAMP receptor protein
wy_04372	-	4639480	4640517	-	1038	hypothetical protein
wy_04373	*yhdG_3*	4640514	4641860	-	1347	putative amino acid permease YhdG
wy_04374	-	4641935	4642189	-	255	hypothetical protein
wy_04375	*pdg*	4642347	4643045	+	699	Ultraviolet N-glycosylase/AP lyase
wy_04376	*resA_3*	4643057	4643719	+	663	Thiol-disulfide oxidoreductase ResA
wy_04377	-	4643716	4644489	+	774	putative NUDIX hydrolase
wy_04378	-	4644486	4645676	+	1191	Serine protease
wy_04379	-	4645705	4646640	-	936	Soluble epoxide hydrolase
wy_04380	-	4646661	4647185	-	525	hypothetical protein
wy_04381	*nhaA*	4647243	4648451	-	1209	Na(+)/H(+) antiporter NhaA
wy_04382	*yhjE_5*	4648680	4650083	+	1404	Inner membrane metabolite transport protein YhjE
wy_04383	-	4650294	4651031	+	738	hypothetical protein
wy_04384	-	4651119	4651481	-	363	hypothetical protein
wy_04385	-	4651635	4653950	-	2316	Putative HTH-type transcriptional regulator/MT0914
wy_04386	*acsA_4*	4654081	4656009	-	1929	Acetyl-coenzyme A synthetase

wy_04387	*oppA*	4656282	4657901	+	1620	Periplasmic oligopeptide-binding protein precursor
wy_04388	*dppB_3*	4657904	4658830	+	927	Dipeptide transport system permease protein DppB
wy_04389	*oppC_2*	4658823	4659683	+	861	Oligopeptide transport system permease protein OppC
wy_04390	*gsiA_2*	4659680	4661290	+	1611	Glutathione import ATP-binding protein GsiA
wy_04391	-	4661302	4662054	+	753	hypothetical protein
wy_04392	-	4662478	4663296	-	819	haloacid dehalogenase-like hydrolase
wy_04393	-	4663695	4664813	+	1119	hypothetical protein
wy_04394	-	4664810	4665988	+	1179	Putative conjugal transfer protein/MT3759
wy_04395	-	4665985	4666791	+	807	hypothetical protein
wy_04396	-	4666791	4667387	+	597	Bacterial type II secretion system protein F domain protein
wy_04397	-	4667448	4667651	+	204	hypothetical protein
wy_04398	-	4667648	4668004	+	357	hypothetical protein
wy_04399	-	4668001	4668372	+	372	hypothetical protein
wy_04400	-	4668378	4670744	-	2367	putative ATP-dependent helicase Lhr
wy_04401	*cspA_2*	4671121	4671324	+	204	putative cold shock protein A
wy_04402	-	4671515	4672099	+	585	hypothetical protein
wy_04403	*topA*	4672245	4675133	+	2889	DNA topoisomerase 1
wy_04404	*dnaX*	4675178	4676365	+	1188	DNA polymerase III subunit tau
wy_04406	-	4676617	4676781	+	165	hypothetical protein
wy_04407	-	4676827	4677024	+	198	hypothetical protein
wy_04411	*lutR_4*	4683092	4683781	+	690	HTH-type transcriptional regulator LutR
wy_04412	*ylmA_2*	4683778	4684599	+	822	putative ABC transporter ATP-binding protein YlmA
wy_04413	*ydfH_2*	4684645	4685298	+	654	putative HTH-type transcriptional regulator YdfH
wy_04414	-	4685295	4686560	+	1266	multidrug efflux system protein MdtL
wy_04415	*pepN_3*	4686546	4687862	-	1317	Aminopeptidase N
wy_04416	*lgrD_2*	4687859	4691758	-	3900	Linear gramicidin synthase subunit D

wy_04417	-	4692000	4692707	+	708	YCII-related domain protein
wy_04418	-	4692814	4693671	+	858	Sigma-70, region 4
wy_04419	*sufS_2*	4693911	4695227	+	1317	Cysteine desulfurase
wy_04420	-	4695421	4696056	+	636	hypothetical protein
wy_04421	-	4696067	4696825	-	759	hypothetical protein
wy_04422	*bdhA_2*	4696846	4697631	-	786	D-beta-hydroxybutyrate dehydrogenase
wy_04423	*snaC*	4698203	4698757	-	555	NADH:riboflavin 5'-phosphate oxidoreductase
wy_04424	*proS_2*	4698881	4699354	-	474	Proline--tRNA ligase
wy_04425	-	4699370	4700482	-	1113	hypothetical protein
wy_04426	*acrR_3*	4700609	4701184	+	576	HTH-type transcriptional regulator AcrR
wy_04427	-	4701178	4701699	-	522	OHCU decarboxylase
wy_04428	-	4701918	4702271	+	354	Glyoxalase-like domain protein
wy_04429	-	4702302	4702739	+	438	Carboxymuconolactone decarboxylase family protein
wy_04430	*echA8_8*	4702778	4703608	+	831	putative enoyl-CoA hydratase echA8
wy_04431	*bmr3_8*	4703642	4705318	+	1677	Multidrug resistance protein 3
wy_04432	*sigI_2*	4705341	4706204	-	864	putative ECF RNA polymerase sigma factor SigI
wy_04433	-	4706206	4706679	-	474	Carboxymuconolactone decarboxylase family protein
wy_04434	*lexA_2*	4706803	4707465	-	663	LexA repressor
wy_04435	*ppa*	4707563	4708054	-	492	Inorganic pyrophosphatase
wy_04436	*dac*	4708234	4709643	+	1410	D-alanyl-D-alanine carboxypeptidase precursor
wy_04437	-	4709665	4710726	+	1062	hypothetical protein
wy_04438	*tilS*	4710723	4711712	+	990	tRNA(Ile)-lysidine synthase
wy_04439	*hpt*	4711725	4712279	+	555	Hypoxanthine phosphoribosyltransferase
wy_04440	*pknB_2*	4712240	4714579	-	2340	Serine/threonine-protein kinase PknB
wy_04441	*aam_3*	4714679	4716031	+	1353	Acylamidase
wy_04442	*ftsH_3*	4716293	4718626	+	2334	ATP-dependent zinc metalloprotease FtsH

wy_04443	*folE*	4718711	4719328	+	618	GTP cyclohydrolase 1
wy_04444	*folP1*	4719325	4720170	+	846	Dihydropteroate synthase 1
wy_04445	*folB*	4720196	4720615	+	420	putative dihydroneopterin aldolase
wy_04446	*sulD*	4720612	4721118	+	507	Bifunctional folate synthesis protein
wy_04447	-	4721124	4721591	+	468	hypothetical protein
wy_04448	-	4721742	4722665	+	924	hypothetical protein
wy_04449	-	4722800	4723726	+	927	Rossmann-like domain protein
wy_04450	*panC*	4723723	4724667	+	945	Pantothenate synthetase
wy_04451	*panD*	4724700	4725122	+	423	Aspartate 1-decarboxylase precursor
wy_04452	*coaX*	4725127	4725939	+	813	Type III pantothenate kinase
wy_04453	*cdoA*	4726090	4726674	+	585	Cysteine dioxygenase
wy_04454	-	4726671	4727093	+	423	molybdopterin biosynthesis protein MoeB
wy_04455	*lysS*	4727176	4728687	+	1512	Lysine--tRNA ligase
wy_04456	*lsr2_2*	4728815	4729168	+	354	Nucleoid-associated protein Lsr2
wy_04457	-	4729856	4731706	+	1851	hypothetical protein
wy_04458	-	4731703	4732866	+	1164	Acyltransferase family protein
wy_04459	*sigI_3*	4732965	4733699	+	735	putative ECF RNA polymerase sigma factor SigI
wy_04460	*clpC1_2*	4733968	4736523	+	2556	ATP-dependent Clp protease ATP-binding subunit ClpC1
wy_04461	-	4736575	4737201	-	627	hypothetical protein
wy_04462	*stp_5*	4737291	4738886	+	1596	Multidrug resistance protein stp
wy_04463	-	4738893	4739540	-	648	hypothetical protein
wy_04464	*osmC*	4739607	4740041	+	435	Peroxiredoxin OsmC
wy_04465	*scnB_1*	4740093	4740425	-	333	Thiocyanate hydrolase subunit beta
wy_04466	*scnC*	4740422	4741096	-	675	Thiocyanate hydrolase subunit gamma
wy_04467	*scnA*	4741106	4741405	-	300	Thiocyanate hydrolase subunit alpha
wy_04468	*scnB_2*	4741402	4741836	-	435	Thiocyanate hydrolase subunit beta

wy_04469	-	4741906	4742724	-	819	putative cobalt transporter subunit (CbtA)
wy_04470	-	4742715	4742909	-	195	hypothetical protein
wy_04471	-	4743228	4743836	-	609	Telomeric repeat-binding factor 2
wy_04472	*desK_2*	4744004	4745248	+	1245	Sensor histidine kinase DesK
wy_04473	*degU_4*	4745390	4745953	+	564	Transcriptional regulatory protein DegU
wy_04474	-	4746129	4746383	+	255	hypothetical protein
wy_04475	*fadK_3*	4746352	4747812	+	1461	Short-chain-fatty-acid--CoA ligase
wy_04476	-	4747853	4748791	+	939	DSBA-like thioredoxin domain protein
wy_04477	-	4748636	4749445	-	810	putative cobalt transporter subunit (CbtA)
wy_04478	-	4749436	4749630	-	195	hypothetical protein
wy_04479	*mhuD*	4749874	4750188	-	315	Heme-degrading monooxygenase HmoB
wy_04480	*bpoC_1*	4750259	4750996	+	738	Putative non-heme bromoperoxidase BpoC
wy_04481	*mutY*	4751003	4751923	-	921	A/G-specific adenine glycosylase
wy_04482	*cynT_2*	4751987	4752628	+	642	Carbonic anhydrase
wy_04483	-	4752790	4753464	+	675	hypothetical protein
wy_04484	*disA*	4753493	4754587	-	1095	DNA integrity scanning protein DisA
wy_04485	-	4754718	4755233	+	516	Thioesterase superfamily protein
wy_04486	-	4755237	4756625	-	1389	hypothetical protein
wy_04487	-	4756704	4757300	-	597	hypothetical protein
wy_04488	*carD*	4757598	4758086	+	489	RNA polymerase-binding transcription factor CarD
wy_04489	-	4758052	4758705	-	654	hypothetical protein
wy_04490	*ispF*	4758788	4759264	+	477	2-C-methyl-D-erythritol 2,4-cyclodiphosphate synthase
wy_04491	*cysS*	4759276	4760685	+	1410	Cysteine--tRNA ligase
wy_04492	-	4760685	4761650	+	966	Putative TrmH family tRNA/rRNA methyltransferase
wy_04493	*kipA_2*	4761654	4762517	-	864	KipI antagonist
wy_04494	-	4762564	4763253	+	690	ZIP Zinc transporter

wy_04495	*kipI*	4763418	4764023	-	606	Kinase A inhibitor
wy_04496	-	4764023	4764775	-	753	LamB/YcsF family protein
wy_04497	-	4764830	4765624	-	795	hypothetical protein
wy_04498	*sinR_2*	4765706	4766254	-	549	HTH-type transcriptional regulator SinR
wy_04499	*novR_4*	4766414	4767241	+	828	Decarboxylase NovR
wy_04500	*dapA_2*	4767238	4768125	+	888	4-hydroxy-tetrahydrodipicolinate synthase
wy_04501	-	4768179	4768637	+	459	putative acetyltransferase
wy_04502	*znuB*	4768704	4769603	-	900	High-affinity zinc uptake system membrane protein ZnuB
wy_04503	*znuC*	4769600	4770445	-	846	Zinc import ATP-binding protein ZnuC
wy_04504	-	4770442	4771401	-	960	putative periplasmic iron-binding protein precursor
wy_04505	*purR*	4771496	4772656	+	1161	HTH-type transcriptional repressor PurR
wy_04506	-	4772653	4775217	-	2565	Trehalase
wy_04507	*kstR_7*	4775285	4775923	-	639	HTH-type transcriptional repressor KstR
wy_04508	*bbsG_2*	4776017	4778197	+	2181	(R)-benzylsuccinyl-CoA dehydrogenase
wy_04509	*caiA*	4778500	4779573	+	1074	Crotonobetainyl-CoA dehydrogenase
wy_04510	-	4779558	4780706	+	1149	Acyl-CoA dehydrogenase fadE12
wy_04511	-	4780703	4781710	+	1008	hypothetical protein
wy_04512	-	4781712	4782134	+	423	hypothetical protein
wy_04513	-	4782131	4783300	+	1170	lipid-transfer protein
wy_04514	*nlhH_9*	4783455	4784417	+	963	Carboxylesterase NlhH
wy_04515	*ntaA_2*	4784521	4785816	-	1296	Nitrilotriacetate monooxygenase component A
wy_04516	*ydbC_2*	4785813	4786760	-	948	Putative oxidoreductase YdbC
wy_04517	*linC_2*	4786757	4787479	-	723	2,5-dichloro-2,5-cyclohexadiene-1,4-diol dehydrogenase
wy_04518	*arnB*	4787479	4788708	-	1230	UDP-4-amino-4-deoxy-L-arabinose--oxoglutarate aminotransferase
wy_04519	*limC_2*	4788760	4789560	-	801	(-)-trans-carveol dehydrogenase
wy_04520	-	4789659	4790141	-	483	hypothetical protein

wy_04521	*kipR_6*	4790435	4791103	+	669	HTH-type transcriptional regulator KipR
wy_04522	*atsA_5*	4791140	4793479	-	2340	Arylsulfatase
wy_04523	-	4793689	4794291	+	603	putative DNA-binding transcriptional regulator
wy_04524	*emrB_2*	4794234	4795754	-	1521	Multidrug export protein EmrB
wy_04525	*tauD_4*	4796165	4797082	+	918	Alpha-ketoglutarate-dependent taurine dioxygenase
wy_04526	-	4797092	4798354	+	1263	hypothetical protein
wy_04527	*yidH*	4798389	4798751	+	363	Inner membrane protein YidH
wy_04528	-	4798748	4799083	+	336	hypothetical protein
wy_04529	*limC_3*	4799251	4800087	+	837	(-)-trans-carveol dehydrogenase
wy_04530	*stp_6*	4800084	4801544	+	1461	Multidrug resistance protein stp
wy_04531	-	4801548	4801943	-	396	Interferon-induced transmembrane protein
wy_04532	-	4802084	4803673	-	1590	Cholesterol oxidase, substrate-binding
wy_04533	-	4804032	4804127	-	96	hypothetical protein
wy_04534	-	4804075	4804893	-	819	MaoC like domain protein
wy_04535	*kstD_4*	4804906	4806609	-	1704	3-oxosteroid 1-dehydrogenase
wy_04536	*kshA_8*	4807166	4808326	-	1161	3-ketosteroid-9-alpha-monooxygenase oxygenase subunit
wy_04537	*hsaA_6*	4808517	4809692	+	1176	Flavin-dependent monooxygenase, oxygenase subunit HsaA
wy_04538	*hsaD_8*	4809702	4810607	+	906	4,5:9,10-diseco-3-hydroxy-5,9, 17-trioxoandrosta-1(10),2-diene-4-oate hydrolase
wy_04539	*hsaC_4*	4810607	4811506	+	900	Iron-dependent extradiol dioxygenase
wy_04540	*hsaB_2*	4811506	4812093	+	588	Flavin-dependent monooxygenase, reductase subunit HsaB
wy_04541	-	4812341	4812796	+	456	Polyketide cyclase / dehydrase and lipid transport
wy_04542	-	4812852	4814909	-	2058	Neutral ceramidase precursor
wy_04543	-	4815023	4815754	-	732	Acetoacetate decarboxylase (ADC)
wy_04544	*hdhA_5*	4815751	4816611	-	861	7-alpha-hydroxysteroid dehydrogenase
wy_04545	-	4816732	4817955	+	1224	Putative cytochrome P450 142
wy_0454	-	481790	481847	-	573	hypothetical protein

6		6	8			
wy_04547	-	4818543	4819085	-	543	hypothetical protein
wy_04548	-	4819285	4819830	-	546	hypothetical protein
wy_04549	-	4819884	4820594	-	711	hypothetical protein
wy_04550	-	4820635	4821063	-	429	Cupin domain protein
wy_04551	-	4821157	4822032	-	876	putative oxidoreductase/MSMEI_2347
wy_04552	*baiE_5*	4822073	4822648	-	576	Bile acid 7-alpha dehydratase
wy_04553	*fabG_19*	4822696	4823505	+	810	3-oxoacyl-[acyl-carrier-protein] reductase FabG
wy_04554	*yidA*	4823591	4824454	+	864	Sugar phosphatase YidA
wy_04555	*arpA_5*	4824495	4825139	+	645	A-factor receptor protein
wy_04556	*mmgC_11*	4825209	4826345	-	1137	Acyl-CoA dehydrogenase
wy_04557	*acrC_5*	4826342	4827328	-	987	Acryloyl-CoA reductase (NADH)
wy_04558	*acdA_10*	4827325	4828464	-	1140	Acyl-CoA dehydrogenase
wy_04559	*menB_2*	4828464	4829327	-	864	1,4-Dihydroxy-2-naphthoyl-CoA synthase
wy_04560	*lcfB_18*	4829324	4830913	-	1590	Long-chain-fatty-acid--CoA ligase
wy_04561	*mmgC_12*	4830979	4832190	+	1212	Acyl-CoA dehydrogenase
wy_04562	*lvr*	4832187	4832996	+	810	Levodione reductase
wy_04563	*kstR2_18*	4833141	4833764	+	624	HTH-type transcriptional repressor KstR2
wy_04564	*pcaF_3*	4833784	4834938	+	1155	Beta-ketoadipyl-CoA thiolase
wy_04565	*bsmB*	4835084	4835779	-	696	Dimethylglycine N-methyltransferase
wy_04566	*mhpC_2*	4835878	4836957	-	1080	2-hydroxy-6-oxononadienedioate/2-hydroxy-6-oxononatrienedioate hydrolase
wy_04567	-	4837051	4837962	-	912	Sodium Bile acid symporter family protein
wy_04568	*cypE*	4838106	4839461	-	1356	putative bifunctional P-450/NADPH-P450 reductase 2
wy_04569	-	4839566	4839853	+	288	hypothetical protein
wy_04570	-	4840156	4841334	+	1179	hypothetical protein
wy_04571	-	4841390	4842169	+	780	hypothetical protein
wy_0457	-	484216	484294	+	780	hypothetical protein

2		6	5			
wy_0457 3	-	484301 7	484408 1	-	1065	Nitronate monooxygenase
wy_0457 4	*catJ_2*	484407 8	484482 7	-	750	3-oxoadipate CoA-transferase subunit B
wy_0457 5	*gctA_2*	484482 4	484571 4	-	891	Glutaconate CoA-transferase subunit A
wy_0457 6	*caiD_6*	484571 4	484649 9	-	786	Carnitinyl-CoA dehydratase
wy_0457 7	*fabG_20*	484659 7	484736 1	+	765	3-oxoacyl-[acyl-carrier-protein] reductase FabG
wy_0457 8	-	484737 1	484828 5	+	915	Putative short-chain type dehydrogenase/reductase
wy_0457 9	-	484835 4	484902 5	-	672	DNA gyrase subunit A
wy_0458 0	*ddn_2*	484912 8	484959 8	-	471	Deazaflavin-dependent nitroreductase
wy_0458 1	*fadA_6*	484961 5	485077 8	-	1164	3-ketoacyl-CoA thiolase
wy_0458 2	-	485095 4	485220 7	+	1254	Steroid C26-monooxygenase
wy_0458 3	-	485238 7	485280 6	-	420	SnoaL-like domain protein
wy_0458 4	*rutA_6*	485281 6	485367 9	-	864	Pyrimidine monooxygenase RutA
wy_0458 5	-	485369 2	485486 7	-	1176	acetyl-CoA acetyltransferase
wy_0458 6	-	485486 9	485593 3	-	1065	lipid-transfer protein
wy_0458 7	-	485594 7	485690 3	-	957	hypothetical protein
wy_0458 8	-	485699 8	485803 8	+	1041	Phthiodiolone/phenolphthiodiolone dimycocerosates ketoreductase
wy_0458 9	*fabG3_2*	485811 4	485887 5	-	762	3-alpha-(or 20-beta)-hydroxysteroid dehydrogenase
wy_0459 0	*fadA_7*	485890 4	486009 7	-	1194	3-ketoacyl-CoA thiolase
wy_0459 1	*kstR2_1 9*	486018 5	486080 5	-	621	HTH-type transcriptional repressor KstR2
wy_0459 2	*paaF_2*	486094 3	486176 4	-	822	2,3-dehydroadipyl-CoA hydratase
wy_0459 3	-	486180 2	486349 3	+	1692	Long-chain-fatty-acid--CoA ligase FadD19
wy_0459 4	-	486349 3	486460 8	+	1116	Nitronate monooxygenase
wy_0459 5	-	486468 2	486631 3	-	1632	Long-chain-fatty-acid--CoA ligase FadD17
wy_0459 6	-	486638 9	486751 0	-	1122	Acyl-CoA dehydrogenase, short-chain specific
wy_0459 7	*mmgC_1 3*	486752 6	486871 3	-	1188	Acyl-CoA dehydrogenase
wy_0459	*subB*	486898	486917	+	189	Ferredoxin-2

8		9	7			
wy_04599	-	4869297	4870208	+	912	Putative short-chain type dehydrogenase/reductase
wy_04600	*mlaE_5*	4870839	4871603	+	765	putative phospholipid ABC transporter permease protein MlaE
wy_04601	*mlaE_6*	4871624	4872475	+	852	putative phospholipid ABC transporter permease protein MlaE
wy_04602	-	4872488	4873666	+	1179	mce related protein
wy_04603	-	4873668	4874687	+	1020	mce related protein
wy_04604	-	4874684	4875742	+	1059	mce related protein
wy_04605	-	4875739	4877118	+	1380	mce related protein
wy_04606	-	4878349	4879752	+	1404	mce related protein
wy_04607	-	4879749	4880537	+	789	hypothetical protein
wy_04608	-	4880534	4881040	+	507	hypothetical protein
wy_04609	*otsA_2*	4881112	4882641	-	1530	Trehalose-phosphate synthase
wy_04610	-	4882675	4882848	-	174	hypothetical protein
wy_04611	*yheI*	4882984	4884681	+	1698	putative multidrug resistance ABC transporter ATP-binding/permease protein YheI
wy_04612	-	4884678	4886456	+	1779	putative ABC transporter ATP-binding protein
wy_04613	-	4886453	4886971	-	519	hypothetical protein
wy_04614	*iphP_4*	4887000	4887827	-	828	Tyrosine-protein phosphatase precursor
wy_04615	-	4888165	4889832	+	1668	Calcineurin-like phosphoesterase
wy_04616	-	4889941	4890423	-	483	DinB superfamily protein
wy_04617	*arnA*	4890479	4891417	-	939	Bifunctional polymyxin resistance protein ArnA
wy_04618	*bpoC_2*	4891530	4892330	+	801	Putative non-heme bromoperoxidase BpoC
wy_04619	*lgrD_3*	4892430	4909163	+	16734	Linear gramicidin synthase subunit D
wy_04620	*pvdA*	4909284	4910639	+	1356	L-ornithine 5-monooxygenase
wy_04621	-	4910636	4910869	+	234	MbtH-like protein
wy_04622	-	4910884	4912338	-	1455	hypothetical protein
wy_04623	-	4912340	4912720	-	381	hypothetical protein

wy_04625	-	4912946	4913128	-	183	hypothetical protein
wy_04626	-	4913364	4913549	-	186	hypothetical protein
wy_04627	*eryG*	4913675	4914313	-	639	Erythromycin 3"-O-methyltransferase
wy_04628	*trxB_2*	4914310	4915287	-	978	Thioredoxin reductase
wy_04629	*puuR_2*	4915379	4915969	+	591	HTH-type transcriptional regulator PuuR
wy_04630	-	4915984	4916115	-	132	hypothetical protein
wy_04631	*clcD*	4916357	4917067	+	711	Carboxymethylenebutenolidase
wy_04632	-	4917038	4917883	-	846	hypothetical protein
wy_04633	*sugE*	4917987	4918301	-	315	Quaternary ammonium compound-resistance protein SugE
wy_04634	*tcrX*	4918530	4919204	+	675	putative transcriptional regulatory protein TcrX
wy_04635	*tcrY_2*	4919213	4920607	+	1395	putative sensor histidine kinase TcrY
wy_04636	-	4920612	4921067	-	456	HIT-like protein
wy_04637	-	4921218	4921880	+	663	hypothetical protein
wy_04638	-	4922012	4923250	+	1239	Undecaprenyl-phosphate mannosyltransferase
wy_04639	-	4923247	4925247	+	2001	hypothetical protein
wy_04640	-	4925295	4925537	+	243	hypothetical protein
wy_04641	-	4925653	4926519	+	867	Phytanoyl-CoA dioxygenase (PhyH)
wy_04642	*purD*	4926570	4927823	+	1254	Phosphoribosylamine--glycine ligase
wy_04643	*aspC*	4927992	4929125	+	1134	Aspartate aminotransferase
wy_04644	-	4929233	4929796	+	564	Bacterial regulatory proteins, tetR family
wy_04645	*purB*	4929833	4931254	+	1422	Adenylosuccinate lyase
wy_04646	-	4931251	4931955	+	705	Dienelactone hydrolase family protein
wy_04647	*yusO_2*	4932032	4932340	-	309	putative HTH-type transcriptional regulator YusO
wy_04648	-	4932557	4934506	+	1950	Putative multidrug export ATP-binding/permease protein
wy_04649	*urdA*	4934357	4935835	-	1479	Urocanate reductase precursor
wy_04650	-	4935961	4937172	-	1212	Cytochrome P450 144

wy_04651	-	4937429	4937980	+	552	Bacterial regulatory proteins, tetR family
wy_04652	-	4937992	4938420	+	429	HIT-like protein
wy_04653	*dcyD*	4938413	4939429	+	1017	D-cysteine desulfhydrase
wy_04654	-	4939435	4939626	-	192	hypothetical protein
wy_04655	-	4940079	4940921	-	843	Putative short-chain type dehydrogenase/reductase/MSMEI_5872
wy_04656	*ycnE*	4940951	4941277	-	327	Putative monooxygenase YcnE
wy_04657	*asnC*	4941429	4941902	-	474	Regulatory protein AsnC
wy_04658	-	4942004	4943053	-	1050	Alcohol dehydrogenase
wy_04659	-	4943055	4943981	-	927	putative transporter
wy_04660	-	4943978	4944541	-	564	Amino acid permease
wy_04661	*thcD_3*	4944690	4945874	-	1185	Rhodocoxin reductase
wy_04662	*fas2*	4945880	4946074	-	195	Ferredoxin fas2
wy_04663	*antA*	4946097	4947209	-	1113	Anthranilate 1,2-dioxygenase large subunit
wy_04664	*ebrB_1*	4947388	4947768	+	381	Multidrug resistance protein EbrB
wy_04665	*ebrB_2*	4947765	4948094	+	330	Multidrug resistance protein EbrB
wy_04666	-	4948264	4949055	+	792	3-oxo-5-alpha-steroid 4-dehydrogenase
wy_04667	-	4949803	4950339	-	537	Polyketide cyclase / dehydrase and lipid transport
wy_04668	-	4950332	4950496	-	165	hypothetical protein
wy_04669	*nemA*	4950558	4951661	-	1104	N-ethylmaleimide reductase
wy_04670	*cpnA_2*	4951772	4952572	-	801	Cyclopentanol dehydrogenase
wy_04671	*proP_5*	4952702	4954078	-	1377	Proline/betaine transporter
wy_04672	-	4954891	4955802	+	912	DNA-binding transcriptional repressor AcrR
wy_04673	-	4955896	4956966	-	1071	S-(hydroxymethyl)mycothiol dehydrogenase
wy_04674	*kshA_9*	4957452	4958495	+	1044	3-ketosteroid-9-alpha-monooxygenase oxygenase subunit
wy_04675	*mmgC_14*	4958495	4959652	+	1158	Acyl-CoA dehydrogenase
wy_04676	*acrC_6*	4959661	4960737	+	1077	Acryloyl-CoA reductase (NADH)

wy_04677	*menB_3*	4960740	4961546	+	807	1,4-Dihydroxy-2-naphthoyl-CoA synthase
wy_04678	*fadK_4*	4961543	4963114	+	1572	Short-chain-fatty-acid--CoA ligase
wy_04679	*hapE_8*	4963214	4965127	+	1914	4-hydroxyacetophenone monooxygenase
wy_04680	*cpnA_3*	4965358	4966113	+	756	Cyclopentanol dehydrogenase
wy_04681	*fgd_4*	4966238	4967239	+	1002	F420-dependent glucose-6-phosphate dehydrogenase
wy_04682	*adh_7*	4967243	4968295	+	1053	Alcohol dehydrogenase
wy_04683	-	4968672	4969562	+	891	Putative cyclase
wy_04684	-	4969605	4970066	+	462	Glyoxalase-like domain protein
wy_04685	*penE*	4970136	4971977	+	1842	Pentalenolactone D synthase
wy_04686	-	4972023	4972817	+	795	hypothetical protein
wy_04687	*gerKI*	4972947	4973879	+	933	dTDP-4-oxo-6-deoxy-D-allose reductase
wy_04688	*mlhB*	4973912	4974790	+	879	Monoterpene epsilon-lactone hydrolase
wy_04689	*stp_7*	4975097	4976611	+	1515	Multidrug resistance protein stp
wy_04690	*alkH*	4976981	4978249	-	1269	Aldehyde dehydrogenase
wy_04691	-	4978401	4978502	-	102	hypothetical protein
wy_04692	-	4978553	4978843	-	291	Rdx family protein
wy_04693	-	4978840	4979592	-	753	Ferredoxin, 2Fe-2S
wy_04694	*fhuC_2*	4979589	4980401	-	813	Iron(3+)-hydroxamate import ATP-binding protein FhuC
wy_04695	*hmuU_3*	4980398	4981456	-	1059	Hemin transport system permease protein HmuU
wy_04696	-	4981453	4982487	-	1035	corrinoid ABC transporter substrate-binding protein
wy_04697	-	4982752	4983810	-	1059	iron-dicitrate transporter substrate-binding subunit
wy_04698	*bmr3_9*	4983934	4985352	-	1419	Multidrug resistance protein 3
wy_04699	*purC*	4985664	4986548	+	885	Phosphoribosylaminoimidazole-succinocarboxamide synthase
wy_04700	*ptrB*	4986550	4988679	+	2130	Protease 2
wy_04701	*dhbB*	4988748	4989398	-	651	Isochorismatase
wy_04702	*dhbE*	4989406	4991088	-	1683	2,3-dihydroxybenzoate-AMP ligase

wy_04703	*dhbA*	4991139	4991912	-	774	2,3-dihydro-2,3-dihydroxybenzoate dehydrogenase
wy_04704	*dhbC_2*	4991909	4993054	-	1146	Isochorismate synthase DhbC
wy_04705	*dhbF_2*	4993232	5000974	+	7743	Dimodular nonribosomal peptide synthase
wy_04706	*yfmC*	5001084	5002103	+	1020	Fe(3+)-citrate-binding protein YfmC precursor
wy_04707	*lgrD_4*	5002195	5011362	+	9168	Linear gramicidin synthase subunit D
wy_04708	*mbtB*	5011399	5011623	+	225	Phenyloxazoline synthase MbtB
wy_04709	*entS_2*	5011631	5012905	+	1275	Enterobactin exporter EntS
wy_04710	*yqjH*	5012921	5014744	+	1824	NADPH-dependent ferric-chelate reductase
wy_04711	*yhjE_6*	5014795	5016147	-	1353	Inner membrane metabolite transport protein YhjE
wy_04712	*gpx2*	5016338	5016832	+	495	Hydroperoxy fatty acid reductase gpx2
wy_04713	-	5016933	5017637	+	705	hypothetical protein
wy_04714	*tetR_3*	5017648	5018394	-	747	Tetracycline repressor protein class E
wy_04715	-	5018479	5018856	+	378	Glyoxalase-like domain protein
wy_04716	-	5018870	5019517	-	648	metal-dependent hydrolase
wy_04717	-	5019659	5019895	+	237	phosphoribosylformylglycinamidine synthase subunit PurS
wy_04718	*purQ*	5019892	5020569	+	678	Phosphoribosylformylglycinamidine synthase 1
wy_04719	*dcuR*	5020698	5021408	-	711	Transcriptional regulatory protein DcuR
wy_04720	*dcuS*	5021405	5023042	-	1638	Sensor histidine kinase DcuS
wy_04721	*dctA_2*	5023191	5024618	+	1428	Aerobic C4-dicarboxylate transport protein
wy_04722	-	5024671	5025798	+	1128	hypothetical protein
wy_04723	-	5025795	5026274	+	480	hypothetical protein
wy_04724	-	5026307	5026657	+	351	Glyoxalase-like domain protein
wy_04725	*apeB*	5026720	5028021	+	1302	putative M18 family aminopeptidase 2
wy_04726	*purL*	5028163	5030442	+	2280	Phosphoribosylformylglycinamidine synthase 2
wy_04727	-	5030446	5032227	+	1782	hypothetical protein
wy_04728	-	5032224	5032889	+	666	CAAX amino terminal protease self- immunity

wy_04729	-	5032896	5033342	-	447	Putative pyridoxine/pyridoxamine 5'-phosphate oxidase
wy_04730	-	5033398	5033784	+	387	hypothetical protein
wy_04731	*purF*	5033893	5035473	+	1581	Amidophosphoribosyltransferase precursor
wy_04732	*purM*	5035617	5036696	+	1080	Phosphoribosylformylglycinamidine cyclo-ligase
wy_04733	-	5036894	5037109	-	216	hypothetical protein
wy_04734	-	5037253	5038248	-	996	L-asparaginase II
wy_04735	-	5038245	5038895	-	651	6-N-hydroxylaminopurine resistance protein
wy_04736	*gcvT_2*	5038892	5040025	-	1134	Aminomethyltransferase
wy_04737	*pabC*	5040051	5040974	+	924	Aminodeoxychorismate lyase
wy_04738	*cydA*	5041131	5042648	+	1518	Cytochrome bd ubiquinol oxidase subunit 1
wy_04739	*cydB*	5042658	5043680	+	1023	Cytochrome bd-I ubiquinol oxidase subunit 2
wy_04740	*cydD*	5043677	5045416	+	1740	ATP-binding/permease protein CydD
wy_04741	-	5045406	5047133	+	1728	putative ABC transporter ATP-binding protein
wy_04742	-	5047414	5048130	+	717	hypothetical protein
wy_04743	-	5048487	5049185	-	699	hypothetical protein
wy_04744	-	5049450	5050676	+	1227	Cutinase
wy_04745	-	5050738	5051043	-	306	hypothetical protein
wy_04746	-	5051049	5051882	-	834	Putative thiosulfate sulfurtransferase
wy_04747	-	5051984	5052457	-	474	hypothetical protein
wy_04748	*trxA_3*	5052710	5053183	-	474	Thioredoxin-1
wy_04749	-	5053180	5054043	-	864	hypothetical protein
wy_04750	*phoP*	5054107	5054868	+	762	Alkaline phosphatase synthesis transcriptional regulatory protein PhoP
wy_04751	*mshD_2*	5054869	5055777	+	909	Mycothiol acetyltransferase
wy_04752	*pstS3*	5055983	5057110	+	1128	Phosphate-binding protein PstS 3 precursor
wy_04753	*pstC*	5057169	5058218	+	1050	Phosphate transport system permease protein PstC
wy_04754	*pstA*	5058222	5059127	+	906	Phosphate transport system permease protein PstA

wy_04755	*pstB3*	5059222	5059998	+	777	Phosphate import ATP-binding protein PstB 3
wy_04756	-	5060081	5061211	-	1131	NDMA-dependent alcohol dehydrogenase
wy_04757	-	5061490	5062476	-	987	Endonuclease/Exonuclease/phosphatase family protein
wy_04758	-	5062550	5063239	-	690	hypothetical protein
wy_04759	*msrR_2*	5063322	5065310	-	1989	Regulatory protein MsrR
wy_04760	-	5065440	5066381	-	942	hypothetical protein
wy_04761	*dusC*	5066459	5067601	-	1143	tRNA-dihydrouridine synthase C
wy_04762	*desA1*	5067699	5068652	-	954	Putative acyl-[acyl-carrier-protein] desaturase desA1
wy_04763	*gltC_5*	5068858	5069775	-	918	HTH-type transcriptional regulator GltC
wy_04764	*ynfM_2*	5069871	5071106	+	1236	Inner membrane transport protein YnfM
wy_04765	-	5071181	5071573	+	393	hypothetical protein
wy_04766	-	5071570	5072151	+	582	hypothetical protein
wy_04767	-	5072256	5073011	+	756	hypothetical protein
wy_04769	-	5074286	5074675	-	390	hypothetical protein
wy_04770	-	5074780	5075592	+	813	DNA-damage-inducible protein D
wy_04771	-	5075589	5075729	+	141	hypothetical protein
wy_04772	-	5075726	5076247	+	522	hypothetical protein
wy_04773	-	5076321	5076596	+	276	hypothetical protein
wy_04774	-	5076593	5076841	+	249	hypothetical protein
wy_04775	-	5076838	5076996	+	159	hypothetical protein
wy_04776	-	5076989	5077984	+	996	hypothetical protein
wy_04777	*sftA*	5077981	5079117	+	1137	DNA translocase SftA
wy_04778	-	5079167	5079781	+	615	hypothetical protein
wy_04779	*ssb_3*	5079794	5080279	+	486	Single-stranded DNA-binding protein
wy_04780	-	5080297	5080818	+	522	hypothetical protein
wy_04781	-	5080815	5081078	+	264	hypothetical protein

wy_04782	-	5081075	5081386	+	312	hypothetical protein
wy_04783	-	5081383	5082147	+	765	hypothetical protein
wy_04784	-	5082144	5082488	+	345	hypothetical protein
wy_04785	-	5082485	5082685	+	201	hypothetical protein
wy_04786	-	5082682	5083128	+	447	hypothetical protein
wy_04787	-	5083122	5083613	+	492	hypothetical protein
wy_04788	-	5083610	5083840	+	231	hypothetical protein
wy_04789	-	5083837	5084013	+	177	hypothetical protein
wy_04790	-	5084010	5084678	+	669	hypothetical protein
wy_04791	-	5084675	5085115	+	441	hypothetical protein
wy_04792	-	5085576	5085767	+	192	hypothetical protein
wy_04793	-	5085793	5086158	+	366	hypothetical protein
wy_04794	-	5086299	5086655	+	357	hypothetical protein
wy_04795	-	5086627	5088147	+	1521	Terminase-like family protein
wy_04796	-	5088141	5089406	+	1266	hypothetical protein
wy_04797	*clpP*	5089403	5090548	+	1146	ATP-dependent Clp protease proteolytic subunit
wy_04798	-	5090551	5090961	+	411	hypothetical protein
wy_04799	-	5090975	5091898	+	924	hypothetical protein
wy_04800	-	5091895	5092332	+	438	hypothetical protein
wy_04801	-	5092352	5092714	+	363	hypothetical protein
wy_04802	-	5092707	5093039	+	333	hypothetical protein
wy_04803	-	5093032	5093361	+	330	hypothetical protein
wy_04804	-	5093363	5093776	+	414	hypothetical protein
wy_04805	-	5093844	5094530	+	687	hypothetical protein
wy_04806	-	5094636	5094971	+	336	hypothetical protein
wy_04807	-	5094968	5095351	+	384	hypothetical protein

wy_04808	-	5095354	5096163	+	810	hypothetical protein
wy_04809	-	5096174	5101459	+	5286	hypothetical protein
wy_04810	-	5101456	5102286	+	831	hypothetical protein
wy_04811	-	5102290	5103864	+	1575	hypothetical protein
wy_04812	-	5103878	5104267	+	390	hypothetical protein
wy_04813	-	5104264	5105388	+	1125	LGFP repeat protein
wy_04814	-	5105465	5106070	+	606	hypothetical protein
wy_04815	-	5106074	5106553	+	480	hypothetical protein
wy_04816	-	5106594	5107271	+	678	hypothetical protein
wy_04817	-	5107264	5108946	+	1683	hypothetical protein
wy_04818	-	5108946	5109830	+	885	hypothetical protein
wy_04819	-	5109903	5110157	-	255	hypothetical protein
wy_04820	*yjbM*	5110154	5110693	-	540	GTP pyrophosphokinase YjbM
wy_04821	-	5111179	5111457	-	279	hypothetical protein
wy_04822	-	5111454	5111660	-	207	hypothetical protein
wy_04823	-	5111657	5111839	-	183	hypothetical protein
wy_04824	-	5111937	5112077	-	141	hypothetical protein
wy_04825	-	5112749	5113237	+	489	hypothetical protein
wy_04826	-	5113638	5113757	+	120	hypothetical protein
wy_04827	-	5113770	5113904	+	135	hypothetical protein
wy_04828	-	5114083	5115009	+	927	hypothetical protein
wy_04829	*xerD_3*	5115103	5115564	-	462	Tyrosine recombinase XerD
wy_04830	-	5115641	5115943	-	303	hypothetical protein
wy_04831	-	5116033	5116215	-	183	hypothetical protein
wy_04833	-	5116959	5117255	+	297	hypothetical protein
wy_04834	-	5117188	5117541	-	354	hypothetical protein

wy_04835	-	5117646	5118458	+	813	DNA-damage-inducible protein D
wy_04836	-	5118455	5118595	+	141	hypothetical protein
wy_04837	-	5118592	5119113	+	522	hypothetical protein
wy_04838	-	5119187	5119462	+	276	hypothetical protein
wy_04839	-	5119459	5119707	+	249	hypothetical protein
wy_04840	-	5119704	5119862	+	159	hypothetical protein
wy_04841	-	5120058	5120846	+	789	hypothetical protein
wy_04842	-	5121163	5121714	+	552	hypothetical protein
wy_04843	*ftsK*	5121771	5121977	+	207	DNA translocase FtsK
wy_04844	-	5122027	5122428	+	402	hypothetical protein
wy_04845	-	5122741	5123787	-	1047	hypothetical protein
wy_04846	-	5124067	5124273	+	207	hypothetical protein
wy_04847	-	5124273	5124812	+	540	hypothetical protein
wy_04848	-	5124994	5125179	-	186	hypothetical protein
wy_04849	-	5125229	5125483	-	255	hypothetical protein
wy_04850	-	5126504	5126782	-	279	hypothetical protein
wy_04851	-	5126779	5126985	-	207	hypothetical protein
wy_04852	-	5126982	5127164	-	183	hypothetical protein
wy_04853	-	5127264	5127404	-	141	hypothetical protein
wy_04854	-	5128076	5128564	+	489	hypothetical protein
wy_04855	-	5128965	5129084	+	120	hypothetical protein
wy_04856	-	5129097	5129231	+	135	hypothetical protein
wy_04857	-	5129410	5130336	+	927	hypothetical protein
wy_04858	*xerD_4*	5130430	5131272	-	843	Tyrosine recombinase XerD
wy_04859	-	5131362	5131544	-	183	hypothetical protein

**Supplementary Table S4. Enrichment analysis of virulence, resistance and TCS genes in core genome of *R. equi***						
**Gene**	**In core genome**	**In pan genome**	**Core genes**	**Pan genes**	**P-value (In core genome vs In pan genome)**	**Rate (In core genome/In pan genome)**
virulence gene	295	376	4057	6172	9.62752E-09	0.78
resistance gene	399	489	4057	6172	2.14226E-16	0.82
TCS gene	198	229	4057	6172	1.14272E-13	0.86
*P-value here reflects the degree of enrichment of virulence/resistance/TCS genes in core genome compared to these in pan genome.						

Supplementary Table S5. Pathway enrichment analysis of the virulent genes						
Pathway	In core genome	In pan genome	Core genes	Pan genes	P-value	FDR
Biosynthesis of amino acids [PATH:ko01230]	14	14	155	200	0	0
Two-component system [PATH:ko02020]	13	13	155	200	0	0
Nitrogen metabolism [PATH:ko00910]	8	8	155	200	0	0
Glyoxylate and dicarboxylate metabolism [PATH:ko00630]	6	6	155	200	0	0
Protein export [PATH:ko03060]	5	5	155	200	0	0
Biosynthesis of siderophore group nonribosomal peptides [PATH:ko01053]	5	5	155	200	0	0
Propanoate metabolism [PATH:ko00640]	5	5	155	200	0	0
Bacterial secretion system [PATH:ko03070]	5	5	155	200	0	0
Amyotrophic lateral sclerosis (ALS) [PATH:ko05014]	4	4	155	200	0	0
Phenylalanine, tyrosine and tryptophan biosynthesis [PATH:ko00400]	4	4	155	200	0	0
Cell cycle - Caulobacter [PATH:ko04112]	3	3	155	200	0	0
Pyrimidine metabolism [PATH:ko00240]	3	3	155	200	0	0
RNA degradation [PATH:ko03018]	3	3	155	200	0	0
Fructose and mannose metabolism [PATH:ko00051]	3	3	155	200	0	0
Carbon fixation pathways in prokaryotes [PATH:ko00720]	3	3	155	200	0	0
2-Oxocarboxylic acid metabolism [PATH:ko01210]	3	3	155	200	0	0
Pentose phosphate pathway [PATH:ko00030]	3	3	155	200	0	0
C5-Branched dibasic acid metabolism [PATH:ko00660]	3	3	155	200	0	0
Pantothenate and CoA biosynthesis [PATH:ko00770]	3	3	155	200	0	0
Tuberculosis [PATH:ko05152]	3	3	155	200	0	0
MAPK signaling pathway - yeast [PATH:ko04011]	3	3	155	200	0	0
PPAR signaling pathway [PATH:ko03320]	2	2	155	200	0	0
Monobactam biosynthesis [PATH:ko00261]	2	2	155	200	0	0
Vitamin B6 metabolism [PATH:ko00750]	2	2	155	200	0	0

Terpenoid backbone biosynthesis [PATH:ko00900]	2	2	155	200	0	0
Steroid hormone biosynthesis [PATH:ko00140]	2	2	155	200	0	0
Ubiquinone and other terpenoid-quinone biosynthesis [PATH:ko00130]	2	2	155	200	0	0
Carbon fixation in photosynthetic organisms [PATH:ko00710]	2	2	155	200	0	0
Degradation of aromatic compounds [PATH:ko01220]	2	2	155	200	0	0
Atrazine degradation [PATH:ko00791]	2	2	155	200	0	0
Citrate cycle (TCA cycle) [PATH:ko00020]	2	2	155	200	0	0
GABAergic synapse [PATH:ko04727]	2	2	155	200	0	0
Glutamatergic synapse [PATH:ko04724]	2	2	155	200	0	0
Adipocytokine signaling pathway [PATH:ko04920]	2	2	155	200	0	0
Inositol phosphate metabolism [PATH:ko00562]	2	2	155	200	0	0
Legionellosis [PATH:ko05134]	2	2	155	200	0	0
Type I diabetes mellitus [PATH:ko04940]	2	2	155	200	0	0
Vibrio cholerae pathogenic cycle [PATH:ko05111]	1	1	155	200	0	0
Prostate cancer [PATH:ko05215]	1	1	155	200	0	0
Ribosome [PATH:ko03010]	1	1	155	200	0	0
Valine, leucine and isoleucine biosynthesis [PATH:ko00290]	1	1	155	200	0	0
Methane metabolism [PATH:ko00680]	1	1	155	200	0	0
Thiamine metabolism [PATH:ko00730]	1	1	155	200	0	0
Phenylalanine metabolism [PATH:ko00360]	1	1	155	200	0	0
NOD-like receptor signaling pathway [PATH:ko04621]	1	1	155	200	0	0
Progesterone-mediated oocyte maturation [PATH:ko04914]	1	1	155	200	0	0
Apoptosis - fly [PATH:ko04214]	1	1	155	200	0	0
Sulfur relay system [PATH:ko04122]	1	1	155	200	0	0
Dioxin degradation [PATH:ko00621]	1	1	155	200	0	0
Plant-pathogen interaction [PATH:ko04626]	1	1	155	200	0	0
Protein processing in endoplasmic reticulum [PATH:ko04141]	1	1	155	200	0	0
Antigen processing and presentation [PATH:ko04612]	1	1	155	200	0	0

PI3K-Akt signaling pathway [PATH:ko04151]	1	1	155	200	0	0
Salmonella infection [PATH:ko05132]	1	1	155	200	0	0
Type II diabetes mellitus [PATH:ko04930]	1	1	155	200	0	0
African trypanosomiasis [PATH:ko05143]	1	1	155	200	0	0
Xylene degradation [PATH:ko00622]	1	1	155	200	0	0
Prion diseases [PATH:ko05020]	1	1	155	200	0	0
Taurine and hypotaurine metabolism [PATH:ko00430]	1	1	155	200	0	0
Aminobenzoate degradation [PATH:ko00627]	1	1	155	200	0	0
D-Alanine metabolism [PATH:ko00473]	1	1	155	200	0	0
Pathways in cancer [PATH:ko05200]	1	1	155	200	0	0
Glucagon signaling pathway [PATH:ko04922]	1	1	155	200	0	0
Viral carcinogenesis [PATH:ko05203]	1	1	155	200	0	0
Styrene degradation [PATH:ko00643]	1	1	155	200	0	0
Central carbon metabolism in cancer [PATH:ko05230]	1	1	155	200	0	0
Epithelial cell signaling in Helicobacter pylori infection [PATH:ko05120]	1	1	155	200	0	0
Cationic antimicrobial peptide (CAMP) resistance [PATH:ko01503]	1	1	155	200	0	0
Chagas disease (American trypanosomiasis) [PATH:ko05142]	1	1	155	200	0	0
Carbapenem biosynthesis [PATH:ko00332]	1	1	155	200	0	0
Benzoate degradation [PATH:ko00362]	1	1	155	200	0	0
Selenocompound metabolism [PATH:ko00450]	1	1	155	200	0	0
Vancomycin resistance [PATH:ko01502]	1	1	155	200	0	0
Estrogen signaling pathway [PATH:ko04915]	1	1	155	200	0	0
Aminoacyl-tRNA biosynthesis [PATH:ko00970]	1	1	155	200	0	0
Carbon metabolism [PATH:ko01200]	11	12	155	200	0.0424708	0.0676179
Longevity regulating pathway - worm [PATH:ko04212]	9	10	155	200	0.0730424	0.1147809
Pyruvate metabolism [PATH:ko00620]	7	8	155	200	0.1248071	0.1887708
Tryptophan metabolism [PATH:ko00380]	7	8	155	200	0.1248071	0.1887708
Peroxisome [PATH:ko04146]	7	8	155	200	0.1248071	0.1887708

Alanine, aspartate and glutamate metabolism [PATH:ko00250]	11	13	155	200	0.1644613	0.2369025
beta-Alanine metabolism [PATH:ko00410]	6	7	155	200	0.1627553	0.2369025
Arginine and proline metabolism [PATH:ko00330]	6	7	155	200	0.1627553	0.2369025
Longevity regulating pathway - multiple species [PATH:ko04213]	6	7	155	200	0.1627553	0.2369025
Glycerolipid metabolism [PATH:ko00561]	5	6	155	200	0.2119095	0.2981518
Purine metabolism [PATH:ko00230]	5	6	155	200	0.2119095	0.2981518
Valine, leucine and isoleucine degradation [PATH:ko00280]	4	5	155	200	0.2754824	0.3663007
Fatty acid degradation [PATH:ko00071]	4	5	155	200	0.2754824	0.3663007
Glycine, serine and threonine metabolism [PATH:ko00260]	4	5	155	200	0.2754824	0.3663007
Longevity regulating pathway - mammal [PATH:ko04211]	4	5	155	200	0.2754824	0.3663007
FoxO signaling pathway [PATH:ko04068]	4	5	155	200	0.2754824	0.3663007
Sulfur metabolism [PATH:ko00920]	3	4	155	200	0.3575798	0.4652382
Galactose metabolism [PATH:ko00052]	3	4	155	200	0.3575798	0.4652382
Arginine biosynthesis [PATH:ko00220]	6	8	155	200	0.4283921	0.5514408
Huntington's disease [PATH:ko05016]	2	3	155	200	0.4634422	0.5781083
Chloroalkane and chloroalkene degradation [PATH:ko00625]	2	3	155	200	0.4634422	0.5781083
Histidine metabolism [PATH:ko00340]	2	3	155	200	0.4634422	0.5781083
Glycolysis / Gluconeogenesis [PATH:ko00010]	5	7	155	200	0.5068351	0.6257863
ABC transporters [PATH:ko02010]	32	42	155	200	0.5172404	0.6321827
Butanoate metabolism [PATH:ko00650]	4	6	155	200	0.5933467	0.7108411
Limonene and pinene degradation [PATH:ko00903]	4	6	155	200	0.5933467	0.7108411
MAPK signaling pathway - fly [PATH:ko04013]	1	2	155	200	0.5997487	0.7114666
Tyrosine metabolism [PATH:ko00350]	3	5	155	200	0.6859694	0.798099
Nicotinate and nicotinamide metabolism [PATH:ko00760]	3	5	155	200	0.6859694	0.798099
Lysine degradation [PATH:ko00310]	5	8	155	200	0.7421644	0.8552561
D-Glutamine and D-glutamate metabolism [PATH:ko00471]	0	1	155	200	0.775	0.868287
Renin-angiotensin system [PATH:ko04614]	0	1	155	200	0.775	0.868287
Butirosin and neomycin biosynthesis [PATH:ko00524]	0	1	155	200	0.775	0.868287

Pentose and glucuronate interconversions [PATH:ko00040]	4	7	155	200	0.8096255	0.8987585
Biosynthesis of vancomycin group antibiotics [PATH:ko01055]	1	3	155	200	0.8723618	0.959598
Amino sugar and nucleotide sugar metabolism [PATH:ko00520]	5	9	155	200	0.8827083	0.9622315
Ascorbate and aldarate metabolism [PATH:ko00053]	2	5	155	200	0.9236197	0.9978392
Fatty acid biosynthesis [PATH:ko00061]	7	19	155	200	0.9999322	0.999988
Fatty acid metabolism [PATH:ko01212]	7	19	155	200	0.9999322	0.999988
Biotin metabolism [PATH:ko00780]	6	18	155	200	0.9999701	0.999988
Biosynthesis of unsaturated fatty acids [PATH:ko01040]	5	17	155	200	0.999988	0.999988
Polyketide sugar unit biosynthesis [PATH:ko00523]	4	10	155	200	0.9902574	0.999988
Streptomycin biosynthesis [PATH:ko00521]	4	11	155	200	0.9969264	0.999988
Acarbose and validamycin biosynthesis [PATH:ko00525]	2	6	155	200	0.9760247	0.999988
Starch and sucrose metabolism [PATH:ko00500]	1	4	155	200	0.9636941	0.999988
Lipopolysaccharide biosynthesis [PATH:ko00540]	0	2	155	200	0.9502513	0.999988

*Column 2 denotes the number of virulent genes involved in each pathway in core genome;Column 3 denotes the number of virulent genes involved in each pathway in pan genome;Column 4 denotes the number of virulent genes involved in all metabolic pathways in core genome;Column 5 denotes the number of virulent genes involved in all metabolic pathways in pan genome.

Supplementary Table S6. The COG function classification and functional annotation of the animal-unique genes																
Orthologs from all the animal-associated species							GI number	COG	Function	Classification	Virulence	Resistance	TCS	Transporter	Annotation
WY	C 7	103S	ATCC 33707	N1288	N1295	NBRC 101255									
wy_00077	APJC01000001_00088	FN563149_00077	CM001149_00078	LRQY01000000_00096	LRQZ01000000_00089	NBRC101255_00089	331699473	[S] COG1652 Nucleoid-associated protein YgaU, contains BON and LysM domains	[S]Function unknown	POORLY CHARACTERIZED	-	-	-	-	Resuscitation-promoting factor RpfA precursor
wy_00093	APJC01000001_00103	FN563149_00093	CM001149_00094	LRQY01000000_00112	LRQZ01000000_00104	NBRC101255_00104	15828151	[G] COG2814 Predicted arabinose efflux permease, MFS family	[G]Carbohydrate transport and metabolism	METABOLISM	-	1 PF07690.11 Major Facilitator Superfamily [ARO:0010002]	-	MFS-Rv0876c1560801 6 MFS hypothetical protein Rv0876c [Mycobacterium tuberculosis H37Rv]	Major Facilitator Superfamily protein

wy_00094	APJC01000001_00104	FN563149_00094	CM001149_00095	LRQY01000000_00113	LRQZ01000000_00105	NBRC101255_00105	111021934	[O] COG3823 Glutamine cyclotransferase	[O]Posttranslational modification, protein turnover, chaperones	CELLULAR PROCESSES AND SIGNALING	-	-	-	-	Glutamine cyclotransferase
wy_00106	APJC01000001_00116	FN563149_00106	CM001149_00106	LRQY01000000_00125	LRQZ01000000_00117	NBRC101255_00119	-	-	-	-	-	-	-	-	hypothetical protein
wy_00108	APJC01000001_00118	FN563149_00108	CM001149_00108	LRQY01000000_00127	LRQZ01000000_00119	NBRC101255_00121	111021959	[HE] COG1932 Phosphoserine aminotransferase	[E]Amino acid transport and metabolism<br>[H]Coenzyme transport and metabolism	METABOLISM<br>METABOLISM	-	-	-	-	Phosphoserine aminotransferase

wy_00109	APJC01000001_00119	FN563149_00109	CM001149_00109	LRQY01000000_00128	LRQZ01000000_00120	NBRC101255_00122	161353731	[C] COG0372 Citrate synthase	[C]Energy production and conversion	METABOLISM	-	-	-	-	Citrate synthase 1
wy_00188	APJC01000001_00197	FN563149_00187	CM001149_00214	LRQY01000000_00206	LRQZ01000000_00230	NBRC101255_00200	111020772	[CR] COG0604 NADPH:quinone reductase or related Zn-dependent oxidoreductase	[C]Energy production and conversion<br>[R]General function prediction only	METABOLISM<br>POORLY CHARACTERIZED	-	-	-	-	Quinone oxidoreductase 1
wy_00216	APJC01000001_00226	FN563149_00214	CM001149_00242	LRQY01000000_00235	LRQZ01000000_00259	NBRC101255_00229	111017270	[DM] COG0768 Cell division protein FtsI/penicillin-binding protein 2	[D]Cell cycle control, cell division, chro	CELLULAR PROCESSES AND SIGNALING	-	1 PF00905.17 Penicillin binding protein transpeptidase domain	-	-	Stage V sporulation protein D

									mosome partitioning			[ARO:3000129]			
									[M] Cell wall/membrane/envelope biogenesis	CELLULAR PROCESSES AND SIGNALING					
wy_00253	APJC01000001_00264	FN563149_00251	CM001149_00278	LRQY01000000_00272	LRQZ01000000_00297	NBRC101255_00267	111022537	[M] COG0739 Murein DD-endopeptidase MepM and murein hydrolase activator NlpD, contain LysM domain	[M] Cell wall/membrane/envelope biogenesis	CELLULAR PROCESSES AND SIGNALING	-	-	-	-	Murein DD-endopeptidase MepM

wy_00331	APJC01000001_00342	FN563149_00330	CM001149_00357	LRQY01000000_00352	LRQZ01000000_00374	NBRC101255_00345	271961627	[P] COG2217 Cation transport ATPase	[P]Inorganic ion transport and metabolism	METABOLISM	-	-	-	P-ATPase-SCO10462 1219562 P-ATPase putative metal transporter ATPase [Streptomyces coelicolor A3(2)]	Copper-exporting P-type ATPase A
wy_00348	APJC01000001_00359	FN563149_00347	CM001149_00374	LRQY01000000_00369	LRQZ01000000_00392	NBRC101255_00362	357389824	[Q] COG0656 Aldo/keto reductase, related to diketogulonate reductase	[Q]Secondary metabolites biosynthesis, transport and catabolis	METABOLISM	-	-	-	-	putative oxidoreductase/MSMEI_2347

									m						
wy_00349	APJC01000001_00360	FN563149_00348	CM001149_00375	LRQY01000000_00370	LRQZ01000000_00393	NBRC101255_00363	220911721	[G] COG2814 Predicted arabinose efflux permease, MFS family	[G]Carbohydrate transport and metabolism	METABOLISM	-	1 chloramphenicol efflux pump [ARO:3001212]	-	MFS-SCO3915212 22322 MFS putative transmembrane efflux protein [Streptomyces coelicolor A3(2)]	Inner membrane transport protein YdhP
wy_00359	APJC01000001_00370	FN563149_00358	CM001149_00385	LRQY01000000_00380	LRQZ01000000_00404	NBRC101255_00373	384148385	[P] COG3301 Formate-dependent nitrite reductase, membrane component NrfD	[P]Inorganic ion transport and metabolism	METABOLISM	-	-	-	-	Polysulfide reductase, NrfD
wy_00368	APJC01000001_00379	FN563149_00367	CM001149_00393	LRQY01000000_00389	LRQZ01000000_00412	NBRC101255_00383	111022665	[H] COG3572 Gamma-glutamylcy	[H]Coenzyme trans	METABOLISM	-	-	-	-	Glutamate--cysteine ligase

								steine synthetase	port and meta bolis m						GshA
wy_ 003 73	APJC0 100000 1_0038 4	FN563 149_00 372	CM0 0114 9_00 398	LRQY0 100000 0_0039 4	LRQZ0 100000 0_0041 7	NBRC1 01255_ 00388	-	-	-	-	-	-	-	-	hypothe tical protein
wy_ 003 83	APJC0 100000 1_0039 4	FN563 149_00 382	CM0 0114 9_00 408	LRQY0 100000 0_0040 4	LRQZ0 100000 0_0042 7	NBRC1 01255_ 00398	11102 2738	[P] COG2822 Iron uptake system EfeUOB, periplasmic (or lipoprotein) component EfeO/EfeM	[P]In orga nic ion trans port and meta bolis m	META BOLIS M	-	-	-	-	Iron uptake system compon ent EfeO precurs or
wy_ 003 86	APJC0 100000 1_0039 7	FN563 149_00 385	CM0 0114 9_00 411	LRQY0 100000 0_0040 7	LRQZ0 100000 0_0043 0	NBRC1 01255_ 00401	11102 2741	[G] COG0148 Enolase	[G]C arbo hydr ate trans port and meta bolis m	META BOLIS M	-	-	-	-	Enolase
wy_ 004 04	APJC0 100000 1_0041 5	FN563 149_00 403	CM0 0114 9_00 430	LRQY0 100000 0_0042 8	LRQZ0 100000 0_0053 0	NBRC1 01255_ 00419	11102 2806	[E] COG0031 Cysteine synthase	[E]A mino acid trans port and meta bolis	META BOLIS M	-	-	-	-	Putativ e cystathi onine beta- synthas e

									m						
wy_00494	APJC01000001_00510	FN563149_00490	CM001149_00518	LRQY01000000_00517	LRQZ01000000_00626	NBRC101255_00514	386743483	[EH] COG0028 Acetolactate synthase large subunit or other thiamine pyrophosphate-requiring enzyme	[E]Amino acid transport and metabolism<br>[H]Coenzyme transport and metabolism	METABOLISM<br>METABOLISM	-	-	-	-	putative oxalyl-CoA decarboxylase
wy_00551	APJC01000001_00568	FN563149_00548	CM001149_00575	LRQY01000000_00574	LRQZ01000000_00683	NBRC101255_00572	-	-	-	-	-	-	-	-	hypothetical protein

wy_00594	APJC01000001_00611	FN563149_00591	CM001149_00618	LRQY01000000_00617	LRQZ01000000_00726	NBRC101255_00615	111022976	[V] COG1132 ABC-type multidrug transport system, ATPase and permease component	[V] Defense mechanisms	CELLULAR PROCESSES AND SIGNALING	VFG012509(gi:26247127) (iroC) ATP binding cassette transporter [Salmochelin (IA013)] [Escherichia coli CFT073]	2 msbA: ATP-binding cassette (ABC) antibiotic efflux pump [ARO:3000460]	-	ABC-CE119125027747 ABC(membrane) putative multidrug resistance protein [Corynebacterium efficiens YS-314]	Putative multidrug export ATP-binding/permease protein
wy_00604	APJC01000001_00621	FN563149_00601	CM001149_00628	LRQY01000000_00627	LRQZ01000000_00737	NBRC101255_00625	111017160	[TK] COG2197 DNA-binding response regulator, NarL/FixJ family, contains	[K]Transcription	INFORMATION STORAGE AND PROCESSING	-	-	-	-	Putative HTH-type transcriptional regulator/MT0914

								REC and HTH domains	[T]Signal transduction mechanisms	CELLULAR PROCESSES AND SIGNALING					
wy_00620	APJC01000001_00637	FN563149_00617	CM001149_00644	LRQY01000000_00643	LRQZ01000000_00754	NBRC101255_00641	111022995	[L] COG1112 Superfamily I DNA and/or RNA helicase	[L]Replication, recombination and repair	INFORMATION STORAGE AND PROCESSING	-	-	-	-	RecBCD enzyme subunit RecD
wy_00661	APJC01000001_00672	FN563149_00652	CM001149_00680	LRQY01000000_00678	LRQZ01000000_00789	NBRC101255_00676	111021891	[E] COG0119 Isopropylmalate/homocitrate/citramalate synthases	[E]Amino acid transport and metabolism	METABOLISM	-	-	-	-	Hydroxymethylglutaryl-CoA lyase YngG
wy_00677	APJC01000001_00688	FN563149_00668	CM001149_00696	LRQY01000000_00693	LRQZ01000000_00805	NBRC101255_00692	269128905	[E] COG0747 ABC-type transport system, periplasmic component	[E]Amino acid transport and metabolism	METABOLISM	-	-	-	-	Periplasmic dipeptide transport protein precursor

wy_00694	APJC01000001_00705	FN563149_00685	CM001149_00713	LRQY01000000_00710	LRQZ01000000_00822	NBRC101255_00709	297565041	[L] COG1525 Endonuclease YncB, thermonuclease family	[L]Replication, recombination and repair	INFORMATION STORAGE AND PROCESSING	-	-	-	-	Excalibur calcium-binding domain protein
wy_00711	APJC01000001_00721	FN563149_00701	CM001149_00729	LRQY01000000_00726	LRQZ01000000_00838	NBRC101255_00725	269127707	[C] COG1053 Succinate dehydrogenase/fumarate reductase, flavoprotein subunit	[C]Energy production and conversion	METABOLISM	-	-	-	-	3-oxosteroid 1-dehydrogenase
wy_00774	APJC01000001_00781	FN563149_00757	CM001149_00782	LRQY01000000_00789	LRQZ01000000_00898	NBRC101255_00786	111018835	[L] COG1330 Exonuclease V gamma subunit	[L]Replication, recombination and repair	INFORMATION STORAGE AND PROCESSING	-	-	-	-	RecBCD enzyme subunit RecC
wy_00780	APJC01000001_00785	FN563149_00763	CM001149_00790	LRQY01000000_00798	LRQZ01000000_00902	NBRC101255_00790	332669158	[S] COG2968 Uncharacterized conserved protein YggE, contains	[S]Function unknown	POORLY CHARACTERIZED	-	-	-	-	hypothetical protein

								kinase-interacting SIMPL domain							
wy_00798	APJC01000001_00806	FN563149_00796	CM001149_00834	LRQY01000000_00827	LRQZ01000000_00923	NBRC101255_00811	407646704	[D] COG3266 Cell division protein DamX, binds to the septal ring, contains C-terminal SPOR domain	[D]Cell cycle control, cell division, chromosome partitioning	CELLULAR PROCESSES AND SIGNALING	-	-	-	-	PPE family protein
wy_00872	APJC01000001_00882	FN563149_00861	CM001149_00901	LRQY01000000_00892	LRQZ01000000_00999	NBRC101255_00887	111018491	[J] COG0018 Arginyl-tRNA synthetase	[J]Translation, ribosomal structure and biogenesis	INFORMATION STORAGE AND PROCESSING	-	-	KSE_49980_PP_HisKa|Kitasatospora	-	Arginine--tRNA ligase
wy_00873	APJC01000001_00883	FN563149_00862	CM001149_00902	LRQY01000000_00893	LRQZ01000000_01000	NBRC101255_00888	111018490	[E] COG0019 Diaminopimelate decarboxylase	[E]Amino acid transport and metabolis	METABOLISM	-	-	-	-	Diaminopimelate decarboxylase

									m						
wy_00904	APJC01000001_00911	FN563149_00893	CM001149_00933	LRQY01000000_00922	LRQZ01000000_01029	NBRC101255_00916	-	-	-	-	-	-	-	-	hypothetical protein
wy_00934	APJC01000001_00941	FN563149_00922	CM001149_00962	LRQY01000000_00951	LRQZ01000000_01059	NBRC101255_00946	111023499	[K] COG2183 Transcriptional accessory protein Tex/SPT6	[K]Transcription	INFORMATION STORAGE AND PROCESSING	-	-	-	-	hypothetical protein
wy_00950	APJC01000001_00958	FN563149_00939	CM001149_00978	LRQY01000000_00968	LRQZ01000000_01076	NBRC101255_00963	111023536	[M] COG0739 Murein DD-endopeptidase MepM and murein hydrolase activator NlpD, contain LysM domain	[M] Cell wall/membrane/envelope biogenesis	CELLULAR PROCESSES AND SIGNALING	-	-	-	-	Peptidase family M23
wy_00966	APJC01000001_00974	FN563149_00955	CM001149_00994	LRQY01000000_00984	LRQZ01000000_01092	NBRC101255_00979	384149649	[NU] COG1989 Prepilin signal peptidase PulO (type II secretory pathway)	[N]Cell motility	CELLULAR PROCESSES AND SIGNALING	10869|vfid|17574|vsiid|27577|ssi	-	-	-	Type 4 prepilin-like proteins leader peptide-process

								or related peptidase	[U]Intracellular trafficking, secretion, and vesicular transport	CELLULAR PROCESSES AND SIGNALING	d|RecName: Full=Type 4 prepilin-like proteins leader peptide-processing enzyme; Includes: RecName: Full=Leader peptidase; EC=3.4.23.43; AltNam				ing enzyme

											e: Full =Prepilin peptidase; RecName: Full=N-methyltransferase; EC=2.1.1.-;				
wy_00972	APJC01000001_00980	FN563149_00961	CM001149_01000	LRQY01000000_00991	LRQZ01000000_01098	NBRC101255_00985	284991840	[UW] COG4962 Pilus assembly protein, ATPase of CpaF family	[U]Intracellular trafficking, secretion, and vesicular transport	CELLULAR PROCESSES AND SIGNALING	3367|vfid|4340|vsiid|4904|ssid|pilus assembly, secretory protein kina	-	-	-	Putative conjugal transfer protein/MT3759
									[W] Extracellular	CELLULAR PROCESSES		-	-	-	

									structures	AND SIGNALING	se				
wy_00973	APJC01000001_00981	FN563149_00962	CM001149_01001	LRQY01000000_00992	LRQZ01000000_01099	NBRC101255_00986	219883049	[UW] COG4965 Flp pilus assembly protein TadB	[U]Intracellular trafficking, secretion, and vesicular transport	CELLULAR PROCESSES AND SIGNALING	-	-	-	-	Bacterial type II secretion system protein F domain protein
									[W] Extracellular structures	CELLULAR PROCESSES AND SIGNALING					
wy_01010	APJC01000001_01018	FN563149_00999	CM001149_01038	LRQY01000000_01028	LRQZ01000000_01136	NBRC101255_01023	257054855	[GER] COG0697 Permease of the drug/metabolite transporter (DMT) superfamily	[E]Amino acid transport and metabolism	METABOLISM	-	-	-	-	hypothetical protein
									[G]Carbohydr	METABOLISM					

									ate transport and metabolism						
									[R]General function prediction only	POORLY CHARACTERIZED					
wy_01093	APJC01000001_01102	FN563149_01082	CM001149_01122	LRQY01000000_01111	LRQZ01000000_01220	NBRC101255_01106	111023719	[K] COG1309 DNA-binding transcriptional regulator, AcrR family	[K]Transcription	INFORMATION STORAGE AND PROCESSING	-	-	-	-	Bacterial regulatory proteins, tetR family
wy_01148	APJC01000001_01157	FN563149_01137	CM001149_01177	LRQY01000000_01166	LRQZ01000000_01276	NBRC101255_01161	111023776	[KL] COG1061 Superfamily II DNA or RNA helicase	[K]Transcription	INFORMATION STORAGE AND PROCESSING	-	-	-	-	Type III restriction enzyme, res subunit
									[L]Replication, reco	INFORMATION STORAGE					

									mbination and repair	AND PROCESSING					
wy_01150	APJC01000001_01159	FN563149_01139	CM001149_01179	LRQY01000000_01168	LRQZ01000000_01278	NBRC101255_01163	111023778	[S] COG3502 Uncharacterized conserved protein, DUF952 family	[S]Function unknown	POORLY CHARACTERIZED	-	-	-	-	hypothetical protein
wy_01181	APJC01000001_01190	FN563149_01170	CM001149_01210	LRQY01000000_01199	LRQZ01000000_01309	NBRC101255_01194	384147681	[HR] COG0596 Pimeloyl-ACP methyl ester carboxylesterase	[H]Coenzyme transport and metabolism<br>[R]General function prediction only	METABOLISM<br>POORLY CHARACTERIZED	-	-	-	-	short chain dehydrogenase
wy_01182	APJC01000001_01191	FN563149_01171	CM001149_01211	LRQY01000000_01200	LRQZ01000000_01310	NBRC101255_01195	384147679	[Q] COG1020 Non-ribosomal peptide synthetase component F	[Q]Secondary metabolites biosynth	METABOLISM	-	-	-	-	Putative diacylglycerol O-acyltransferase/MT180

									esis, transport and catabolism						9
wy_01183	APJC01000001_01192	FN563149_01172	CM001149_01212	LRQY01000000_01201	LRQZ01000000_01311	NBRC101255_01196	111023814	[I] COG0657 Acetyl esterase/lipase	[I]Lipid transport and metabolism	METABOLISM	-	-	-	-	Carboxylesterase NlhH
wy_01184	APJC01000001_01193	FN563149_01173	CM001149_01213	LRQY01000000_01202	LRQZ01000000_01312	NBRC101255_01197	-	-	-	-	-	-	-	-	hypothetical protein
wy_01226	APJC01000001_01237	FN563149_01215	CM001149_01257	LRQY01000000_01243	LRQZ01000000_00183	NBRC101255_01241	111023852	[TK] COG0317 (p)ppGpp synthase/hydrolase, HD superfamily	[K]Transcription<br>[T]Signal transduction mechanisms	INFORMATION STORAGE AND PROCESSING<br>CELLULAR PROCESSES AND SIGNALING	2506|vfid|2750|vsiid|3107|ssid|GTP pyrophosphokinase [Mycobacterium tube	-	-	-	Bifunctional (p)ppGpp synthase/hydrolase relA

											rculosis H37Rv]				
wy_01267	APJC01000001_01277	FN563149_01265	CM001149_01297	LRQY01000000_01283	LRQZ01000000_01365	NBRC101255_01281	404216650	[Q] COG2175 Taurine dioxygenase, alpha-ketoglutarate-dependent	[Q]Secondary metabolites biosynthesis, transport and catabolism	METABOLISM	-	-	-	-	putative dioxygenase/MT3514
wy_01423	APJC01000001_01432	FN563149_01420	CM001149_01452	LRQY01000000_01440	LRQZ01000000_01521	NBRC101255_01436	310823954	[M] COG1388 LysM repeat	[M] Cell wall/membrane/envelope biogenesis	CELLULAR PROCESSES AND SIGNALING	-	-	-	-	Chitinase class I
wy_01447	APJC01000001_01456	FN563149_01444	CM001149_01476	LRQY01000000_01464	LRQZ01000000_01545	NBRC101255_01460	111017863	[O] COG0638 20S proteasome, alpha and beta subunits	[O]Posttranslational modification,	CELLULAR PROCESSES AND SIGNALING	-	-	-	-	Proteasome subunit beta 1 precursor

									protein turnover, chaperones						
wy_01473	APJC01000001_01482	FN563149_01470	CM001149_01502	LRQY01000000_01490	LRQZ01000000_01571	NBRC101255_01486	256372750	[NW] COG3170 Tfp pilus assembly protein FimV	[N]Cell motility<br>[W] Extracellular structures	CELLULAR PROCESSES AND SIGNALING<br>CELLULAR PROCESSES AND SIGNALING	-	-	-	-	hypothetical protein
wy_01515	APJC01000001_01525	FN563149_01513	CM001149_01544	LRQY01000000_01532	LRQZ01000000_01613	NBRC101255_01528	111017663	[R] COG2764 Uncharacterized conserved protein PhnB, glyoxalase superfamily	[R]General function prediction only	POORLY CHARACTERIZED	-	-	-	-	Glyoxalase-like domain protein
wy_01561	APJC01000001_01575	FN563149_01560	CM001149_01588	LRQY01000000_01577	LRQZ01000000_01662	NBRC101255_01577	111017924	[T] COG1716 Forkhead associated (FHA)	[T]Signal transduction	CELLULAR PROCESSES AND	-	-	-	-	Glycogen accumulation regulat

								domain, binds pSer, pThr, pTyr	mechanisms	SIGNALING					or GarA
wy_01626	APJC01000001_01639	FN563149_01626	CM001149_01715	LRQY01000000_01640	LRQZ01000000_01726	NBRC101255_01640	333919163	[T] COG0589 Nucleotide-binding universal stress protein, UspA family	[T]Signal transduction mechanisms	CELLULAR PROCESSES AND SIGNALING	-	1 PF00582.21_Universal stress protein family	-	-	Universal stress protein/MT1672
wy_01688	APJC01000001_01676	FN563149_01664	CM001149_01752	LRQY01000000_01677	LRQZ01000000_01762	NBRC101255_01677	436835776	[K] COG2207 AraC-type DNA-binding domain and AraC-containing proteins	[K]Transcription	INFORMATION STORAGE AND PROCESSING	-	2 "PF00165.18 Bacterial regulatory helix-turn-helix proteins, AraC family" [ARO:3000000]	-	-	DNA-binding transcriptional regulator AraC
wy_01694	APJC01000001_01680	FN563149_01669	CM001149_01757	LRQY01000000_01682	LRQZ01000000_01767	NBRC101255_01681	333918499	[T] COG2200 EAL domain, c-di-GMP-specific phosphodiesterase class I (or its	[T]Signal transduction mechanisms	CELLULAR PROCESSES AND SIGNALING	-	-	-	-	EAL domain protein

								enzymatically inactive variant)						
wy_01705, wy_03195	APJC01000001_03281,APJC01000001_01690	FN563149_01679,FN563149_03181	CM001149_01767	LRQY01000000_03281	LRQZ01000000_01777,LRQZ01000000_03327	NBRC101255_01691, NBRC101255_03269	384149469	[M] COG1004 UDP-glucose 6-dehydrogenase	[M] Cell wall/membrane/envelope biogenesis	CELLULAR PROCESSES AND SIGNALING	15206|vfid|24404|vsiid|45224|ssid|SubName: Full=UDP-glucose 6-dehydrogenase 2; EC=1.1.1.22;	-	-	-	UDP-glucose 6-dehydrogenase TuaD
wy_01720	APJC01000001_01713	FN563149_01703	CM001149_01789	LRQY01000000_01717	LRQZ01000000_01799	NBRC101255_01714	-	-	-	-	-	-	-	-	hypothetical protein
wy_01723	APJC01000001_01717	FN563149_01706	CM001149_01792	LRQY01000000_01721	LRQZ01000000_01803	NBRC101255_01718	111024032	[HR] COG0596 Pimeloyl-ACP methyl ester	[H]Coenzyme transport and	METABOLISM	-	-	-	-	haloalkane dehalogenase

								carboxylesterase	metabolism					
									[R]General function prediction only	POORLY CHARACTERIZED				
wy_01724	APJC01000001_01718	FN563149_01707	CM001149_01793	LRQY01000000_01722	LRQZ01000000_01804	NBRC101255_01719	-	-	-	-	-	-	-	-	hypothetical protein
wy_01725	APJC01000001_01719	FN563149_01708	CM001149_01794	LRQY01000000_01723	LRQZ01000000_01805	NBRC101255_01720	-	-	-	-	-	-	-	-	hypothetical protein
wy_01726	APJC01000001_01720	FN563149_01709	CM001149_01795	LRQY01000000_01724	LRQZ01000000_01806	NBRC101255_01721	111017727	[S] COG5637 Uncharacterized membrane protein	[S]Function unknown	POORLY CHARACTERIZED	-	-	-	-	Polyketide cyclase / dehydrase and lipid transport
wy_01728	APJC01000001_01722	FN563149_01711	CM001149_01797	LRQY01000000_01726	LRQZ01000000_01808	NBRC101255_01723	428775140	[J] COG0154 Asp-tRNAAsn/Glu-tRNAGln amidotransferase A subunit or related	[J]Translation, ribosomal structure and biogenesi	INFORMATION STORAGE AND PROCESSING	-	-	-	-	Gas vesicle synthesis protein GvpL/GvpF

								amidase	s						
wy_01729	APJC01000001_01723	FN563149_01712	CM001149_01798	LRQY01000000_01727	LRQZ01000000_01809	NBRC101255_01724	-	-	-	-	-	-	-	-	Gas vesicle protein G
wy_01731	APJC01000001_01725	FN563149_01714	CM001149_01800	LRQY01000000_01729	LRQZ01000000_01811	NBRC101255_01726	-	-	-	-	-	-	-	-	Gas vesicle structural protein
wy_01732	APJC01000001_01726	FN563149_01715	CM001149_01801	LRQY01000000_01730	LRQZ01000000_01812	NBRC101255_01727	-	-	-	-	-	-	-	-	Gas vesicle synthesis protein GvpL/GvpF
wy_01733	APJC01000001_01727	FN563149_01716	CM001149_01802	LRQY01000000_01731	LRQZ01000000_01813	NBRC101255_01728	-	-	-	-	-	-	-	-	Gas vesicle protein
wy_01734	APJC01000001_01728	FN563149_01717	CM001149_01803	LRQY01000000_01732	LRQZ01000000_01814	NBRC101255_01729	-	-	-	-	-	-	-	-	Gas vesicle protein K
wy_01735	APJC01000001_01732	FN563149_01718	CM001149_01806	LRQY01000000_01733	LRQZ01000000_01818	NBRC101255_01732	257055184	[G] COG0297 Glycogen synthase	[G]Carbohydrate transport and metabolism	METABOLISM	-	-	-	-	D-inositol 3-phosphate glycosyltransferase

wy_01736	APJC01000001_01733	FN563149_01720	CM001149_01807	LRQY01000000_01734	LRQZ01000000_01819	NBRC101255_01733	108805896	[M] COG0451 Nucleoside-diphosphate-sugar epimerase	[M] Cell wall/membrane/envelope biogenesis	CELLULAR PROCESSES AND SIGNALING	-	-	-	-	dTDP-glucose 4,6-dehydratase 2
wy_01738	APJC01000001_01737	FN563149_01724	CM001149_01811	LRQY01000000_01736	LRQZ01000000_01823	NBRC101255_01737	256396808	[T] COG2172 Anti-sigma regulatory factor (Ser/Thr protein kinase)	[T]Signal transduction mechanisms	CELLULAR PROCESSES AND SIGNALING	-	-	-	-	Anti-sigma-F factor RsbW
wy_01751	APJC01000001_01749	FN563149_01736	CM001149_01825	LRQY01000000_01748	LRQZ01000000_01835	NBRC101255_01749	256372750	[NW] COG3170 Tfp pilus assembly protein FimV	[N]Cell motility<br>[W] Extracellular structures	CELLULAR PROCESSES AND SIGNALING<br>CELLULAR PROCESSES AND SIGNALING	-	-	-	-	hypothetical protein
wy_01761	APJC01000001_0176	FN563149_01749	CM001149_01	LRQY01000000_0176	LRQZ01000000_0185	NBRC101255_01764	111017264	[IQR] COG1028 NAD(P)-	[I]Lipid trans	METABOLISM	-	-	-	-	putative oxidoreductase

	4		834	1	0			dependent dehydrogenase, short-chain alcohol dehydrogenase family	port and metabolism						YghA
									[Q]Secondary metabolites biosynthesis, transport and catabolism	METABOLISM					
									[R]General function prediction only	POORLY CHARACTERIZED					
wy_01771	APJC01000001_01773	FN563149_01758	CM001149_01843	LRQY01000000_01770	LRQZ01000000_01859	NBRC101255_01773	108763328	[ER] COG1063 Threonine dehydrogenase or related Zn-dependent dehydrogenase	[E]Amino acid transport and metabolism	METABOLISM	-	-	-	-	Glutathione-independent formaldehyde dehydrogenase

									[R]General function prediction only	POORLY CHARACTERIZED					
wy_01779	APJC01000001_01781	FN563149_01766	CM001149_01851	LRQY01000000_01779	LRQZ01000000_01867	NBRC101255_01781	374983637	[L] COG0122 3-methyladenine DNA glycosylase/8-oxoguanine DNA glycosylase	[L]Replication, recombination and repair	INFORMATION STORAGE AND PROCESSING	-	-	-	-	Bifunctional transcriptional activator/DNA repair enzyme AdaA
wy_01832	APJC01000001_01835	FN563149_01820	CM001149_01902	LRQY01000000_01834	LRQZ01000000_01922	NBRC101255_01835	-	-	-	-	-	-	-	-	hypothetical protein
wy_01891	APJC01000001_01894	FN563149_01876	CM001149_01957	LRQY01000000_01892	LRQZ01000000_01988	NBRC101255_01893	-	-	-	-	-	-	-	-	hypothetical protein
wy_01892	APJC01000001_01895	FN563149_01877	CM001149_01958	LRQY01000000_01893	LRQZ01000000_01989	NBRC101255_01894	111018085	[J] COG0564 Pseudouridylate synthase, 23S rRNA- or tRNA-specific	[J]Translation, ribosomal structure and biogenesis	INFORMATION STORAGE AND PROCESSING	-	-	-	-	Ribosomal large subunit pseudouridine synthase D

wy_01924	APJC01000001_01927	FN563149_01909	CM001149_01990	LRQY01000000_01926	LRQZ01000000_02021	NBRC101255_01926	111018117	[N] COG1215 Glycosyltransferase, catalytic subunit of cellulose synthase and poly-beta-1,6-N-acetylglucosamine synthase	[N]Cell motility	CELLULAR PROCESSES AND SIGNALING	-	-	-	-	4,4'-diaponeurosporenoate glycosyltransferase
wy_02017	APJC01000001_02022	FN563149_02001	CM001149_02083	LRQY01000000_02018	LRQZ01000000_02118	NBRC101255_02020	-	-	-	-	-	-	-	-	hypothetical protein
wy_02081	APJC01000001_02086	FN563149_02065	CM001149_02148	LRQY01000000_02095	LRQZ01000000_02182	NBRC101255_02085	111018283	[P] COG1613 ABC-type sulfate transport system, periplasmic component	[P]Inorganic ion transport and metabolism	METABOLISM	-	-	-	ABC-Mb2422c31793578 ABC(binding protein) PROBABLE SULFATE-BINDING LIPOPROTEIN SUBI	Sulfate-binding protein precursor

														[Mycobacterium bovis AF2122/97]	
wy_02099	APJC01000001_02104	FN563149_02083	CM001149_02166	LRQY01000000_02113	LRQZ01000000_02201	NBRC101255_02104	111018308	[S] COG3552 Uncharacterized conserved protein, contains von Willebrand factor type A (vWA) domain	[S]Function unknown	POORLY CHARACTERIZED	-	-	-	-	VWA domain containing CoxE-like protein
wy_02121	APJC01000001_02126	FN563149_02105	CM001149_02188	LRQY01000000_02135	LRQZ01000000_02223	NBRC101255_02126	-	-	-	-	-	-	-	-	hypothetical protein
wy_02122	APJC01000001_0212	FN563149_02106	CM001149_02	LRQY01000000_0213	LRQZ01000000_0222	NBRC101255_02127	111018339	[U] COG0823 Periplasmi	[U]Intracellul	CELLULAR PROC	-	-	-	-	Prolyl tripeptidyl

	7		189	6	4			c component of the Tol biopolymer transport system	ar trafficking, secretion, and vesicular transport	ESSES AND SIGNALING					peptidase precursor
wy_02126	APJC01000001_02131	FN563149_02110	CM001149_02193	LRQY01000000_02140	LRQZ01000000_02229	NBRC101255_02131	111018342	[C] COG1526 Formate dehydrogenase assembly factor FdhD	[C]Energy production and conversion	METABOLISM	-	-	-	-	formate dehydrogenase accessory protein
wy_02263	APJC01000001_02273	FN563149_02246	CM001149_02330	LRQY01000000_02279	LRQZ01000000_02370	NBRC101255_02274	111023463	[H] COG1920 2-phospho-L-lactate guanylyltransferase, coenzyme F420 biosynthesis enzyme, CobY/MobA/RfbA family	[H]Coenzyme transport and metabolism	METABOLISM	-	-	-	-	2-phospho-L-lactate guanylyltransferase
wy_02312	APJC01000001_02323	FN563149_02295	CM001149_02379	LRQY01000000_02327	LRQZ01000000_02420	NBRC101255_02323	403510945	[E] COG0686 Alanine dehydroge	[E]Amino acid trans	METABOLISM	-	-	-	-	Alanine dehydrogenase

								nase	port and metabolism						
wy_02335	APJC01000001_02346	FN563149_02319	CM001149_02402	LRQY01000000_02350	LRQZ01000000_02443	NBRC101255_02346	111020474	[R] COG0702 Uncharacterized conserved protein YbjT, contains NAD(P)-binding and DUF2867 domains	[R]General function prediction only	POORLY CHARACTERIZED	-	-	-	-	NmrA-like family protein
wy_02345	APJC01000001_02356	FN563149_02329	CM001149_02412	LRQY01000000_02360	LRQZ01000000_02452	NBRC101255_02355	111020485	[J] COG0154 Asp-tRNAAsn/Glu-tRNAGln amidotransferase A subunit or related amidase	[J]Translation, ribosomal structure and biogenesis	INFORMATION STORAGE AND PROCESSING	-	-	Tmar_0491_PP_HisKa|Thermaerobacter	-	Glutamyl-tRNA(Gln) amidotransferase subunit A
wy_02405	APJC01000001_02474	FN563149_02388	CM001149_02473	LRQY01000000_02486	LRQZ01000000_02511	NBRC101255_02472	407648093	[O] COG0695 Glutaredoxin	[O]Posttranslational modification, prote	CELLULAR PROCESSES AND SIGNALING	-	-	-	-	Putative glutaredoxin.1/MT3292

									in turnover, chaperones						
wy_02406	APJC01000001_02475	FN563149_02389	CM001149_02474	LRQY01000000_02487	LRQZ01000000_02512	NBRC101255_02473	111023342	[F] COG2816 NADH pyrophosphatase NudC, Nudix superfamily	[F]Nucleotide transport and metabolism	METABOLISM	-	-	-	-	NADH pyrophosphatase
wy_02541	APJC01000001_02609	FN563149_02516	CM001149_02609	LRQY01000000_02624	LRQZ01000000_02647	NBRC101255_02608	111023238	[C] COG1012 Acyl-CoA reductase or other NAD-dependent aldehyde dehydrogenase	[C]Energy production and conversion	METABOLISM	-	-	-	-	Succinate-semialdehyde dehydrogenase [NADP(+)]
wy_02547	APJC01000001_02615	FN563149_02522	CM001149_02615	LRQY01000000_02630	LRQZ01000000_02653	NBRC101255_02614	111023234	[L] COG1201 Lhr-like helicase	[L]Replication, recombination and repair	INFORMATION STORAGE AND PROCESSING	-	-	-	-	putative ATP-dependent helicase Lhr
wy_02551	APJC01000001_0261	FN563149_02526	CM001149_02	LRQY01000000_0263	LRQZ01000000_0265	NBRC101255_02618	111023228	[C] COG0578 Glycerol-3-	[C]Energy	METABOLISM	-	-	-	-	Aerobic glycerol-3-

	9		619	4	7			phosphate dehydrogenase	production and conversion						phosphate dehydrogenase
wy_02602	APJC01000001_02670	FN563149_02589	CM001149_02670	LRQY01000000_02685	LRQZ01000000_02708	NBRC101255_02669	111021894	[K] COG2207 AraC-type DNA-binding domain and AraC-containing proteins	[K]Transcription	INFORMATION STORAGE AND PROCESSING	-	2 "PF00165.18 Bacterial regulatory helix-turn-helix proteins, AraC family" [ARO:3000000]	-	-	transcriptional regulator EutR
wy_02608	APJC01000001_02677	FN563149_02595	CM001149_02677	LRQY01000000_02691	LRQZ01000000_02715	NBRC101255_02676	111023182	[S] COG4430 Uncharacterized conserved protein YdeI, YjbR/CyaY-like superfamily, DUF1801 family	[S]Function unknown	POORLY CHARACTERIZED	-	-	-	-	hypothetical protein

wy_02612	APJC01000001_02681	FN563149_02599	CM001149_02681	LRQY01000000_02695	LRQZ01000000_02719	NBRC101255_02680	111023176	[L] COG0389 Nucleotidyltransferase/DNA polymerase involved in DNA repair	[L]Replication, recombination and repair	INFORMATION STORAGE AND PROCESSING	-	-	-	-	DNA polymerase IV
wy_02613	APJC01000001_02682	FN563149_02600	CM001149_02682	LRQY01000000_02696	LRQZ01000000_02720	NBRC101255_02681	323359222	[S] COG4544 Uncharacterized conserved protein	[S]Function unknown	POORLY CHARACTERIZED	-	-	-	-	hypothetical protein
wy_02625	APJC01000001_02694	FN563149_02612	CM001149_02694	LRQY01000000_02708	LRQZ01000000_02732	NBRC101255_02693	111023159	[K] COG1595 DNA-directed RNA polymerase specialized sigma subunit, sigma24 family	[K]Transcription	INFORMATION STORAGE AND PROCESSING	-	-	-	-	ECF RNA polymerase sigma factor SigD
wy_02634	APJC01000001_02703	FN563149_02621	CM001149_02703	LRQY01000000_02717	LRQZ01000000_02741	NBRC101255_02702	269127881	[CR] COG0604 NADPH:quinone reductase or related Zn-dependent oxidoreductase	[C]Energy production and conversion	METABOLISM	-	-	-	-	Crotonyl-CoA reductase

									[R]General function prediction only	POORLY CHARACTERIZED					
wy_02644	APJC01000001_02713	FN563149_02631	CM001149_02713	LRQY01000000_02727	LRQZ01000000_02752	NBRC101255_02712	111018178	[R] COG3021 Uncharacterized conserved protein YafD, endonuclease/exonuclease/phosphatase (EEP) superfamily	[R]General function prediction only	POORLY CHARACTERIZED	-	-	-	-	Endonuclease/Exonuclease/phosphatase family protein
wy_02653	APJC01000001_02722	FN563149_02641	CM001149_02722	LRQY01000000_02736	LRQZ01000000_02761	NBRC101255_02721	111023149	[O] COG0443 Molecular chaperone DnaK (HSP70)	[O]Posttranslational modification, protein turnover, chaperones	CELLULAR PROCESSES AND SIGNALING	-	-	-	-	Hsp70 protein

wy_02667	APJC01000001_02735	FN563149_02654	CM001149_02735	LRQY01000000_02749	LRQZ01000000_02774	NBRC101255_02734	-	-	-	-	-	-	-	-	hypothetical protein
wy_02703	APJC01000001_02770	FN563149_02689	CM001149_02770	LRQY01000000_02784	LRQZ01000000_02810	NBRC101255_02769	111023109	[J] COG0199 Ribosomal protein S14	[J]Translation, ribosomal structure and biogenesis	INFORMATION STORAGE AND PROCESSING	-	-	-	-	30S ribosomal protein S14 type Z
wy_02727	APJC01000001_02794	FN563149_02713	CM001149_02794	LRQY01000000_02815	LRQZ01000000_02843	NBRC101255_02792	403511169	[P] COG0748 Putative heme iron utilization protein	[P]Inorganic ion transport and metabolism	METABOLISM	-	-	-	-	Pyridoxamine 5'-phosphate oxidase
wy_02753	APJC01000001_02820	FN563149_02739	CM001149_02820	LRQY01000000_02841	LRQZ01000000_02870	NBRC101255_02818	111023039	[I] COG0446 NADPH-dependent 2,4-dienoyl-CoA reductase, sulfur reductase, or a related oxidoreductase	[I]Lipid transport and metabolism	METABOLISM	-	-	-	-	putative N-methyl proline demethylase

wy_02880	APJC01000001_02947	FN563149_02867	CM001149_02948	LRQY01000000_02968	LRQZ01000000_02997	NBRC101255_02945	111022670	[E] COG1878 Kynurenine formamidase	[E]Amino acid transport and metabolism	METABOLISM	-	-	-	-	Kynurenine formamidase
wy_02881	APJC01000001_02948	FN563149_02868	CM001149_02949	LRQY01000000_02969	LRQZ01000000_02998	NBRC101255_02946	111022669	[G] COG2133 Glucose/arabinose dehydrogenase, beta-propeller fold	[G]Carbohydrate transport and metabolism	METABOLISM	-	-	-	-	Soluble aldose sugar dehydrogenase YliI precursor
wy_02884	APJC01000001_02951	FN563149_02871	CM001149_02952	LRQY01000000_02972	LRQZ01000000_03001	NBRC101255_02949	336119956	[P] COG2217 Cation transport ATPase	[P]Inorganic ion transport and metabolism	METABOLISM	-	-	-	P-ATPase-SAV533129831874 P-ATPase putative cation-transporting P-type ATPa	Copper-exporting P-type ATPase A

														se [Streptomyces avermitilis MA-4680]	
wy_03046	APJC01000001_03117	FN563149_03029	CM001149_03124	LRQY01000000_03129	LRQZ01000000_03168	NBRC101255_03115	-	-	-	-	-	-	-	-	hypothetical protein
wy_03048	APJC01000001_03119	FN563149_03032	CM001149_03127	LRQY01000000_03132	LRQZ01000000_03171	NBRC101255_03118	62391375	[V] COG3467 Nitroimidazol reductase NimA or a related FMN-containing flavoprotein, pyridoxamine 5'-phosphate oxidase superfamily	[V] Defense mechanisms	CELLULAR PROCESSES AND SIGNALING	-	-	-	-	Pyridoxamine 5'-phosphate oxidase

wy_03049	APJC01000001_03120	FN563149_03033	CM001149_03128	LRQY01000000_03133	LRQZ01000000_03172	NBRC101255_03119	333918209	[V] COG0494 8-oxo-dGTP pyrophosphatase MutT and related house-cleaning NTP pyrophosphohydrolases, NUDIX family	[V] Defense mechanisms	CELLULAR PROCESSES AND SIGNALING	-	-	-	-	hypothetical protein
wy_03085	APJC01000001_03158	FN563149_03070	CM001149_03165	LRQY01000000_03170	LRQZ01000000_03210	NBRC101255_03157	111019809	[C] COG2218 Formylmethanofuran dehydrogenase subunit C	[C]Energy production and conversion	METABOLISM	-	-	-	-	GXGXG motif protein
wy_03119	APJC01000001_03193	FN563149_03104	CM001149_03199	LRQY01000000_03204	LRQZ01000000_03244	NBRC101255_03191	111022485	[G] COG2814 Predicted arabinose efflux permease, MFS family	[G]Carbohydrate transport and metabolism	METABOLISM	-	2 PF07690.11 Major Facilitator Superfamily [ARO:0010002]	-	MFS-YfmO 01607780 7 MFS similar to multi drug-efflux transporter	Multidrug efflux protein YfmO

														[Bacillus subtilis 168]	
wy_03122	APJC01000001_03196	FN563149_03107	CM001149_03202	LRQY01000000_03208	LRQZ01000000_03247	NBRC101255_03194	229821441	[G] COG0153 Galactokinase	[G]Carbohydrate transport and metabolism	METABOLISM	-	-	-	-	Galactokinase
wy_03123	APJC01000001_03197	FN563149_03108	CM001149_03203	LRQY01000000_03209	LRQZ01000000_03248	NBRC101255_03195	111022457	[G] COG1085 Galactose-1-phosphate uridylyltransferase	[G]Carbohydrate transport and metabolism	METABOLISM	-	-	-	-	Galactose-1-phosphate uridylyltransferase
wy_03124	APJC01000001_03198	FN563149_03109	CM001149_03204	LRQY01000000_03210	LRQZ01000000_03249	NBRC101255_03196	-	-	-	-	-	-	-	-	hypothetical protein

wy_03126	APJC01000001_03200	FN563149_03111	CM001149_03206	LRQY01000000_03211	LRQZ01000000_03251	NBRC101255_03198	327404659	[S] COG1704 Uncharacterized conserved protein	[S]Function unknown	POORLY CHARACTERIZED	-	-	-	-	LemA family protein
wy_03127	APJC01000001_03201	FN563149_03112	CM001149_03207	LRQY01000000_03212	LRQZ01000000_03252	NBRC101255_03199	-	-	-	-	-	-	-	-	hypothetical protein
wy_03128	APJC01000001_03202	FN563149_03113	CM001149_03208	LRQY01000000_03213	LRQZ01000000_03253	NBRC101255_03200	-	-	-	-	-	-	-	-	hypothetical protein
wy_03130	APJC01000001_03204	FN563149_03115	CM001149_03210	LRQY01000000_03215	LRQZ01000000_03255	NBRC101255_03202	111019483	[P] COG2072 Predicted flavoprotein CzcO associated with the cation diffusion facilitator CzcD	[P]Inorganic ion transport and metabolism	METABOLISM	-	-	-	-	Phenylacetone monooxygenase
wy_03131	APJC01000001_03205	FN563149_03116	CM001149_03211	LRQY01000000_03216	LRQZ01000000_03256	NBRC101255_03203	111018272	[S] COG5649 Uncharacterized protein	[S]Function unknown	POORLY CHARACTERIZED	-	-	-	-	hypothetical protein
wy_03132	APJC01000001_03206	FN563149_03117	CM001149_03212	LRQY01000000_03217	LRQZ01000000_03257	NBRC101255_03204	-	-	-	-	-	-	-	-	hypothetical protein
wy_03171	APJC01000001_03244	FN563149_03155	CM001149_03250	LRQY01000000_03255	LRQZ01000000_03297	NBRC101255_03242	111022453	[L] COG0699 Replication fork	[L]Replication,	INFORMATION STOR	-	-	-	-	Isoniazid-induced protein

								clamp-binding protein CrfC (dynamin-like GTPase family)	recombination and repair	AGE AND PROCESSING					IniA
wy_03173	APJC01000001_03246	FN563149_03157	CM001149_03252	LRQY01000000_03257	LRQZ01000000_03299	NBRC101255_03244	111022451	[O] COG0443 Molecular chaperone DnaK (HSP70)	[O]Posttranslational modification, protein turnover, chaperones	CELLULAR PROCESSES AND SIGNALING	-	-	-	-	Chaperone protein DnaK
wy_03188	APJC01000001_03264	FN563149_03172	CM001149_03269	LRQY01000000_03274	LRQZ01000000_03318	NBRC101255_03260	111022418	[Q] COG1020 Non-ribosomal peptide synthetase component F	[Q]Secondary metabolites biosynthesis, transport and catabolism	METABOLISM	-	-	-	-	Tyrocidine synthase 3

wy_03218	APJC01000001_03300	FN563149_03202	CM001149_03299	LRQY01000000_03304	LRQZ01000000_03346	NBRC101255_03288	111022322	[Q] COG1020 Non-ribosomal peptide synthetase component F	[Q]Secondary metabolites biosynthesis, transport and catabolism	METABOLISM	-	-	-	-	Putative diacylglycerol O-acyltransferase/MT0919
wy_03231	APJC01000001_03313	FN563149_03215	CM001149_03314	LRQY01000000_03317	LRQZ01000000_03359	NBRC101255_03301	111022166	[IQR] COG1028 NAD(P)-dependent dehydrogenase, short-chain alcohol dehydrogenase family	[I]Lipid transport and metabolism<br>[Q]Secondary metabolites biosynthesis, transport and catabolism	METABOLISM<br>METABOLISM	-	-	-	-	3-oxoacyl-[acyl-carrier-protein] reductase FabG

									[R]General function prediction only	POORLY CHARACTERIZED					
wy_03263	APJC01000001_03357	FN563149_03247	CM001149_03358	LRQY01000000_03361	LRQZ01000000_03402	NBRC101255_03344	111022142	[S] COG1432 Uncharacterized conserved protein, LabA/DUF88 family	[S]Function unknown	POORLY CHARACTERIZED	-	-	-	-	hypothetical protein
wy_03276	APJC01000001_03370	FN563149_03260	CM001149_03375	LRQY01000000_03374	LRQZ01000000_03415	NBRC101255_03361	111022125	[E] COG3104 Dipeptide/tripeptide permease	[E]Amino acid transport and metabolism	METABOLISM	-	2 PF07690.11 Major Facilitator Superfamily [ARO:0010002]	-	POT-CE290325029459 POT putative tripeptide transporter [Corynebacterium efficiens YS-314]	Di-/tripeptide transporter

wy_03280	APJC01000001_03374	FN563149_03264	CM001149_03379	LRQY01000000_03378	LRQZ01000000_03419	NBRC101255_03365	111019328	[I] COG1960 Acyl-CoA dehydrogenase related to the alkylation response protein AidB	[I]Lipid transport and metabolism	METABOLISM	-	-	-	-	(R)-benzylsuccinyl-CoA dehydrogenase
wy_03284	APJC01000001_03378	FN563149_03268	CM001149_03383	LRQY01000000_03382	LRQZ01000000_03423	NBRC101255_03369	111022122	[DM] COG0768 Cell division protein FtsI/penicillin-binding protein 2	[D]Cell cycle control, cell division, chromosome partitioning<br>[M] Cell wall/membrane/envelope biogenesis	CELLULAR PROCESSES AND SIGNALING<br>CELLULAR PROCESSES AND SIGNALING	-	1 PF00905.17 Penicillin binding protein transpeptidase domain [ARO:3000129]	-	-	Penicillin-binding protein A

wy_03315	APJC01000001_03412	FN563149_03302	CM001149_03419	LRQY01000000_03413	LRQZ01000000_03457	NBRC101255_03403	284992719	[G] COG2301 Citrate lyase beta subunit	[G]Carbohydrate transport and metabolism	METABOLISM	-	-	-	-	(3S)-malyl-CoA thioesterase
wy_03316	APJC01000001_03413	FN563149_03303	CM001149_03420	LRQY01000000_03414	LRQZ01000000_03458	NBRC101255_03404	111021616	[E] COG2902 NAD-specific glutamate dehydrogenase	[E]Amino acid transport and metabolism	METABOLISM	-	-	NH8B_3110_RR_unclassified|Pseudogulbenkiania	-	NAD-specific glutamate dehydrogenase
wy_03376	APJC01000001_03472	FN563149_03352	CM001149_03469	LRQY01000000_03464	LRQZ01000000_03517	NBRC101255_03462	111022086	[Q] COG3485 Protocatechuate 3,4-dioxygenase beta subunit	[Q]Secondary metabolites biosynthesis, transport and catabolism	METABOLISM	-	-	-	-	Dioxygenase
wy_03393	APJC01000001_0348	FN563149_03369	CM001149_03	LRQY01000000_0348	LRQZ01000000_0353	NBRC101255_03479	284990602	[ER] COG1063 Threonine	[E]Amino acid	METABOLISM	-	-	-	-	NADP-dependent

	9		486	1	4			dehydrogenase or related Zn-dependent dehydrogenase	transport and metabolism<br>[R]General function prediction only	 POORLY CHARACTERIZED					isopropanol dehydrogenase
wy_03477	APJC01000001_03572	FN563149_03451	CM001149_03569	LRQY01000000_03564	LRQZ01000000_03617	NBRC101255_03562	529065158	[PR] COG4638 Phenylpropionate dioxygenase or related ring-hydroxylating dioxygenase, large terminal subunit	[P]Inorganic ion transport and metabolism<br>[R]General function prediction only	METABOLISM<br>POORLY CHARACTERIZED	-	-	-	-	Carbazole 1,9a-dioxygenase, terminal oxygenase component CarAa
wy_03542	APJC01000001_03637	FN563149_03516	CM001149_03635	LRQY01000000_03628	LRQZ01000000_03681	NBRC101255_03626	111019332	[IQR] COG1028 NAD(P)-dependent dehydrogenase, short-	[I]Lipid transport and meta	METABOLISM	VFG005767(gi:225368	-	-	-	Short-chain reductase protein NovJ

								chain alcohol dehydrogenase family	bolism		31) (cylG) 3-ketoacyl-ACP-reductase CylG [Beta-hemolysin/cytolysin (CVF171)] [Streptococcus agalactiae 2603V/R]				
									[Q]Secondary metabolites biosynthesis, transport and catabolism	METABOLISM					
									[R]General function prediction only	POORLY CHARACTERIZED					
wy_03559	APJC01000001_03654	FN563149_03533	CM001149_03652	LRQY01000000_03645	LRQZ01000000_03698	NBRC101255_03643	111020880	[I] COG1788 Acyl CoA:acetat	[I]Lipid transport	METABOLISM	-	-	-	-	putative succinyl-CoA:3-

								e/3-ketoacid CoA transferase, alpha subunit	and metabolism					ketoacid coenzyme A transferase subunit A
wy_03647	APJC01000001_03750	FN563149_03626	CM001149_03744	LRQY01000000_03740	LRQZ01000000_03794	NBRC101255_03739	296139021	[M] COG1316 Anionic cell wall polymer biosynthesis enzyme, LytR-Cps2A-Psr (LCP) family	[M] Cell wall/membrane/envelope biogenesis	CELLULAR PROCESSES AND SIGNALING	-	-	-	-	Transcriptional regulator LytR
wy_03670	APJC01000001_03773	FN563149_03649	CM001149_03767	LRQY01000000_03764	LRQZ01000000_03817	NBRC101255_03762	333920301	[E] COG0747 ABC-type transport system, periplasmic component	[E]Amino acid transport and metabolism	METABOLISM	-	-	-	ABC-SCO6451212224757 ABC(binding protein) putative substrate binding protein [Strep	Oligopeptide-binding protein AppA precursor

														tomyces coelicolor A3(2)]	
wy_03682	APJC01000001_03785	FN563149_03661	CM001149_03779	LRQY01000000_03776	LRQZ01000000_03829	NBRC101255_03774	433609550	[M] COG0739 Murein DD-endopeptidase MepM and murein hydrolase activator NlpD, contain LysM domain	[M] Cell wall/membrane/envelope biogenesis	CELLULAR PROCESSES AND SIGNALING	-	-	-	-	Murein DD-endopeptidase MepM
wy_03683	APJC01000001_03786	FN563149_03662	CM001149_03780	LRQY01000000_03777	LRQZ01000000_03830	NBRC101255_03775	111019298	[IQ] COG0318 Acyl-CoA synthetase (AMP-forming)/AMP-acid ligase II	[I]Lipid transport and metabolism	METABOLISM	7571|vfid|9028|vsiid|10946|ssid|2	-	-	-	Long-chain-fatty-acid--CoA ligase

									[Q]Secondary metabolites biosynthesis, transport and catabolism	METABOLISM	,3-dihydroxybenzoate-AMP ligase [Bacillus anthracis str. Ames]				
wy_03705	APJC01000001_03810	FN563149_03685	CM001149_03806	LRQY01000000_03801	LRQZ01000000_03854	NBRC101255_03799	-	-	-	-	-	-	-	-	hypothetical protein
wy_03754	APJC01000001_03858	FN563149_03760	CM001149_03854	LRQY01000000_03848	LRQZ01000000_03902	NBRC101255_03847	-	-	-	-	-	-	-	-	hypothetical protein
wy_03767	APJC01000001_03885	FN563149_03773	CM001149_03867	LRQY01000000_03861	LRQZ01000000_03930	NBRC101255_03874	111020438	[M] COG0744 Membrane carboxypeptidase (penicillin-binding protein)	[M] Cell wall/membrane/envelope biogenesis	CELLULAR PROCESSES AND SIGNALING	-	1 PF00905.17 Penicillin binding protein transpeptidase domain [ARO:3000129]	-	-	Penicillin-binding protein 1A

wy_03772	APJC01000001_03890	FN563149_03778	CM001149_03872	LRQY01000000_03866	LRQZ01000000_03935	NBRC101255_03879	407649417	[M] COG0744 Membrane carboxypeptidase (penicillin-binding protein)	[M] Cell wall/membrane/envelope biogenesis	CELLULAR PROCESSES AND SIGNALING	-	1 PF00905.17 Penicillin binding protein transpeptidase domain [ARO:3000129]	-	-	Penicillin-binding protein 1A
wy_03773	APJC01000001_03891	FN563149_03779	CM001149_03873	LRQY01000000_03867	LRQZ01000000_03936	NBRC101255_03880	-	-	-	-	-	-	-	-	hypothetical protein
wy_03784	APJC01000001_03902	FN563149_03790	CM001149_03884	LRQY01000000_03878	LRQZ01000000_03947	NBRC101255_03891	407644134	[N] COG1215 Glycosyltransferase, catalytic subunit of cellulose synthase and poly-beta-1,6-N-acetylglucosamine synthase	[N]Cell motility	CELLULAR PROCESSES AND SIGNALING	-	-	-	-	Beta-monoglucosyldiacylglycerol synthase
wy_03786	APJC01000001_03904	FN563149_03792	CM001149_03886	LRQY01000000_03880	LRQZ01000000_03949	NBRC101255_03893	-	-	-	-	-	-	-	-	hypothetical protein

wy_03827	APJC01000001_03960	FN563149_03833	CM001149_03929	LRQY01000000_03921	LRQZ01000000_04006	NBRC101255_03949	111020634	[K] COG1595 DNA-directed RNA polymerase specialized sigma subunit, sigma24 family	[K]Transcription	INFORMATION STORAGE AND PROCESSING	7794|vfid|9513|vsiid|11569|ssid|RNA polymerase sigma factor AlgU [Pseudomonas aeruginosa PAO1]	-	-	-	ECF RNA polymerase sigma factor SigM
wy_03828	APJC01000001_03961	FN563149_03834	CM001149_03930	LRQY01000000_03922	LRQZ01000000_04007	NBRC101255_03950	-	-	-	-	-	-	-	-	Anti-sigma-M factor RsmA
wy_03829	APJC01000001_03962	FN563149_03835	CM001149_03931	LRQY01000000_03923	LRQZ01000000_04008	NBRC101255_03951	111020636	[K] COG1309 DNA-binding transcriptio	[K]Transcription	INFORMATION STORAGE	-	-	-	-	Bacterial regulatory proteins

								nal regulator, AcrR family		AND PROCESSING					, tetR family
wy_03904	APJC01000001_04036	FN563149_03908	CM001149_04006	LRQY01000000_03999	LRQZ01000000_04080	NBRC101255_04027	407648607	[R] COG2936 Predicted acyl esterase	[R]General function prediction only	POORLY CHARACTERIZED	-	-	-	-	Cocaine esterase
wy_03905	APJC01000001_04037	FN563149_03909	CM001149_04007	LRQY01000000_04000	LRQZ01000000_04081	NBRC101255_04028	271964173	[I] COG2267 Lysophospholipase, alpha-beta hydrolase superfamily	[I]Lipid transport and metabolism	METABOLISM	-	-	-	-	hypothetical protein
wy_03906	APJC01000001_04038	FN563149_03910	CM001149_04008	LRQY01000000_04001	LRQZ01000000_04082	NBRC101255_04029	111020719	[J] COG1190 Lysyl-tRNA synthetase (class II)	[J]Translation, ribosomal structure and biogenesis	INFORMATION STORAGE AND PROCESSING	-	1 mprF: peptide antibiotic resistance gene [ARO:3000863]	-	-	Lysylphosphatidylglycerol biosynthesis bifunctional protein LysX
wy_03907	APJC01000001_04039	FN563149_03911	CM001149_04009	LRQY01000000_04002	LRQZ01000000_04083	NBRC101255_04030	111020720	[J] COG2501 Ribosome-associated protein YbcJ, S4-like RNA	[J]Translation, ribosomal structure	INFORMATION STORAGE AND PROC	-	-	-	-	ribosome-associated protein

								binding protein	and biogenesis	ESSING				
wy_03908	APJC01000001_04040	FN563149_03912	CM001149_04010	LRQY01000000_04003	LRQZ01000000_04084	NBRC101255_04031	111018562	[C] COG2041 Periplasmic DMSO/TMAO reductase YedYZ, molybdopterin-dependent catalytic subunit	[C]Energy production and conversion	METABOLISM	-	-	-	-	TMAO/DMSO reductase
wy_03909	APJC01000001_04041	FN563149_03913	CM001149_04011	LRQY01000000_04004	LRQZ01000000_04085	NBRC101255_04032	111020223	[K] COG1802 DNA-binding transcriptional regulator, GntR family	[K]Transcription	INFORMATION STORAGE AND PROCESSING	-	-	-	-	transcriptional regulator NanR
wy_03910	APJC01000001_04042	FN563149_03914	CM001149_04012	LRQY01000000_04005	LRQZ01000000_04086	NBRC101255_04033	111020225	[C] COG0277 FAD/FMN-containing dehydrogenase	[C]Energy production and conversion	METABOLISM	-	-	-	-	putative FAD-linked oxidoreductase
wy_039	APJC0100000	FN563149_03	CM00114	LRQY0100000	LRQZ0100000	NBRC101255_	111022917	[IQ] COG0318	[I]Lipid	METABOLIS	-	-	-	-	Long-chain-

13	1_04043	918	9_04014	0_04006	0_04087	04034		Acyl-CoA synthetase (AMP-forming)/AMP-acid ligase II	transport and metabolism	M					fatty-acid--CoA ligase
									[Q]Secondary metabolites biosynthesis, transport and catabolism	METABOLISM					
wy_03924	APJC01000001_04045	FN563149_03929	CM001149_04025	LRQY01000000_04008	LRQZ01000000_04089	NBRC101255_04036	470177347	[T] COG1073 Fermentation-respiration switch protein FrsA, has esterase activity, DUF1100 family	[T]Signal transduction mechanisms	CELLULAR PROCESSES AND SIGNALING	-	-	-	-	Alpha/beta hydrolase family protein
wy_03925	APJC01000001_04046	FN563149_03930	CM001149_04026	LRQY01000000_04009	LRQZ01000000_04090	NBRC101255_04037	111020722	[J] COG0684 Regulator of RNase E activity	[J]Translation, ribosomal	INFORMATION STORAGE	-	-	-	-	Putative regulator of ribonuc

								RraA	structure and biogenesis	AND PROCESSING					lease activity
wy_03926	APJC01000001_04047	FN563149_03931	CM001149_04027	LRQY01000000_04010	LRQZ01000000_04091	NBRC101255_04038	-	-	-	-	-	-	-	-	hypothetical protein
wy_03927	APJC01000001_04048	FN563149_03932	CM001149_04028	LRQY01000000_04011	LRQZ01000000_04092	NBRC101255_04039	284043817	[K] COG1309 DNA-binding transcriptional regulator, AcrR family	[K]Transcription	INFORMATION STORAGE AND PROCESSING	-	-	-	-	HTH-type transcriptional repressor KstR2
wy_03928	APJC01000001_04049	FN563149_03933	CM001149_04029	LRQY01000000_04013	LRQZ01000000_04093	NBRC101255_04040	158318652	[R] COG3173 Predicted kinase, aminoglycoside phosphotransferase (APT) family	[R]General function prediction only	POORLY CHARACTERIZED	-	1 PF01636.18 Phosphotransferase enzyme family [ARO:3000000]	-	-	Putative aminoglycoside phosphotransferase
wy_03930	APJC01000001_04051	FN563149_03935	CM001149_04031	LRQY01000000_04015	LRQZ01000000_04095	NBRC101255_04042	284043813	[I] COG1788 Acyl CoA:acetate/3-ketoacid CoA transferase,	[I]Lipid transport and metabolism	METABOLISM	-	-	-	-	Glutaconate CoA-transferase subunit A

								alpha subunit							
wy_03932	APJC01000001_04053	FN563149_03937	CM001149_04033	LRQY01000000_04017	LRQZ01000000_04097	NBRC101255_04044	284043815	[R] COG2070 NAD(P)H-dependent flavin oxidoreductase YrpB, nitropropane dioxygenase family	[R]General function prediction only	POORLY CHARACTERIZED	-	-	-	-	Nitronate monooxygenase
wy_03933	APJC01000001_04054	FN563149_03938	CM001149_04034	LRQY01000000_04018	LRQZ01000000_04098	NBRC101255_04045	470177472	[IQR] COG1028 NAD(P)-dependent dehydrogenase, short-chain alcohol dehydrogenase family	[I]Lipid transport and metabolism<br>[Q]Secondary metabolites biosynthesis, transport and catabolism	METABOLISM<br>METABOLISM	-	-	-	-	Putative short-chain type dehydrogenase/reductase

									[R]General function prediction only	POORLY CHARACTERIZED					
wy_03934	APJC01000001_04055	FN563149_03939	CM001149_04035	LRQY01000000_04019	LRQZ01000000_04099	NBRC101255_04046	393200482	[I] COG2030 Acyl dehydratase	[I]Lipid transport and metabolism	METABOLISM	-	-	-	-	hypothetical protein
wy_03935	APJC01000001_04056	FN563149_03940	CM001149_04036	LRQY01000000_04020	LRQZ01000000_04100	NBRC101255_04047	336177976	[I] COG2030 Acyl dehydratase	[I]Lipid transport and metabolism	METABOLISM	-	-	-	-	bifunctional enoyl-CoA hydratase/phosphate acetyltransferase
wy_03936	APJC01000001_04057	FN563149_03941	CM001149_04037	LRQY01000000_04021	LRQZ01000000_04101	NBRC101255_04048	472321768	[CR] COG0604 NADPH:quinone reductase or related Zn-dependent oxidoreductase	[C]Energy production and conversion	METABOLISM	-	-	-	-	Quinone oxidoreductase 1
									[R]Gener	POORLY					

									al function prediction only	CHARACTERIZED					
wy_03940	APJC01000001_04062	FN563149_03945	CM001149_04041	LRQY01000000_04052	LRQZ01000000_04106	NBRC101255_04053	-	-	-	-	-	-	-	-	hypothetical protein
wy_03982	APJC01000001_04103	FN563149_03987	CM001149_04083	LRQY01000000_04094	LRQZ01000000_04148	NBRC101255_04095	111023053	[S] COG3193 Uncharacterized conserved protein GlcG, DUF336 family	[S]Function unknown	POORLY CHARACTERIZED	-	-	-	-	hypothetical protein
wy_03998	APJC01000001_04119	FN563149_04003	CM001149_04099	LRQY01000000_04110	LRQZ01000000_04164	NBRC101255_04111	62388955	[M] COG5337 Spore coat protein CotH	[M] Cell wall/membrane/envelope biogenesis	CELLULAR PROCESSES AND SIGNALING	-	-	-	-	CotH protein
wy_04004	APJC01000001_04125	FN563149_04009	CM001149_04105	LRQY01000000_04115	LRQZ01000000_04169	NBRC101255_04117	407641258	[P] COG0607 Rhodanese-related sulfurtransferase	[P]Inorganic ion transport and meta	METABOLISM	-	-	-	-	Thiosulfate sulfurtransferase GlpE

									bolism						
wy_04021	APJC01000001_04142	FN563149_04026	CM001149_04122	LRQY01000000_04132	LRQZ01000000_04186	NBRC101255_04134	407641276	[O] COG3824 Predicted Zn-dependent protease, minimal metalloprotease (MMP)-like domain	[O]Posttranslational modification, protein turnover, chaperones	CELLULAR PROCESSES AND SIGNALING	-	-	-	-	Possibl zinc metallo-peptidase
wy_04022	APJC01000001_04143	FN563149_04027	CM001149_04123	LRQY01000000_04133	LRQZ01000000_04187	NBRC101255_04135	-	-	-	-	-	-	-	-	putative membrane protein/MT3943
wy_04031	APJC01000001_04160	FN563149_04036	CM001149_04137	LRQY01000000_04142	LRQZ01000000_04205	NBRC101255_04151	111021030	[I] COG0204 1-acyl-sn-glycerol-3-phosphate acyltransferase	[I]Lipid transport and metabolism	METABOLISM	-	1 PF01553.16 Acyltransferase [ARO:3000000]	-	-	1-acyl-sn-glycerol-3-phosphate acyltransferase
wy_04091	APJC01000001_04210	FN563149_04090	CM001149_04196	LRQY01000000_04192	LRQZ01000000_04255	NBRC101255_04202	404216581	[GEPR] COG0477 MFS family permease	[E]Amino acid transport	METABOLISM	-	2 PF07690.11 Major Facilita	Mrad2831_1269_HK_Cl	MFS-PP44332699 1121	Proline/betaine transporter

									and metabolism			tor Superfamily [ARO:0010002]	assic|Methylobacterium	MFS amino acid MFS transporter [Pseudomonas putida KT2440]	
									[G]Carbohydrate transport and metabolism	METABOLISM					
									[P]Inorganic ion transport and metabolism	METABOLISM					
									[R]General function prediction only	POORLY CHARACTERIZED					
wy_04106	APJC01000001_04222	FN563149_04102	CM001149_04208	LRQY01000000_04204	LRQZ01000000_04267	NBRC101255_04214	111021100	[E] COG0520 Selenocysteine lyase/Cysteine	[E]Amino acid transport and	METABOLISM	-	-	-	-	Cysteine desulfurase CsdA

								desulfurase	metabolism						
wy_04107	APJC01000001_04223	FN563149_04103	CM001149_04209	LRQY01000000_04205	LRQZ01000000_04268	NBRC101255_04215	111021101	[CR] COG0604 NADPH:quinone reductase or related Zn-dependent oxidoreductase	[C]Energy production and conversion	METABOLISM	-	-	-	-	Quinone oxidoreductase 1
									[R]General function prediction only	POORLY CHARACTERIZED					
wy_04110	APJC01000001_04226	FN563149_04106	CM001149_04212	LRQY01000000_04208	LRQZ01000000_04271	NBRC101255_04218	111019497	[J] COG1734 RNA polymerase-binding transcription factor DksA	[J]Translation, ribosomal structure and biogenesis	INFORMATION STORAGE AND PROCESSING	-	-	-	-	General stress protein 16O

wy_04111	APJC01000001_04227	FN563149_04107	CM001149_04213	LRQY01000000_04209	LRQZ01000000_04272	NBRC101255_04219	111021108	[V] COG0627 S-formylglutathione hydrolase FrmB	[V] Defense mechanisms	CELLULAR PROCESSES AND SIGNALING	2470|vfid|2678|vsiid|2999|ssid|secreted antigen 85-A FBPA Mycolyltransferase 85A fibronectin-binding protein A antigen 85 complex A [Mycoba	-	-	-	Diacylglycerol acyltransferase/mycolyltransferase Ag85A precursor

											cterium tuberculosis H37Rv]				
wy_04114	APJC01000001_04235	FN563149_04110	CM001149_04221	LRQY01000000_04212	LRQZ01000000_04280	NBRC101255_04227	-	-	-	-	-	-	-	-	hypothetical protein
wy_04115	APJC01000001_04236	FN563149_04111	CM001149_04222	LRQY01000000_04213	LRQZ01000000_04281	NBRC101255_04228	111021109	[FV] COG0737 2',3'-cyclic-nucleotide 2'-phosphodiesterase/5'- or 3'-nucleotidase, 5'-nucleotidase family	[F]Nucleotide transport and metabolism	METABOLISM	-	-	-	-	Endonuclease YhcR precursor
									[V] Defense mechani	CELLULAR PROCESSES AND					

									sms	SIGNALING					
wy_04118	APJC01000001_04238	FN563149_04113	CM001149_04224	LRQY01000000_04215	LRQZ01000000_04283	NBRC101255_04230	111021111	[Q] COG0412 Dienelactone hydrolase	[Q]Secondary metabolites biosynthesis, transport and catabolism	METABOLISM	-	-	-	-	Dienelactone hydrolase family protein
wy_04192	APJC01000001_04326	FN563149_04185	CM001149_04290	LRQY01000000_04290	LRQZ01000000_04370	NBRC101255_04318	111017599	[S] COG3795 Uncharacterized conserved protein	[S]Function unknown	POORLY CHARACTERIZED	-	-	-	-	hypothetical protein
wy_04193	APJC01000001_04327	FN563149_04186	CM001149_04291	LRQY01000000_04291	LRQZ01000000_04371	NBRC101255_04319	-	-	-	-	-	-	-	-	hypothetical protein
wy_04194	APJC01000001_04328	FN563149_04187	CM001149_04292	LRQY01000000_04292	LRQZ01000000_04372	NBRC101255_04320	-	-	-	-	-	-	-	-	hypothetical protein

wy_04195	APJC01000001_04329	FN563149_04188	CM001149_04293	LRQY01000000_04293	LRQZ01000000_04373	NBRC101255_04321	111019383	[H] COG2226 Ubiquinone/menaquinone biosynthesis C-methylase UbiE	[H]Coenzyme transport and metabolism	METABOLISM	-	1 PF12847.2 Methyltransferase domain [ARO:3000000]	-	-	Ubiquinone/menaquinone biosynthesis C-methyltransferase UbiE
wy_04197	APJC01000001_04331	FN563149_04190	CM001149_04295	LRQY01000000_04295	LRQZ01000000_04375	NBRC101255_04323	111021153	[S] COG1738 Uncharacterized PurR-regulated membrane protein YhhQ, DUF165 family	[S]Function unknown	POORLY CHARACTERIZED	-	-	-	-	hypothetical protein
wy_04198	APJC01000001_04332	FN563149_04191	CM001149_04296	LRQY01000000_04296	LRQZ01000000_04376	NBRC101255_04324	333918551	[S] COG5473 Uncharacterized membrane protein	[S]Function unknown	POORLY CHARACTERIZED	-	-	-	-	hypothetical protein
wy_04219	APJC01000001_04354	FN563149_04212	CM001149_04318	LRQY01000000_04317	LRQZ01000000_04398	NBRC101255_04346	384148667	[J] COG2867 Ribosome association toxin PasT (RatA) of the RatAB toxin-antitoxin module	[J]Translation, ribosomal structure and biogenesis	INFORMATION STORAGE AND PROCESSING	-	-	-	-	Polyketide cyclase / dehydrase and lipid transport

wy_04220	APJC01000001_04355	FN563149_04213	CM001149_04319	LRQY01000000_04318	LRQZ01000000_04399	NBRC101255_04347	119718217	[G] COG0524 Sugar or nucleoside kinase, ribokinase family	[G]Carbohydrate transport and metabolism	METABOLISM	-	-	-	-	putative sugar kinase YdjH
wy_04230	APJC01000001_04364	FN563149_04223	CM001149_04330	LRQY01000000_04328	LRQZ01000000_04408	NBRC101255_04356	111019503	[K] COG2207 AraC-type DNA-binding domain and AraC-containing proteins	[K]Transcription	INFORMATION STORAGE AND PROCESSING	-	2 "PF00165.18 Bacterial regulatory helix-turn-helix proteins, AraC family" [ARO:3000000]	-	-	HTH-type transcriptional regulator CdhR
wy_04241	APJC01000001_04375	FN563149_04234	CM001149_04341	LRQY01000000_04339	LRQZ01000000_04419	NBRC101255_04367	148555011	[IQR] COG1028 NAD(P)-dependent dehydrogenase, short-chain alcohol dehydrogenase family	[I]Lipid transport and metabolism<br>[Q]Secondary meta	METABOLISM<br>METABOLISM	-	-	-	-	3-oxoacyl-[acyl-carrier-protein] reductase FabG

									bolites biosynthesis, transport and catabolism						
									[R]General function prediction only	POORLY CHARACTERIZED					
wy_04269	APJC01000001_04403	FN563149_04262	CM001149_04369	LRQY01000000_04367	LRQZ01000000_04447	NBRC101255_04395	111023086	[M] COG1463 ABC-type transporter Mla maintaining outer membrane lipid asymmetry, periplasmic component MlaD	[M] Cell wall/membrane/envelope biogenesis	CELLULAR PROCESSES AND SIGNALING	-	-	-	-	mce related protein
wy_04282	APJC01000001_04423	FN563149_04275	CM001149_04390	LRQY01000000_04380	LRQZ01000000_04467	NBRC101255_04416	284042951	[CP] COG2181 Nitrate reductase gamma	[C]Energy productio	METABOLISM	-	-	-	-	C4-dicarboxylate transporter/mal

								subunit	n and conversion						ic acid transport protein
									[P]Inorganic ion transport and metabolism	METABOLISM					
wy_04294	APJC01000001_04438	FN563149_04290	CM001149_04419	LRQY01000000_04394	LRQZ01000000_04482	NBRC101255_04431	111021244	[S] COG4292 Low temperature requirement protein LtrA (function unknown)	[S]Function unknown	POORLY CHARACTERIZED	-	-	-	-	Bacterial low temperature requirement A protein (LtrA)
wy_04305	APJC01000001_04450	FN563149_04302	CM001149_04430	LRQY01000000_04405	LRQZ01000000_04494	NBRC101255_04443	336119150	[P] COG0038 H+/Cl- antiporter ClcA	[P]Inorganic ion transport and metabolism	METABOLISM	-	-	-	ClC-b238916130321 ClC orf, hypothetical protein [Escherichi	putative ion channel protein

														a coli K12-MG1655]	
wy_04393	APJC01000001_04575	FN563149_04382	CM001149_04551	LRQY01000000_04536	LRQZ01000000_04620	NBRC101255_04567	111021315	[UW] COG4963 Flp pilus assembly protein, ATPase CpaE	[U]Intracellular trafficking, secretion, and vesicular transport	CELLULAR PROCESSES AND SIGNALING	-	-	-	-	hypothetical protein
									[W] Extracellular structures	CELLULAR PROCESSES AND SIGNALING					
wy_04415	APJC01000001_04597	FN563149_04404	CM001149_04573	LRQY01000000_04555	LRQZ01000000_04639	NBRC101255_04588	111021353	[E] COG0308 Aminopeptidase N	[E]Amino acid transport and	METABOLISM	-	-	-	-	Aminopeptidase N

									metabolism						
wy_04457	APJC01000001_04644	FN563149_04446	CM001149_04620	LRQY01000000_04597	LRQZ01000000_04686	NBRC101255_04635	403510573	[M] COG0815 Apolipoprotein N-acyltransferase	[M] Cell wall/membrane/envelope biogenesis	CELLULAR PROCESSES AND SIGNALING	-	-	-	-	hypothetical protein
wy_04459	APJC01000001_04646	FN563149_04448	CM001149_04622	LRQY01000000_04599	LRQZ01000000_04688	NBRC101255_04637	556564367	[K] COG1595 DNA-directed RNA polymerase specialized sigma subunit, sigma24 family	[K]Transcription	INFORMATION STORAGE AND PROCESSING	-	-	-	-	putative ECF RNA polymerase sigma factor SigI
wy_04481	APJC01000001_04667	FN563149_04469	CM001149_04643	LRQY01000000_04620	LRQZ01000000_04709	NBRC101255_04658	111021420	[L] COG1194 Adenine-specific DNA glycosylase, acts on AG and A-oxoG pairs	[L]Replication, recombination and repair	INFORMATION STORAGE AND PROCESSING	-	-	-	-	A/G-specific adenine glycosylase

wy_04492	APJC01000001_04678	FN563149_04480	CM001149_04654	LRQY01000000_04631	LRQZ01000000_04720	NBRC101255_04669	407641673	[J] COG0566 tRNA G18 (ribose-2'-O)-methylase SpoU	[J]Translation, ribosomal structure and biogenesis	INFORMATION STORAGE AND PROCESSING	-	-	-	-	Putative TrmH family tRNA/rRNA methyltransferase
wy_04493	APJC01000001_04679	FN563149_04481	CM001149_04655	LRQY01000000_04632	LRQZ01000000_04721	NBRC101255_04670	111021437	[E] COG1984 Allophanate hydrolase subunit 2	[E]Amino acid transport and metabolism	METABOLISM	-	-	-	-	KipI antagonist
wy_04494	APJC01000001_04680	FN563149_04482	CM001149_04656	LRQY01000000_04633	LRQZ01000000_04722	NBRC101255_04671	111017671	[P] COG0428 Zinc transporter ZupT	[P]Inorganic ion transport and metabolism	METABOLISM	-	-	-	-	ZIP Zinc transporter
wy_04495	APJC01000001_04681	FN563149_04483	CM001149_04657	LRQY01000000_04634	LRQZ01000000_04723	NBRC101255_04672	111021443	[E] COG2049 Allophanate hydrolase subunit 1	[E]Amino acid transport and metabolis	METABOLISM	-	-	SCATT_02250_PP_HisKa|Streptomyces	-	Kinase A inhibitor

									m						
wy_04518, wy_00669	APJC01000001_04704	FN563149_00660,FN563149_04506	CM001149_00688, CM001149_04680	LRQY01000000_04657,LRQY01000000_00685	LRQZ01000000_04746,LRQZ01000000_00797	NBRC101255_00684, NBRC101255_04695	397775666	[M] COG0399 dTDP-4-amino-4,6-dideoxygalactose transaminase	[M] Cell wall/membrane/envelope biogenesis	CELLULAR PROCESSES AND SIGNALING	15205|vfid|24403|vsiid|45223|ssid|SubName: Full=Lipopolysaccharide biosynthesis protein O-antigen-related ;	-	-	-	UDP-4-amino-4-deoxy-L-arabinose--oxoglutarate aminotransferase
wy_04566	APJC01000001_04752	FN563149_04553	CM001149_04727	LRQY01000000_04704	LRQZ01000000_04794	NBRC101255_04743	556567616	[HR] COG0596 Pimeloyl-ACP methyl ester carboxylest	[H]Coenzyme transport and meta	METABOLISM	-	-	-	-	2-hydroxy-6-oxononadienedioate/2-hydrox

								erase	bolism<br>[R]General function prediction only	 POORLY CHARACTERIZED					y-6-oxononatrienedioate hydrolase
wy_04636	APJC01000001_04824	FN563149_04626	CM001149_04799	LRQY01000000_04777	LRQZ01000000_04867	NBRC101255_04815	111021714	[FGR] COG0537 Diadenosine tetraphosphate (Ap4A) hydrolase or other HIT family hydrolase	[F]Nucleotide transport and metabolism<br>[G]Carbohydrate transport and metabolism<br>[R]General function prediction only	METABOLISM<br>METABOLISM<br>POORLY CHARACTERIZED	-	-	-	-	HIT-like protein

wy_04637	APJC01000001_04825	FN563149_04627	CM001149_04800	LRQY01000000_04778	LRQZ01000000_04868	NBRC101255_04816	111022641	[R] COG1434 Uncharacterized SAM-binding protein YcdF, DUF218 family	[R]General function prediction only	POORLY CHARACTERIZED	-	-	-	-	hypothetical protein
wy_04639	APJC01000001_04827	FN563149_04629	CM001149_04802	LRQY01000000_04780	LRQZ01000000_04870	NBRC101255_04818	111021716	[M] COG1807 4-amino-4-deoxy-L-arabinose transferase or related glycosyltransferase of PMT family	[M] Cell wall/membrane/envelope biogenesis	CELLULAR PROCESSES AND SIGNALING	-	-	-	-	hypothetical protein
wy_04651	APJC01000001_04839	FN563149_04641	CM001149_04814	LRQY01000000_04792	LRQZ01000000_04882	NBRC101255_04830	407642519	[K] COG1309 DNA-binding transcriptional regulator, AcrR family	[K]Transcription	INFORMATION STORAGE AND PROCESSING	-	-	-	-	Bacterial regulatory proteins, tetR family

Supplementary Table S7. Functional annotation of the human-unique genes				
Orthologs from human-associated species		Resistance	TCS	Annotation
WY	ATCC 33707			
wy_00032	CM001149_00032	-	-	hypothetical protein
wy_00082	CM001149_00083	-	-	hypothetical protein
wy_00204	CM001149_00230	-	-	hypothetical protein
wy_00657	CM001149_00677	-	-	hypothetical protein
wy_01649	CM001149_04974	-	-	Phage antirepressor protein KilAC domain protein
wy_01752	CM001149_01826	-	-	hypothetical protein
wy_02940	CM001149_03018	-	-	hypothetical protein
wy_03129	CM001149_03209	-	-	Heat shock protein 15
wy_03690	CM001149_03788	-	-	hypothetical protein
wy_03739	CM001149_03839	-	-	hypothetical protein
wy_03912	CM001149_04013	-	-	putative enoyl-CoA hydratase echA8
wy_03915	CM001149_04016	-	-	hypothetical protein
wy_03916	CM001149_04017	-	-	Carbazole 1,9a-dioxygenase, terminal oxygenase component CarAa

wy_03917	CM001149_04018	-	-	Methane monooxygenase component C
wy_03918	CM001149_04019	2 PF00903.20 Glyoxalase/Bleomycin resistance protein/Dioxygenase superfamily [ARO:3000000]	-	2,2',3-trihydroxybiphenyl dioxygenase
wy_03919	CM001149_04020	-	Tmar_0491_PP_HisKa|Thermaerobacter	Glutamyl-tRNA(Gln) amidotransferase subunit A
wy_03920	CM001149_04021	-	-	5-nitroanthranilic acid aminohydrolase
wy_03921	CM001149_04022	-	-	Asp/Glu/Hydantoin racemase
wy_03922	CM001149_04023	-	-	2-hydroxymuconate semialdehyde hydrolase
wy_03923	CM001149_04024	-	-	4-hydroxyacetophenone monooxygenase
wy_04063	CM001149_04169	-	-	hypothetical protein
wy_04165	CM001149_04266	-	-	hypothetical protein
wy_04225	CM001149_04325	-	-	hypothetical protein
wy_04776	CM001149_01609	-	-	hypothetical protein
wy_04777	CM001149_01610	-	-	DNA translocase SftA
wy_04778	CM001149_01611	-	-	hypothetical protein
wy_04779	CM001149_01612	-	-	Single-stranded DNA-binding protein

wy_04780	CM001149_01613	-	-	hypothetical protein
wy_04781	CM001149_01614	-	-	hypothetical protein
wy_04782	CM001149_01615	-	-	hypothetical protein
wy_04783	CM001149_01616	-	-	hypothetical protein
wy_04784	CM001149_01617	-	-	hypothetical protein
wy_04785	CM001149_04961,CM001149_01619	-	-	hypothetical protein
wy_04791	CM001149_04952	-	-	hypothetical protein
wy_04795	CM001149_04946,CM001149_01629	-	-	Terminase-like family protein
wy_04796	CM001149_04945,CM001149_01630	-	-	hypothetical protein
wy_04797	CM001149_01631	-	-	ATP-dependent Clp protease proteolytic subunit
wy_04798	CM001149_01632	-	-	hypothetical protein
wy_04800	CM001149_01634	-	-	hypothetical protein
wy_04801	CM001149_04940	-	-	hypothetical protein
wy_04813	CM001149_01646	-	-	LGFP repeat protein
wy_04814	CM001149_01647	-	-	hypothetical protein

wy_04816	CM001149_01651	-	-	hypothetical protein
wy_04835,wy_04770	CM001149_01603	-	-	DNA-damage-inducible protein D
wy_04838,wy_04773	CM001149_01606	-	-	hypothetical protein
wy_04839,wy_04774	CM001149_01607	-	-	hypothetical protein
wy_04840,wy_04775	CM001149_01608	-	-	hypothetical protein
wy_04850,wy_04821	CM001149_01656	-	-	hypothetical protein
wy_04851,wy_04822	CM001149_04913,CM001149_01657	-	-	hypothetical protein

Supplementary Table S8. Pathway enrichment analysis of the stable core genes						
Pathway	In stable core genome	In core genome	Stable core genes	Core genes	P-value	FDR
Biosynthesis of amino acids [PATH:ko01230]	33	33	231	280	0	0
Ribosome [PATH:ko03010]	27	27	231	280	0	0
Butanoate metabolism [PATH:ko00650]	11	11	231	280	0	0
Carbon fixation pathways in prokaryotes [PATH:ko00720]	11	11	231	280	0	0
Glycine, serine and threonine metabolism [PATH:ko00260]	11	11	231	280	0	0
Glyoxylate and dicarboxylate metabolism [PATH:ko00630]	10	10	231	280	0	0
Phenylalanine, tyrosine and tryptophan biosynthesis [PATH:ko00400]	9	9	231	280	0	0
Pyrimidine metabolism [PATH:ko00240]	9	9	231	280	0	0
2-Oxocarboxylic acid metabolism [PATH:ko01210]	7	7	231	280	0	0
Methane metabolism [PATH:ko00680]	7	7	231	280	0	0
beta-Alanine metabolism [PATH:ko00410]	6	6	231	280	0	0
Cysteine and methionine metabolism [PATH:ko00270]	6	6	231	280	0	0
Lysine biosynthesis [PATH:ko00300]	6	6	231	280	0	0
Lysine degradation [PATH:ko00310]	6	6	231	280	0	0
Pantothenate and CoA biosynthesis [PATH:ko00770]	6	6	231	280	0	0
Protein export [PATH:ko03060]	6	6	231	280	0	0
Tryptophan metabolism [PATH:ko00380]	6	6	231	280	0	0
Bacterial secretion system [PATH:ko03070]	5	5	231	280	0	0
Monobactam biosynthesis [PATH:ko00261]	5	5	231	280	0	0
Nitrogen metabolism [PATH:ko00910]	5	5	231	280	0	0
Pentose phosphate pathway [PATH:ko00030]	5	5	231	280	0	0
Synthesis and degradation of ketone bodies [PATH:ko00072]	5	5	231	280	0	0

Valine, leucine and isoleucine biosynthesis [PATH:ko00290]	5	5	231	280	0	0
Arginine biosynthesis [PATH:ko00220]	4	4	231	280	0	0
C5-Branched dibasic acid metabolism [PATH:ko00660]	4	4	231	280	0	0
Homologous recombination [PATH:ko03440]	4	4	231	280	0	0
Longevity regulating pathway - worm [PATH:ko04212]	4	4	231	280	0	0
One carbon pool by folate [PATH:ko00670]	4	4	231	280	0	0
Porphyrin and chlorophyll metabolism [PATH:ko00860]	4	4	231	280	0	0
Sulfur relay system [PATH:ko04122]	4	4	231	280	0	0
DNA replication [PATH:ko03030]	3	3	231	280	0	0
Histidine metabolism [PATH:ko00340]	3	3	231	280	0	0
Mismatch repair [PATH:ko03430]	3	3	231	280	0	0
Peroxisome [PATH:ko04146]	3	3	231	280	0	0
Riboflavin metabolism [PATH:ko00740]	3	3	231	280	0	0
RNA degradation [PATH:ko03018]	3	3	231	280	0	0
RNA polymerase [PATH:ko03020]	3	3	231	280	0	0
Selenocompound metabolism [PATH:ko00450]	3	3	231	280	0	0
Vancomycin resistance [PATH:ko01502]	3	3	231	280	0	0
Arginine and proline metabolism [PATH:ko00330]	2	2	231	280	0	0
beta-Lactam resistance [PATH:ko01501]	2	2	231	280	0	0
Biosynthesis of unsaturated fatty acids [PATH:ko01040]	2	2	231	280	0	0
Caprolactam degradation [PATH:ko00930]	2	2	231	280	0	0
Carbon fixation in photosynthetic organisms [PATH:ko00710]	2	2	231	280	0	0
Central carbon metabolism in cancer [PATH:ko05230]	2	2	231	280	0	0
Drug metabolism - other enzymes [PATH:ko00983]	2	2	231	280	0	0
Fatty acid biosynthesis [PATH:ko00061]	2	2	231	280	0	0

Glutathione metabolism [PATH:ko00480]	2	2	231	280	0	0
Limonene and pinene degradation [PATH:ko00903]	2	2	231	280	0	0
Novobiocin biosynthesis [PATH:ko00401]	2	2	231	280	0	0
Ribosome biogenesis in eukaryotes [PATH:ko03008]	2	2	231	280	0	0
Thiamine metabolism [PATH:ko00730]	2	2	231	280	0	0
Tropane, piperidine and pyridine alkaloid biosynthesis [PATH:ko00960]	2	2	231	280	0	0
Vitamin B6 metabolism [PATH:ko00750]	2	2	231	280	0	0
Adipocytokine signaling pathway [PATH:ko04920]	1	1	231	280	0	0
African trypanosomiasis [PATH:ko05143]	1	1	231	280	0	0
AMPK signaling pathway [PATH:ko04152]	1	1	231	280	0	0
Apoptosis - fly [PATH:ko04214]	1	1	231	280	0	0
Arachidonic acid metabolism [PATH:ko00590]	1	1	231	280	0	0
Cationic antimicrobial peptide (CAMP) resistance [PATH:ko01503]	1	1	231	280	0	0
Chagas disease (American trypanosomiasis) [PATH:ko05142]	1	1	231	280	0	0
Chloroalkane and chloroalkene degradation [PATH:ko00625]	1	1	231	280	0	0
D-Alanine metabolism [PATH:ko00473]	1	1	231	280	0	0
D-Glutamine and D-glutamate metabolism [PATH:ko00471]	1	1	231	280	0	0
Dioxin degradation [PATH:ko00621]	1	1	231	280	0	0
Drug metabolism - cytochrome P450 [PATH:ko00982]	1	1	231	280	0	0
FoxO signaling pathway [PATH:ko04068]	1	1	231	280	0	0
GABAergic synapse [PATH:ko04727]	1	1	231	280	0	0
Glucagon signaling pathway [PATH:ko04922]	1	1	231	280	0	0
Glutamatergic synapse [PATH:ko04724]	1	1	231	280	0	0
Glycosaminoglycan degradation [PATH:ko00531]	1	1	231	280	0	0
Huntington's disease [PATH:ko05016]	1	1	231	280	0	0

Longevity regulating pathway - mammal [PATH:ko04211]	1	1	231	280	0	0
Longevity regulating pathway - multiple species [PATH:ko04213]	1	1	231	280	0	0
MAPK signaling pathway - fly [PATH:ko04013]	1	1	231	280	0	0
Metabolism of xenobiotics by cytochrome P450 [PATH:ko00980]	1	1	231	280	0	0
Naphthalene degradation [PATH:ko00626]	1	1	231	280	0	0
N-Glycan biosynthesis [PATH:ko00510]	1	1	231	280	0	0
Nucleotide excision repair [PATH:ko03420]	1	1	231	280	0	0
Pathways in cancer [PATH:ko05200]	1	1	231	280	0	0
Phosphatidylinositol signaling system [PATH:ko04070]	1	1	231	280	0	0
Primary bile acid biosynthesis [PATH:ko00120]	1	1	231	280	0	0
Proteoglycans in cancer [PATH:ko05205]	1	1	231	280	0	0
Renal cell carcinoma [PATH:ko05211]	1	1	231	280	0	0
Retinol metabolism [PATH:ko00830]	1	1	231	280	0	0
Thyroid hormone synthesis [PATH:ko04918]	1	1	231	280	0	0
Tuberculosis [PATH:ko05152]	1	1	231	280	0	0
Type I diabetes mellitus [PATH:ko04940]	1	1	231	280	0	0
Vibrio cholerae pathogenic cycle [PATH:ko05111]	1	1	231	280	0	0
Purine metabolism [PATH:ko00230]	17	18	231	280	0.0277578	0.0450293
Carbon metabolism [PATH:ko01200]	25	27	231	280	0.0310736	0.0498543
Propanoate metabolism [PATH:ko00640]	13	14	231	280	0.062995	0.0999704
Valine, leucine and isoleucine degradation [PATH:ko00280]	12	13	231	280	0.0771545	0.1211242
Aminoacyl-tRNA biosynthesis [PATH:ko00970]	9	10	231	280	0.1410434	0.2167615
Fatty acid metabolism [PATH:ko01212]	9	10	231	280	0.1410434	0.2167615
Terpenoid backbone biosynthesis [PATH:ko00900]	8	9	231	280	0.1721746	0.2618489
Alanine, aspartate and glutamate metabolism [PATH:ko00250]	6	7	231	280	0.2559457	0.3774553
Citrate cycle (TCA cycle) [PATH:ko00020]	6	7	231	280	0.2559457	0.3774553

Peptidoglycan biosynthesis [PATH:ko00550]	6	7	231	280	0.2559457	0.3774553
Glycerophospholipid metabolism [PATH:ko00564]	5	6	231	280	0.311685	0.4550601
Fatty acid degradation [PATH:ko00071]	10	12	231	280	0.3467691	0.4963558
Pyruvate metabolism [PATH:ko00620]	10	12	231	280	0.3467691	0.4963558
Amino sugar and nucleotide sugar metabolism [PATH:ko00520]	4	5	231	280	0.3792627	0.5223807
Aminobenzoate degradation [PATH:ko00627]	4	5	231	280	0.3792627	0.5223807
Cell cycle - Caulobacter [PATH:ko04112]	4	5	231	280	0.3792627	0.5223807
Tyrosine metabolism [PATH:ko00350]	4	5	231	280	0.3792627	0.5223807
Fructose and mannose metabolism [PATH:ko00051]	3	4	231	280	0.46113	0.6176603
Nicotinate and nicotinamide metabolism [PATH:ko00760]	3	4	231	280	0.46113	0.6176603
PPAR signaling pathway [PATH:ko03320]	3	4	231	280	0.46113	0.6176603
Benzoate degradation [PATH:ko00362]	7	9	231	280	0.5126635	0.6623793
Glycolysis / Gluconeogenesis [PATH:ko00010]	7	9	231	280	0.5126635	0.6623793
Phenylalanine metabolism [PATH:ko00360]	7	9	231	280	0.5126635	0.6623793
Sulfur metabolism [PATH:ko00920]	7	9	231	280	0.5126635	0.6623793
Geraniol degradation [PATH:ko00281]	2	3	231	280	0.5602325	0.6990935
Glycerolipid metabolism [PATH:ko00561]	2	3	231	280	0.5602325	0.6990935
Inositol phosphate metabolism [PATH:ko00562]	2	3	231	280	0.5602325	0.6990935
Taurine and hypotaurine metabolism [PATH:ko00430]	2	3	231	280	0.5602325	0.6990935
Folate biosynthesis [PATH:ko00790]	6	8	231	280	0.5775185	0.7145568
Cyanoamino acid metabolism [PATH:ko00460]	1	2	231	280	0.6801075	0.7880611
Galactose metabolism [PATH:ko00052]	1	2	231	280	0.6801075	0.7880611
Legionellosis [PATH:ko05134]	1	2	231	280	0.6801075	0.7880611
Pentose and glucuronate interconversions [PATH:ko00040]	1	2	231	280	0.6801075	0.7880611
Primary immunodeficiency [PATH:ko05340]	1	2	231	280	0.6801075	0.7880611
Streptomycin biosynthesis [PATH:ko00521]	1	2	231	280	0.6801075	0.7880611

Ubiquinone and other terpenoid-quinone biosynthesis [PATH:ko00130]	1	2	231	280	0.6801075	0.7880611
Xylene degradation [PATH:ko00622]	1	2	231	280	0.6801075	0.7880611
Base excision repair [PATH:ko03410]	4	6	231	280	0.7171513	0.8244416
alpha-Linolenic acid metabolism [PATH:ko00592]	0	1	231	280	0.825	0.8791971
Butirosin and neomycin biosynthesis [PATH:ko00524]	0	1	231	280	0.825	0.8791971
Carotenoid biosynthesis [PATH:ko00906]	0	1	231	280	0.825	0.8791971
Chlorocyclohexane and chlorobenzene degradation [PATH:ko00361]	0	1	231	280	0.825	0.8791971
Ethylbenzene degradation [PATH:ko00642]	0	1	231	280	0.825	0.8791971
Fluorobenzoate degradation [PATH:ko00364]	0	1	231	280	0.825	0.8791971
Phenylpropanoid biosynthesis [PATH:ko00940]	0	1	231	280	0.825	0.8791971
Plant-pathogen interaction [PATH:ko04626]	0	1	231	280	0.825	0.8791971
Proteasome [PATH:ko03050]	0	1	231	280	0.825	0.8791971
Salmonella infection [PATH:ko05132]	0	1	231	280	0.825	0.8791971
Starch and sucrose metabolism [PATH:ko00500]	2	4	231	280	0.8575399	0.9007254
Steroid degradation [PATH:ko00984]	2	4	231	280	0.8575399	0.9007254
Two-component system [PATH:ko02020]	6	10	231	280	0.9226142	0.9621548
Oxidative phosphorylation [PATH:ko00190]	5	10	231	280	0.982978	0.9966305
Degradation of aromatic compounds [PATH:ko01220]	3	7	231	280	0.9805173	0.9966305
Biosynthesis of siderophore group nonribosomal peptides [PATH:ko01053]	0	2	231	280	0.9698925	0.9966305
Toluene degradation [PATH:ko00623]	0	2	231	280	0.9698925	0.9966305
ABC transporters [PATH:ko02010]	14	24	231	280	0.9968489	0.9968489
Photosynthesis [PATH:ko00195]	0	3	231	280	0.9949099	0.9968489

*Column 2 denotes the number of genes involved in each pathway in stable core genome; Column 3 denotes the number of genes involved in each pathway in core genome; Column 4 denotes the number of genes involved in all metabolic pathways in stable core genome; Column 5 denotes the number of genes involved in all metabolic pathways in core genome.

Supplementary Table S9. Prediction of prophages and functional annotation in *R. equi* N1301 and NBRC 101255								
CDS	From	To	Strand	Virulence	Resistance	Completeness	Detail-predicted by PHASTER	
N1301_00473	460928	462250	+			intact	Phage-like_protein	DNA helicase;phage(gi971742508);PHAGE_Mycoba_Snenia_NC_028778 5.03e-73 MNIIGSSFDHTEGLLESVEGEVTLVATLLSVASRHLVPDLLDEIHGSDFSDPHLGLVWESAQQLARDSQRISKRTLLKTRDTPAIRARLDALAGEPVKDLAVREAVASVLEMSRRRELLNTLRVSAEYAANADTYSQALHFASERLGTLTEGSTPDEVRSFADAVSAWQEWMAEPPHVARTIHTPWSDLDEMLSGGLKPGRTYVIGGRPGEGKSVGLLNLVTHAAQNGHPGVVFSVEMSETEVVSRVIAAGAAADYGQITRRQLDDYNHTRIHHYVDQFRDMPLKLVDKADVTVAYIASTCRTLKRTQGLDVVVVDYLQLLKETDSKQVRERQVAEISRALKVLSRELDCAVIIACQLNRGPANQDRKPALPDLRESGAIEQDADVVILLHHEQMNGERTGMVELVLAKNRTGKLGGVLVEWRGYQARFTSPTRKTTFTE
N1301_00474	462408	463088	+			intact	Hypothetical_protein	hypothetical protein;phage(gi372450001);PHAGE_Rhodoc_REQ3_NC_016654 1.24e-30 MPDQFFITRADMQALVAKLREIPELVANLDITLTRQSRTGAGGMGTTNGTDDQPLPYDLGASEAAHRLYGVLRDWAEYVATEHGIAPPSLGRAPHYARWLIEHVTHLAMTEAAPDALPGITKTIERTEKAIDIPEEPPPLPINKAKLTQAESLELNARSCVAIAKRLGRWHEGLTQRRIKYLTETKVLTPLRTVRGIDGARHRIYRLGDVLDATRNDDTDTFDESA
N1301_00475	463434	464015	-		Acetyltransf_1;PF00583.19 Acetyltransferase (GNAT) family [ARO:3000000]	intact	Non_phage-like_protein	Acetyltransferase (GNAT) family protein N/A MMIECLDTAESRTELQKFFASLIPDIEYNAVPDVEHDDVYAPLALAIRDGGSGEIVAALLSCHTQFVATALMTDARGLPFKDPDAVRLGGRHSLLDLMATAPEARKRGLATKLLEHAETELASRGVRAWYGCVDDESHADELRAFYERRGFRVLPDGAPLPPLLGTDRWNDEVTPPVFWFYKSIRSIGGSSAR

N1 301 _00 476	4641 01	46 43 46	+			intact	Hypothetical_prote in	hypothetical protein N/A MTAAAQTQIGGYALTPAALRDAGMLGVTIQEIGK ALRNPIPVVPVPRTDGRCNTYGEIANGRNIRMILHP LGSRIENITRA
N1 301 _00 477	4648 02	46 51 43	+			intact	Terminase	terminase small subunit;phage(gi971482396);PHAGE_Mycoba_Brusacor am_NC_028747 1.90e-18 MSESDALRAAGRRLYESLHKDDEPYELTAMIVEA ARIKDRLDELDRVLSGDADVWMRLDEVRGNGDV LEIRIDSAMQESRQQANTLRQLLGEIRRQKDAAGG DGDDYDPLDDL
N1 301 _00 478	4651 21	46 66 83	+			intact	Terminase	large terminase subunit;phage(gi589893327);PHAGE_Mycoba_32HC_N C_023602 2.07e-93 MTPSTICDEDIDAKFGEIIAAEWPKLEGRQTPNSLA YTPGDVALGLKAVELAKRSASRIRAMPWQSWSLE RIMSKAADGTWTHPECCLIVPRQNGKSLILSLRVL YGLFKLGEKIVFSAQQWETAKALWKRTWAIVKTT PWMRKHVESHTCSQGRGTIVLASGAEVVFTTRSA NAGRGLDRVDLEIYDEAYDLTEADMAALSPTKMN SDDPQTIYTSSAVNQDQHPNGQVLAAVRQRGHEG EEGLFFAEYMAPEELDRADPQTWRWANPSFGVIQ TDKKLAAELRKFSTDAGKKSFDVEYLGRGDWPGA VDETPAVIEAEHWNTMTNPTPERVGPISLAVDATP NAEWWTVAAAQRTSNGRIHLEVGFHKQVPRADV VEYLVSLVRRWKPCALVIDRKSPAMILVPELLAAK IEPETTTAPQMVTACTGFYNDATHGDLSHVGDEV LAAAIAGATQRNVTGGGWAWNRTTNAVISPLVA ATLARWGLMEFGALAAPPKQMPTQKSARTRSETD GLMAVGF

N1 301 _00 479	4666 95	46 79 51	+			intact	Portal_protein	portal protein;phage(gi589893334);PHAGE_Mycoba_32HC_NC_023602 5.61e-118 MESQTTPAPLTKPVQAKTVEIGYASESDSAFWASRETTPELLWPNSVRVYDQMRRQDAQCTSVLRAVMLPVRRTTWRLEPNGARPEVVTLIAEDLGLPIAGQEATSRRRARGRFSWGEHLQQALLMLPFGHMFFEQVYRIENRQYRLRKLAPRMPQTISDVKVARDGGLVGIEQYGLGHHIGTDMIPVERLVAYVNEREAGDWLGTSLLRSAYKNWLIKDRLLRVQAQTIDRNGMGVPVYQAGSRDEQEQLDRGEEIAQSYRSGNASGAALPYEAKLRLLGVEGNLPDAQPAIDYHDSQIGRSVLAHFLNLGQQTGSWALGSTFEDFFTMSLQTVAQSVADTANQHIVEDLVDANFGESEPAPLIVFDEIGSRKSATAEALKILVEAGILTPDEALERAARADYGLPAKTTSIEGARP
N1 301 _00 480	4679 48	46 91 95	+			intact	Head_protein	capsid maturation protease;phage(gi589893335);PHAGE_Mycoba_32HC_NC_023602 2.45e-79 MNRNRRRPRASAQQGRRDWYRIVNSADTETGAAEVMIYDEIGYWGVTAADFASDLKAIDSGTLTVHLNSPGGEVYDGIAILNALRNHKATVTVIVDGIAASAASFIAMAGDEIIMCRNAELMIHDSWGWCSGNAEEMTKAAAELDRVSDNIASIYAERAGGTAEEWRALMREETWYSAQEAVDAGLADRVAAKEAPDDGEDDTEASNRFDLSVFNYAGRRSAPAPRIPRRSNRADTRPGSQPRASDNPADESAAGTTTEGTATVEFTDEQLAALREALDLEDDATADDIVTAVEELATTPDPEEADPAESDDTAVVDRTSLARLQADAALGREAHARLARQDRERIVDSAIVTGRIAPAKRDTWISRLEADPTEASTLAKLEPVYPVGEPIGHDANPQAAGEDALYNKLFPTKEA

N1301_00481	469198	469563	+			intact	Phage-like_protein	scaffolding protein;phage(gi589893050);PHAGE_Mycoba_Donovan_NC_023552 3.31e-23 MADHTPDYLTGAAVTFLASADVTGGQLVAISGNESVAPTSAATAAWRGVAAFDAKAGDGVTVLSGGVHTLTSTGAIAAGAVVIAAAGGKVAAIGSGTDYSQVVGIAEAAAANNKVRVKLAR
N1301_00482	469590	470513	+			intact	Head_protein	capsid protein;phage(gi764161514);PHAGE_Mycoba_Malithi_NC_026605 2.20e-77 MSYTYPSPAPNINGDVLTISTFLKNPALVARRIRDLANERFIADAILTGRYEVVGGAIQYEQGESLYSDRPPKAIAPGGEYPLTSIGGGQSQIAKAEKWGQDSLVTDEAIVRQKMDPVNRGMTKQVNQMVKTVDSISLAAISSQVTQTIAATAAWDTSGSMILRDVLTTVAKITALNEGFVPDTLVVDDLTFAIVASDEKIAAMRAREDTSNPVYSGSFPIVGGLRMLPTPNLGMAKTAFVLDSTQLGGMADEKLGGPGYHSAGGVGVEAKTIRDEDNDQWKIRARRVTVPVVVEPKAAYKITGVLS
N1301_00483	470510	470875	+			intact	Head_protein	head-to-tail connector;phage(gi971482402);PHAGE_Mycoba_Brusacoram_NC_028747 3.55e-10 MTFVVTAALVVAAGADGTLGYHYQGQPINWLSAEDAQRLIADGMIEKATAAPTLAADAEETVTDGGDGANQTPTGAIERPAPTDPKPEWIAYGIAAGLDADAVEGMTKPQIAAAVDALNEQ
N1301_00484	470887	471237	+			intact	Head_protein	head-to-tail connector;phage(gi971482403);PHAGE_Mycoba_Brusacoram_NC_028747 4.46e-09 MPAFATPTEVIGTGRPTSEQENAQLLLDAAARWIRRKRPDIADDDQDARFVSIMVVRDALSAGAMRGHVSYSKGIGPWSKSGTLVNPDGALRWTDDHKEMLGIPVGTMPRGRFGDR
N1301_00485	471237	471566	+			intact	Hypothetical_protein	hypothetical protein N/A MAFGEKVTFKRGPTLDGNNNPIPGSGEVVAEECIVEWLGSADVTDLTRNGITDQARIFLTIRPDGEIRTSDTAMVRGQPFRVEGIADDWIDPEEPEFGGLVVTVKRGQG

N1 301 _00 486	4715 66	47 18 44	+				intact	Hypothetical_prote in	hypothetical protein N/A MAEPVFQLDHAGVQEILNWPTVAAMVNEVAEQIADNVRGQLDDDIPVEVDAYTNDRHAASVAIKHPGGKGFQLQRGVLTRAAGMVGLEVKSR
N1 301 _00 487	4718 41	47 22 39	+				intact	Hypothetical_prote in	hypothetical protein;phage(gi589893342);PHAGE_Mycoba_32HC_NC_023602 2.62e-13 MKARVQPRDATTPVKDFLTDQMVLIDPEITAALSLPSEWTPDDPPAVVVFDDSGPVNWPVSTSPQIRVTVWADGRDRARDIAGKCLGWLLCSKVPGITHIGPGVGLLDGRDEQNGGLTASFTVTTIVRTVSF
N1 301 _00 488	4722 89	47 31 13	+				intact	Tail_protein	major tail subunit;phage(gi589893343);PHAGE_Mycoba_32HC_NC_023602 3.56e-06 MSNAVINPNDSRIWDEAEVYVLFKEDAPNGITPFIPASVEDELDLKWLFTGLLDASAGIPFTPSLEITHFDGFGHPRYRSKAKKGSLDTGFTALEDNSVTRRLVLPGSAPNKIGKPKAVYAYVAYVLRDEDIITQCLISLEPALISVGAFTKGEGAQEMYAFTCAHPNDPNGDVFVRIESGAGPSEWLVTVPAGTTAGSFTLKVGNESVTGISYNAATAAVKSALESLSTVGTGKATVTGSAGGPFTVKLTDGGVLSSPAASLTPAGAVTVAAA
N1 301 _00 489	4731 85	47 34 93	+				intact	Hypothetical_prote in	hypothetical protein N/A MRTPNQHEIEQAAVELGYIKPGERVPPRLYSKVAKAVQLTADLPPEDDTDDAPAEDFVDDIVTTHTALIEGGLSDTAADRVVAAIAPAIWRETRGAAHAFPR
N1 301 _00 490	4734 77	47 40 01	+				intact	Hypothetical_prote in	hypothetical protein;phage(gi589893347);PHAGE_Mycoba_32HC_NC_023602 9.24e-09 MPSPDDFDAFEADTDDYADPTLDAGEDVDAESAEELRRRLAAAEARESRQDKPAGRPRGGRGGRGSRRSGSAVPANAPKPQDHKQKKTPAQREGEGDSIFVLEYNGTDYVVPADPEDWPTVAVQYLTANRAADALELILGPSQWATFNRENPRLRDFKRFSMELGRGMGISLGN

N1301_00491	474040	474405	+			intact	Hypothetical_protein	hypothetical protein;phage(gi589893346);PHAGE_Mycoba_32HC_NC_023602 7.45e-08 MEADLRRFYGVRLSDVWRRDERGHRLLTLREVFNYLRHLPRDSALAIDDNGGVMPWLPAESLLADLWELQANTGRKGKPKIEHSGRKKQHQKQLEHKASRKKSAFDRAKARDARRRAQAAS

N1301_00492	474415	478362	+			intact	Tail_protein	phage tail measure protein;phage(gi431810935);PHAGE_Erwini_ENT90_NC_019932 1.31e-51<br>MATSIGWASLQIAPVIPGIAKAVTQQIQGPLQQAG<br>QSAGQDFGDAVAQGVKKAEAAVVSATRKLAAAR<br>DKEADSQGKLNVAQAKLNELQDQGVTSGSRYVA<br>AVEALERAKRSHNKVSGAAEAAEVQLRNANDAV<br>TDAMHAEADAVNELSTAHDQLSKSSESASKAGDA<br>LHAGLGKIGGITAGAVAGVMGVGAALDGIGTAIE<br>KNDINSKLKVQLGLTAQEAGAAGAVAGDLYYKG<br>FGENYEQVNEAVAAVGSTIADFGTSSQEDIENLTT<br>SAMNMSDVFGVDVAESTAVAGQLIKDGLAGDAT<br>TAMDLMVGAMQQVPVAMQGEIFPVFSEYSKHFA<br>GLGLEGEQAFGLVVAAAQDGAIGMDKIGDGLKEF<br>QIRATDMSESTKEAFQSAGVNMEQMTADLLAGGP<br>AAGAAFNDIVAGLQNIQDPADQATAALALFGSPLE<br>DLGVNNIPQFLDQLSAGKEGLGEFEEAAHMMNED<br>MASGAGNAKEILERSMEGIYEALGPLMQPIAEVLG<br>EVTKLLQPMVETLGGALADAVQALLPALPPLAQT<br>FGALIEALAPLLPVIAEMVSGLIQALAPALTTIIEAL<br>TPVIETVADALMPVFEEISPILADVAAMIAEVLVTA<br>LDALAPYLPQLFEALGMVVQALLPLLPPLLELGMS<br>LMPLLTGAIQILAPIIVQVSEIFAGIVGILADMLIPVI<br>GVLTTVMTTIATFISDVFVGAFNLGKDAINGVIAVF<br>GWLKDKAGEVLGWVGEKISGFIGIVGDMGGKIKN<br>AASGMWDGIKDAFKGAINWILTKWNEFKLEIGGQ<br>KISIAGLDIDIPTITLNTPDIPLLASGGVAGRTKNGL<br>LWGPGTGRSDSILGIDAFGIPTARVSAGEGVVMES<br>AMRGGGDRVVAALNAGWTPSAEFLHGLTRGEFR<br>SNPFGIEEDSRLVAAALGARSLIADGDFTGNLHDA<br>FGIEEDHPVVGSILGLRELIRSLPAYADGGAVVTTG<br>ELAEFAKPIEGQTYDFGGWGNGWITDCSGAVAAL<br>ANFATGRLAAGTGQRFATGTEGQALAALGFKPGL<br>GPAGSLQVGWYNGGPWGGHTAATLPNGVNVEM<br>GGNRGNGQYGGPAAGASWGPFTDHAHLPMISQE<br>ALGALQTKVTPYPTSPQVARADVGASNPSSTPTGT<br>AEPQPEKAFSARDRWKTMFTDIAGVWADSSIEML<br>GVGEYLDLADRYTIKTDDGTPVTPAPGSGVEGIDG

								DANAVPWLMDVRNFLSSVGLFDTGGVWEPGTFG FNGLTQPEYVLKDAHWKVAEANIEKVDELVGAG VGVGRGGPVINQTFNTTVADQDGFHRRANRDRDL AMMQYGGRPF

N1301_00493	478362	479279	+			intact	Tail_protein	tail protein;phage(gi338826872);PHAGE_Gordon_GTE2_NC_015720 6.04e-54 MVDIGIIGANKDRIDVSGRLAGRQGIILAAGQVQGIHDAPVKTEWTSGRLEVGGRSKGASYEVRDLLLGFHLFGEDSDGGVTELSSRLRKMFTYERDPWDPDEQLAAMFCKTRRSLRKLHFQLYDPIANDPEFDPEIDEYENVIFPLRAGQPMWESRKDVTVWKTTQSSDSGFIEVWNPTDRPLLQTWVLTRGQWTISDTSWIGPKGKRVPGGEFGGRTLPLNPIDAVHGGARINLDPMKLMVESWSGTNLLGELAGQFFFMHEIPPYTPPTLLPIAVTGAPSGGARAELHMRRFWSRPWGLEWR
N1301_00494	479270	480973	+			intact	Tail_protein	minor tail protein;phage(gi530547105);PHAGE_Mycoba_Jabbawokkie_NC_022069 2.70e-156 MALTATPDFELTLEEQCEAIWAATEARQAAMDAIRAKPPLIRLWDGNRKLQFLIQNDDGFKFEDPDNETGPGEITLDFETPQAQWLNDMYGRKQRGEKIDVLISIDHCGGRWSGLLDRTIVEEDESGYTRLIARFHSDYEQLKWLQFWSSPMWPAAIQKRLFLLGGPLPWVILTSIFVNLWRKEGNLWHLPDDPLKLSSWLAGPKPLDQSNWSIVVKPLSFMDALASGAPMTLMSSRWKTAHEACADTLADGEYSLTWRRWFEGDPEPWPGAAGKIAHGALVIGCEDKSGVYEGTANGGTWMDGFTRTIRRFSDDLYEDTETAQSTAPVIGDYRIPGRLNTNRRAPYVFFHPKSPGVKFGSFTQSPAKGKVINTGGHSMPGVNETLSAAVQGIFDIIGNLFQIGSIGGSVDAIAKMFYEDTILAWISVKLLDRARDAGWFGYFEYFIDSGGKAYTLSSIMVIRSGRWATRSWSSSSMEIADGAPYLVGDQGQGHFWKGDRVSRLIKGDKSRRLYIDRVSKLTLSGGRGQRAGYTIEVGNGEAQMDPVVRLMQIAEKAKATAKELGVF

N1301_00495	480974	481408	+			intact	Tail_protein	minor tail protein;phage(gi530547106);PHAGE_Mycoba_Jabbawokkie_NC_022069 2.73e-13 MHSHLRQRLQQRIKEIDQDKIPTVDNCDPADPKEMAWPAFMSPPGMNESAFLMPAPYYSLMSWHLHDFGLRRVCDACGHMPEATIKLLHSRGGDPSMWTGAGIWVPADTPEPDTTELERAVDALSPGDRSAFYKELARRQREGL
N1301_00496	481405	482868	+			intact	Lysis_protein	lysin A;phage(gi764160952);PHAGE_Mycoba_Milly_NC_026598 8.22e-175 MTVKQLDYDRAIVRQETGYWCGPASAQVVLNSRGIHVEESVLAEEMGTTWNGTDFIGQIERVLDQRVPDARYTSVLMPNDPPTVEQCARLWRDIVRSIDAGWGVIVNIVAPSSNYPRAVAPSTISPAYSGGTVYHYMAVMGYDDERRSVWIADSGFAPFGYWLGFDQLATLIPPKGYAFADVEAAPEPAPAPAPAPRGMDVDAFAQAMGGTVSRERYTALLPAFTAAMREAGCTTVERAAMWCAQLGHESNGLLWMEELASGADYEGRRDLGNTQPGDGRRFKGRGPIQVTGRHNYTECSRWAHGRGLAPSPTYFVDNPDDLASDRYGFVGAVWYWTAARPQLNALADARNLDGATRAINGGLNGIADRSTRYQRCLDMGAALLPTGDTVTNVVEEGALQLRPHPGLLRKINRPQNVNESTRDPENAWPYAMWADLWNEAVWDGYDIRPEYADVPDDVGRSLVALVQTIAARQVRIEKKLDQLLGGK
N1301_00497	482868	483233	+			intact	Hypothetical_protein	hypothetical protein N/A MLNNPNTRKWVYVAQVVLGAALVVLVASGVVDQATSDQITSSLDRIVAGIGGLALLGGGELARRNLSPKPATISADGVQVITNALDAYSARAQPAIEKAESQLQKAAQDLREQYVDPFIRG
N1301_00498	483238	483543	+			intact	Hypothetical_protein	hypothetical protein N/A MLDSILKWLSPDTIQALGIAATAVLTAWTSRLAIRVKAAEARVAELEKQGEKDRGIIKAAAKFIRDQSVHIAILCGLLRHHVPDAEIPPEPKLPEILDKEM

N1301_00499	483547	484410	+			intact	Tail_protein	minor tail protein;phage(gi530545960);PHAGE_Mycoba_Velveteen_NC_022060 2.18e-05 MGLAPGSHPDGTLSGAGGIAAWAAESAEAFKSGLAGRIVTSSNGIKLFGDGVNFMRTRVDAHNLLIETQGEQIADLEEFAGMETTTPIWASTGGWDLVTFPDTMMNRVRYFDGTSTTLSDIPVFSQAKRTLDMAFLRGGRDTPTPLEVVRIITGADTNLFDIDAWYVGIYVYDKNNDRMQLLWNSGNLLNVLTGQRMRYHLGTGMTQAAANDQLLAVATLQIAPGLVQKPRGIGCIFQTGVKEQAGTIPQARHAYLNNVDALPVSISMSSLNYDSSKLMWAAVGASS
N1301_00500	484407	484706	+			intact	Hypothetical_protein	hypothetical protein N/A MIDNGRLSITAAELADFTSLAEIEPVARHWWLINTTTGQTRIERDPSALPDSGDWLLLMTGPTPAWVQQWDGDLQRAVDEQLNPLLEAAMNYSTGEVTP
N1301_00501	484703	485032	+			intact	Hypothetical_protein	hypothetical protein N/A MTLGWSPIDETIELTNGDWIFERTNEAGAIPPDTVIEIKWANGVTWPGEIHGATVRWRVESTACTAALIPDGTAFEIFVRYPNDATSTTDDYVWMGGRALRTDFTRQNF
N1301_00502	485041	485385	+			intact	Tail_protein	minor tail protein;phage(gi764159236);PHAGE_Mycoba_Minerva_NC_026584 1.36e-06 MANTVLATANALANHWASLGNSYSLHIGNPGAAGTANEASGNGYTRQATTFGSATSGVVTGSQMTFKFQGTATHMCRWNGSTLLDIIDTVDATVTPAGEIKVTPSFNANYVAWT
N1301_00503	485388	485978	+			intact	Hypothetical_protein	hypothetical protein N/A MTAVEFPVADRGIAALPTVDRAIVALPSARGVAVPFPRPPAPVREAVSSMDGLAAASLIVVPVRAVGVSMEGAAVAAVGVVAMVDAAVVMDSQPYAHRAIDASASMNGTASATLSLAVAVGAAAGMDGAAGIVGEAVAIVVATVVTSMDASASVAIEGFAAAPASMDASASAVVKIPPRYPSSGLFPATTLYPEEE

N1301_00504	485979	486428	+			intact	Hypothetical_protein	hypothetical protein;phage(gi372449849);PHAGE_Rhodoc_REQ2_NC_016652 1.20e-28 MPPYTRVNWQDGEEGGTPMSAANFNRMDKGIADLDAEAYRKPAPGIPGVDIEPGAVGATQLTAEVLVVFMFAALLMQAATQDGELVGLPRGFLAQDVQDDLGKAATAVQGSKNGTPAALKVWVGTEAQYNAATNNGANEAANTIYLRGA
N1301_00505	486428	487312	+			intact	Hypothetical_protein	hypothetical protein;phage(gi372449850);PHAGE_Rhodoc_REQ2_NC_016652 2.15e-179 MAGKISFPGVGQIKKVGACGMAVKKISSGGAVLWSSSIPRQGVLKSGTQQLSQNNYEKVTGFKFDPAFPETDVAAAQAANGLLVSGRSAFVAEGTMATSSTANQNRGVQVRGNGAVLGTTDGTSATDSRTAKVVVPDGTDVLLELYASAGSSITSYRVLAADTTKLSYRTGGYYEQLTPVTLLQNQITEATLTANAATELPRVPGSGIFLEAGTYELIWNLYSVGWGVNWSVGCRVGAEQNQVIAGSANSNSWNRPVQTITVPSAQFVMPTVRASSSSDLTIDAGRLNLFISKI
N1301_00506	487845	488141	-			intact	Hypothetical_protein	hypothetical protein;phage(gi372449851);PHAGE_Rhodoc_REQ2_NC_016652 4.40e-07 MSRPAPAWVPWAVLAVAAIGMVGCVANATYAAAILWAAIAACSAAVLARRRMRQVRLERAEQAAHDAAIAARADAQNEAYLDGDTTGLYGKYRPTDPI
N1301_00507	488138	488377	-			intact	Phage-like_protein	putative HTH binding protein;phage(gi589893331);PHAGE_Mycoba_32HC_NC_023602 5.70e-32 MPDPMHYLSANQVAERIGVKPPSLSRYKLPPADAITGPVNEDGSLPRGTTRGWLPATIDEWNARRPGRGARTDLQGGES

N1301_01631	1709744	1710934	-			intact	Integrase	integrase;phage(gi764159278);PHAGE_Mycoba_Minerva_NC_026584 8.14e-30 MARVRDLWMSKDSRGKKTIRTARYGKGNRWLAVWEESGREVSKTFSTQDAAESAIANATVAKENGTYIAKAKRDITVGEVWPIWWATKAGKSKSTRDGYAAAWKHIEPKWGSVPCSQITRAGAAAWLPTLTSRYKDADGTPKPLGESSLRVVMIVLHGVLGAAVEERIIPANPIRLADAPRQRETSRRYLTVVEVDLLRAAMPHAQARLVVDTLVRTGVRPGEAFGFQVGDLDGMRGRLRVARDVDAQGGADDTKTGRHRDVPAGGDLLLDLENAAEGRARDEWLLTMPDGRGWTLPRWRQLWAAATVEAGLEGLDTYELRHTAASLAIHSGANVKTVQRMLGHRSAAMTLDIYGHLWDEELDTLPTQMDAHMKAERARFEARRRRAETELRRNIG
N1301_01632	1710971	1711867	-			intact	Hypothetical_protein	hypothetical protein N/A MSKFTDMRHQIDGSSWLDNLARASLVAADAKFGDGGRIPILAVDFAEDVDATSLSIVTESVQNAVARMGKYISNSSSAATQLRETDRQLTRIFPRRQFGRRIEFSFEPPLEAEGRLFDIPTVESVAEIAARELIQVLPGTADDQQAVAAVFARDRAPINAVRDIALAVERTSGIGMILTTNFGDEFGSIVTSEQARRITERLNDSKDLERETIKVEGRIDGMRTRRRIFYLESTSRDYEGAFDSGMANAVKTHIDRPVVATLERVRPLKKAGPRGRWSYHLKGLEEPQSEETLFGPTD
N1301_01633	1711864	1712175	-			intact	Hypothetical_protein	hypothetical protein N/A MTNHGGGDCDASVLILPDDWGRVLDLDEARQAQLYSLLTLQEIVVGSPLYLGLDNIRPVASMLDGFLCYPGHEDRWHDTWATVKGFDGQVIDGIEKGYVEVSL
N1301_01634	1713046	1713291	+			intact	Phage-like_protein	CRO protein;phage(gi530547144);PHAGE_Mycoba_Jabbawokkie_NC_022069 3.37e-05 MSSATLRVRPGVLKRIRELHRLPSDAALAHAIGVSRATLDRINRGEVAPSPHFIAGLCLFTGIGIGEAFEVLATPGGRNAA

N1301_01635	1713391	1714179	+			intact	Hypothetical_protein	hypothetical protein;phage(gi418487673);PHAGE_Escher_TL_2011b_NC_019445 7.50e-16 MNNDSATLSPVAIPGASMPIQVDPSRELAALRPMVEHFGLDFRTQLRKLKGKSWATVVNMTTVGADGKTREMVGIDRRTTGMWLATLEENRVADEKRADLRAYQAEAADALDAYFHDGGVINPRATEDQLDRLSRQAQAQASVIQALRGVVDPKHLEAKGRIVLARAMGETPELDPAGIPLYVSDFLKSKGLASDQVAAKASGFGKRLKGLYVAEHGDAPRKAFQELPNGTVREVFAYTQADRGLFEQVWSRHYEGVTKAAA
N1301_01636	1714370	1714741	+			intact	Hypothetical_protein	hypothetical protein N/A MKRYIDGSLITLLDHVPPSGQARALIDHHSSIADEFVNQIAAEELLAADAVQASTSRVGPRSTSADAVRVDYQTDGRFRLVRSGLVWTVEEEAAPGTFVQRGGHYQSRGAAASYIDTIVEAGR
N1301_01637	1714738	1715043	+			intact	Hypothetical_protein	hypothetical protein;phage(gi530546023);PHAGE_Mycoba_Velveteen_NC_022060 1.04e-05 MTELIRQYAIRLPDGNLLAEPEHAPTWRRLLSSAPEPRTAVFDDEDAAARALEQMRETARLFGVTNLGAYVVPRVVAVGPWGDVDITPSIDAVERHANGAT
N1301_01638	1715148	1715423	+			intact	Hypothetical_protein	hypothetical protein N/A MFGFTHRTPATPADVSDTANAWPDEIAGEFAADPDGAILATAEAGRAGIDRAQRHRREAWLAVIVLGIALGGIAWAGATAAISSPLAGLTA
N1301_01639	1715420	1715668	+			intact	Hypothetical_protein	hypothetical protein N/A MTASTDPRLPTTPESSWKACSLHEFRRHTGKRVVYLSVATGKPAETGIVSHVDDRWVYVHYSGSQGPLATHPDNLRLTGERR
N1301_01640	1715665	1715823	+			intact	Hypothetical_protein	hypothetical protein N/A MSDSGIDDIERDVLAETRLRSAHALLIEQRRANAHAIAAGAPVIDFEETTCD

N1301_01641	1715816	1716811	+			intact	Hypothetical_protein	hypothetical protein;phage(gi530547039);PHAGE_Mycoba_Daenerys_NC_022068 9.08e-79 MTELQTRKPTGRPPFPLILLEGPSKSGKTYAAAELTASERIGRAFWLDLGEGSADEYGAIEGADFEIVVHNGSWPSIFAAVEQVHAVAARAAAAGEPPVMLVFDSMTTEWEMIKDWVNRRARQTKAALKILQDDPDADIKAGSLWNDGADRHGQLMRLLKTFPGIAVVTAIGTETVALDKDGRPIPGERDYKIEAHKTLTRDVSAWVRYSHDTGPRVVGVRSPRAGYRPGVDRLEMRAEFTLEWLVFDLLGCGSDTTPRDLVAPLSLVDLEGFALAADSAEWVNETWAMAKDAAMLDVALPGGSTARELLTEAAGRIKAASGQPAARGGAQ
N1301_01642	1716808	1717941	+			intact	Phage-like_protein	gp51;phage(gi593774681);PHAGE_Mycoba_BigNuz_NC_023692 7.66e-55 MSITIDTKSLVEILTDLTLTATGHRGVHLRTTRGHYGDEPEETTLLVGTSTNGAVLGHTWTRCTGELPPMIWPLMNCKVIIGALRSLAKNDKNHAVDVVLDGKTVTVVEAATLFDDGDRFEFQVAEIGAFPLAKIHRILSGEPLPIPRNDTGDDMPDTARTTWSPEHLAALLKIATRRARTLHLFRRHSNQIHLAQIGDNWVGAITPLSAWDHDDGDRPNTDVYVEAPEQLDLSWLRSYSGGAFAGNRDVEDDDPAEPTDSASAPEQPTLDDPGPDDAADASAPAKDDELDVEKLVAAGEDDLLRDAAQMVLATKFGSASMLQRRLRIGFAEATRLLDELSALGIVSAADGSKARGVLVDEAGMRAALEQRGDRGTP
N1301_01643	1717991	1718605	+			intact	Hypothetical_protein	hypothetical protein N/A MAWQLVKEVLDGAPPSLKAPERLVLVAIAEWCDVETRTCWRLNEELRLRVNMTASGLRAAFARLAAVGADPRVPIAFAADGAPVYAYKGRTTTFQLPYMTAVDGHEGDTTATPSEGDTTASPSAGEGDTTAAPLNTPAPVSNSSTGDTAVAALADAKATPQHAKATPQRIKGDTTVAPSPSKTKRNQFFPREQAPSYPQLRAIN

N1301_01644	1718618	17191003	+			intact	Phage-like_protein	single stranded DNA binding protein;phage(gi372449997);PHAGE_Rhodoc_REQ3_NC_016654 1.47e-72 MAGDTVITIIGNLTADPELRFTPAGAAVANFTVASTPRIFDRQSNQWKDDDALFLRCSIWRESAENVAETLTRGMRVIVSGRLTQRSYETRDGEKRTVVELQVDEIGPSLRYATAKVNKVSRSGNGSGGGYAGNQDRERPPADDPWGNASQAGSYDDEPPF
N1301_01645	1719121	17196642	+			intact	Hypothetical_protein	hypothetical protein N/A MQSTTDTVVLGIDPGGRSTGLCLITGREVVLHQVVVNDADKMLPIELTYVHEVLGAMATIVERTALVVDVTIAVEDVVRPSWHMKGRAAADPSALLATAQILGAVLAFDPTMRTVLVRPRGNGSRALGEYPTALVSAAERRKDGWQMRIGTGQLRHARSAYDVALAGQEAIHA
N1301_01646	1719639	17199002	+			intact	Hypothetical_protein	hypothetical protein;phage(gi966198577);PHAGE_Gordon_GTE6_NC_028665 1.62e-06 MSAELPEPSRCRSCHAEIHWGKTPAGKHLPVDATPAKSGTVALDVHGGVLYAGVLVGAQLAAVRRSTRPLYEPHWVNCPDAKAWRNR
N1301_01647	1719899	17202100	+			intact	Hypothetical_protein	hypothetical protein N/A MRDTPQRPFAHLFPVHTVVIARTLLDGQKFLRARQAPVENPLIVTHPGQLDGGVRIRKDLVLCTPAAHSNPRFQEIAARLQAAIRGALAIANARANNKIGDQA
N1301_01648	1720207	17209771	+			intact	Lysis_protein	lysin B;phage(gi971762031);PHAGE_Gordon_Gmala1_NC_028972 1.99e-43 MITVLACRGIGEPLQGNMCSAVTRRLDPKRFRVIEVPWTASYGPVPLPLGPAFDDSLRTGRELLLRMIDEDPNPVLLLGYSGGAALAGNVAAEVARGEHPRLEVVGCGLISDPFMPQYASPTRGAWGIAGYRHIALSVPTLWAADPTDAICCCPANSPLRTIADQTRAFSLSNLLGWGIDLVGRLKRQEWQQVAIDWRNRDEVHAIYRQAAHDLDGYLRRGDHTSYALRIKPRTSTTYTDWLADQINTLGRELA

N1301_01649	1720968	1721363	+			intact	Hypothetical_protein	hypothetical protein N/A MTEQELNALVASLASLEAGASSELWTYSEDRRTIEGLAGIPIALGIARATDGYLAVSARNALPSLLGALQAARMERAENVARADRLQERVARLTEKVQAWCPHLSTSGCITGDERCDECGLVDPHGEAGDR
N1301_01650	1721575	1722927	+			intact	Phage-like_protein	gp72;phage(gi593775761);PHAGE_Mycoba_Dori_NC_023703 0.0 MTLTMTDLFCGAGGSSTGALSVPGVSVRMAANHWPLAIETHNTNHPTTDHACADISQVDPRRFPRTDILWASPECTNHSVAKGRKRNLDSTPDLFGEILPDEAADRSRATMWDVPRFAEHHKYRAIITENVVDAAKWIMFPAWLQAMDLLRYDHHIVYMNSMHAQAAGDPAPQSRDRMYVVFWLRGNRGPDLDRWTRPRAYCAHCDEVVSAVQAWKKPTRWGRYRAQYVWRCPRVSCRNAIVEPGWLPAAAAIDWTIQGERIGDRARPLAPKTLARIEAGLRKFAGPFTAEVAGNTFERRPGVRTWSTDDVLKTLHTTPSKALLVPVEGRDGKDARSAADPLRTMTTRNETAFVVPLRANNTPKPVAEPYDTFAANGNHHALAQAETVDVDDCLFRMLEPHEIAAGMAFPRDYRILGTKREQVRQAGNAVCPPNARDLIAAVAESLGVAA
N1301_01651	1722924	1723355	+			intact	Hypothetical_protein	hypothetical protein;phage(gi372449897);PHAGE_Rhodoc_REQ2_NC_016652 2.03e-58 MTEIPYDEMRRILGLPEPPFNAGGLWNTQPAAARGTFPHAVPRSGGKAPQYVQQLSHPFTEAEKLAIAEGFRHLGLVIQDWQLRFIEHMAEITKAARHIVETVESLTPKDQRPHPMHPAIPRPHHTPPMWAPNPTRTHRRRNR
N1301_01652	1723352	1723546	+			intact	Hypothetical_protein	hypothetical protein N/A MTRYRIRKTGRRWLIVTPSGHRTYRNTWPGAMRLVDRFIVAKRAQLARTTQSDYALAAPGGVNG

N1301_01653	1723539	1724204	+			intact	Hypothetical_protein	hypothetical protein;phage(gi372450001);PHAGE_Rhodoc_REQ3_NC_016654 2.03e-17 MADQHNLNRSEISLLVDRLDLIVEWVAAELENAITCQTAFTNKTIARVGKDEEVPLAFDEKASRCAYDLLGTLRTWTNYVATEHGLPWPGDGRSPHFARWLSRHAFHLARTDDAALAYRQIIDAYNAAVSVVDRPAEKPPRQIDESKWAEARRLNLNARACVQMAHTMGAEYTDLTQRRVKYLADEQVIAPVWIAKAGKRSERVFILGDVLDAHLKHPKNA
N1301_01654	1724652	1725302	-			intact	Hypothetical_protein	hypothetical protein N/A MQLREAESRFEEVKKGHEAEIATLREVFEAVASDAIGNERLLLTGQDFPLQESVLRALRFLGYDVQDMDGVFPDREPREDYRIRDPDAPDWIAIGDATGVAKGAKTAKIGALGGFSLKYVLDEGATSPPRQWLLVNRKIHQDPNTRGEVYRDDDVMAFAASLGLAIDTVALFLLVRAVSDATVSAAEVRTWLRDRTGQLLISDVREWLPERGGTAD
N1301_01655	1726442	1726675	+			intact	Hypothetical_protein	hypothetical protein;phage(gi764161273);PHAGE_Mycoba_Keshu_NC_026603 3.92e-09 MTPTIGRIVHFTESSGRTIAAVVTAVHPDHCVELTVFQPRAAPGWITRGPKGTPDETPLIPFAEQPTPGHWNWPPRA
N1301_01656	1726786	1727124	+			intact	Terminase	terminase small subunit;phage(gi589893046);PHAGE_Mycoba_Donovan_NC_023552 2.68e-23 MREDLADGGQQLLDELTQDDDPFEMTALIVEAARMKDRLDCLHQILIGDEELWLRLIPSRGDTKVLEIRVDSAVQEARQLATVFRQMLAEIERRRAGDGDSDGGEGDDLAGL

N1301_01657	1727075	1728637	+			intact	Terminase	terminase large subunit;phage(gi372449823);PHAGE_Rhodoc_REQ2_NC_016652 1.26e-117 MTATPTVARATTSLDSDWPTLTGRQTPHALSVFEGDTTLGEKASELSRRIGSKPMPWQRDTLHALLTRSADGTWTHPDACIICPRQNGKSLILTLRILYGLFKLGETIIFTAQRWTTAEDIYLRTWALIEPRKSLRERVLKHTCSQGRGVIEMKNGNKVVFTTRSADAGRGLTKLDLVIYDEAYNLTDSEMSALGPAQLAAEDPQTIYTSSAVNQDEHSNGLVLSKIRDRGLAGDAELYYAEYMAPDGMDREDEETWRYANPSYGVIQTAKKIRKLMRGFATQAGRKSFDVEILGRGDWPKEVDAASMTWQIVKEGQWHGLVDYPELVGTRAVGVARVGAQWVVAAAQRTDDDRIHVEVGYLRIAANPDIVDLIVRLDDVLEPCAIATDARSPAVVIEPLLKKAGIELIKSSTSQTALMGSGFVDDADDGLISHTGQPALDVALEAAGKRLLPRGDWVVDAAGDPAVAPLLAVVVARWALVTFEARATGPAAMPAWDGQEATGGQPSGPSDDFDALAVAF
N1301_01658	1728649	1729920	+			intact	Portal_protein	portal protein;phage(gi372449824);PHAGE_Rhodoc_REQ2_NC_016652 3.93e-178 MSDTPLYAHQIEVGYVNGRADGYAQWDETERVPELQWPNSVNVFRRMMSEDGRVSSVLEAISLPIIRSQWRVDPNGASDEVTAFVASNLGLPVLGQSQAPASRTRGRFSWSDHLQDALQMLVFGHSFFEQVYRRGDDGRFHLRKLAPRPQETISRINVAPDGGLVSIEQMAPASGATVTRSSSVSIPVSNLVAYVRKRRSGVWTGQSILRPAYKHWLLKDEFMRIQAATARRNGMGVPVGTAAKADDPAEVEQMHRIASAFRGGMNSGIGLANGQTLQLLGVQGNLPDMHQAIEYHDKQIALAGLAHFLNLDRGGSYNLASVLNDTFVQSVQTIGEQVRDVANAHIVEDLVDLNFGRDALAPRIVFDEIGSRQDATAASLALFVQAGLLTPDEAVKIAVRQQLGLPDGTPPEFTDPNEAGGDV

N1301_01659	1729917	1731338	+			intact	Head_protein	capsid maturation protease;phage(gi971744026);PHAGE_Mycoba_Phayonce_NC_028796 1.30e-06 MTDLEPVTVPALSRLPNVELMHAGTWSASTGVHTFTVDDLAFAVAALDCPAVRRPILKLGHTDPRFDGEPAVGWIDHLAVASDGRTLQGDYVGMPGWLGPVLASAYPDRSVEGQWDYRCAVGHEHPFVLTAVALLGVEHPAIGTLESLQDVAQLYGVAASAGDRTGAVSIHLKGGTMPNPKARSVAMGVTAEDVRRAYYDDAPWSVWIEEIQLDPLQLIVIDDDTGGRLRVPVAVSGDGTEGVTFGEAVPVVVRYEDADGGSDAEPEPEGEAVAASRIRFACRAESRPGDSPRASRSRVAAAAGPTEGGSTVEITDDQLTTLREALGLAEDADLDAIITAVEELATAPGNDSETNGEDTPASVAAKAKKFGLSVMDAATLEARLARGDAAYAQLQREKCERVVDAALSKGKIAPASRAVYLKLMDKDPDGTEKFLAELPDESVVNLSEKGHGVGSEVNASKVTDDPQYKKWSL
N1301_01660	1731340	1731780	+			intact	Hypothetical_protein	hypothetical protein;phage(gi372449826);PHAGE_Rhodoc_REQ2_NC_016652 2.13e-53 MAGIPQVTKTGPKTFTPAEVVLGGQLVEARAAGRIGVAAAGSVKVLGVALTDAQSPDAPQGGTTTDAIGRPIANAMGIPTCVAVAYGPVEVQVGYAAAANFGDRLIAAAGGKVTPAAVDADARTIVGICTEPAGVAANKSGLVRLA
N1301_01661	1731808	1732749	+			intact	Head_protein	putative main capsid protein;phage(gi372449827);PHAGE_Rhodoc_REQ2_NC_016652 1.66e-137 MTTNLVSVTDGGGITVSDLVANPMFVPTKLKELMENQFISEALFRNAGSNKSSIVKFTEGDPTFLEGDVQDVAEFGEIPVSHGRRGEPRIAIATKRALGVRISKEMQDENDIDAANKQMTGLRNTFVRANDRAAKAVLMSSAVPTMPVSKAWDDVASKPRTDIVNAVDEISNAAPNEAQGGSEDEYFGFEPDTIVVNTGLLATLMDNEDILKVYQGNIADQNIAYTGALPAKIYGLNVIQSRTFPRDRALILQRGVAGFYSDSRPLTFTALYPEGGGPNGGPTETWRSDASIKRAIGLDQPKAALWLTGLVTP

N1301_01662	1732746	1733135	+			intact	Hypothetical_protein	hypothetical protein;phage(gi372449828);PHAGE_Rhodoc_REQ2_NC_016652 4.58e-37 MTEYILTALRFDQILEQDDKGRVIKKIRHRQGALITDLDDLEAQRLLGAGAIRPAGIADDEQDEEAEASEPSAATPKSPADPAADAPRRPRATATIEKWIAHAQAVGVNVDGLADKDAVIAAVDRLAEE
N1301_01663	1733150	1733518	+			intact	Head_protein	head-to-tail connector;phage(gi971482403);PHAGE_Mycoba_Brusacoram_NC_028747 2.05e-09 MPEYATADEVARGRRPLTADQRLDVEALIAAAGKWIRDRRTDCGKPAIPVDDPTAKLVVLQVVRTALGTEEYAGHISYSRVVGGLSESGTLANPGEFLMFTDFHHQLLGIPTTAMPRATFGD
N1301_01664	1733520	1733867	+			intact	Phage-like_protein	gp9;phage(gi157168382);PHAGE_Coryne_BFK20_NC_009799 8.76e-26 MLFTDKITILRPKSGSSRYSTETVPDYSNVERIPVEELVSVQPASATEQGENRASTITGWRLTTPAGVNLDLLAIDQIEHAGRVLSVIGEPLRWPHPTRPGDVHHLEAALQVVSG
N1301_01665	1733867	1734145	+			intact	Hypothetical_protein	hypothetical protein N/A MAAPYRLSRKQFEAIMRNPQVQQRLKDVGRGVMSRAQVITNDEGGSAQISLESGIRPGGRAFTNVTSSSAAEEYGNSKTTRRRALGRAVRER
N1301_01666	1734150	1734350	+			intact	Hypothetical_protein	hypothetical protein N/A MKIKHTAHRRNSMIGKTEDLPDDEAYRLIQAGFAVAADVAPAAVAAHSFAPAGDALKATPVKPAKS
N1301_01667	1734354	1734731	+			intact	Hypothetical_protein	hypothetical protein N/A MASLDVEALLVQFLGGFGLRVFGGDLPPRFEESGPLPVVRVIALPGDELARAWNGPRLADRAEIDVDIFGADDDQAADTAARVQSLVEQLALDGIAVLRAPAFTRRPDWNDKVRRRGAVLTLATR

N1 301 _01 668	1734 756	17 35 34 9	+			intact	Tail_protein	gp12, major tail protein;phage(gi157168385);PHAGE_Coryne_BFK20_NC_009799 8.00e-31 MSQPLSNYDNQLVRLGVTGAVRRGPLGLVLPVHMAPWPVGAVDLGWISDEGITENREGDVAKFTPWQTNSPVRAETTSEEISWEFTMWTTSYDTVSAYFKVKAEDMEYDEESGVTSFVDGDKKPRDLGVWGFDVIDGVYARRVLAPQAEITARGSQVYKKDELIGMPVTLTAFPGPEGWSVKREFREGWRPPVTSGP
N1 301 _01 669	1735 472	17 35 91 5	+			intact	Phage-like_protein	gp13;phage(gi157168386);PHAGE_Coryne_BFK20_NC_009799 6.39e-13 MTDNVIPENAIDFDAILAKRKEEVGSPDRFPLVFAGQTWWVMDPTLADDDWTEDLRDLGYEKDDDGNLVEDEEGNLVELDSVNTIALAEHYLGEKQYEKFAAAGGRSAYVLQALKIHLERQSDKDAEGRPTQRSRSSRATRRRQKRR
N1 301 _01 670	1736 032	17 36 30 1	+			intact	Hypothetical_protein	hypothetical protein N/A MDGHAWGNNEALLWLISHKLGVLDQRLVWHRGKRPRWPDFKQFPWLKDTTKLGDRGKATVTQVMDYLRAIGPGGKGLPDDGDITTVIDH

N1 301 _01 671	1736 357	17 41 52 8	+			intact	Phage-like_protein	tape measure protein;phage(gi372449839);PHAGE_Rhodoc_REQ2_NC_016652 0.0 MSEDVIWVPVLPSLRDFARQLGTEGRRAGQEAGQQTAQAMAEAVSKGQAAVAKAVADIEKLRNREADATGKARVAQEELNKLLQAGETDTVKLTRAREKLAAAERKQTEAQSAAAAAVDKHQAAQRDLESTMQRASQATEGGSSALSGFTDKASAGVGKLKNFAIAAAGIGTAVEMAMQAIENIDLKNTLAASLGATGSLAAEYGGMAGDLWKQGVAGSMEQASEAVAAVASTFKVAGSEGERAVDDIAAAALNFSTIFGTDVNETVQTANQLITNGLAKDSTEAFDLMTAAWQRTGTGMRDELGELMNEYGTFFSSLGFSGQEAFGMLVNASDRGKIAMDKVGDALKEFGIRATDIGDQAAMDALTGLGLNANDTANALLAGGDAAKTAFGNIVSGLLKIDDPAKQASAALAVIGTPMEDLNKVELPAFLQAMSGAGDAMAGFEGSAQQAADTLNSGPGAAIRKLKNTIQGGITDAFGVAAQATLGAVNLISSGVGTVLGPAIDTAKLFIGTITGSGADVDIPWMNTVIDAGTRVRGVIDGVRTSVSGVWEFITTGNIGEDMARLFNFDLPILGKLEDLRNLAVDVVNEVIGGFRAMVAAFTDGGTDATSSGLAGYLERVGLVARGLWDGFTWGLGILREVWGFVSATFGPIFSWLAGIVLDLTVGALQGLWDGLQRGYDVINTVIDAVMSVVDWFTRHKDVAIAIAGVIGAMLLPALASMVFELGLTAIAWGIVAAQTAASTIATTAHTVATKAANAASKAWAAGVWLVNAALSANPIVLVIGAIAALVAGIVLAYRHSETFRNIVQAAWEGIQTAAGIAWDFLKGVFAWFGDAFTWIGEKATWLWQNVISPVFEFIGNAARVLMAVIGTVLIAPFLIGWNVLSAAIQYAWNNLIKPVFNLWAGVATWLWTSVLQPVWGAMQVGLQALGAFFGWVWNTLIKPAWDGLGAGISWVWENVIRPAWDGLKVALQAVGAFFGWVWNTLIKPAWDGLGAGISWVWENVIRPAWDGLKVALQAIGDFFGWVWNTLIKPAWDGLGAGISWVWDNVIRPVFEGIQGGLGAVRTAFDEAVKFIERVWEGIRGAVAKPIKFVIDSVYNNGIRAAWNKVAGFVGLDELPEYKPEWLGAYASGTSVLPGYSPGVDNMRFVSTDGRAAIDLGGGEAILRPEVTKAVGPQWVDGVNAAAAAGG

								TGAVQRYLGGFAGGGIVESIIAIAREHFPGLSVTSSF<br>RDSNDLHGQGKAVDLSNQVAGGPSTPLMQDAAR<br>FFYEGYGPQLAELIHWPLNGWENIDEGQPFDFGPG<br>TNAEHTDHVHVAAHQPLGAPDGDGGNWLSRTWG<br>SVKRGLRSMVEKLFDSVIDPIGNAIPDFGGSTIGQF<br>PRKVFDTLKDKAKEFLLGKADEQGSGPAGGGAEQ<br>WRQMMIEAYRNQGYDPTPAKIDAWMRQIATESG<br>GDPNIAQQIVDVNGTGEAAGVGLGQMIPTTWAAY<br>RDPSLPDDRRDPWAMLNAMVRYGEQKYGADLLD<br>VIGQGHGYDQGGIADGTGLMPKKILKPERVLSPAQ<br>TAAWEALVPHLIDVEAAMEAILQLAGAGDFTPQL<br>RQLIGSEEDSAVVDAVLSTRDGVLATQDAIDGLIA<br>DLESAYADVPQSALEEHSREALDFFGLGKWGDLL<br>FADQPQSVAVEPDPVADGLTDPAAADTVTESDRH<br>ENPPPAAEPGPRAPLVNIEEALAFDLNELVEMLTRE<br>LRHVVLSDGMNGGWDG

N1301_01672	1741521	1742393	+			intact	Tail_protein	minor tail protein;phage(gi372449747);PHAGE_Rhodoc_RGL3_NC_016650 8.92e-09 MADGLIPGGMTIVYYVSPDGVVIHLSGGTLAGTEGLVLGDGPEGLGHAEAAAIFDDAARMVGEEYVATTYSKGSIDLPIHVFGRTIDEFHHKRDWLRELISRERQGWLCVFTTLGWRMLAVRRGAIKPSLKRDPAGATGTTLFVLFYADNPLARAADGDVREWENKSGLIANGHRALYPGREVDGWPKFTFTGPGLLRLRYDGVDGPVDLKFPVLYAGEVLKIDTEYGMQRLRAQAANGTERNLWPLMGGSMEPQPIPAGVVTTVKFSVTGGSSATRLWATVPRCQEGLL
N1301_01673	1742390	1743829	+			intact	Tail_protein	minor tail protein;phage(gi530545820);PHAGE_Mycoba_DrDrey_NC_022059 1.45e-30 MSRKLYSRMAQADEDERRAYAHPRARIRFLTKQLEMWGPCGDHQGLKFKHKKLTPGSLSILVPENEHWVEYFYGQPKYAMRPIVVDLPGYRTFWLTVKFGRVRRRGKRWIQVEAVHGLKYFDHIHMWPAWEAPAWLQPESTTGIGHPITVLKGELGGQLFRLQAPGRWVPRFNLFNMSTWRRNREWFWPLMINPRNRNPKSSKWVITDARMDKFSDVMVEVCKTENIVPTVDLYIHGEDEQPFPEFIELDRNTLIVDFIEKDPEKPVARYAVGKWSPASEVEQLVHMPMATRITGGGKSPNWLNDLVVNTGNFIAGAIGVAFGVAGLSLGVLTDQLKNKFLAFETWEDRKLAEEAGPLRFHEAFVSGASTTLSLDLAAAMKSTAFDHRGYTSQKIVVANGADGNYIGRDLEMGDQVEYELPDGSIEVDSLEEINYEESPKRIGFELQIGSGDAEREPGSLALAKLRKFTSTLTRAVLGG

N1301_01674	1743866	1744990	+			intact	Phage-like_protein	lysin;phage(gi372450115);PHAGE_Rhodoc_REQ1_NC_016655 7.40e-84 MTVTLPPRPAFDEKWMLSQSRSSRFGAAVTNALWHTEEGNATAVALALYCGNPANNASYHDIVRDRIVAHVVDDDFASWSALAANPYTYNLCFAGSRAAWTEAQWMARADDIRIAVWLTLEVARRKGTIATEIIALGGGRYRGRMSGIADHYYVTKVLGIGNHTDLGMNFPWWFAAQVLAEYLAPAPLPAPVVPAIDEEIRRIREVNDWLGARITVGENDCRGDRGGKWVKLENGQAYWSAQTGAKIVPNALLDTYGLYDWEVGPLGYPIGDHTVLTEPSTGTPWGDVQHFEGGTLYRRYSADRGWWVHGLIYATWRRSGFENGPLGWPTSDEVELDNGDRVQHFEGGDIYWSPTGTVALKPADGPDQHFPISH
N1301_01675	1745004	1745453	+			intact	Phage-like_protein	gp17;phage(gi222435687);PHAGE_Mycoba_Phlyer_NC_012027 1.42e-20 MLDYLRRLIPATSRERIYTYAVAIVMLLVGLGYLSDTVAALWIAVLGASVTLIFALLHSLSPWRTALYGLLAAVAPLMLWFSIGTQQGWAAALGFAGVLFGITKAATNTRVLGLVVDGDVASHHLQDVDEALNAVDGASSVGQHARNEE
N1301_01676	1745458	1745868	+			intact	Hypothetical_protein	hypothetical protein N/A MEQVNTHPAVVLAMVVVAVAVVLIAGGKKIAGMVSPGWAWFQARHERRFQRQVRIEAAARFLNDERVEILLKRIDGLHAEIVAQREELTAQRDEERRRADRTEQKLAIVEGQLDQALGEIRQLKRNQDEGVSGDVR
N1301_01677	1745855	1746256	+			intact	Hypothetical_protein	hypothetical protein N/A MTSADRPEPAPAESDRADVFELDDPLVTDLSRFLLSAPLSDGTSTRMYPGNVEFVSQAVLNWLNGLVFDGGEWVPRAQIEVITDFGEVETTTLSDGEAVKMRHLPTGVVAIGVDAHEAWKQLRRRVTEVTGDA

N1 301 _01 678	1746 249	17 47 31 9	+			intact	Tail_protein	putative tail protein;phage(gi372217616);PHAGE_Nocard_NBR1_NC_016569 3.71e-11 MPEDPIERLNADIEVYGIPQTPGMPPMTESFLHIRRRQDGAPTRAVLGLPAYQGEPGERGPAGMVHQGTRTRAELEGYAMTLGENELNFTYRCAGTTDLWVWTGTTFKVYENAFGAKGDKGDPPAVQGGTVTVGGEPLDAPAGVRVTGDIGGPYTVGLDLPELPEGPPGPPGLSGPIYTSVDVDQTTPPADGQIPVHDAASGKLVWRDAMLPAEEYVVGSEAFPTVNKNAADVQHVLVSVVIPARPYLYRPDISGGVDIRRMIGQQIDVAVVMGDPNGGEVIGYGRGDAPDLVGGWWHVRVGSYSPITLNPGMRDGTVPPNTEMTFHMVALRRSGGGSWGTRNNYAQLRIRLQRVV
N1 301 _01 679	1747 319	17 47 95 1	+			intact	Hypothetical_protein	hypothetical protein N/A MAELRILPKPLARRPNRIDNLEYLGDFGRTMEDTRQRTNALEGIIGYGSFVMSQNQWLGADAGGAHRMRMQFDFPYGDSKGLTPISPTGRIRFDEPGLWKIDAQTRAWQTPAGGDNLIWLDIEARRANGQLLRKQSIDYPAGTGGKILRGGMTVEIPEEVGCYAEIWCYSGRWRWFWGGAEWTEFTLRKLSSNTMSQALPNPGQPGGNPE

N1301_01680	1747959	1749512	+			intact	Fiber_protein	putative tail fiber protein;phage(gi509142391);PHAGE_Rhodoc_E3_NC_021347 7.92e-07 MSIEFQHITGAWMITDYDSRDDADALPEQKSLKGTCTFRARFDPSDRAAAIHVPDPAGSFLLSVREMVFPIVHGRLIDREARDGVMLPAVVGGVPIIWTATPELQEDPGTGIQGARVPANVITFGPADPDGDGNRAVNLADIADTVIEYPEPVVSRVAVLVKQATDARDEIDGFVQYIADEVVPDIAAKTAAAREHAEQAGQYAQQAFEAIPPATDTQLGKIKLRGDLGGTAEDPTVPALTSKRDIVAGMNRVYTTDASGTQANIPYSSSATATTIMLRDAGGRTKVGTPAAAADAVTKEYVDTLLAKKADLGADGKTLQAQQNAIAVTDFLGNVASQTAMLALVGQRGDWCNRTDLGTEWQLVAEPSTSLSSWMQKIYPASDVTSVHGRKGAITTSSADITDATATGRALMKATDAAAARSAIDAPAQADLFQIAMDLGSTIDGVFVALNLRDAGLWSASGTYEVGQVVTYNHGRYYCREAHTPVAAFPAAKYVWLGLAAVAATSDPGGGRLWVKI
N1301_01681	1749512	1750243	+			intact	Hypothetical_protein	hypothetical protein N/A MAIEFVGATSASTTGMTLTVPVPAGGQAGDLWVMVLRSWTYPVANIGWGALPAYGVVGQWVERLVNTSRGGGSNLYVFTGFVDPAATDSPLVYFSGSGNHHATLLSFRGVDPDSPVNAVAQAYSSSGANNITPSVTTTVPNCMVVRAVAAGFSLSNGSCTWVSTVDELTDNRSSSGYSYMSTAASSSGPSPGPVGTATATYSTAGYGIMATIALAPKATSRLHVGSTSVPLMLGSTEVEIKGP
N1301_01682	1750545	1750817	-			intact	Hypothetical_protein	hypothetical protein;phage(gi372449851);PHAGE_Rhodoc_REQ2_NC_016652 6.72e-22 MKRPGGVDLAAWIVLAVAVLGLIGCLAIGAYGGALVWIIGGGGAGLALAGLRYRRAAAAAAEIAARADRENELYLDGDSSGLYGRYRPPS

N1301_01683	1750814	1751053	-			intact	Phage-like_protein	putative HTH binding protein;phage(gi589893331);PHAGE_Mycoba_32HC_NC_023602 1.03e-34 MPERPAVYMSRVEFAERIGVQPGALSRYKLPPPDVVVGPLNDDGTIPRGTVRGWLPATIDEWNANRPGRGARTDLGGTK
N1301_01684	1751046	1751204	-			intact	Hypothetical_protein	hypothetical protein;phage(gi479336495);PHAGE_Mycoba_Butters_NC_021061 4.64e-06 MLDVMRRNYDIVGTAATGGALWALHAPWPLWAVWGGIIVFIAQDRIRAARHA
N1301_01685	1751296	1751415	-			intact	Hypothetical_protein	hypothetical protein N/A MTESRMALVVGILTLITNIVMVIQNRKAKPKRRGRHRKR
N1301_01686	1751561	1752088	-			intact	Hypothetical_protein	hypothetical protein N/A MQIDVDRYRIIAKSAVAEGKYRSAPDTVVKALATLIGDAVATAAVGDVRVVQGETEWRIACLTEDGLLAYVSASCAISEWDSHQYDPEVLPTVAGWIASVSSVVDREVLGISDARGQGRMVGWHAATRTNFAGGRSLTLPLFGEVPVDDEGAADKFRVALDQAIAAAAVPSRALA
N1301_01687	1752120	1752659	-			intact	Hypothetical_protein	hypothetical protein;phage(gi712912393);PHAGE_Bacill_Waukesha92_NC_025424 1.77e-05 MIVRSRAGKIAAGVMSGIAAVAVLAGCSTEDSDAKVVDSTSAADAADAKTASEAKAVVGQTQEISTGGKKLNVTVADLVPATASPYAMPVKGSLYQVTVTIQGVEGTMPVNPMYVSARAADGTSYDSALGTVDGQLDSTDVSAGDTIKGNVAFDVTGAPIASIRYEGPLGDQLASWSVQ

N1301_01689	1753341	1754786	-			intact	Non_phage-like_protein	GTPase Der N/A MTDDYSADSATEYAGDGIWSEESDWDLADFLDGEDGEEHVAVPTLAVVGRPNVGKSTLVNRIIGRREAVVEDIPGVTRDRVSYDANWAGRRFMVQDTGGWEPDAKGLQQSVARQAELAMNTADAILLVVDAVVGATATDEAAVRVLRRSKTPVILVANKVDDARTEAEAAALWSLGLGEPHMVSATHGRGTGDLLDEVLRVLPETPREGTGGGGPRRVALVGKPNVGKSSLLNKLSGDERSVVHDVAGTTVDPVDSLVELGGKVWKFVDTAGLRKKVSHASGHEFYASLRTKSAIEAAEVAILLIDASKPITEQDLRVLSMVADTGRALVLAFNKWDLVDEDRRLMLEKEIDRELVRVPWAQRVNISAHTGRAVQKLVPALDTALESWDKRIPTGRLNNWLKEVVAATPPPMRGGRLPRIMFATQAGTRPPTFVLFTTGFLEAGYRRFLERRLREEFGFDGSPVRISVRVREKRDRGNKRR
N1301_01690	1754783	1755475	-			intact	Non_phage-like_protein	Cytidylate kinase N/A MTDGTALVVAIDGPSGTGKSSVSRRVAIALGARYLDTGAMYRIATLHVLRHGVDLGDPAAIAKATASLPLSIGTDPEADGVQLAGEDVTAEIRGAAVTQAVSAVSAVPEVRTFLVDLQRRIVAQAGRIVVEGRDIGTVVLPDADVKIFLTASAEARATRRNAQNIAEGRGDDFAAVLADVQRRDHLDSTRAVSPLRPAEDSVTVDTSELGIEDVIGRLLLVVGDRTGAVQ
N1301_01691	1755472	1756437	-			intact	Non_phage-like_protein	Ribosomal large subunit pseudouridine synthase B N/A MNKPARRDGTPDRKKRQPAKGAARSTAANKQQTNKRGKPKSAKPQRAQAQSHTPTVSNAKPAKHQYADVDRERAPMKGDGVRLQKVLAQAGVASRRAAEELIDQGRVEVDGRIVLEQGLRVDPENAIVRVDGIRVVVKKDLVHIALNKPRGWQSTMSDDLGRPCIGDIVSERVSAGQRLFHVGRLDADTEGLILLTNDGDLAHRLMHPSYEVPKTYLATVQGVVDRNVGKTLKAGVELDDGPAKVDAFTLLDVNGGKSLVRVVLHEGRKHIVRRLLEAVGHPVVRLVRTDIGNVALGDGRPGTLRVLGRGEVGGLYGAVGL

N1 301 _01 692	1756 450	17 57 16 3	-			intact	Non_phage-like_protein	Segregation and condensation protein B N/A MTSVGSDDVGTDDARSFEDEFTELPDSELAAALESMLLVVDTPASSAMLADVVGVPEARVETMLRTMAADFTERGSGIDLRYAGDGWRFYTRTEYAPYVERLLLDGARSKLTRAALETLAVIAYRQPLTRARISAVRGVNVDGVIRSLLARGLITEAGVDTDSGATTYATTELFLERLGLASLADLPPLAPLLPDVDVIDDITESLEADPRFARMDRSTSSSGSTATAPDPGIDIEN	
N1 301 _01 693	1757 160	17 57 98 7	-			intact	Non_phage-like_protein	Segregation and condensation protein A N/A MVVATDVVTEAPAADGPSGFRLRLRNFEGPFDLLLTLISQHQLDVTEVALAEVTDEFISYTRSLGSELELDQTTEFLVVAATLLDLKAARLLPAGEVDDDEDLALLEARDLLFARLLQYRAYKQVAQLFGELESAALRRYPRSVSVEERYVGLLPEVLLGVDPLQFAEIAATAFRPRPVPTVGLDHLHAPTVSVPEQAARVLELLRERGAGVWTPFGALIAECDVQVQIVARFLALLELYREQAVLFEQPEPLGELMVSWTGEDRTAPATEEDYG	
N1 301 _01 694	1757 987	17 58 91 6	-			intact	Phage-like_protein	sporulation initiation inhibitor protein Soj;phage(gi589892278);PHAGE_Mycoba_Adler_NC_023591 5.12e-25 MVTPQPPEAEPAHPHSRGALSYAENVEPVPDAPLGPTGRPLREIPEPGPVTAHGPARIIAMCNQKGGVGKTTSTINLGASLAQYGRRVLLVDLDPQGALSAGLGVAHHDLELTVHNLLVERTSIDDVLMRTRIDGLDLLPSNIDLSAAEIQLVTEVGREQTLGRVLHPVLDRYDYVLIDCQPSLGLLTVNALACADSVIIPMECEYFSLRGLALLNDTVDKVRDRLNPRLSLEGIVVTMFDARTLHAREVMSRVVEVFGDLVYDTVINRTVRFPETSVAGEPIVTWAPKSGGAEAYRALAREVIHRSGR	

N1 301 _01 695	1759 017	17 59 94 6	-			intact	Integrase	integrase;phage(gi971742625);PHAGE_Mycoba_Carcha rodon_NC_028779 1.08e-16 MTELGGLIDSYLDHLAVERGAARNTLTSYRRDLD RYARFLEARGTDDIRSVTETDVSEFVVALRKGDPA EGVVALAPSSAARALIAVRGLHRFAAAEGVTSGD VARAVKPPTPGRRLPKSLPLDDVLAILEASGGDGS ADNPRTLRDRALLELLYSTGARISEAVGLDVDDLD TQSRSVLLRGKGGKERVVPVGRPAITAIDNYLVRG RPALSVRGGPALFLNVRGGRLSRQSAWQVLHTAA EKAGIAVAVSPHTLRHSFATHLLDGGADVRVVQE LLGHASVTTTQIYTLVTVGALREVWAQAHPRAR
N1 301 _04 222	4446 252	44 46 91 4	+			incomplete	Phage-like_protein	DnaQ;phage(gi589893377);PHAGE_Mycoba_32HC_N C_023602 5.54e-16 MIPIVFVDTETTSLHPETRRPWEIALIRRDDHGEREF LFQIEDPDLSAADPFSLKIGKFYERHETDSMASDVR HPYDGVQRKTDTEACVALQVEEITAGAHLVAVNV AFDADVLDRMLRRARRMPRWDYHLLDMPAMAIG YLAGLAAHGHSNPACSMSDIPCVDDPVALPYRSY ELSERCGVDRPAEDEQHTALGDARWVARWWDAI APRAIALDGARVE
N1 301 _04 223	4446 914	44 47 18 6	+			incomplete	Hypothetical_prote in	hypothetical protein N/A MERYVSIPVEVTAHRMNVPSDADMIARWFGEQVT VKRELRRSGTTTVLERMLVCFPDQELVLYPGDWL VESRNGVFVLENSSFTDRYVKR
N1 301 _04 224	4447 183	44 47 43 1	+			incomplete	Hypothetical_prote in	hypothetical protein N/A MSRGGENPVLVAQNMGPINDYRRRNPAARSWPH MTRRSLDGVRGAKVSEIRVLEPMILTDQMITILYPA LSGAQVRRAAREQ

N1301_04225	4447653	44489972	+			incomplete	Hypothetical_protein	hypothetical protein;phage(gi41057231);PHAGE_Strept_VWB_NC_005345 3.24e-07 MWFQVDDQLTLNRKVRTLAERALEGREDGLAAMGVWTLAGSDCQASRADGVVRRADLIRLTLNPEMADRLAGLLVEVKLWHGPGHDCSRCPAIPDGTWLFHDWFDLGYNTREQLDISERKKRELKDSKIHAQVWARDCLDPNNPTVGACRYCGHEVKKKDTRSPDRRPELDHIDPNVADGARNIVLACRACNRHKGNRTPEEAGMTVLPAPGHNTATQTAPPTRSAAGVISPATTDAETTSPEPVAAEAAVTTAVSQHQVSPERANAETSQDVSPRSTAADTRQAVDQDATTHEPTADHPADHRTGAIPARGARAGAGQAGSGQGQGLGSGVGEAGSPDPDPHRAPRRKRRRGRGRGKPSTHQNPTAQTDPNPAPDPQPTPRRWDAGEPPPVKVPGTLGSPWRGWTGPPSTVVDESTCPDHRLPQPCRKCSTEYLGGNT
N1301_04226	4448969	44497748	+			incomplete	Hypothetical_protein	hypothetical protein;phage(gi764160901);PHAGE_Mycoba_Sparky_NC_026597 3.14e-19 MTEPHDHTCRADTSCRGRRRNEHSEWIPATTENPNTLCQSCTRAVETAVAHLWDDYLGLRRSFRDRDVRPDTGPKSGTPTPQIPVNVHTDALMHEIEDTTIRCAEAITDVLRTPPADIRDLGRALEIVEPNITALLRVPEHDAMDWNRAGEQWIPVTYDGPTLALQLADLHRRARATLGVERARDRMPVPCPRCEEMQLGRWHGDTTVTCLACGSSWTEDDYHRLTLILADDYHDITPPRRERHLARAYRNHHGNGGRQ
N1301_04227	4449745	44504497	+			incomplete	Hypothetical_protein	hypothetical protein N/A MTHPIEQLITFTPDEIRVGGDRIPGVIVAETVTVHQVPGHDDAWVVSLTLMTTALPLLAPGVHLDARGRVQVDPDPGERQLPPDREYRWVVESDNAASLVTADRYRAEGWLRSLFDAGIEATLTPPSKWLRIEDTPDGVWLLDDDGEYCQRADIIGLDDPATTGPFRLATDCCPADDPAPDCPVHGIRCGTVAASSASVQQDSVRYRVADIVQDLSEQGIIPTLARMDCETIGRAVQDQLNPSAQHGTRP

N1301_04228	4450676	4450822	+			incomplete	Hypothetical_protein	hypothetical protein N/A MTQQCGEISVKGYRCQRMAGHEGAHMHGLDPGSNLTTAWGWSAIGGSR
N1301_04229	4450819	4451589	+			incomplete	Lysis_protein	lysin B;phage(gi971762031);PHAGE_Gordon_Gmala1_NC_028972 3.37e-41 MISVLSCRGIGEPLQGNMCSAVTRRLDPTRFRVVEVPWTASYGPVPLPLGPAFDESLRTGRELLLRMIDEDPYPVVLLGYSGGAALAGNVAAEVARGEHPTLEVVGGGLLSDPFMPPLASPAQGTWGIAGFRDIVPRFPRWWAADPADVICCCTANSPLRTIADQTRGFSLSNLLGWGIDLVGRLKRQEWQQVAIDWRNRDEVHAIYRQAAHDLDGYLRRGDHQSYALREMPGAGYGYTYTDWLADNINILGRRLA
N1301_04230	4451586	4451828	+			incomplete	Hypothetical_protein	hypothetical protein N/A MSQDLVRERRGIHSGPVTHVQQTETALAAAVHENNELRAENARLQAADARARSLAAYWKQRAGDAPRFAAALTRTLDGDH
N1301_04231	4451980	4452309	+			incomplete	Hypothetical_protein	hypothetical protein;phage(gi764161655);PHAGE_Arthro_vB_ArtM_ArV1_NC_026606 3.59e-09 MAATATDRCLPVAWDGHPVEWGPFSSTVFICRVEADGTSTAVLDMCWNCGVHADRVQCEGRVDIPHFEYVLHLYRCTRCGHDQVQNPRTRDLWDLDESDYGPDGSWEEP
N1301_04232	4452306	4452647	+			incomplete	Hypothetical_protein	hypothetical protein N/A MTRYDDLPAIPMWEIPIWQNSAVANEPIPYDEMRAILGLPDVRAAVARDQLVRTATRVRVLVEAAATQIGAQLVVAAESMRPALDHFAEALRPRPTPPMWAIDETRTHRGRRR
N1301_04233	4452644	4453054	+			incomplete	Phage-like_protein	gp89;phage(gi304361034);PHAGE_Mycoba_bron_NC_014461 1.45e-20 MSGLWPWPADTPVDIARRLLQSYREALQRRDPEACSRIDAQAQAFGQGWVVPRLETVDVDALVSGVEAAELAGVEPQVIWQWAYRGHIPRRTGVDGSTCYRVGDVLDHQARQRRRRGARRRRHADTGDGACVDSRA

N1301_04234	4453135	4453443	+			incomplete	Phage-like_protein	gp97;phage(gi304361041);PHAGE_Mycoba_bron_NC_014461 1.68e-14 MRDQPRSKGRTGRPYRRAVEQVKRRSQVCWLCGEAIDLTLKNPDPMSFSADHVETVKSLPPNDPRLNDSRNMRPAHLSCNSRRGDGTRIEGPSLRTSRRWLR
N1301_04235	4453586	4453906	+			incomplete	Hypothetical_protein	hypothetical protein;phage(gi372450002);PHAGE_Rhodoc_REQ3_NC_016654 6.66e-14 MTGSIDLGWEPQPVTMVVSGGAAWIVAIAPVSYPVGTTVGLEVTNHDFSEHVFEADVDPASVSWRIEAADVDQLDDGAAVRVRVVFPDSPTPTPVLWMKGSVRRDD
N1301_04236	4453899	4454363	+			incomplete	Hypothetical_protein	hypothetical protein N/A MTELGGGTVPVPPAARVVRVGVPGSSPGVAIPIAGQLTDAQIALIVDEAVKRVVEQLGGAGGAAPFVTTVQPGPVDLDDMASMSQVITVVHNLGTTTPNVSVQNSRGDYVLVPWNSKGTPNAVRLTFGPRFTDVDMESGPIIAPPDTYTVRIAK
N1301_04237	4454360	4454923	+			incomplete	Hypothetical_protein	hypothetical protein N/A MSDNPAPGEEASPPAADMVAHVGSLFPMLPDGAYWESLSTFFQFQSLSMSVEARIEEFRTFAEDLVLFTGRRELGYGAARLGTAPALTHGGGVWGVRARVITPTPDADTEIHCGYYFPDNNTVWYPFSDYVNGEFGVRKLIIPAGSLTGEADLGGSHVPYGACLLARVMAVGENPGNGLAVTFIGRR
N1301_04238	4454926	4455174	+			incomplete	Hypothetical_protein	hypothetical protein N/A MPTATLIAGGFVFSHRCGDRDLRQVLPLAPTTLWRVETADPLTITPSILCPVCRVHGWIRDGEWVPAAPAAVDPAAPPAVPM
N1301_04239	4455184	4455597	+			incomplete	Hypothetical_protein	hypothetical protein;phage(gi508177872);PHAGE_Mycoba_PegLeg_NC_021299 2.96e-14 MDESTTEIPDPPVGLLKRGSALWAALAPARAGDPGALVLLAEACRVADRCERLDALLRGRNREWMRLVVDDRRPRTFVVHMDGALREARQQQLALRQLLAQLGVGKGDAAPAPETKGSVLDELRRRREERERGAGAG

N1301_04240	4455542	4456999	+			incomplete	Phage-like_protein	gp4;phage(gi304360953);PHAGE_Mycoba_bron_NC_014461 6.12e-114 MTNCGDDAKSESGAPEPGSQPPRLCNYPPFFTTLGDDAIDLAAAAELELLPWEQVLLRKSLGETEDGRWAATQVGLLVPRQNGKNIVVEARELAGLFLLEERKILHTAHEFKTAKDAFGSLAARIAVLPELEELCQLPHRTSNEEVSIRMRDGRYCRYIARSQGSGRGFREVDLVVADEAYALTDTHLGALRPTQSAAAARGRAQFWMTSSAGSGSSTVLARVRAQGIAQENPRLLFAEYSCEPDIDITDRRQWAVANPSLGIFITEEFLAEQLDLMGEIEFAREHLGIWDDPRTTAVITPDMWKACRDEDSEVTDPIALAVDVSPDQSVASIAIAGRRPDGLPYVELVARDQGMSWVPEAAARIFFEWDALAVVVDGAGPAGALISAFKELEIEPYVTGPREMAQACGAFFAEVAAQQLRHNGEPELASAVSASRKRAIGESWAWARRDATADITPLVACTLALHGYSRKVSEPAKRQRTGKVW
N1301_04241	4457004	4458350	+			incomplete	Phage-like_protein	gp5;phage(gi304360954);PHAGE_Mycoba_bron_NC_014461 9.12e-128 MLVESEAVAVAKDLLPKLKRESARLTRLDKFLRGEQAPAKLPRSATPERKALAELSKSPWLGLVVTTLAQAMFVDGHRVPDDKENSQQWKTWLANNFAARQIAVHRAALAYGYSFTVVTPGVDTFGQTQSVIRGVSPRRMFAAYEDPAEDEWPIYALRDDLGGRLRLYDDHLVHRLRRSGSDEIVHDGFEVHDAGVCPVIRYAPDLDLEGRATGQVAPFISTAARIDKTIDDRLQAQHFNSWKVRTATGMAEPDTEEEANRAKLKLRQSDLLIAEDPDTKFGTLDETPLAGFIDAAEADVDTLASVSQTPSYNLTGKMTNLAADAITAARAAFTQKVFEITTSLGVSHNQTLRLSALLEGDYAAAGDVQAGVTWQDLEVRSLAQAVDALGKAAKMLGVPPQALWARIPGVTKTDVEEWSELLLSNDPVTRYLNAQFGPDASGDAGLGI

N1 301 _04 242	4458 350	44 59 97 2	+			incomplete	Head_protein	capsid maturation protease;phage(gi508177875);PHAGE_Mycoba_PegLeg_NC_021299 3.62e-28 MAVTRVGQRLTEEHRAAQREVALRTVADLRRAWPVLDFERLRETQGPWLQAALPVLARGHEASQEITAQYLEQFRAAERPDIERLVLTEPALRLGDRPNRVMVREGVDPGPRSRVTVSGGGDTAGRSRVRVVDPVAFDEQSAARTLLSTGPARVQVLMPAPETVAMEKAFVTSTNAGVRVALDGGRDYTRRVVDLDAAAFGYARVTKTDPCWFCALLASAGAVYKGQSSFDTSDARFEGLGTAKVHDGCHCTLEPVYDERAKLPGQAADFRTLWDESTGNATGKEAVLRFRRAFEGRPVDGGAQSAAAPAVETDREIAERLLPRFEAQLSQLIADGRGEDSEPVVYHREQIDRHRETLGLADGEPFVPETAGGDGPADPPGPGDGGAAVAGDDEWQVPESYDGVRGAETVPFRPADRPPFEEDDVEYILDGDPMPPVGAGGGGHRYDARREGKTEFPDWTDEKVVELLDRILAAPQRVLARSDGSFEFIGYREGIVAKVVVEPIGRGWVIRSAHPISGAGVRTFSAGDYIDRELDLSVLN
N1 301 _04 243	4459 974	44 60 27 0	+			incomplete	Hypothetical_protein	hypothetical protein N/A MAGMRPLEEFRDDVLARVDGIFTDDDRLSIATSLDAGESIEVVRLAIGAAAARDLRLPTGLLDEAYHWGVEQTTRRRSAEAIARQIDHLRSLNVRRSA
N1 301 _04 244	4460 445	44 61 01 1	+			incomplete	Phage-like_protein	gp6;phage(gi206600187);PHAGE_Mycoba_Ramsey_NC_011289 1.63e-29 MPKTQENGESQENGGGAGGNGPEGGGGQENGGGGDSFTPITSQADLDRIIGQRLTRERAQYANHDQYKASHEELTKLRDGEKSELQREKERADAAEKRSKELEDDKLRRDVAADKGLPAKLAARLRGSTKEELEADADELLADYAPPADSGRRQLPGKPRENLRGGGSPDDEPEETDPAKLAALVPRD

N1301_04245	4461073	4462221	+			incomplete	Phage-like_protein	gp8;phage(gi304360957);PHAGE_Mycoba_bron_NC_014461 1.54e-93 MPQNETNEFLKAAVIINAGLGILEREIAVPRLVWLNGFGDFAGAKDDTISIRVPGRLTARSRKLRATGEDRKIHMDTIAETKVDVTLTDDIYQAVPITDEELTLDIKDFGKQILVPQVRSVAEGLEDGLVEEMRSADYQATVTVDPAKTYNSFVDARKALNDENVPFAQRSALIGSGIEADILKDPQFVHADQSGSTSALRDALIGRIAGCDVYVSNALDDDEGYMFHKTAYTMVTRAPVVPDGASFGASQSYNGLAMRWLRDYAFDITTDRSLVDAYVGYGHVIEPDGRFVRAVRLQRGTTAITVSPTTASVKVGETTKLTVKDDGGDAIQSRGATYTSSDPTKATVSSAGVVTGVAVGNATITAKYQGKTATAAITVTAA
N1301_04246	4462233	4462727	+			incomplete	Phage-like_protein	gp9;phage(gi304360958);PHAGE_Mycoba_bron_NC_014461 8.97e-20 MTQPTAGPHPLATPAQLGLRMQRTLSGAAESRAAAILDDVSAFARSEGRQWKDAATAGPDVVAVVLAAAQRVFKNPDMYVSRQAGTFSHRVHDSAFATGTFTAAELAILRRRRGKSGMWTQETTRGEDGLATGFLPVEGSSEPFPVYAADDPFNSYGDHYSGGW
N1301_04247	4462727	4463053	+			incomplete	Hypothetical_protein	hypothetical protein N/A MGPGEGQSITVIRPVPVDRHRDPVDGATPAERTVDDVLIDWDGTIDTRGRRRQQVADVRLYCQPGSDIKAGDTVVLPGDEKFTVVSARPWAYGGWEPGVVVTLKGVQG
N1301_04248	4463050	4463376	+			incomplete	Phage-like_protein	gp11;phage(gi304360960);PHAGE_Mycoba_bron_NC_014461 7.23e-07 MIRNYRPNHAGVGALLRGPDMRRLVQHKAELAQALYRARVARRTGRLARETRVFTFIGGPKNDRWIGRMVAYAPYAALHEFDRVDDNGRVIPGARDLAAVLARMDSLP
N1301_04249	4463373	4463816	+			incomplete	Hypothetical_protein	hypothetical protein N/A MNDFPSIDHLTDWPDVEDLMCTYLERFGRTVTETDLSDDGRRELETFLGGGGVLIEINRAGGGAPDGLKDIAPIVVKVTTARRSRSWRVMNNQIRKAVKVAENGVAVDGVLLDSMSETGDLSLVPGLDPDDRSVEML

								FLVTCRRPRF
N1 301 _04 250	4463 907	44 64 88 4	+			incomplete	Phage-like_protein	gp13;phage(gi304360962);PHAGE_Mycoba_bron_NC_014461 1.35e-47 MSITSVKNARDELLISALDLAVILAPYGTDIPDSLSGPDMELLPFTPEWEGQGLIEKKAGVDITNESDSTPIESYGELDPTKMIKGKKTTTIDYTAQQSGRSQLEVYWSADYSDVVPDAQSGEVRLPAPSAPNIIYYSAILLGQDGGPGEEIYPYWICPKVSVTKTGKQSLTPEGIITYPGTLTAYKSRIHGYSIAQGFCGTGWKNLVDLGVTGFSSGNPVNEIQKITIEGSPTGGTFTLSHGGPATSAIANNPTVAQLKSALEGLPSIGSGNVTVTGATGGPFTVEFVGARAGQNVTQLTANSSGLTGGTAPTVTVTTETQGHA
N1 301 _04 251	4464 968	44 65 32 7	+			incomplete	Hypothetical_protein	hypothetical protein N/A MSDNVLGGKFLEYQERAAGKAKSAIVFGADEGFDPPIVLPTPTADRVDRWNRAFSDKDKLKVLIAGSVDAEGEAAKNFDRFWAVVGQWDLPTFGELVTDISDHFFGKGATKVPGGSQRS
N1 301 _04 252	4465 537	44 65 76 1	+			incomplete	Hypothetical_protein	hypothetical protein N/A MQSGPPPLRGYSAQMYQQAQAFNTMIALLAGLSGADGDGMMLQPPETALMRARKNIRSNRMSSLLAKALPHQFQ

N1301_04253	4465777	4470372	+			incomplete	Phage-like_protein	tape measure protein;phage(gi359802294);PHAGE_Gordon_GTE5_NC_016434 5.96e-46 MANYSAGSASVDIGSDLTGFHHDLERKLNAIEAEFDVRVAPDVTGFAQRLEADLSRLDATFGVGIAPDITGFAPRLDAELSMVRARLDVDIDPDLTGFGASVEAELARLDGIEIEVPIRPTFSVAAQLAARGEIERFARTLHPRVTIDVDVDRDRLGALGFLGGSGGGGLGGVTSSTRSISQLGGAAGNTAGQIGGLSSSMGSLASAAGSGAAGVAVVVAVVGLIAEVVPVALGAIGSLGIGLAGLATVAGPAIGAVAVGLQGLGEAFTAGEAASSAAGQDAEAAAKRYESSVRTLGDAQRTYERSVRDEVRAQEDVTRARKGAQEQLEDLALTVRGGVIAEQGAVLALAQARIDASKIETGETPLDRANKLQRVAEAEQRLLEIQERNKDLSDDVADQGARGVEGSDQVVAAKQRLFDAEERVTTAQQGVRDAQDAVNEAMTSGSSTADAYAQALEQLSPNAREFVTAMRALNEEGGAWHEFSMSVQDGLFDGTADTVTNLANTVLPAITPAMTDIARSVGGIVESISDVLTGPAGEQLTTLLTAIPQFFTAVTPGIEATTQGFMSFGAALAPAMESMGAGFGTFLGRIGEAFTEMEQSGTLTAAFDGFGQAMAGLGEWVGPLVTMFGELGATVGPILGDMFTSFGRTVELMTPAMNQIADVFGNHLVVIFEALEPVMPALTQAFADFFTAVQPLSAVFAELGATVLTGAAQGFSTFAVAMAPVAKTLADELAPIIPILADYFADTIPIIADVSSVLSQKLTQAVADLAPYLPEIVRAAADLAIALIPLAPQFVDLATAAIPPITDALVVMAPHIADMITLFTNVAEWLLPKLIPALQGLEGPVSAFGGYFTSMALVVAGVWDGIVNTIKGAVGQVGQIIKGIGDKTAPLPGNHGEQMQRLGQSMIDWGGRTSDLQGGLKSALAPVPGNAQQESELEPGVTAGKRQRRRGYAVGGRLRGDGTWTSDSIPILASDGEFIVRAASTHKYLPLLEAINNDTLDLPGFATGGAVGRASGWAASKDGIPYVLGGLDCSMYASGVYQVLTGGDPDVRAFDTTKFATPQLAAAMGFEPGLGGLFSVGVTPLPGASGHMAFTLGGVNGESGGGHGVMWGQYAAGADDPQFSVQYHLPGYLFDPPTADTGGFASLFSNDGIYVLPGLTGGDGAAYGDYTPGDLYGPGMLGGDYEDSWNRVDGYTTDPRNRAGEDKNL

								SVQEYGRKAGEIGANWLLGIVGLENSILSESNVYN<br>RAYNDVQKFADDRGAAERAADLRLYDQMNRPAL<br>EGGVAGEAAALARQYSTIEPFTYSAPNPPPDHVWD<br>PTQGAEQWYSTVEEVAIGTGRPISVVPPTIEQIGIES<br>SGNPDAINDWDINAQNGDPSIGLLQMIGETYDRYA<br>DPRYPGGRTVPKSNIAAAFNYVDAEYGGVHNIWP<br>KVMGYANGGPIEGPGGPRDDLIPILASNGEFMVHA<br>EAAAANRPLLEAMNAGARWSRAVLPSGSVAPSPL<br>TGGSVTHDRSINYGDVHVGNQDELFAIRERDTTGA<br>HLAALGAHW

N1 301 _04 254	4470 383	44 71 27 0	+			incomplete	Phage-like_protein	gp17;phage(gi304360966);PHAGE_Mycoba_bron_NC_ 014461 1.38e-55 MSEHLTYELFGCEDSYWCISGPNAGEQGVELAPKP SKLMDAPVKTLWSKSAFGEKYEGKKRLRRDPVLA LDIEGEDPDHWRGVDSRIRMAFDYDRQSRIRATTA DGVRDLWVRLLEDPEQTTQDGKDPHLFAQSRLTC KLAAEFPNWQAKVTTASWTLPSGTSGTGYLPVYN DGDVEIWLDYVCTGPKGIYTLPDFSFKNDVAAAR TVPLPELVPTDGDLTVRTMQSEEPLITTNRTPIWNR WNGNGFLFPIPPFTGTKKDPIMLPVSVKNGIAGVST VQVRNPHWYSRPMGVKR
N1 301 _04 255	4471 267	44 73 00 0	+			incomplete	Phage-like_protein	gp18;phage(gi304360967);PHAGE_Mycoba_bron_NC_ 014461 1.87e-173 MTTGFTAPSVEELEAIWNRGTAIREDHKALRRTPP LVRLWANDPADEKGLILRGSAQDSVAGQFPFKKK GASTGTLRLRIDHYLARWMMSIPNDSAAKKNVVI SVDHMGGAMRWSGLLKNWTIRKDRDGIRYLEAT FIDDLQFLQYLHGAPNPLLPLWLFQFPRVFNVLGPS RWCVALLIWMNLARFQSNIYNVPDDPFQLGSWID GWNMNTWQVLIKGVSFLGDSSLWTILSSRMDKVE SAIEDALDDAQLVLTYRRVFTVDGETVDGVVGVT NPRNGVLVLEVVDRSGYFGADGTATRGTALHGFV RTAVEFAEGFVEQADVFVSEDQSLFPPEYYWENW LGIKPSHPCFVVRDSEYTQTEAAELSWGPAGPVQII VGGENEYADSAVELAIQVTGNLLGYFMLGGFSSA GDIAATVIMPLLRGTILAWNSWKSTSRAQQLGWV HLMEMYQQGANNAWTLSAAAALRAGFEATKAT GSGRMDMRDGEPFLPGLHIRPGDRIGQTIQGMTGS VTTDPVFVNQVELMTLGWDYANDTPHGWEIAAG TNKSVLSAAERSARLLSKLLSSVQNQGVSIV

N1 301 _04 256	4472 997	44 73 50 6	+			incomplete	Tail_protein	minor tail protein;phage(gi508179934);PHAGE_Mycoba_HINdeR _NC_021308 2.87e-11 MSGIPLQDDCSPDDPEEHLLWGFIDLPLTHGHSVV MITQPSVLREWSKRMYQRGFRHHPELQTEFYVPP ATGHSPFGAVGTWVPREEADAARAQNQAAAQAEI DALTAKVVESMPEMAERIAAMSDDERAAARESM KGILGEQLQQLRQHAEADSASSAAAPNAEENDE
N1 301 _04 257	4473 503	44 74 62 7	+			incomplete	Phage-like_protein	lysin;phage(gi372450115);PHAGE_Rhodoc_REQ1_NC _016655 2.50e-84 MTATLPSRPDFDEKWMLSQSRSSRYGAAVTNALW HTEEGNATAVALALYCGNPANNASYHDIVRDRIV AHVVDDDFASWSALAANPYTYNLCFAGSRAAWT ESQWMARADDIRIAVWLTLEVARRKGTIATEIIAL GGGRYRGRMSGIADHYYVTKVLGIGNHTDLGMN FPWWFAAQVLAEYLAPAPLPAPVVPAIDEEIRRIRE VNDWLGARITLGENNCRGDRGGKWVKLENGQAY WSAQTGAKIVPNALLDTYGLYDWEVGPLGYPIGD HTVLTAPGAGTPWGDVQHFEGGTLYRRYSADRG WWVHGLIYATWRRSGFENGPLGWPTSDEIELDNG DRVQHFEGGDIYWSPTGTVALKPVDGPDQHFPISH
N1 301 _04 258	4474 643	44 74 86 7	+			incomplete	Phage-like_protein	holin;phage(gi530545497);PHAGE_Mycoba_Hamulus_ NC_022056 8.21e-14 MLTKKFWTDSAERAAKTGAQTLLALLAVGTTVL DLDWGNALAVTGTATLVSLLTSIVSSTIGDPSSASA LGREE
N1 301 _04 259	4474 873	44 75 12 1	+			incomplete	Hypothetical_prote in	hypothetical protein N/A MVPDIIQAIGVAVAAILAAWNARQAKQIADLRTD MERLQQSELESRRLLRSAVRNIRDWLRWDAAGRV GAPPAIPDDLRDEV

N1 301 _04 260	4475 125	44 76 22 5	+			incomplete	Hypothetical_prote in	hypothetical protein N/A MSSPGGPSGINRPEYGVSANDGSAARLAALTQEAV IAQKKAQLKGSGSWGGAWDNFLATIGAAIGGVIA GVLNGFANVIDAIFGTVNNDYVKQMPIINDHTQTI KDLREAYDQMILQGKSIVFTSDNTYYPTPGVTSVE VIIIGAGGGGGAGEWNVLANQIYGGAGGGGGGEV HAILPASLLPAAGVPIAVGYPGSGGGSSGAPGGGG GSTSFGPLMAGGGQGGTASSAAVGFAAEGGTGMI RGGRGGAGAYGGNGGNGTDSTSPYALYGGGGGG GGGGIHAGSVFQPGWGGTGGISPGGAPGTTGTAPS GIVATGAGGGGGSSRLVGGAGGMPAGGGGGGWA SGVDWTRGGQGGAGIVFVIERFT
N1 301 _04 261	4476 225	44 76 98 6	+			incomplete	Hypothetical_prote in	hypothetical protein N/A MAIATNMMVFTASGTWHKPPRCRAIELILRAGGG GGSSGIFHPETTKRLPGMGGGGGAAVVTRRINAFE LPDDVPVAVGVGGAGGRSPSSGSLTFGGNGGESSF GDFAIVAAGRGGGDGYVFAAAAGGLSFLRGGHG GVNGSPGESVTSGVVLLMAGGGGGGSGAGYVNG NPLPSQGDGGWSGQVPPGVSLPSYWQACQSGGGG RGGGYGALAQPGGFPAGGGGGGDGGGQAGANG AAGCVTVIEYIEGEISE
N1 301 _04 262	4476 979	44 77 31 1	+			incomplete	Hypothetical_prote in	hypothetical protein N/A MSEMGTATVVQEELGGYAGVAHCYRVDPPLENF THVTVWVQTAYGTPEAVIVGARESGAAFTMSRLP GSYVHPQATHSGALWLAGYEVVTAEPEPEPEPEPE PEPEPEVS
N1 301 _04 263	4477 311	44 77 63 7	+			incomplete	Hypothetical_prote in	hypothetical protein;phage(gi372450002);PHAGE_Rhodoc_REQ3_N C_016654 6.52e-06 MAEKMFGWQPTKRGMILSAGADFAQALRQKNSA GVFPEGTTARIDFVVDDEVIATWPATVEETEVSWL VQSTEADEIPDRAGYRLYISFPFDPPLDMLWFHGA VQRKQ

N1301_04264	4477650	4477985	+			incomplete	Hypothetical_protein	hypothetical protein;phage(gi971755901);PHAGE_Mycoba_Llama_NC_028923 2.66e-15 MAISQPATRQILANAYAPTAYISVHTGDPTTANEATGGTPAYARKQTTWTPGTGGIKTGSQVAIDVPPGTYTWVGIWDAATGGNLLDKQAMNSTTLGYQGQILVSPTFTVS
N1301_04265	4477999	4478988	+			incomplete	Hypothetical_protein	hypothetical protein;phage(gi372450030);PHAGE_Rhodoc_REQ3_NC_016654 2.30e-14 MIDGRTPIVADPPTVPVDGIVDLWQALTRIVAEEHLPAVSATVVPSEWVPLLKRVPIALSAVGTLALQVGVRASVPRALSAVGTLSLAVSVRTSVSRVFAAAGAVASAVTEKAPVPVGRSASGTLSATAVAGHPRAVAFSGSGQLSVDVRTRAKVAPTLAAETWLGHYYSPRTAAPLPASATGALSVDTALPKVPMGMNKVGTQQIVGDSNSSTPIFPRSTKVRGWAVRAGYPSTVVFDDGLRATAAMSVNFTGRVSFQSGGVAQFVQFMVVKNDVEVLATGSTGSTLTGTATLEPGDTLSLYAWGSSAVTNTYRNVVEANTYLYWAVS
N1301_04266	4478997	4479146	+			incomplete	Hypothetical_protein	hypothetical protein;phage(gi372450031);PHAGE_Rhodoc_REQ3_NC_016654 5.12e-22 METVTLPGLPGLPGHTITLAVDGAWLACEVRDSSGEVRYSAGWDTTPTT
N1301_04267	4479212	4479370	-			incomplete	Phage-like_protein	gp44;phage(gi29566152);PHAGE_Mycoba_Che9c_NC_004683 8.86e-07 MKKEHMIDAVDQEAVRLLETDPAKFFDSPRRIPFGFVSSKSSSSGDTTSSES

N1301_04268	4479367	4480311	-			incomplete	Phage-like_protein	gp45;phage(gi29566153);PHAGE_Mycoba_Che9c_NC_004683 2.43e-41 MTETIDDDGFRWPHWMAYAPANVPPALFAVTGIAGSVVGFPPGWWRLIPIGIMATAFAFAWAIDAARSSREDRDYRQVQRERDEARAQLATLRAEFNAWVTNYRASLLHYDLHNLLVLVATAVATNNRQDRASQARTARQSIICAAARLIGESEQNGTRANLFLFETPQLKGSADTSAAMSLEPGAFHGRGVQSSRRFAWDHPTTLATLKNKAQFVIVSELEPSDDPDDDVPYAAYATHPVSVNDQVIHGVLTVDSMSEGVLEEKVDLPMMAVLSDLIAITLECEKYPKRPEPRQSVTSIEFTSRVRSVQRGFA
N1301_04269	4480510	4480770	-			incomplete	Hypothetical_protein	hypothetical protein N/A MRDDDPQLTGLELYATMCRAWQEYIDAFADPDAAGFFGEMTEQDRLAMAPEIVEFLRSDPEFWESYSGTDQALIARAAEAAARGEC
N1301_04270	4480983	4481180	-			incomplete	Hypothetical_protein	hypothetical protein N/A MLDTARLPAPTARPWRWIDTHGDGAPVPDRQIEQALTALAAAGPGATGSRIYATYHRQARDRGLL
N1301_04271	4481200	4482021	-			incomplete	Integrase	integrase;phage(gi109392319);PHAGE_Mycoba_Halo_NC_008202 6.18e-85 MTTRSIPVGWMPYLDDFTLALRAAGAPQTTIATRLNHLRRAARTLGAPTPGDVDGDRLIRWAGRQEWSIETRRSYRQSLLAFYRWAEGSGRVADNPALALPHVPAATPKPRPCPDHVYAAAMAKASARERRMLRLGAECGLRRAEVAVAHTRDLIEDLEGWSILVHGKGDKDRVVPLPDGLAADLRTLPHGYLFPGNENGHLSPRWVGKLMTELLPGEWTMHKLRHRFATRAYRGTRNIRAVQEVLGHASVATTQIYTAVEKDELRAAVRAAA

N1 301 _04 907	5152 467	51 53 41 4	+			intact	Phage-like_protein	gp55;phage(gi593775280);PHAGE_Mycoba_Avani_NC _023698 2.29e-45 MSELNLFDAEQSPFDQIRQVRTDGTEFWSARDLMP MLGYDRWENFEAAINRAEAAAIAQGHDVITLFRD VTKKGVGRPQADVELARFACYLVAMNGDPRKTE VAAAQAYFAIRTREAEVAPVRPELTEDEIVHRALA ITTRKVETLTATVAELEPAAAAWESLADAEGDYSL REAAQILSRDSAINTGQNRLAKSLRELGVLDRRGE PYQAHINHVRRRPTAYTHPHTGEPKLSSQLRITVSG LRYLHGKLGGTRPLRLDIETGSAVAGMTDHPADS VLDATLEHIPPSGRARALVDHHSAIADQFVNQFKG EQP
N1 301 _04 908	5153 411	51 53 93 2	+			intact	Hypothetical_prote in	hypothetical protein N/A MTDATDVLRGRETLRELVARMLSAGLIDQAVAAE LLSVDAAQAAASPVGPRNLPARSALVEYLPDGQF RLVRTGKVWTVEELIIGDEWEQQGGHHQCKRDAL VYIDRVRNPRQFTVRHGAAATVGTPGVDFTVRPG LAGAIRLIARTDTTSFCATLTPAAAADLAAELTGAI L
N1 301 _04 909	5154 006	51 54 28 1	+			intact	Hypothetical_prote in	hypothetical protein N/A MFGFTRQTPATPVDVIAPANARPDEIAAEFTEDRD AAILATADDGRAGIDRAQRHQREAWLAAIVLGVT LGAIAWAGATAAISSPLAGLAA
N1 301 _04 910	5154 278	51 54 52 6	+			intact	Hypothetical_prote in	hypothetical protein N/A MTASTEPRLPTTPESSWKACTLHEFRRHTGKRVVY LSVATGKPAETGIVSHVDDRWVYVHYSGSQGPLA THPDNLRLTGERR
N1 301 _04 911	5154 523	51 54 68 1	+			intact	Hypothetical_prote in	hypothetical protein N/A MSDSGIDDIERDLLAETRLRSAHALLIEQRRANAH AIAAGAPVTDFEETTCD

N1301_04912	5154674	5155669	+			intact	Phage-like_protein	gp58;phage(gi206600137);PHAGE_Mycoba_Pacc40_NC_011287 1.09e-79 MTELQTRKPTGRPPFPLILLEGPSKSGKTYAAAELTASERVGRAFWLDLGEGSADEYGAIEGADFEIVVHNGSWPSIFAAVEQVHAVAARAAAAGEPPVMLVFDSMTTEWEMIKDWVNRRARQTKAAQKILQDDPDADIKAGNLWNDGADRHGQLMRLLKTFPGIAVVTAIGTETVALDKDGRPIPGERDYKVEANKTLTRDVSAWVRYSHDTGPRVVGVRSPRAGYRPGVDRLEMRPEFTLEWLVFDLLGCGSDTTARDLVAPLSLVDLEGFALAADSAEWVNETWAMAKDAAMLDVALPGGRTARELLTEAAGRIKAASGQPAARGGAQ
N1301_04913	5155666	5156802	+			intact	Phage-like_protein	gp51;phage(gi593774681);PHAGE_Mycoba_BigNuz_NC_023692 1.14e-58 MSITIATKSLIELLTDLVLTATDIRGVHLRTTRGHLGEDPEETTLLVGTSTNGAVLGHTWTRCAGELPPMVWPTMNCKVIIGALKSLAKGDKGHAVDVVLDGKTVTVMETATLFDDGDRFEFQVSDIEGYPLARIHRILAGDPLPIPRNEGGDDLPDTARTTWAPGHLATLLKIATRRDKTLHLFRRHSNQIHLAQIGDEWVGAIAPVSGWDHDDPDRPNTDLYLEDPDRLDGAWLLRYGGGAFIGSTDFPDEQADPQPEGAASEVVQPALDDPGPDVAVDVSAPAEDDELDVEKLVAAGEDDLLRDAAQMVLATRFGSASMLQRRLRIGFAKATRLLDELSVLGIVSAADGSKAREVLVDEAGMRAALEQRGDRGTP
N1301_04914	5156852	5157487	+			intact	Hypothetical_protein	hypothetical protein N/A MAWQLVKEVLDGAPPSLKAPERLVLVAIAEWCDVETRTCWRLNEELRLRVNMTASGLRAVFTRLAAVGADPRVPIAFAADGSPVYAYKGRTTTFRLPYMTAVNGHEGDTTATPSEGDATASPSALKGDTTAASLNDPQPVSDSSTGDTTVAALTDAKATPQRAKATPQRAKATPQRIRGDTTVAPSPSKTKRNQFFPREQASSYPQLRAIN

N1301_04915	5157500	5157985	+			intact	Phage-like_protein	single stranded DNA binding protein;phage(gi372449997);PHAGE_Rhodoc_REQ3_NC_016654 6.34e-74 MAGDTVITVIGNLTADPELRFTPAGAAVANFTVASTPRIFDRQSNQWKDGDALFLRCTIWREAAENVAETLTRGMRVIVSGRLTQRSYETREGEKRTVVELQVDEIGPSLRYATAKVNKVSRSGNGSGGGYAGNQDRERPPADDPWGNASQAGSYDDEPPF
N1301_04916	5158002	5158160	+			intact	Hypothetical_protein	hypothetical protein N/A MRHTPDTYRPGAYAHCPKCHGALPNHGLVHVRHGNGGGHNEPCPLDDREDGR
N1301_04917	5158157	5158669	+			intact	Hypothetical_protein	hypothetical protein N/A MNAPTVLGIDPGGRSTGLCLIHGRDVLAHETITHDTQHLLPVSRNYLRDVVERVNWMRRFNPLIDPVLAVEDVVRPNWHMNGRAAADPSALLATAAVLGAVLAIDSRGHTVLQVRPKGNGSRPLGAYPAELVSPGERRKPGWQLRIGSGQLRHARSAYDVALAGQEAIHA
N1301_04918	5158666	5158929	+			intact	Hypothetical_protein	hypothetical protein;phage(gi966198577);PHAGE_Gordon_GTE6_NC_028665 1.90e-06 MSAELPEPSRCRSCRAEIHWGKTPAGKHLPVDATPAKSGTVALDVHGGVLYAGVLVGAQLAAVRRSTRPLYEPHWVNCPDAKAWRNR
N1301_04919	5158926	5159237	+			intact	Hypothetical_protein	hypothetical protein N/A MRDTPQRPFAHLFPVHTVVIARTLLDGQKFLRARQAPVDNPLIVTHPGQLDGGVRIRKDLVLCTPTAQSNPRFQEIAARLQAAIRGALAIANARANNKIGDQE

N1301_04920	5159234	51599998	+			intact	Lysis_protein	lysin B;phage(gi971762031);PHAGE_Gordon_Gmala1_NC_028972 7.26e-45 MITVLACRGIGEPMQGNMCSAVTRRLDPTRFRVIEVPWTASYGPVPLPLGPAFDDSLRTGRELLLRMIDEDPNPVLLLGYSGGAALAGNVAAEVARGEHPRLEVVGCGLISDPFMPQYASPTRGKWGIAGYRHVALSVPTLWAADPTDAICCCPANSPLRTIADQTRAFSLSNLLGWGVDLVGRLKRQEWQQVAIDWRNRDEVHAIYRQAAHDLDGYLRRGDHTSYALRTMRETGVTYTDWLADQINTLGRSLG
N1301_04921	5159995	5160339	+			intact	Hypothetical_protein	hypothetical protein;phage(gi509142197);PHAGE_Rhodoc_E3_NC_021347 4.81e-15 MTDLKGYCPMGCGQTLIAFAHEGGRIECSNVDCPSPHAVDRILDDPTPPSTSEWGTDRWKVNLNDRAFEHGHINLDHNGRTMSLTGVPWTLTPADARTVAMAMLNAAAWMDERR
N1301_04922	5160336	5160536	+			intact	Hypothetical_protein	hypothetical protein;phage(gi372449889);PHAGE_Rhodoc_REQ2_NC_016652 1.63e-23 MSTAMLLPDCVLPGCRRPVPEVGQPCDECRSAFGAMLQSGGRPLTSDQIEARDRTVVAAYTRRGFT
N1301_04923	5160533	5160979	+			intact	Hypothetical_protein	hypothetical protein N/A MTLTAQQRAFLFCASPFWIVPALARSGAGVDRLLPSGDGGHGGPIEPSWAAEAYGWMPAGEGPSRRFHIWNSNGPRLELKAEAFGDVLVAVTRQQLRAYARTLPYPVVAELRALDEPRGPRTDDIDRVLETALNLTRPGHESEELTLW
N1301_04924	5160973	5161464	+			intact	Phage-like_protein	gp121;phage(gi29566854);PHAGE_Mycoba_Omega_NC_004688 1.23e-09 MVTEPAPTIPYDEMRALLGLPDVLPADWPFPARDRLYTGLTRGIPWCIYTSPLGCALNGYARIPDGIAIDVDELEVHGGVSYGSGICEGGWIGFDTTHGGDWWDLDELVERVGTHVGPAGIAFNHFEREMAQRFPSLSRHWTVDLLIAECTSLAAQIDERSKA

N1301_04925	5161461	5161691	+			intact	Hypothetical_protein	hypothetical protein N/A MTVGELIAKLTQVPPSTVVVTDDNERFDFAEVYAYTFRGQVERHEGWMHVREDNGRDDVETILLISAFGHEDMEEL
N1301_04926	5161688	5161864	+			intact	Hypothetical_protein	hypothetical protein N/A MTTSQETPMPDQWTEIGAGVYFAPIGTPAPTFTDLPATPRQRVDQTVREWMQSEGLAP
N1301_04927	5161861	5162526	+			intact	Hypothetical_protein	hypothetical protein;phage(gi372450001);PHAGE_Rhodoc_REQ3_NC_016654 1.52e-20 MTEQLNLDRSEVSLLVDRLDKIVEWVAAELENAITCQTAFTNKTITRVGKDDEVPLAFNERASDCAHELLGTLRKWTNYVATEHSLAWPGDGRTPHFARWLSRHAFHLARTDDAARAYTEIIDSFNSAVAVVDRPAEKPSRQIDEAKLDEARRLNLNARACVQMAHTMGAEYADLTQRRVKYLADEKVVAPVWIAKAGKRSERVFLLGDVLDAHLNHPKGT
N1301_04928	5162995	5163186	+			intact	Hypothetical_protein	hypothetical protein N/A MAAPRKFKDTAEYRFDSGCEAKEPNAIQRMHKRGWPIGVIARVMRCTDASIRHALAAPIGGDR
N1301_04929	5163183	5163803	+			intact	Hypothetical_protein	hypothetical protein N/A MTAIDWMPWQLDALDLAASSSLDISTPRQNGKSTIAREVARRAALRGENVLYLTDGRRLALDALHHIEVMQRDSDPSLIRRVIRGYPDRQIHIGAGSITFCSYRSERGPDNQDRVICDATPELAPEDTLIVTTGPLLRVGVQLERGLPRTEMIRYGMDRDDDPASEETWRKANPAIDTVIPIDTMRVLFDALTLDRFKADCLNAPR
N1301_04930	5163800	5164156	+			intact	Hypothetical_protein	hypothetical protein;phage(gi372449903);PHAGE_Rhodoc_REQ2_NC_016652 4.04e-49 MTQTTTEKGLGWQHQKVRGGLLRVHVDGTPCWWCGKPMYRDRTQNWDYEPDSTDRASGSLAADHSHARTHGGTKADRLLHGICNKERQDGSRDHQRPVLRRRRPEAFPWPELPAAEDR

N1301_04931	5164297	5164653	+			intact	Hypothetical_protein	hypothetical protein;phage(gi372449822);PHAGE_Rhodoc_REQ2_NC_016652 8.35e-42 MTTKKSPLRAVADDEVVTPTKPLTVTQAAKSGSHRALLVSMRDRIADAVTNKDCPPRDLASLTKRLQDIANEIEAIDARAEDEAPGRLRDLESALRELDPEHPLLTGMIDDRFDASAI
N1301_04932	5164625	5166145	+			intact	Terminase	terminase large subunit;phage(gi372449823);PHAGE_Rhodoc_REQ2_NC_016652 0.0 MTGSTRRLSEVARHLVIPDGIVTSQFPRVYKRLQDIGVAFDEWQQGFGRVALGCREDGKYAATVGGVVASIPRQVGKTYTVGNLIIGLCLEFPGLRVIWTSHHNRTTTNTFRSMQGMVLTKKVLPHLASNGIRTANGEQEIRFANGSIIMFGAREQGFGRGMDAIDVEVFDEAQILGTKALEDMVPSTNAAKHEHGALLFFIGTPPRPTDDGAAFTAKRKRALDGKSHDQMFVEFSADATADSDDREQWAVMNPSYPHRTPLESMLRMRENIPDEDSWRREAMGIWPDVSEDADKPVVNLGAWSNLYDGEPTFLGVTCLSVDMSTERETRDRTCSIQVAAKTAKGAHLQLGYHGSADTTAVVKFLVAAVEAGDPVAVVIDPKSTAQVLIQPLQRAGIEPELMRPQDVMASTAGFLSATDEGLVTHDDDPRMVEAFAVAKLREIGDGGGVAWARKKSGTICQLVAASNALWGLSQFEPKAVPPPAEVGFEPAPDGGSVMGDLMEVSW

N1 301 _04 933	5166 139	51 67 40 4	+			intact	Portal_protein	portal protein;phage(gi589893334);PHAGE_Mycoba_32HC_NC_023602 7.46e-108 MVSMVEAPSPGEVGYVVANPDAGYRAIDVEDVPELQWPQCLKTYDSMRRQDAQVRTVLKAVTLPILATPWRLDPAGADPEVVADIGEQLGLPIVGSEGEKANPRRLRRFSWSEHLRMALLSLPFGAMFFEQVVEFDPAVRRYRLRKLAPRMPATLADIKVARDGGLIAIEQRPPGGTQVFRASDAGRIPIPVDRLVAYVHEREGSNWGGESMLRAAYKNWLLKDRLLKLEALTIERNGMGVPVYTNPENASPTDIEKGRRMANAYRAGDGSGASLPHGSDLTMKGVTGQLPDPRPAIEYHDAQIGRVALAHFLNLDGQGGSYALASTQADLFTTAVKSVAEMVRDAANRYIVEDLVDWNFGETVAAPQIVFDEISESSLSVATALRTLVDAGVIIPDRSLEEQVRRWLDLPAKDFPPKEAG
N1 301 _04 934	5167 401	51 68 54 6	+			intact	Head_protein	capsid maturation protease;phage(gi508178433);PHAGE_Mycoba_Fishburne_NC_021302 3.08e-67 MSKQQWCRINALAGGGAELLIYDVIGSYWRETSAQAIAEQIKDITADEITVRINSPGGSAFDGIAIMNALRAHDARVVVHVDGLAASAASVIAMAGDEIVMGPGSQMMIHEAWVFTEGDATFLRSEADRLDKMSDSITSLYARRAGGDFDEWRALVAAETWFTAEEAVTAGLADRVDGDAAENGDLAATFNLSRFRFAGRAQAPAPRIPSAEAVEANNRKEGAMPTLKEALATRLGTAADADDDTVVAALDEALAERADTSASGSSDIEARAGVVTVDADQFASMQSEIVASREFRTRQEAAETRAYIDAALAAGKFPPSKAEHYEKLIAAAPAETRALIDGMPANVFPVAEIGHDGDGVGGDGNGGDIRQSAAYKALEGF
N1 301 _04 935	5168 549	51 68 95 9	+			intact	Hypothetical_protein	hypothetical protein N/A MSGINVKFEKGRISYTAEAAVTGGQVVDPGTGKRTVKPAAADSERILGVALTDAAPAASSTPGVLVATTDVVTVASGMGCVPVVSDGSAAVGDLVVPSGGGAVKKAAGTEGVGAIVGRVKELLSDDGKSVLVDLGL

N1301_04936	5168973	5169896	+			intact	Head_protein	putative main capsid protein;phage(gi372449827);PHAGE_Rhodoc_REQ2_NC_016652 8.47e-85 MAKTIVSIHDGNKITVSDLIGAPDVIPTRAIDLVRDNLVAETLFRNGGSPASPVVQFTRSAPVFLDGDPEIVGEFGEIPVAGSGEGQPEVAVGHKLGLANRISREMKDYNQIQLVLKNLTRTTNTFIRANDKLAQKALADAGVPEIPASDTWGGSTSNPRSDIAAAIESVNGALHDNNPNEPYGYVADTIVMNPALWPVLMDDDRFSKLYNGDAASEHIGFKGALPGSLMSCNVLQSRFWPTDKVLVLQRGITGFYADPRPLEATGLYPEGGGPNGGPTESWRSDMTQIRIIGVDEPLSACWITGIA
N1301_04937	5169893	5170312	+			intact	Head_protein	head-to-tail connector;phage(gi971482402);PHAGE_Mycoba_Brusacoram_NC_028747 1.27e-06 MSDLYRLVSKSWQPPGTRDRITRGTVFTPPENQIDRLLRIGAIVAVDGADEPGLDVDTRSPDGSGDESSEGTSADGDADADADAPAGHDQDTEHPAAAETERPKQAAPKAVWVEYAVAGGMDRDAAEGLDKRELIAALS
N1301_04938	5170332	5170694	+			intact	Hypothetical_protein	hypothetical protein;phage(gi372449829);PHAGE_Rhodoc_REQ2_NC_016652 4.44e-36 MAEFATVDEVTRGRRPPTEEDRANIEAMIVAAGIWIRGHRSAIADDDRAANFVVVDAVRTAMDTEKYRGLVAFTKTTGGVSRGGTLANPGEILVFTDFHHQLLGISRVGTPSWHFGGLDG
N1301_04939	5170687	5171019	+			intact	Hypothetical_protein	hypothetical protein;phage(gi372449830);PHAGE_Rhodoc_REQ2_NC_016652 4.73e-57 MDDLGNQTVRVIERVRGKRGVMTERPGAVWPGCSVQPVDSAEGVPLERHTRWVLYAPPGFPESSENVLAVDGIGDRLHVDGDLQTWFDDEGVAEYVWGYLERWNGKGTNG

N1301_04940	5171012	5171341	+			intact	Hypothetical_protein	hypothetical protein;phage(gi372449831);PHAGE_Rhodoc_REQ2_NC_016652 6.39e-39 MANGKPGSIRFSSKDAKAIMASKHVQAATAKQAKVGADAFRKEARRHKRSGQLADKVRVEPAKGWDGRPGSRIVASRRGNQSALFGTSRSRPVRAQEAAIRAMNQRRRY
N1301_04941	5171343	5171756	+			intact	Hypothetical_protein	hypothetical protein;phage(gi372449832);PHAGE_Rhodoc_REQ2_NC_016652 5.93e-74 MVVEIDDTLFPDIEDVLMAYLTPLGETDTEPPDPDEGGRGIQINRVGGHDDGISDYPRVMIVCHDPDPRAASKLARQVANYMERIVDAEIDVEDEPKPIGVDSCRVDTPPEAEPFENPDVRRVVAYYALSLSKPWRR
N1301_04942	5171824	5172510	+			intact	Tail_protein	main tail protein;phage(gi372449833);PHAGE_Rhodoc_REQ2_NC_016652 1.61e-122 MPTDIGKLEVARHQRELILKPKRMHIYGMPMSVDPIQAITEGSSAQLAELPEGAFDFGLLLKDDAITLTREVEKDDINAIGYSNPVMSDFISDVSGIQFTGLEANRYNIQNNLGVDLSAMTADPVTGEVAFDQPAVAEILRLRYLLMAQFNTGVDRIYLARLLYCGEVAEMGEQTLSDAGGALTWPTKVNAMVDPVHGVSVRHFFAGPGWDRVLEDAGFKKGTAPGGQ
N1301_04943	5172616	5172951	+			intact	Hypothetical_protein	hypothetical protein;phage(gi372449836);PHAGE_Rhodoc_REQ2_NC_016652 5.15e-17 MGQMPTFNADGWQPVTLVSPDGRTYTPGDHTEHRQLLASGYREAPAESSPSVPDVAAAAASAAADDPDVKLALDAQQARDAAESAAESAAAATAAAEAPKPKTSRRNGGAQ

N1301_04944	5172948	5173331	+			intact	Hypothetical_protein	hypothetical protein;phage(gi372449837);PHAGE_Rhodoc_REQ2_NC_016652 1.42e-36 MSAKDIDTVTSFEPRNGAAARQYDSTRPKKEPFTFRVADSPEFVVEEPDGYTVMDIEEAKTSRSVLKLFLGDQYEDLEDYIGPLDPDVLVDIARDMSKHFGLFDADSAINRAERRSRDRRRGGRGRR
N1301_04945	5173334	5174143	+			intact	Hypothetical_protein	hypothetical protein;phage(gi372449838);PHAGE_Rhodoc_REQ2_NC_016652 3.50e-128 MNADENGSTASFFETLLDEAVGPFFVSLDDGTELVVEVPAADDVADLDTTTSIHDVLDLLVDEDTADTIADAYARRPISELADLVDDIRAHFALLTPPDQGWAYLVDEIDRYGIAIDRDLFGMPGNARLYDWVRDHEDLPWNNFLRMLPTLPVGGWYYAAIADDDERADQILEMEARGELPPRSNRPSLIGWTPERELMTEVAELLEHVVHGIWGASPKFKGKGGKPPQRRPRPETAHDRAEERDALREHDDLSSQLLGARYARRYSEN

N1<br>301<br>_04<br>946	5174<br>154	51<br>79<br>40<br>3	+			intact	Phage-like_protein	tape measure<br>protein;phage(gi372449839);PHAGE_Rhodoc_REQ2_N<br>C_016652 1.52e-112<br>MADLQGGQTFLNVLPSMTGYFKRIRSEIQGNPIEQ<br>NVDVKINERRLAKAKSDLESMVKAEEAARRKAA<br>DAAGAQTVAEAKLQALREKGVTDVGRLAAAEEA<br>VEKAKRGSEAASKSLAEAESKRKAGDMRVQRIEA<br>QFDGSKADAQSESFLQRLAAKSETSGHGIGSKLVG<br>GIGAAMKTGAIAVGATVATVLGAALTKGFNRLTA<br>IENAQAKLKGLGLAADEIKSVMGDVSASVSGTAF<br>GTNEAADAAAMALSNGIKPGKDLQRTLKLVGDA<br>AAFANKGFGEVAPMVMKVAESGKLGGDVLTQM<br>GENAIPIMAGLQKVTGKTAEEVRKMASDGKIDFET<br>FQKALESSIGGSALAAGDTFQGSMKNMGAALGRL<br>GETLLSAPFKAAPGVFKGITGGIDNINAKTKGVLEL<br>LTTGAFGEAWEKAFPGKDLSNSGLARGIVTISENIH<br>SLFAATKLFATGDFTTDIGRALGVSEDSELVDRILR<br>TREAIEQARDTVKLFFGVIAGDGADVDLPWMNKV<br>IDAGVMVRGWGEQIGRIFGTVKDILVELGPPVGQI<br>VGSLGQASAAIGISTWSLFLDVLEALLPVIGDILVP<br>AVQTLADLMAAHPGIVTLVVGAYTAYRGAVVAA<br>TAAVKVQDVWAKRSMITTKAHAITTKASTIATRA<br>WAIAQTVASTASKGAAVGVRALNAAIKANPIMAI<br>VSVITLLVGALVWFFTQTETGKRIWETVWSGIKAA<br>MAAAWDFIRPIFEAIGAIFTWLYENIVQPVFTGLKI<br>ALAVVITAFLLFWEGVKLYLEMVGAILTWLWENV<br>AQPVWELMKVGLQALGAFFGWVWNSLIKPAWD<br>GLGAGISWVWQNVIQPAWNALKAALQAVGAFFG<br>WVWNSLIKPAWDGLAAGIKWAWENVIRPAWDGL<br>KTALQALGDFFKWIWDNAIKPAWDGLGAGIAWV<br>WEKVIRPAWDAMTGALGKVRDFFGEVVRGIQDK<br>WNGLKAILAKPINFLINTVWNGGILKAWNKAADL<br>LGLDKAEELAGIPEHRNGGAIRGPGTGTSDDVLM<br>WGSNGEHMLTTKEVQRVGGHNAVYALRDMIMR<br>GVPFTWDGGQIIRELGRDNVDAYGAKVAVKGLG<br>NVNPQGMFDWLLPKYKDGGEIRPAWQSQLENGH<br>RAAKMRNGNPYTWGFEDCSGYVSAIADAILNGGN<br>GSWKWATGSFPGGQPWAPGLGEGFSVGVWDDPG

								GPGGGHTAGTLTGVGPYSTTNVESGGAHNYVAYGGPAVGADAPIFAGKSPGLFHLAIGADGAFESAGGPSPQQKTSWLQQKIAGIFDFFLNPIKAGIGAAIGTPPPEWLGIPPSFLDHGRDKASAFLSEKILGLGSLLSSVWGRAKDGFGLFRDQGGWIPNGLSIVRNETGKPEAVLNWEQIMALRDLFETGDYTSALSRVGIQEDHPYVDAVLGLRDLIVNGDYTPAVRRQFGIDEDHPIVGAVLGARDGILAAQANLDAAGTYASEIDWGGVGAQIGTSLLSEWGNDLLGMVGLSNRFEGMKLVDDTGRRRSDEAGARNEASFDDGPTGDPDPAGDSGTAPASAAAPAPVDSAGDPESVVDAVKRAFAPLGWDTGEQWAAVDFIVGKESSWNPLARNPQSGAFSLFQFLGSTKDQYLPDENPDPYIQGLAGRDYIRDRYGDPVRARSFWEKNGFYDQGGIARGKGYLLKDIIHPERVLSPRQTETFEQLIPMLDRMQQSTTAPADVMPASALSSLEYAPVGAEGDTFHVYGKADRETMNEVGIWSNQRTRSRRYDRGQR

N1 301 _04 947	5179 400	51 80 23 0	+			intact	Tail_protein	phage tail protein;phage(gi372449840);PHAGE_Rhodoc_REQ2_NC_016652 1.58e-168 MSLIDSVRARGVDGSVWPLGGPRNLAKDAWIVGGLDALFVPPPFTAARTSRAYQIGSTPRMTKVEERLLDFRVRLQGRTRSDFEQLLAAWNRAWSKDADLDLDTRTETGGTRTISLRLDRQMKPASRISMQARKLDLEMVAVACWPYLRSGIDVEKFKCPAGTHEGTILVSNPTDVPLWVEWGGTPGRWFLPDALSGRVVPIPVQNDVWKVRTRQNFETLSSASGEFVWPKMRGVNFQFEIPPGTPPTEIPVRVENAPTKAELRVFMPRYHQMPWG
N1 301 _04 948	5180 234	51 81 80 8	+			intact	Tail_protein	phage tail protein;phage(gi372449841);PHAGE_Rhodoc_REQ2_NC_016652 0.0 MSVDLDAGLAAIDARFEAEQAASNERDRVFIYDRNLELVGEVFGENDADWEDICNDAGEAAVTIPGEDDLAQWLIDAQTVDHDVFLVFQTPWKRECYKVKDIEIDEDDHGNMVVRPIALHILDEAKHWQCWPNTMAPLAAQFPKSDTQFGNAIKVIKGYAHRNLLREQQPGWIPFFDIWNAANWKANFDNAKWPMVMIPYIAERDGVAEWCALDSRMDNFYDLVIHTLEDAGLQLTGDVWFPGDPQPCPSHFILDRPTLVFDVVRRRVAAGITGTIADPIRDLVRIIAPDGTSETVTIADPNDDPNNADPTAPWVILRRGQHVGLRSKMTIHKPIEHTITTGGKSPDAINQGAKLLSNILLGLLGTAIGMPWLTLGIFDKAVEDVILAWAIFTDHQRKARMGRYSHKSGFETGGGVAVSPSGLQVGRVGLHKARGYVSFAAEVDDAAPHVMGRHIDVGISAGFEVGNRIWLSNISAGRRSWSRTQAPAWQIAVGDYRALEPDGTRALRYIGTLTGAVRQISSST

N1 301 _04 949	5181 822	51 82 21 1	+			intact	Hypothetical_prote in	hypothetical protein;phage(gi372449842);PHAGE_Rhodoc_REQ2_N C_016652 2.16e-43 MPEPDPDPDLNAELLAAIPPDMHPYAYVFMHPSM EGVTPKFEPDEMNALGRHLERLGFRPVAEQLDRY EPPKSGPIHPHNPGKWIPKSRPRSAAAPTVAERFVA AIGQLSEGDRTEVIASLTDSKETSG
N1 301 _04 950	5182 208	51 83 33 2	+			intact	Phage-like_protein	lysin;phage(gi372450115);PHAGE_Rhodoc_REQ1_NC _016655 9.58e-84 MTVTLPPRPAFDEKWMLSLSRSSRFGAAVTNALW HTEEGNATAVALALYCGNPANNASYHDIVRDRIV AHVVDDDFASWSALAANPYTYNLCFAGSRAAWS EAQWMARADDIRIAVWLTLEVARRKGTIATEIIAL GGGRYRGRMSGIADHYYVTKVLGIGNHTDLGMN FPWWFAAQVLAEYLAPAPLPAPVVPAIDEEIRRIRE VNDWLGARITVGENDCRGDRGGKWVKLENGQA YWSAQTGAKIVPNALLDTYGLYDWEVGPLGYPIG DHTVLTEPATGTPWGDVQHFEGGTLYRRYSADRG WWVHGLIYATWRRSGFENGPLGWPTSDEVELDN GDRVQHFEGGDIYWSPTGTVALRPADGPDQHFPIS H
N1 301 _04 951	5183 409	51 84 01 4	+			intact	Phage-like_protein	putative holin;phage(gi966198573);PHAGE_Gordon_GTE6_NC _028665 3.96e-20 MTTSDASITVGVEVVGAHSIDSRAFWLDLTDRTVR TFLQNVLVFLSVGTTILEVSWTTALSSAALAALVSF LLALSTATAISSGNFLIDLADRVGRTFVGALVGAIP ATGTLADIDWQAALSIAATTAIVSAITSILSINLGTA KGLPSLAPVQPDVIALPDTSSGSDATITVGIDPDHV LSREQADQLAGRHRSRRYDGR

N1301_04952	5184018	5184497	+			intact	Tail_protein	minor tail protein;phage(gi971751060);PHAGE_Mycoba_Pari_NC_028874 7.48e-12 MGLSAVDITALLTASGASGVVGAVVNGMFARRATTAAAAKDAAEASKAAAEASRAGTEETKLDAEAAEIIADTAVRLVAPLGERVTRLETEVGELRTENAEIKSENADIKSENAETRTLLTSALDFIRTLLEWIEARIPGQSPPEVPANLRKGVHGDVT
N1301_04953	5184538	5185215	+			intact	Hypothetical_protein	hypothetical protein;phage(gi372449848);PHAGE_Rhodoc_REQ2_NC_016652 7.21e-157 MREMADQFIREFFGNWLSGNYTPLDTFRDGQRDVEWRLDELEKVSGYCNLVMSKNWNVNGGGLNGFARAIPFDTPMGPEPKNARPFKGLFHGSGDERYGILIEKPGTWRLDAQVTTDGKGSLDAGIPAQIYLSAWNKDTRRLYSERRFDATLEYIKVSNTISHTIVVPEALAGKIVVCVSFAHGQGRWMVLGGDRWSGLSVNRWDLDASGPGNDTTDPVDGGNYD
N1301_04954	5185208	5186890	+			intact	Hypothetical_protein	hypothetical protein;phage(gi372449849);PHAGE_Rhodoc_REQ2_NC_016652 0.0 MTDLHADITDIAGGRRDGTATMTSMVLRPAYSRSGTIAPVIPHQVRITDGTFTMTNLDPGPATLEISMGAWVESWSVNIPDADETITLKTLLDNYVEYEPGVVSETRANADRAEASAVRSENAADRAVAAEQYVQGVVADGAAAVRAEVSTNADDARAAAAATAADRAATGEDRAATGADRTATENASSDARSRAEAAAVARGAAEDARDDASEHRQAAEAAAQTASEHGDAARSARAGAESAADTAAADAASIVTTALQQAVAADRQAAETARAGAESAASTAAADAAGQVTTTLQQAVAADRQAAEAARGGAETAATTARGHEDAAEGFAEAADASAQAADAARQLAEDAAENAQQGAPSGGWLRTHLAPDVQDALSRADNALTGVPAATDQARGGIKITGVLAGTAESPDLASGAVNYGHLAEAVTDDIIKGAGIANAVEFYGGWPYETMTAEVKASLDKADSSYQKPATGIPRTDLDAETKQKIDDGYVKPGAGIPAADLAEPVRNDLSKAATAMQGSKNGTPATFKVWVGTEAEYNAATNNGANEAPNTIYLRGA

N1301_04955	5186890	5187774	+			intact	Hypothetical_protein	hypothetical protein;phage(gi372449850);PHAGE_Rhodoc_REQ2_NC_016652 3.99e-178 MAGKISVPGVGQIKKVGAGGMAVKRISSGGAVLWTSSPPRQGVLKSGTQQLPQNNYEKVTGFKIDPEFPDTDLAAAVAANGLLVSGRAAFVAEGTMATSSSANQSRGVQIRGSGAVLGTADGTSATSSRTAKVVVPDGTDVLLELYASAGSSITSYRVLAADTTKLSYRTGGYYEQLAPLTLARDQITEAILTANAATEWPRVPGSGIFLEAGTYELIWGLFTVGWGAYWSVGCRVGDEPNQVVAGATSSNSWNRPVQTITVPSAQFVVPTVKSSNSSDLTIDAGRLNLFISRI
N1301_04956	5188396	5189913	-			intact	Hypothetical_protein	hypothetical protein N/A MDGDCEALTGPGVAAPAVGAPLSLLVDSNVFIAAEDHGGDGHVFGREASELIRLAQKLRYRLLVSNGTRTDLLSAGPDIRRRRTRALEKYDVLPRIDIDEAVRSTFPDELSRNNFADLEVLSALAAGAASWLVTNDSTMRSRARRAGLQAALSIEGALELLRPLVSVEPPVPSTQIVKGYQIRTSSPIFDSLREDYDFDAWWRDKVCAQHRGVIVLGDPTAPEGIGVLKVENDRPYGFPNPTLKVCTFKVSDEFRGSKRGELLLKAVISHARRLAVTNMYMEVLPTKVDLLGWLEGFGFDVAEGARTDRGELVMRKLLRPGPKDPSSTALDQAITYGPGFFRPSRGHVVPIKDGYHYRLLPEADTEEGLFQGNEACGNAIRKAYLCHAPTRRVRAGDALLFQRTGDRAAITTIGVVEEAFVSSVPEEIIRRVGTRTVYTAEEIRDRCSGNRDVLVLLFRFDRTLEPAWSPESTQRAGVVNGWPQSISEVSERGMDWVRKQLDASL
N1301_04957	5190212	5190376	-			intact	Hypothetical_protein	hypothetical protein N/A MGKKLRGFIVSVVVPLGFLAMFTFAGLHQFWGLPQFAADRFVSIFDASSDKDGE

N1301_04958	5190560	5190838	-			intact	Hypothetical_protein	hypothetical protein;phage(gi372449851);PHAGE_Rhodoc_REQ2_NC_016652 6.41e-24 MTRQRGTADVVAWIVLAVAVLGLIGCLTIGAYGGALVWVIVGGVAGLSLAERRRRRSAAAAATIAARADRENELYLDGDSTGLYGQYRPPSN
NBRC101255_02383	2501876	2502016	-			intact	Hypothetical_protein	hypothetical protein;phage(gi372450031);PHAGE_Rhodoc_REQ3_NC_016654 8.67e-27 METVTLPGLPGHTITLEVDGAWLSCEVRDSGGEVRYSAGWDTTPTA
NBRC101255_02384	2502026	2503117	-			intact	Hypothetical_protein	hypothetical protein;phage(gi372450030);PHAGE_Rhodoc_REQ3_NC_016654 0.0 MPIVRNGRVAAALRYIDADGVEHQVVQVRLGDRIVFDGTTPALASVRRFTGSGQVLAPSVQAGQSVALQRSTAVSEFRAMTVSAGARIAMPTLTGTGTAFAPDVTGAAALEVLAAPATGRFLPASGGEIAAGVVYLPAMTAIGSVPPPIAAVPADVEVEIMTAVGEVRLVSVCGQALADAIAATMSGQAFAPSVRNGQRQDVPRSTATSKATAPSVSAGARVGMPVATATGQMRAADTGKPFPMGMDKSGNQSITSFAVTDLIGFVVRSGFPDTNLVGDKLVSGASMTVTFTARVGASGNIGQTVRVVRNGTEVVASGSVNTVLTGTVIFTSATDTLHLQVTNAGGTGVLGGSLNTYLYWTVG
NBRC101255_02385	2503133	2503570	-			intact	Hypothetical_protein	hypothetical protein;phage(gi372450029);PHAGE_Rhodoc_REQ3_NC_016654 1.40e-102 MAHIGKFYGSFLLSAMNKEVDLDSDTLKVMLCTSAYTPNQDTHRYKSSITGEVSGSGYTAGGVTLTGVTVTYDAATNTLKLDADDASWPAATITARHAVIYDSTPGSDATRPLIGYTTFDQDISSTAAAFQLIWDPAGICTMTVS

NBRC101255_02386	2503587	2504000	-			intact	Hypothetical_protein	hypothetical protein;phage(gi372450028);PHAGE_Rhodoc_REQ3_NC_016654 4.38e-95 MTILTGTVRDIGAYDDLTVFKFATPVVREDDSGDGIITTRKVRCQANAGVLTTPDLEPGIAILTIQGDPHPYQITVPDSPTPVQLWPLIQAATPPDPGSWTTGYISNAGGIARAQAVPLTAYPGMVKDPETFYVIFE
NBRC101255_02387	2504003	2504662	-			intact	Hypothetical_protein	hypothetical protein;phage(gi372450027);PHAGE_Rhodoc_REQ3_NC_016654 2.38e-161 MTYPVGPAPDGAYVVGSDYGQSYDEANAMALMTGGVKGAFSGAQDQFKDQFKVFTDGQLALNQRTDLLSPLLDFGSAYMNTNQGFNQTGQCSFSNQIGPMQGCRLSGGRIILDDKGLWDIRCQLWFDFINILTGTIEWQIRVLTPSGNVFSQTRAKLNDSEAVSSTNICSVVVPAPGYQVQAWVSYIAAFRGILGGPDRNRLTVQHISRDTSTGNTGQG
NBRC101255_02388	2504664	2505029	-			intact	Hypothetical_protein	hypothetical protein;phage(gi372450026);PHAGE_Rhodoc_REQ3_NC_016654 2.88e-83 MSGYQTALIGVAAPIVAALFTYLGTRVNRQSTKESNNTEAWAEILKANNEQNARLNAEIREVRTDQNELRGRVEDLERKLEHEQRVRRGAFDYIRILLRWIETHLPGVTPPAAPELLREEL
NBRC101255_02389	2505005	2505433	-			intact	Hypothetical_protein	hypothetical protein;phage(gi372450025);PHAGE_Rhodoc_REQ3_NC_016654 1.46e-97 MYRRIAAAAIGISSIVRGSSYLSPQLPDSSVAQLAFVDETIPLTWYAVGWIATGSIALFAAAWPHPRILGAAFGAGIGFNMLWALSFMASQVFLGVPRAYVSATSYLVIATLIFCVAALSERLKLPPAPRLGGDRCRGTKQP

NBRC101255_02390	2505461	2505733	-			intact	Head_protein	caudovirus prohead protease family protein;phage(gi372449807);PHAGE_Rhodoc_RRH1_NC_016651 2.95e-15 MTSPTQVAHPWRAVARTVFAAVVGLASLIPTVILASGVPPEGLAAQAVAVCAAITRVLADPRVNLFMTRFLPWLSAAPGGTSAGRHRADT
NBRC101255_02391	2505733	2506485	-			intact	Phage-like_protein	lysin;phage(gi372450023);PHAGE_Rhodoc_REQ3_NC_016654 0.0 MTQKQLPYDRGIVRQETGYWCGPASAQVVLNSRGINVAEATLASDIGTTWNGTDFIGLIERVLDRRVPEANYTSVSMPNDPPTGAQKDRLWRDILRSVNAGWGVIVNIVAPPSNYPRGVHGSTSPAYGGGTVYHYMTVMGYDDVARAVWIADSGFSPYGYWMSFEQLTTLIPPKGYCYANVDGPAPAPGGTMEDDKLTAVHQKVTGRYKSRVPGSTYEDDMLGYIHQIDRAVFEQGQQLKRIEDLLKGGK
NBRC101255_02392	2506487	2507041	-			intact	Hypothetical_protein	hypothetical protein;phage(gi372450022);PHAGE_Rhodoc_REQ3_NC_016654 1.37e-127 MQSRIPLQAMCDQSDPKTAFKWALVGLPWAGPQKFTPPSDLADDWSEHLWKLGFRHHPELQELKLIPPPRGQQHPQNATMQWVGIDEPEPPPVVIPDVSSKEYTRNEQAAIAEQLYRDGVIPTPELERDMASVERTFNPADYTPSEVRGYLIGADDRERARVLALEMTGKARPQILNDPRWKGM

NBRC101255_02393	2507041	2508699	-			intact	Tail_protein	minor tail protein;phage(gi372450021);PHAGE_Rhodoc_REQ3_NC_016654 0.0 MGVAAVTTVQTIDFDAVFEQITAKIKTEADERLTPPLIRIWDGDWNLRGICKREIGAEFSFIDNETGVGMLEMPADYFLSEWLAAADERPTSNVHVTMDKDGARWGGRMSELQVIDDERGRRVRVLFEHDYEELKHILAWPNPFMPIGFQFPKVWMQFGRARTALKTTLYVNLHRLEKSPSWVAGNPLTVSAMNAVGDLNTWSQVVKPDLTPDTSVGAIVRSRMKTIHEASRKQVADGQLSWEQRRYLQGDPPPWPGANLRHGTLVWDLIDKSGWNTGTSFGGSIFSGLIRQFTSIGADGLEESLETVADPNVPAAYSQPGVKGSDPSMPAVVYREGEFTGIQSSRYSLKPARDRQIVAGGNSMPMVNETIGAAIEMAGDLTAMIPGVPPLGGVANEILRPLYTDVVLAFGKWDNITRAQRMGWSHYHERFQEGADRAYTIAWLLAMRTGRWETREQHSVTLEVADGAPWKIGQNGFGHYFLGDRIGFSLKAPLAPGRIIVERVSELTLSWDRDTTPTWRIQVGQRKHEDPVLKAFEQMQEFMGLLQDLGVI
NBRC101255_02394	2508681	2509592	-			intact	Tail_protein	minor tail protein;phage(gi372450020);PHAGE_Rhodoc_REQ3_NC_016654 0.0 MGAAPTVELEGVNGQMFTLSGPGAGAEGVTLKTDIQGLYDSPVKPIYNSHAFEKGSTYGGKRYLQRDIQFGVGVLGAAGSWEELDSAWRLAFDYGRTARLWVETESSRRYIDIALAREPEVKDPFKGHALGYTRIVMHCVAADPYWYDKVDTFPWASTIDTTGGSTANGTIVVPFANPTPDPIWAQWMVQAATDARWTIPDYSFGDDRYRRATADANRRIVMPKLLAAEHLYVNTDEAAKHEQVRSNIDTQIYLRMNGTQFLYPIKSGQKRTEFQVSVTGAPAGVGIQLRLIRPYSRPWGLRL

NBRC101255_02395	2509602	2514359	-			intact	Phage-like_protein	tape measure protein;phage(gi372450019);PHAGE_Rhodoc_REQ3_NC_016654 0.0 MSLNVGTLKAVLTLDDSEFNTRIQRVPQALTSLDQAVSQTGARVSGKLGQAGEQGGRQFGSKLSSAATSSASAAGDKAGQGFGQRFSSSLSSAVSNASQELQSKLGVSLPTSAAAIGVTAGAALGAAMFKGWNRLTSIENAQAKLSGLGNSAQDVAAIMQNAMAAVKGTAFGMDEAASTAAGAVAAGIKPGQDLQRTLTLVGDSATIAGMGMSEMGAIWNKVAASNKIQGDVIAQLNDAGIPIVQLLGKELGKTAEETINLASKGEIGFETFRAAMEKGLGGAAQKSGETVSGAFKNMGAAVSRFGALLAQPVFDRAGGVMGGITEGIDGAGKAVKAAQGAFDSIPAPVQSAVAVFAALKLASMALGTEMGQKLTSPVRNAASAIASSTGNISGNVGAWRNLSGELKQTAPALNAVQRNAMALSTGTGAIGKMGTAFMDAAAQAKRMPNVMGAAAASWQGAKSAASGLSNVLGGPLGIGIMAATALWVKHSNEVAKAKAQTEQIGAAVSDTATVLSSTGGKYSEAGQQAAAAALETIKLKDGTTSLGEALSRANVPLDQAAKGLAGMGDAAEKTREQLKDAHEQAADQMGLWDRIKQDFFDLDIRTPFSGPKRNDEYDTEYSDALAAYEKAQKAIKSKQDAIKREAEAGGFKLEVAVDGTVALDGMTESLKEFESNAKGAAAGVDILTGALNKLSDDQFTVHDAQAKLNESFRELKELSGTLGNVGLDGSGLIDTKTKEGATADKAARQAAEGYKELAASLYESGLRGQGLTDALRPQYDQFIELATGMLKSREQAEILAATLGMYPDDIQINLDVKNAANTKTLLDSIGDQFKGFDGTTSTITVKSLTDEAKTALEGVGFTVEQIPGTKEFTIVPNTKAAFDALDGLRGKYDGIPAVKPITIETPGYEGVMAGLEQLGIKVHSDNEKHIVIDDNSPEVRARLSELGIQTTTLPNGKVVITDNSVDIAGNIDRNLNGKTTTGSHTIYVNEVMSGTGTTGTPRAAVGQHYGADGGLRGIGAGAWMLPFRKRANGMLDTAHIVQGQGQGIFAQTPLGNVQYGEGETVWEAYIPGAQSKRTRSLAILKETARRFGYGIINKRDVMADGGMRVDRSAAEAYAKAHNGEGYVYGALDCSGYLSGIYSKLTGKNVRFTTASDFAALGFVPGYDPNGFSVGTNGGVGENGHMAGTLYGTNVESASG

								AGIQYGGGAQGATDFPEVWHLPEASTGGDNPATA<br>SGGGQQADGQRVFVTNWPSGAGFGGGSGSSGGST<br>SPGAGSAPAPTSSPASNPIADAVDGVRALLERGDY<br>TSSLSRVGVEEDDPRVIAALGLRSLLANGEFTQNL<br>RDAFGVEEDDPRIVRWLGLRAAANGQFDQNVRET<br>FGVEEDDPRVAAAFGVRSLLADGQFTSNLRDAFG<br>VEEDDLRVAATLGVRSLLKDGDFTANLRDAFQVE<br>EDDPRVVGLLDLRKAIVDRFPHLQPMAIEPAAPDA<br>PAPTDQAARENPSEVKPYEVKTPQERMAELTEQA<br>GQEWLDSFGLKPGGAIGALIDAGKEPENVRALEQ<br>MVEVLRVIAEDPPVQVVINAKDGDDAVRKWQEYI<br>GGKSLSWIK

NBRC101255_02396	2514557	2515120	-			intact	Hypothetical_protein	hypothetical protein;phage(gi372450018);PHAGE_Rhodoc_REQ3_NC_016654 3.56e-135 MPYNDLREFFDPDLHLPINGKTYTIHTPNAADGLRLRAIFADDEQTLTNTDELAEIAKLFGADIDDDGNPTGGVWQQMNDDGISWGEIIHAGRTALMHYGISADFGAIYWRTGMDGVLGNPLPPNPNGETGENPSQSASAPESAEEPSPADPAPTAPTTPAAAHTSKRTASATGSTPNRSKRPRKAS
NBRC101255_02397	2515193	2515930	-			intact	Hypothetical_protein	hypothetical protein;phage(gi372450017);PHAGE_Rhodoc_REQ3_NC_016654 8.90e-175 MALASTLARDWILEVRPVGGSTWTRVRGLSSVAPIFEGAEQDASDIDSDGYSSPIITGLSYRIEGGGKRKGDNTAGFVDDPGQNFLRRQGRITGADNFIEARIYRRDELPDAYQSTNIVKWTDTAAGDPNALQEFSFTLGGVGKPEEIVKPPITGGSAKTFNVSFGGATAGNATLTWNGKTTANIASSAANTAVASALGALDDGYDASDFTVTGSAGSYVVTVPHGTLTGSGSGLTGGTLTITPA
NBRC101255_02398	2515939	2516388	-			intact	Hypothetical_protein	hypothetical protein;phage(gi372450016);PHAGE_Rhodoc_REQ3_NC_016654 2.37e-108 MTPRPPTSDELEAALAQRLHDMGLADYQPTGAYPTNLTVPAVFFGSMPDKPNGAVLINVYNDDRERDPHTPIYLVQLRFRSISGTRRDTERMANWTFSALDDRVNERSNSQWGAIRVLHCHRHLRAPAEPDMNGRWSRPDSYTIMTNPS
NBRC101255_02399	2516385	2516711	-			intact	Hypothetical_protein	hypothetical protein;phage(gi372450015);PHAGE_Rhodoc_REQ3_NC_016654 1.25e-74 MPVEFNYGIAATVRGAAKSGLHDAAEVVKQEAIERCPKETGALRNSAGTASDGMEAVVYFDTPYAARQHEEVGWHHVDGQAKYLENAVNATQATVAEVIGEAIRRSIA

NBRC101255_02400	2516720	2517052	-			intact	Hypothetical_protein	hypothetical protein;phage(gi372450014);PHAGE_Rhodoc_REQ3_NC_016654 4.20e-76 MTDPLEHWWRWPVTVERLAGYGADGPVFDPKDTSLRGKITNKRKKVLAPDGAEVISEARVSMPATTPLIPPGSRVTLPDPFGGRTAEVLAEQLHHDGAGKTPNFYSIDLT
NBRC101255_02401	2517049	2517459	-			intact	Hypothetical_protein	hypothetical protein;phage(gi372450013);PHAGE_Rhodoc_REQ3_NC_016654 1.86e-94 MLVFATEDQLSSWMGTPGPLNAGVLLREASILVRDACRCDVFDTLPNGLPVDDDKREALQDATCAQAAVWAATGDDPLKGPGGQEPRMTLSGIDGAQVSFDTYLTAGDRAKSLTDLCASAYRILRSAGLASSAVQS
NBRC101255_02402	2517462	2517674	-			intact	Hypothetical_protein	hypothetical protein;phage(gi372450012);PHAGE_Rhodoc_REQ3_NC_016654 4.07e-45 MAYAPTIIPRSVIKGWMEAAGIQWDDLRHIDIDPHRVELTYFRRDADGRHVLAGDSIATEKITVGIGKED
NBRC101255_02403	2517886	2518290	-			intact	Hypothetical_protein	hypothetical protein;phage(gi372450011);PHAGE_Rhodoc_REQ3_NC_016654 6.05e-91 MATINAPVKDFTGVVAGVHFADGQAETDNEAAIQYFERHGYGVDGSADDASERKPAGNLEEFSVSKLREYAKAKGIALGDATRKQDILAKIEKAESPIALQEGADPAAAAAIERESVADQMDDAADEPAEPAED

NBRC101255_02404	2518335	2519339	-			intact	Head_protein	major capsid protein;phage(gi372450010);PHAGE_Rhodoc_REQ3_NC_016654 0.0 MAVSLAESKNNAVQDYDPAVIDEFRKESAILDSLIFDDVVNPAGGGATLDYGYRRLITQPTADFRALNTEYTPANVTTQKYSTTLAVLGGSFEVDRVIAKIGANASGTVALNMSQKIKAARTKFQDAIINGDTAVDANGFDGLDKALVGTTTEFNAGKVTDWTDFDTNPRAEHKALDAIDEFLSLLDGAPTVILGNSKALARVRAAVRRAGQYTKDPVEGLLGPGGRPVVRETYGGVLLVDPGAKAGSNNPIVPVATRTVATVSTTGLTDLYAYRVGLDGFHGVSTVGGQLVESWLPDFSSAGAVKKGEVELGPVGVALKSTKAAAVFRNIKVQ
NBRC101255_02405	2519349	2519948	-			intact	Hypothetical_protein	hypothetical protein;phage(gi372450009);PHAGE_Rhodoc_REQ3_NC_016654 6.29e-139 MANLIDDLRFLVGAGFGVGPSRITRHPRRDDEGHDTGNKVDATDTTDDDGDQNETGDVDADAGNADDDSSKDAAHWKAMARKNEREAKANRAAAAELEKLKQAQMTDAEKQAATTKATEERAAAAEAKAAVLEAALEHGITDKKDLELLEGLPADKVAAFAKRLAAQKAPAGRSGNPVDGEKPKPKPTTLTGAIGAHYA

NBRC101255_02406	2520100	2521878	-			intact	Head_protein	minor capsid protein;phage(gi372450008);PHAGE_Rhodoc_REQ3_NC_016654 0.0 MALDPSEAAGIPDELIAMYSDAELALLAAMTQAILEGIDTPDWEARQPLEMLRFRQAAEAIAAQLQAQMPAMVTAAVTAAAAKGVAAADADLADVPNVLPKPPEGYLPPRRTQQQAREAWVTLAQFTQRIPGNSERLYQDVVSRVQVRDVPASGGTRLDAVQEALNHLTKRGITGFRDNRGRNWSLTSYLEMKSRTIVNQTLIDSHTDRMVERGQDLIVVSSHRNPAPQCQPFEGQVLSLSGDTGTVIRPSAVGGRGVKVRIKATLEQARAAGFQHPNAVLEGSTFVTYGEAIEVVRSRYSGPAVTLSTGYSETTIGFNHPVMTVRGLIPAHDLKEGDQLVYDLRCVDGSGFASLDDNLHEVPLVEDAYKALELASGYTRIAATSHDLHGDARFGEGEIEVVRPERGLLPVLDPALAELLSEDALMRSGVEDHVLSRLRSQSATRERILVAAASGMGGSGVGGSHSFVTLPLERVTVKWWDGWAFDSTAELSLYCSDGLVVSNCGHAVSAFVPGASRTFKTEPNPEGYEATQQQRAMERGIRDTKRQLAVAATPQAKRELNARLKAQREAIRDHIDEWDLKRRPKREQLGAR

NBRC101255_02407	2521882	2523483	-			intact	Portal_protein	portal protein;phage(gi372450007);PHAGE_Rhodoc_REQ3_NC_016654 0.0 MSLPEANTAWPPPELAAVTARVAESHVWWEGDLDKLATFYGAEGRTSPSGIKARTKAAYEAFHGRTPTATGRAPKRYHAPIPGVIAKLSTTELFSEQLKFLDAGKSKEVQARADLIFNTPRFHSSLVEAGESCSALSGSFQRIVWDPTIADNAWIDFVDADRAIPEFRWGRLVAVTFWSELAGGDGQEVWRHLERHESGYIVHAVYKGTATSLGWMMALTDHPATRDIAVEGADEGRGAYVETGVKDLTAAYVPNVTPNPEWRHDPKLRYLGRADLSTDLFPTFHELDRIYSSLMRDFRIGAGKVHASESVLTNLGMGQGVSLDEEQEVYSRVGSGGFNANGDMETIFEFFQPAIRVLEHDQGAALLLREVLRKTGYSPVSLGLSDEVAQTATEASGKKDLTVKTTRAKARHFGSALGPLSTTCLRVDAIKFPGKGAAPSEELELEWPKFARESDLAKAQTVQAWSVASAASTKTKVAYLHEDWDDERVQEEADLIDNANTVSAPTFGFGTDQPPLPTENDPATDPEAVDEGE
NBRC101255_02408	2523480	2524778	-			intact	Terminase	terminase large subunit;phage(gi372450006);PHAGE_Rhodoc_REQ3_NC_016654 0.0 MTLDGLAVSAKQLRSIALANADASKGRVNIWHGAVRSGKTIGSLVAFLAGVAAAPEQGEIVVIGRTRDTIYRNLIGPLQNVAMYGTMVEHIRYNRGAPTAEIFGRTVHVIGASDVRSEAVIRGMTISLAYLDETTLVAEEFFNQLVARMSVPGSRIFTTTNPDGPRHWLKVNYIDRAEERGHKVFHFNLEDNRAYLPEGYIEGLEAQYTGLWHDRFVKGLWTMADGVIYECFDPARHVVDTLPTMQRVLAVGVDYGTTNPTRGIKLGLGDDNRLYAMAEWAPGTGTTADRETGLRAFCATDRPDYVFVDPAAAEFKVQLQRGGWGGGHADSPPPYANGMNRVGAGIGLVSALLSTDQLLIHSSCTELLGEIPGYVWDTKAAQKGEDAPVKLNDHAVDALRYAVATSRPMWQPFLPQIDAAKRLPDERIEVAA

NBRC101255_02409	2524775	2525272	-			intact	Hypothetical_protein	hypothetical protein;phage(gi372450005);PHAGE_Rhodoc_REQ3_NC_016654 3.90e-116 MAEELTPEREATIREGHALGRSQADIARELEVPQPIVSRWAKRMGLLWSISPNVQAMNDKVRERIALERALLAEAAIADAKAIRERIWDEYEIVASTPAGPQRMTLDLPDAKAVGDFASAVQKLVMTHDNLTRMGAGSSADHAKSMLNQLMEQARRLVNEEGEEA
NBRC101255_02410	2525312	2525497	-			intact	Hypothetical_protein	hypothetical protein;phage(gi372450004);PHAGE_Rhodoc_REQ3_NC_016654 3.52e-39 MDAIGAITSITAGVMRRIADSIEGTPAPQPQSVVHYHFHLSQADSLDKPEDKGRGFTLFKR
NBRC101255_02411	2525499	2526212	-			intact	Tail_protein	putative tail fibre;phage(gi372450003);PHAGE_Rhodoc_REQ3_NC_016654 2.95e-170 MTIEISDGVTVTVNVPNRMPTICPAATPGGPITVPVQGGLTAEQFAHIAATVTAKVVEELGGQPGTVTWEAVQEKPTAFPPETHEHPIDEIDGLRTSLDGLASDVANRSEIGHRHNASDIDDLPSGGGGAGIDDATIGTDTTWSSQQITTDRAPGMWPVVDQSSGEVIAEVPILDGMNILYFLATGSIVWEVASGTARPNPSVKLVSSLPTFPSENQIYAVRGEGGIVTWHMPNIGG
NBRC101255_02412	2526202	2526501	-			intact	Hypothetical_protein	hypothetical protein;phage(gi372450002);PHAGE_Rhodoc_REQ3_NC_016654 2.63e-65 MDYTDIGAPQQRIRIWLKRGSPLRFALSPVTLPAGATVTLEAGARSFPATVTADSVSWRIEPADADLIPDRAVVRIRCMFLDDPTTVEPWLKGVVARDD

NBRC101255_02413	2526757	2527437	-			intact	Hypothetical_protein	hypothetical protein;phage(gi372450001);PHAGE_Rhodoc_REQ3_NC_016654 3.27e-161 MPVADDQFFMPRHDQAALVAILRTIPGLIEDLAVTITRQDRLTTGRPRISTGEQEQPLPFNVNASDAGDYLHFTLASWVRLVLDQRGMEYDGTDATTSVAAWLDRNIIALAMTEGSGTALDEIRAAVSQATRACDRPRDPRWMQASVEDAANTRLNARGIEALAKELGGEWAGITRDRVRTLHRAKRIAPVAVDEGSDEQLMFAAGDVLAAHIAFPAGPSRKKRSA
NBRC101255_02414	2527424	2527756	-			intact	Hypothetical_protein	hypothetical protein;phage(gi372450000);PHAGE_Rhodoc_REQ3_NC_016654 8.71e-74 MTIDLDKLEGELLDQYGCSDLEELANYHGTAHTMLAELRKSQRSIEWLQTLAEKHSLDSARSERELQEAQATIERVRALAYDPDDADEYAATAIVVDATLLQRALDGARG
NBRC101255_02415	2527753	2527965	-			intact	Hypothetical_protein	hypothetical protein;phage(gi372449999);PHAGE_Rhodoc_REQ3_NC_016654 3.75e-46 MTNTAPIIGYIVVSKRSRGDGGFGFIDVSPLCTERAHAEINRNGWLERATECPGRYRDTEFIIAEVRGNA
NBRC101255_02416	2527962	2528333	-			intact	Hypothetical_protein	hypothetical protein;phage(gi372449998);PHAGE_Rhodoc_REQ3_NC_016654 1.18e-89 MNQRDELASLTETLHAHRELWNIRGCFAGCSCGWDGFQKHHRRHQADAILAAGYSRPRVVETIEELAALPEGSVVMWDDYGNQAVAILGDEDYIEHTSNDYWNSRMDCIGLPATVIHQPEDKP

NBRC101255_02417	2528343	25287789	-			intact	Phage-like_protein	single stranded DNA binding protein;phage(gi372449997);PHAGE_Rhodoc_REQ3_NC_016654 2.28e-104 MTDTLITIIGNLVADPELRFTPAGAAVANFTVASTPRTFDKQANEWKDGEALFMRCQVWREAAENVAESLSRGSRVIVTGKLKSRTFETREGEKRTVIELEVDEIGPSLRYATATVNKANRGGGGSSRATKPAEDPWGSTPPADAPPF
NBRC101255_02418	2528786	25294000	-			intact	Phage-like_protein	transcription factor;phage(gi372449996);PHAGE_Rhodoc_REQ3_NC_016654 2.19e-150 MTRPRAQGFHDFISTNTHHPATNGYPEQVGDPVGLIVTILRDTPRLDGAACAGSTNPDMWFAPPGTTERAQAQRICATCPTRQACTTLGDTNDEHGVWGAHNHDSTANPDMTGHCDTGHSLAEHGRIIRPRNSNPYVRCLACTPPPAPPAPKTHCIRGHEYTPENTAIVRGGKSRACRACKRERQRGYRAAEQNTELKALEAIA
NBRC101255_02419	2529528	25298875	-			intact	Hypothetical_protein	hypothetical protein;phage(gi372449995);PHAGE_Rhodoc_REQ3_NC_016654 5.82e-77 MSAAEQIIADSLKALGAIDYASVAAHVVAALTNAGKVVVDVPSVAHKGPFDTDAAFFRQVAQNLDRDLCAGGSNVRHAVSTLLRNVADALTDDRRTTSEIRGSAARAAEGGERRG
NBRC101255_02420	2529937	25305593	-			intact	Phage-like_protein	endonuclease;phage(gi372449994);PHAGE_Rhodoc_REQ3_NC_016654 2.79e-162 MKATRVCCIDGCDRSITPDDYHKICPMHRGRLRRGGDLTAPARIFGNDEKRFWSKVDKSGECWIWTAALKPNGYGHAWFNKRSQYAHRVSYELTFGEIEAGMVIDHMCRNKACVNPSHLDAVSNAENVRRGLVESVERGQETVLTHCPHGHEKSPDNVYVVPGTGERRCRTCIRADDIRTRAARRERQRVAREAKRLRLADESMRALAPFVRVDGDKA

NBRC101255_02421	2530692	2531174	-			intact	Hypothetical_protein	hypothetical protein;phage(gi372449993);PHAGE_Rhodoc_REQ3_NC_016654 2.42e-112 MTDAITPASLRAHADWMVGAGQDRSGQLMRQEAARLEAESARDEEAEFYAKVYHRTRRRKSPVLAKWDSLPDDARADNIHIMRVVLDRLAADGRLLPEGATVLTAEQAAEARELLDAVDLDAEEFHLLRAAFTPPAVSVPDSGPDGTPEKPWETRCGGEK
NBRC101255_02422	2531171	2531491	-			intact	Hypothetical_protein	hypothetical protein;phage(gi372449992);PHAGE_Rhodoc_REQ3_NC_016654 8.86e-71 MTDNTRELIADRAFYAVPSVSTEESWRIADAVLAALHPEITTAEELDALPTNSVVIDAGRWIHERWERAVPDDHGWVRVGWAFNEPADPPNLPARVLHRPDTEESR
NBRC101255_02423	2531488	2531676	-			intact	Hypothetical_protein	hypothetical protein;phage(gi372449991);PHAGE_Rhodoc_REQ3_NC_016654 3.02e-40 MLVCDCGTFDRRHDYGCKGWRERETPAATFPSWALEARTDSTDDPWGWAARGITNPDKETQR
NBRC101255_02424	2531676	2532038	-			intact	Hypothetical_protein	hypothetical protein;phage(gi372449990);PHAGE_Rhodoc_REQ3_NC_016654 5.21e-83 MSNPNKAKGDRAERAVRDYLRANGFPHCERTRAGYTRDAGDLHPAPGLTVQVKDRAQYAWREWLRQLDEQRDEAKADHAVLVVKRRNLGDPGQWLAVMPLAAIARLLRDAGYGDVLEEVA
NBRC101255_02425	2532035	2532157	-			intact	Hypothetical_protein	hypothetical protein;phage(gi372449989);PHAGE_Rhodoc_REQ3_NC_016654 1.85e-20 MSFERHLQVQLDRLGRDDRDLDDDTTDHDYDRYTDGEYAL

NBRC101255_02426	2532154	2532504	-			intact	Hypothetical_protein	hypothetical protein;phage(gi372449988);PHAGE_Rhodoc_REQ3_NC_016654 8.77e-79 MRGIQREAIDAYLDGIKAGKPADHGVFGLMLTVNDPTLRRWLIAVRLHERITEHRAATAELVTNDNPVQDTVVDVLRYRVEDRIRYATVRLAIDGDLDDVMCVEMPLHDLEIRRAA
NBRC101255_02427	2532501	2532770	-			intact	Hypothetical_protein	hypothetical protein;phage(gi372449987);PHAGE_Rhodoc_REQ3_NC_016654 2.99e-58 MQTFIERGEVIALDCSHQDTILFYDPSYGPYEKCVECEVILDEEDALADNDLTDEEVVADAGVWVAIWLSALAWTAVVGLGLVWWAVTA
NBRC101255_02428	2532998	2533492	-			intact	Hypothetical_protein	hypothetical protein;phage(gi372449986);PHAGE_Rhodoc_REQ3_NC_016654 9.82e-115 MIADRDIETAAQVLAKCAANDPWFPKGGHAIVQAWAEVFAESKLSREDLLAGVARAYRTEETGYRPLPASIVKHARAAYFEALRDLPEEERERMDHVNHALQDMGFAPPAAHRFARRIALGRRPEVGLTEDEKVELRRRLAERAAIEEAPRRAVVVSPNFGKRA
NBRC101255_02429	2533489	2534316	-			intact	Hypothetical_protein	hypothetical protein;phage(gi372449985);PHAGE_Rhodoc_REQ3_NC_016654 0.0 MEKVWLASTYYMDPKLAGCSPAVEAMFTRLLAYAGNAETRGFLPENCHVLVAFPRKKCAVLELVSRQVLSVVEGGGYCFTAWADWQKNGDELLERREKDRERKRRKRAEEANLSADTSAEIHPQRREEKRREPTYVGSSPSVSDAPEPPKRSRGMRAVDAMNETARPLEVYRFMHEYEQASQTPIDQKTLSQIETAVTPLIAQGIPPEQVAAGIRAWEASNSFSATQIASFVHKAGAKASAQPAAGNAHDAKVVGYLDIGRSLTSQSTEARRELA

NBRC101255_02430	2534316	2534591	-			intact	Hypothetical_protein	hypothetical protein;phage(gi372449984);PHAGE_Rhodoc_REQ3_NC_016654 6.08e-59 MKPSHALPPPVDGLESDIDVVARFANAIVESVHTTDPRELLHELAILAEGSPARTAQIMMALAAWVNIDEPLSVRGARVEAITAPAQGRAA
NBRC101255_02431	2534683	2534979	-			intact	Hypothetical_protein	hypothetical protein;phage(gi372449983);PHAGE_Rhodoc_REQ3_NC_016654 6.76e-66 MSLTADDCLASEVAIHRTWIDHANAALDQCIRGAVPFSADTVRALIPDGIDAHHPNALPALFRAASAARRIVAIGYTTPKRASRHGNPNRIWIKGPNA
NBRC101255_02432	2534976	2535818	-			intact	Phage-like_protein	Rect-like protein;phage(gi372449982);PHAGE_Rhodoc_REQ3_NC_016654 0.0 MTTTEIELRQQDSLAEKIEWSKAMATGDMLPRQYRGNPANLMFACEYADALGIPRINALTSIHVIDGKPTASADLIASLVRKAGHRLRVEGDDTYAEVTIIRADDPDYIPTPVRWDEAKARKAGKWGTKGPWTNYPGAMLRSRAITEAARMWASDALFGVIYTAEELGGAIDEEGRPAPAPRQQQRSQRPTSISEALAPEPEPVVVDMESLMDAAARLDTYEALLALWDANVANLTEEQAEELKAFMKARKAQLAAESESEPEPADEPTLDDAIDAEVVA
NBRC101255_02433	2535815	2536129	-			intact	Phage-like_protein	endonuclease;phage(gi372449981);PHAGE_Rhodoc_REQ3_NC_016654 2.91e-74 MTVHGIGKLAHRCSYESFVGPIPDGMTVDHMCFKPACINPDHLRVLSLSGNAAAQRSALTTHCIHGHEFTPENTYIKPGFRNGKRQCRICQRAAVARYKERKSA

NBRC101255_02434	2536215	2537150	-			intact	Phage-like_protein	endonuclease;phage(gi372449980);PHAGE_Rhodoc_REQ3_NC_016654 0.0 MNLTGIRLDVDLIPGSPEWLATYSASQVAAICGLSEWDTPRSIYDAKKGIVPPQPQTDVQGRGHQFEPLIREWVAEQNPSWTIYETGTWAHSERTWQTANPDGVIGLADRDAPIELLEIKTAADMHEWGDVPPIKYLVQCMWQMDVIGARRTHLAACGPFELFHRRPRMFVIDYNPREAVMLREKVLEFDAMLRAGIQPPANHDRECDRLAVRYGNTAIVDDPGVEIPDELAELYLGPYVEKADIEARAKSGASRLLEYLGESKKATYRGTTIATRIAGRGDKPPTLRAANGLAEKAADLLNANESDSNAA
NBRC101255_02435	2537147	2537419	-			intact	Phage-like_protein	endonuclease;phage(gi372449979);PHAGE_Rhodoc_REQ3_NC_016654 7.56e-61 MHDKNTAETLDKAIARLNELLGGSYWQIEHSEALFHEWLVAVPLPDGITHPDTEIGTRRNWIRTQGRDLAAMLSDAADKVAEALAMRGAA
NBRC101255_02436	2537477	2537680	-			intact	Hypothetical_protein	hypothetical protein;phage(gi372449978);PHAGE_Rhodoc_REQ3_NC_016654 7.12e-42 MSNPRNWARHIADIPTTNDHEIAIGAQFHVDDDGTEHRLIVIDGHAIDISQVGDLSDALNRAVAAHT
NBRC101255_02437	2537677	2537970	-			intact	Hypothetical_protein	hypothetical protein;phage(gi372449977);PHAGE_Rhodoc_REQ3_NC_016654 6.08e-65 MPATLRIRRDKLAEAMVAAGMSQQKHLATAMDMSEPSIHRILKERDIQGKTLARLLAALPDANFYELFEIVDEPEKSRIRTPFQELLRAAGTEQVAA
NBRC101255_02438	2538233	2538664	+			intact	Hypothetical_protein	hypothetical protein;phage(gi372449975);PHAGE_Rhodoc_REQ3_NC_016654 1.85e-97 MCATVTRMTNFADWLWTTARRRGLETEAEISRAVNTSQSIVGRWRLQGAVPEIKTLRRIADGLNVPIQEALVAAGYVLPEEVGVVRTDIKDIPAAELLANIATRDLLLELARRLGEDELGGEVAAIRSARPLNPDEPTWRPGK

NBRC101255_02439	2538606	2539016	-			intact	Hypothetical_protein	hypothetical protein N/A MPTSSTGPVYFMCAPTRPPDEDLAVLGIALEQPHGVITHRIVRKQLIVEIGKLVVIGSTIHAIPKAGLTARQHAQLIGAATVAFLADCTVNPGWEHSIVDGQPRWTAWISVPAETISATSLAAMSAHLDSADAQTV
NBRC101255_02440	2539638	2540876	+			intact	Integrase	phage integrase;phage(gi372449972);PHAGE_Rhodoc_REQ3_NC_016654 0.0 MPRPSLPVGAHGRISRTKLPDGRWRAACRFRDADGVTRQVVRYTPPTVDRDKTGAAAERALVDALKGRSTTGDLSADSRVSELWMAYRAQLEEKNRSQSTLQDYDRMAAKILDGLGNLRVREATTQRLDTFVREIATRQGAGTGKKAKTILSGMFRIAVRYGAVQANPVREVTDLGAGRKKRAKSMDRELLVQLLADVRGSEAPCPVVLSEAQIKRGVKTTSKAGQVPSVAQFCQAADLADLIVMFAATGARIGEVLGIRWEDVDLKKRTVAIAGKVIRVKGDGLVREDSTKTESGLRQLPLPGFAVEMLEKRLVDRTGPMVFPSKVGTLRDPDTVQRQWRQVRAALDLEWVTTHTFRKTVATILDDEGLTARQAADHLGHAQVSMTQDVYLGRGRTHSAAAAALDAAVAKR
NBRC101255_03941	4159955	4160845	+			incomplete	Integrase	integrase;phage(gi589893371);PHAGE_Mycoba_32HC_NC_023602 1.42e-80 MTALLPPEVAQGVPDTPRLGRGLDDYEVWMRGAGLSDRWIGDSLATLRQLERTAGSPAADVEPVEISRFLGRAHLSANTRSTYFGQLASYYRWTADHGGANPMQKIRRPRVPRSTPRPVSDAGLNRLLAQRMHRRTRAMILLAAFAGLRVHEIAKFRGEDLDLEERVIRVQGKGGHRAVLPAHPVLVDIAASMPTAGWWFPANSTRPGAHIHGRSVSQIIGQAMRRADVPGTPHSLRHWYGTALVRSGTDLRTAQTLLRHASLATTEIYTAVADSGRAEAIGRLGVGIRAVEGPGV

NBRC101255_03942	4160848	4161234	+			incomplete	Hypothetical_protein	hypothetical protein N/A MTALPVDDHVEAPQPAHRFEWERVMRRVSMPAGVKGLAFVLASYADGDGTRIHPSNARLANVMEKSEKTIERGMTWLVAAGFVVITRRRNSRAGIANEYRLTLPVDVLDRMVLDPDEKPIVLPDMGVG
NBRC101255_03943	4161653	4161901	+			incomplete	Hypothetical_protein	hypothetical protein N/A MTTRRGGRTEPRIVPLLIAILSDRPNLPDAVCRNDFTLFDAALHAENPGARTTAQIAAERLCRQCPHTTACPESVITRRTPA
NBRC101255_03944	4161898	4162248	+			incomplete	Hypothetical_protein	hypothetical protein N/A MKNLLDNVRQLTERRREARGEELSTRTTPITPWTELPDDPVHPTFENATRYLIAQRAHQIHHDARDDYEGVPITATYIDGLGDQVELGPWSLSRREARELADSLHALADALDAQLE
NBRC101255_03945	4162591	4162920	+			incomplete	Hypothetical_protein	hypothetical protein N/A MTDQSEHYPAATPLTGEELRQYAIATVAHAVRARQAQQRVDTQTTKTAADEFAALDETIRLALRSYVQPRVFVREVLAVVLEQCPELVGRLDALLDQALIHADVTGQWP
NBRC101255_03946	4163309	4164304	+			incomplete	Head_protein	capsid protein;phage(gi589893337);PHAGE_Mycoba_32HC_NC_023602 1.90e-40 MTAPAHQPTRAERRRAGAAIKAQIIARRSGQKGTAQMTRSSTSTIYSGIGGGATVNHVLAQPDIIAAKIGDNLTTSVTSAVFGDATSQAVGGAILYQTVHPGDEFTDGDITARAEGAEYQLVRLPEPDVRRDPVEDLGGKFICTEEAITRNVLPVLTNGITQVSASLRKAIDGRTIDTLEAAVAEYSLDLPGHNWSTYIGGGATPTPNAQSPLADLVQAQLSFDVDGMGAVTSTLIVHPEQAAALRIAYGPDLADVLDAAGGVTIHASTHIGPGDAYVIAGQPGGLGVETPLTVQVIPKPESRSMLVQAYIVARPYISKPYAIRRLTGLAG

NBRC101255_03947	4164333	4164701	+			incomplete	Hypothetical_protein	hypothetical protein;phage(gi589893336);PHAGE_Mycoba_32HC_NC_023602 2.90e-10 MGTTPQLYAPGADITAHAATDLEAARFVEVAGPRATGNITVRHAAAGSRVLGVTGRTTKLGELTHILRGSRIVGVAAVGTIAAGADVAAAGDGKATTAGADAIVVGQAVDTSSEGIVYVALA
NBRC101255_03948	4165001	4165321	+			incomplete	Hypothetical_protein	hypothetical protein N/A MHANFGLYRPMSIRALALPPHKDDEGDDVDGSITIYLFGDSEYQEPSVNLSMTIEEAEELSQQLANLAKSCRNGEYNAAGHQLVEYSKAKDLEKARRILGEEASTN
NBRC101255_03949	4165502	4165828	+			incomplete	Non_phage-like_protein	ECF RNA polymerase sigma factor SigM N/A MVNACLDRIRRNRVRQTVSLSNDDAEDPGDDRDRISERETSILVDQALQRLSPDQRAAVVAVDMEGYSVAEAARRLGVPEGTVKSRCARARTKLASHLEYLRDVGNRS
NBRC101255_03950	4165893	4166660	+			incomplete	Non_phage-like_protein	Anti-sigma-M factor RsmA N/A MTLHDDDAVLGPPFSTDLLADLHAGVLPDAVSDKLWPLVRQDPEAVAVLDALDAVSARLAEVGRDHSVETPIPHDVAARINSALGLNVSAPRSDVVPLADATAKRRRMAWLGVAAASTAAAVAVVFALTGVDRSGSTGPEAVASATTTAPDVAPARVELSGELDRGQLLALVGDTESAADGVGALARPEVRSACLSAVGVGATRPVLGMRAVRFQDTDAVLLLVAGPTPPTLLALVVGTGCDATHPDLIDSTEIG
NBRC101255_03951	4166667	4167284	-			incomplete	Non_phage-like_protein	Bacterial regulatory proteins, tetR family N/A MSAHVNGSSVQSGRPRDPRIDAEVLAATRDMLVEVGWDQLSLRAIATRAGVSRAALSRRWPSKAHLVLDSILGSTPDLAPFEGTDRAGWIDWVVAGSAQIFSRPDVRAAAPGLLAALRDHDDLRKALWQGFSGPAAELFAAHAGPGESEEERRRDLLDARAVLVMAAGSALFSSLIVGDDATPELRARILELLAPVAERRPQSER

NBRC101255_03952	4167510	4168637	+			incomplete	Non_phage-like_protein	FtsX-like permease family protein N/A MFLALRELRFARSRFGLMGGVIALISVLMVLLSGLSSGLVEDGVSGLKATPVDGFAFNEGTKTDSAFSRSVVTDTQVEAWREQPGVEEAAPFGNMLVNAHNDKDVPVDLALFGVPVDSFLAPTAAQGTGLGDPNGIVVSKSALDEGVALGDTVTIDRLGTELKIVGITEGKQTFGHVDVAYLPIEMWRQLHSGVRPGEPVPAQSMTESTAVALKTEPGATLDYAAGDAAAGTSTVALEDSFASSPGYSAETMTLNLIKVFLYAISALVVGAFFTVWTIQRKHEIAVMRAMGASTGYLLRDGLAQAFILLLGSVAVGAAVGYGFGSLIGGGVPFLLEPGAVAVAAVLLMVLGLVGAAIAIVRIASVNPLNALGGQR
NBRC101255_03953	4168634	4169359	+	7481|vfid|8848|vsiid|10766|ssid|putative ABC transporter ATP-binding protein [Bacteroides thetaiotaomicron VPI-5482]		incomplete	Phage-like_protein	ABC transporter;phage(gi371496158);PHAGE_Plankt_PaV_LD_NC_016564 2.37e-43 MSAGKDSAVTTSAETRIDTAALEIVDANLTLGDGEQTVTALDGVSLTVRPGEMVAVVGPSGSGKSSLLAVAGALTTPDSGAVRVGGHDLLTMKKSEAAKFRLEQIGFVFQSGNLIPSLTAADQLRLVSHLGNTKGRKFRDPLALLEQVGLAHKADRRPDQLSGGERQRVGIARALVSTPSLLLVDEPTAALDRARSHEIVQLLARETRESKVGTVMVTHDYDVLDYCDRVVEMTDGRLTPR
NBRC101255_03954	4169390	4169983	+			incomplete	Non_phage-like_protein	Fatty acid metabolism regulator protein N/A MPRINAPTVGEHRAAQQRALLDAAREVLAEGATDIPSFGEVAARAGLARSSMYQYFKSRQHLLEALIEDAFPRWSARVTAAMAHAGAPERRVLAYIDANLDLVAEGEHAIATALAQLAPGPEINAKSRAMHDDLYTPLSDALRELGASDVPAMSELINAVLHAATRQVERGEDVEQVRRTAADLVRPYVADLARRAG

NBRC101255_03955	4170190	4171179	+	thioredoxin reductase;2935|vfid|3610|vsiid|4172|ssid|thioredoxin		incomplete	Phage-like_protein	gp344;phage(gi593777800);PHAGE_Bacill_G_NC_023719 3.33e-76 MTTPTTVRDLIIVGSGPAGYTAGVYAARAELEPLLFEGTQFGGSLMTTTEVENFPGFREGIIGPDLMDEMREQAKRFGADIRTEDVEEIVLDGPVKKVVANGETYYARAVILAMGAAARYLGIPGEERLLGRGVSACATCDGFFFRDQDIVVVGGGDSAMEEATFLTKFARSVTLVHRREEFRASRIMLERAKANEKIRFVTNAEPVEVLGENSVTGLVIRDTVTGEQSTLDVTGMFVAIGHDPRSELVKGQVDVDDAGYVKVQSPTTATSAAGVFAAGDLVDHTYRQAITAAGTGCSAAIDAERWLADQGDITDNTLAAAGEPVAVDA
NBRC101255_03956	4171232	4171555	+	thioredoxin;15145|vfid|24343|vsiid|45163|ssid|SubName: Full=Thioredoxin;		incomplete	Phage-like_protein	putative host-like protein;phage(gi658311036);PHAGE_Dinoro_DFL12phi1_NC_024367 3.52e-12 MANTITINDDSFKQDVLDAEKPVLVDFWATWCGPCKMVAPVLEEIAGEHGDKLTIAKLDIDQNPGAARDFQVMSIPTMILFQGGKPVKTIVGAKGKAALLKDLADVL
NBRC101255_03957	4171698	4172900	+			incomplete	Phage-like_protein	endolysin;phage(gi557478866);PHAGE_Bacill_CampHawk_NC_022761 1.69e-10 MQLLRHGDHGPAVAEIRGTLTGLGFLHNGVAETQRESVNGSHWVSPDAMFDHHLDSAVRAFQQQRGLLVDGIVGPATYRSLKEASYRLGARTLIYQLSAPLYGDDVAALQTRLQDLGFYVGRVDGYFGPKTHEALSSFQREIGIAADGICGPATLRSLELLGTRVSGGSPHAISEEELVRRSGPQLSGKRIVIDPGLGGADTGLVVQTPNGPVSEADILWDLASRLEGRMAATGMETFLSRPQHANPSEAERAETSNGFDADLMIALRCDSHSSPAASGVASYHFGNSHGSTSMIGQILTGFIQREIVARTPLRDCRTHGRTWELLRLTKMPTVQVDIGYLTNESDVRVLTDSRSRDTIAEAILIAVKRLYLLGQDDAPTGTFTFAELLAEELSSAPQAR

NBRC101255_03958	4172950	4173639	-			incomplete	Hypothetical_protein	hypothetical protein N/A MSTSVTSLTLDGLDKLSSHARRCVFWETDPAAVRAARETGNFYDPEFEKEAWLSMVMLQWGSCGQVAMVDDKPAGCALYAPPSMVPRADLFPTSPVSADAVLLTTMRLEPIGDEHGLGATLIQAAVGDLVRRGVRALEAFGIRGEAPSDVPTATAALDCSPQECMISADFLEDVGFEVIAPHHRFPRLRLELDRDHLWKADVEAALDRLLEVAALSMLDIDEKSPVAVR
NBRC101255_03959	4173874	4175025	-			incomplete	Hypothetical_protein	hypothetical protein;phage(gi28876350);PHAGE_Strept_315.4_NC_004587 4.04e-08 MAQTRKGGLGRGLAALIPTGPAGGGPGLGTAAADVIIGTSAPTSDAAAVETAADTAVETEAPAKASKPAKKASAKAPAKAASKTAAKAASSDDVVATAPTPAPAVRTDGEPLSPTGAVYHEIPPLQIQPNPKQPRSVFDEEALGELVHSIREFGLMQPIVVRRIGPEQYQLVMGERRWRASQLAGLETIPAIVRETTDDSMLRDALLENIHRVQLNPLEEAAAYQQLLEEFEVTHEELASRIGRSRPVVTNMIRLLKLPIPVQRRVAAGVLSAGHARALLSLEAGADAQEVLAARIVAEGMSVRATEEAVMLANREGPSPTPPVKRKPIQMPGLQDVAERLSNSFDTRVTVSLGKRKGKITVEFGSVDDLERIVKMMEQQAGS
NBRC101255_03960	4175027	4176034	-			incomplete	Phage-like_protein	sporulation initiation inhibitor protein Soj;phage(gi589892278);PHAGE_Mycoba_Adler_NC_023591 1.73e-17 MSAGSESAVSRETVSRETSTSSDAPNGGSGDGYAHSPYSGISLADTPIGAAAQRASQVLHPGSVSLPRPPERRVFTIANQKGGVGKTTSTVNLASALAIQGLTVLVVDLDPQGNASTALGVPHTSGTPSSYELLLGEVTAKEAIQQSPHNERLYCIPATIDLAGAEIELVSMVARENRLKNALSEKALADLDFDFILIDCPPSLGLLTVNAMVAAKEVLIPIQCEYYALEGVGQLLRNIELVQAHLNPDLHVSTVLLTMYDARTKLADQVAEEVRKHFGDVVLRAVIPRSVKVSEAPGYGMTVLDYDPGSRGAMSYLDAGRELAARSMSTNTQEQ
